# Stakeholders’ Perceptions on Shortage of Healthcare Workers in Primary Healthcare in Botswana: Focus Group Discussions

**DOI:** 10.1371/journal.pone.0135846

**Published:** 2015-08-18

**Authors:** Oathokwa Nkomazana, Robert Mash, Sheila Shaibu, Nthabiseng Phaladze

**Affiliations:** 1 Faculty of Medicine, University of Botswana, Gaborone, Botswana; 2 Division of Family Medicine and Primary Care, Stellenbosch University, Cape Town South Africa; 3 School of Nursing, University of Botswana, Gaborone, Botswana; University of Geneva, SWITZERLAND

## Abstract

**Background:**

An adequate health workforce force is central to universal health coverage and positive public health outcomes. However many African countries have critical shortages of healthcare workers, which are worse in primary healthcare. The aim of this study was to explore the perceptions of healthcare workers, policy makers and the community on the shortage of healthcare workers in Botswana.

**Method:**

Fifteen focus group discussions were conducted with three groups of policy makers, six groups of healthcare workers and six groups of community members in rural, urban and remote rural health districts of Botswana. All the participants were 18 years and older. Recruitment was purposive and the framework method was used to inductively analyse the data.

**Results:**

There was a perceived shortage of healthcare workers in primary healthcare, which was believed to result from an increased need for health services, inequitable distribution of healthcare workers, migration and too few such workers being trained. Migration was mainly the result of unfavourable personal and family factors, weak and ineffective healthcare and human resources management, low salaries and inadequate incentives for rural and remote area service.

**Conclusions:**

Botswana has a perceived shortage of healthcare workers, which is worse in primary healthcare and rural areas, as a result of multiple complex factors. To address the scarcity the country should train adequate numbers of healthcare workers and distribute them equitably to sufficiently resourced healthcare facilities. They should be competently managed and adequately remunerated and the living conditions and rural infrastructure should also be improved.

## Background

‘Health workers save lives’[1] and primary healthcare (PHC) holds the key to improving access to healthcare for underserved communities and reaching the Millennium Development Goals [2]. Severe shortages of healthcare workers (HCWs) have, however, hampered the efforts of many low-to-middle-income countries in providing universal PHC [3, 4]. Promoting the retention of HCWs and developing effective systems for PHC delivery will facilitate improvements in community health and equity, and potentially reduce the total cost of healthcare services [2].

A well performing health workforce is one of the six pillars of strong and effective healthcare systems [5]. There is, however, a documented global shortfall of over two million doctors, nurses and midwives, and 36 of the 57 countries with a critical shortage of HCWs are in sub-Saharan Africa. In addition, sub-Saharan Africa, with only 1.3% of the global health workforce, bears 25% of the global burden of disease, dominated by infectious diseases [1, 6]. The shortage of HCWs is complicated by inequitable distribution, skill mix imbalances, negative work environments and a weak knowledge base [6].

The causes of shortages in human resources for health are many and complex. Effective mitigating strategies should therefore be comprehensive and context-specific and derived from an adequate understanding of the context [7].

Botswana is an upper middle income country with relatively high expenditure per capita on health [8] and a destination for many health professionals, especially from sub-Saharan Africa [3]. The country therefore has more health workers per population than many countries in the region [8]. That, notwithstanding, the country has a documented deficiency of healthcare workers which hinders it from providing universal primary health care [8, 9].

Although Botswana has a recognised inadequacy of human resources for health, which is worse in primary healthcare, [9], no research has been done to investigate its determinants. Therefore, the qualitative research reported in this article explored the perceptions of HCWs, policy makers and members of the community on the shortage of HCWs in Botswana, and to identify the perceived causes and potential solutions. Based on the proposed solutions, an intervention will be developed and piloted in one health district.

## Method

A descriptive qualitative design [10] was used to explore the perceptions of policy makers, healthcare workers and members of the community on the shortage of HCWs in PHC in Botswana. The checklist for consolidated criteria for reporting qualitative research guided the research process [11].

### Setting

The study was conducted in 3 of Botswana’s 28 health districts, namely Gaborone, an urban health district, Mahalapye sub-district, with headquarters in Mahalapye, a rural health district and Ngamiland, with headquarters in Maun, a rural and remote district.

Botswana has a population of just over 2 million, 59.4% of whom live in urban areas [12]. The healthcare system is based on a primary healthcare model and services are provided through a network of 3 national referral hospitals, 7 district hospitals, 14 primary hospitals, 265 primary care clinics (101 with maternity beds), 343 health posts and 861 mobile clinic sites [9]. Management of the health services, including recruitment and deployment of HCWs, procurement and distribution of equipment and drugs, is centralised at the Ministry of Health headquarters. District health management teams (DHMTs) were created in 2010 to manage healthcare services in their respective districts. The DHMTs work closely with the tribal, political and executive leadership of their districts to enable inter-sectorial coordination of services.

Until 2007, training of doctors, dentists, laboratory scientists, pharmacists, physiotherapists and other allied health professionals was outsourced to universities in the region and overseas. The training of doctors and laboratory scientists was localised in 2009 through the creation of the University of Botswana Faculty of Health Sciences in Gaborone.

### The study design

Fifteen focus group discussions were facilitated with policy makers, HCWs and members of the community from March to May 2012 in all three districts chosen for the study.

### Sampling strategy

Separate meetings were held with policy makers, HCWs and members of the community in January and February 2012 in each of the three districts to recruit participants for the interviews. In Gaborone only one combined meeting was held with all groups. The following criteria were used to purposively select people to attend the meetings:

1. Policy makers were defined as administrative, political, or traditional leaders in the districts whose work had an impact on healthcare. District executive managers (district commissioners) and DHMTs were invited as administrative leadership in the district. The DHMTs included senior doctors, nurses, other HCWs and health administrators in the district. In Gaborone, the capital city, the national human resources manager and primary healthcare coordinator were also invited. Village health committee, village development committee and district council chairpersons were invited as political leaders and local tribal chiefs as traditional leaders. They were included as policy makers and not in the community groups as they are part of the district inter-sectorial leadership. All of the designated people above were invited to the meeting.
2. HCWs were defined as public employees involved in primary healthcare. These comprised doctors, dentists, nurses, community health workers, lay counsellors, pharmacy technicians, laboratory technicians, health education assistants and family welfare educators. Invitations were sent to all the primary healthcare facilities in the district.
3. Members of the community were defined as people that had lived in the district for at least 6 months. Invitations were extended to those in organised community groups and structures such as village health committees, home-based care volunteers, and village leadership (including village development committee members and village headmen). Invitations were sent to all villages in the district.

**Fig 1:**
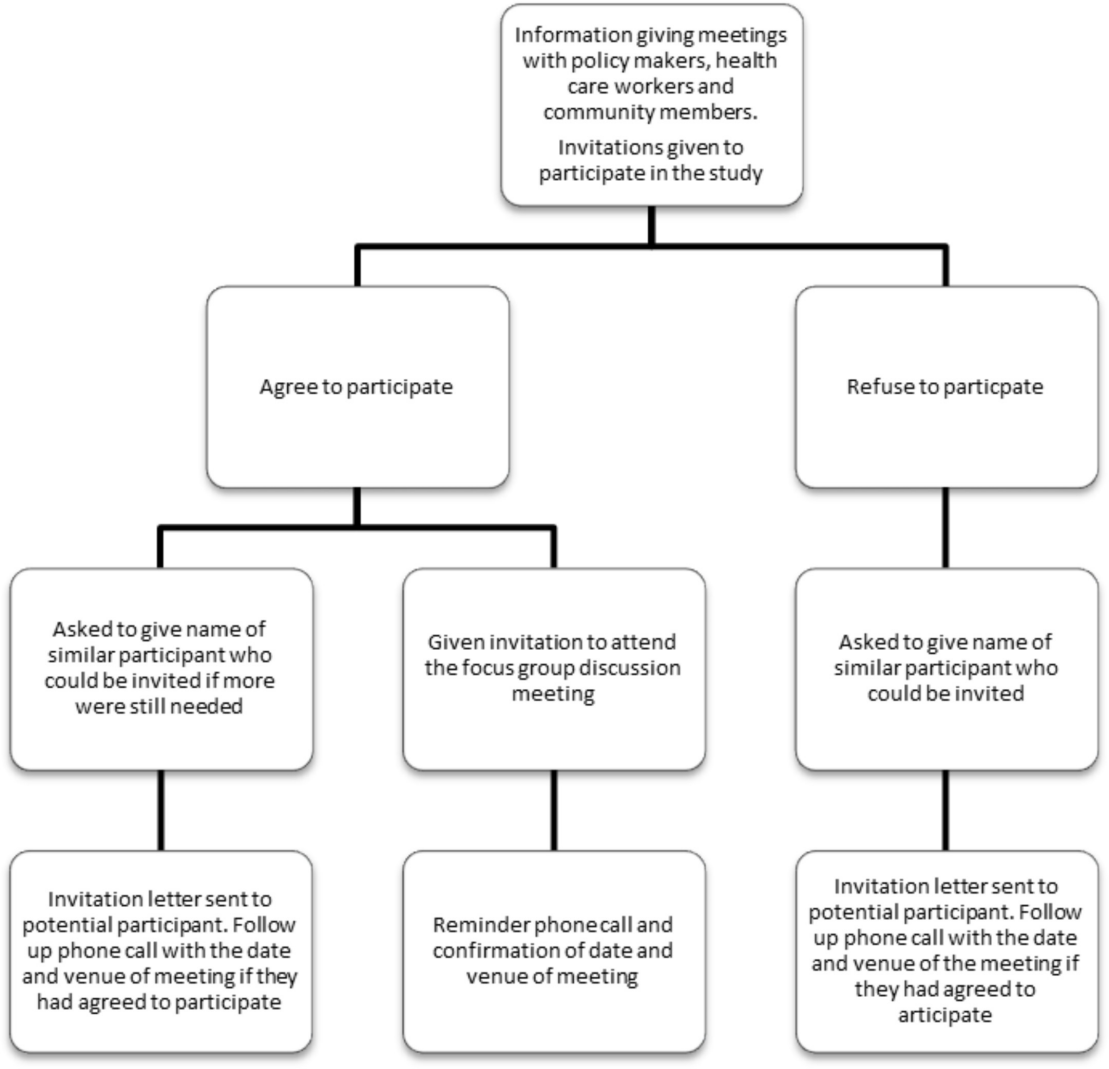
Sampling Strategy.

At the meeting the study was explained and all attendants were invited to participate. All attendants were asked to identify additional suitable people that could be invited should more be needed for the interviews or who could replace them should they be unwilling or unable to participate (Fig 1).

### Data collection process

A series of 15 focus groups, comprising 5 to 12 participants each, were conducted in the three districts. Each district had one focus group with policy makers, two with HCWs and two with community members. We conducted the interviews using a semi-structured interview guide. Each focus group was held in a quiet and private space and lasted between 2 to 3 hours. The groups discussed the following questions:

1. In your opinion, is there a shortage of healthcare workers in primary healthcare in Botswana?
2. What do you believe are the causes of any shortage?
3. What do you think can be done to address any shortage of healthcare workers in primary healthcare?

The interview guide was piloted with members of the university community (HCWs and support staff) and revised based on their feedback. The discussions with community members were in Setswana (the national language of Botswana) and the other group discussions used both Setswana and English. ON (female, MD) facilitated 12 of the 15 interviews while NP (female: PhD, Nursing) and SS (female, PhD, Nursing) facilitated the remaining three. The discussions were audio-recorded and transcribed verbatim. All the members of the team were trained in qualitative interviewing and were bilingual (English and Setswana). The Setswana documents were translated into English before analysis.

### Ethical considerations

Ethical clearance to conduct the research was obtained from the University of Botswana Institutional Review Board and the Ministry of Health Research and Development Unit: Reference No: PPME 13/18/1 V11 (368). Written informed consent was obtained from each study participant.

### Data analysis

Data analysis was performed using the framework method [13–15]. ON developed a thematic framework, with supervision from RM, using two of the transcripts. ON then applied the index to code all the remaining 13 transcripts. The themes were derived inductively from the data. ATLAS.ti software (version 7.1.3) was used to manage the transcribed data and to expedite data analysis. No new themes were identified after analysing the first seven transcripts; nevertheless, all the transcripts were analysed. The number of times that a code, relating to particular theme, was used in the 3 groups was also tabulated to indicate the relative strength of the item in the respondent groups. The emerging themes were also presented to and discussed with the different participant groups for respondent validation [16].

## Findings

Fifteen focus group discussions (Table 1) were conducted with a total of 133 participants: 46 HCWs, 27 policy makers (PMs) and 60 community members (CMs). The average length of service of HCWs at the districts was: Gaborone (G) 13 years, Mahalapye (MH) 10 years and Maun (M) 8 years. In the quotes below the voice of the participant can be identified by using the abbreviations above for the district and type of participant.

**Table 1:** Membership of the policy makers and healthcare workers’ focus groups.

Cadre	Policy makers N = 27 (3 females)	Healthcare workers N = 46 (36 females)	Community members N = 60 (50 females)
Doctor	8	8	0
Nurse/midwife	4	21	0
Administrator	7	0	0
Other health worker	2	15	1
Community/political leaders	6	0	1
Civil society organisations	0	0	2
Home-based carers	0	0	6
Traditional healers	0	0	1
Other community members	0	0	49

The themes that emerged from the data are presented in three sections below as they relate to the perceived shortage of HCWs in PHC, the reasons for shortages of HCWs and suggested strategies to rectify the situation.

### Shortage of healthcare workers in primary healthcare

All the groups believed there was a shortage of HCWs, more especially of Setswana-speaking doctors, midwives, and specialist nurses (Table 2):

**Table 2:** Frequency of coding for a perceived shortage of healthcare workers.

Themes	Community members	Healthcare workers	Policy makers
No shortage of HCW	4	2	2
Shortage in rural and remote areas	12	2	4
Shortage of Tswana speaking doctors	11	3	1
Shortage of doctors	7	3	1
Shortage of health-care workers	16	14	12
Shortage of nurses	7	1	0
Shortage of midwives and specialist nurses	15	10	11
Shortage of pharmacy technicians	3	2	1
Shortages of other healthcare workers	3	7	2

*‘… doctors are not enough … you can go to the clinic … when you get there they will tell you he will come on Friday while you are sick on that day …*.*’* (GCM1).[S9 Text]

*‘… most of the time we meet only foreigners*. *When he consults with me what can I say since I do not know English*? *And if there is no nurse you are in trouble*.*’* (GCM2)[S9 Text]

The community perceived a greater scarcity of HCWs in rural and remote areas:

*‘… I think that there is shortage*, *especially at faraway places*, *deep in the rural areas*.*’* [GCM3][S9 Text]

Conversely a smaller dissenting voice from the three groups denied there was a shortage, blaming the inadequate health services on idleness, poor morale and inequitable distribution of HCWS:

‘… *there are enough*, *the problem is laziness that is too much … instead of starting at half past seven*, *the doctor will show up … around eleven … results in people becoming too many until the sun goes down* …’ (MCM1)[S4 Text]

### Reasons for the scarcity of healthcare workers in primary care

Increased need for health services, inequitable distribution, migration, and training inadequate numbers were blamed for the deficiency of HCWs in PHC (Table 3).

**Table 3:** Frequency of coding related to the causes for a shortage of healthcare workers.

Category	Community Members	Health Care Workers	Policy Makers
Increased service need	4	2	6
Inequitable distribution	9	14	4
Migration from public sector	23	28	10
Training of health workers	35	40	37

The participants argued that population growth, the HIV/AIDS epidemic, and increasing prevalence of non-communicable diseases led to the establishment of new clinics and services, without equivalent increases in the number of HCWs:

‘… *when we plan to build extra health facilities*, *we don’t come up with extra nurses …’* (MHCW1) [S1 Text]

‘… *the epidemic of HIV and other diseases have gone beyond what we even expected to have at this time* …’ (MPM3) [S5 Text]

HCWs and the community were particularly unhappy about the unfair distribution of HCWs, which favoured towns and large villages:

*‘… mostly it’s to do with the distribution … they tend to focus mostly on the cities … and forget the rural areas* …’ (MHCW2)[S1 Text]

Nonetheless, some HCWs alleged that allocation was reasonable, but there was higher attrition in rural areas:

*‘… they are distributed equally*, *but … they leave the service in high numbers … in rural areas … compared to urban areas*.*’* (GHCW1)[S7 Text]

The problem of training inadequate numbers of HCWs was reported by all the groups, who also claimed that those trained in other countries did not return to work in Botswana:

*‘… research … revealed … that at the rate at which Botswana was training doctors … it would take … 150 years to have adequate numbers … people sent abroad to study … do not even come back* …’ (MHCW3)[S14 Text]

On the contrary, some community members claimed that adequate numbers of health workers were trained, but not retained:

*‘… [there] are many institutions that produce nurses … every year … let’s just say they are not happy [and leave]* …’ (GCM5)[S9 Text]

At the same time HCWs and policy makers were concerned that training of HCWs was not always informed by Botswana’s health system needs:

*‘… the government will allow me*, *if I choose [to] study community health nursing*, *not to do midwifery … while we don’t have enough midwives*.’ (MHCW4)[S1 Text]

*‘… but I think the training is too focused on curative medicine … the primary health component of healthcare is being eroded … But … our kind of problems can be best addressed at primary healthcare level …’* (MPM1)[S5 Text]

Migration of HCWs from the public health system was held as a worrisome contributor to HCW scarcity in rural areas by all the groups (Table 4). The migration was mainly to private health services, to urban areas, and to other countries:

‘… *they leave us here and look for jobs in private companies* …’ (GCM6) [S9 Text]

‘… *most of the nurses migrated to European countries around 2000 or 1999 … saying they were going to greener pastures* …’ (GHCW2)[S7 Text]

**Table 4:** Frequency of coding for the reasons underlying the migration of healthcare workers.

Category	Community members	Healthcare workers	Policy makers
Poor healthcare management	98	85	60
Poor human resources management	63	104	83
Lack of incentives for primary care	12	29	30
Personal and family factors	7	18	12
Socioeconomic and contextual issues	37	32	39
Poor working conditions	23	31	9

The study participants acknowledged that ineffective healthcare and human resources management, inadequate incentives, personal and family issues coupled with socioeconomic and contextual challenges contributed to the movement of HCWs from PHC.

Healthcare management was deemed weak and ineffective by all the groups. This resulted in demoralising working environments characterised by poor infrastructure and absence of critical resources such as patient transport, medicines and equipment:

*‘… there are no tablets*. *Daily you see sick people … as a human being this depresses you … there is not even a car to take that person to the next clinic* …’ (MPM3)[S5 Text]

*‘… you don’t have equipment and drugs … how are you going to work …*?*’* (GHCW4) [S7 Text]

Human resources management practice was a matter of concern for all the groups, especially HCWs and PMs, who maintained that policies on the length of rural deployment, transfer, promotion and continuing professional development were unclear and not always informed by the need for health services:

*‘… whenever a midwife is transferred out of Maun … they would take junior nurses who have just finished from [school] and send them as a replacement …’* (MPM3)[[S5 Text]

*‘… imagine somebody of my age just going for a degree [course] after 25 years of service the nomination of people for further studies … you don’t know which criteria are used because people who are long in service are left [overlooked] … and those newly qualified are the ones who are nominated to go for further studies …’* (MHCW3).[S1 Text]

These policies were also inconsistently and unfairly applied and often favoured those working in hospitals:

*‘… you find that … more health workers working at the hospitals are promoted unlike in clinics …’* (GHCW5)[S7 Text]

Additionally, a lack of supportive supervision was of particular concern to HCWs who were generally demoralised by the lack of appreciation, recognition and rewards:

*‘…how are you going to be seen that you are doing well when they visit you once in six months … and all they hear is when you are referring [accompanying] a patient to Princess Marina hospital and someone complains that there is no nurse in the clinic*. *How are you going to be on the list of people who are rewarded*?*’* (GHCW1)[S7 Text]

The community, on the other hand, blamed ineffective supervision for the tardiness of HCWs:

*‘… the leadership is supposed to take action*, *against nurses or doctors who refuse to see patients at night … but … they are reluctant … there is lack of supervision …’* (MCM5)[S4 Text]

Furthermore, all the groups were worried about the demotivating practice of posting junior and inexperienced single nurses to clinics, without the possibility of adequate supervision and support:

*‘… young nurse*, *who just [graduated] from school … you are sending that particular person to the health post [to work] alone …’* (MHCW4)[S1 Text]

The participants decried the non-competitive remuneration packages and the absence of incentives for rural and remote service:

*‘… the government has no incentives for the people who are staying in very remote areas … they end up being demoralised …’* (MHCW3)[S1 Text]

Policy makers intimated that loneliness, lack of psychosocial support and recreational facilities, in rural and remote areas, increased the risk of depression, substance abuse and attrition of HCWs:

*‘… it is so depressing that people can find refuge in alcohol and drugs in those areas*.’ (MPM3).[S5 Text]

All the groups reported special difficulties that were encountered by HCWs who were separated from their families when deployed elsewhere:

*‘… the government doesn’t recognize the social needs of workers … you will stay there*, *a distance of a thousand and something kilometres [from your spouse] … you end up resigning’* (GHCW5)[S7 Text]

*‘… a lot of divorce*! *Even if you don’t divorce you are not connecting well …’* (MHCW7)[S1 Text]

All the groups indicated that inadequate rural infrastructure such as roads, telecommunication, electricity, running water, shops, accommodation and schools for children, were reasons for the increased loss of HCWs in rural areas:

*‘… some people are not coming because of the school for the kids … they wanted their kids to study in English [medium] school …’* (MHHCW3).[S11 Text]

*‘… frustration in these people who are in this primary healthcare is a lot … poor accommodation … no electricity … they cook what they are going to eat now*, *because they cannot keep it for tomorrow*.*’* (MHPM7)[S3 Text]

Likewise they viewed the deficient rural infrastructure as an impediment to continued professional development and further education:

*‘… where there is no electricity you can’t do anything to further your studies …’* (MHHCW2)[S11 Text]

#### Proposed solutions for the scarcity of healthcare workers

The research participants advanced a number of potential solutions to the shortage of HCWs in PHC. These propositions mirrored the reported determinants for the shortage of HCWs.

The participants stressed the importance of tackling staff welfare problems, which should include keeping families together as much as possible:

‘…*those who are married … ensure that they have easy accessibility to having a good family … staying together* …’ (GHCW2)[S7 Text]

All the participants wanted the resource deficiencies to be addressed urgently:

*‘… There is use of cell phones nowadays; there has to be an arrangement where you can pick the cell phone and talk to a doctor … about the patient*. *Now you find that there is no such facility and you end up using your own cell phone which is wrong*.*’* (GHCW4)[S7 Text]

In addition, policy makers and HCWs desired the decentralisation of healthcare management to the districts or at least to allow for district specific health service delivery models:

*‘… maybe the government should come up with flexible or area specific models of service delivery …* (MPM3)[S5 Text]

The participants recognised that the socioeconomic obstacles to retention of HCWs could not be tackled by the Ministry of Health alone, but required a concerted multi-sectorial approach. However, they wanted appropriate accommodation to be provided to HCWs as a matter of urgency:

*‘…sometimes you may find that an issue is beyond the scope of … the ministry … example of electricity maybe or telephone … water … so if these resources are not available in the clinic you may have the best nurse and all the drugs*, *but find a fridge does not work … [inter-sectorial] coordination is very important*.*’* (MHPM1)[S3 Text]

Policy makers and HCWs contended that there should be more incentives for HCWs working in rural and remote areas:

*‘… but if people in remote areas were given some incentives … that would help*. *We are not only talking about monetary incentive but … about other things like housing …’* (MPM1)[S5 Text]

Furthermore, they argued that differential incentives should be given to midwives and nurses with additional qualifications or who perform non-nursing duties:

*‘… government should start giving incentives for some of these courses*, *like midwifery*. *Midwifery … is a high risk job …’* (MHCW5).[S1 Text]

The participants proposed that human resources management policies on promotion, transfer, length of service in rural areas and in-service training needed to be clarified and applied fairly, consistently and transparently. They also insisted that HCWs should not work in rural areas for a very long time. In addition, they recommended that the career structures for nurses be reformed:

*‘… you need to make a clear policy of transfers*, *because if you send someone to a rural area he has to know and be prepared that I’m going … to provide service [for] two years …’* (MHCW3)[S1 Text]

HCWs also wanted their supervisors to visit them regularly to appraise their work accurately, understand their work challenges and reward them appropriately:

*‘… how do we reward people for putting in long hours and working so hard …your employees when they’ve done a good job*, *do you congratulate them or do you always look at the bad things …’* (GHCW6)[S7 Text]

All the participants requested role clarification and adequate supervision, especially for junior and inexperienced staff.

The participants advocated for differential increases in the numbers of HCWs trained, based on the healthcare needs of the country. They also proposed that training should instil appropriate knowledge, skills and attitudes:

*‘… midwifery should be a compulsory course [for nurses]*. *I think also the government should strike a balance between all the post basic [advanced nursing] courses*.*’* (MHCW4)[S1 Text]

*‘… During training they can do their practical in rural areas*. *So they can be familiar with rural experience …’*. (MHPM2).[S3 Text]

## Discussion

To our knowledge this study is the first to explore the perceptions of policy makers, HCWs and the community on human resources for primary healthcare in Botswana. The number of HCWs in PHC were perceived to be inadequate, a perception that is confirmed by existing evidence [9]. That notwithstanding, Botswana, an importer of healthcare workers from other countries [3] has more healthcare workers per population than many low and middle income nations [8].

Like many other African countries, Botswana is perceived to be failing to produce enough health professionals for its health systems [17, 18]. This situation has been exacerbated by failure to retain those that were trained in the public health system [9, 19].

Botswana’s situation has been further complicated by its policy of outsourcing the training of many health professionals to other countries, with the result that the majority have remained in the host countries after graduation [9]. There is hope, however, that the opening of a medical school and a faculty of health sciences for the training of doctors and other HCWs at the University of Botswana will address this challenge. Local production of HCWs in Botswana, as in many other countries in sub-Saharan Africa, is however hampered by the limited of capacity of training institutions. [17, 18, 20]. The policy makers and healthcare workers were of the opinion that the training of the healthcare workers was not informed by the needs of the health system.

Inequitable distribution, favouring hospitals and urban areas, and higher attrition rates in rural areas have resulted in fewer HCWs in PHC and rural areas [9]. This ‘inverse care law’ [21] besets many health systems, especially in low-and-middle-income countries [1, 22, 23]. Similar to other southern African countries, Botswana’s need for HCWs has also grown, primarily because of the HIV/AIDS epidemic, which has placed increasing demands on the healthcare system [1, 24].

Despite its being a destination of migrant health workers from the region [3,9], Botswana suffers from both internal and external migration of HCWs, a problem which plagues many low-and-middle-income countries [25, 26]. The determinants of migration are multiple, but personal and family issues stand out as a priority. Living away from the family is significantly associated with attrition of HCWs from PHC and rural areas [22]. HCWs also migrate because of a dearth of opportunities for career progression and continuing professional development, especially in rural areas [19].

Weak and sometimes incompetent healthcare and human resources management, characterised by lack of essential resources for patient care, poor career structures, unclear policies, absence of supportive supervision as well as poor conditions of service, were identified as ‘push factors’ for HCW migration. This finding is in keeping with studies on migration of African HCWs [19, 27, 28].

HCWs are also reluctant to work in rural areas because of poor infrastructure such as roads, schools for children, accommodation and telecommunication, and limited opportunities for career and continuing professional development. Deficient development has also been blamed for the failure of many countries to staff rural healthcare facilities adequately [7, 22, 23].

Incentives and remuneration packages were deemed unattractive and non-competitive compared to those in Namibia and South Africa. Inadequate incentives and low salaries are known to be strong ‘push’ factors for migration of health professionals [7, 23]. Conversely, the hope of ‘greener pastures’ is a ‘pull’ factor to high-income countries.

Our study suggested that in order to tackle staffing insufficiency in PHC, multiple barriers must be addressed simultaneously [28, 29]. Few such strategies have been evaluated in Africa, but Ghana’s monetary incentives and Zambia’s more comprehensive strategies have had some success [23, 25].

Interviewing the three groups from three different settings enabled the exploration of different perspectives, varying experiences of the healthcare system and triangulation of themes. The study was conducted in three of the 28 health districts only and it is possible that other viewpoints would have been obtained elsewhere. Given the consistency of responses and saturation of themes, as well as the exposure of respondents to other districts in previous postings, we believe the results are transferable and likely to be replicated throughout Botswana.

### Implications and recommendations

Various recommendations can be made based on this research, as suggested below.

Capacity should be built in human resources for health, healthcare management and systemic monitoring and evaluation. Botswana should also institute strategies to train and retain more healthcare workers. The training should be informed by needs of the health system and also inculcate appropriate competencies [30]. The retention strategies should include better incentives including fiscal, accommodation, continuing professional development opportunities, and improvement of rural infrastructure. Moreover, the capacity of DHMTs to provide supportive supervision to HCWs in PHC should be strengthened.

## Conclusion

Botswana, despite its relatively high expenditure per capita on health [8], has a shortage of healthcare workers, which is worse in primary healthcare and rural areas, as a result of multiple complex factors. The causes of the deficiency of health workers are similar to those of countries which spend much less on health, some of which are also sources of the Botswana health workforce [9, 23]. To address the scarcity the country should train adequate numbers of competent HCWs and distribute them equitably to sufficiently resourced healthcare facilities. These HCWs should be competently managed and adequately remunerated and the living conditions and rural infrastructure should be improved.

## Supporting Information

S1 Text Maun Health Workers group 2_File.pdf. (PDF)

S2 Text Mahalapye community group 2_File.pdf. (PDF)

S3 Text Mahalapye policy makers_File.pdf. (PDF)

S4 Text Maun Community members group 1_File.pdf. (PDF)

S5 Text Maun policy makers_File.pdf. (PDF)

S6 Text Gaborone policy makers_File.pdf. (PDF)

S7 Text Gaborone health care workers group 1_File.pdf. (PDF)

S8 Text Gaborone healthcare workers group 2_File.pdf. (PDF)

S9 Text Gaborone community group 2_File.pdf. (PDF)

S10 Text Gaborone community group 1_File.pdf. (PDF)

S11 Text Mahalapye healthcare workers group 1_File.pdf. (PDF)

S12 Text Mahalapye community group 1_File.pdf. (PDF)

S13 Text Mahalapye healthcare workers group 2_File.pdf. (PDF)

S14 Text Maun healthcare workers group 1_File.pdf. (PDF)

S15 Text Interview guide for policy makers and healthcare workers_File.pdf. (PDF)

S16 Text Interview guide for community members_File.pdf. (PDF)
