# Supplementary material for: Stakeholders’ Perceptions on Shortage of Healthcare Workers in Primary Healthcare in Botswana: Focus Group Discussions
Source: PLoS One. 2015 Aug 18;10(8):e0135846. doi: 10.1371/journal.pone.0135846 (PMC4540466; doi:10.1371/journal.pone.0135846)
Supplement: S1 Text — (PDF) [file pone.0135846.s001.pdf]

A qualitative study of Human Resources for Primary Healthcare (HURAPRIM) in Botswana (Focus Group Discussions)

Date: 25/04/2012

Interviewer: D

Interview Duration: 02.31.54

Audio File Name: Health Care Workers 2 (Maun)

HURAPRIM (Health care workers 2)

Int: Umh! may we continue with the discussion...?i am going to ask you the questions about the healthcare workers in your area, there is no wrong or right answer,please answer each question in detail, your truthful answer will help the ministry of health to understand the health care workers situation in the country. The first question is....it's what your understanding of primary health care is, what do you understand by primary health care? Anyone to break the ice?

Part1; it is the care that is given to the community to…. at their respective areas and this care should be affordable, accessible... acceptable…. and the others, A wa bohelo!(The last A)

Int; Ok! Umm...Anyone else? What does somebody else….!!!!!Anything different that we could add?

Part2; And it should be tailored...Let me put....it should be tailored around the the the traditions and customs of that community so that it doesn't......so ga di gotlhagotlhane

Int; Ee P2!!! Thank you, ee P3! You were going to add!!!

Part3; Ee! In addition P2 has already said, this is the first hand, the first hand care that the client receives from the health facilities and basically is the care that people will be able to access, that they will able to afford, to accept, you know there are some cultural believes tse e leng gore sometimes some people are not allowed to accept or access health facilities. But with primary health care we are looking to the the four eh…

factors which are in line with that particular care that is the accessibility, affordability eh…acceptability... and acceptability<br><br>Int: okay thank! You anything else!!Okay I am going to ask the second question? Do you think there are enough or not enough health care workers in Botswana? And if they are not enough, why do you think this is so? Like For example, are there adequate numbers trained, first of all lets answer, do you think they are enough?<br><br>Part4; The health care workers are not enough, they are not enough!!!Mmm<br><br>Int: they are not enough, okay! What does somebody else say? Do you think they are enough health care workers in our country? Ee!<br><br>Part5: They are not enough<br><br>Int: They are not enough? Ee P6! Yes!<br><br>Part6: i think they are not enough!!<br><br>Part 7; So generally we agree that they are not enough!! Yah!<br><br>Int; Ooh! It's a consensus we all agree that they are not enough, and if you think they are not enough why do you think is so, why are they not enough!!Why are they not enough? Wat do you think? Ee P3!<br><br>Part3: One reason could be that...is that for a long-time our our country has been lacking behind as far as training of some of health workers is concerned the...meaning doctors, the physiotherapy and others so we have been concentrating on training nurses alone<br><br>Int:mmh<br><br>Part 3;Ee<br><br>Int: Ok! Yes umm...anybody else like, what about ummm...deployment and distribution and the way they are distributed? And deployment?<br><br>Part8:i think if there is an imbalance in terms of distribution of health care workers	

because if you look in Gaborone you will find that a hospital like princess marina is overstaffed and certain clinics or hospitals in district areas and remote areas they lack healthcare workers therefore, yah! There is a 05;16………of skills

Int: okay!! Umm! What about the issue of retention? Retention!!!Retaining staff.

Part1: okay!! The retention is also poor because… if i may I may give an example, i have been working in the local government for the past 25years and i have realis…and i have been working for 22 years I have been working in north west district council.So what i have realised during my stay here is that most of the the the health workers being posted to northwest ….and that was the time when northwest was Okavango,ngami and Chobe, so those health workers who were posted here or transferred to northwest, most of the times we would receive the names and the people will not appear. They will resign before they can even step their foot here. And and the other thing that was alarming us was that when they refuse to come here,they will go back to the ministry and sometimes the same person the ministry would decide to post her somewhere else other than here and the other reason was that when they resign that was the time when we were still… there was still local government primary healthcare and the ministry of health.When they resign from local government, the Ministry of Health will absorb them so we end up empty handed from the local government when the ministry the ministry of health is getting saturated with the health care workers

Int: Mmm! EeP1!!!! Anything else!!! Anything else!!! We could add to that like!!!!! Okay ee P2!!!!

Part2: we are also finding it a lot that the reason of people going and leaving the health offices in the country. Resignations, attrition…is is is really high, mmh!!!!!!

Int: okay!!!! People are resigning.What the issue of recruitment? What about recruitment for health care workers in primary, it it contributing to them not being enough. Are they being recruited? Is enough being recruited?

Part3: I think when you talk about recruitment we we evoke another factor that is very important which is remuneration, eh you find that the other reason people don't want

to come which is to to come is because of the pay like he has been talking about people that are leaving the country like why are they leaving the country, uh..Do they having enough salaries to sustain themselves and uh... those kind of provisions<br><br>Int: okay!!!! Okay!!! I think we exhausted that one let me...oh!<br><br>Part4: Sorry!(jumps in) Especially, especially those those professions which are not trained in this country, it very very difficult to recruit them because you have to go outside the country to recruit them and you have to remunerate them better than those outside..... Professional workers than those in the country and this will mostly attract them…. the foreigners to work as health workers in our country....mmmh!!!!!!!!<br><br>Part 3; Very expensive(Jumps in)<br><br>Part 4; So is it become very expensive and it becomes very very difficult to recruit them, to attract them you know.<br><br>Int: am!!! Do you think there are gaps issues or problems related to heath care workers for primary health or not? If there are gaps or problems what do you think are the most important gaps or issues? Which what issue or problems do you think are the most important in primary health care?<br><br>Part5: The...some of the issues is that the government has failed to...to attract health workers so that he can retain them and therefore people are moving out, they are moving out to the private....they are moving out to the other countries,...so people who are... you find that the government has no incentives for the people who are staying in very very remote areas and this people they end up being demoralised because you are put where there is no electricity, there are no better roads, there is really nothing there you can't even send your child to a better school so you find that people are not....people not happy about this and sometimes when you are put in that far far far clinic you find that sometimes they even...whether they forget you that you are there or not because you will stay there for more than 5 years while the other people are staying for bo…for 20 years in Gaborone. So this kind of....even the transfers themselves affect the retention of the workers because if people…if...we we people were you	

were sure gore after 3 years I will be transferred from Bodikwa to Gaborone, i think people.....most of the people would go to Bodikwa. But they go there with the fear that they are not sure when they are going to come back.<br><br>Part6: And the other thing would be lack of defined policies on issues of transfer because you know...for an organisation to run in a smoothly, avoiding a lot of hiccups or a lot of imbalances in the way thing are done, policies define or stipulate what is supposed to be done. Like Part 5 has already sited, if we have a defined policy looking at the high turnover or high retention of nurses in the cities there and then you take for example Okavango or this side going the furthest there.. area there, if the policy is clear on how to transfer of people looking at the duration of how long should you stay there, how long should you move to another area and then....is something se e leng (that) our government or our ministry works on it not gore it will work for others it will not work for others then in that way maybe it can somehow avoid a lot of nurses being placed...or a lot of health workers being placed in one area as compared to the other areas which have little manpower or little resources. So lack of defined policies also is a contributing factor to on how that thing is......<br><br>Int: lack of the defined policies!!!!!! Umm...also...<br><br>Part5; Can you,can you ask your question again i have forgotten it (Laughs)<br><br>Int; Ok! The question was do you think there are gaps issues or problems related to heath care workers for primary health or not? If there are gaps or problems what do you think are the most important gaps/issues or problem? What do you think are the most important if they are there?<br><br>Part 5; The other thing is that…. As, i will talk as if am at local government because am still new at ministry of health. At the local government, when we were in local government you find that we were not treated the same way as the nurses were treated at the ministry of health, ke gore the nurses who were… even though we were in the same class you go to local government and your colleague goes to ministry of health, the person who is at the ministry of health is better than…that than…is looked at by the the employer kana by the government as if he is…she or he is better than you who	

is at local government. And even for the promotions you find that we finished…completed school together same, same month and we graduated same day. The person who is at the ministry of health will be promoted first before you are promoted at local government and… right now we are we are grieving because we have been disadvantaged at the local government…we at primary let me say at primary health care because colleagues even our our juniors who came...who completed after after we have completed they have been long been promoted before us and we are now... after the merging, now we are their juniours, we are...and no one is considering us that that we have been long disadvantaged what can we do about this people? No its its....to them its okay we have disadvantaged them and its ends there so this thing is is demoralizing us…you find out that you are now a junior to a person that was a junior to you when you completed.So this are the things that are demoralizing and i think...i i think..if i remember well there were a lot of nurses and some of the nurses they left when the merging came they t retired those who were ready,due for retirement they decided...who were over 45 they decide to retire and some they still feel like going and am one of those people. I feel like...every morning i feel like going because i feel am so demoralized and I can't work because things are not the right way. If there was a problem that was there, why can't you correct it? So it's like nobody is...doesn't care about that. So this are the...the other thing from the primary health...the other thing...i this one is is affecting the nurses both nurses from the primary health care and the hospital is whereby you find that there are some nurses who have some extra qualifications midwifes, community health nurses, …ophththalamic nurses ,nurse admin and the others. This people when you...when the promotion come, these qualifications...they don't matter to the government. Somebody who is single qualified can even be promoted before you are being promoted and wena you have been...you are working...you are... ke gore o bereka dilo tse dintsi. You are a...you are a nurse, a general nurse and you are a midwife and the other person is a general nurse alone but sometimes you find that this person is promoted a mid wife is promoted and yet the government is expecting this person to operate as a general nurse as a midwife and is high time we are thinking we have been lobbying with the government to say look at..Let's sit down and discuss this because we feel this is a...a qualification on its own	

so we need an incentive of the extra qualification that we are having so that it can maybe pick up our...our morale for those that can be picked up by the money...by the...by the remuneration because sometimes if you are demoralized is not about money alone<br><br>Int: Ee P5! Umm...Does anyone want to add something to that? Ee P5!<br><br>Part5: Ee D! The other issue i will speak of course for health workers but specifically looking at the nursing profession. The other thing is you find that as nurses, you are somewhere out there ke gore is like eh…each and every...you cover each and every profession that is in the health care centre. You are a pharmacy technician, you are a lab-technician, you are a doctor, you are everything so that is why some, who cannot stand that, they will look for greener pastures or go to private sectors where maybe they will get focused on the nursing profession itself. So that is in a way also is demoralising people, it brings productivity down and people are not goin...are not performing as they are supposed to because you find that your hands are full but also the renumeration that you get... is something that cannot take you somewhere. So those extra duties that are there also, they demoralises eh...health care workers as much as encouraging...eh encouraging them to go and look for jobs somewhere else where they can work in a piece of mind with the job that they have been trained for without doing other duties which are actually hampering their profession.<br><br>Int: ee P7!!! You were going to say...............<br><br>Part7: ee D! Yah!!! I was going to say... (Clears throat)!!!!! I think… (Clears throat) because we have more nurses here am just gona try and speak in for doctors. I think the reason why doctors choose not to come to rural areas is because eh...first of all we don't have...our primary health care system don't have facilities to support the role of a doctor. I mean if you have a patient in in a rural setting you can diagnose the patient but what are you going to do about it when you don't have an easy gym in a rural setting, you don't have the right medication in the setting that you are in right now. And for me to come here to just be demoralizing myself because i won't have the necessary x-rays, the necessary equipment to help me...help the patient better. So those	

are the reasons why people maybe choose not to work in a primary healthcare setting. The other issue is when you get into a family, you can't move to a rural area you want the best for your children you want to send them to good schools so if the government had better schools in rural settings then am sure everyone would love to work in a rural setting. So those are the other reasons why people choose not to work in the health care system and the other issue that my colleague just mentioned is just that...i think like you said, the eh..The ministry lacks a proper framework for promotions. They don't have a good or...to say policy that has been set in place that is going to be used to judge whether someone is eh...fit enough to be promoted or not. So there are a couple of reasons that am sure we have...<br><br>Int: okay!!!!!!!! Umm!!!!! The next question or let me just say that i think to increase on that. Are they adequately trained for the job? Differing with the question I asked about numbers trained. Are they adequately trained? Is it adequate!!!!!<br><br>Part8: for nurses and doctors and others....?<br><br>Int: yes!!!! Are they adequately trained, they are like the primary....we are talking about the primary health workers, is the training adequate? Are they adequately trained for the job<br><br>Part 3: yah! Uhh! A kere yaanong (let me just say) this time we are not sure what the curriculum is like at their training. But from my point of view i think the training for for for the nurses, for the health education officers i...i... think they are the best because even the people who are moving from here to other countries they say that side people they grief...they..They go for them because they have been trained like P5 was saying for multiple roles. You end up...you end up... you do multiple roles as a nurse you become a doctor, you become a health education officer you become a nutritionist you become a whatever, so when you go to the other countries, to them you become...you are gold. Mmm<br><br>Int: okay!!!! So they are trained?<br><br>Part 3 and 4: Yah!	

Int: Umm...are the health workers...P5 covered this one when talking about how nurses cover all areas now just to increase on that, are health workers assigned jobs they are adequately trained There are training umm!!!!!! Are the health workers covers that one when it comes to the area, they are adequately...they adequately...are health workers assigned jobs they adequately...they are adequately trained for?<br><br>Part2: Most of the time especially in the nurses who work in the rural areas they are not assigned any job but they find themselves in a situation where they have to perform other eh... duties that they are not trained for so they don't have a choice they just have to make sure gore they function as lab technicians, they function as a doctor, they function as a pharmacy technician, they function as a driver at times, they function as a security guard. All those things because they are...they...it's a situation that they find themselves in.<br><br>Int; Mmm<br><br>Part 2; Yes<br><br>Int; Umm...somebody else...to add on to that....<br><br>Part 3; I don't know whether I will be relevant or...what I have realised is that the nurses who are trained at the the the university level those who are coming with degree...they are doing the degree level...first degree, when they come to the health facilities i think they are lacking the practical part and find that they are not...if you can pair them with those who are coming with a diploma from IHS, the diploma one is better than him or her. They are not...i think they spend most of their time in class than in the practical world...the practical side!<br><br>Int: So how do we relate that to adequate training, what would you say about that in relation to adequate training?<br><br>Part1: i think they have to be...they have to to to do their practical…with their...their practical is not good…it's not enough so they have to do their...more practical before they come to...before they graduate.	

Part 2; Mmm! Their practical part is not good enough for them it is not enough for them. There is sort of like...have theory than the practical part. And you find that on top of that ...as much as you are posted somewhere there, you coach the the that individual, their progression is not the same as for those who have been trained at IHS and that thing in itself it doesn't sound good to those who have in practice and all of a sudden there comes somebody with a degree and you are the teacher to that individual you coach him and they progress, whether because of the degree...the degree because UB diploma...that particular individual will be a degree holder so they progress, after two years is like now you are already on the same level. You are there in service...14 years in service and she is 4 years but you are at par. so that in itself, it doesn't look okay<br><br>Int; Okay<br><br>Part 2;Mmm<br><br>Part2: okay let me acknowledge.Thank you P8 for joining us!<br><br>Part 8; Yes!<br><br>Int; ...If you may shortly introduce.....<br><br>Part 8; Ya! am P8<br><br>Int; Thank you P8. Umm...What would you say about umm...the cadres? Would you say that are they...are they...is there shortage in certain cadres than others? Is there more shortage in certain cadres than others? Ee P3!<br><br>Part3: Eh... i think rona ka we are nurses we will be focusing mo go nursing. (Clears throat) But eh...in the nursing cadre there is shortage of midwives. Ee! There are very few midwives. They are very few eyes nurses, there are very few psychtric nurses and the... ke gore the post e basic....courses...they are offered at IHS and very few people who are admitted to do those courses so there is a shortage......<br><br>Int: O mongwe... (Anyone else?)	

Part5: In addition even pharmacy technicians they are of short, doctors a shortages because you find that in our setup here in Ngamiland you will not find a pharmacy technician outside Maun who is based there because of the shortage that is there. Even doctors are not there. So you find that that critical shortage it is actually hampering on the production or the services delivery that is supposed to be delivered to our customers. So those cadres....shortage<br><br>Int: ee P6!!!!!<br><br>Part 6: even....even in health education assistants, they are shortage because you find the mother clinics with one HEA instead of two or three and everything....se ne go buiwa ka sone (and everything we have been talking about)<br><br>Int: ee P9!!! You were going to add onto that....<br><br>Part 9: No I wanted to...actually P6 has said exactly what I wanted say...<br><br>Int; P6 covered it?<br><br>Part 4: (Jumps in) we are all there is nothing we can say. When we talk about shortage every cadre, we are short staffed of, because if we if we be having enough we wont be going out saying we are going for ARV outside because there will be doctors in all the clinics outside so even in Mau in Maun where there, we find out that there are a lot of patients like in Maun clinic in Sedie clinic. You are going to find out only one doctor and he will be consulting the whole day non stop and it is not healthy to work like that, Ee! It’s not healthy and at the end of the day people get demoralisation because the work is over whelming. Even they are he the nurses, even though even though the midwives yes we are running we cannot say anything about midwives midwives are we are short staffed of midwives. Because gape (also) the other thing is that when people plan, when we plan to may be to build extra health facilities we don’t we don’t we don’t come up with extra nurses to say we are going to need 14 nurses at Matshwane clinic because now that we are having a new clinic. The old the the same nurses who are already short staffed or we are short staffed of are the same nurses that we are going to pull from them and we can take them to to Matshwane. Ke gore we	

don’t we don’t know how we are going to operate. Right now we are having three three clinic that are new that are supposed to open, and we are suppose to pull the the staff from the same staff that we already that are already existing. So what are we how we going to work now, and if we talk about working in a 24 hour clinic we are talking about more than around 18 if you want to the the the facility to work properly, around 18 18 nurses. So that they alternate in shi... in shifts. So the that are that thing thing alone le yone is hampering our progress where in the country because we building building and yet we know that we are already short staffed.<br><br>Int: mmh... okay.......!!!! mmh!!! Is there adequate support to do the job in terms of resources the coordination of the management, is there adequate support for the health workers. We yes, I know you have already touched on that but if you may please expand it a bit like look at the coordination, the management and resources as well. Is there adequate support?<br><br>Part 4: okare jaanong re a o kare re bua re le nosi (Its like am the only one who is talking). Re tshaba go bua jaanong ka o kare ba bangwe….(Its like others are afraid to talk)<br><br>Int: Ee P4<br><br>Part 4: a a a re bueng rothe (Let us all talk)<br><br>Part8: Ee support if I may talk about support from the ministry of health a kere we have different programmes like bo PMTC, TB, ACRHC and others, yes we do have support some support visits from them they come here but the problem is that like we were saying because of the shortage when this when this akere we said that at the national level each poli.. each policy programme is coordinated by a person so when they go down they go they they land in the head of one person. This person is expected to work and so excellently and to perform so excellently in all the programmes that are existing in the health facilities and sometimes you you you you the support its like you are when people come its like they are going to show you how you are not working and you feel so demoralised because you don’t know how to do it because the work is overwhelming to you, you are trying but there is no way how you can do it. Like if I if	

I may give an example of the health posts are our health posts are not staffed we are only having one nurse in the health post and if this one nurse is on leave or is sick is sick or is on mobile, it means the clinic closes. So if we were having atleast two nurses in a health post it means when the other one is not there there is going to be somebody who is gape(also) there will be sharing this programme so that they they they perform to their ultimate best but the the the programs alone. I think there is a lot of a lot of work that is supposed to be done by one person especially at the health facilities. Yes at the national level there might be there there are the they are okay, I think they are okay because one person is is is facing is looking at one programme. Whereas wena o le ko kliniking (When you are at the clinic) you are expected to do all the programmes and to do perfo… to perform. Ha ba ta ba go botsa (when they ask you), when they come they say where are the registers and they find that the registers are not up to date ha ba tsena ha ba ta bo ba bolelela (when they come they will the) management its like they are they are crucifying because you are wena you feel you are trying its only that you can the work is too much for you. Maybe that is one thing that has to be looked into.<br><br>Part6: yah the other thing looking at the ah... resources i will say there isn't much of the support maybe from the ministry level, headquarters there where things which are coming from (and management) and management because if i can set an example of where i am working in Makalamabedi is a clinic with maternity, there is no Motlakase (electricity) just the permanent electricity there in the facility so what we use is the generator and its you the nurse, maybe a client comes at 11 o'clock that you have to operate that generator before you go and see the client so that in itself if you are a health worker there you will feel that somehow somewhere there is no support from the management of the ministry because you you don't feel you you are doing, you don't feel your services are being appreciated, because something can happen in between. You are there operating and maybe it's a mother who is in a labour there delivering something might happen but then it becomes painful for you the service provider at that time you feel that guilty consciousness ya gore you haven't done something right. But yet the if you look at the the bottom line of the whole thing is that resources are not adequate enough for you to perform to your maximum or optimum	

level, so somewhere somehow there is where we are lacking this this support from the management or from the top people there who are policy makers or who are responsible to cater for all those thing that you need for you to perform well. And even like P4 said for the programmes you will find that everything is for you there who is in that facility to make to make it a point that all those programmes run to the expectations of the employer or for you to to perform. So it will be one nurse in that facility like P4 has said who is performing all this and should there be an error or omission of something its like now the the the the negatives are the ones who will now be on the surface, the positive its like they hidden. But then the little you are doing alone there in that facility needs to be appreciated and be supported. Because when you talk of manpower like health post in ideal situation is supposed to be staffed with two nurses so that when the other ne is not there the other one is there. But this is still the current situation that we are in. you look at a facility with maternity there are those staffing patterns that are supposed to be the number for the staffing pattern of that facility. But then you will find out again that those outside facilities they don't work on shifts like here in the hospital or here locally where the clinics closes up at half 4 and then people off. There you work on call, if you are on call you work around the clock. So something can actually trap your mind you can a lot of mistakes you can what and then that in itself it becomes for your customers as well as you as a human being. So support is there partly.<br><br>INT: Ee, anything we could add? Ee P7!<br><br>Part7: adding on that supporting because of the shortage of staff we are having mmhh some problems are failing because there is no resources, as we have said and o ithele le le gore ha e le gore wa re o bata go dira tiro e o go bo go go disturba ka gore o itheele e le gore dididrisiwa ga di yo(You find yourself having to do the work but you are disturbed by the fact that the resources are not there)...ee D!!<br><br>Int: Okay ee P8<br><br>Part8: ee to to mma ke ke akgele gone ha. Di resources tota ha re bua boammaaruri yone, if i may may I compare please...	

Int: Ee P8 you are welcome to do that.

Part 8: Like to tell the truth ee when we were with the local government, primary health care was with the local government yes we were having a lot of problem with the resources, especially the human resources the human resources was a bit more

Int: P8, uhhm I will like to say that we actually have a question coming up of local government and...

Part 8: Ehe

Int: Ee maybe we save that one for that

Part 8: yah I wanted to say that. Thank you. I think for me maybe there is no support since us doctors we are suffering in the hospital (someone coughs). We are suffering a lot we we are specialist, we are general practitioner, we are doing everything. If you are having a case you have to to you need to send to a specialist you have to try then yourself. We are doing calls in that big hospital alone. You start at 7:30 you knock off at 7:30 you don't even knockoff you still continue doing other things alone. And when you are having emergency you are doing emergency alone. When you start to call a specialist who is outside like Nyangabwe or Marina you will spend almost 2 hours to get just to..for him, for him to just assess that patient. Sometimes they even refuse the patient, sometimes you even argue with the specialist. There is no support, right now there is surgeon in the hospital we are suffering. Most of those patients who are coming they are having such and such case you have to to look out at the patient. When you try to explain to the patient the the reality you end up having trouble. The patients not even believe, will not even accept to what the effort you are doing for her or for him. I don't know for me I think in this point medical officer or everyone there is no support.

Int: Okay...

Part 7: And there also in the health post once the nurse who was at eh... where I am working now they send her out to the health post. I was just asking her, whats happening there how many nurses are working there. She just say I'm alone. How

alone, young nurses, who just from school or 2 years from school you are sending that particular person to the health post, who should stay alone. Dispensing medication, taking…vital signs, consulting the patients, and she is the one who is referring also and accompanying the patients to the hospital. I don't know may that way is working here but other places they do not work like this.<br><br>Int: Okay! Anybody to add to this<br><br>Int 2: What about the issue of transport now as a resource?<br><br>Part 7: ke yone e key neng ke re ke a e bua go bo gotwe re ta ta re compera re tsena kwaa. (That's the one I wanted to talk about but it was said that we will later compare when we get there)<br><br>Int: Okay! Uhhmm maybe uhhmm any anything to add on to that umh...the gaps in in health health.. gatweng (what) health care workers, maybe the conditions of services. I know you just talked about that.<br><br>Participants! Coughing and sneezing!!!!<br><br>Int: The conditions of the services what would you say about them. Maybe in a station if you were to describe the condition of services what what would you say<br><br>Part 5: the conditions of services<br><br>Int: for for health care workers in primary health conditions of services<br><br>Part 3: They are usually going down, I have to close it. They are not appealing at all they are not good. Let me say all health workers, like you have set an example gone gore kana (that) the conditions of services they we are talking of things like promotions, the progression whether its going well or not, we are talking of staffing, how facilities are staffed, we are talking of safety, we are talking a lot of things so the the point that we have said here tota they actually define or include gore conditions of service are not okay.<br><br>Int: okay! Thank you!	

Part 3: they are gradually degrading they are going down going down down there they are not picking up in fact.<br><br>Int: Okay! Anything else or we could move on. okay the next question is about health care workers in rural areas, rural areas that we are highlighting is there a problem i know I was just asking about, first I was just asking about health care in general im.. we are going we are going to look at rural areas. Is there a problem of health care workers in rural areas if yes why do you think it is so. Touch on the living conditions in rural areas, how they contribute; touch on the remoteness how it contributes. Anybody who wants to talk about that? Is there a problem? First of all lets address the question, is that is there a problem in rural areas if it is there, why do you think is there?<br><br>Part 7: There are plenty of problems in where I am umh... like umh... there is no there is no adequate accommodation, you find that eh...the few houses that are there they have to be shared. You share despite the fact of whether the other one is married or not and uhh this housing units will just be there, so many many people will have to look for accommodation in the village.<br><br>Int : ee P4<br><br>Part 4: especially ……… in Gumare. And eehh some villages don't even have rental houses so actually have to struggle to find a place where you can stay. So its its it's a big big problem. The other problem is the the distance from the main village. You find out that uhh the transport will not be enough to make it easy for you to reach the the next place with better facilities. So its always a struggle.<br><br>Int: okay! Ee, ee P5 you were going to add mo go ene<br><br>Part 5: ee D! What I real want to say, in addition to what has been said, i think another problem is that of staffing eehh rural areas you find that there is only one nurse in that facility of which during her/his absences there is a problem and there is nobody to provide the service. Let me say she has gone for mobile, she is on leave then there it becomes a problem. In addition to what has just been said transport, I mean	

accommodation is another problem. We share whether you are married or not, you share with someone, you are a married lady the other one is not married, you share the house.so…<br><br>Int: do you think the the remoteness contributes to that being a problem<br><br>Part 1: It does. It does like we have aaaa alluded before the lack of defined policies. You that somebody is somewhere out there the remotest area, she will be there for four five years in that area where there is no transport, where she hardly reaches his or her family, where he hardly.., where she can hardly take children like my colleague has just said to the school of his/her likings. So you know by the time you are out of that place its like you don’t have life.So the remoteness also is a problem where you are.<br><br>Part 3: akere if we are talking about remoteness we are in talking about areas where there is no electricity, so you don’t even, you cannot. I don’t even know if this are there some combined fridges for you to buy so that you can use gas that side. So it is very difficult for you to live there even the shops, there is no shop there. For you to reach a shop, for you you are surrounding are are the semausu(tuckshops). And those semausus(tuckshops) you go there you try to buy mealie mealie, kana I worked ilived, I worked in the rural area. You find that the mealie mealie e santse e senyegile go na le diboko mo teng (expired). So these are the things, you want to go on offs, or you are on leave you want to reach town by two days. You are going to stay three days before you get there, so these are the things that are demoralising the people to go there, because you go there its very especially for our children who are coming after us. Rona (us) maybe we could bear that, but because this one’s bone ba goletse mo motakaseng (grew up with electricity around them) I don’t even feel gore go ta nna le mongwe yo o taa yang(there will be anyone who will go) that….. if the government is going to find a way of improving those areas I don’t see a re.. I don’t see he, I don’t see people going there in the near future.<br><br>Int: okay!<br><br>Part 1: the other problem is that like ehh P1 had said that when you are there you should know that ahh you are forgotten and there is no appreciation that atleast you	

have agreed to go to that remotest area in terms of an incentive or allowance of some kind. You are just like somebody in maun or somebody in Gaborone getting the same pay, so I think ehh if the government could appreciate people that are working in remote areas. Re bo re bona le rona (we would also recognize) that the government is saying thank you for working in areas that people don't want to come to.<br><br>Int: ee P4!<br><br>Part 4: Point of correction, I think gone mme go na le there is an incentive, go na le remote area allowance but its very very small. You can imagine if am in Gaborone and am transferred to Bodikwa to be given 400, that is nothing to me. I will rather stay in Gaborone than going to get 400 because at the end of the day that 400 cannot do anything to my life because for you to come back from Bodiba to go back to Gaborone you use transport, you are going to pay more that and its in a in a single journey.<br><br>Int: Ee anyone else who want to add to that. Okay not like what about the cost of living in rural areas? Does it contribute to the problems, cost of living, what would you say about the cost of living in rural areas? Anybody to break the ice!<br><br>Part 5: it is very expensive, it is very expensive if i may give an example, right now there is choppies here, there is choppies in in Ga... in maybe in one of the towns that side, and you go to choppies in shakawe you compare their prices, those prices are very high. And those that sho… that choppies it is the one that is being used by people from Bodikwa , Betshaabonitsoga bo Seronga bo Kankuu and all the other areas. So for to come to Shakawe to come and buy some groceries is is expensive for you to move from that side to shakawe and you find that even what you are going to buy is still expensive. So the cost of living in in in a rural area is very expensive, gape (also) you cannot eat what you want because ga… some of the the the commodities are not available where you want to, where I live in, where you are staying, tsone di fruits tse go tweng re di je, where you are encouraging people to eat fruits to eat what, where are they going to get it when you are in Bodikwa, you see?<br><br>Int: ee P8! Anything else about the cost of living? What about the opportunities for continuing professional development and furthering your education?	

Part 8: (numerous) zero, zero zero ahh zero<br><br>Int: What would you say about that?<br><br>Part 8: zero zero<br><br>Int: Okay anybody to clearly come out and say. Opportunities for professional development, maybe furthering your education<br><br>Part 10: it is zero because like we have mentioned of lack of resources in those areas there is no electricity, you cannot pursue a distance course we are if you want to upgrade yourself, amh. Sometimes like it has been said you are forgotten, people don't even know gore you do exist, you are out there. Some of this development courses that can actually bring you up, you are forgotten and then you lose a lot of things out there. By the time you come nearer to town its like you are a new person in this profession, in the profession.<br><br>Int: mhh...what about aahh..<br><br>Part 10: can i….<br><br>Int: ee P10!<br><br>Part 2: in fact with the primary health care, when we were still with the ministry of the local government for a nur for for a nurse. Im talking about a nurse because im a nurse, fro a nurse to be for a degree course, was a taboo, while our counterparts in the ministry of health have long started progressing rona we were just starting when we were being relocated we were just starting to be, to be developed. And rona (us) you can imagine somebody of my age e le gone a isiwang ko degree after 25years of service. What do you expect me to do there, wa bona, so ga ha go na ha go gape le gone even the the nomination of people to go there you don't know which criteria are used because you find that people who have been there are left and there are people who are coming are coming are coming who came la after you are the ones who are nominated to go for further studies.<br><br>Int: okay umh somebody earlier on talked about family and children, so what I would	

like to know is what about availability of jobs, schools for partners' maybe. Availability of jobs for partners or schools for children if you were to …….<br><br>Part 5: In remote areas<br><br>Int: yes<br><br>Part 5: we have already talked about it.<br><br>Int: so partners how they would fit in. to eeh like in rural areas.<br><br>Part 5: they don't fit in.<br><br>Part 4: kana when you are transferred to a to a rural areas you are being parted from you're your family and the chances of divorcing they are are increasing. So most of the some, if I may give example, most of the nurses who were at who were were who started working at my age… with me they lost their a their marriages because of the rural areas. They got divorced you there to you go there you are on off you go back home you find that husband is is always… the mood is no longer the one you know. So you go back you come again now when you come into the house he he goes out of the house. So these things have affected a lot of our social li… our social lifes.<br><br>Part 4: Mmm even the growth of your children you don't stay with them there, sometimes the guidance you can give at the right time. Its not there by the time you reach them now you are just talking, ke raya gore its like ha go na molao (there is no order). They even know better than you, so its very difficult. Even guiding them on their school work what, by the time you you are now with your family you are now looking for this private schools out there because the you're your daughter or son has not performed well, you are now parting you're your salary again to look for improvement in her education. A lot of divorce is high ministry has to advocate for this rural areas there not even divorce on itself even connecting well, you can be together but go sena that connection because the other one is staying on the other side of the river the other one is this side.<br><br>Int: anything else on that. The next question, what do you think should be done about	

the lack of primary health care workers in Botswana? we just been talking about the shortage that the shortage is there. Now what do you think can be done?

Part 5: like it has been said looking at the nursing profession, shortage of midwives nna i will suggest gore if eehh there is this three years training of general nursing why cant there be a package ya gore you do the general nursing when you finish you you continue with midwifery at once. So that when you come to practice you are fully fledged with midwifery part you are a generally nurses as well as a midwife because the other things key gone gone gore some of we don’t like midwifery, some we term it it’s a it’s a dirty job so its not all of us who actually ehh do midwifery but then it is if it is made compulsory when you finish your registered nursing you do midwifery then maybe that can cap the shortage of the midwives.

Int: okay! Yah! Shortage, somebody else!

Part7: Yah I think one other thing that the government could do is to reintroduce the compulsory community services for health care students that complete their studies. If could atleast be a year or two you know it will be certain that in that period of time the primary health care it has staffing. For those that graduate out of school there will go straight into the primary health care they will work they will give back to community. That way you will know that there are people that are learning the health post, they are learning the health clinics in the rural settings and they are also learning about those rural settings because i never been to rural setting or health post when I graduate it will be less likely for me choose to go there. But if I am as a requirement forced to go there for me to get my medical practitioner’s license i may decide that you know what when I finish or when I complete this community service I may want to choose to stay here for a year or two before I commit with my life that way you can convince people to know about this rural settings to understand more about the primary health care. Before they commit with life, before they get married and ha eh... forced to cater in their children their spouses and all of that. Because im still young I can afford to stay in a rural health post without a spouse for a year or two while im still exploring myself. But for someone who is already committed into marriage, for someone who has kids it will be unfair for them to go to a health post or rural settings with eehh will not have

any access for the their children will not have ehh good education so eehh I think community services its its for me could work for the government.<br><br>Int: okay ee P4!<br><br>Part 4: eh like…to add to what P5 was saying about mmh midwifery, P5 mentioned something like people should be nurses should be midwifery should be a compulsory course. I think I think gape the other the the the other thing is that the government should strike a balance between all this other post basic courses. Because you willfind that like P5 said this coming nurses who are co like the young nurses they are not not very keen to go for midwifery and yet the government will decide if I choose to go for community health nursing I don't have midwifery the will send me and yet they didn't even strike a balance to find out how many community health do we have, how many do we really need, should we continue ka training the community health nurses while we don't have enough midwives so those things the government should strive to balance on that. And the other thing is that like what I have previously alluded that the the government should start giving incentives to some of this courses, courses like midwifery. Midwifery is a course that is, is a high risk job so if the government can start some incentives on this ones I think this also can make people to. Some of the people will even though they didn't want to go there they will end not going for a community health nursing course and go to midwifery<br><br>Int: okay! Ee rra<br><br>Part 1: yah, I just want to add onto that, I want to say that they also need to look at it first right form the beginning that is right from eh... primary the secondary school level. In that aspect they need to make science subjects more attractive for the the pupil. Most of them go through school believing that science subjects are very difficult. Probably because of the way they are been taught or the way they are being introduced to science subjects. So they end up believing that ohh its too difficult its so, eventually you find that a very few of them go into sciences and you know I think that could also contribute. If there are ways of making the the children, the students more interested in science ……. you find most of them going into health related professions.	

Int: yah! We we have touched on training and I would like for us to also look at the distribution of primary health care workers, look at the retention strategies and look at the the skill mix,and task shifting but not really limiting to those, those are some of the things we could look at. Okay anybody…<br><br>Part 6: I think maybe you need to break it down..<br><br>Int: like we have been talking about training, when we look at training lets look at the the quantity and the quality akere(right?). We are trying to come up with what could what should be done about the lack of health care workers or primary health care. Okay you have just touched on the training and I am also saying, lets also look at the distribution. What do you think could be done about the distribution or deployment of primary health care workers?<br><br>Part 8: Yah I think quite a number of this have been touched by those who spoke earlier. Quite a lot of the issues mentioned, eh.. are very important where you talk of distribution because at present situation because of all those things they have said there is a gross disparity between the walk strange and the big cities and the rural areas. If those issues they talked about are addressed then more people will be interested or would not feel resentful about being posted to rural areas. So I I think those issues they mentioned are cardinal in showing equity in distribution.<br><br>Int: yes!And the retention strategies that could be adopted. What strategies could be adopted to retain primary health care workers? What retention strategies in in rural areas?<br><br>Part 5: give them more money<br><br>Int: yes<br><br>Part 2: yah more money just like he said, additional, apart from more money promotions that could be another alternative. Free accommodation, Accommodation, Transport, even transport.<br><br>Int: even Transport...	

Part 1: Then there could be ways that you could arrange for their welfare like for instance they mentioned about things being expensive in the in the rural areas. Supposing there is a way in which they could be assisted with getting their groceries, from cheaper sources and taken to them. Instead of them having to leave their posts on their own and then searching for the very expensive food items.<br><br>Int: okay! And what about the the….<br><br>Part 5: And also resources, resources must be there. Have a house with electricity, clinic with electricity. Schools for the children. Work for the spouses.<br><br>Int: I was saying what about the right skill mix in in clinics or in rural areas? And task shifting, what do you think about that?<br><br>Part 3: coughing!!!!!!!! Yah!! That one also can be looked into. The right skill mix like you find that this is a health post e e leng gore (that) it has a non somebody who is not a midwife or non-skilled, so itsa problem because that the community itself you would expect gore this would be a community without women who can become pregnant, they will need those services in that particular health post. So then like we were talking of distributions, if there is a standard pattern of to say a health post must have a non midwife and a midwife. Clinics with maternity must have so many midwives and so many non midwives. Then that way maybe it can work because you will find that the mix that we have out there, it is not actually the mix that the community or we expect it to be there. Because some are unskilled on certain issues like midwifery. Most of the health post here they are non-midwives so it is the clinics with maternity which actually visits maybe weekly or twice in a week to that facility. But guess what something can happen in your absence there. Maybe this nurse is confronted with a mother giving birth or delivery. She doesn't have the skill of how to conduct this delivery and may the repercussions may be somehow negative so the mix is not right. I think when that can…<br><br>Int: Yah, i also mentioned a task shifting. Ohh sorry<br><br>Part 4: Not, ke raya gore (I mean) we should not talk about skill mix mixing of nurses	

alone, even like we were saying you find that there are we we are short staffed as far as the lab technician, the ………, the le bo (and) pharmacies technicians. So we need those people at the health facilities because if they are there kana if they are not there it's the nurse who sees to it that the drugs are in stock, she is the one who checks the the drugs stock the stock and she is the one who saying I need this amount of paracetamol, I need this amount of amoxicillin. And this is not the work of the nurse it's the work of the… a pharmacy officer. So if we are having, if we are having pharmacy officer at the health facility atleast the the each mother clinic should be having the a a a pharmacy technician and a doctor. We need doctors in the clinic so that we relieve this nurse. Nurses are overwhelmed by the wo... the work that is not that does not belong to them.

Int: anything else we could add tothat? Now in your experience what solutions or interventions have already been tried to resolve..

Part 2: You skipped the task shifting

INT: Ohh sorry, ohh I thought P4 covered it

Part 2: Okay

Int: But we can go back. Elaborate on that, the task shifting what do we think about task shifting? Shifting of tasks in primary health care.

Part 4: okay! Its partly what P3 has said

Int: Yes P3 touched on that but you can also add on that.

Part 5: yah like P3 has already mentioned it is very important to shift the tasks because them on itself we actually avoid things like burn out. Will actually avoid things ike being overwhelmed by a lot of things and then at the end of the day you are somehow stressed by a lot of issues. Like for an example, is because of this shortage, clinic outside there, I think shifting will be much applicable if you work on shifts but the problem is staffing. Because if you work on shifts you will know gone gore (that) you are going to work for a certain period of hours somebody takes over. And then instead

of like like the doctors has said (24 hours) and I have said that you work around the clock. That actually creates burn out and it even lowers your performance and production also goes down. So like in addition to what P3 has said if we..,there is task shifting in the facilities with the expected cadres all of them to be there I think work will be much easier thou not easy to say but will be able to work like normal people.<br><br>Int: ee P6!<br><br>Part 6: this is because as a primary health care officer you are only concerned about the client that comes to the health facility you also have to go out into the community to see how they are working how they are living because some of the problems that they bring at the health facilities is because of the factors that are surrounding their environment so the causes of their illness some of them are because of how they are living in the village so you have to move into the village to see how they are working you have to make follow-up of your clients who are defaulting we have to go out to see our….clients we have to go out for mobiles those who are just far away more than 5 kilometers from your health facility, you reach them by going there taking the services to them so if you are not overwhelmed this things should be done smoothly.<br><br>Int: On the issue of task shifting I think the reason why I'm asking this question is because there are people that indicated that there are certain tasks which they felt ahhh… could be done by somebody else eh… they were saying there are certain tasks that they feel for example if you are a nurse you would be doing something that could be done by somebody usually of lower qualifications. I don't know what your take is.which tasks…<br><br>Part 6:yes its true, if you may remember very well in 2008 the nurses in the clinics in the local government ba ne bana le (they had a) slow down gatwe ke eng? (What is it called?)(go slow)go slow because we were saying its high time the government pulls out some of the activities that can be done by other people because some of them are not part of the nursing the nursing duties things like collection of blood from a patient its not a nurse's duty its for…gatweng kana la mpalela lehoko la teng (I cant pronounce the word) its not part of the nurse's duty le yone (and also) the prescription	

of drugs even the dispensing of drugs its not part of nursing duties is the pharmacy officer and we were saying some of these jobs a goromente a bone gore a di ntsha mo go(the government should relieve nurses off these duties) nurse they should be worked by the person who is responsible for that duty so ke sone seo neng o bona rena le tsone ditiro tsene tseo tse eleng gore (that is why we have such duties that) they are worked by the nurses and yet…

Int : is it is it a general consensus like this room or I don't know what..

PT2: Yah,its quite relevant in all fairness its really relevant because what happens is that you end up doing duties that are not your primary duty and it impacts on your own primary duty, there are things which you could have concentrated on and done better but because you are dispersed doing this doing that doing that you end up being inefficient in what you are primarily trained to do so in the long run I think its quite fair to allow you know ahh task shifting and people should do things that really its their specific job description

Int: Okay,is there anything else on that question. Okay! moving on.In your experience what solutions or interventions have already been tried to resolve these problems the problems we have been talking about. for example we know that the government tried performance based reward system, you know what do you think about that and maybe the moving of primary care from ministry of local government to the ministry of health

Part 2: can you come again with your question.

Int: in your experience what solutions or interventions have already been tried to resolve these problems and then I gave an example of PBRS and removing of primary health care from ministry of local government to ministry of health like all these interventions which have been tried which other interventions have been tried and have they worked? Ee P2!

Part 2: ee! the intervention of PBRS,PBRS was a very good pro what should I say? eeh system system it's a very good system but I think its not going well alright because we are sort of not using it the right way. Yah, because at the end of the day everybody gets

100% and everybody is performing even though the work is not there that has been done so I don't know how we can do it because people I think gape some people we are lacking some honesty when it comes to PBRS even when you know that at this person is not working we are not able to call these person to say here you are not working you are giving this person a 100% o tla utlwa batho ba ntse ba re (you will hear people…) people will be saying ntsha semang mang le ene o promotiwa a ntse a sa performe le rona re fitisa hela so this thing ke raya gore its not doing the right its not doing its right purpose its purpose its not been done ke raya gore ga ke itse I don't know but we can still but I think there is something gona le gole gonye nnyane mo go teng atlist e dirile sengwenyana mme we still have a long way to go with PBRS I think change our attitude towards it because even people when we are supposed to go and I mean to start assessing people it takes you hammer on people to say lets come and seat down and assess each other, people don't want to be assessed so its very its very gape ga o le (also if you are) a supervisor at the end of the day it demoralizes because you have to be running around with people who are refusing and writing letters to people who are refusing to sign PBRS with you maybe if there was something like we heard that there are people who have been expelled because they were refusing to be assessed and refusing to sign maybe that could be an example because gone mme gone at the end of the day PBRS eo was a very good system put in place by the government(so what)ee D(no its ok) ke feditse(ehe o setse o heditse)<br><br>Int : I was going to say what do others think of PBRS, ee P2<br><br>Part 2: yah I think PBRS is a tool for improving productivity not really for addressing those issues that we talked on early and you know when you look at this tool first of all its not very user friendly a lot of people believe me still don't even understand how to fill those forms and people still have a feeling okay I was given 97% last year and I have not seen what difference it has made to me so that in itself makes a person feel a bit discouraged about filling it again the next year because the forms are been filled but really no difference has happened the reward has not showed up so those are areas where people could get frustrated but nevertheless I still think it's a tool directed at improving productivity the tool in itself is good yah but it still does not address the	

those salient things that have been raised in this forum and what you mentioned about again the other side which is aah putting the ministry of health, primary health care under ministry of health, it's a very good move but nevertheless I think that one is a long term thing what I think is been done that's towards addressing the problem, is what you are doing now I believe the first step is what you are doing, finding out from concerned people all stakeholders what they feel how they think the way forward can be constructed and I think to me that is the most the most important step that has been taken so far.<br><br>Int: thank you,ee P9 le nele tsholeditse,ok<br><br>Part 9: no,he partly said what I wanted to say concerning, yes<br><br>Int: ee P1<br><br>Part 1: on the relocation of primary health to ministry of health mmh… there was no consultation in the first place even people who were relocated were never called were never consulted to say we have decided or we are thinking of maybe moving you to this ministry how do you feel about it so people we were sort of in a reality shock here there was a lot of shock between us and the ministry we were re locating to because a lot of things were not looked into even the resources were moving to were not looked into like human resources you will find that in Ngami we were the clinics were moved to Ngami DHMTwe formed a DHMT gakere?and you find that there are only two or three people for admin officers who were employed for the hospital and these people were never increased the numbers were not increased and yet there were 26 health facilities moving from ministry of health into the Ngami DHMT plus the hospital so there was never a time when people ke raya gore people did not look into these resources to say we are going to need these resources to move the primary health care to ministry of health, the other thing I feel is that this talk was gape (also) was brought about by the fact that it was done abruptly it was not done great… maybe if the government could have done a pilot test of maybe taking two districts a time to say we are taking CDC and we merge it the other districts remain with ministry of health after a period of time we take a few districts again we put them into ministry of health until	

all the districts finished but it was so abrupt and people were not prepared those from the ministry ke gore there were lots of two attitude clashing together because rona (us) even now we are still filling that attitude even when people are addressing us they will say this people from khansele oa bona (council yoe see?) ke gore we are at the end we were just like in no man's land because people at because we were sort of moving to the hospital they did not want us and it was very difficult for them to accept us so it was traumatizing to us even now it has traumatized us, it has demoralized us in a lot of aspects gone jaana right now akere we are bringing our own resources that we were using from local government we moving with our vehicles our houses our what right now the vehicle that we where moving from…I think like I was saying there were a lot attitudes maybe ele rona (even us) we had some attitude towards them le bone (and aslo) they had an attitude towards us in such a way that people will be addressing us like I was saying labeling us, the vehicle that we were bringing from local government rona when we were doing local government when it come to transport we hada lot of vehicles, a lot of vehicles and vehicles that are road worthy and they were brought into a vehicle that was taken care of at the local government and were brought into the hospital and because all the programmes bo PMTCT,CHBC, ASRI and other programs TB all of them they did have their own vehicles so that these programs could operate without any disturbance le bo ARV tota,2-3 vehicles for a program so we came into the ministry of health with these vehicles and when we got there we were told rona we don't have a vehicle that is called PMTCT vehicle a vehicle is a vehicle, right now yesterday I was told that our PMTCT vehicle it was given to a one of the departments not the hospital the department so the vehicles got damaged and they were not repaired all the vehicles are down we don't have vehicles, the program officers that we supposed… kana when we talk about a program we are talking about the people who are into the community, you go into the community and to see how people are doing to take care of home based care patients even the home based coordinator cannot go in the village right into the Maun she cant go into the village there is no vehicle to go to use at the end of the day sometimes we even use our own vehicles to go to the patients because you really want to do your job sometimes you fail you seat down because you get demoralized because you cant work sometimes even the ARV program a very	

sensitive if patients we default to go to the patients, today the clinic to karinga,we were supposed to go to Kareng a doctor was supposed to go to karinga the trip fails because the program vehicle is not there so we are facing a lot of problems a lot of problems, I don't know about other districts but our district we are facing a lot of problems.<br><br>Int: ee P1 thank you, anyone else what on the move, what do you think about the move.<br><br>Part 4: the move was good was really good but as P1 said there was aah I think people were not aah oriented well because it was like in our district it concerned a few people they were the ones who were always having meetings and the rest of us we did not understand what was happening and at the end there was this merging and then the merging le yone gape up to now we are failing to be one thing, we having to be one thing there is segregation we work together yet we don't know what the other people are doing its jus chaotic Mh… so we are hoping gore you know eventually all of us will see gore we need each other we need to work together in order to make this merging a success.<br><br>Int: ehe…just to clarify that we are talking about the merging of local government and ministry of health, yes<br><br>Part 9: and its like this merging has brought, the work now is too much and the staffing levels have not been been increased so a lot aah some jobs are not been done satisfactory because now you find that in the hospital there were a few administration officers who were you know just doing hospital job and now there is an addition like P1 mentioned of 26 health facilities that have joined the hospital now these administration officers cannot cope up with the work load so a lot of things are always behind a lot of things are always behind and expectation is that people should be doing their job satisfactory but because of short staff its not possible and this issue ya lack of transport or inadequate transport and breaking down of vehicles, it is just causing a lot problems, programs are not being done patients are not been referred on time to other referral centers, its just chaotic.<br><br>Int: ee P9, anyone else on the move from local government	

Part 6: yah, the other thing ke gone gore ever since this move was there of course like they have mentioned it had some ups and downs in the way the operations as far as the operations are concerned and it has actually impacted negatively on the standards that we have as a district because when we talk of transport if I may pick it as an issue we have got standards which we are to operate within, looking at our clients, clients waiting time. the facilities which are outside there like we have mentioned they don't have doctors and the outreaches for ARV clinics are done by doctors locally here and then the personnel will reach the facility around 11 around 12 when you are about to knock off for lunch because of transport problem and you look at that poor client who has been there since 6 am coming for the service s/he has to wait up till maybe 12o'clock the first client getting into the consultation room just because the core problem there is the transport issue which emenated from the merging so it has actually resulted in a lot of things which are not working well, a lot of negative things like they have said also the cohesion that is there in between is some how questionable.<br><br>Int: ee P2, the other intervention,,, P1 touched on was eeh DMT the setting of district management teams, what about it it was an intervention I believe as well. Help us with this one.<br><br>Part 2: yah i think for me its like giving a new name to an old structure just changing the name does not in anyway in itself bring about any change i don't know what others feel about it but for me I dont think they just gave a new name I cant really see any impact.<br><br>Part 2: gape (also) if you compact with the DHT district health team that we had with ministry of local government that was a real team than what we have now, here we don't have a team its called a team but its not a team gape (also) what we have had even thou we did not have anything written though, the DHMT was supposed to be an independent institution from a health facility we find that rona we are housed in a health facility and we have been swallowed by that health facility, we have been swallowed by letsholathebe even in a meeting they will always be saying here in letsholathebe we are planning to do this this because they are in their house they are	

not in DHMT Ngami DHMT so maybe if we can move out of letsholathebe things will be better but the fact that we are in letsholathebe we are no longer DHMT we are a letsholathebe crew.<br><br>Int: ok, anything else about the DMHT, ok another thing<br><br>Part 5: The structure was not well defined I think they should re-define the structure well and fit it gone gore who fits where because you will find that for example yes you are DHMT but you are on the same level let me say that, like maybe in a facility you are all on the same scale i think you must convey meetings as a team not like I will be there alone and the other person would be there but we are on the same roof you have the same objectives and goals and the you know for the operations that are within the DHMT but then if there is pulling in and out you are not together for example you are in a facility if you are here you have divided ideas divided opinions we cannot say that we are a team yah it is just a word like we have been saying its not clear.<br><br>Int: ee P9!<br><br>P9: I think P5 mentioned a very good thing when talking about a proposed structure there was a proposed structure that is in place which we are not adhering to you find that in the preventive part akere preventive services there is a D1 post somebody who is in D1 is the one who is heading the preventive services and from you can imaging from D1 you fall into the ground to a C1 ke gore there is nothing in between and its very unusual for you to be in C1 and you are supervised by a D1 there should be something in between C1 and D1 ee (yes), and when you go to nursing services the nursing services the nursing services we have akere we start from this side there are 2 branches, there is the hospital there is the primary health care gotswang haa (from there) when you are in the primary health care the hospital has got 2 D3 while the primary health has got 1 D3 so there is a lot of imbalances in the structure even if you go to other districts I don’t know why I don’t maybe they are still to come up with a new structure because if you go to okavango things are very different there, you find that primary health care is led by somebody who is on D4. You see, and she I mean when primary health care nursing cadre is somebody on D4 and the D4 has gone on	

transfer, now this side there is a D4 umm in the hospital there is a D4, there is a D3, there is a D2 with highest person. There is no D1 and here the structure is saying the highest person in the in the in the nursing cadre should be a D1 person. So there are a lot of imbalances, we don't know what wha what is what?

Int : Okay! Another intervention which was mentioned by the the policy makers team was the , they talked about scarce skill as part of the intervention. What do you think of that intervention?

Part 2: scarce skill allowance?

Int: Yes, scarce skill allowance

Part 2: there I think there is a to to to mention it akare we have been lobbying with the government to give to us also as nurses because other cadre are getting it

Part1: Kana the scarce skill I there was that eh… research, what is it, when doctors started getting it pharmacists started getting it some cadres were left out with the term scarce. When you talk of scarcity what are we real looking for? We are looking for that particular cadre gore does it have enough manpower to provide the service, and then the issue was that they were those cadres or those professions which were applicable for scarce skills. And then there came an issue ya we nurses okay fine, according to the definition it appears we are not scarce but those people who is doing their jobs? The work is being done by who by a nurse so then why cant a nurse be given an allowance on top of what she is getting because she is taking other roles of other people into her or his profession. So it is it applies for certain professions for others it does not apply but those professions where it does not apply it is also applicable because when you talk of scarcity it means this people scarce so their job is not done well because they are not enough in the market. And then who is doing the job is this other person yo e leng gore ene (whom) she does not have any allowance of doing other people's duties. This is why nurses said okay fine let us withdraw from this other duties tse e leng gore (that are not…) they are not ours. Because the owners are there but they are not doing it, then why cant there be an appreciation of those who are doing it? So nurses need scarce skill though they are not given it because they are actually the umbrella of each

and every service that is being provided there. Or even if even if it cannot be termed scarce, let there be an allo allowance catering for this other duties that they are doing.<br><br>Int: Ee P1. Okay! umm, the other thing we should do it is also the introduction of the medical school, medical school programmes and faculty in rural areas which and primary care.What do you think about that the introduction of the medical primary school? I know that we have some students where in Maun who are from the medical school, others are in mahalapye, others are in Gaborone. So what do you think about the introduction of the medical school?<br><br>Part 8: That was very very very good and it was long over due.<br><br>Part 7 : It is good<br><br>Part 2: Mmm…this is very very very good because in some few years to come we will be having many many doctors. It was a real good idea. And it is not enough we need other other one in the northern…<br><br>Int: Yes P1 you were saying?<br><br>Part 1: Kare (am saying) it is not enough one it is not enough because we eh... to train vigorously, because we have been we have been lacking behind maybe if we can get another one in five years to come, maybe in the northern part, so say so that gore re re re nete ka bonako (so we can quickly…), re (we should) produce so that we produce a lot of them quickly (someone coughs)<br><br>Int: mmm yah<br><br>Part 1: Like you are saying they are there we appreciate but then we are looking at gone gore when they graduate or they finish we are having a problem with the doctors being posted to the rural area, the retention part of it. Let us look at the incentives that will work for them so that they are out there and like he has mentioned let them have a taste of the rural areas before they graduate to work for work. Yah, because then there will be others maybe who will like those areas out there for the first one or two years they will like to work there. But then if they haven't they did they didn't have a taste	

before and then now is somebody who is to start work he has been in Maun, he has tasted the life in Maun it is going to be difficult for them to go to move out there. So maybe during the training the practical part will be at us seeing them one in Phuduhudu one in Somelo so that they may have a taste of how those ares appear, how they are.<br><br>Int: Okay! And maybe lets say you will say we have, you will say something about this, we are just talking about the introduction of the medical school. Like as a medical student what do you think about that, the introduction of the medical school programmes and faculty in rural areas and primary care? What do you think about that as an intervention?<br><br>Part 3: Like as part of training, you have to do certain or faculties….<br><br>Int: we are talking about interventions that will help alleviate the problem of shortage of primary health care workers.<br><br>Part 3: Okay!<br><br>Int: So I was just asking about the medical school like what do you think about the medical school programme of some faculties in rural areas and primary care ? Akere we have, like we have you guys you are here, and we have other students in mahalapye and others are in Gaborone. Like what do you think about the intervention of the medical school?<br><br>Part 3: Yah we do come here for 10 weeks for like in Maun, and Maun I don't know I mean Maun its okay its more modernised type of ah… it's a town, you can live here for 10 weeks, it has water, it has electricity, it internet but I would work in maun. But eh… to go into a remote area in a remote setting its less likely, it's a challenge and I would'nt do that because im used to my internet, im used to my own …… that's the thing. Its difficult to live without this this things, and I think the the medical school yes it it it it did a good job in trying to, it is still doing in a good in trying to revolutionise health care system in our country because now we can have researches like the one you are conducting now. So ah... to me the medical school is bringing something good to,	

as an intervention to to the health care, primary health care. More so that we will also get taught public health in incorporated into our curriculum. Therefore, we get a taste and feel of what we could expect in this kind of settings and yah and the attachments of us in this uhh… rural areas it is good although they are they may not completely be rural. Eeh... it still gives us a chance work in a hospital that has limited resources and see how we can maneuver around that so I think it could serve as an intervention to the shortage of staff but we will see as time goes on.<br><br>Int: Okay! Ee P2! we just been talking about intervention ohh<br><br>Part 2: I was… am thinking aloud<br><br>INT: its okay<br><br>Part 2: I have seen in other African countries where by they they train this people who are called clinicians, I think their courses are lower than the doctors, they and they don't they don't they don't take very long time in their courses like the doctors. So maybe that could be another another way of improving our heal where by the doc the maybe the the... our government starts some training of the clinicians so that they can maybe go to this rural areas while the doctors because most of the doctors they will be in bigger facili... health facilities.<br><br>Int: Umm okay! Now looking at all this other interventions we have just mentioned, like the solutions. Akere the government was trying to provide solutions to the problem. Would you say they worked, everything we have been looking at DHMTs, medical school, moving from the local government, would you say these interventions worked?<br><br>Part 4: I think those interventions have just been done, they have not been there for a longer time.<br><br>Int: Okay<br><br>Part 5: I think right now it can be very difficult to say worked or not. Ee I think we need to give them sometime, to see to assess whether they worked or not.	

Int: Ee P1<br><br>Part 1: But nna I have realised that that short time it has real showed me that if we were really merging together as a teaching team. I think I I I the aspect of human resources was becoming better, especially for nurses because when we were with the local government most of the nurses were moved to the hospitals, hospitals and local government was given a very low share and right now I think the situation is improving gradually very gradually, but it I think it is something.<br><br>Int: Okay<br><br>Part 9: mmhh<br><br>Int: what intervention would make the biggest difference to improve primary health care?<br><br>Part 3: come again<br><br>Int: what intervention, like what do you think will make. We were just been talking about the ones which have been introduced by the government akere?<br><br>Part 3: mmmm<br><br>Int: Now what intervention would you would you, do you think will make the biggest difference to improve primary health care?<br><br>Part 4: I my opinion from what has been said so far I think rural development it is a very cardinal intervention. The only thing is that that has a lot of eh… cost implications and something that cannot be eh readily immediately assessable. So I think in in the interiam what has been suggested recognising that you know…those especially in the rural or remote areas are making a sacrifice. If that is recognised and attracts ah... appropriate incentives then I think it will be very major intervention<br><br>Int 2: Okay! Anything else….. That could make a difference<br><br>Part 4: Providing the required resources, like equipment, and staff… staffing.	

Int: Okay Building building of effective primary care team have been suggested as a potential intervention to improve primary care in Botswana. Now what is your understand of an effective primary care team? Remember the goal is to improve primary care. What do you understand an effective primary care team? Ee P1<br><br>P01 it's a team in which each member acknowledges the work of the others. And it's a team where by people come together, maybe to set goals. People come together to discuss issues pertaining to primary health care and pertaining to the hospital. So if we we do give those reports to say I'm in Maun clinic I go, I I I sit down with the DHMT head and I give him the overview of Maun clinic to say im working in Maun clinic and this is what is happening. I am seeing this number of patients per day or per month an and im im im facing this challenges but I have done this and this, I failed this because of this. I think this things could make us to be able to acknowledge what others are doing. But now I what I have realised right now is that a a kere ne go tulwe (they had said) the DHMT should be mend by the eh... the head should be public health specialist and most of the time you find you you see that public specialist is somebody who knows what is happening in the hospital and what is happening in the primary health care. But rona mo (But us in) Ngami our head is somebody who has been heading the hospital and I don't think she knows much he knows much about the clinics. He only knows the superficial things and he makes conclusion from those superficial things. He doesn't know exactly what is happening in the primary health care, so these are the things that make people that make, that can make people to to think that maybe people who are at the are not doing their job is not of that, ga e na mosola kana ga e na boleng as compared to the hospital. So these are the things, if we were being lead by a people who knows who who who... a person who knows from both side, I think things will be good because he will be knowing the primary health care is a unique care and if you don't know about it if you don't know about the programmes and you being called to say why are you refusing to to to give the the the the transport to somebody who who wants to do this for administrative duties when wena you wanted to make a follow up of the patient who did not come for a TB treatment. So she it should be somebody who is learned on this programmes and the public health specialist is the person.	

Int: Okay still on this team, a primary care team who do you think should be the members of an effective primary care team? What should the roles of the different members be? We ahev just talked about the primary care team and what we think it is, who do think should be the members of the primary care team? What roles should different members be playing and P1 was also talking about the leadership, we were to have a primary care who should be the leader? Who should be the members, what role should they be doing, who should be the leader of a primary care team?<br><br>Part 6: Rona kana re a dominator jaanong re tshaba go bua (*we are dominating and now we are afraid to talk*). But I also I will be biased because I will say the family physician should actually be the head of eh... primary health care team because eh... the nature of the training of family physician eh... entails going quite deep into clinical creative aspects of health care and also quite deep into prevention, and also into health administration its all incorporated into training of a family physician. So in my own opinion I would have said a family physician, but I I I stand to be corrected because I I would be a biased eh... respondent since I am in that field myself.<br><br>Int: Okay! Remember we said from the beginning there is no right or wrong answer and I I I think I would like us to talk more about this primary health care team, because its actually an intervention which has been suggested. It a potential suggestion which has been suggested, I would like to get your views on the team, like what you think like so maybe this time around I will go around to have everybody's opinion on what they think about a primary health care team and who should be in it who should lead it and if if and also touch on if we should have this team in every primary health care post or in every clinic? Yes doctors P8 you are next.<br><br>Part 6: Yah, I think the FMP is also the one who is supposed to to be there in the primary care because as the doctors are in the hospital in the primary care also they need someone who have those qualifications to be in the primary care. The FMP is the the the one for me I think that can be in the primary care not in the hospital.<br><br>Int: yah who are the other members who are the other who can form this team?<br><br>Part 6: yah! The other members will say the registered nurse, the pharmacy technician,	

the phlebotomists, because we are collecting also blood.<br><br>Int: Okay<br><br>Part 3: Yes<br><br>Int: eehhh...P5 what do you think about the primary health care team?<br><br>Part 5: uhhmm talking about a team I should think everybody should be implored in a team but then choosing I wouldn't real know like saying who should be leader or so on,<br><br>Int: Okay<br><br>Part 5: yes<br><br>Int: okay P7 what would who put would you, who should be in a primary health care team and what do you think about a primary health care team as an intervention?<br><br>Part 7: Are you talking about at at the grass roots<br><br>Int: Yes, primary health care now we are looking at the grass roots akere we are trying to improve primary health care even in rural areas. Now what im asking you is primary primary health care team has been suggested as a potential intervention now what do you thing about that? That that the the intervention of having a primary health care team and whether you think also whether you think we should have a primary health care team in maybe clinics or all clinics, all health posts like and if we do have a primary health care team who should be in it? Like who should be in a primary health care team, maybe somebody will say a nurse, a counsellor, this person, a midwife, this person, a doctor. So what do you think?<br><br>Part 7: I think for us to come up with a composition of the primary health care team we need to understand the role the team will be playing and to what extent will that team is going to be playing that role and what kind of power it is is is going to have. That's when we can come up with the components of the primary health care team and who should head it knowing all the roles that the team is going to be playing. So with that	

being said I I would like to just say that aaahhh for the team, there need to be proper involvement of all necessary stakeholders for it properly and effectively do its uhhh duties. Therefore, uuhhh for all the stakeholders i would say the nurses forming the interviews between the health care system and the community they are a very key player in in a primary health care team. The patients themselves, the community, whether they will have representative, they need to be involved when certain decision are being made for the community to know as well. We may what to say a councillor or a VDC or a the local chief, I don't know but that that person has to be in the team perhaps as, and also someone from the hospital to know what the patients are being referred to the hospital they need to know what goes on at that that grass roots level. So I will say that someone from the hospital needs to be there as well. A family physician like the doctor said they play a very important role in clinics and they have to be in the team as well, the public health specialists they have to there in the team. So, but there is a dif... ah eh…, composition of the team will vary depending on the role that the health team is going going to be playing so I cant…<br><br>Int: Yah, there is also a question on of the roles. Akere we are interested in forming, we know we want to form a primary health care team. There is also a question on what roles should different members paly if we were to form, we are trying to improve primary health care, we are trying to improve health care at primary level, now it has been suggested that primary health, primary health care there should be a team as a potential intervention now if we were to have that team. Who should be members, what should be the roles of different members, who should lead that team and maybe how should that team be assessed? Like the quality of the teamwork, how can it be evaluated and I would also ask the question of do you think it should be or it could be in each and every health post. So we are trying to get your perspective about the idea of a primary health care team. Ee P10!<br><br>Part10: In addition to what my colleague has just said i was thinking that we also involve the members of the community (coughing),The chief, the councillor, the religious or eh...a representative from the religious leaders, minister fraternity which is the religious leaders, traditional birth attendants they are there, the traditional doctors.	

**Int**: Ok! Ee P10! What do you think about the team? Anything about the team?<br><br>Part1: Umm..!In my opinion all cadres should be represented.<br><br>Int: All cadres?<br><br>Part 1: All cadres! If possible and having it at district level i okay i don't think it will be fine to have team at every health post because...actually a health post is having one person working there. So having it at maybe district level is fine. Eh..!As for who leads it, personally i don't want to prescribe to say doctors a what what... a person with interpersonal skills should be the one to lead and I think most of the problems we are having is that we are giving titles to this posts to say a permanent secretary should be a doctor i mean does it necessary have to be a doctor? Does it necessarily have to be a nurse? it should be someone who can unify this team because i might be a very very good doctor, i might be a good pharmacist but when it comes to leading a group of people like that its its its a very different thing. So what we are doing...we are pre... ...we are giving a...a profession to a post like that i...personally don't think so because, where i come from its different we don't prescribe a profession to...to this high posts we just need someone who has got interpersonal skills because he has got a team below him. He has got a pharmacist, he has got a doctor, he has got a nurse so the idea is to unify this people so that honestly so that they work as a team so honestly to say it is supposed to be this profession i don't know but personally i don't believe that.<br><br>Int: Ok! So P2 you had your hand up!!!<br><br>Part2: Yah! I just have eh... a little bit of different opinion in terms of the structure in...I would rather wish it were cluster based rather than district based. Because you find that most of the time So i am, i still agree with all the suggestion at all members in the house so far and some of the time when it is right at the centre of the district they lose touch of what is going onat the cluster level. So umh...i still agree with all the suggestions of the members who are here hare offered so far. Umm...in terms leadership well i think he has a basis like it was earlier mentioned it has to be someone who has a broad knowledge, someone who knows what is going on in the place. Eh...even though...yah people with good leadership can get information from all	

direction and still be able to administer very well. But i still think somebody who really has the depth of knowledge should be expected, the only thing is if he fails to provide proper leadership then there should be a form of way which the person can be asked to step aside because definitely leadership is part of the responsibilities attached to to...certain positions. So I think eh,,, instead of just having an open blanket approach and say or somebody can just be chosen out of the team to be the leader i would rather say somebody who is adequately qualified who is adequately exposed to all aspects preventative and curative and all aspects of the team should be the lead but if he fails to provide the good leadership there should be away of eh... changing such leaders

Int: thank you P2, ok, lastly on these team i was asking about how...

Part 3: (Jumps in) you didn't finish

Int: Oh sorry mam!, Ee P7! There anything else?

Part7 :Mmm! Ga ke ise ke ko ke tsholetse (I did not raise my hand)

Int: Ehee! Oh I was saying....

Part 3: Nnyaa ke raya gore akere you were going by getting our opinions on the team.I thought you said you will go around

Int: Ok i apologise for that. Ee P3! About the team

Part 9:Nna yame is like P1 has said

Int; Ee! Ehee P1 has already covered that?

Part 9: Ee!

Int: Ee P3! The Team

Part 3: Ee! Yah! Nna...because we are talking of primary health team not DHMT.I will maybe suggest the teams looking at the principle of primary health care. What does primary health care focus on? They are focusing on prevention okay? So who are those people who are on the prevention part...preventative part? there are nurses there, there

are community health nurse...community health nurses, we have a team as like the doctor has just said in a facility like a clinic we have got pharmacy technicians, we have got social workers and then we are looking at community involvement when we talk of primary health care principles we must engage in our communities in there be it VHC members village health committee members or VDC committee member or some members who are there who are actually in health related programs and then we are talking of multi sectoral collaboration involving other stakeholders like it has been said. We are also looking at ehh... appropriate technology. How will you work out...u know we are looking...we are living in a vast environment where now we deal with a lot of technology surrounding us. So we are looking at metric things like...equipments, because for us to perform effectively we need to have resources. We need to have thing which will keep us going or keeping us moving so when we talk of the human aspect there, people who will actually look at what we need...resources can be in terms of man power. Shortage of staff, how do we distribute looking at all the problems we have at the back there. If we have teams which involve the aspect of what primary health care is all about, the principles in that may we will take the facility as it is bo nurse, a social worker, pharmacy technician, doctors and then we move out to the community community leaders, VDC, VHC and then we look at other stake holders where we are who are those people who are are around us. And then we look at people who have got the skills human skills so that they coordinate and some of this things we don't have human skills, human skills, interpersonal to be in that team so that they coordinate because some of these things you find that we are in positions but we don't have the human skills we don't have the technical skills we don't have the conceptual but if we have people with different skills in there they can lead the team.<br><br>Int; Ee P3! And also touch on the quality of the team work like how...how should it be evaluated? The impact of their work be evaluated...what would you say about that?<br><br>Part 1: Okay like the P3 has just said gore maybe when you talk about this primary health care team we must maybe classify it so that... ke gore this issue needs to be decentralised to feed people above there because when now it is centralised you find that there are some nitty gritties at the grassroots level which may be will be presented	

better by people down there than somebody at the top. So then evaluations of this whole thing may be done quarterly. I don't know if the question is...am answering your question. It can be done quarterly, those people who are actually in the team meeting together. If there is a team in Shorobe, team in Makalamabedi, they meet with the tall people there they evaluate what is happening on the ground. Those people now will be able to assess and see where the weaknesses or where the loopholes of this whole thing is moving and then with people ba e leng gore they have interpersonal relations ba eleng gore they have expertise in analysing things they will see from the presentations or the evaluations done quarterly gore where are we lacking where how can we improve.<br><br>Int: ee P1!!Ee P1 The team, what do you think about the team, each member...the roles of each member and evaluation<br><br>Part5: I think they have covered up most of the things.<br><br>Int: Yah maybe you can elaborate further on the evaluation<br><br>Part 5: Eh...i think the evaluation can be done quarterly akere (Isn't) we are also trying to aline our evaluations with the people in advance eh...quarterly so each quarter we seat down and see how far we are, eh...what are our weaknesses and what are our strong points and how can we mmmh...how can we...re ka thusa jang di weakness tse di re di bonyeng (Weaknesses we find). Mmmm!<br><br>Int: Eh...do you have something to add P7?<br><br>Part 8; Mmm! Yah i mean...Just thinking about the team. Am not a good....am not into the idea ya support teams or you know the idea for teams or committees because most of the time they are just redundant.<br><br>Int; Eh.... please...<br><br>Part 8: I was saying i am not a a very big proponent or an advocate for teams you know because most of the time they or committees because most of the time they are just redundant they dont do much so if we really like to go...to effect change at the	

grassroots we need to come up with things that are relevant for the grassroots you know P1 mentioned about centrally placed eh..teams when we centrally place teams eh...most of the time they don’t understand what is happening at the grassroots so for monitoring and evaluation we need to come up and we need to come up with the levels and for monitoring and evaluations they need to have proper guidelines or proper documentations favoured for MMEs for monitoring and evaluations they need to be...they need to follow principles monitoring and evaluations. The team that is at the grassroots level needs to be well thought of. And it is not something that is going to be.....it needs commitment even from people from the ministry of health so it means it needs some sort of political commitment or so because it doesn’t...they are not going to get some funding. You can form a team but there is no money to make it work

Int; Yah! We have talked much about the primary health care...

Part 10; eh...Still on evaluation...

Int: Oh yes sir

Part 10; Just to add a little bit.... Umm! Wat i just want to say is that evaluation can be both qualitative and quantitative. Qualitative in terms of finding out where these...primary health care teams they are supposed to the delivering services to the communities so first of all the committee itself should evaluate them in the form either through interviews or through questionnaires. They need to get the feedback from the community are they happy with the health care team that is assigned to them that is one step. Then two, through parameters that are regularly submitted by the team you know they need to look at the indexes and see has there been relative improvement in the health indexes of that community since the team was assigned to that cluster. So i think those are two ways in which the teams can be assessed.

Int: Okay! Okay we have talked so much about the team. The next question is what is your opinion about building a primary care team as one of the interventions? And i think we have covered that...

Part; Yeah

Int: Yah we have just covered that. Now let's go to the last part which is interested in your experiences. Do you remember as a health care worker...we would like to know your experiences. Do you remember any problematic situation your work you found particularly morally difficult? Yes can you tell us about the situation, about what happened for example you had to turn a patient without treatment. Like where you work. Or maybe..,. Even here we are talking about health care in general. Or maybe...maybe patients wait too long.Can you tell us about that experience and why it was difficult in closing. Ee P9!<br><br>Part9: Nna i am going to talk about my experience of primary health care,<br><br>Int: Ee! P9 e bile ke kopa re kuke lentswenyana (raise you voice a bit)<br><br>Part 9: Ee nnyaa ke a lwala (am sick). My experience was when i was providing primary health care was at the district commuter of nurse in the Okavango that was in 1995 and we were doing a polio campaign and we were supposed to be giving polio drops to these children who are in the islands of the Okavango and we didn't have transport to go there. We didn't have a helicopter. But there were children there because people are moving into that island to go and and plough some maize so and it was during this time when people are in the field they are eating letlhafula so obviously there are a lot of children there so we have to go there and we were supposed to reach those children and we didnt know how but at the end somebody came with a suggestion that we should...we should maybe there was...there was a lodge, the owner was was ga tweng? He didn't have a problem of givin us...of borrowing us boat, an engine boat to... so that we can go there, and by then i...i was afraid of moving with a boat and going to the waters and i had to go there because i was a community health nurse so in the end it was me who was supposed to be doing that work. So ultimately we went and it was in the afternoon and when we reached there and we managed to immunise about 28 children by the time we finish immunising them it was already sunset and when we...when we came back, we found that the river was flooded. Where we were crossing that was little water, but when we came back there was a lot of water and we could not cross by the vehicle. So we tried to cross as we didn't have an option, we were in between the waters. So when we tried	

to cross the vihi...We were using a landrover vehicle V8.Our vehicle on the way, because water was getting in our vehicle we nearly drowned, water was getting in side the vehicle so the engine stopped and we were far away from the lodge, a little bit some meters away from the lodge and it was now, it was night and we started hooting. Hooting hooting and we didn't know who we were hooting and ultimately the BDF, that was eh... i think they had a camp somewhere there and they realised...the saw us passing by and to them they wondered how we was how we are going to... to manage to cross because they had realised that the waters were now vey high a vehicle cannot cross. So there was no way out so brought their big big truck to come and pull us out of the water and that was the end of my community service in that area and i told myself that i will never again go back even if there is something i will not go there. So it means the patients were going to suffer akere? Because i was not going to give the services again back to that community.<br><br>Int: thank you!!!! Is there anyone with any different experience that has actually one day maybe forced you to turn away a patient or maybe make a patient wait too long?<br><br>Part10: Yah! It happened like where i am working presently, its a facility where the motlakase is being generated by the generator when it is on and it is a facility where it is visited fortnightly for ARV clinic to see patients there and the experience or the challenge that we had there or that i had there is that sometimes the generator is off as it can go for up to 3 or 4 days down not working because of this relocation or this merging. This generator used to be serviced by the council employees when we were still under local government now because this merging now it is difficult to get the service immediately when you need it for the generator to be serviced when it has a breakdown. So there was a time whereby the generator which was off for 4 to 5 days. It was attended, it didn't work. The people came and tried to fix it thinking gore the problem could be here or that it didn't work. Now because...now now again we don't have ehh...before the merging we used to have the security guards of which when we were on call we would know gore somebody is on call when the patient come they will go to your house and knock and tell you gore there are patients at the facility waiting. And because of this darkness the generator was not working, the security guards were	

not there and its like some patients came to the facility at night. So they didn't know where to go, who is on call akere there in no security who would direct them or who would go to that particular individual who is on call. And then in the morning this guy came to me she said i want to see the nurse in charge. So into the consultation room at he expressed his eh...feelings concerning gore he came to the facility at night and then nobody assisted, what is the problem ,have nurses stopped working t night or what? And the explanation i gave is the explanation i have just cited gone gore that the generator was off is not working and actually when you come to the facility if there is nobody to go and wake the nurse who is on call to come and assist nurses wouldn't know that there is someone waiting at the facility. Some are brave enough they would go door by door knocking to see who is on call to come and assist. But this come just came to the facility waited there and then he went back. In the morning then he came and confronted me then i tried to explain. It was not good for him how it happened despite the fact that the explanation that I gave was for those two reasons that we have. So its like it became a problem they take us nurses as people who are not willing to help. And yet we are paid a lot of money, we are paid overtime and and other things. So if you are confronted with such a situation whereby tota some of these things are beyond your control you feel like eh...you will feel somehow...it becomes painful on you that you haven't assist your patient...you client, you customers and yet there was nothing you can do. The other incident happened whereby there was this pregnant mother who came to the facility and according to their story they went back, there was no one to assist and ultimately this ldy delivered at home. And in the morning they brought the baby to the facility for assessment. And then some of my colleagues they assessed the client the baby all of a sudden she started changing the condition and she started having blunting respirations not breathing well. Then she was referred to the hospital we came to the hospital she was assisted for a maximum of three to four hours and then ultimately at the end the baby died. Because of gone gore they came to the facility to find out gore who is on call to go and open...to go and tell the nurses to come and assist. So those kinds of experiences are actually not good because it brings a picture to our service that they are not good enough for our communities. And le yone experience hela ya gore you work in a facility where sometimes you are in	

darkness. Y ou don't even have a choice of going somewhere you are just stuck in your house you are just stuck in that darkish environment, maybe you are somehow....you you have your fridge there full of meat and the meat gets spoiled because the generator will be off for three or four days. Those kind of experience they impact negatively on us as health workers and as well as on our communities because the services which we are giving at them is like we are at fault but we are not the ones who have control of proving the resources that we need to service them the way they want. So those kind of experiences are somehow you know they are experiences tse e leng gore even if i move out of that area they will still haunt me a bit because if things happen that way where you are you feel like you are not doing enough for the community.<br><br>**Int**: Mmmh! Okay! Thank you very much!!!!!!<br><br>Part1: I wanted just to please voice an example...Am working in the hospital but i was attached to the primary health care. Once i went to Maun clinic. I used to...diabetic clinic i used to see the sugar level...everything. But when i went to Maun clinic there were no way...you will see...they will bring you the results of sugar level. And then i was trying to explain to my patient to say listen...As you are a diabetic you are coming for your review you follow up it will be better for you to have a sugar level for this day because myself i would not know if you are having hypoglycaemia or if you are having hyperglycaemia...but the patient was just fierce ''hey no! I gave you figures and figures! Serious? And then i went to the nurses who was in charge there and then i asked her what is happening here there in no sugar they are not checking sugar for diabetic and then they brought a meter for me without stick. And then i was just like...that day...and then we knocked off and i explained to the patient and then i referred the patient to the hospital for blood sugar fasting blood sugar or random blood sugar told them that there was no sugar treatment and brought a sugar stick and that day we knocked off and referred a patient to the hospital for the sugar or random blood sugar. The following day another police was there too, he saw the diabetic patient; he didn't check the sugar and then sent the patient back home. At the evening i was on call in the hospital and the other patient brought that patient with hypoglosemia and it was i...they couldn't even say out what the level of the sugar was...and i just said, and i	

said...i saw the writing they said the patient was in the clinic the same day in the morning at 10 o'clock but at 8 o clock the patient was brought in the hospital with high level of the sugar and i said to the patient now you see if there is no....in the clinic it will be better for you...just go to the hospital to check the sugar level there because if you know the sugar level, if that time you know your sugar level you not end up in that situation now that condition. As we didn't know they just sent you home, in the evening they bring you in the hospital like you are nearly a dead person. That is why i was saying in the primary care there is very...i don't know what is happening there it is very difficult to monitor the diabetic patients. Hypertension is better because you can have eh...a BP machine but the diabetic is very very difficult you cannot manage them in the primary hospital. Unless if they change the conditions there and then we will start managing the diabetic in the clinic.<br><br>Int: okay P2 and please you will be the last one so we can close...<br><br>Part2: okay!!!! Yah! I also had an experience not really at the primary care level but the patient was referred brought in dead because of a snake bite in the rural areas. This person well came already dead like i said but we had a situation where even if the nurse at the rural area had the appropriate anti venom knew what to give and how to give maybe that patient could have survived so it was a painful experience because i just felt this is a death that could have been prevented if the right things were made was accessible are at the right place so that's why i think if this things are at cluster level they would be able to say at this cluster we have lots of patients with snake bites we need this we need that we need that and it will help to make them more efficient.<br><br>Int: okay thank you very much and to conclude that there is anything that i have not asked that you wish i had asked? Okay there is nothing...Ee P4! Is there anything you would like to add to conclude just...a concluding note? Okay thank you very much for this participation your perspectives area very important this will help us to be able to address the problem of shortage of primary health care workers and i would like to apologise for the length of time i took on that you have given coupons...those are coupons!	

Part 4; No i just want to ask gore when are we going to have a feed back?<br><br>**The end....!**	
