# Supplementary material for: Stakeholders’ Perceptions on Shortage of Healthcare Workers in Primary Healthcare in Botswana: Focus Group Discussions
Source: PLoS One. 2015 Aug 18;10(8):e0135846. doi: 10.1371/journal.pone.0135846 (PMC4540466; doi:10.1371/journal.pone.0135846)
Supplement: S2 Text — (PDF) [file pone.0135846.s002.pdf]

Mahalapye Healthcare Users 2

Date: 2012

Interview Duration: 02.40.58

Audio File Name: Users of Health Care

INT: according to you, do we have enough health workers? If they are not enough what could be the cause? It is the first question, that according to you are the health workers enough? But if they are not, what could be the contributing factors? Yes P1….

P1: no! I think we do not have enough…enough health workers, if you look especially in the clinics, but what causes it! I think these days people get sick a lot more so that it even overwhelms these people!! We are very sick especially in large numbers, having different illnesses.

INT: Yes it is made difficult by increase in diseases. Yes P2..Did you want to say anything? Or someone to add on to what P1 was saying?

P2: Yes INT! It's like P1 said what I wanted to say.

INT:Ehee

P2: Yes INT…

INT; Does anybody want to add on what they were saying? Yes P3...

P3; for me I see that there is a problem in the clinics, because you will find that there is only a Sister, Nurse, and family welfare educator only, but there is always a shortage. The way I see it is that P1who said it could be caused by the fact that our villages have grown in large numbers, even in clinics from large villages, clinics and in the rural areas. But the way I see it, if only a clinic should have a Doctor from when it is handed over. Even the Sister and the Family Welfare Educator, I think it would be better. Even

right now we have a huge shortage for us in Home –Based Care, we do not have vehicles. All vehicles that assisted us have been moved to the central government. You will hear that there is only vehicle that delivers for Mahalapye, when you are engaged in home based care you will come across patients who stays at home, but like P1 was saying they would be in the clinic. To come to the hospital there is a shortage. The vehicle will be delivering this and that…It will now be based at the primary hospital delivering to clinics, there is a shortage, maybe they will call the one from Kudumatse, but it will also com with patients that it was already scheduled to transport from other rural areas, hence it is going to be difficult for that patient to be attended to, but while you have gone for home based care you will find maybe three patients here and there sitting and waiting, because it is the time for them to go see a Doctor at the hospital. But those patients will sit there until evening without consulting with the Doctor. If only we had vehicles for the hospital and for home based care they would help. I am telling you we are really pressed very much, regarding the Doctors!

INT: yes P3! But when you consider the health workers numbers, are they trained in enough numbers?

P1: I was about to go in to that one…

INT: you were about to go in to it? Yes P2…

P2: I was about to go in to it, to say…but the training of health workers is so low because now if you consider that you will hear that Pharmacy Technicians are trained for instance 7 of them in a class. But when you consider the Mahalapye hospital most of the people that are here will be waiting to be issued medication, but with only three health workers but the queue will be so big, I fail to understand what the problem could be if government is failing to increase…

The issue of training so many people like those who dispense medication, because at the moment there is that system where nobody is allowed to dispense medication unless they are trained Pharmacy technicians, but now there is a shortage of these people. If you could just look at the queue for patients who are waiting to be issued medication, you will feel bad for them. And to think that they are patients who long came to the clinic in the early hours of the morning and has since been waiting, because of the shortage of health workers. They will be waiting for the Doctor; they will say he is doing this! Later he went there! But while patients are in queue waiting, but then when the Doctor comes and realize how many the patients are, him being one! If it was possible for the Doctors to be trained accordingly, so that

one deals with eye care, the other TB, while others deal with other diseases. They should be trained, and they should also specialize, not a situation where by I will say my lungs are painful, my head is aching and so on! So that they do not confine themselves to all these. They should be trained according to theirspecialty. Patients should not continue queuing like they are doing nowadays. If you see the situation today! We are just about 2 million in population, but when you look at the number of Doctors it is really difficult. And if you also go to clinics you will find a prenatal care nurse having so many pregnant women that you will wish there were two maternityrooms. You will find the nurse doing the consultation being alone, yet there are many people outside waiting. The children you undergo weighing to determine their health will be attended by the same nurse, more so that people wish there was a family welfare educator who will assist the nurse with the weighing, so that when they need consultation with the nurse they can go straight to the nurse, so that the queue can get moving. At times the nurses allocated are not equivalent to the patients in that particular area, for instance Mahalapye can be allocated about 10 or 4 nurses. That is why when you go to a clinic you will find a nurse consulting, at the dispensary, and doing other tasks. So we are requesting that health workers can be trained according to their cadres and they should also be increased to reduce the queuing of patients.

INT:Yes P2! We are grateful, is there anybody who wants to add on to what P2 was saying? Yes P3!

P3: yes INT! I am adding to what P2 there was saying. You will find that in a clinic there is only one nurse, only one! While that nurse is still consulting with the patients that are there, but they will also be injecting some patients...at the dispensary too...if there is a patients who is in critical condition the nurse will have to transport such a patient in an ambulance, maybe from places like Pilikwe, maybe from Moshopha, Maybe from Pallaroad, or from Dibete, they will then bring a patient here and find that there is a huge line. Hence the patients that waited for her will have to leave with sore hearts and with their illnesses. Some with high blood conditions without medication, without anybody to give it to them. Now let the number of nurses maybe so that they are 3 or 4 in the clinic.

INT: ok...Yes P1.

P1:you will find that like…I will give an example of our situation where we workwith places like Mhalapitsa, Maapye and Pilikwe. You will find that they….the nurses in Pilikwe are 3, at Mhalapitsa they are two nurses…you are on leave and then someone should take your place maybe from Pilikwe to

Maapye, one from Pilikwe taken to Mhalapitsa that will mean for us as we remain with 1 nurse, even though there is a way of consulting. Blood tests have to be done in a particular way, more so that it will be difficult for that person alone, resulting in people waiting for the whole day. The day when they will be together, because you said we can speak freely….

INT: yes it is like that P1…

P5: even when they are together they are always be playful, you will find that most of the time the nurse will be talking onthe phones when they are suppose to be working, not that it's for personal usage, but they will be called to the phone while you are there waiting, and then go elsewhere , where they are needed for….at the caravan where pap smear is done, she will go there while you are still in line maybe you are the first, you are going to end up being assisted late.

INT:yes P5, to ask on to the question you have been answering, because I am looking at how nurses are distributed, the way they are distributed in hospitals what can you say about it? How do you see it? The normal posting of nurses in hospitals, what can you say about it? Eee...Let me take those ones that have not said anything….

P3:thank you INT, the way they are…the way the nurses are distributed in the clinics in rural areas, really the situation is worse, and they often bring people who are still young, more so that someone does not know how to handle an elderly when they come for consultation, it's like they…its like they are uncomfortable more so that…its like hospitals in rural areas. They could give us more nurses that are mature rather bringing the youthful ones or at least they should be mixed.

INT: they should be mixed?

P6: yes INT...

INT:I mean even with the distribution, even the numbers that are given to the clinics….do you think they are allocated equitably?

P7: no INT... I do not think they are allocated equitably! Because where I come we have two clinics,Shoshong clinic and Shelaketla clinic, but you will find that around Sharewood where we will like to think that is where there are greater people, they have been given youthful nurses. While at Sheleketla, there is only one nurse, same nurse if someone is critically ill she will be the person to take the patient to the hospital, we call it a big hospital at Shoshong clinic then it will mean…that the patients will not have a nurse then! Maybe there would be many students mainly because Sheleketla is closer to the senior school, then you leave them to go there….

INT: yes…we are grateful P7...someone else to add on to the issue of distribution of nurses, or maybe something different... yes P5...

P5:yes INT...mmm the way the nurses are distributed is even difficult on the nurses themselves, because you will find that these nurse...they...they are all over the place not knowing what to do. Sometimes you find a nurse working alone while there are pregnant women and other patients waiting. Hence she ends up doing many tasks resulting in problems for her. And then we will say the nurse is lazy and she does not know her job, without a way to divide herself due to the shortage of health care workers. We should really talk about increment of nurses so that when one goes to leave the other is not left alone.

INT: yes P7…

P7:well it's like P5 has said what I wanted to say! I was saying even them...even here in Mahalapye there is a shortage of nurses in the clinics, more so that patients will complain, then we as volunteers will like we have been saying only one person cannot manage the job.

INT: yes P7…I mean like you are saying there is a shortage in Mahalapye, is it in all the clinics or some them? Or you may find that some have been given many!

P6: there is a shortage in clinics, there is a shortage in all the clinics, but outside its worse, outside the village it will only be one person.

INT:Only 1

P4:you will be only 1, now here in Mahalapye there is a shortage because firstly we would ….because in the past we used to see family welfare educators at the dispensary, they no longer inject patients, they do not dress wounds, going to immunize children on her own, now there is a shortage when they were distributing….nurses should be allocated in small groups not two or three of them.

INT:yes P2..

P2: to add on to what was said. …I will say what was left! At the weighing room you find family welfare educator. These days the paper work is thorough, unlike first when a child would be weighed and be determined if they needed to be given supplementary feeds. These days there are injections that should be taken. Hence if they finished with the health care educator they could go straight to the nurse. From there the family welfare educator would go to the store room to issue the suppplementary feed. Even though at times some would lose patience and start arguing saying they have long been waiting for assistance. I mean this job is really difficult; there should be a lady who does the weighing, one who records, and one who issues the supplementary feeds. The job getstoo hard for them.

INT:Okso you are saying there is nothing like someone has been given a lot of nurses? They are always few, there is nothing, bottom-line is that there is a shortage.

PALL: yes there is none! It’s all the same…

INT: Ok

P4: now the worse part of it, since these family welfare educators…they never say we can hire you with a contract so that you can work for some time, and there is no…they are no longer trained, when you go to the department of child welfareright now, the worse part of it is that, they are no family welfare educators??? They are no longer being trained, when you get to the department of child welfare whom do you find there? Because now you find only the nurse doing all the functions. If only the government had left the family welfare educators in their former roles like they knew them better. It would make the situation better, and it could also be reducing the nurses work load.

INT:moving on! What do you think can be done so that the health workers who work for a longer time in these areas? How can we attract them to come and work in some of these areas? I will get back to you, let me start with P4 there….

P4: I was saying maybe when family welfare educators should be reinstated; maybe the situation will be better. The shortage in clinics at times is caused mostly by fact that there will only be 2 nurses in a clinic, the other one weigh, and will also supply supplements for the children and also taking temperature. While still performing these functions they will then be taken to Pallaroad, and only one nurse will remain, The remaining nurse will remain massaging patients, consulting, weighing, at the dispensary….ee while giving medication now this will delay her and it seems like they are lazy and slow, she does not know her job, now outside we become impatient but while she is alone, its better if the government would reinstate family welfare educators, to help the nurses maybe it would be better.

INT: thank you P4! Let me repeat the question again. What do you think can be done to attract workers? Yes P1...

P1: repeat yourself! What do you mean by attract?

INT: to attract people, to call nurses to come and stay in these places and do their jobs. What can be done?

P3: at times you will find that …maybe a place like Pilikwewhere they do not have their luxuries, they have a shortage of…of...it’s a village, then it would mean then the nurses would have to be increased to stay in the three houses for nurses, so actually I would say it will be better if even accommodation for nurses be increased so that they can live comfortably.

INT: yes, someone else!

P1: even if it’s like that, even we as the nation needs help, let place ourselves in a situation where by we have a shortage of nurses but in a village like this…let me give an example of a village like Pilikwe, let there be parents who will volunteer to give nurses….

I was saying even us as parents in the village since we want worthy help from Doctors and the workers there, so that when there are three houses in the clinic for three people, but we wished that they could be 5 instead, there should be I do not mean volunteering to say you will not do anything much, there should be parents who can say I have a house and a nurse can pay the same amount that is paid by those at the clinic houses. So that we get help because at times we become selfish, that why you will find that nurses what do you call it?There is a failure to bring workers to us because of when workers are supposed to be deployed here I will say my house rent is seven thousand pula. What do they earn? You see! So we as parents or committee we should play a significant part.

INT: anybody else who want to add…ee...P1 I will give you a chance…anybody who wants to add?

P1: me?

INT: yes…you will have an opportunity! There is no problem. Yes P6!

P6: no... I was …was adding on what she was saying, in her own words that we as parents we are the ones who are sick, we are the ones that need the nurses, we are the ones complaining that there is a shortage of nurses, now we should have the care and patience to attract them to come and assist us, so that they take us out of the situation we are in.

INT: yes P2!

P2: I was saying that you will find that in Pilikwe there are houses in the village like P1 was saying, but then the nurses would not want to share but prefer to stay alone, that is the only one that I want to say; that they could have the love to accommodate them because as a parent you have some things they might need like electricity and other things.

INT: yes P5! Regarding the issue you are talking about, I want you to explain like you said when there is no electricity, so that we know how they see it.

P5: yes INT, there is no electricity, there is no bath inside which they want to bath in, instead feeling about taking a washing basin and dispose the water afterwards. Hence they should be given houses that have everything in them; they should be given their houses.

INT: Ok...anybody to add? May we pleasemove on to the next question? Do you think there are gaps or shortages regarding health workers in clinics?(repeats the question)

Like maybe you can think it's because they are not trained enough and efficiently.

P4: I was adding on to what P1 was saying, like the P1 is saying that our children used to go to TireloSechaba (Community Service), but Africans have considerate hearts because if you could go to a village likePallaroad or Setsile if there are 5 nurses are going to help with the nursing job, I do not think a Motswana woman would deny such people accommodation not after the National service (TireloSechaba), because elders those days they would give them houses at very low fee. Because they were talking about how people now demand a lot of money, but if these workers could find houses before they can be offered one by the VDC, or if government is still planning to build houses for them, it will be a good thing. It's a beautiful thing. Some of them would be just Batswana children who just want to enjoy work and get paid; there would be some who want to those bathrooms with bath tubs. Most people are mislead to think that when they hear there is a nurse coming they would decide to increase rent , so I think even us as a nation we should be considerate when we hear that we are given a nurse, even though there might be some who wants electricity, some will opt to be like Batswana children. So we as parents should refrain from stingy behaviors to want more rent money, so that the worker can continue working until VDC or government to offer them accommodation.

INT: yes P1, I want us to get back to that question where I was asking whether you think there is are shortages and gaps, or problems are related to the health care workers?

P1: HeyI think Idonot understand you!

INT: shortages…maybe you might see that there is a shortage caused by people are not being trained in sufficient numbers? It is the kind of problems that I am talking about.

P2: actually when we are looking at the training of nurses or maybe the Doctors, let me say health workers in general. So when we look at the government's consideration when training health workers, that it considers the budget, consider how many can be trained,

Then not consider…resulting in training a few people. If there was first priority that nurses need to be trained, Doctors can be trained, Pharmacists trained, Lab technicians trained,

And others to be trained, so that at the Ministry of health they will people will be considered ,so that it is given priority especially during the Budget allocation, like the ARV Program, so that the other amount should be allocated to schools such as IHS, so that it cannot fail to train about a 100 nurses maybe at school. It should not fail to train 80 students in Kanye, so it takes a particular number here and there. So since Gaborone is training Doctors, it should not fail to train at least 50 Doctors,

So that they consider that there is enough money allocated just for training. So what makes the shortage greater is the shortage of money allocated for training. I am wondering why there is training in fewer numbers not taking more people so that the country is saved. So that the nurses also work at a nice pace that does not pressure them. I do not know if I am saying the right thing that won't get me in prison tomorrow.

INT:no P2...those are the ones…there is no problem, to add on what we have talking about, yes P6!

P6: yes! I was saying there is a shortage like you were saying with regards to Doctors, Nurses, and in all the health workers. But if we could look closely you will notice that some health workers leave the system to be independent, while some of the nurses leave for overseas. That also brings shortage like P2 was saying that if training is to be done it should be done in larger numbers so that if there are those who will leave, there will not be any shortage, so that the shortage would not be evident like it is to every Motswana. In the Botswana health system there are many gaps because you would have gone to a clinic from 7;30 am ,but by16;30 you will still be there without being attend to.

INT: do most of the people decide to leave in large numbers? Is it a problem that makes the shortage of health workers low?

P6: what is evident now is that as there is shortage of health workers and others continue to leave the shortage is getting greater. If there could be training of a group to ensure that as others leave there are spaces that are left, like the clinic at the air-strip,

The clinic let her go, the very same one with the most patients, who come from different smaller clinics from around the area, but you will find that there is only one Doctor. There would be only one Doctor where there is so many people waiting.

INT: now we are talking groups, Do you think they have been trained enough and doing the jobs they have been trained for? Or is it one of the gaps for health workers that we want to find out if there are there? From the many workers that have attended to you when you had gone to the clinic did you think they were well trained for their job?

P6:it is exactly what I am saying that different people could be trained, if it is a midwife or premartenity nurse they should be trained for such a job so that they focus only on it. So that when they are still doing the prenatal care she will not be requested to go to care for other patients because there is nobody attending to them, That why we are saying there is a shortage, that is why we were saying many people should be trained so that pressure does not fall on the few that are there to reduce the chance of getting stress. More so that you will hear that the person is mentally sick. Due to the fact that the mind was overwhelmed, just that when I wake up I will go do this and that, you spend the night planning, instead of resting. Thinking that tomorrow there won't be anybody at Pallaroad, and also Radisele. Later they will be yawning at work, which is not a good thing. That is why we are saying people should be trained in numbers that won't keep others and even those that came to the clinic under tremenderous pressure.

So that I know that when I get there I do this. Also there should be two Doctors, so that the other can do this and the other does something else. So that patients can be attended on time to see a Doctor. Like P2 here was saying that you will go to the clinic at 7; 30 am hoping to be the first one in line, it will never happen. The other day a man said he came here at…when he got here at 4.30 am and found a huge queue…it was so long as if they woke up at around 3 am, so that they become the first, he found he was number 87. That is why many people should be trained. The government should put aside money to train many people so that the ones that are already doing the job should not have…so that the government knows that it's responsible for people. Knowingit's responsible for people who must live and also be productive members of the society and to develop the country. Like right now they are talking about poverty eradication. That is why we want more people to be trained.

INT: ok! Just to be sure, I want to know if health workers are really doing what they have been trained to do? Is what they have learnt at the school is what they are doing at the hospital?

P3: the way I see it is that workers who were from training, like at Pallaroad, our nurse is lazy that you will never know whether what they are supposed to do. She is so lazy that she can consult and disappear this side on the phone. While there are patients waiting. At times are think they are the ones making their jobs more difficult because they will not know what they are supposed to be doing. Just to dress up in a white uniform and think that she is a nurse.

INT:yes P2! Let’s move on. Are the health workers doing exactly what they learnt from school? Like when you are posted to Palaroad as a midwife, will you confine yourself to what you were trained to do? Or as a nurse doing what a nurse is supposed to be doing? Yes P2…

P2: no, actually in clinics it does not matter! The nurses there are having it tough, if they are trained for prenatal care, but if the patient comes she will attend to them. But they are not confined to midwifery that they have learnt.

INT: anybody to add on to what P2 was saying…

P4: actually telling the truth about nurses in clinics, some of them they do not know anything about caring for pregnant women. In 2010 we had a prenatal care nurse in our clinic in Baitiredi, but when she was not there, pregnant women will not be attended to. Hence they end up going to the Madiba clinic because there is no one attending to them.

That is when I realized then that it means the nurse was trained for prenatal care for pregnant women because when she was not around they would not be attended to. But I do not know if there is any truth to it as women were requested to go to the Madiba clinic or beasked to come next week since she will be in then. Now we do not have proof that she has been trained for such a job because we see her attending to other patients and doing all the other functions.

INT: Ok, yes P6…

P6: I am just happy that there are experts who have come here to find the truth. Just recently somewhere someone was prescribed medication, but now the person who was suppose to be issuing the medication was not sure what was written, so the other had to tell her what’s supposed to be issued. The same thing happened to me the other time as I showed the person the medication that I am

suppose to be issued, they wanted to issue medication that I had received in February from the main hospital. She would hear from me who did not attend school. When you are in home based you are most likely not being educated, but you do understand what the Doctor had written on such a day. Yesterday one of the nurses was shown by someone, so I decided to keep quite because when you are working with nurses you cannot say you did the same thing yesterday. Some of these nurses who issue medication most of them are not well trained in issuing medication because they are required to perform many function because they would have been placed at Madiba or Baitiredi clinic. Since there will be shortages she will be required to fill those gaps since they are employed. There should be different cadres so that when people are requested to start in April they will know that this one deals with eye care, the other deals with children and the other for maternity and so on! As we speak now we are beginning to have a shortage of family welfare educators who are suppose to be going in to the field to check on patients. Some of them now are doing the nurse duties, but while the education they have is not ideal for that.

INT: yes P7…when looking at the different cadres of health workers, is there any particular cadre that you can say has more shortage than the others? More so that the shortage becomes clearly evident.

P7: there is a shortage! Even if you go for consultation at the clinic, and you request for an x-ray, they will not write anything on the card more so that when you get to a hospital you start the process again consulting. Because when you have been seen by a nurse and you were referred, they should have written something that will inform the purpose of your visit. Now it like you has come to try things again, instead of a Doctor issuing you with medication. Now you have to restart the process of consultation. Or maybe requesting complete body checkup or x-ray, you will find that they are things that are all mixed up. Maybe the relevant prescription not written!

INT: yes P6…

P6: yes INT, yes I think there is a greater shortage of pharmacy technicians. Like now if you go to clinics...there is a shortage of personnel trained for issuing medication. More so that they are adequately trained to thoroughly understand the side effects of drugs;it’s not like me who will be told that a paracetemol kills pain. But they do know that a paracetemol is a pain killer, but what are its side effects? Under what conditions are you suppose to prescribe it? So i think every hospital must have more pharmacists more so that they will also work in shifts like nurses, so that when others are not there they can be covered! Since there are patients who will come at night and require medication.

INT: yes! What are the cadres that have a greater shortage than others? Is there anybody who wants to comment?

P7: I want to comment on the one of shortage of health workers. Really there is a shortage of Doctors especially at the clinics, because you find that a Doctor might come to a clinic once...but while the nurse needs the Doctor's recommendation for you to be reffered. However at times you can request her not to send you there because you do not have transport money. Since I am going to spend the whole day here without any progress, I do not know if it's because of laziness. I do not know if it's because of the shortage of Doctors? If at least the Doctor were requested to visit clinics at least 3 days in a week, it could be better as it would reduce the congestion here in the primary hospital. So I am saying there is a shortage of Doctors in the clinics. Now if Doctors and pharmacists were to be placed at the rural areas

INT: yes P7…Do health care workers really receive enough help such as resources? Yes P6...we can talk if we have raised our hands. Whether health workers do receive the assistance they need?

P6: yes INT…is you saying I talk too much?

INT: no P6! I am not saying that. I realized how quite they were so I wanted them to participate. You will comment again later!

P7:thank you INT! No I do not think they do receive enough support in terms of resources because there are times when we needed something for a patient that you we could not have, they will say they are still awaiting for orders. Then the patient will start to be angry accusing us that we are not helping them despite being informed that we are there to assist them. The health workers do not receive enough support in terms of resources. Thank you.

INT: yes P7...

P6: no, the health workers do not receive support in terms of resources. This is because you can take a diabetic to be checked, but when you get there when they are supposed to perform tests they will tell you tell you that there is no machine that tests. Even if someone has to test for other ailments they will always tell you that they do not have the machines to test. Therefore indeed the health workers are not receiving enough support in terms of resources especially at the clinics.

INT: yes P2...

P2:Ee…at times there is a shortage of medication itself. You can be prescribed medication that is not there at the clinic. Then you will be requested to go get the prescription at the hospital, of which there is also a shortage there. At the end of the day the patients will be harsh on the person at the dispensary, mainly due to the discomfort they are under. Maybe if it is a case of high blood pressure or diabetes where they encourage you to take them on a regular basis to reduce the impact of the disease, now they cannot issue you with the medication because they are not there. What are we suppose to do? Obviously the person is not to blame, but they are the ones that are going to face abuse from patients. So some other times they are not adequately supported in terms of resources they need.

INT:All right .. what can you say about the work conditions and renumeration ? what can you say about them? Are they alright?

P6: to the workers?

INT: yes P6!

P7: I think remuneration and positions they are…are…remuneration is not enough, because there are times when you have gone to the hospital and …they…Do you mean payments? (INT: Yes P7) because there are times when the nurses are on strike demanding salary increases, that is why I realized that there remuneration is not enough, because after that you will know there is no money. So I think there remuneration is not enough.

INT: yes! Anybody to add on to what P7 was saying?

P6: we are just going to speak without having done research that mmm…what the starting salary scale for registered general nurses, and also considering family welfare educators and Doctors. But now if you come and guess about their income you will be surprised that maybe you are…because of this reason, it's clear that they are paid based on how long they have been in the service. Then we should really…we should remember the Lord. It's because we depend so much on money, the same money that sold Christ, if we continue to depend on money there is no way the country can develop. We should as Batswana children we should know that we are serving our nation. But we should tell ourselves that irrespective how little the money is, it's better than someone who is not employed. But people are always complaining that they are not well paid hence they do not work as much as they could and end up being required to do many things at a go without equivalent remuneration. You become a pharmacist, Doctor, cleaner, and other tasks more so that you wonder what you are since they will be doing so many tasks at a go. That is why they will be complaining that they are not getting enough. If only a person did the job they were trained for, for instance, a midwife should assist in giving birth so that after that they record what they were doing. Because I don't think there is anyone who would just demand an increase without justification.

INT: so it means you also agree with the issue said by P7 that remuneration is not appropriate, they are not appropriate as you are giving reasons because it is caused by doing other tasks that are not for them.

P6: yes…

INT: Ok...

P4: I differ with them somewhere, I have a very sharp eye, I want government to ensure that every year when someone went to work… it's the same as when a child gave you water you just say thank you, it's the same as when you knock off from work and your younger sibling has cleaned the house you say thank you. But what I doubt is that government every year should consider increasing payments, because what we can remember from the past years at the beginning of ever year they would be given something. I do not want to know about thereremuneration because there is no nurse here, there is no Doctor because we could be talking about their output. If you had talked about us volunteers I could give it my all. I am touched by every Motswana, be it you are at the wild life areas, at university, at banking, where ever you are. Every beginning of the year there should be some action taken by the

government or the president to know how this child is handling their job, It's like when I am knocking off from home based care, when I have a sister in the clinic and when I greet her or when I go to her to report something and she does not have a friendly face that I actually I am serving my country mmm…but I am not getting anything, you should really write it that Batswana are crying about their children who work, even when you are a volunteer. At the beginning of the year everyone should be given something, starting with the person working at the Office of the president. I am saying this because I am not really happy…these people should get something.

INT: yes let's move on, the next question. Is there a problem of shortage of health workers in the rural areas? If it is like that what could be the cause? For example we are considering things like resources, could they have an influence in the shortage of health workers in the rural areas? Yes P6…

P6: thank you , yes INT your question is clear, the answer is that there is a shortage of resources health services, even at times there is a shortage of accommodation, there is really a shortage of accommodation more so that now at Shoshong there are nurses who stay in Mahalapye, there is no where they can stay. It is one of the things that greatly depreciate the health situation, resources together with shortage of accommodation.

INT: Yes some resources or certain development that are lagging behind. Well you talked about accommodation! What can others add?

P2: yes there is a shortage of resources like vehicles that transport patients, at times patients do not have the taxi, and then they will spend some time there without anything to bring them.

INT: the workers at the rural areas, how is their transport situation?

P7; even workers at the rural areas when a patient is supposed to come here they would say there is no transport.

INT: yes...I mean how their travelling around the village is, do they access to transport?

P6: yes...even their transport...they are also far where there is no transport, when they are suppose to get their belongings when its month it becomes difficult because they take a long time to find transport.

INT: yes P4…

P4:even there is a shortage of medication, more so that you find a nurse not knowing what to give to patients, medication and resources for us people of home based are in a critical shortage.

INT: yes INT! I mean the resources in the villages where the workers will be working. How are they?

P6: they are not there, there is a shortage, and furthermorethey get them with difficulty.

INT: Yes P6!

P7: but at times you can find that, you will find that…by resources do you mean resources that nurse's use in the hospital such as pills?

INT: yes P7, I also mean the one's workers need, like we discussed the issue if transport that you will find that some are so far away where there is no transport.

P7: yes, but including what I said like pills!

INT: yes P7…

P6: you will find that those pills the person who is ordering them, will often take time in ordering the pills before even considering what is there and what is not there! It's because of laziness more so that they are not there, because it would mean that at the rural areas you will first order while a patient here needs the pills, but they were not ordered. It is the same for some of us who have diabetes when you are suppose to be checked they will tell you the machine does not have a battery.

A diabetes machine can be ordered right! At times you will find that it has been taken for maintenance and you will be told you can come for tests on a certain date, but when you come you will be told the machine is in Maapye. At Mhalapitsa where you will end up missing the checkup-up, or at Pilikwe or other places. At times it is because of the delay if health workers in hospitals. No.. I was saying at times you will find that they their situation is made worse by some of those villages that do not have electricity, now it's an issue for a nurse to stay in a house without electricity. Without an option to buy what they need, you find that they are very stressful.Because you will find that even when a nurse was suppose to stay here they will request for transfer. Or you will they have gone to other countries. They have gone for greener pastures because where they are were staying the conditions of the environment does not favor her.

INT: Mmhh, what can we say about faraway places? Does the distance have an influence? Yes P7...could we please say something.

P7: yes INT there is an impact because at times someone is working in Mokgenene, but she has to come get resources for the clinic for patients to be attended to inothervillages, but there is no transport that she can come with and return back. They will then have to ask for a lift coming here to order and maybe be told that they will come on a particular day.

INT:Mmhh , yes P2 we have been talking about faraway places.

P2:ee...thank you INT, these faraway places have an impact as they are making things hard for the nurses, ever since the health services were transferred to the ministry of health, there are absolutely no vehicles more so that workers are having a difficult time regarding transport.

INT: yes P2, when we consider the commodity goods at the rural areas are they…

P7: let me… let me complete it by saying, even placing people at the rural areas contribute, that why when a person is told they are going to Makgenene they would rather resign. The government will place someone there for 5 to 6 years, why are they not at least placed there for two years, then someone else also for two years. So more so that later someone does not feel they have been demoted what do you call it? Something like that!

INT: yes P7…that you have been isolated. Considering the prices, how could it be affecting health workers?

P6:Aahh, let me speak?

INT:(laughs) yes P6 speak…

P6: it does affect them, due to the payment they receive. If they go in to a shop, they will find that in a shop you will find 5 kg rice being sold for P110, while 5kg being sold for P70, something's irrespective of how much you earn you cannot manage there. The shops in the rural areas are very expensive. Firstly they will be telling you that petrol is expensive. The distance of going to buy stock and returning is also expensive. In acquiring the actual stock is already expensive at the wholesalers. Wholesalers are also expensive like tuck shops, but now it is difficult to go buy hoping that you will get profit these days, so it would mean if you had bought a commodity at P110, you will have to add P50 to make up for additional costs.

INT: yes...P6to add…P4

P4: there is a correction, clinics even these ones…I am adding on to what that P6 said regarding the diabetes machine, we have a clinic that is more than 30 years, but as I speak to you there is no electricity, but the biggest clinic which is in the middle of the village named Baitiredi, it does not have mere electricity yet it is central to the village. There is no electricity, there is no toilet, and there is not even a shade where patient can sit. We have tried to speak to the VDC, we called councilors, and we even called the chiefs without any success. When people have come to the clinic they just sit in the sun waiting for the Doctor. It's only the small clinic that you can find a nurse a here, even the ladies that

weigh, supplementary food was left out because there is no space; boxes are laid for babies so that when they are being dressed so they can be laid there. There is nothing that is there, we have difficulties.

INT: thank you P4…yes P6!

P6:the problem is the clinic from..from.., from the beginning it was a place for people who are.. there is something calle d development trust, but when it was things are getting worse thet were taken over by the council so that they make it a clinic because there was no clinic at Madiba next to the school.

The issue was that it was to be changed to the standard of a primary clinic, so we do not know what delaying its transformatifon in to a primary clinic. It used to belong to the Trust, because the Trust had build some houses knowing well that they are settling there, that is why now its condition is causing concern.

INT: im very thankful ladies and gentleman! Let go back to that one of prices in the rural areas, was there anybody who wished to add on to it?

P4: actually the health workers in the rural areas are having it hard because you find that even when they have come to Mahalapye to get access to the banks, even when you try to buy things in bulk and you happen to forget certain things upon your return, it will be expensive for you to go back and buy those items.

That is why a worker can refuse to go rural areas when they are posted there because of the expensiveness of the place. In addition the lack of transport, because when they are supposed to come here there is nothing that can transport them. Maybe having left your child here because there she might get sick...there is nothing you can do because there is no network, you find that in the rural areas there is no communication, communication is very important these, but at itmes you will find that someone is in a desert where there are no phones.

INT:yes P4...

P4:maybe where they say they will get a ride from the clinic vehicle at least to buy a few items at Spar, but that is also a problem because if the inspectors can find that driver he will be fined, he will get fined for stopping in a place where he was not suppose to, and that vehicle is not for groceries. Then tell me which other vehicle will the person use, where by the owner will say you have a lot of groceries hence you will pay for one more person. Do you get what i am saying?

INT: yes P4. Does having or not having an opportunity to further your studies have an influence in the rural areas?

P7: there is no way you can further your studies, firstly for you to further your studies there need to be teachers like for senior school. Like right now those that live in mahalapye go to school at Mahalapye Senior, some are at Trust being taught by teachers from schools from places likeParwe, those from BOCODOL are taught by these teachers, they can develop themselves academically because when they knock off they can rush there! But when you are in the rural areas how can you develop yourself? Being helped by who?

INT:yes P7…anybody who want to add?

P6:but it is like that because there is no way you can develop yourself, and it can also influence people's decisions to want to work in the rural areas. Now you will be whereby when you knock-off to go home there will be a knock at the door saying the key to assist a patient, come do your work aaah!

INT:yes P6, anybody who wants to contribute?

P4: when a person stays there for 5 years without developing themselves academically, even that government job that they are working it's now like someone was heading livestock instead. Because when you are taken and be transferred you will be better enlightened , but if you could be taken to a place where there is no opportunity for further studies it would be like someone who went to plough and then the rains don't come those seeds die. But if there could be a secondary school or where one can be taught, the clinic should be close to the school so that the nurse there can go increase their qualifications like University. The same ones that helped our children who are now in other countries. I think we need education for the nation.

INT: is the opportunity for jobs for workers could it have an influence? Family maybe as a man who works at that rural area, but your wife having an opportunity to work there. Yes P7!

P7:yes INT...I think an opportunity like that is not there because when we speak of nurses she will be working at a clinic at Mokgenene since it is the only one there. But now if your husband is a teacher in a junior school, but there is only a primary school there, that means there is no way your husband can have an opportunity to stay where you are, there is a problem!

INT: yes let's continue, we are also considering the children, and school for instance.

P4: no INT…that one irrespective of whether an individual works in the remote areas it's not in our agenda. If a husband is a teacher at Madiba and the wife is a nurse there is no way you can be transferred to Madiba so that the wife stays with him. So what if you are placed at Mokgenene while your husband is placed at Madiba, where there are no English medium schools at Mokgenene where your children can attend, It's what will make you stuck there while your husband is burdened with taking care of the children because you will want your children to attend English medium school. That why there is disintegration of families, when he hires a maid that the end of the marriage. It won't be good because the wife will be at Mokgenene.

P7: I cannot say much regarding the issue because I can hear that if… you said effective health care teams? We are talking about primary health care but like we were saying that we have moved to central government rather than somewhere where it is not clear what primary health care is. Where will we end? But I would wish that Primary health care management, administrators should also…because their decisions affect those at the bottom. But if it could be our team including everyone up to management, like we are talking about the issue of transport, it also affects me while I am at the clinic. But the person who is responsible for transport is the transport administrator based at the hospital, I do not know if they meet with CTO? But now you see that if it ends with us our grievances will remain again with us, because I feel that it is a team that we will be airing our grievances. So that we come up with solutions so that we are able to improve our jobs or our workers or where we work.

INT: Ok... anybody's views regarding these teams? Is there anybody who wants to comment? We are about to complete our discussion. We are going to our last question, and you should ask it in Setswana

and English. Ok...one of the pillars of the national health workers plan of 2010/2020, is to follow appropriate procedures that guide the health system. But a shortage of health workers can hinder such an initiative. Do you remember any problematic situation in your work you find morallydifficult, if yes can you tell us about this situation, about what happened?

P6: Morally?

INT: mm...Like ethically difficult.

P6: mm, I will share with you, likethe incident that P4 talked about. We had this client; thispatient was reported…this guy was shot by a gun, so they came to the hospital. Luckily he was attended by another nurse who called me; I went there so that I can help her, when I arrived there was not even a single vehicle so we ended up calling the police and the hospital. It was very difficult because we did not get any vehicle since they were doing other things. Actually it was very difficult that I and my colleague ended up in a situation that we ended up deciding to use my car! Then I told her I am not sure about it because I called and I was told there e is no vehicle available. Imagine someone shot on the chest with a gun! After 30 minutes I received another call from the police that they are on their way and the vehicle that they are using is not in good condition, its lights do not work. They just wanted to take a chance, but the situation of the patient changed that was a patient who breathed spontaneously! All of sudden he had respiratory distress, and if it's like that hey! My colleague and I got the van, but what could we do? Because if it is like this we look at the patient's situation of what if the patients die? The first concern is that when someone comes and finds the patient dead in your car they won't…they consider that you took the first step of action to take the patient to the hospital, They will ask you why you had to carry the patient in your car while you see the situation she is in, even though it is a condition which you see that the patient needs immediate medical attention at the hospital, we also thought of what if my colleague's car were to break down on the way especially that it was at night, it was at night early in the morning, what if we had an accident? We started having a dilemma if we should do it! And the person who brought the patient started requesting us to at least use our own vehicle; the significant other was putting significant pressure on us. We ended taking a decision that was good and also not good for the patient; it was rather good for us. It was also not good for us because we were dealing with a human life, and we were in a rural area that did not even have management which did not even listen to our complaints and concerns. That is why at the beginning I said that I feel the government does not give priority to primary health care. I still stand by those words because really the way we are treated! Because right now if you could go to Departments such as BURS, you will never ever have a shortage of cars, but when it comes to health! Look at the size of Mahalapye sub district!There is a greater shortage more so that the hospital can have a shortage of vehicles to go transport a patient 80 km away from the hospital. That is why I feel that these situations are not at all alright. That's what I went through.

INT: It was a difficult situation..Did you manage to assist the patient?

P6; At the end we ended up using our own vehicle. Luckily it took 1 hour 30 minutes to meet the police along the way, so we transferred the patient to the police vehicle, because we felt that at least we were with other public service workers. In addition the police also assist with the transportation of patients, more so that we arrived at the hospital where the patient was attended to. Haa..You will never know.

INT: has anybody ever been in a similar situation? Yes P7..

P7 for us the problem that we encounter is the CHPC patients, the Doctor writes the dates that they are supposed to come for review aahh..They sometimes they do not get a chance to see the Doctor, which then means they have to re-book and at the end some of them end up dying before that.

INT: anybody to add on to that since we are coming to the end of our discussion?

P4:yes! On the particular day I was not there, but I was given a report that there was a pregnant woman who came….her BP was so high that we phoned Mahalapye that we have a patient in this condition hence we are requesting for transport that was not there. But then you will have to call other places like the library and schools. The school will tell you that they only have a minibus. As she was calling, the patient's high blood pressure got so high that she started getting fits episodes, yet there was only one nurse…ahh because there is no vehicle …. In our clinic, so she ended up using her own car to transport her to Shoshong clinic where they have a maternity ward. But when she got there, there was no transport in Mahalapye. They ended up requesting for a vehicle from a different department that just happened to be passing through, to at least transport the patient for them since she was having fits episodes, so they agreed on condition they the mahalapye people would return the patient. And you also know how small these twin cabs are, so the mattress was moving all over. However she did manage to take her there. But it is difficult because like she ended up using her own car, which is something not good for you!!! But if you were to have an accident no one would give you anything…because you will be driving at the same time you will be checking on the patient , so that you can record your observations. Since you cannot just records without observation. So you cannot drive and record at the same time while she is also having some fits episodes, you can end up causing an that accident. But if the patient dies, you will lose your job because everyone will have so many questions to ask, more so that you could have never agreed to do it.

INT: Yes! Is there anybody who wishes to add? They are difficult circumstances we understand……

P2: I did not hear your question. Were you saying as a patient or as a worker?

INT: irrespective of whether you are a worker or not! Having happened to you or having seen it happening to someone else.

P2:Mmhwell it’s just that my problem is that…maybe I can give an example from our area, there are patients that the nurse feel that they deserve to be transported, Butyet there are some patients whom I feel they are in the same conditions as patients that are being transported. The other thing is that they always say there are standards, so I do not know if these outline which patients is to be transported. So we end up arguing that really this patient deserves to be transported. The other one I can debate with the case that I have, I have a problem with my check-ups, so I see a government specialist in Gaborone. I have had this situation for 7 years and I have had 6 operations at different times. I am just asking to be moved up the line, because now I am told that I will have to write many letters as it is a long procedure, and I am still having the same condition that do not get better, that is very painful.

INT: Ok...have we said everything we wanted to say? We are very grateful. Sowe want to re-assure you thatwhatever was said in this room will remain in this room.
