# Supplementary material for: Stakeholders’ Perceptions on Shortage of Healthcare Workers in Primary Healthcare in Botswana: Focus Group Discussions
Source: PLoS One. 2015 Aug 18;10(8):e0135846. doi: 10.1371/journal.pone.0135846 (PMC4540466; doi:10.1371/journal.pone.0135846)
Supplement: S3 Text — (PDF) [file pone.0135846.s003.pdf]

HURAPRIM PROJECT

Participant ID: POLICY MAKERS MAHALAPYE
Date: 13/04/2012

Interviewer Name: Dr N

Interview Duration: 2.03.37 min

Audio File Name: Mahalapye

Done by

INTRODUCTION

INT: If it were possible re ka di tlhokomologa for a while but I know some of you can't ignore them and also there are
no wrong or right answers akere as I have said This is your own opinion, your own perceptions so it is it. No answer is wrong so is ok People should be open to give what is realy is their perceptions answers or what can be done and I don't know which other one you would want to for us to observe ok I think we can start the first one is I said it is adressing the Human resource for primary health care

First question What do we understand about primary health care. What is our understanding of primary health care? So that we may all understand what we talk about the same thing. Tota primary health care, go tewa eng? Anybody can break the ice

P 01: Is Blind care.

INT: Anybody else ,ee rra

P2 I will give a try Provision of basic health basic ah ah!! Measures...

INT: Any other addition. Rona re etlhaloganya e raya eng How do we understand it.What are its intention. P3

P3 Ok, The intention is to Help or to assist the

community before they reach the hospital or health clinics with basic level...<br><br>INT: Ee rra<br><br>P 04: I think is addressing the condemned health premises of the community.<br><br>P 04: Is also Making health care very accessible<br><br>INT: Eerra<br><br>P 05: I don't have anything.<br><br>INT: You do not have anything. Ok, lets………eerra<br><br>P05: yes but I think is primary health care is similar to first aid as is to help the person when the problem occurs accidentally.<br><br>INT: Ok, so we seem to have agreed gore that primary health care is near where the patient is before they get sick in the community and the basic before they go to Mahalapye district hospital. In your opinion are there enough health care workers on our own sight in Botswana. If not enough what is the course of shortage. Amme gone badiredi ba botsogo mo Botswana a re bona ba lekane ke raya ka tebo ya rona fela. Ga ele gore gab a lekane go ka tswa go bakiwa ke eng. Mxw!!Eemma<br><br>P01: nna ke tsaya gore bodiredi ja botsogo ke bona e kare boatlhaela<br><br>INT: gongwe mme fa le ka thatolosa lentswe nyana diotse di rekapa mantswe.<br><br>P01: bo Bodiredi jwa botsogo ja botsogo bodiredi bo a tlhaeala, hela o ka itlhela e le gore jaaka mo di cliniking bo nurse bababedi, ke gore nurse o mogwe o tlaabo ahapisa kenta yo a bona balwetse go bo go dira gore go nne bonya gore batho ba bone thuso ka bofefo.	

INT: Ok, She thinks believes there is shotage because the numbers usually there are few nurses, and it affects services as patients waits long long time to get services. Anybody else<br><br>P 02: is true there is a shortage of health personel across the board in the country at all levels this could be for many various reasons 1, we have not trained adequately, 2 were have not being able to recruit and retain, on the other hand health services have also improved a lot tremendously from what where we were in the 66 up to now although Botswana is a small population and but it is very is spread out country, 2 million people is not too many people if all of them are leaving in one city , in one city we can probably have two hospital for 2 million and acces health facilities .But now the way Botswana is Government have said every Motswana should be accessible to healh care, even if you look at very small settlement with maybe 100 people government is still is providing health care there but in order to do that you still need a number of people you can't just have one clinic with three people ,you need ten people a minimum of ten people . As much as we have lack of training and lack of recruitment is also the expansion on the service that causing that shortage.<br><br>INT: so ngaka o bona gore bothata ke gore ga re ba rutuntshe ka dipalo tse di lekaneng, gape le ha re barutuntshitse ga re bag are ba khipe mo servicing ba tsamaya gape Botsawna o phatlhaletse gora gore jaanong bodiredi bo tswhanetse gore bon ne bontsi gore bo kgone go fithelela bokhabare ba Botswana jo bo phatlhaletseng, gape le gore yone tirelo tsa botsogo di oketsegile thata esale jaaka nne re tsaya boipuso le botlhoki ja motho le jone bo bo agola. Ke dumela gore ke sobokile se ngaka nne ase bua. Ee Yo mongwe ene areng?<br><br>P 02 nyaa...mogongwe mogongwe mme kere ke Migration of the people to various places you	

will see that sometimes le tsenya bonurse ba le two somewhe, tomorrow those people go to town and elsewhere for green pastures, jaanong bo nurse bao ba nna ba sena ehh.. tiro e kalokalo, the movement of people sometimes are not steady that I think it also contribute to this factor.<br><br>INT: emma<br><br>P 03: talking talking about Retentiontion<br><br>INT: ee...<br><br>P 03: gore gongwe in-in in our chaleenging situation in our training may be we create also to try multiskill as much as possible, and also as we multiskil also taking into consideration our pay structure gore gare multiskiler then how can we also encourage in terms of remuneration gore a gone a appreciate this multiskill with multiresponsibility that is expected to perform at the same time having a course that helps to facilitating the skill that will allow him to function alenosi, bale babedi kana balebararo without needing any speciality on certain areas.<br><br>INT: so …..O dumela gore tota ha nne batho ba ere ha ba rutintshiwa boo ba fa fiwa botsipa, boitsaanape jo bontsistsi jo boanameng mme ere le fa batla go bereka go bo go supa gore motho yo o nale boitsanape jo bontsi boo ba bo duelelwa ke gore dituelo di supe gore motho yo o dira dio tsedi tse dintsi. Ee rre<br><br>P 04: eema I think the other problem is that ehh... there is no decentralization of employment like if I were to give an example, Mahalapye district cinic DHMT even if they have the shortage they can not employ for themselves they will depend on central government,or ministry to feed their vacancy which means now this people will depend wherever they want to post you may find that all the stationary hospital they might not sharing the same what we call manpower it might be the activities might be	

different and you may find the other people are there just only because is a referral hospital but not meaning that the workload is the same like the other hospital so yet this is another thing, if this people were given the powers to employing and retaining their staff it could be somehow different and again also the incentives or even the incentives are the same I just get the same salary like anyone in Gaborone, anyone who is in may be Mmaphashalala, or Xaqwa but life of the conditions of life are different even the way we live yet we get the same salary. So somebody will ask themself why should I go there yet I will still get a better salary in in a better area there should be somehow a better incentives for those in rural areas. Everything is same same everything same you go there no accommodation there nothing yet salary is the same.<br><br>INT: So P03, o bona gore bothata jo botona ke gore khiro e dirwa hela kwa ko ko headquarers so jaanong ha le tlaelelwa kwano mo Mahalapye le tshanetse go kwala dikwalo tse di ntsti le kwalela ko Headquatershe jaanong ka gore ba ko Headquaters ba tla boba lebeletse Botswana jotlhe ba hira Botswana botlhe le ha le tlhaelelwa.Mme botlhoki jwa dipatela tse ga bo tshwane le tsone ga di Tshwane bone mme ba lebelela hela gore sepatelasele le sele se tswhanetse go nna le baoki ba le palo e kana. Gape le gore ga gona dilo tse di rotloetsang batho ba ba nnang ko dikgaolong. Le ha oka ya o ko Xaqwa o nale dituelo tse di tshwanang le tsa motho yo o ko Mochudi mme botshelo bo sa Tshwane le dikgwetho tsa botshelo di harologana jaanong selo se dira gore motho are ke tlaabo ke elang koo ha ele gore nka ya ha Mochudi ka amogela go tshwana le o mongwe.<br><br>INT: Ee P05.<br><br>P 05: ee ba di tsere tsotlhe nne<br><br>INT: ba ditsere tsotlhe.	

P 05: kere ke are the other disadvantage ya train train gore we train and after training we lose them to advanced country’s take our professionalls

INT: yaa We heared maabane in Gaborone the DHMT in Gaborone bare in two months they lost twelve nurses. Eerra.

P 04: the other thing in relation to remuneration and other incentives I think it all starts from interest in enrolling into profession which starts at academic training I think there is no motivation in terms of growth when you want to enroll in a certain skill or profession and want to grow, you want to grow in both administrative and along profession (INT: profession), but in the current system growth is more in the administrative system when they is a pyramid like this like as P03 said kana we are employed from one point this is the only line of growth if you do not go to the ministry you not growing anymore professionally but we have to grow. So we should start parallel structures for you to grow kana health is science, if we want to grow along science line. Well so that if you are not interested to want to grow in the administrative this leaves a narrow path.

INT: So, tota rre ene obona gore bothata jo botona ke go re mogo rona gore o tswelele ha ole modiredi wa botsogo ke fela fa o ya ko minisrtry wa health o ya go nna administrator. Mme tota ha nne ba ka dira gore le fa ole ole mooki wa o belegisang o gole mo boitsaanapeng jwa gago, gore re tle re bone baoki hela bab belegisang baba godileng ba tona ba gola ba amogela mo professioning ya bone go supa boitsanape ja bone le experience ya bone ya dingwaga tse dintsi. Doc do you want to add something?

P 06: yaa to add on that one on concern about remuneration you know twoTwo years back I saw savingram there were from the ministry of

	health they have identified rural areas and they were saying all the people at work , working in the rural area they supposed to have some amount it was like P500 or P600 for fuel because all the administrative issue for them they are doing it in Gaborone even to renew the car everything but remember they have to implement that but oit was a motivation when this thing came the people from rural areas were happy that atleast now the government is thinking about for us to have something more than those staying in Gaborone but I don't know why they didn't implement it but it was decided when the saving gram came but never two years back until now, no one reached back it can motivate one in rural area if put in action because it was already decided the team decided saving gram came but never implemented.	
INT:	Rre	
P 07:	eemma kene kere mo gongwe gape godirwa ke gore gagona strategic deployment ya ya bo nurse I think its high time ya gore clinic di bulwe bosigo because you could find that most of the patients could have been attended during the night will have to wait or go straight to the hospital instead of being attended by primary hospital o bo o itlhela e kare ba shorta mo motshegareng mo di primary health care ka ntata ya gore batho ba nna bantsi ba ka bo attendlwe motshegare ke raya bosigo (INT: sso may be) gongwe ha ba ka theogela ka go shifta oka itlhela ele gore ba fokoditse palo ya ba tlang motshegare mo bosigong	
INT:	So may be that one should be all implemented by strategic deployment	
P 07:	Gore ba shifte hago nale ba ba theogelang bosigo go leka go hokotsa mosuke mo sepateleng bosigo se sengwe le o motshegareng kagore yo mongwe o tla lwala motshegare yo mongwe bosigo mme obo o itlhela e le gore okabo aile ha clinic e gaufi le	

	ene go nale gore o kabo aile crowda ko sepatela se se tona kagore yo mongwe oka bo a ile bosigo batho ba alwala bosigo ba bangwe ba itshoka bosigo a lwala ba be baya clinickingt phakela mme abo aitlhela e le gore go tletse ele gore bonnurse ba babedi fela.	
P 08:	gongwe The other issue eke ebonang is the saving of health professional gore at a tender age We are not aggressive in making awareness ya bana ha basantse ba le kwa dikolong gore ere ha ba ira career choices they realy know what is in the health system for them and as a country gore re tsamaya hakae gore ha ele gore re nale shortage of doctors maybe ya nna something like a lobbying system gore science related kids ba hiwe the demand gore e lebega e ntse jang inthat way we may get good numbers ya dio tse re ditlhokang for training ka gore sengwe sa dio tse di rebusetsang ko morago ke gore re depend mo gore go pasitse bana ba le kae ba batlang go nna dingaka but we do not realydo enough lobby gore re nale kgwetlho ya bongaka kgwetlho ya dipsychologists rena le kgwetlho ya 1, 2, 3 to support the health system so the other shotage is the result of our lobbying system we do not do enogh lobbying ya bana go appreciate booki.	
INT:	ee so re rotloetse bana ba rona goa gonna dinngaka baoki tsone diprofession tse ke tsaa gore ke sone se mme ntseng ase bua, OK may be we should move on many of this will keep coming. Now the next one, what we want to find out is do we think that there are issues or problems related to health care workers or primary health care or not? If there are problems yet what do you think are the important gaps or issues or problems related to health care workers or primary health care? I know Some of the issues we have already raised we talked of inadequqte training atleast, but we talked of numbers but could there be any other issues related to that are not specifically numbers, are there any other issues	

	ago nale dio tsedingwe di ditlhaelo kana diphatlha mo bodireding ja ja botsogo ja jone ja primary health I mean some of them we have already said gore gona le shortage te eleng gore maybe of specific cadres more than others but I think may be now at this point look at them.	
P01:	mghh Many of the issues have been raised; Primary health is basically first line help at the where the community is now to have the people there is also question of choice . like one recruitment, two deployment. Many people would not did not like to go to those areas there because of lack of other facilities so obviosly where they do not go lot people would be concerned in one area but not remote area about the same thing like remuneration , transport, education allsorts of other things . secondly you know what primary health care have is been administered by government of Botswana ok I don't think any private sector does provides primary health care here I don't know not in a big word but there are many people in private sector who are still providing services to Batswana but because of inflexibility we can't utilize them so we always say ther is shortage of doctors in the government but no shortage of doctors in private sector, for example you may find a place like mahalapye may be when we were there are five doctors in hospital we already have three in town ok Gaborone I think many doctors numerous doctors many spealists but the problem with flexibility problem simuilarly there many nurses in private sector many retired nurses there so if there was a flexibility to say ok if I wanted to do a subwork in that clinic and I will be paid for that I'll go there you are now rationalizing all your staff within that country and the private doctor, private hospital, private clinics will run themselves who will run this but now primary health care is being run by government. If there was a flexibility then we could be utilizing this people and say you go and do outreach at that village and they will do it gladly doing it for the	

money.<br><br>INT: ok so P1 ene o dumela gore ha nne goromente aka dira gore baba ikemetseng bale nosi nako nngwe ba dirisiwe go thusa mme ba neelwa dikatsotsa go tla go dira mo dikokelong kana mo diclinicking gone goka thusa. What do others say? Ba bangwe ba reng?<br><br>P02: mmhh the other thing In relation to what P1 was saying we are not amassing what we have if I may give an example I had a training trip to Singapore recently, and their health system is in such a way that even the retired people who still have enegy to work I get similar benefits not all my benefits If I feel I can still work for few hours unlike the system of government of Botswana when you are a local contractor officer lots of benefits are taken away which is not attractive after retiring if you feel like I do not have anyting to do but I can give one to two hours at work there is no system to allow you unless you come fully employed, so in that way but the skills are still there but not being utilized because we feel parted ways with you and then you do not availe anything to say for primary health care what can we do may be provide a system we can bring them back not necessarily full time to work just as a way of gointsha bodutu.<br><br>INT: eehh so Mme ene are nnyaa tota ke gore hela le rona system ya rona gae e ntse hela jaana ga e obege, gongwe ha ekabo ere mme e le mooki aretierile are nna nka nna ke tla go thusa laboraro le labone nka ta go bona balwetse fela fa le ka mphira, mme ebe gotwe nyaa gore re go fire hela ke fa ele gore re gotsaya 40 hrs a week jaaka mmereki mongwe le mongwe ebe ele gore mooki are nyaa nnna kana montage le labobedi nna ke tlaabo ke na le bongwaanangwaanaka jaanong go raya gore ga nkake ka tla. Jaanong are goraya gore re leke go obega.ehee mme le rre re tsamaye jaana.	

P03: ke tsaya gore mme o boleleela ruri gone go kanna botoka ka gore batho baba retirileng bonnurse ba bantsi mmegongwe ha nne go ka bulega gone moo eseng baba hira gone jaaka le bua lere motho anne gone hoo malatsi ale mararo kana ale mane a lebeletse balwetsi ke tsaya gore gone go ka nna botoka gona le gore gompieno kgang ya teng e ntse jaaka go ntse jaana kgang ele e eutlwisang botlhoko jaana.<br><br>P04: I think the other aspect the government can look into is how they provide primary health care as I have seen in other countries where by the you having a local GP here and a local pharmacy here and the government will reinvest them for the services they provide, it might be costly to the government but nna my view is that for you to employe someone you have to pay lot of things like terminal benefits, and other benefits but you just paying only a service providers as the government then you can calculate your physiccosts, I have to pay a pharmacfists this much, a GP this much then they do not have to wory about their remunerations no strike, nothing (the group laughing) I think the government should venture into that one.<br><br>INT: Ok, so rre hale ene one a re goromente gogongwe le ene o a ikimetsa. Gongwe le ene a akanye gore o ka bopa bodiredi jo sesha, gongwe a dira gore ee sesengwe sone re tla sala re sedira gore ha o bone ngaka ele re ka mo duela P40.00, fa ele gore o filwe dipilise tsa highblood goromente le ene a itse gore o tla duela bokae pharmacists kagore gongwe go hira batho ba bantsi go a tura ka gore ba batla ditlamelo tse dintsi, di tuelo tse ba di batlang didintsi thata. So I think that’s a suggestion. (Participant arriving) re a leamogela u are welcome. re kopa lo ikitsise (Participant; Ke bediwa Selai), ehee nne re setse re simolotse re lo amogetse, tota se re se buisanang fa ke gore why is there shortage of primary health care workers for primary health care le gore go ka irwang ka gone.ke eng gona le thaelo ya	

bodiredi,a gona le diphatha mo primary health care Ba rile primary health care ke bodiredija botsogo ja ntlha, the first basic health care needs in the community where are met. We use both lanmguages Setswana and English so those who know enlish may speak in ennglish ke tla tswelela ke ranolola ka gore go tswa far a go dikwalolola re di ranolela mo sekgoweng. Are tsweleleng rre o tla nna a ntse are tlatsa.<br><br>P05: ehh so I would like to agree with P1 and mme Mtlonda tht I could go and work point pf view that if our government could be more flexible if given the chance I could go and work one evening on another clinic in that way I would release the manpower shortage because work from 4:30 for nothing I can not work for nothing at the end of the day I will get paid. This is very acceptable it is done in other countries we are not just based in hospital. U can work one hour there two hour there but at the end of the day, it can work but this red tape thing is the one that is causing a lot of this shortage.<br><br>INT: ee rre ene are nyaa tota ha goromente aitse gore ke theogela 40 hrs mo Mahalapye ke bekeng phakela ka 7:30 ke tshaisa maitseboa ka 16:30 nna ke ekutlwa gore nka go theogela ko tliniking ele ya ext 3 ka 18:00 ke tshaisa ka 22:00 akere go raya gore ke extra ke bo ke duelelwa extra e e leng gore ke half ya go tswa ka half seven go fitlhela ka half four. The other issue does our nurses adequaterly trained when they are from IHS like the nurse I saw at Makapong as I seen recently in Maun? Is that young nurse adequaterly ready to serve that community, aba rutintshitswe go lekana gore ba dire tiro e re esolofetseng mo go bone. Ee P5<br><br>P 01: ee No training is adequate what is like any other training person is different from the actual practicals but training itself is adequate what is lacking might be an experience and again also work experience is different. Like myself I have 31 years experience but if you put me in clinic I will have a problem because I have never	

worked in a clinic, so really but we have all been basically trained the same I will take time to get used to even the same one either trained in UB or Gaborone IHS will take trime to get used to the community but as for the training itself they are adequately trained there is no doubt. What might be a little bit of difference is experience because clinic and hospital and health posts because they do not work the same even though they are all providing health care.<br><br>INT: eee…ee... So for the purposes of this I think it is important that we realize we talk about the whole health workers not just nurses when we discuss this all the people that we want in our clinic.<br><br>P 01: ok ee Sory because. No as for training there it is adequate there is no doubt about it.<br><br>P 02: maybe academic training could be there but she to go to a primary health care area where you are sole person, when you come from school you learn a lot of things under supervision and guidance the best teacher is experience, that's why a lot of graduate, graduate yess they have a degree but no experience that's why are placed under internship the sole reason for internship is so that they may lean. If you just graduated abound placed in a rural area without supervisor then I do not have a system to fall bak into it, and say what will happen tome what can I do, maybe we need to have a system when we have to say new graduate should pass through supervision of experienced ones and other experienced ones posted to rural areas. So there should be linkages because primary health care is dealing with everything with community in a clinic is not like in Marina where junior doctors and senior doctors if I have a problem with an eye quckly arena I quicklyshut it but in a rural area in the very remote area I have never seen it so I might give it a wrong medication or even worsen it, as I do not know if it is kokoma is it troma because red eye is not	

	the same. I think it is very important that apart from training we get a certain bit of exposure so that we get quality. 	
INT:	So se borre ba se buanag ke gore gone mme thtuntsho yone e lekane, bothathta ja teng ke gore fa ngwana a tswa mo a tswa go rutiwa ngwana a bo a romelwa gotwe tsamaya o e gobona bon kuku kwo pilikwe gosena yo mongwe o mothusang ebile ke mo supegela kwa o tla abo alatlhegile go botoka a kabo a bewa pela fa tlase ga o o morutang fa afetsa, ba bangwe ban ne ke ba bona ko maung nne ba re le ha ele rojaroja ga esatlho e bereka ka gore gagona yo o nnang fa stationing sa rojaroja. Jaanong Fa molwetse a le fa tlase gag ago ke gore o bone gore oirang wena kegone mo nneng are go nne le system ya gore ba ba gologolo ke bone ba ye kwa go senang batho ba bantsi. Eeeraa ka ke gone o tsenang 	
P 01:	ee nna nne kere ke botsa gore in our system does not have kind of an induction course, whereby you can assemble may be the new staff before before posted somewhere else as in that meeting everyone will know gore o postelwa kae and then the difference condition and environment abe ale gone hoo abe a itse gore o simolola a khanselwa a lot of counseling maikutlo a bone gore kana golo o yang teng go ntse janog ga go Tshwane tsa urban areaor town ke tse tsa rural area ke tse. So that ha ale kwa psychologically or mentally will be prepared, on his arrival le gore bae ba mofa the same thing the community leaders just maybe to sit down with her that kana mma dikhondishine ke tse you are welcome here re tsile go bereka le wena, tse o tlileng go berekela mo go tsone ke tse so that orientation ko tshimologong in that particular training go itlhela fa o goroga fela so that she is prepared mentally. And then again above that I know gonale banna ba modimo balaodi le dikhansel secretary who are experienced in this area. But ha ba tsena teng ba theogela hela mo cliniking	

	ke gore o setse ole boss, ke gore ga gona connection, but you are maybe new just as the doctor was saying here, but they are supposed to sit down with here and say kana mma etsho situation ya rona ke e fa if you want to adapt this is the way to go, e ke tse cultura ya rona e ntse jaana ga e Tshwane le yako ramotswa. . So that at the end of the day psychologically will be o setse arutegile. Keraya gore this are the things istead of just droping jaaka tirelo setshaba ene e direga e le gore bana balatlhelwa koo they are bored ga bana social life ga gona ko baitisang teng, ka e bile ba tla ba rutegile ba tshotse degrees something like that. Mme hela nna I feel gore they need to be configured may they can adjust nicely that's what I feel.	
INT:	ee, what others do say? eeraa	
P 02:	ehmma I do agree with honorable Councellor. That there is a problem But I'm thinking this should start ko skolong During training they can do their practical in rural areas. So they can be familier with rural experience while still undergoing training.	
P 03:	enngwe gape ke feleetse go bua, gape nna ke nale feel ya gore problem ya to forget people whether from the miniustry from region, from health quaters once you droped they forget you they will only need report everymonth from you. Especially from the ministry because the ministry is the one resposible and until hao boa gape o tsaya transfer	
INT:	Eemma	
P 01:	Gongwe go go tlatsa honorable a se buang ke gore our transfer policy di tshwantse di bereke eseka ya nna hela on paper, because in paper we are saying it is minimum three years but we see people serving bo more than six years in one place a sa sute , so re bona motho fa	

atsaya transfer o tswa koo a le agreesive because o akanya gore gongwe o latlheletswe and go afecta even the output ya gagwe<br><br>INT: I think our policy is wrong because it says only minimum three years kana basically means it can still be twenty years. But Ok Any other point ke raya gore batho ba kana mma ba re ga go a lekana gare ba thuse gole enough ka fa ba rutiwang ka teng before ba tsena gone ko ba yang teng. Ga gona orientation e lekaneng. Mme gongwe nte ke latlhele hela ka gore ke nna ke botsang dipotso, amme gonne orientation e ka thusa e lenosi ke buela mo kgang e nne P6 a e bua, I think orientation can help a lot a lot, especially attitude but experience yone you may have read a bok, when a child comes like this mme experience yone like, when you are in one off the clinic maybe in one of the clases when you in third year, maybe the other one might be doing the work and you will be looking. It's like when you are driving, nna when I'm not driving in a place that I do not know sometimes ke a timela next time when I drive to the same place. People should get experiences before they go to places where they will be on their own. Ok a are tsweleleng mme gone aha re le mo tirong do they actually do the work they have been trained when they get to this clinics, aba go dira tiro tsone tse ba di rutetsweng so that tiro ngwe le ngwe e ba edirang they are competent to do the work e ba e rutesweng or ba go tswelela ba dira tiro ngwelegngwele e basa erutelwang which they are incompetent the work that is beyond the skill that they acquired.<br><br>P 01: No, I say we can hear from them, AHH!!!!!!!!!AHHH!!!!!!!!!!(Laughinh by th group)<br><br>INT: (laghing, ee lona ga le itse).<br><br>P 02: The judgement should be from the community to	

come from the community.<br>Ehhhh!!!! Ehhhhhhhh!!!!!!!!!!!!(group laughs)<br><br>P 03: Nnyaa gone ke raya gore from the little experience bona ba a edira tiro they donot specialize ba general ke pharmacist, ke eng gape (the group answers ke ngaka) ke ngaka wa kenta wa consulta ha wa ko clinicking le leino o ka go raya are o ka lentsha (the group laughs) generally ke raya gore from an idependent point of view tota bone ba dira.<br><br>INT: ee rraa,<br><br>P 02: ee mma, ke tsaya gore le fa ke saitse tiro ee mme gonne ba e dira sentle fela, problem hela e tona ke e resatswang go e bua ke gore go gongwe o itlhela ba lebabedi fela mme ba imeditswe tiro mo cliniking e baling mo go yone.. E mpusetsa ko morago gore gonwe fan ne go nale dishift gfo ne go ka thusa thata ga dira gore diclinic di bulwe bosigo kgotsa nne go ka fokotsa mosuke wa motshegare. Ha a ya go tla phakela o fitlhela go na le batho bale 100 people that are there also mo mosong, tota ka tsamaiso fela go tshswantse ga lebogiwa modimo pele, go rapelwe pele ha eya gore bo one esetse a jele half ya nako ya tiro, mmme ene otshwanetse aya dijong tsa motshegare. Finally clinic e felelwa e tswalwa gone fa ba bangwe ba sa bona thuso ba be boa basa bona thuso mme hene e le gore b aka theogela le bosigo bane b aka thusa hela thata.<br><br>INT: Yes Sir,<br><br>P 01: To comment to this one they did everything Yes, during their first years at work they start well doing their job but with time they may change. Primary health care job description seems to be a challenge in affecting service delivery. Sometimes nurses who are trained in certain	

areas refuse to offer that service but expecting him or her to say he failed but no he saying is not part of their job description is the doctor. It has happened with one nurse lady after finding another policy that one is not in my job description if someone is leaving life because of another policy why would he have to do that and he can't help in the primary care because the primary care is an emergency you have to save without following the policy they can call you when they call you even when you are attending the other patient you see the patient is dying because there is no caddler there is no tender but he knows how to put but he refuses because he say no is not in my job description this job description in the primary care sometimes can not help to provide first care to the patient when somebody else know well trained but he refuse saying no other person can do that not me.<br><br>INT: It is stated that your job description sometimes are an issue that hinders service delivery.Yes mum<br><br>P 01: May to go back to the issues tse e le gore say the government has not streamlined but jobs are just standadised.Now because is not in the job description then she request for the next level. So it ends up causing issues of industrial relations. I think because we trained them for multi skilling we should appreciate them for that even though we hired them for something else. As the have their own professional description of what to do.<br><br>INT: Is there shortage of any profession than others and shortages in primary health.Ago nale bodiredi jo botlhaelang thata.<br><br>P 01: Midwives for example, midwives they are very short of midwives in primary health care going back to what I was saying, a very young nurse fresh from school posted to a area community wherenobody is there no one to look up to now she has to attend to maternity and you know	

	how maternity is as one of the millennium development goals is to reduce maternal dearth and for me if we do not have that skills we can't achieve that goal. So to me midwives is in short. the second one is the famiy health practitioner, if we have afamily health practitioner nurse whose a very,is above a general nurse this one are trained more this is like a sm gpi they more or less like a general practitioner more or there in the primary health care can help.	
INT:	And then should I continue, others lona la reng ha batho batla ko go lona ba complaina, bare go shorta eng go thaela eng thata	
P 01:	I think issue ya ya ya ya if we can improve on our saterlite nnetlanne set up where we have mother clinic taking care of others or help small ones, at a small scale, Having those small certerlite at the small scale. And improving those services of pharmacies, lab and try to have those have consultation for this nurses in small scale having those in saterlite so that if those nurses have to do consultation for this nurses will not have to travel long as far from mahalapye distances a tswa ko bo pilikwe, kobo kgenene ko teng ga sekgwa atleast o ka helela ha bo otse if you have to say gore a thusiwe gore a saportiwe botoka,if we have such to get service ka gore re ka senne le pharmacist everywhere gape e tlaabo enna under utilization if we do that.	
P 02:	just adding almost adding to what she was saying u see as much as we want to multi skill a certain individual there always imitation on how much they gona do so to inhence that you also need to engage recruit other proffessionals and support them because kana health is an intergrated science and intergrated means multi skills together as much as we want those person to have multi skills we need to also have specific support for individual professional as well and therefore in that regards I foresee a	

shortage in are ya pharmacy bo lab jaana.<br><br>P 03: Nna ne ke botsa gore ke botse.<br><br>INT: Eera botsa<br><br>P 01: Gone ga go nke go privatisiwa<br><br>INT: Go privatersiwa<br>P01: prioteriterisiwa<br><br>INT: Ok Prioteriesa<br>P01: gore mid mid what (the group midwives) Midwives gore ere hela go dirwa staffing whatever ,ke raya training ga re battle bana gore ha ba hirwa ke nngwe ya dilo tse eleng gore maybe re tshwanetse re simolole re traine more of this o bona gore ke raya jana ha le gore generaly bo nurse re batla bale 10 and then the demand high and low, supply and the demand bo o fithela ele gore tota boammaruri we need every ten with general nurses they must be atleast ba trainer bale 5 ba midwife instead of 1ke raya gore can't we look at the situation and current and see that no this is the priority we just need to do it go na le gore jaanong bo re re gongwe re nna le bo general nurse baba ntsi bo re shorta re na le pharmacists yo one instead of 4 why cant we balance this things and re dira hela sentle ko tshimologong.<br><br>P 01: ahee rra I will try to answer you rre M, you see the problem is the one who is training is still an employer at the ministry of health,so you if maybe the training was done, I will use you as an example you are my friend there is no problem if maybe Moatse was the one who was producing the lessons then in that it could work but is the ministry of health who is also emplying and also training,I will give you an example they might say that we might say that we need about let say 20 midwives and the ministry will say I can only train five you see so it means now your demand it depends where from the employer the other thing is there is no	

incentives midwive and a general nurse they still get the same salary and yet the work is different so this really a complex situation maybe this things discussing it may help us and again since the politician are here it may also help because if really the schools were running independently from the ministry of health and then people will look for employment the ministry is not loyal and decides will train so many midwives in a year you see there no how we can say now since we are short of 200 midwives Mr Moatse school train 200 midwives is only the ministry that will decides we willget so many midwives irrespective of the demand irrespective of the shortage is the one that will decides but.<br>P02: Let me get this properly akere gona le there are always short term strategy where by all the supritendent tsa dipatela administrive people ba e leng gore they sit down and discuss it is either they stood up with the council secretary or the permanent secretary and whoever yoo ko godimo gone ke a itse gore DPSM on the other side ke the employer but ministry key one e leng gore they sit down and plan so during the planning can't you sit down and make sure that mare gone ke a thaloganya se ose buang and sit down and tell them our priority are 123.<br><br>INT: OK others because we runnin out of time…<br><br>P01: I'm not necessarily answering that one that maybe would help us because what P2 is saying I understand it but saying as much as the government owns all this institutions is the one which is also understraining from drain drain so if you are talking about draining more midwives you are talking about the same training that the government is not able to retain it's a visious cycle<br><br>P02: Whats the solution then<br><br>INT: Ok coming to the solutions I'm going to expect solutions from this house,so lets keep going about the problems the next part is the solutions	

	and so now the last thing with problems is their adequate support for the jobs to do the jobs for the heath care workers interms of resources,interms of coordination,interms of management do we in our opinion do we believe that they are adequetly supported todo the job.	
P(ALL)	(Laughing)	
P 01:	Yes and No yes to ok for yes at certain areas yes yes certain no aah you se the government tries to I'm ok employe people train people I'm posts them by drugs there is also an issue of coordination sometimes you may find that it is beyond maybe beyond the scope of institution or the ministry for example I will give an example of electricity maybe or telephone you may have a clinic ministry of health is not really looking after the like electricity telephone or the water so if this resources are not run in the clinic you may have the best nurse all the drugs and find a fridge have not worked, that kind of coordination intercoordination is very important yes we have our own deficiency drugs run out so maybe procument distribution also sometimes they not adequate I will say also yes and no on the other hand they buldind a fantastic institution with the latest state of the art you know your facilities with support system you know machines and on the other hand the maintanace issueis not there a support systems infiles transport maybe right now there is a huge problem of transport which is beyond ministry of health and by the way the ministry of health is the provider of primary health care when you are talking about primary health the ministry of health is the custodian of primary health care in this country ok but now for for a vast sub district like mahalapye we got we start somewhere like upto to martin's drift from there dibete up to rasesa no no radisele ,radisele is part of ,up to pilikwe ,pilikwe is within so that vast area you can have clinic there a nurse is there aa drugs are there but there is no transport for you to transport the patient.	

INT: so erra,<br><br>P 01: No I was just asking to say what P3 was saying as there is lot's of complex in this issues as clearly stated like good infrastructure, good personel but there and there you may find that here are lot something present which is hampering the job there.<br><br>INT: Tota mme potso ene ereke ray aka gore ha gongwe inka tswa ke seile ba bangwe ko morago ga ke sure gore a mme bone badiredi ba a barotoediwa a e bile bana le ditsompelo tse di ba kgontshang gore ba dire tiro e ka tswa ele tsone tse b aka tswang ba di buile dipilisi kana le dilo tse dingwe tse ba berekela teng le gone gore bogogi le boeteledi pele bo siame gore ba kgone go dira tiro ga ke itse gore Setswana se siame.<br><br>P 01: Ee mme se siame<br><br>INT: Mme ke utlwe mme<br><br>P 01: Ne kere gone jaaka mo go setseng go buiwa jaaka bo dipilisi bo eng tota ha re berekelang teng ko madiba clinic gone go worse o tla fithela e le gore ha go kentelwang teng ga goyo koore otla fithela ele gore ha go bandagetswang balwetse le gone go seyo ha go tseelwa dipilise teng le gone go go nyenyane mo e le gore bongaka jo bo hoo tota go a shorta mme clinic yone e ya madiba ena le overload ya babelegise b aba ntsi mo eleng gongwe nkabo madiba a atolositswe go se kahe nyana gore gongwe batho ba kgone go thusiwa dipilisi o kgona go fithela gongwe tsone tsa sukiri tsa high blood bo eng dipilisi hela tsotlhe ka modiro wa tsone o tla fithela e le gore ga diyo go tla bo gotwe ya go tsaya ko baitiredi ga o yak o baitiredi ga di yo o tla boo feta key ole o ya airstrip ha o tswa airstrip ha gongwe o ka di fithela tse dingwe o fithela di seyo so bo gotwe sepatela gone golo mo o fithela ele gore jaanong golo mo o kare bolwetse botla gakala ka gore balwetse ba bangwe b aba itsapang	

jaaka bone b aba senang sepe se a ka palamang ga bedi gongwe a ya go tsena ko spatlela o hella a itsapile ka gore go sale gotwe a ye go tsaya dipilisi se a se buang ke sone se a reng e sale gotwe ke ye ga tsaya dipilisi ga gona se ke palamang ka sone.<br><br>INT: Gona e rre keyo.<br><br>P01: eemma nna ke bona gona le mathata gone fa, kagore tota from the council point of view ka ko councileng we experiensitse mathata a matona especially gone ko dikgaolong kana ne re tshwanetse gore re maitantane di infrastructure tsa teng ko ore like for instance bo motlakase re reng transport jalo jalo le gone hela ga ke itse gore gone gompieno ka gore re ko ministry rotlhe le ya go kgona jang ka gore nako ya teng re ne re straggler mme ne re itse gore gona le parkage nngwe e tisiwa e tswa ko local government e sa tswe ko ministry of health e le gore re ne re supplimenter ministry of health ka gore ko local government gona le budget e lebaganeng le mo, re ka reka koloi ya clinic re ka reka mo re ka dira sele re ka dira sele jaanong since sengwe le sengwe se ile ka ko go lona di constrain tse ne re na le tsone nako ya rona I'm trying to imagine how are you going to manage those constrains but for sure gone ko dikgaolong is very painful o fithela nurse a ntse fela jaana go sena sepe sepe jaanong ke raya hela ha re akanya ka di solutions ha gone we are only talking about problems.<br><br>INT: Ee mma<br><br>P02: ke batla go tlatsa mokhanselara, kana gone ha gone go ntse go nale koloi ya madiba airstrip ward gone jaana jaaka ke bua le wena jaana balwetse ba rona ba tsewa ke koloi e leng gore e tswa tewane, akanaya hela ha ele gore motho o na le bothata jo bo tseneletseng, ha bothata jone jo bo tdeneletseng ha ele go gongwe mokhanselara o gaufi ke ene a etle a thusi jaanong ha mokhanselara seyo gona le molwetse yoo tseneletseng go bidiwa koloi ko	

draught gone mo ke bothata.<br>INT: Ok, so we were talking about resources transport is a big one and also drugs for primary health drugs is a big problem hypertensions,diabetic drugs are often not there were have to one place to another some them with no resources at all.Ok now we will do the last version with problems then we take a short break there is tea this side but we can take the tea stop for five minutes then we can finish the rest of the tea here and the other problem is there a problem of heath care workers in rural areas not just in primary health care in rural is there a problem in rural areas if there is a problem why is it so,Akere we talked about general,we talked about primary health care now we talk about rural areas study have shown that is worse in rural the situation of health care workers,in your own opinion is that true that there is a problem why is there a problem why is it worse in rural areas ke eng bothata jo ja bodiredi bo bo ntsi thata ko dikgaolong tsa magae tse di ko kgakala ha e le gore go ntse jalo go ka tswa go bakiwa ke eng?Ee…<br>P 01: I think jaanong e ka re busetsa ko go e ne ng e buiwa ke rre ha ya tsone di job description o fithela e le gore gone koo ke nurse hela ene o ka kgona go dira dilo tsa go nna jalo mme bo go twe go emelwe ngaka,ngaka e ta ata ka labone ene gole Monday wena o emele ngaka go tla ko cliniking ka labone ke tsaya gore tsone dipolicy tsone tse jaaka rre a sa tswa go bua ke tsone tse di dirang mathata a matona mogo maswe tsone di job description tsone tse gore ka dira se o ka seke a dire se mme motho tiro a kgona go se dira a bo a ya go emela ngaka e yang go tla ka Thursday and go end up motho a thokafetse mme nurse a ne a ka kgona go thusa motho.<br>INT: What you are saying is you think the big issue is our job description ke yone e restricting it restricts can you just say it?<br>P 01: ok what I was saying is that the job description for the nurses it is realy restricting them to save lives and manage to help the patient and too	

much to wait for the doctor who is coming on Thursday and is Monday you are told to wait for the doctor who is coming on Thursday I think this policy should be changed I think nurses should never be givin some job description to do they must do everything that she can rather than waiting for someone who is coming from far<br><br>INT: Ee Rre<br><br>P 02: Ok another issue in the rural area is that you will find that there once he get to that there is no one who is replacing him and it takes time to bring another nurse people come as far as the cattle post they don't find anybody I think that also lead to the problem.<br><br>INT: But why rural areas, why is this particularly a problem in rural areas<br><br>P 01: aahh, particularly in rural aareas because, One number of staff is not adequate,secondly the support that we give them to facilitate to the referrals kana if the nurse is to refer to a doctor the doctor should be accessible but we don't have enough doctors but still we don't even have transport because we don't have no and you need doctor I'm reffering you and I will give you transport then generally in those rural village you will find that maybe there are five vehicles privately five vehicles there is no policy to say if you are over whelm you use private people then we pay them just to assist us to ferry the patient so to get transport to come to mahalapye from makgenene you need to have wait by the road for three hours hoping to get a vehicle passing by so the the the there are a lot of issues.<br>INT: Now how about because we are talking about adequate number of health care workers what about rural areas that actually get more difficult to get adequate number of health care workers,ke eng ha e le gore bodiredi bo ka tlaela thata ko dikgaolong tse di kgakala go gaisa mo dikgaolong tse dingwe can I call you	

back to it<br><br>P01: The reason being the planning system kana goromente plans to say how many people are used immediately and how many I 'm going to provide so ha gonwe etla bo ele gore the population can only provide two nurses because if I provide more than that in a way koore the rural area are very difficult in the sense that to provide more is a wastage ba go nna bas a dire sepe because people are not many to provide nurse it's a challenge because that leess ha e nna le dikgwetho you have nothing and to be able to to to to relief like honourable councilor is saying is not very easy because each and everyone of those have enough for herself ga kere so that ha ele gore ba babedi bao ga bay o ha ke tsaya yo mongwe key a go mo paralyser one way or the other.<br><br>INT: Is there problem retaining health care workers in rural areas than it is in other areas and if so why?<br><br>P 01: Gape le gone nna ke tsaya gore for instace hela mo Botswana have shortage of doctors mme gona saterlite e neng e dirilwe and clusters tsa teng e leng gore it was meant to provide doctors to cover certain areas jaanong gongwe accommodation le yone hela ke rela gore alone mo gonna ke fila gore le yone is a big problem let alone we don't have a nurse there or a doctors there, he needs a good accommodation e leng gore le ene o ka nna sentle but if the accommodation is not there even clinic e sena all the resources tse di thokahalang who is going to be recruited to stay there.<br>INT: so re bua ka bothata ja go nna ko dikgaolong tsa ma gae,gore go bothata gore baoki le badiri ngaka le babangwe badiredi ba botsogo baye teng gongwe jaaka mahalapye<br><br>P 02: let say mahalapye is not a rural area,a rural areas means area like bo otse and you ask if its retention so I don't work there but I can foresee the problem whereby people have kids have to	

go to a certain schools that will be difficult to keep the person there.

INT: Ee ba bangwe ba reng?

P 01; I think the other factor ke yone ya life opportunities, in rural areas there are no life opportunities some people like to do life investments like some other people ave suggested gore ban aba kaya ko dikolong yo mongwe o ka reka ploto any other life opportunities that are there I think are thethings that makes people not to go to the rural areas.

P 02: Ee mma the other thing is that I think people who are who are handling atleast if they could be somehow renumerated more or atleast a little bit of allowance than those people who are not in the rural areas this are the factors that there is this rural,there should have something better than those who are in other better places, maybe if they were given maybe cars or even housing allowance or something that will really push somebody to go there a little bit of more incerntives because somebody will ask why should I go there when I'm going to get the same salary like somebody who is in Gaboborone and even the roads are so bad

INT: Why are your expenses more?

P 02: mma

INT: Why are your expenses more?

P02: the expenses are more because to get maybe to have to come to mahalapye to buy phaletshe you have to come to mahalapye,grocery still like that and you knows if you are driving you will drive if maybe you are you don't have a car you have to ask forlift and pay that person yet somebody who is in mahalapye will pay three pula for taxi to do grocery so this things if really there are concert and thought in a constractive way they will attract people.

P 03: I do agree but to add to this one you need to make a clear policy of transfers because if you send someone to a rural area he has to know and be prepared that I'm going I'm not gone then to be there and dye rural because there people who are city people and rural people if there is clear policy of transfer people will be coustious prepared and that I'm here to provide service but in such time two years not atleast ending and dye there we are all human beings everyone need to move to replace those in the rural to live in the city they can't say no they just choosen by god for them they are city people those one are there no if there is a clear policy a clear and conscious people will know I'm here to provide the service handling my time I will move to somewhere if there is a clear policy of transfer make people tto give themselves which is time after this time I'm allowed to move also and another person from the city can also come if it is clear it can also help people from rural areas to work with conscious and to be not arthitected or to refuse to go there if theygo there is for good but to go there I will come back or I will move.<br><br>P 04. The other one is families we have challenges demploying people because I want to be near my family maybe I'm the mother I need to be where my kids are because as a mother I feel I can take better care of them where I I'm unlike like other where saying I need them to go to school,the school that I feel will give them good future not just any school so you will find that tota in rural areas this services,even the support services that should be supporting the health workers like education for their kids is neglected is not really taken care of in such that they can stil feel that I can still go there and my kid will go for a proper school or even the school that will provide them atleast to really have to learn so that they can also have a good future so we struggle deploying them there,even we do force them because altimately ga kere we saying experience of service and then if they can not	

	find any other employment somebody will be oblige to go non the less but hela the amenities that are there dont be good enough to support their lives and I have to buy se mr sebego a se buang gore if there we cannot be able to provide everything for them atleast lets give them a package that will compliment the losses that they incure interms of social life is so distructed	
P 05	May its true all the things that has been said its true let me look at it in a different angle as well,you there is a shortage of staff in the rural areas the fact being that if you look at a clinic in the rural area and a clinic in the urban area the staffing number will be the same ok now if we assemble a clinic next to princess marina is staff with say two nurses and then you have a clinic far away in gantsi staffed with two nurses now apart from the money aspect of it the economics guys will say productivity output taxes economically doesn’t make sense but you know in Botswana,they say every Motswana should be provided with health care ok nomatter where you are,now that posses a challenge you can have a settlement with 250 people or 500 people we have to have the same kind of because your standard will be lower right those people will have the same immunization the same drugs same programme everything as a city challenge ok all those programme now in the city we got an alternative can close and then the alternative we will go to a hospital or private sector continue now in the rural area what happens is that you don’t have any other hospital next to maybe a 100 km or 200km hospital or clinic there with two nurses you cant get more people there because of reasons and the other reasons as mma matho said why should I send four nurses who would do almost nothing because they will see almost five patient per day it is possible as it you are saying the country is short of nurses now can I send for 4 nurses to a clinic that see 10 patient yes if you don’t send those four nurses there those 10 patient may suffer because after to other clinic	

24 hours you need a minimum of 6 nurses if you have shift even if you don't have shift because you cant say one can work 24 hours shifting not on call something must be open there 24 hours somebody cannot work for 24 hours 24/7 so we need to have maybe 2 nurses in the morning 1 in the afternoon 1 at night 1 off sick 1 has gone for so continuously so you see to have those nurses 6 nurses in need a lot of packages a lot of things but you need to provide the services whereas in the city you can have those two nurses running closes at 4:30 no problem the hospital is next door you see<br><br>INT: Ok any other thing, otherwise we will take our break to have tea and we will be back to come with solutions.<br><br>Break!!!! Break!!!!!!!!!Break!!!!!!!!!!!!!<br><br>INT: Ehee..jaanong..Thank you for coming back,so this next potion is realy coming up with solutions,so the first one ke gore what should be done ..what do you think should be done about the lack of health care workers for primary health care in Botswana? I know some of them we are alluded to but this one is even..,ok now that we are aware of all this problems how can they be solved?what can be done to improve human the capital ya teng, the number of health care workers..tota ka ha thoko ka hana bodiredi mo di…mo..mo..primary health.Ee mma.<br><br>P:Nna I think the first think that we should do is…is to have standard that inform our manpower planning.<br><br>INT: Ok, what do you mean by standard?<br><br>P: Standards mean number of..the facility..per level of facility,gore if we say we have men in this facility properly what kind of manpower should we have at ..at all levels,then it will inform our manpower planning then our timing plans.<br><br>INT: Have we send the intergrated health sciences	

plan,is that..is that what we are talking about or is it something more?<br><br>P: This is ..this.. just come out I think also they are not aware of that,maybe you can give us a brief that that's what Botswana has come up with.<br><br>INT; Ok...<br><br>P; Five levels of care<br><br>INT: Five level of care,so they have come up with. At this level this is what we expect in this level,it was suppose to be implemented from 2010,it suppose to be from 2010 to 2020..its beautifully written and I think a lot of work gone in to that.<br><br>P: If that is ..if that is there then maybe we should start looking on implementation.<br><br>INT: For Standards?<br><br>P:Ee mma<br><br>INT: What else can be done?re boile ka mathata a mantsintsi..Ee rra ,ale tsholeditse?<br><br>P:Nnya mma ne ke sa tsholetsa ,mme le gale ka kene ke biditswe ke tla bua.Nna tota I think what should be done I that this people who are in rural areas ,it should be a way of giving some incentiments,some incentiments of some sorts that atleast they will attract them to that a..that area.<br><br>INT: What source of incentives? ke raya gore ga ele gore..<br><br>P: Maybe they can even given free accommodation,and then some maybe even 5% of their salary allowances for staying that side.<br><br>INT: Ok...so re akanya gore e..ne re bua gore gate ba ka dirang go tokafatsa jaanong na bua gore nnya gongwe tota re..re ira gore go..go nne le kgogedi ya gore batho ba berekele kwa,gongwe re kare ba fiwe boroko for free,so free accommodation,are we saying government	

should constracting houses because for instant batho ba ba tswang koo..the people who..who..when Ngamiland I think the place that attracted me more than the Mahalapye district because probably the ..the levels are very different,they said even in a village there is no house to rent so that is ..that is the problem.<br><br>P: mm,so for.. for those villages where there is no house to rent government should be strategizing those villages and building accommodation.<br><br>INT: So accommodation? And you should be free if you are rural?<br><br>P-all: Free..yaa..mmm<br><br>P: Somebody subsidise because even accommodation because you can..local empowerment is ok people can build houses ,rent it out to the government,government pays them but people who stays there should be free.<br><br>P:There should be free accommodation.<br><br>INT:Ok..anything else,what do others say? So we call that incentives,so this will be part of retention strategies?Ee rra..<br><br>P: I think issues surrounding the job descriptions should be clarified because it seems to be the issue in the rural areas.<br><br>INT:Ok..What about..how do you get the right skill mixes anyway in this places,I mean we talked a lot about the nurses doing this and I know we even…or to maybe nurse can be a.. skilled so that they can do a lot but then there was also another deal no have all the people and will provide,so how do we actually sort out this issue because as..there is a reality,for instant I went to Makalamabedi I think they have 6 nurses or 5 nurses or 4 nurses I don't know,and …and they say to be honest there is…there are not a lot of patients but because we are 24hrs clinic we need all the 6 of us but when got there after we charted with them they have been sitting there just waiting for people to come over but..so how do we get the right skill mixed but still we ??	

P: (clears throat) I...I think they..the way it should work like the cluster system,this cluster..a cluster should be like a mini mini hospital or hospital of smaller smaller level.That cluster should..thats a mother clinic where we like P4 was saying,maybe a pharmacy technician should be there,a lab,a midwife everyboby covering a certain area,I mean they need close accessibility there,so even if you can obviously is not possible to get midwife probably in all the clinics,is impossible..is not possible because of the I mean ..the farness of the area but if we can really mend this areas well they could have outreaches, this people could…even before going to the hospital they could be able to come here and get certain level of care,stabilized care and before they can be transferred there so this clusters need to be really beefed up with midwives,FNPs,doctors,pharmacy the lab everybody should be there even social worker .the counselor all those.<br><br>INT: How about...you know Im talking about Maun,it dosent apply to you because your cluster seems to be closer,but for instant when we were in Maun Cavin was like ..there was a young woman who works in Makakung I have talked about her because she tract me,I don't know how many kilometers from Kareng is it,100 I think so there is no transport she is alone she is ..she is a general nurse and she must be in her 20s so I don't think she has been there maybe 3 years and so she had to go on horse back, she had to get a ride from a horse to go to Kareng where there is midwife to get to eventually get into the ambulance so Kareng there is bigger clinic but is 100 kilometres and there is no transport between the 2 of them so how do we deal with those kind of situation because Botswana has to say ?<br><br>P: The other issue is looking into transport, for...if...if I may have to site Mahalapye area we also have sandyveld but the types of vehicle that we have there are not going to manuvouor the areas,so when its time to provide that transport which is not there currently we should also look for transport that manuvoure the..the… the terrains that we are having around the country and even the way our ambulance will be build should address the terrain issue ya lehatse.<br><br>INT: Other participants talked about transport for	

patients and for themselves,like you are saying that when you are posted to areas like that and you want to move from there maybe to go and see your family, what ..what should be done to address those concerns?(Mma S)<br><br>P: The issue of allowances has been…is...Is we have sited it, we cant leave it so that even I...I know maybe the vehicle will come once a week but atleat re tla ema ka compensation that …that is saying to me we understand your situation although we cannot help forthweek but have this so that once you have a vehicle atleast you can be able..without depleting your.. your salary when you arrive home you are almost..almost spending on your salary on.. on trying to check the family,now you are no longer suppoting the family you are just working far away.<br><br>INT: So,how..how the level of this because I understand that the very remote…it is still there it it P400 a month but somebody was telling us that he works in Stragledum and he comes from mxm..gate ke kae? From the North East going home he used to spend P5000,as to Come from Stragledum to to..what did she said..anyway to go to the North East to do..so if 2 relatives dies in a month he (laughs)<br><br>P :Its like you are in America<br><br>P-ALL:mmmm<br><br>P:Long trip(laughs)<br><br>INT: Anywhere ,so..so we talked about about skill mixing that maybe we should beef up and strengthen this clustering and then there will be pecularity,like there will be clusters that can easily cover 200,500 kilometres so that need to be handle separately.<br><br>P:Separately<br><br>INT: Exatly..and then how about task shifting?task shifting is the concept that is now being thrown around in the world of a..human resources basically its..looking at the tasks that needs to be done and then decide gore no this task there is no need for this task to be done by somebody who needs to be trained for 5 years,can this	

task be done by somebody that we can traine for 3 months,and this task that is been done by somebody whose been trained for 7 years maybe it could be done by somebody whose been trained for this amount of time with the proper training and of course with proper recognition and compensation.Is it something that you think maybe could improve the situation in health.. in primary health ?<br><br>P-All: .Yes.<br><br>INT: Ee rra...<br><br>P: I think it can work provided …provided that the...the platform is condusive for that,such as policies,job discriptions because you will train me but you wont change how you hired me,that is our main problem.<br><br>INT: So they should be... government should be supportive to...<br><br>P: Alignment from the policies and the ..the regulations that we are working on.<br><br>P: As those are being acquired as they will enable them to use them?<br><br>INT: Ok...Ee jaanong the other thing, in your experience what has already been tried? what strategies have already been tried to address this problem so that we are not going to do the thing that have already failed.What is already being done to try and address this, go setse go dirilwe eng ke raya gore ke maano afe a kileng a lekwa gore re leke go rarabolla bothata jo? Rre wa VDC what do you think?<br><br>P: Nna ke tsaya gore go setse go...go dirilwe mathata already(All-laughs)<br><br>INT: Mathata, eseng solution?<br><br>P: He is alright, this councilor...this hounarable is right.<br><br>P: Ke raya how do you...how do you take health facilities	

tsotlhe o bo o diisa...o di isa o di centraliser? We are talking of decentralize so how do you go back to centralize, that's my problem.<br><br>INT: So that doesnt work?<br><br>P1: I don't know..i don't know I have to look at it but ke raya gone ..Im not sure wethear it will work but I think it was much easier with that decentralization e neng e ntse e exista before where by we have our own clinics and our own nurses and we were operating from Local government and then ba health ba operator from Ministry of Health and then coordinate it together so go raya gore, I think that colision we were closer to the people, much much closer because ne re kgona go nna le ...We were able to have our own budget from local government and then??? for transport and other staff but today it is very very difficult because le rona tota hela mo khanseleng ga re leba we pass by the clinic,mokhanselara ga a feta they just cries.<br><br>INT: Do we...do we think that this is just a transitional a ?? or that probably ..That its looks like is gonna be a big problem fo along time.<br><br>P:Gone the idea is good a kere you want it,ne le batla all health problems same ministry but the implementation ya yone hela is not..is very difficult.The idea is good.<br><br>P:(clears throat) I think..i think<br><br>INT: Ee mma..<br><br>P:Nna I think implementation the idea is good but maybe there was no planning for implementation so and having no plans for implementation is the one which is leaving us with no guidelines gore we do this then what then what and who is doing what. Now we are running to the second year without structures tsa di DMHT which has really throwing us all over we don't know what we are responsible for we are trying this and that so I think the idea is good but implementation was not planed for properly<br><br>P: to add more on that,original consultation I don't think it was adequate,it was not what they should have done ke	

gore they should have gone through all the councillos mo eleng gore they will address the issue to the political people together with the …the community so that was not properly done that's where the problem is because ne go tla bona hela go ntse gote diclinic di a mover unlike water utilities there was thourouly consultation so it was difficult but it was slowly going there because there was consultation consultation after consultation but before we do it.Now this one we just implemented and then position ya teng is very difficult because like she is saying le rona tota we don't know what is expected of us,the waythey say things….. boa koo like they don't belong to us,<br><br>INT: Ne re bua ka..golo fa re bua ka go ntsha ditirelo tsa botsogo ko local government go di isa ko goromente wa..wa..gore a gone ga..ga.. go lebega go tla re thusa ne.(gone ga tokafatsa? Mma S) go tokafatsa seemo sa bodiredi.<br><br>P: Maybe what we are saying ke gore tokafalo ya teng ga e ise e bonale sentle, because we have teething problems tse re seng sure gore ko bofelong ga di tswa maybe di sena go addreesiwa jaaka re bua ka bo transport wa bona, we had a challenge ya gore kana di standard tsa local government le tsa central government there are very different ko central government ga o dirisitse koloi 5 years e nna.. e boaboela ko CTO we put it in the yard,go raya gore Its unroadworthy,ko council bare hey budget ya rona e nnyennyane nte re nne re e iteyeteye ka sepannere e..e.. e re thusethuse until they are able to do something so re di tsere dile in a state sa gore di setse dile bo over 5 years, over 6years almost 10 years tse dingwe and ka di standard sa central government there are not roadworthy so di mo di yard but we don't have fund tse dire facilitaitelang gore re reke tse di di repleisang so as we speak gone the health system is in a very serious challenge in terms of facilitation ka ntlha ya gore to facilitate easier is to have a vehicle around so ga di facility dile 42 le di operaita ka only 11 to 17 vihicle is streaching,so what we are saying ke gore the idea is good but gongwe we didn't plan well so that go nne smooth transition knowing exactly what we have, and what we don't have,what are we going to	

do and the budget will provide it gore kana we are going through that transition we should have a budget e ka addresang issues tse eleng gore we come across.<br><br>P:Mhh..mme gape gone foo,ga kere ke batla gole tsenella.ga kere maybe we must..re adopte motto wa local government o o reng batho pele,because in central government is not batho pele,NO rona mo local government ga re re batho pele,ke gore like she is saying koloi ga e kake ya..yare e sena leotwana gona le le lengwe le le ka phechiwang to go and assist somewhere else ra seka ra diega we have to force him gore monna bona gore o tswa jang gore re ye go tsaya molwetse ole. Motho pele,because rona ga re le makhanselara we adhere to and we don't want to see that happening because otherwise if that is failing it means mokhanselara has to take his own vehicle a ba a ya go tsaya molwetse yole but at the same time we feel gore tota ga nne go kgonagala even the ..the ba eleng gore they have vihicles because we are running short of vihicles ke bone ba eleng they can use their vihicles then you can claim o bona gore ke raya jang. So that at the end of the day o tla fitlhela ele gore nnese o fa,mokhanselara o fa in your case,but in our case a.. le ga re sa claim rena le ward allowance e eleng gore we can supplement that you see what I mean but ga ele mo central government ke mathata a sele ga gona motho pele because they had to bind with the regulations tsa bone CTO is not like CTU,CTU and CTO there are different,that's why gona le di BX nna re le BD ke gore ke.. private number plates because re bereka batho ,re mo bathong so jaanong I think..i think gone ha hela go le nosi when we move this to central government the way they operate the way local government operate is not the same , ee re farologanye tota so lets..lets go down lets come back to the people.<br><br>INT: To local government?<br><br>P.Nnyaa,ga kere come back to local government,ke raya gone gore lets go down to the people.	

INT:Ok ,ee..nnyaa go siame nte re utlwe Mme ka ha.Nna ne kere a ware gongwe o batla go boelwa ko local government.<br><br>P:Kene kere kana matlho gongwe ga a lebele gongwe.<br><br>INT:Ee mma<br><br>P:Ee,jaanong nna ke tsaya gore ga e sale tota goromente yo motona a tsaya di clinic ke bona o kare go na le mathata,mhh gone jaaka kere go na le mathata ke raya hela ka go tlhoka di transport, ne go na le transport tota e ne o fitlhela e thusa di clinic mme hela ga e sale goromente a tsaya di clinic tota o fitlhela go na le mathata a mangwe a matona dilo di shota ,ke gore waitse o fitlhela go shota ke gore o ise..waitse o fitlhela go shota hela le mo go nyennyane mo eleng gore golo gongwe go ka bo go tsisitswe jaanong o tla fitlhela dilo di shota tota kana ke gore re berekela mo di clinic re okomela dilo tse di mo di clinic kana mo o tla fitlhelang ele gore dilo dingwe di a shota mo di clinic hela jaaka sale goromente yo motona a tsaya ga gona dijo ga gona eng,ke gore ke mathata hela a a tlhatlhaganeng.<br><br>INT:Mmhh..yes..<br><br>P:Yaa.ok to add to what you are saying because of local government Ministry of Health but all of us we agree it was a good idea but the problem with the Ministry is evaluation and monitoring,they can decide on a new program but they don't evaluate or monitoring the program because when you are saying now there is a lot of complications,why not to sit and to see which part works and which one didn't work because you know you can say now he is SMH for circumtision,nna Im SRH we have to meet but the Ministry they are just starting the new program but monitoring and evaluation is not..what is going to happen or you increase the program ,you are not increasing the number of people, you mix the local government but there is no evaluation,everything is now… you cant control because you are not evaluating you are not monitoring because if the evaluation was there we were suppose to know now the fallen side of transport for the clinic part he works to mix but for the transport and the patient is not working lets sort it out	

,lets sort the problem,if there is evaluation..I remember when this thing DHMT started every Thursday having our boss here P3 every Thursday the DMHT are meeting in Mahalapye for... to see how they are going to implements but if there was problem there were suppose to meet to sort out the problem.. to solve the problem because if we say no..no now we have to sort we have to go back but if there is a way to solve the problem why not to invite after 2 years to evaluate and do solve the problem,<br><br>P:Gone.. gone if I may have to ask,whether Im not suppose to ask because Im a policy maker,kana when you introduces a new system you have to go phase 1,phase 2,phase 3,phase 4 ke raya gore why just jumping like,nte kere just jump and then lend like batho ba ZCC bamodimo (all laughs) because gongwe we could have gone with 1 stage,say ok.. re simolla ka region ya gore,from that region we go to another region,from that region we go to another region,water utilities just did that ga go a simologa hela bare just go sshuu just like that they started with Mahalapye,from there they go to Pallaroad,from there..step by step,step by step o bona gore ke raya jang,jaanong the only problem e ileng ya nna teng ke gore once..April 1,2 everything came,ke leng 2004,everything sshhuu just like that then we say aahh…where was the preparation,where was the preparation and you know..Im sorry my tongue was saying Im part of the system but ke raya hela gore kana o utlwa re bua we are not trying to analyse the problem now we are trying to see gore how is the way forward,how can we solve ..how can we solve the problem now so there is no body who can say we can go back ,we have to come up with the ideas to say fine this is what and both of us we riding the same boat will be able to walk out so what do we do,like he is saying now I think Im buying his ideas to say maybe we should start maybe try to evaluate from a certain area not just the whole country gore ba bare where are the…where there are big probems lets start there where we can sort it out in that small area and then go to another area,go to another area,you see.<br><br>INT :.Any other strategies,umh..umh we..we I think we many people have this strong ideas about the move in all the district so far?Any other strategies we havnt been tried,has worked or not worked?	

P: Gongwe we should also pick some new things from the youth, nna that I see, gore aahh...gompieno as Mahalapye district we have a senior pharmacists in the hospital, Im giving ana example of a... a unit,a unit within the health system who is now able to oversee the whole district and know what is happening and also to re-distribute resources as an when they comes,so nna to me that is a pass gore because we are now under 1 manager being use of help in the permanent secretary we are able to easily share all this resources with 1 overseer knowing what is happening within the district so in that sense I still see the light,if all the support can be afforded to the district.There are good things that are happening we are also able to redeploy even staff gore if we are having a challenge in one area in a clinic we are able to say lets look at the hospital,let us request 1 or 2 and strengthen ko clinic X so redeployment of.. more so it has been centralize..decentralised to the district also,they ke raya gore..although its centralized to ministry of health but as district we are still decentralized if the DMHT structures are put in place then management of the district is ..is decentralized we are able to manuvour our resources within district before we can ask for more.<br><br>INT: One of the issues that came up within the country there...that came was that a... they..especially people those who came..the health workers who came out of the local government they feel that primary health care is actually put under curative situation They think that that may actually be detrimental to the whole primary health care services, when we were in Marina we had policy makers from Marina some of the team from DMHT will say for every thing it has to go to Marina first and then through the Marina systems then it goes to Ministry of Health and then so the..the the its much longer there ,the decision loop has become much longer ,I don't know wheather that was the intention or is that how is suppose to work or its just the way they are implementing it.<br><br>P: Mmhh..nte ke botse,a kere Mme we have this Serowe hospital,Mahalapy hospital and Molepolole,I thought	

there were suppose to relief Marina ,a kere instead of getting into Marina we can have those doctors too,to beef up a kere? So that the clusters that we are talking about that's why we are going to decentralise re dirisa cluster system it was as if we have ,ke raya gore now we are trying to penalbeat what we have now,so that a..we..we reduce the number of doctors..ok distribute of the Marina and make because it is a district they can..le bone ban ne le.. they can make decisions.<br><br>INT:Ee.. no..ke raya gore ..it wasn't ..ke gore what I was saying the Marina district akere ..sorry the Gaborone district the primary health care is from Gaborone district they now..now their decision has to go through Princess Marina were as before the.. they had a different decision making,maybe lets look at it.<br><br>P:I think the,maybe like what Mma was saying it's a credit to put all the health services together for validing purposes,and the light that I have seen also was in terms of human resource especially on the pharmacist side,I think the..previously even though the results are not yet coming but the clinics were severely affected by the staff shortage but then merging them with the hospital that was an advantage of sharing resources,now when we share resources which are over spend will not… the results are not obvious from there but its an advantage of that.So now coming back to.. to preparations for take over I also agree that I think preparation for taking over was not well done because a lot of us understand the clinics.. a kere the clinics when they came they did not have their own budget they had to relie on the hospital on many aspects so in that way it made them under..even run by the hospital because of that financial arrangement but you cannot run primary..you cannot allow this..secondary to run primary health care because they should all be running concurrently each have its own full set of administration but that is not the case now.<br><br>INT:I think that's the biggest complain it's a regrographic rather than ?.Ok any other issues?<br><br>P :Yes.. I was saying what has not happen realy is ..is operational of this DMHT because DMHT are not realy operating what has just happen is that just assign people to those rural but there is no body..in paper they say	

there is a structure of… that is why people still going to Marina it should not happen,because princess Marina should be stand alone,DHM should stand alone,they should have 2 different people running 2 different thing,now what is happening in the ??? and that ??? dosent take too long you see,you will say no..no.. you take care in the stock which is… that's way it will never run so what now is neede urgently is to have a fully operational DHMT with structure,with its head with everybody,with its finances with everything that goes with it,that way it's a good thing it will actually be decentralizing your Ministry at the district level but now unless you have a structure and resources to run it will never happen .<br><br>INT:No..any other initiative.. that we were trying to address,Ok what are the things we have , Maybe people are aware maybe they are not aware that how development of medical school was issued to start training centres in the rural areas,if we can call Mahalapye and Maun rural areas so that is not..is not just at headquarters in Gaborone but actually this students will spend 27 weeks of their total training time in Mahalapye and Maun as part of their training and so.. I just want..is that something that you think can be helpfull in terms of medicine and why?<br><br>P:People will be helpful because you know traditionaly schools, the medical schools you know how is it as it says oh the medical schools through out all in the cities,they are looking into a… you know they are looking into text books, but then eventually when you graduate you are suppose to be send to the rural areas and that what exactly what Hournourable Senai was saying.This guys are detatched from the training when they come but now with this attachment every medical students will be able to know what a rural health setup is ,so they are actually getting the whole idea under practice so when they graduate they are very..they have the background already so it is very very helpful,there are getting exposure to the primary health a..a.. you know.<br><br>INT:Any..any other idea,otherwise we are going on..<br><br>P :Maybe the other thing also is that we should tell them the new thing that has happen is that post graduate	

turning into family medicine and where you are going into eventually what the vision is.<br><br>INT:Ok..well we share the vision with Ministry of Health although I have seen it in international services plan there,they are actually are not there,but a… a we.. because we talked about skilling ,multi skilling that you know we need a cadre that can do a lot of things,this is specility in medicine which is got a pharmacisian it's a specialist,it's a speacilist in primary care so that..this person is able to.. when we have a mother who is about to have a baby and they can do surgery ,they can do scissorian section and I come with appendixcitis they can do surgery and they should be able to do operation at Mahalapye hospital and if I come they don't retrieve(all laugh) if I come with..with a child who is sick they should be able to attend and ,so they are specialists but that are speacilist in breath of medicine and then…but obviously they are..you cant be the best paeditrician or you cant take the best care of children to the highest level but that means they can attend to most thing in so that who ever get reffered there further up is somebody who realy really need speacilists so this is a new cadre that we are training,and we are training them in Mahalapye..can you believe it? And also in Maun we have 2..we have speacilists people who are training..there are doctors that are trained as speacilists already there are second year in Mahalapye and also in Maun so all their 4 years of speacilists training will be spend in this places,in the 4 years that there are training means there are providing patient care they will be…from next year they will be in the clinics so that you will start actually to see Batswana speaking speacilising doctors in the clinics and so that over 4 years they should be always be 1 of them in the clinics and hospitals around so this is a new speacility that we are hoping actually will be introduce to be the speacilist in primary health care maybe in this clusters of the clinics and also in the hospitals because to have 1surgeon,1paeditrician,1 optician dosent work so that why if we have a surgeon but we also have this person who can provide for us and who can also do other operations then atleast we have so that's that,so that's realy that.<br><br>P :So that's the multi-skilling?	

INT:That..this..they are called the integrate speacilists,so th intergrate care because primary health care is about person is a person is not the leg or the head or the eye it's a prson so they try to treat the whole person.<br><br>P :Le ga o opiwa ke tlhogo kana o tshwerwe ke mala jaana.<br><br>INT:Ehee,and ..and le gore ga o tla o na le TB after they have treated you they say who is at home with you and they go to look at everybody who is at home to make sure that ga ana TB,so that's..thats the ...Anyway I think that's not..now one of the strategy that have been thought of for improving primary care may you please know this is the last but one also and is building effectiveprimary health care teams as a strategy to improve health care service at primary health care level,now what is you understanding of effective primary health care teams? Operation ..effective and then opposite the primary health care we have got team.<br><br>P:O tla simolla retla tla ko morago eish is not …<br><br>P:Well its..its a multidisciplinary team that should be..that should be there at the primary health care,is a multidisciplinary team,they have got a team work and they are all stationed there so is ..is a primary effective primary health care team will be like a family position and may an FMP,a midwife,a pharmacy technician,a laboratory,a .. a.. umm this one this a.. radiology,yaa maybe a radiographer ..a social worker so that's a team there in your bigger cluster mother clinic so you know you are realy doing all sorts of things at that level,and that is there in an intergrated health services so you know right now we might look a bit disjoined but maybe 10 years from now will be different hee.<br><br>INT: But maybe this can health us to start not in 10 years maybe next year.<br><br>P: Yaa..yaa this is eventually health care team that you will get that package.<br><br>INT:Ee so I heard your team ngaka does anyone..who so who should be in this team,you have already mentioned some people but who else should be in this team,team e	

go teng etla bo e provider health care e tshwanetse e nne le bo mang gape,o..P3 o setse a buile batho ba bangwe gape ba akanyang gore ban ne mo team e tla bong e provider, e fa bo...e... e.. eha bo bo bo bo..<br><br>P: Health educators, community...community yes,there should be someone from the community<br><br><br>INT: So Ok…and in that health care team that you spell out I ddnt hear anyone of you who is actually working with the community in that team, I have somebody in the lab, somebody in the pharmarcy, somebody…<br><br>P: The family with health educator could be one of them with… plus somebody from the village its self should be part of that team.<br><br>P:Maybe in the villages I know we have some VHC(Village Health Committee) I don't know how they operate maybe Mr Sebego can help us but my mind would be saying there are already there ,there are part of the system is maybe up in the relationship and the partnership.<br><br>P: And what could happen is that like for example we have hospital advisory board...I mean hospital advisory committee we could have primary health care advisory committee in that village so that we can have Kgosi for example ,the councilor so leadership there they will be sitting there and seeing the needs of people and what is it that..What the gaps are, so from there they can strengthen so that we also get the community participation of the team so that should be the team.<br><br>INT: Great, so what do we see as the value of this team? Team e keng... e botlhokwa go le kae,botlhokwa jwa yoneke eng ,are abo re ntse re oketsa jaaka Dr a ne a ntse a bua gore we kep adding one thing after another thing after another thing,what the value of this team?<br><br>P: The team is giving us the feedback mechanism as implementers of health care we are able to know what the community is saying or what is happening within our..our facilities because the community is involved and they provide checks and balances which are very	

necessary so that we don't just do it for the sake of doing it but we do it to add value to the life people we are serving so partnership with the community really assist us to be on our toes as health workers so ethically we are able to be seen to be doing what we can do.<br><br>INT: Who should lead this team?<br><br>P: Whats the question?<br><br>INT:Who should lead this team?(all laughs) Ehee..Ee Rra<br><br>P:Nyaa nna ke akanya gore that will depend on ballot,thats what I think full balance.<br><br>INT: Ee... ke khupelekhupele?<br><br>P: Eee(all laughs)<br><br>INT: Ok...<br><br>P: I ... think the leadership of that team either it's a it's a... they should be...it should also have similar you have ours like a management committee that side ok, it should be...they should be somebody dealing with the professional aspect of it,somebody dealing with administering aspect of it,somebody dealing with the ethical community aspect of it,all this three should be there and who ever you put there as whatever particular head at a different table,I think because the concept of team should come,you have to have someboby professional,you know the whole idea of this team is to have the standadised care through out the country,so the treatment for sugar diseases should not vary between Kopong,Gantsi and Mahalapye and it should be standard so everybody should get the same kind of treatment so with this team ,it will facilitate.Now how..like what that man was saying,how is it that we know..how do people know I wil be doing the job when we are happy,I will be doing the job isn't it?So now for that somebody has to evaluate,who should evaluate? ,there should be that team,I mean maybe the leaders team,why should we worry about the professional thing,ohh what the problem blab running,what..what..what once you know about the financial ,ohh what this is and once you know about the really the satisfaction whether the job has been done or	

not,that's a community involvement so these three people will sit together,that's it then who could be incharge of that there is another.. there is a DMHT again so obviously they have to report to the DMHT and then it goes like that.<br><br>INT:Mmhh..ba bangwe bar eng? Ee Mma..<br><br>P :Ga ke itse bcause we are talking proba..primary health care provision akere? And we are saying who should be the team leader ,that means team leader will be the one with know how on what primary health care intails,so in this sense there have been a debate gore should it be a primary health care speacilist or should it be the matron,the..the nursing or should it be anybody but to me we have been talking family medicine,so graduating in the long run I see them as taking the lead,if..if.. if there is no different between primary health care speacilist and family medicine,Ee I think that will be a way foward.<br><br>INT:Yaa..Ok..anything else?and then how are we going to.Ok..how should the quality of the team or the work of the team be evaluated and also how should its impact be evaluated?impacts of its work..Ee..<br><br>P:I think the community can…since the community have been a part and also the community will be the beneficiary ,they are the ones really to evaluate the effectiveness of the team and the quality of the team,like the UH, Health team,like the VDC chairperson ,they cannot from..ee since they will be in maybe they can be put as the secretary being given the power to report to wherever the report has to go in that way they will be the better people evaluate the team work.<br><br>P:Yaa also evaluation can be done in many ways,you see there are certain programs that the government is running obviously those are the monitoring tools,so if you have cut of point for infant mortality we have to maintain that to get better in this maternal mortality so audit will be done so people will come and say ok show me your book,how many children have you..what is your catchment area.,how many kids are there,.how many immunisation have you done, those are things that are ..that is your monitoring and evaluation so obviously because this..the basic idea of this team is to provide	

health care so they have to have those indecies of health care that they need to monitor plus the community aspect of it could be..could have pitch holes,you could also have your suggestion box and so they now will also tell us how to improve or where the gaps are so even if you for example you have reduce your martenal mortality but if you don't attend your patients in time that will still be an issue,or even if you have immunize all children but you are very rude that will still be an issue so those two issues should have to merge.<br><br>INT:Should we define the population that should be served by specific team ,this is not part of this Im just..as..as you are talking Im thinking because then how do I know if Im in gate ke eng..Airstrip clinic and we were told this is your catchment area should we defend up because some...the problem with knowing what my marternal mortality is I don't have a reliable denominator .I really don't know how many mothers mmm…<br><br>P: Are delivering. Ok no...Ok...<br><br>INT:I will leave it hanging and left out<br><br>P : Ok..no the problem is this thing when they are going to start to operate it,before they evaluate they have to fix some objectives to identify some problems and gaps and to fix now we are going to improve in that way,they have to have that standard of operation,that way we will meet them after a certain of time to sit and evaluate ,according to the ?team and the gaps they have been identify before to work.All the teams are saying no they are starting we have this problems shortage of midwives,we have the cluster,you have to top up with numbers of workers,and after one year you can say no if here ,the financial year we have to evaluate if we did well or we ddnt but the first thing they have to identify the problem fix the objective ,It will be easy for evaluation if they know what they are doing and what they failed and what they ddnt improve they can improve on the next year but first to identify the problem and to fix the objective.<br><br>P:I think not just the population only should be a denominator in Botswana because you see there is the vastest of the area and land I mean most of the people who live in the Eastern side and the Western side then	

people will be biased towards the East and not to...so I think we should look at the population as well as the distances maybe that way we could be able to see because even if some village are very far of ..maybe the whole team should be there even looking after a fewer people because we already know that there are teams offset the whole balance many will die in far off place and the whole balance case so i think we should look at the distances as well as population.<br><br>INT: No...what I was saying is supposing we decide on to set up a team here, shoud we say ok the team which is centred around Airstrip will set this radius,I mean in many countries like he was saying you will find that ,they will say ok this GP here will serve this population in this radius and so they will..they will..actually that allows you to know your population and know gore there are 10 families in this population wena you have an education assistant,I want you to map out all this ,how many young children under 5,how many you know…Im just putting it out because Im sure if we are implementing we will have to pip through all this things.<br><br>P;They will cost us<br><br>INT:Because in that way even records ..,you can keep records and be reliable in that when somebody comes you already know you can find their records in your system.<br><br>P:For a bigger village maybe you look into the wards,maybe you say this other wards are for smaller village you can look for smaller wards--------------right now we still have for example which clinics are served by shoshong and for a place like Mahalapye you separate it with wards to say which wards.<br><br>INT:Ee Mma<br><br>P: Also we are now going patient management system gore maybe in the long run we will be ble to network our systems that will be even easier because even if we have a visiting patient then we will be able to know from our system gore this is a visiting patient,this patient usually stays in Maun and now that there are travelling they are passing through they need health attention so I think our	

clustering will do us good in the long run in few hospitals but as cluster rolled out I hope it will also assist us in defining our populations.<br><br>INT:That's fine..now what do you think as this building effective primary health care teams as an intervention that should actually tried,Ok now this is the last question,this question is very different from everything you said so far that's why is the last question.If you read the documents that some of you know some of you don't government..Ministry of Health has written a nice document which is called Intergrated Health Services plan which has visions and mission ,it is a strategic plan of the Ministry of Health ,a ten years strategic plan in there it also has values,one of the values is ethics ,but everyone of our people that we deal with will be treated in an ethical manner but sometimes when you are a policy maker espeacilly working in a constrained environment you get,make decisions that are ethically challenge or sometimes morally challenged so this question really says,because of the constrains which you work have you had to work in a..ah can you tell of any kind of ethical or morally challenging issues you have to tarcle in your function because of this issue we are talking about.<br><br>P:Can you repeat your question<br><br>INT:The..the.. this is an issue of ethics gore we as government or Ministry of Health said we will treat all our clients ,this days they call them clients..when I was a student we always call them patients so I find it difficult to call them clients,all our clients will be treated in an ethical manner and..and as a policy maker you distribute resources.you transfer people you do all those sorts of things because you are policy maker,because of the concern of other issue have you..had ethically or morally challenging decisions that you had to make or the issues that you have to tackle in your function as a policy maker because of the constrains whether its in distributing of the resources,whether its transferring of people,whether is..whatever it is because of the constrains.<br><br>P:Hey…fokotsa<br><br>INT: (laughs)so thats why I said very interesting and	

different so that people can say yes and um..lots of many interesting things have come up as we travelled around the country.Ee Mma<br><br>P:Ee mma,going back to transport,if I..Im just giving an abstract scenario gore sometimes we have to decide,there will be a child who needs to be refered for some..for something,a mother who is delivering…who is having a difficult delivery and (maybe a patient who)maybe a cancer patient who have to go to onthology clinic and maybe there are going to their terminal state so those are the issues that we have and we have 1 ambulance to try and address this issues,1 to Franscistown,1 to.. to..2 to Marina different conditions ,1 needs to be a streacher case 1 is just a patient who can situp but we only have 1 ambulance with one streacher and you have to decide gore who are you serving first and why,what can wait and how long.<br><br>P:Still on that,the resources that we use sometimes we..we are not able to confirm their quality especially on the line of drugs,kana drugs are manufactured Just like any other commodity they state the conditions under which you should keep it,if they say below 25% you need to be sure that there is aircon above there,so in rural clinics there are no aircons ,no temperature control anywhere but we know we are giving a patient something that we cant gurantee the quality.<br>INT:Ok..any other example?hello..P5 is in deep thought(all laughs)Ok.. I think that's fine um..a.. thank you very much unless there is ..is there anyone else who has something you wish we would have covered that we didn't cover?Ok no.. that's fine this is the end of our discussion and thank you for your patience,.Um so the next stages are we will take this we are going to analyse them and then we will come back to you sometime this year to tell you what the overall result is and then also what the concernsus for our interventions but I think in all places we have been starting primary health care a.. teams come up is something that people will like to see it,piloted and I think its beautiful,yesterday we had people from Ministry of Health.	

