# Supplementary material for: Stakeholders’ Perceptions on Shortage of Healthcare Workers in Primary Healthcare in Botswana: Focus Group Discussions
Source: PLoS One. 2015 Aug 18;10(8):e0135846. doi: 10.1371/journal.pone.0135846 (PMC4540466; doi:10.1371/journal.pone.0135846)
Supplement: S4 Text — (PDF) [file pone.0135846.s004.pdf]

University of Botswana and Human Resource for primary health care Project
Transcription of health wokers focus group

Participant ID: Maun Focus group one(1)
Date: 28/03/12

Interviewer: N

Interview Duration: 01.50.33

Audio File Name: Focus group one(5)

INTRODUCTION

INT:……for us to hear your opinions on what we will be discussing, I have guiding questions that I will use in order to get your ideas and opinions regarding the theme on discussion. As I have said earlier everyone feel free to participate in the discussion and say out your views more so that every answer is right and there are no wrong answers. All your answers are very important. Research in Botswana has provedan escalating insufficiency in the health sector services; in hospitals and clinics especially in rural areas. According to you, are there sufficient health care workers in Botswana? If not, what is the cause? It is the first question; according to you are there sufficient health care workers in Botswana? But…

P1: yes, there are insufficient health care workers in Botswana, they are not enough the reasons being Batswana health care trainees are taken to overseas countries to study medicine but when they finish their studies instead of coming back home they decide not to and claim to be seeking for greener pastures.

Int: do they claim to seek greener pastures?

P1: yes INT isn't it that now they apply for posts at their preferred countries that they regard greener pastures where they will get better opportunities because in Botswana they are not paid, let me just say there is no much payment only foreigners get paid.

Int: Don't they go back home?

P1: yes INT they do not go back home.

Int: yes P2, thank you and what is someone else's view? What do others have to say? Health care services in Botswana?

P2: yes INT myself I take it that there are enough the problem is laziness that is too much, there is laziness.

Int: there is laziness?

P2: there is too much laziness, yes even if we can go to the hospital right now right here, eeh…instead of starting you will realize they are going to start at half past seven and then the doctor will show up probably around eleven and this delay eventually results in people becoming too many until the sun goes down, personally I take it that the sector is fine.

Int: mmh thank you somebody else, yes P3

P3: I say they are insufficient because the government does not pay our children accordingly, it pays foreigners from other countries, one other thing leading to the insufficiency seem too high is laziness, if the person is right and a patient comes to the hospital for help doctors and nurses take their phones and make some calls ignoring the patient, after so long when the patient becomes seriously ill and just about to die that is when they start running around thinking they can help the patient, that is what makes it appear though there are no workers.

Int: he/she is saying laziness and what are others saying?

P4: yes I am also saying there is insufficiency because if you can look into our clinics you find there is only one nurse in a clinic, such that if the nurse goes on leave for some few days the clinic will not function until the nurse comes back. That is to say if they were enough if one goes on leave the other one would be there to continue the work in the absence of the other nurse. You will also realize that even time really not only in rural areas there lack of time consciousness.

Int: time is not considered?

P4: yes time is not considered in the sense that you find that patients would have long came at half past seven at the hospital/clinic and the nurse will come around nine. People will have increased in numbers waiting for the nurse and when the nurse comes that late, it takes long again to start helping patients and eventually you realize people would all be helped around eleven had the nurse came on time but because of late coming to work some patients end up getting help after two in the afternoon.

Int: thank you sir, yes let us start with P5.

P5: greetings parents, eeh… in Botswana there is insufficiency of workers in general, as elders we have had illnesses for so many years, admitted in hospitals and undergoing some surgeries but as the other parent said earlier there is that insufficiency. Our children who go to outside countries and study medicine do not come back home because there is less payments offered in our country even in the health care sector. Secondly, even if they came back home after their studies Ican always repeat it is laziness; the laziness of our children is a serious setback to the government.

Int: when they come back, are they lazy?

P5: they become lazy and foreighners are better than them and now they come back to teach foreigners that here in Botswana things are done hap-hazardly. If you are sleeping on a bed and you say my child help me with that you will hear hei…this old man is so tiring, it is because the love of our children for parents is too low especially in rural areas some of us who live in rural areas children are weighed very late while people are gathering then they release one nurse to weigh children, the one weighing can not be able to do all the work alone, he/she is the one holding a scale; to write in the cards of children, really really the service is

insufficient.

Int: it is insufficient?

P5: yes sir it is insufficient.

Int: we are thankful P5, aah.. P6 wanted to add something that side as well

P6: thank you parent, I was saying the main thing of this issue as we are gathered liked this as youth the great thing is time and when you get into our clinics you find that you are taken back by the fact that I am going to do my personal things then Icome late to work. The great thing is time then we release one nurse to help so that we go out at the right time. At times you find that I come in the morning as a person and it is the blood issue, blood should be collected around 10 a.m and people are going to wait until 9a.m and at 9:30a.m that is when they decide to start they will be now opening. When you hear me say this, that the great thing is time is that must work considering time and if half past seven is the set time it is a must that everyone start work at their office at half past seven but we do not respect time really, if I come to collect blood and I come to collect blood at 10 a.m and then I come to the clinic to collect blood, I am sent back let me say the nurse is not there I am sent back and I go back while my condition is not known and I say things like those our people we are pulling heavily but it is us who cause that, us I am referring to our children and again our children instead of our children the government takes children from somewhere else and they are placed while our children have gone to out- side Botswana and when they come back it is said they look for jobs and they do not find them they have been given to those from somewhere-else and this thing this issue has to be corrected so that everyone lives where they are and to live I mean working happily and working freely now these things our people, we do not do them, I do not know if it is due to the government or it is us children or the learned ones I do not know and now I am requesting that we correct this issue our people children who study and those on transfer should not be pulled back when the person is suppose to enjoy the results of his/her country rather they are enjoyed by wrong people.

Int: okay... No I take it that we discuss a bit longer the one for health care services and whether it is sufficient, we hear what a lot of people are saying, now we have been talking about studies, they have been talking about studies, those are trained if the numbers of those who are trained is enough according to you when they are trained, that is the numbers of those trained enough, are they trained in enough numbers?

P7: yes
Int: yes P7

P7: yes… I do not understand there, that in numbers you mean in nursing or studies that people will then differ as per their duties? There I do not get it properly.

Int: yes that people who are trained in medicine, are those who have been trained, trained in enough numbers?

P7: they are not enough

Int: they are not enough

P7: not medicine only I mean the health care sector in general.

Int: the whole health care sevices sector

P7:they do not train enough nurses, enough home-based care workers, enough doctors

Int: mm

P7: as a whole the services department as a whole.

P6: it is insufficient

Int: it is insufficient

P6: it is not enough

Int: yes

P6: it is not enough because of the reasons that you hear me saying that

Int: okay…

P6: when he/she takes a patient to

Int2: do you think this is caused by insufficient numbers trained or they are equally distributed, what do you see as a problem?

P6: they have not been equally distributed

Int: mm

Int 2: are numbers enough, if we take the whole of Botswana as a country, do we take it that numbers are not enough, are they not trained in sufficient numbers, enough numbers or many have been posted in other places and they do not go to other places or both, we should think of what causes the shortage?

P6: yes, I was intending to answer it but let me give the others some chance.

P1: yes, the other time I asked, that as of your question, when we are given one nurse, he/she prescribes medicine for us and when she is still writing she leaves to go and help at the mternity side, when we who are waiting for pills are left he/she has gone to the one helping those who are about to deliver babies since that one is faster, (cellphone rings)……

Int: mm

P1: is it because there are no other nurses in other areas so we can also be given

P1: at times it becomes too much, then it is said that we are not enough, they have answered me to say there is no other nurse out there doing nothing so he/she can be taken and brought to us.

Int: mm

P1: the thing is when he/she is still prescribing medication for us, leaves to go and assist on the other side where wounds are taken care of, goes to.. Just to show you that there shortage in the services given.

Int: mm

P1: is it not that they are concentrated in some place?

Int: mm

P4: yes, it is there is shortage only...

Int: mm

P4: yes

Int: the training one what do we say about it, the shortage one we hear that there is a shortage, I do not know that somebody else…

P5: I take that the training one is a lot or enough

Int: it is enough

P5: beause really if we can look….

Int: mm

P5: year after year there should be those completing

Int: okay…

P5: every year they complete at schools, then you ask yourself where all these people go such that it is said that there is shortage.

Int: mm

P5: now really really you find out that,er… in rural areas especially

Int: mm

P5: really really people or doctors or workers do not like working at rural areas because of resources.

Int: okay…

P3: yes, like now when I am taken to chukumicho

Int: mm

P3: without a bank without what, it is going to be difficult for me to go and stay there, there is going to be only one person there.

Int: mm

P3: then you tell yourself that you will stand this situation, the other person will not stand for it.

Int: Mm

P3: these things are the ones that cause, er… lack. I do not know if I will say lack.

Int: yes, thanks P3…, P2 yes you were still saying something.

P2: no I wanted to add on that first question. I wanted to know whether this thing doctor…(pause) how long does he/she take working?

Int: Mm

P2: Or the disease itself when diagonized in a person takes is it not an hour, normally doctors take how many hours.

Int: Mm

P2: and the disease if found in a person how many hours does it take?

Int: Mm, I take it that we will get back to that one when we talk about other things athe end.

P2: okay…

Int: maybe we will answer it along our continuation,

P2: thanks INT, yes

Int: we will hear how it unfolds.

P6: I think that means that(…coughing)

Int: let us raise our voices so we can be heared

P6: like I said, when I look at this issue those who are taken for training it means only a small number is taken because, you find out that at times we do not have doctors, as for us at rural areas we really do not have nurses, you find that we are helped by one nurse who is also performing so many other roles.

Int: Okay…

P6: now I mean they might be taken for trainining in small numbers.

Int: in small numbers at school

P6: mm

Int: is there somebody who wants to say something?

P7: the question asks whether…your question says are people doing medicine taken to school

Int: Mm

P7: in large numbers or small

Int: in sufficient numbers

P7: sufficient numbers but the answer is the numbers are sufficient. The government takes er… so many nurses to school.

Int: Mm

P7: like that P6 has just said…because every year the government has graduating doctors.

Int: Mm

P7: every year the government has doctors who graduate.

Int: Okay…

P7: now the problem is that our children say the government does not pay them.

Int: Mm…

P7: now some go out and say they are going to look for jobs somewhere else.

Int: Okay…

P7: that is what causes shortage of doctors to us

Int: Okay…

P7: because there is no sufficient payment in Botswana

Int: Okay…

P7: that is what I see causing shortage of doctors

Int: Okay, thanks P7

Inter2: is it that when they have come to work in rural areas do they take long, I mean there might not be an increase but can they work for a long period your workers?

Int: let us hear what others are saying

P6: yes, no, they do not really take long, yes, the person can take two years then you hear he/she isn applying for a transfer that means he/she sees that the place is not good for him/her.

Int: Mm

P8: yes, when I come and go back to the one of numbers that they are taken to school every year, I mght take it that numbers are not enough, I say this that it is not enough because the numbers of people increase every year.

Int: Mm

P8: which means an increase on the numbers on people who are taken to school

Int: Mm

P8: but after being taken to school it is not that all of them they study for… that lot that lot study for one particular job

Int: Mm

P8: medicine jobs are… I take it that they are different, some you find working specifically with eyes the person can go to school specifically for eyes. Then that means patients who come with other different sicknesses he/she can not handle them and that eventually means the number is not enough to help people.

Int: Mm

P8: those that are Bangwato

Int: Mm

P8: yes

Int: okay, I take it that we can move further if there is no one who wants to add something.

P8: I know a lot of them

P8: because, it is (laughing) I do not know that I am going to answer starting from which point.

Int: Mm

P9: yes, I take it that really really doctors, let us talk about workers, wokers it it is in rural areas the truth is it is not many times that they take long.

Int: Mm

P9: given excuses some of them even a year the person can not even work for a year.

Int: Mm

P9: a person sometimes say to him/herself that he/she has a certain sickness that…er, doest not want me to live far from a hospital or where there is no some certain doctor, is it not that there are doctors, nurses there is what and what.

Int: Mm

P9: who really really will attend to asthma? Just like me I suffer from asthma er… that requires I live next to the hospital just like you can take people to Phikwe they say the smoke from that does..

Int:

P9: yes, it does not treat them well, the person end up coming for the whole month, before it finishes

Int: Mm

P9: the person will be writing letters seeking for transfer

Int: Okay..
P9: now this one that we say there are large numbers, there are large numbers because if there was a shortage

Int: Mm

P9: there would be no shortage of jobs

Int: Mm

P9: now it shows that you find that people we are many

Int: Mm

P9: those that go to school

Int: Mm

P9: that is why we end up staying home not going to work

Int: Mm

P9: I mean I am supporting the answer that I gave earlier the one I was …

Int: Int: Mm

P9: I answered

Int2: do you mean there some health care workers who have not been hired?

P9: they are there they can come out from homes

Int: Okay…(pause) now what are you saying about taking too long done by employees at work? Are you saying they should stay this long or when the do not stay this long, is it important that the take long or what?

P9: yes

Int: Mm

P9: INT, (coughing) children can not stay long

Int: Mm

P:9 reasons being that, this is the issue was said by our mother that side, it is because our children are not being paid

Int:Mm

P6: just like you heared me talk earlier mother was talking about how our children are not paid. He/she coud stay if he/she was getting a salary that satisfies him/her that makes him/her happy.

Int: Okay

P6: now for him/her to work and then say I am going to look for a space somewhere else I see that where he/she is he/she does not live well.

Int: Mm

P6: that is what leads to shortage of doctors

Int: Mm

P6: or shortage of nurses

Int: Okay…

Int 2: is it that they get salaries less than those who come from outside?

P6: so much

Int: okay thanks P6

Int: the other one… P5

P5: em… I support him. I was once admitted at the hospital during the year 2010, the month of February, expecting that hospital, is it Bokamoso the private one of Gaborone in Gaborone.

P5: I do not want to talk like this and this but there are other workers who were applying for jobs there but it is not a government hospital.

Int: Okay

P5: it is because there is good payment

Int: which is a bit better?

P5: This is a bit better.

Int: Mm

P5: that is why you see our children when they come from school from that side coming this side there is no payment put in front of them unless they go back home where they come from (they studied).

Int: Okay

P5: really if a hand can be stretched, a spoon being spread ontop of the one existing, our children can hold on to work anyway I do not know maybe it is their laziness.

Int: okay

P5: the laziness they display come from our mouths

Int: okay, thanks P5. Can we move further? Do you think that there are loopholes, shortage or problems that are related to health care workers at the clinics...the problems that are there or loopholes or problems related to health care workers at the clinicsor shortages?...(pause) which are the shortages that you think…

Int 2: maybe we can continue giving examples. Do you see our children having been taught enough for what they are suppose to be doing? These are things we can help each other think about. When they come here do they do what they were taught I mean these are some of the things we can think about or you think about.

Int: yes P1

P1: yes, our health care workers or doctors some of them I do not see them having jobs that they were trained for.

Int: Mm

P1: because there is too much negligence

Int: Mm

P1: now maybe I come from… what shall I say? Nigeria, I am a doctor.

Int: Mm

P1: but in reality I am not a doctor, I have just forgered a certificate

Int: Okay

P1: I am not a doctor, I forged the certificate and then I apply to the Ministry. When the Minister sees these forgered documents says okay... this is Mr So and so has done doctorate and sees all these documents in front of him/her all the forgered documents.

Int: Mm

P1: He/she hires the person and the person comes to Botswana.

Int: Mm

P1: the person comes here in Botswana... (pause) when the person comes here in Botswana he/she does not know anything because I do not know because he/she has forgered things that belong to somebody else's head.

Int: Mm

P1: what would you do it is just to come and panic after the other panic only

Int: yes P1

P1: yes

Int: ok

P1: then it happens you do not have a job. I say this because I have an idea that I can straighten up, it is just that I do not want to take... mm,mm, the long route

Int: yes ...

P1: I have seen someone who came from there with a stolen certificate from someone else

Int: yes, this P1 said something important; did you want to say something as well?

P2: yes

Int: Mm

P2: I was saying, just like P1 has said, some people do it some do not.

Int: Mm,

P2: they have laziness

Int: the work they were taught for?

P2: yes INT

Int: yes

P2: the problem now is on the other side they are demoralized by the resources they use in clinics

Int: Mm

P2: at times you find tha the person has to collect blood from a patient and you hear a person saying there are no gloves as for me there is nothing I can do to you

Int: Mm

P2: that means the person stops, the work does not proceed

Int: Mm

P2: those of you who have come for blood collection it means you go back home

Int: Mm

P2: tomorrow when you come again, the issue is going to take two weeks still being the same thing

Int: Okay

P2: there is no work progress

Int: okay

P2:yes

Int: thanks P2, can somebody else say something before we can move further

P6: Mm, the thing is we realize one and the same thing, yes there are loopholes

Int: okay

P6: loopholes are there but they are caused by... just like the woman who has just spoken, the resources used at the clinics...

Int: yes

P6: they are not enough

Int: Mm

P6: they are the ones that make a lot of loopholes visible

Int: yes, now are there any other types that are not enough? The ones with a shortage higher than others, the other types in the services that you see not enough as compared to others.

P6: Mm

Int: Mm

P6: so much

Int: is it not that we already agree to the fact that they are not enough

P6: Mm

Int: so there might be the ones that are not enough as compared to others. Which ones are those?

P6: some of these things like eye clinic resources or eye doctors

Int: eye doctors?

P6: Mm, they are not there

Int: there are no eye doctors

P6: so there is lack and a lack and they are not able to...

Int: they are not enough

P3: yes, they are not enough. They are too many as you see nowadays there is an outbreak of this our disease HIV/AIDS

Int: yes P3

P3: yes, as for the blood collecting machines really make us pull heavily, you can take the whole month going to the hospital for CD4 Count check up without any help.

Int: Okay

P3: then you tell yourself that if a person is sick and it is said tomorrow then he/she does not get resources

Int: yes P3.

P3: what is his/her future, (pause) they are too many

Int: yes sir, P5, is there something you wanted to add?

P5: they are many my of my own. As for us the elderly our resources are not enough. There are machines I can refer to as those scaring the elderly people

Int: yes

P5:Its just that I took about….(pause) nine months

Int :Mm
P5: I sleeping in a hospital but the machine here is not working.

Int: Okay

P5: I was transfered to Francistown. The machine at Francistown was well is not working. It is then that I started analysing myself on my own to say that is there any machine in the country that can scan me. I used my own money for transport to Gaborone

Int: Mm

P5: really I will say in all hospitals in general

Int: Mm

P5: they are not enough

Int: is it

P5: yes

Int: thank you P5, I am done with those that are not enough according to you? Yes

P1: yes, I take it that people, maybe people who live in town are better. As for us who live in rural areas your blood can be collected then you are told there is no car.

Int: Mm

P1: the blood it means maybe they throw it away because there is no where they can take it to. You can get to a doctor or a nurse to be treated then you are told there are no pills and there is nowhere you can find them unless you get transport and come here at Maun

Int: Mm

P1: but that means if you do not have money you will not come here

Int: okay

P1: yes

Int: pills, a car

P3: the other investigation our doctors are careless on us

Int: Mm

P3: myself I have a child who is sick, he/she has once experienced some damage on the head and it was said he/she should see… be taken for a scan

Int: Mm

P3: then it was found that he/she can not be scanned unless at Francistown. They then took the child there and I experiencing some money loss not communicating that the machine at Francistown was at default. From Maun to Francistown it is too much loss because I live in a place distant from Francistown.

Int: Mm

P3: I ended up using P1000 as it was said my child was spending a night there and they were saying people are there… they were asying doctors at Maun do not have a vision why did they not tell me the machine at Francistown was not working

Int: okay

P3: now I mean that is what is hurting us in clinics because they are careless on us

Int: Mm

P3: yes

Int: now do the workers get help properly? Help with the equipment they need, ideas and leadership, do the workers get the help they need according to you? The assistance that they need

P1: they get help because myself even as I am speaking nw I have not gotten my pills

Int: Mm

P1: I live in this area of Tsorogwe. I see the doctors nearby in Sedie

Int: Mm

P1: but if you get to the clinic you can be given paracetamol while you are sick

Int: okay

P1: If you have a pain, they tell you there is no a pill that can heal you that one of brufen, unless you go for it at Moeti or General Hospital or at Letsholathebe

Int: Mm

P1: but when you are like me not working and without money to get a taxi

Int: Mm

P1: tell me what assists you, they are not there they are not enough

Int: there is no equipment that they need

P2: yes, I am adding on that one that they are not enough because if I give an example the time when my child had dislocated his knee cap it was said his blood should be collected, I took three months digging that the blood be collected and they were saying there were no glasses that collect blood.

Int: Mm

P2: I ended up giving up and leaving that

Int: Mm

P2: ee

Int: thanks, okay how are the work positions in accordance to the payments…?

Int 2: That P8 had her hand raised up

Int: okay

P8: yes, I was workers who work at the clinics, them… what was the question again?

Int: it was saying do they get help properly?

P8: they do not get the resources that they need

Int: Mm

P8: because us we can run short of pills. Us really at the rural areas we are in shortage of pills. We can even have a shortage of plastics that pills are filled in

Int: Mm

P8: while it is said that they are not there, even injections children do not get injections at the right time

Int: Mm

P8: they have finished

Int: Mm, thanks

P1: bu t that one then you… you know some of these things, for example let us take the Sedie clinic you find a lot of traffic in there because you would have included people in all surrounding areas of Sedie then you find that even if there were some few pills

Int: Mm

P1: you are going to find that due to such a lot of traffic…

Int: Mm

P1: it causes some encountering shortage of pills

Int: okay

P1: now you find that the Sedie clinic if at least there was another one near it there

Int: okay

P1: maybe they could be assisting each other in a way

Int: okay, let us talk about the positions at work. Positions and payments, is their payments appropriate for the work they do fine, do the payments that they get correspond to the positions that they work under?

Int 2: because we know that a lot of you have already spoken about low payments, I do not know if there is anything you want to add. If you think it has already been said there is no problem

P7: positions of work refer to the way they sit or in the manner that…

Int 2: anything is it not that they can say they do not have accommodation or some say probably where they are going to live is far from where they come from. I mean just many things, things that you may think affect people that they do not stay for long

P7: Mm

Int 2: or that they refuse to go and work there

P7: that one I think we have already discussed it

Int: we have already discussed it

P7: because that P3 has been saying a person can go stay at Sekumo there or at Mhembo, without banks and nothing at all and now that is why people end up not living there for long.

Int: Mm

P7: at times being taken there but as a patient, with no resources to help him/her now that forces the person not to stay in a place where he/she can get help

Int: Mm

P9: I take it that we have already … even the one that they are not paid well we have already said it.

Int 2: we have said it , it means we should move further

P9: yes distance, let us talk about distance

P9: yes the other thing not going back to payments and distance

Int: Mm

P9: One person at our area came back from there without having even three months there

Int: Mm

P9: saying her child, she wants to take her child to a civilized English Medium school

Int: Mm

P9: it is many things we can not pin point…

Int 2: yes

P9: and finish them

Int: Mm

Int 2: yes. That one it si it comes afterwards…

P9: but if we continue well … it is there

Int 2: yes it is there finish it up so we do not leave it…

Int: okay, distance might have impact, distance, is it not that we come from different areas?

P9: Mm

Int: even some hospitals you find that they are in distant areas, do you think this distance has impact, distant from big towns like Maun and Gaborone in this manner.Mm

P6: yes Itake it that distance has impact

Int: Mm

P6: because right now we take it that our clinics, nurses are working… where they work there is shortage of resources , at times it is not the shortage but their laziness to come and take some of the resources they need at Maun like medicince and pills

Int: Mm

P6: such that at times you can go for treatment right now then you the person telling you there are no pills

Int: Mm

P6: when you have just given up then the person says bring the cards I will get you pills from Maun

Int: Mm

P6: that shows pills are there the worker is the one lazy to go and get them

Int: it is not distance it is laziness

P6: yes

Int: thanks P6, what is the other one saying? Others, we talk a bit talk a bit…(pause) distance

P3: distance can have an impact

Int: Mm

P3: Er, especially when we look at other places, er, since I like these places in this side, places around Serowe, you find that ..that for you to come from Sembuye, like you are that side and you have to come to Letsholathebe, you find that for you to come from there to Letsholathebe you find that the road there, for you to use it and reach your destiny you end up asking yourself so many questions as to whether you should start off or stay, you see. Such that later you end up telling yourself aah… I will go then tomorrow becomes tomorrow.

Int: Mm

P3: yes, people not getting help the way they may need it

Int: thanks P3. Let us talk about expensive or high prices of resources at rural areas, as to whether they also contribute. High prices of resources or the expensiveness of resources… may it have an impact?

P2: yes there is an impact because people have refused to come and work in rural areas

Int 2: it is just the same because prices do not differ for someone working at Francistown from those of the one working at Serowe

Int: Mm…P5

P5: Hei, at times it is a huge problem

Int: Mm

P5: because if it is in towns you might tell yourself that copensations of the things that they buy when they have gotten their monthly salaries

Int: okay

P5: now those who are at rural areas you find that as for them is it not that prices are just set to be high hence they then get some discomfort from that

Int: Mm

P5: even if they got money the monthly salary they are left with nothing, they are left empty handed even if

Int: okay

P5: even in rural areas this side

Int: yes, someone else, others.

P3: it has an impact because…(laughing) you find that those people in rural areas, you find that they even eat rotten food, food that really have expired. The thing is when you leave right now you buy fruits and you find that when you arrive there they have already been bypassed by time… is not that you are the one who buy more so they will last you long

Int: okay

P3: yes, you are going to end up having your rot, is it not this point of prices

Int: Mm

P3: that at Sembuye 1kg is P20

Int: Mm

P3: now if not that I buy it in Maun at P5 each, it means I should buy how many? 20 of them

Int: Mm

P3: then they rot then health goes down again

Int: yes, thanks P3. The availability of the job opportunities for companions of workers, maybe those who are marri ed or even those who live with their companions or schools for children might have an impact

P: that one I take it that ,that P6 has already answered it

Int: he has answered it; I take it that we can move on. Now what do you think can be done to improve the situation of healthworkers who are not enough at the clinics? What can be done to improve this shortage? To better this insufficiency?

P3: yes there can only be improvement that the government can focus more on doctors, nurses to empower them on what they have learnt.

Int:empowerment

P1: empowerment taught based on negligence.we are saying that others are not much into it.

Int: Okay

P1: You cannot be empowered on how to take care of the community then you neglect the community. You should have good menase and good behaviour for your work to succeed.

Int: Mm,

P1: without all these things your work cannot be successful.

Int:it cannot be successful

P1:yes

Int: thank you…

P6: Mm…even though I did not understand the question.

Int: yes P6, we were asking for your opinion on what can be done.

P6: okay…

Int:to improve shortage of workers

P6:if it were that money is not there at this time

Int:Mm

P6:I would only be intrested in being taken to school for doctors

Int:Mm

P6: like in doctors the government should have that intelligence so that women.a woman takes care of patients who are….but because where she will be going where she will be working she might endup meeting those with various illnesses.since am the government or that company ill have to empower her for a certain period of time.

Int:Mm

P6: It would be the beginning of what she has gone for to school.

P6: Okay

Int:it would be on our best interest that the government would be intelligent to empower Prince in becoming an animal doctor.now there is no place where there is a disease outbreak for him to gain experience.

int:yes P6

int 2:that is why we are saying not meaning to interrupt you

P6:yes INT2

Int 2:so that you bear in mind that there have to be specialist and they can cure many diseases

P6:so many

Int 2: yes being taught…..having high knowledge and able to cure so many diseases.

P6:I was there

Int:thank you P6

P: Okay,gentlemen the issue is that

Int:Mm

P6:the government is empowering our children…and this empowerment shows love

Int: Mm

P6:truly speaking doing this keeps people in rural areas,love,loving and working for someone is tough

Int: Mm

P6: it is tough but the biggest thing here is showing love to someone.

Int: Mm

P6: you will see you will be able to stay the thing is to be polite to everyone, without looking at the fact that how is this one,this one has a disability, this one how, using love

Int: Mm

P6: you laugh with a nurse, you laugh with a customer, you laugh with everyone, that is the love that you are suppose to use the love of God

Int: Mm

P6: love is the one that is going to keep our children in rural areas

Int: Mm

P6: because parents are going to love him/her

Int: Mm

P6: now when in rural areas you have come to love the old man only and you sideline me

Int: okay

P6: now you see this one of the challenges which are not good

Int: yes, you are saying they should be taught how to work with people

P6: to work with people

Int: yes

P6: love alone just like that is the one that can hold our children and our parents in rural areas

Int: okay

P6:because if you love us all just like that not considering he is an old man that shows love

Int:thats love

P6:not speaking out with anger us in rural areas over there we leave a patient right there mm and take care of him

Int: yah

P6: you get the issue

Int:its clear P6

P6:now the government has to teach us what the meaning of working with people....love

Int:thank you,what do others say,those who want to add someting before we continue foward

P9:yes,talk about empowerment and them being changed

Int:Mm

P9:transfers

Int:transfer

P9:if a person does not have any weakness,he must be checked,he shouldn't force himself of being asthmatic while its not there.he shouldn't be transferred fast ,maybe he will still be there when trying to leave and fast enough he is shifts

Int:Mm

P9:he asked for it and he was gone

Int: okay

P9:its because where he works ,he should be empowered ,if he is a nurse he should be a midwive ,the issue is that he should enough experience in the area he is at if there happen to be no doctor like us who stay outside,sometimes you would hear someone being refered to an eye specialist

Int:yah

P9: its just that you waste money travelling to see an eye specialist but why cant this nurse be taught how to treat eyes and she can also teach us as well

Int:yah

P9:nowdays being an eye treatment unless you come this side there are no longer doing it or a date can be set for eye specialists to come .its just that all these days if you have an eye problem there is nothing you can do ,unless the date is set

Int:Mm

P9: now nurses and doctors like you have just mentioned there should be taught various illnesses

Int: okay

P9:so that when i come telling them i have a problem with my leg ,i have a back probem or uterus e.tc and all these things he should be having them

Int:yes,P9 was adding on what has already been said that's P6

P3: yes

Int: yes, P3 did you want to add something?

P3:yes i think they have grasped something

Int:yes

P3:yes,i think for someone to be taught on that can be done but (cellphone rings) we don't know whether we can say its the government ,i think in the past there we associations such as red cross and what what ,i think they can also be empowered

Int:Mm

P3: whats wrong ,what are the challenges

Int: Mm

P3:so that you see where there is shortage of doctors or nurses they can also have a say in assisting lke today there is only one nurse over there.those people from red cross who have been taught whats that,community developers what do you call them

Int: Mm

P3: They should also be taught certain teachings

Inter: Mm

P3: they can have a say in helping the with the conjunction in the clinic,tey should help patients at a certain time

Int: Ok

P3: am referring to that it can also help

Int: Ok, thank you,now are there any jobs er....those done by health workers which you feel can be done by other people?...(pause)

Int 2: maybe i need to clarify that there are jobs that nowadays are done by doctors that can be done by somebody else not by name but other workers or nurses but maybe done by certain people a certain.....

Int:those with lower education

Int 2: yes

P5 yes,i believe thats the one i have just mentioned like today weighing a child,i dnt think weiging a child has expe...a person who is not even a nurse from nowhere can do it

Int: Mm

P5:Thats what is done by people of low class

Int: okay

P5:or distributing tsabana

Int: okay

P5: yes

Int: yes,ok distributing tsabana

P8:yes,P5 already covered it

Int: Mm

P8:because you would find only one nurses,i think weighing children and writing on the card is something done by other people,and at the dispensary they can take some one who knows how to read and write,do you understand?

Int: Mm

P8:i believe it is a lesson that can take a month and a person would understand

Int: Mm

P1: here is the medicine,you can give it to the patients

Int: yes, but did you want to say something?

P1: yes,i am saying that those that can be done by other people other than a nurse those that do not require much education such as distribution of pills,getting temperature

Int: Mm

P1:even weighing

Int: weighing ,yes

P1:there are so many

Int: Mm

P1:because it can refuse,cleaning in hospitals like washing toilets and washing maternity

Int: yes

P1: i think they do not require an educated person, they need someone who has done seven or standard 2.

Int: Mm

P1:he can you wash,clean and cook for kids that are there ....children with lower heads

Int: okay

P1: yes

Int: what else can be done by people withlow education

Int 2: maybe before you mention the rest you should specify who can do them.meaning that we are sharing opionins so that when we go to the health department we would be able to tell them the burden nurses have in their job which can be given to someone either social workers or volunteers or any thing.am just saying we can talk about it

P4: yes,there are volunteers

Int: Mm

P4:those who are there stay in a clinic they are the one who can do them because they have volunteered for a long time,they can take five years volantaring but when people are hired the would be no posts,these are the ones who can be placed in those posts.

Int: volunteers

P4: volunteers

Int: yes, P5

P5:jus like....infasizing on that ladys voice like those of home based care

Int: yes P5

P: those from home based care i expect that...its just a name they are not taught on how to do it

Int: Mm

P5:what do you do with someone who is sick

Int: Mm

P5:yes ,just like in rural areas,where do you see them just walking,if you ask them how they do it,no we are just checking someone there,really they are close to being doctors

Int: Mm

P5:so that some time they should be called to for leassons

Int: Mm

P5: yes now we need them for home based care when there are at cattle posts

Int: Mm

P5: now there are in danger where they go patients can have.....

Int2: virus

P5: a virus

In: okay, thank you, P9

P9:going back to P6 point

Int: Mm

P9: like accidents on the road....only those in the village according to their difference,we have volanted to the government he is supposed to teach us regarding certain jobs within a certain period,there should hold a fve days workshop

Int: Mm

P9: like,you can see that we are here now they should come so we can listen to these things.you wait till you are old from there

Int: Mm

P9:yes,that one has to be there,we have to show love and the government has to know that we are trying

Int: Mm
P9:with different reasons and patience and love and you with us together we go foward

Int: okay

P9: we show circumstances by

Int: Okay,does someone want to add on this before we go forward......according to you which ideas have been attempted in Botswana in trying to resolve this issue?ideas that have already been used or used by the government or someone ,ideas

P5: (coughs)...ideas that the government has tried to use have made a developments such as building clinics in rural areas
Int: Mm

P6: P5 has comeup with better ideas

Int: Mm

P6: now those clinics in rural areas

Int: Mm

P6:and they would have...(pause)their own sectors,like those who would be looking at these things such as VDC...(siren)

Int:Mm

P6:same as chiefs(siren)

Int: Mm

P6: now these facilities like these sectors are developments made by the government (siren) taht VDC and home based care move around checkingup on patients

Int:Mm

P6:those are what the government has tried to create(siren)but he has done them by word mouth

Int: Mm

P6: Just because people in rural areas as we have just mentioned there is shortage of workers(siren)

Int: Mm (siren)

P6:as for people in rural areas they send nurses and social workers but these people are said to be not working all of them a nurse has a time when he has a specific time when she has to go to rural the area there(siren)

Int:Mm(siren)

P6:sometime it becomes month end without them checking on the children just because the nurse did not go there,probably because they would be no car (siren)

Int: Mm (siren)

P6: so that she can go check on those people,that is why am saying the government can manage the developments its only by the word of mouth(siren)they are not working

Int: okay (siren)

P6: (siren) but even those who help people in their homes like the gentleman had just mentioned they are emphasising that the government should help people but the government is supposed monitor them,provide them with facilities taht can help them as they help peple in their homes

Int: (siren) Mm

P6: (siren)these are developments made by the government

Int: (siren) yes

P6: (siren) but they are no longer used

Inter: thank you P6, any other ideas that ....(pause)have been tried to resolve the problem,maybe is the government people in the community or any other stand alone ogarnisation.(siren)

P1: (siren) Ee INT ke ta tsena mo kgang ya home based care

Int: Mm (siren)

P1: (siren) many in rural areas you will find that they are jobless the reason been that you will find there are jobs when there are less patients,you will find that they are people who do nothing,there is no work.

Int: Mm (siren)

P1: such that the government has tried to implement this project.,but you can better it by empowering them so that they can help in clinics(siren)

Int: Mm (siren)

P1: (siren) they can distribute pills, suc as weighing children

Int: okay (siren)

P1:I think it would be better(siren)

Inter: okay (siren)

P1: he has tried mm just that he ended in the middle because people have been denied the opportunity tobe impowered so as to go foward (siren)

Int: Mm (siren)

P1:is now that they have put them selves in danger because what they used was not right(siren)

Int: (siren) Mm

P1: (siren) they are contracting diseases as well

Int:Mhe,what are other ideas?....(pause),maybe examples,introduction of school for doctors maybe they have tried to increase gifts ,these ideas they have tried to use them ...the reason being to improve that(siren)

P8:we do not know how they are paid (laughing),all we ever hear is how low it is,is very low,we don't know exactly where

Int: okay (siren)

P8: but i think trhe government is really trying(siren)

Int: Mm (siren)

P8: like today i think er, each morning before medicine is disrtrbuted(siren)

Int: Mm (siren)

P8: there are leasons that were intense before(siren)

Int: Mm (siren)

P8:i believe that alone is an initiative to empower people .now you find that they do not go as .....i don't know how to put it just that i don't know i would sometimes say in a wrong way because when you are teaching someone it has to be in a proper way ,now you would find out that aperson becomes impatient(siren)

int: Mm (siren)

P8:a nurse stands in front of you,and sometimes you do not answer her and she goes(siren)

Int: okay (siren)

P8: yes(siren)

Int: (siren) moving clinics from the ministry of Local government to the ministry of health, what do you say about it... (Pause), to move them from ministry wa Local government to the ministry of Health what do you say about?

Int: (siren)in improving workmanship

Int: (siren)Mm, in improving workmanship

Int 2: (siren)it is just they are ideas created by the government ,so that the movement from the ministry of Local government to the ministry of Health should make a difference in the labour sector ,taht is

P2: Ok (siren)

Int: yes P2(siren)

P2:even us who are near the labour sector sometimeswe o not realise first(siren)

Int: Mm (siren)

P2:but what we have realised is that after they have been moved from the council
To the government, we have realised that there have been shortage of high medicine like we have just mentioned .it is just that we feel that it is not the same like the first time in council where tere was shortage of pills it was not the same as now,now there is high shortage of pills.(siren)

Int: Mm (siren)

P:2 (siren)now we in rural areas over there we have a problem that in councils there hired security for us.when you got here been a patient we would wakeup the security people and they would wakeup the nurse.The nurse would wakeup,nowdays even if you come in labour pain you can even deliever outside because you are the one standing there ,if you know who you would ask to wake the nurse and they even held a meeting so we should not even try call them,if i just come and try to wake her up she would not even getup.

Int: Mm

P2: now it is not even in an appropriate manner and we kept on singing it pleading them for help it is not even important anymore.

Int: Mm (siren)

P2: (siren) though sometimes we sat down with the ministry of health we have tried to show her that we do not get help during the night and our clinic is even better because we have a nurse who deliever, even when you are not feeling well there is where your temperature can be cheeked at night till morning they can bring you here if it is not an emergency.

Int: okay (siren)

P2: (siren) now there is no use ,there is no use ,but at the council it is important.a nurse would check on u and you would feel better in the morning because she would have given you some pills,give you medicine for a couple of days and if you come back again sick she would giveup and you would stay home

Int: okay (siren)

P2:which means it was made in a wrong way(siren)

Int: okay (siren)

P2:they took our people from the council and took them to government sector ,it is not right for us not at all(siren)

Int: okay (siren)

P2: yes(siren)

Int: (siren) what do others have to say about this issue of been moved?

P2: (siren) yes,it is not right a long time ago but before when there was shortage of blankets in the hospital

Int: (siren) Mm

P2: (siren)if you are a woman and you are sick they would tell you that if you have blankets,you should come give them to your patient,and if it is an emergency they would ask you whether you have time and ask you to come help them look after that person you have brought in nowdays it is no longer there.people even steal nowdays with staff but before you could cook for a patient but now you cant

Int: (siren)Mm

P2: (siren) you cant dish for your patient

Int: (siren) okay

P2: (siren) nurse would be the one to stll them

Inter: (siren)Tanki P2, yo mongwe yo o neng a bata go lathela sengwe

P5: (siren)no that is the whole issue which the elders had raised,let me emphasise more on this one of development

Int: (siren)Mm

P5: (siren)yes nurses do not want to wakeup at night because they fear for their lives

Int: (siren)Mm

P5: (siren)or they came to steal ,which means she is not on a same side,which means she only work till half past five

Int: (siren) okay

P5: (siren) from there she is off duty.....,half four she is off for real

Int: (siren)Mm

P5: (siren)this puts a patient under hash conditions,this means even if a person become sick during the night there is no where to go

Int: (siren)Okay

P5: (siren) but there is a hospital just as P2 had mentioned ,there is a place where she/he can sleep at the midnight,there is supposed to be a service at night but because there is no hospital it becomes a white elephant

	But having a hospital just like P2 has just said, at times a person get admitted there in the middle of the night, it is a must that the hospital have a night service but because of the absence of a hospital it becomes a white elephant.<br><br>Int: Mm<br><br>Int: Thank you , but did you also want to add some thing<br><br>P2: yes ,but others i would not ,i still emphases taht moving fro the council to the government sector is not right for us<br><br>Int: Mm<br><br>P2: my voice is low?(Coughs)i was saying moving from the council to the government sector is not right for us because for us who stay in rural areas with only one nurse nowdays<br><br>Int: Mm<br><br>P2:we have days without a nurse where there would be using an ambulace and an ambulance sometimes when you report a patient when it is timeup the driver refuses<br><br>Int: Mm<br><br>P2: telling you that he is not paid overtime<br><br>Int: Okay<br><br>P2:he is not taking the patient anywhere,that alone does not put us in safe side because there is no way you can help the patient hei the driver refusing to take te patient to the hospital<br><br>Int: Okay<br><br>P2: Nurse would not be there as well<br><br>Int: Mm<br><br>P2: in a rural area<br><br>Int: yes, we are talking about ideas?<br><br>P2: Mm<br><br>Inter: yes, implementing leadership in health sectors in rural areas what do you say about it? What they call district management team......leadership......in health....what do say about it?

P2: (coughs)....i not sure whether i have put it right way anyway you will correct it

Int: Mm

P1: leadership in health,parents,there is shortage

Int: Mm

P1: i do not know whether it is lack of education in them or the number ,i do not know

Int: Mm

P1:as we have just now heard like us who stay in rural areas kids are the last step

Int: Mm

P1: they are not well

Int: Okay

P1: because he didn't come holding a child at night but she is expectant she is going to go back without seeing a nurse ,a person working is supposed to wakeup the leadership is the one who is supposed to take action,show te nurse or doctor your working limits .nowdays you find that it is being reluctant you find that if you are in rural areas a nurse would say no she is asleep

Int: Mm

P1:but since it is in rural areas those things are what we are saying there is shortage of leadership

Int: Okay, is there anyone who wants to add something? if there is anything we are talking of ideas and whether they can work.yes ,some of those things is been taught on how to be a doctor ,what do you have to say about it?

P8: yes, about starting school for doctors

Int: Mm

P8: according to me i wouldn't say it worked

Int: Mm

P8: just because it just started last year,will just have to wait and see whether it would be useful anywhere

Int: Mm

P8: but we were hopeful it would be useful

Int: Mm

P8: but we can be hopeful but there is laziness and the same thing would happen

Int: Mm, some of them,school for doctors

P8: i would say it has be translated because now this initiative was not there

Int: Mm

P8: because nowdays we are taking parents ideas

Int: Mm

P8:to show it has worked somewhere

Int: Mm

P8:because it would make some changes,improvement by the things we have just mentioned

Int: Okay

P8: it shows improvement

Int: Mm

P: yes

Int: thank you P8, P9 do you want to add something

P9: yes but they have already mentioned it

Int: they have mentioned them…

P9: yes, because there was no peace long ago…. Doctors,parents and the community

Int: Mm

P9the only education was that of our parents, our father the government

Int: Mm

P9: or the job faced P9 saying hes a mechanic …what do they say , of songs

Int: Mm

P9: of asking again whether wew would meet as we have met like this.

Int: Mm

P9: taking such leasons because those lessons are that of being taught and those of years ago of human nature such as those making plans being God given talent

Int: Mm

P9: am just saying

Int: thank you P9, is there anyone who wants to add something before we move on,yes,P6 has raised his hand

P6: no, P3 will add on that, let me add on that, i would like to emphases on those words they are really nice words

Int: those that am emphasising on

P6: that the government has made better improvements than before because long ago the bible was only read by men

Int: Mm

P6:i would read these word skipping some of the verses you see

Int: Okay

P6:from there

Int: Okay

P6:now the government has allowed everybody to open their eyes

Int: Okay

P6: so that everybody must know howto live their lives, how to stand

Int: Mm

P6: like we have gathered today we have learned so much

Int: Mm

P6: we learn such thatyou have come to teach us and we also teach you now what they are saying is that elders should learn from their kids

Int: Mm

P6: those are the governments initiatives

Int: Mm

P6: now we are the ones being lazy after we have learnt these we

Int: Mm

P6: you see now

Int: Mm

P6: you see now

Inter:yes

P6: now we are the ones pulling ourselves back

Int: Okay

P6: you see

Int:now according to you what is it that can make huge a difference to improve work in clinics ,that can make a huge difference

Some we have just mentioned but we can add on the ones we have just mentioned earlier, what is it that can make a huge difference in the health sector in clinics…that can make a huge diference

P5: Mm i think there should be an increase of nurses in clinics

Int: yes P5, nurses should be increased

P5: nurses should be increased

Int: Okay

P5: and to show them… and the government should teach those that are there

Int: Mm

P5: so that if they do not work hard what action can be taken against them

Int: Okay

P5: they should be shown the action tat would be taken against them if they don't deliver

Int: Mm

P5: because i believe nowdays there are actions taht can be taken against you
Int: yes

P5: the way they are doing it

Int: thank you P5, what else can make a huge difference? That can make a huge difference in improvement…we talking about improvement

P5: i believe if the government could…just making sure right, what did you say… increasing facilities because other people can have potential and it would be impossible for him because there are no facilities

Int: Mm, they are increasing facilities
P: yesINT

Int: ok, thank you P5

P5: Um…the government should be like other sectors that cant be mentioned

Int: yes P5

P5: there should have strict leaders

Int: Mm

P5: those that if a patient or a nurse is asleep
Int: Mm

P5: they should encourauge them to clean their work place
Int: Mm

P5: there are other departments which i just cant mention, those department stand alone we are here because of them

Int: Mm

P5:like other schools you are not supposed to teach while the headteacher iwatching

Int: Mm

P5: now in health there is laziness in the highest leadership
Inter: yes P5

P5: yes or they are just afraid that if they make noise…in nursing, nurses wont manage to work properly

Int: Mm

P5: we don’t know anything like that

Int: Okay

P5: to much laziness, from top to bottom

Int: yes P5, we have already talked about laziness, we really appreciate it P5

P1: i thought it would be better if the government would come up with another initiative of implementing another sector

Int: Mm

P1: he will stop at health

Int: Mm

P1: like it is now there is VDC there is also PTA now

Int: Okay

P1: so they can go around the hospital, being in hospitals watching staff whether they are working as well as taking care of patients

Int: Okay

P1: maybe it would make things better

Int: Okay

P1: yes

Int: thank you P1, what do others say?...(pause) other things that can make things better other than what we have just mentioned

P1: yes, i just don't know if in other government sectors there have wards such as water affairs

Int: Mm

P1: there is a ward where a worker doesn't cooperate is sent there for trial. With a worker doesn't do what is right he is taken there for trial.

Inter: Mm

P1: i think that even in clinics if it were like that there would be no laziness

Int: Okay

P1: yes INT

Int: now they have to create mini working sectors for health workers which they believe they are better ways of improving work in clinics in Botswana , what they call primary care teams, these are working teanms for staff, now what do you understand regarding these health facilities, primary care teams? Is there any one who has heard anything regarding these primary care teams?

P9: yes this is the first time i have heard

Int: primary care team

P9: yes it is the first time i have heard

Int:primary care teams

Int 2:yes, yah thats what we are saying you can come to a clinic and see a nurse or doctor ,we were thinking if tey could implement sectors,each sector madeup of doctors,different health workers each one having their own tasks if its weighing i would just come knowing who is this and that.if am sick i would know exactly whom to go to.......but it would be the same staff which knows ....thats complete.......that would give a complete health care ,thats what we are saying

P9: Okay

Int: whether it is something that… I do not know wh then a doctor over thereat the question is, I had just fit myself but really groups means that they work in groups not as individual nurses there, but they work as a team and as team members amongst themselves they know as to how we will handle the health of patients, you will do this and we will do that… we will do this together

Int: Mm

Int2: just like that

Int: Mm, yes P2 you wanted to contribute?

P2: yes, but I take it that they are there because there is someone who takes the blood

Int: Mm

P2: there is a nurse who weighs children

Int: Mm

P2: there is the one who takes temperature

Int: Okay

P2: I take it that those are the groups that you are talking about, there is a nurse whom a patient can go to be assessed, there is a doctor, I take it that those are the groups you are talking about

Int: Okay

P2: they are there the problem is the laziness of workers

Int: laziness

P2: That is what I was saying earlier that it can only be better if there can be a committee like Medicine, to come and put an eye on them so that if it sees workers not doing the work properly it may report

Int: Okay

P2: As with elders of Health, it will be better because it will be Police officers and the Security

Int: Mm

P2: as they fight crime look now they made

Int: Okay

P2: the nation better, theft has gone down

Int: Mm, P5 wanted to say something?

P5: yes, no as for me I did not understand, I wanted to understand something and now I understand.

Int: Now you understand, the other one

P5: like that P2 is explaining…

Int2: Now you believe that groups are there and they know themselves that as a group we are there and each and every one of them knows what to do

Int: Mm

P2: Where should he/she start and where should he/she end? Won't you be returned after being returned and being told no you go and see a nurse, the nurse tells see a doctor then go back and see that nurse, is it not that if it is a group each person will know that as for me I work particularly with this, that are the groups there, that is what we are asking… (Pause) and if they are there do they really understand themselves as to how they are suppose to be working, each and every one…

P2: I take it that it seems they are not there because for us to say they are there it is when they work together

Int: Mm

P2: Now it others are not there it means it is no more that team because on the other side it is torn apart

Int: Mm

P2: Because sometimes you find that your temperature is taken, after your temperature is taken you have to wait for your blood to be taken, and at times you find that the one who was taking your temperature is the same one who takes your blood.

Int: Mm

P2: Such that you find that one person is doing a lot of work alone

Int: Mm

P2: But a group the work is done by people corporately, the other one this and the other one this

Int: Mm

P2: I take it that at this point in time there are no teams as yet

Int: Mm, do you think that kind of a team, its importance, what could be the importance of that team if it is there? Yes P1

P1: I am saying a team like that can be important because it can lead to the work being done at faster pace

Int: Mm, the work to be done faster. Yes, you wanted to comment saying what?

P8: yes, no P1 has already answered

Int: yes, now who has to be in this team, who are suppose to be members of this team, this team can consist of who and who? P9

P9: Mm, maybe I can talk looking at… because

Int: Mm

P9: the health people like the woman who was addressing a community in the village

Int: Okay

P9: yes, because sicknesses are for us all, even doctors get sick

Int: Mm

P9: Parents it is the community but is is sick, maybe when we are together this thing

Int: Mm

P9: we can help each other think about and be able to diter what is to be mis-directed

Int: Okay

P9: Like

Int: discussion has mentioned the health people and those who are from villages. Who else should join these teams?

Int2: Who are the health people? Is it not that the team working at Nyanngagwe may differ from the one working at Sembuye. Now we are talking about clinics, and for us to as a certain clinic has a tough team we will say so refffering to the team which consists of who?

P4: Mm, er… I take it that we should say a clinic has a tough team

Int: Mm

P4: I take it that those who are important like doctors, there must be a doctor

Int: Okay

P4: I take it that they should start with the one who takes temperature until to the doctor, that team should be there

Int: Okay

P4: To avoid a lot of movement

Int: Mm

P4: yes, because if the doctor is not there, the nurse is not there that side

Int: Mm

P4: the work that was suppose to be done by a nurse it means if the nurse is not there

Int: Mm

P4: the expectation from everyone is that the doctor has been trained for every thing

Int: Mm

P4: even the nurse has been trained for everything so that when the other one is not there he/she can do that job

Int: Mm

P4: so that they

Int: Ok

Int: yes, thanks P4, others

Int2: So it has been said that a doctor

Int: yes

Int2: But I have not heared that who else are in this team

P5: doctors, nurse and those who are suppose to take temperature is it not it

Int: Mm

P5: But to take temperature, I take it that if a person has done Form 2, or Standard 7 the person can be able to take temperature that person can also be in the team

Int: Mm

P5: To take blood

Int: Mm

P5: when it comes to the treatment of a person, there shoud be one with knowledge about eyes, it should be… they should be trained for different diseases

Int: Mm

P5: They should not say they specialize in one disease, that will not solve this problem quickly

Int: Okay, I heared someone talking about those who are from villages what will be their role? Those from the community

P3: From the community

Int: yes P3

Int2: those from the nation

Int: those from the nation

P1: those from the nation should be like those watching over villages like VDC

Int: Okay

P1: those like the council, a Councilor who is watching over his/her district

Int: Mm

P1: no those who are from…. It is a parent who maybe would have volunteered, someone volunteer in a hospital a volunteer who is not working you see?

Int: Mm

P1: Those are the people who must be in the team, a volunteer, the Councilor and VDC as well as doctors and a nurse

Int: Okay

P1: they will remind each other to say, how should we do our work

Int: Okay, ok, thanks

P1: As well as the welfare ones

Int: As well as the welfare ones, okay

P7: I was going to answer the way P1 did

Int: Okay, yes P1 has said your opinon?

P7: Mm

Int: Okay, is there someone who wants to add something. Maybe we have left something behind

Int2: Are we saying there should be a doctor in each and every clinic?

P6: yes

P6: Yes INT, things are going well

Int: Mm

Int: should the doctor take a period of two years to three?

P6: Mm

Int:Okay

P:

Int: Okay, this team should be led by whom? Okay before we go there

P1: the reason why we say there should be doctor is that our district of Maun is too big and most of us we live at the cattlepost and at the villages and now if there is no doctor it is too difficult to get money to come here and again there is shortage of cars.

Int: Okay

P1: Each and every clinic in a district should have a doctor

Int: yes, thanks P1, now this team should be led by whom? Who is really fit to lead this team that we have been talking about, according to you? Who should lead it? P5

P5: (coughs) this team can be lead by parents in the village

Int: Parents in the village

P5: Parents in the village like you may say…or VDC er a Councilor or any parent who can be elected in the village

Int: Mm

P5: Because if it can be led by a worker, his/her reason to go out

Int: Okay

P5: because a parent in the village, even that parent should be assessed to see it is a parent who…er because there are parents we can elect who are at the village.

Int: Mm

P5: because we see that this parent has taken part in clubs there and there, and there and there

Int: Mm

P5: He/she does not have that laziness

Int: Okay

P5: yes, according to me

Int: yes, what are others saying? P5 is saying a parent in the village, he has said reasons why he is saying that, P9

P9: Mm, I am just like him, a parent in the village reffering to a Councilor or the Minister of Parliament of a particular district… because when things come to be there at Gaborone maybe his/her situation instructs the person to move fast

Int: Okay

P9: Maybe if there is shortage of pills mabe he/she has an opportunity maybe he/she has bought a car but an ordinary parent in the village may not be able to reach where he/she has been called to

Int: Okay

P9: Mm

Int: Thanks

P5: I take it that myself as an individual I will not take it that when I think that the doctor is the one who can lead since he/she knows everything then it becomes him/her who leads because he/she knows everything to be used in what or what

Int: Mm

P5: because everything that is needed in anything that can take place the doctor once a patient comes the doctor is the one who knows where to start with the patient, then how far to go and how far to go

Int: Okay

P5: now a parent really, really we would have put him/her in a situation which is not appropriate

Int: Okay

P5: because once I come here bleeding, a parent at home will not know where to start, until I get to see a doctor

Int: Mm

P5: yes, I take that mine says so

Int: Thanks, P5 says a doctor. He says this doctor should be led by a doctor and has given his reasons, what are others saying? P1

P1: As for me a doctor is just like a parent

Int: Mm

P1: A doctor has certain things that he/she does that is why you see nowadays in Botswana our children are not being hired to be doctors

Int: Mm

P1: Children from outside have been selected to come and be our doctors this side

Int: Mm

P1: Now someone from elsewhere can not come and lead our country, I think it is better a nurse is chose because nurse are the ones who are our children in Botswana

Int: P: the nurse is the one who can be elected to lead the team because he/she is the one who assists a doctor

Int: Okay, what is the other one saying?... The other one says a doctor, the other one a parent from the

community. What are others saying? Yes P8

P8: I want to add on the idea of that man that a doctor should be the one elected because he/she knows every form of their work

Int: Mm

P8: those who say a Councilor and a nurse tghey are workers, they have transfers, a councillor even when you have elected him he/she will always even when you have elected him/her

Int: Mm

P8: He/She is voted out, after being voted out and the person is gone where are you going to find him/her?

Int: Mm

P8: but the doctor all problems that can happen he knows them, he knows what he can do and how he can do it, now if you elect me just as a parent, I do not know anything, what am I going to do?

Int: Mm

P8: If a person comes bleeding, what do I do? Nothing, it is just to stand and tie him/her around with some cloths

Int: Mm

P8: Moreover, I do not know that I raise the level of the person's risks

Int: Okay

P8: because the cloths I use are not clean

Int: Ok

P8: yes

Int: thanks P8, what do majority say? Does it say a doctor or a parent in the community? When you say a parent give reasons to that and when you say a doctor give reasons why you say so

P6: Er… I take it that is a doctor together with the chief

Int: Mm

P6: It will not be doing because people who are working right now those who work as a team in this very time

Int: Mm

P6: we end up getting used to one another, if this one is not working, this one did not do, you think this one is as usual saying nasty and useless things but when the chief utters a word

Int: Mm

P6: they will say no why does it happens like this. I take it that we can continue and tighten things and then get back to doing things right

Int: yes

P6: yes

Int: yes, a lot say a doctor. Ok, let us move further. Do you remember some time when you were sick or you saw someone sick or a sick person that that you talk to the hospital and was treated unfairly by a health worker or treated in a discriminative or diminishing or despising way? A certain person that you once saw, or yourself or someone you had taken to the hospital. Yes P3

P3: yes to get into that one. Anyway I did not take anyone to the hospital

Int: Mm

P3: I had just gone and I found people complaining. I then ended up going back without any treatment

Int: Mm

P3: And then I went and stayed at the clinic and I stayed at the clinic

Int: Mm

P3: And when I was still waiting there, then came a sick person

Int: Mm

P3: the person was sick but did not have a card and I could see that the person was very sick…(pause) I could see that the person was so sick and I was waiting to see whether what people are talking about is what really is happening

Int: Mm

P3: Personally I know that if I do not have a card

Int: Mm

P3: Or not having P5.00

Int: Mm

P3: I go see a doctor and they write in my card that I owe P5.00

Int: Mm

P:3 is it not. But the person went in He/she was sick

Int: Mm

P3: it means they talked about inside and then the doctor refused to give him/her treatment. He/she then came out and told me that this person refused to give me treatment

Int: Mm

P3: I then took the person and said let me go and find you P5.00 and I went to find P5.00 for the person…

Int: did the doctor refuse to help the person because he/she did not have a card?

P3: because he/she did not have both the card and money

Int: He/she did not have P5.00

P3: Then I went I went to find P5.00 for the person. I came and gave him/her the money and the doctor healed the person.

Int: Okay

P3: Then I went in without a card and the doctor refused to attend me as well but anyway at first I could…some people he wrote them in papers

Int: Mm

P3: Writing for them in papers, just a lined paper like this one turned into a card

Int: Mm

P:3 Then he/she refused to attend/treat me and then it is fine I will go ask for a paper from the Police officers because you are refusing to give me a card and you are also refusing to write for me on a paper

Int: Okay

P3: It means I should go and I ask for a paper from the Police officers, because there was Police woman there the doctor then said no let me attend/heal you. Then I made him/her that He/she has been refusing to attend a very sick person and even told him/her that if that person could have died because he/she refused to attend the person who came so sick to the hospital, that he/she was going to be responsible for the death of that person

Int: Mm

P3: then it meant that how it was and we had friction

Int: Mm

P3: then that meant I was not speaking well, I was not suppose to talk like that in front of the community

Int: Okay

P3: Then I felt that was not right and it was some form of discrimination because people were talking before I spoke.

Int: Okay

P3: yes

Int: okay, thanks P3, P1

P: thanks INT

Int: yes

P1: I took a patient, I have an aunt who is seriously ill, she is the one that we took to the hospital, between eight and nine and then we got in, and we given chairs to sit, nurses welcomed us and they made her lie on a stretcher, before they, they caller a doctor, the phone rang…the doctor says he is resting

P1: (laughing)

P1: people we are seated here, just like we are seated here, and people kept on asking him, you are resting?

Int: Mm

P1: Oops, we waited, now the person's condition was worsening, ad the nurse kept calling and the doctor kept on saying he was resting. Later the person, ladies this person is leaving, then they took a screen and placed it between us and the patient because they saw the person was now tired.

Int: Mm

P1: They continued calling him and later the doctor came and uncovered the sheet from the patient… (Laughing) uh! We were there and then giving up, we then went out since we had given up, that is why when you stopped my question earlier and you said I should hold it right there, you said it was going to be addressed later I wanted to know that this person known as a doctor works for how many hours and how long sickness takes in a person. Yes now that is the question

Int: Mm

P1: the one I am telling you about

Int: Okay

P1: now I want you to tell me right there, about the doctor as to how many hours he/she really have to work

Int: Mm

P1: And the disease in you or in a child, you perceive it to take how long?

Int: Mm

P1: yes INT, that one I take it that INT 2 will answer that one for us, INT 2

Int2: No, I take it that that we have come to help each other think, is it not that those are the things we want to improve

Int: Mm

Int2: Is it not that you know that a person, if you do not get help when you are suppose to be helped that very second, that is when two days come in.

Int: Mm

Int2: now that is what we are helping one another to think on what should be done that if the doctor could realize that the person needs help in how much time and help them quickly, that one will be helped tomorrow, that is what we are looking at that how could that be improved

P1: is it not that when a doctor is phoned he/she should come

Int2: that is the thing

P1: yes

Int2: that is what we want you to say how things can be improved

P1: Can he/she say I am resting

Int: yes let us hear, P6

Int2: P6

Int: P6

P6: our people; that is why we were talking about teams

Int: Mm

P6: it is VDC, it is a Councilor, a volunteer or with a combination of a group of nurses

Int: Mm

P6: these things

Int: Mm

P6: then they advice him/her just like we are giving each other advice to say no you should do this and that, this is what you are working for and that is what they pay you for

Int: yes P6 we were still talking about your experinces what happened to you or to someone you know

P6: Ok

Int: or the one that you took to the hospital is not it?

P6: yes

Int: so that we do not go a little bit back

P6: yes, we are talking improvement, that how can this improvement be done

Int: Okay

P6: because this was complaints is it no it?

Int: Okay

P6: now how can this be improved

Int: Okay, yes P6 that one of complaints we have already talked but we will add on it later as we

P5: this one of complaints sir I want to interrupt

Int: yes P5

P5: now the doctor works this much of hours, for this period

Int: Okay

P5: No but these ones are the ones that demand us to correct them

Int: Okay

P5: so that it comes to the right condition

Int: Okay

P5: That is the issue I was trying to say

Int: Okay

P5: yes it is fine others will take it further

Int: thanks

P5: thanks

Int: yes

P1: I was saying I once took a patient; a child to my grandmother…coughs. He/she was in a shocking condition but he/she ended up dying, he/she died at the hospital. I went with my aunt and his/her sister, it was me who had brought them to the hospital, when we got in we found a nurse, the nurse then parted with the patient. Helped him/her lie on top of the bed. We were sitting outside, the nurse took the phone and started calling friends. Then I got back inside andasked the nurse, do you see that we have long came here, and when he/she answered me he/she said; I have just called the doctor, I do not when he/she is going to come because I do not instruct him/her to assist a patient

Int: Okay

P1: The other patient died I forget his/her name, how could he/she not die when you do a job like this, right now we are going to destroy your job

Int: Okay

P1: then the nurse got serious and called the doctor, when the doctor came the person had already died. They could not allow the person to spend a night and they said the person came dead already

Int: Okay

P1: the Police will be called before they take the dead person to the mortuary, we came with a person still alive and he/she died because of so much delay by the nurses and the doctor. I mean that is how the person died at the hospital, they did not help him/her at all

Int: Mm, P5

P5: um, oops, myself INT I am the one who was taking care of my treatment in 2010 on the 8th of February, I got sick, around half past three in the morning. I came at half past three, the first hours

Int: Mm

P5: now that situation eventually affected

Int: Mm

P5: that situation affected my children, at times doctors do not care, they look at people and say this one I eat with at home or what I do not know

Int: Okay

P5: Now it is not them, the government is the one that has left the doctor to do as they please

Int: Mm

P5: at the freedom square actually... (laughs) is it that you see. There are three

Int: thanks P5, er… P8

P8: myself I will speak like P5, myself as well myself I would be myself. I once went to…it is some days ago at Seleka clinic, I went to see a nurse (pause) at Maun. The doctor there I was attended by more than one person who were the owners I found sitting on the chairs when I arrived. When they started to help me I then listened to the other thin one and went back where pills are taken. That woman comes from our neighbouring countries, pills are not there, after I had asked her for about four minutes and I said woman of God I have reffered to this hospital from the clinic and you are saying there are no pills, are you not giving any other way of a private doctor, what shall I say, what should I do, myself I do not know… (Pause). I went and sat and said let me and go and check at Boitseme clinic. Is it that when things are not there in the market you do not do a certain writing to check for these things, it is failing, since God is there, it is just to go back.

Int: yes,thanks P8, P6 before we go back to you

P6: yes, I was saying concerning medicine department INT medicine department has a problem

Int: yes P6

P6: myself some days ago in 2011

Int: Mm

P6: My mother was sick, she was taken here with a high level of urgency, she was taken there on the 7th, she was taken there and the doctor was from outside countries, she then came from that side to Francistown. The same doctor who came from outside us the one when the woman was vomiting blood she said she will see her on Friday, Sunday, on Sunday she was still vomiting blood, when we say doctors do not care we mean that boss

Int: Mm

P6: Then they keep on saying to the person er… I will come and see you on Monday; I will see you on Monday. Such that on Monday when she came from there the doctor started crying saying I have made a mistake. That is why you hear me say that…mm, the government must take care of people, behaviour, love is God's because if you can in a person

Int: Mm

P6: Love, they have this thing of joking with other people's lives

Int: Okay

P6: They do not treat people well, at times the person thinks that the other one is joking then eventually the person dies

Int: Mm

P6: but about today

Int: okay, thanks P6, P8 over there wanted to add something

P8: yes, even myself I hear that also happened to me through a child to my grandmother at the Sedie clinic

Int: Mm

P8: After she prescribed some pills for me I went to where pills are taken and found there were not there so Icame back to her and told her there are no pills and I can not walk to go and collect them more so that I stay too far and I do not have money and I am sick

Int: Mm

P8: when he/she answered he/she said to me you know I can not hear properly, or my mind is not functioning, or should I give a way to others who are sick

Int: Mm

P8: I went to the nurses and they said the Social worker was not around, please let us go you help listen with me that woman is not speaking to me well but I am sick and I was only pleading with her kindly to change some pills for me because the ones she had prescribed for me are not there and I do not have money to go and take them at Boeng, Iam wondering to Boeng what are you transporting me with or you are taking me there with a car. That woman went to talk to the doctor and then the doctor said if it is like that in Botswana if she has written she has written and she is not changing.

Int: Mm

P8: now when the person can die while you have prescribed pills and they are not there what are you going to do then she said it was not her fault, it is not my fault, leave that. I mean those are things that do happen so much.

Int: Yes P8

P8: Yes

Int: Thank you, maybe we should take the last one

P1: INT I do not see anything wrong with the doctors I blame the government because I do not know that if they are hired they are taken through an assessment to see if they are positioned well with their certificates. To really see if this person is a doctor or what. I see the government to as the problem.
Int: With the government

P1: they are not or when they do applications they are not assessed, they must be assessed to ensure that their certificates are real

Int: Okay

P1: Or it is the ones that a person has stolen from the other person then he/she writes

Int: yes mam

P1: yes then it is said it is going to Botswana

Int: Thanks P1, you have spoken an important issue. Er, I take it that we have come to the end of this conversation but once again I want to aplored for having taken your time to come and talk to us. Your comments are very very very important but we promise that the message that we got here like I said when we started we won't mention anyone by his/her name where this will help in making some developments.
