# Supplementary material for: Stakeholders’ Perceptions on Shortage of Healthcare Workers in Primary Healthcare in Botswana: Focus Group Discussions
Source: PLoS One. 2015 Aug 18;10(8):e0135846. doi: 10.1371/journal.pone.0135846 (PMC4540466; doi:10.1371/journal.pone.0135846)
Supplement: S5 Text — (PDF) [file pone.0135846.s005.pdf]

A qualitative study of Human Resources for Primary Healthcare (HURAPRIM) in Botswana (Focus Group Discussions)

Date: 29/03/2012

Facilitator : Dr N

Interview Duration: 01.49.28

Audio File Name: Maun Policy Makers

HURAPRIM (Maun Policy Makers)

Int : okay so we are going to start mmh...I... we...now I think we all know each other although we didn't ask Part 2 and Part 5(laughs) No No! And we didn't ask them introduce themselves but I...I hope I...if they are not...know ooh! Maybe i don't know maybe quickly they can just say...because everybody did introduce themselves. Just in case somebody doesn't know who you are

Part 5: Can we start with you?

Part 2: Ladies first

Part 5: (Laughs) Am Part 5

Int: Okay!

Part 2: Well am Part 2

Int: Okay, well, you are welcome! So as i have already explained so I am going to ask questions about the health care workers in the area, and there is no right or wrong answer, okay maybe before we start am very sorry maybe we should have some ground rules I know you are all very busy people and you need to have your phones on. But may I beg that at least they be on silence so that eh... (Participants whisper) and...And eh...any other ground rules how you would want this to be conducted. Okay! I said there are no right or wrong answers so we will respect whatever anyone says without eh...making them feel like no they have said the wrong thing and we will...I will appreciate it if one person at a time speaks so that

we can actually listen to each other. And eh...because there is no right and wrong answer so that we hopefully can come up with something that will truly help us as a country. So as I said the study is about health care workers, human resource for primary health care. And I think as a start I would like to know your understanding of what primary health care is.<br><br>Part2: Actually primary health care has been defined in several ways, but the way i understand it myself it is eh… essential care which actually is offered at the community at the community level, and eh…with eh…with their participation. It's a care which is ummh eh eh…has got community participation and it should be seen to be sustainable. I think these are the important things about primary health care. I…i have said so because there is another aspect of primary health care where some actually people use it has if its normal to mean primary care where you know people go to see their GP. But I think it goes beyond…beyond that. Its actually essential care given to the community, in which the community participates and which should be seen to be sustainable<br><br>Int: yah anybody else has something to add<br><br>Part3: yah mmh… Yah i agree totally with what Part 1 has said, but maybe to add one more thing would say eh… primary care is that package which is scientifically proven to be working. And number two it has to have accessibility, the affordability and the acceptability of the community and effectiveness, one of the chararistics of primary health care, efficiency and eh…<br><br>Part 1 (Adds) Equitable!<br><br>Part 2: Equitable! Yah! Those are the three (E)s and the three (A)s of primary health care<br><br>Int; okay anyone else want to say something? Okay are we happy that they have covered everything? Okay! Now, studies have shown that there is a...a shortage of health care workers in primary care especially in rural areas and even in Botswana,	

now in your opinion is it true? Are they enough or are they not enough health care workers in Botswana. If they are not enough, why do we think it is so?

Part3: okay I will set the ball rolling. I...I think that eh...I think I will turn to conquer with those tallies, eh... from my observation point of view because actually I have worked in the health care delivery system like since 1988 eh... for 4years in Zambia and the rest here in Botswana where I came in 1992. And my experience has shown that actually that eh...when you work in a clinical setting you will see that most of the cases which come to the clinic could have been prevented at eh...at an earlier level at the primary health care level. And you saw most of these cases they surface at the clinic which points to the fact that there is something lacking in that area of prevention. So I think I will turn to agree with those studies from my experience as a clinician for many years.

Int: Okay! What do other people think?

Part2: (clears throat) Mmh...! I think on the surface of it you can say there is a shortage but there is also a strong component of allocation insufficiencies we don’t allocate workers where they are supposed to be...the primary health care level. We turn to allocate more human resources at secondary and tertiary level

Int: Is...Is that something that people conquer with is is...do we want to say a bit more? Ee Part 3!

Part3: (Clears throat) well i...i wanted to...to add another point saying the...you know there is a a... geographical discrimination in the distribution of a... health care workers or service providers because you will find that eh...there are certain areas which we agree eh...that they don’t have enough...enough manpower maybe primarily because there is no infrastructure associated with that area being desirable and I think to some extent...you know the word rural is not enough especially the context of Botswana you find that we have rural areas then we have remote areas. That is why the government have got remote areas dwellers policy which are even more isolated than the rest.

Int: Okay! Yah still on that...yes Part 2

Part2: Yah mmh! Just to add on what everyone has just said that really in way yes we are still lack eh... manpower to cover all eh these clinics/health posts that we have in the country. Apart from that we have most of the people in urban areas. No one wants to go to rural areas. And the eh...very few people are found there. But even then, even those...am sure we appreciate one point that Botswana actually even the remotest part of the country there is at least a health post. There will be a clinic somewhere, there will be a nurse. But probably what we are looking at as as a main constraint now, those people that...that happen to be found in those areas i think in a way we have not empowered them. We have not given them that empowerment to... you know to manage those cases...to manage cases effectively in those areas. That’s why most of the time you will realise that patients who come from a remote area, a condition that could have been prevented you know from getting worse or complicated would have advanced so much by the time it reach maybe a district hospital or the primary health...primary hospital level because there has been a delay, because people have not been able to read the condition not say this condition we cannot manage it here eh...we immediately need to refer to a higher level of mmh...Attention. So there is that lack of empowerment in terms of eh...education.

Int: Okay! So...so are we saying yes we agree there are not enough?

Part 3: Yes!

Int: And then we have talked…talked about distribution being an issue is that is that something that we agree about? Ee Part 4!

Part4: Ee! we are about the distribution that is not equitable, like ee others have said and we find that in other areas, gape the equitability think the problem lies with the norms cause it looks like in the country we don’t have norms that we are going that we are following in the distribution of health and power. And then the other problem we have in the country though maybe at, for instance in the cases of nurses you find that they at basics we have general nurses but we have got a

critical shortage in specialised nurses.

Int: Ee, ee Part 10!

Part 10: okay...yah! I don’t want to derail much on the topic, on the question of availability of manpower there, but i want to add something on what Part 2 had said, Ee! He said the performance of a health worker at the rural areas it seems it is lower when compared to those in urban or city areas.

Int: may i just without cutting you short say we we think as we proceed we will go on to cover all those, are you going or maybe i will just let you, but what i just wanna to say is this is just at this point we talking about the numbers, the adequacy of them, but we will cover other aspects of the human resource, but you can finish your point.

(Participant quickly answers)

Part2: yes! Actually i just wanted to say I mean the level of understanding of the people there, on the use of medical health care system maybe its low such that you find that in those areas they are other alternatives sources of health care or medication, Eh… which therefore limit the consumption of the health care resources.

Int; Ok no thank you very much, o ne o batla go ......and could there be other issues? Ee! Maybe as an example, are we training enough, are we keeping them, are we you know all, yes sir!

Part 3: Ya! Thank you F! Aah… i agree there is shortage; i know at some point the international standard would say the ratio eh… the nurse patient ration should be should be 1: 25, however there is a point when Botswana was 1:225 thereabout , that shows indeed there is a shortage not only in the in the rural area in the primary health care but at all levels of of health care, because the same would apply even to the doctors.ee now to get back to to… it is also of course agre… eh aggravated by the education system, i think the education system does not address the needs of the the community, neither does it address the any needs for that

matter be it the national or international needs, because what we have been experiencing over the past years the world over, is critical shortage of… health professionals at ee different levels, so i think the government should have focus instead of sending a lot of people in areas which are not critical, with all due respect to other professions i would say admin…public administration like i did, i did my myself i but I don't think it was a skill that i was critical in the community development, so i think we are not training enough, neither are we are we trying to address it at at its lowest point, for example the use of the paramedics and other you know semi health eh…skilled you know, professionals that would eh eh eh… help alleviate or arrest the situation, i think we are not training enough, and i think we are not even trying to retain enough, we are not even trying to recruit enough.<br><br>Int: maybe someone want to say a little more about those issues because they haven't been said! Ok, Part 4 and then.....<br><br>Part 4: Ya! Mmm! thank you madam chair, it true the shortage is visible to the eye, and eh… worldwide it is known that Africa has the smallest health care workforce, and that applies to Botswana more so with the event epidemic of HIV, our disease have gone beyond what we even expected to have at this time, we may have trained a number of health workers, but when you look for example the number of nurses that Botswana has produced by itself, for quite some time now the number real's explanation increased, and when you quickly look at the ratio, patient to nurse, it was really grown up quite a lot, although the ratio of patients to doctors is still lacking behind, but there the progress has quite been quite magnificent , but what is making us to feel it is still not enough, it is this disease spreading, if we were eh in a western area where we had for example cardiovascular diseases and metabolic diseases. Our nurses would not be feeling that much pressure that they are feeling now because of a lot of infectious diseases that we have, so my point is, the burden of disease that we have has overtaken all the effort that we have made to train more health workers. Part 1 already mentioned a mismatch between urban and villages, where a village here will really	

feel the pitch. The example is whenever a midwife is transferred out of Maun to a city, like it has been the case in the last two weeks; we are going to expect no midwife coming over here. They would take those junior nurses who have just finished from IHS and send them as a replacement for those very experienced nurses who have been taken to better positions in towns, that is not solving our problem its making it worse, because it is putting pressure on the few that's experienced to start teaching even those that have just come in. The the second thing that i think it's the reason why we are not performing well in this regard, is that, like my brother said here! We are training but we probably are training for other countries. Because we want to get the standard of the UK and bring it in the country training curriculum. The result is when we finish training as Batswana Doctors and look back at the salaries that we get in the country, we make a choice we say; let me work in Australia i earn better there. Now when we bring it down really down, today we are deregistering erode nurses. I hear they are being deregistered, those erode nurses who have been in the system, they know the system, they are working hard, they have been working hard, today we are deregistering them so they can't be employed in our health system, those are the people whom we can use as a skill mix in the country to make the impact at the primary health care level where we want to make the impact. In other countries they are training health care workers that are not exportable, and this is here what the country is not doing. We are training people who are really easily exportable, so once they finish the market for them is so huge that they can find themselves elsewhere very easily. Our suggestion would be let's keep those erode nurses), let's give them skills, it would reduce poverty, it would them a hand in to the health care system. The last that i want to talk is budget for health, budget for health in Botswana i happen to have looked at it, it is below what is recommended by WHO. I think WHO if my mind serves me well, the budget allocation for health if you want impact in your health system it has to be above 15 percent of your GDP, i think its 15 if am not mistaken, 15 or 14 percent.<br><br>Int: Is it of GDP or annual budget?	

PART 4: Of your…your annual budget. It has to allocate i think 15, if its low i think it should be around 15 or 18, but when you look at the budget allocated to health in our country here the last i looked it at it, eh I don't remember the figure but it quit below 15, so obviously with the disease burden, all the health needs we have we can only face the situation we are facing, thank you!

Part 5: And the other thing, to add on what he was saying. I know he is talking about, now even in the in the health care profession itself, you find that it's difficult, there is no real opportunities. So that as a nurse, at some you are now forced to choose between being an administrator or being a nurse. And then now you decide if i had become the administrator i might even go up in level, but if i choose just to be a nurse, i mean there there is is less good advancement in that area.

Int: Yes yes Part 7!

Part 7: Well i think, I don't think we are training enough, even the training is like focused in certain cadre. And this other who are trained like you take nurses for example, a lot of nurses are being trained and are expected to do everything. At the end of the day it's the nurses and they are out there and expected to do everything that would have been done by the pharmacy technician, the lab technician and everybody else, because these other cadres are not trained, they are very few in the country. And this nurse get overwhelmed, by being alone out there because these are people that are many and the tendencies to now continue train them on other thing to cater for the other people that have not been trained. And when it comes to like my brother is saying, progression, why can't we progress like, like if you are a midwife you continue to be a midwife. At some stage you become an administrator depleting now the specialities that… that taking care of the need, to have those nurses that have been have specialities, but as they go up the ladder they become something else, depleting now the speciality they were intended for.

Int: Part 8 were you going...want to close off this question ... (participant starts

talking)<br><br>Part 8 :Yah Madam Chair, Madam chair i think maybe there can be a bit of digression or eh…emphasizing, can you just repeat the question please!<br><br>Int; The question was do you think that are not enough, or not enough health care workers in Botswana? If they are not enough, why do you think this is so?<br><br>Part 3: Ya! Umm! because when you say health workers, i was thinking that eh… because the subject of this topic is primary health care, now when you talk about health care workers that could could also mean the type of health care worker you are talking about, eh… was under the opinion that this discussion we are trying to focus on health care and to me the issues of primary health care which my brother has pointed to the disease burden, consisting of things like tuberculosis HIV Malaria Malnutrition, sexual and you know Maternal death and so on. Most of these disease burden, they can actually be addressed by prevention. But i think the cadre which is being trained is too much concentrating on the curative medicine, so that even make it a serious more serious problem because it seems like the primary health component of health care is being depleted because of the cadre that are being trained for a setting which is not in the third world, that is why my brother said these people are easily exportable. Because i think you will agree with me that the disease burden is different from here and the west. But i think in Africa and the third world per say, our kind of problems which can be addressed at the primary level, primary health care level, i think the kind of<br><br>Cadre that is being trained is not being addressed, but most people are being found in the curative part of health care.<br><br>Int: So i think we have covered that, but basically we are saying we are not training enough. Even when we do train we are focusing only on one cadre, and even that one cadre may not be trained to address what they really should be and we somebody mentioned that we are not recruiting enough and we are not retaining them. So i think we will and that we will we will equi… equitably deploying them. Now, the next one is focusing mainly on primary health care. So	

do you think they are gaps, problems or issues related to health care workers for Primary Health or not, if they are gaps problems, what do you think are the most important gap issues of problems. I know this is a difficult question, but the things that are or not very clear. But the things we are talking about is, i will just give an example, for instance, Are they adequately trained for the job? Are they assigned to the jobs that they are adequately trained for? Is there a shortage of certain cadre more than others? Is there adequate support for the job, in terms of resources coordination and management? Are the conditions of service conducive or helpful and any others that you can think about? So these are the kind of issues that we would like this question is trying to find out. Whether those are are they are they issues issues are they problems that would make health workers for primary health care? Or affecting health workers in Primary Care. E e Part 3!<br><br>Part 6: Ee! I think they are issues because like you will find that some of those people are far somebody will be working in Shomela. The road is not good, they will be referring to Letsholathebe they will be referring here, going back they will find another patient another patient there they will come back. And in most cases you find that it would just be a nurse there alone, and in most instances when she is alone people come to the clinic when she comes back then she would find some people then she will have to refer again. And those instances we say ee! it's not conducive, ke gore the the... ,even somebody who would not be adequately qualified, you will find that at times its just a general nurse in a what, a health post, so if its a pregnant woman if they are any other complications she or he will not be able to deal with those.<br><br>Int: Any other issues? Part 9! In green.<br><br>Part 9: I think i wasn't raising my hand, but let me just make a comment, i think maybe the one thing which creates weak sports in e e our systems here in Botswana including the health, in that they you know we have a once fit all for most of our services. I think if certain areas, for example eh… big areas like eh… bo Ngamiland bo Kgalagadi eh… you know the policy makers, the real policy makers can at least propose service delivery models, which are suitable which are	

applicable to all those places. You know because you will find that the the health service eh… delivery model, which is used here in Ngamiland, it is the same one which is used in Kgatleng. E e but villages in Kgatleng or district health eh… areas in Kgatleng, they are they are small, so if so if I don't know maybe the government can come up with a flexible or area specific models of eh… giving services. Which in our department is killing us, such that you find that we are we are compared let's say eh… youth officers in Molepolole. They gonna say we are not performing cause the very same model of service delivery is the same model of service delivery which we should use here in Ngamiland, what else we have villages like Kwa bo Mabaleng bo Phuduhudu. Whereas bone their area radius is small and more often than not are accessible.<br><br>Int: any other issues? Ee Part 6<br><br>Part 6: Ee! i was talking eh...am talking about the conditions of service that they are not conducive. Cause in most cases you find that eh…tota people would just be giving basic training and there after they will be forgotten. So there will be no further development, it doesnt matter whether you are working at Shomelo, whether you are working at Gaborone but at least if people work in remote areas where you will given some incentives that would help. We are not only talking about monetary incentive but we are talking about other things like housing, you find that people are there they are just thrown in places like Maun without being given any accommodation. So really it's not conducive enough.<br><br>Int: ok please continue Part 1<br><br>Part 1: i think eh… one of the gaps is eh… the fact that, i have already alluded to this, but its like there is an emphasis on creative services.<br><br>Int: Are you a Public Health Specialist?<br><br>Part1 : Yes i am! (Quickly answers, (laughing)<br><br>That being the case, it's like Primary health care which is basically prevention, is looked at as second class service, you find that more resources are being pumped	

in the clinical services. Forgetting things start at the Primary Health Care level, so the gap that it's there is that. i think there is burying of head on the sand, in the sense we forgot where the problems are coming to. Things like transport, i think the transport facilities which are being allocated to to prevention efforts not only in Ngami but i think as a country as a whole is not as much. I think we need a paradigm shift, where it would come to light that the primary care the primary health care prevention should have as much if not more emphasis in the clinical services. So i think to me the gap here is the emphasis and respect which is accorded to Primary Health Care is minimum.<br><br>Int: Any other inputs? So what will the type of issues that I… that i wanted us to explore with, Are they assigned jobs that they are adequately trained to do, I know …i think… Part, Part 8....<br><br>Part 8: Part 8, we talked about it.<br><br>Int: Oh earlier on, we have also talked about certain cadre that are more eh… needed than others. And then we talked about adequate support to do the job, i know you alluded to it Part 2, maybe you can say a bit more than that.<br><br>Part 8: Emm first of all um, i want to say that all those factors that you have mentioned there i think they have a bearing on the service delivery to the health care workers who are found in this primary health care. All those factors that you mentioned there, firstly the further awy the health care facility is from the center. We need some kind of standardization, you know as country, to say when we talk of a health post we should be talking of a health post any health post that you go should have this kind of equipment this kind of personnel, this kind of structure, you know, so that everything is standard.<br><br>Secondly we need to make sure that apart from the structure standardization we should be talking about what kind of eh health personnel should be found in each ummh health facility, This would help now, this would go as a package in that health setup with body abled people who will manage conditions different conditions of patients in that setup with a good or sound knowledge of ehh what	

they been eh… what been eh… what what what as a country we would have established. When you send someone to a health post, you send someone to a clinic, this people should have some certain level of of training, you know when they go there they should be able to hold on to issues of that place. Ummh in terms of qualifications I mean, that empowering them with education like i talked earlier on. Then you are talking of you see, primary health care we are talking about it in the periphery most of the time. Of course when when you look at it from Part 1 point of view eh… even in town we are at it from a prevention point of view which is a wide of eh… but we still need to empower these people who are in the the education health education system.<br><br>Int: Sorry Part 8 are you talking about health care education assistants or you talking about something totally different? So that i follow what you are saying.<br><br>Part 8: Not assistants really, slightly more higher than the assistants. Health education officers you know eeh!<br><br>Int: You mean community based, community based people?<br><br>Part 8: Yes people who are in the community there, you know that can play a major role from prevention point of view, eh… but we are also saying that frustration in these people who are in this primary health care is a lot, because like it has been said, poor accommodation, someone who eh…doesn't have electricity. You only cook food what is going to eat now, because you cannot keep it for tomorrow. All those all those things have an effect on the productivity of an individual and you know how he is going to work and how he is going to eventually perform. So eh... he has talked about transport eh...there are quite a number of issuesout there. You know when you are faced with a client you are overwhelmed with work first of all you are few. And then you don't have support resources okay you don't have eh...you don't have a Sphygonometer you don't have this you don't have that...you know all these are frustrating eh...mmh...kind of things when you don't have and you cannot and they cannot help you carry out effectively your...your duty. So supporting...support eh...logistics is very very	

important in all this you know for us to have a well functional primary health eh...care system.<br><br>Int: Ee! Am going to point you... but one of the things...thank you Part 2 One of the things that eh... for the purposes of this study we we we have appreciated the definitions okay but the other thing is really is also the first point of contact with the health care system its also important so that its not rural its actually all of primary health care whether is in...<br><br>Part 4: Yes! In town or in the village...<br><br>Int: Yes! I see that there there i are...maybe i go this way that way and then Part 6.<br><br>Part 6; well, until and unless we understand what primary health care is like we started then will be talking of adequately prepared officers because how you are going to prepare the officers is what you expect them to do when you talk of primary health care so if you are going to train a nurse and you send them out there and that is primary health care because there is a health post out there and you are expected maybe to carry out all the primary health care activities kana....okay the communities will have access to this health post, its affordable to them. But the officer who is providing this services is not adequately prepared because hela from the training it was like you are prepared like everyone else to work in a referral hospital, to work anywhere like that like you were saying primary health care is all over is an entry point even in....<br><br>Int: In old Naledi...<br><br>Part 6: Mmh! Le ko Gaborone. Yaanong, and then it becomes also difficult when it comes to the support. What support are we going to give to these officers out there because here i am le nna yo ke sa itseng (even I don’t know) primary health care and am supposed to support that it becomes a bit difficult. Support will now be in terms of going there once maybe once a month just to...but you find that you need to really follow them up see what they are doing and the conditions of service like people are saying you are taken out in the rural area where there are no	

houses not even a house to rent because ko Shomela ga gona ntlo epe e o ka e rentisang (In Shomela there is no house that you can rent) there is no house there and you are a young somebody with kids. A kere of late people need their kids to go to certain schools. O batla hela a simolola from bonyenyane (You want them to start from) the crèche that you are taking them to, preschool and whatever. You are taken to a rural area o sa ntse o le monyenyane jalo e bile (while you are still young like that)...you are separated from your husband before you can even start a family.You are out there. E be go sena...e be o lebalwa Koo (You are forgotten there) in all aspect of life go raya gore (it means) you are cut off. E tshwanetse e nne gore (there has to be)...people will refuse to go to those areas e be go nna le (There will be some) congestion in districts tse e leng gore (where) people have access to these modern life.<br><br>Int: Thank you! You had your hand up...<br><br>Part 4: Ehe! No but she I think she said a lot of what i wanted to say, but i was saying even eh eh...to me support also goes to psycho social supportthat these eh...health care providers receive because i believe that as she is saying all you are saying all that Part 2 is alluding is is is depressing but there is no structure to even come in to check that this person is made to feel like a person. First of all there is no even the...there are no tablets, everyday you are seeing this people they are sick, it it it...as a human being as a human mind that depresses you and there not even the car to take this person, there are no tablets, the medication is not there, there is not even a car to take that person to the next clinic. There in not even a single person that you can talk to because o fitlhela e le gore even the tele-communication structures, the ICTs le tsone they are...are lacking if not minimal. So that i believe that when people now come out in town and they want to go there they will be hesitant. Le wena o le Part 2 trying to send somebody there you know gore hela even if you know gore there is...they need a nurse and a health care worker and is Tumelo and then you look among your staff its difficult, its difficult because thats when they start saying o na le favouritism. (Laughs).I mean those are things that we we we should also look at.	

Int: Thank you! Part 1<br><br>Part 1: (clears throat) Yah mmh...In terms of assigning proper cadres to primary health care i think we know that nurses are the fulcrum of primary care.<br><br>Part 5: (adds) E kare ke bone hela ba baitseng (it's like they are they are the only ones who know) primary health care<br><br>Part 1: And from what i know, initially the ministry used to ask nurses to chose whether they want to work in the primary health care or want to go to secondary health care and my experience has shown that in terms of skills, the nurses who are in primary health care have acquired enough skills to...to run most of the programs even those who come from....they are able to able to learn on the job if this one doesn't teach them but you find that within a short time they are able to do most of the things. My worry now is that we are...after relocation everything is being mixed at this we don't know how much skills we are losing from people who have been running programs very effectively. And so one thing i want to emphasize of having a minimal package like what Part 2 said i think this is we need to...people need to know that when you are at this level what things are you expected to do, what are the resources that you need at that level so that if if you are able to make target you know with what i have i can do one, two, three, four.<br><br>Int: Okay! Please and can you be the last one because i think some of these i think we will give them....<br><br>Part 8: Okay! I think one thing again should be considered for the primary health care system to work effectively. You know i think people in the health facility can give a testimony that home based care have really helped our country to manage HIV/AIDS and maybe its high time again to come with the...community based primary health care model whereby the...because nowadays we have a lot of you know...learned people university graduates from a lot of this villages. Let's say if possible they can be recruited and trained on the basics of primary health care eh like eh...Part 2 just said its like the emphasis is is more on the second stage. This stage is not emphasized a lot so if we could use eh...local people, local young	

people i think issues tse di tshwanang le (issues like) accommodation eh...will be resolved and issues of sustainability because you will be working the local people/community<br><br>Int: Okay! Thank you very much. Am eh...This is just a closing in these. Yesterday we had a meeting with the health care workers. One of them works in Makakung she is pregnant so she...she says she had to take a horse ride from Makakung to Kareng because she joined the ambulance in Kareng.(Laughs) So i just i mean just eh...this was just telling. Eh...Now is the problem of health care workers in rural areas? If yes why do you think it is so? Now i will just give some of the probes so that you...problems of health care workers in rural areas now my brother just said we should also talk about remote areas. We could include them there, and so we want...this is looking at things like eh...do the living conditions in those areas contribute to those problems if they are? Eh...how about remoteness you know all those things that you can think about but first of all i want we to answer...is there a problem but i think so far we have said there is a problem and so maybe now we can say why are there problems.(clears throat) Ee! Part 4<br><br>Part 2; could I break the ice?<br><br>Int: Yes!<br><br>Part 2: Eh...there is a problem, a serious one! I think we have been alluding to that...just to give you an example in this district here we are trying to drive the agenda of having a doctor in all bigger clinics. We managed to stick the doctors in Maun, we tried to put one in Shorobe, we put one in Makalamabedi, we put one in Sehitwa. They resigned i think less than six months down the line and i don't know if we are going to continue putting them there because each time we try it moves against ourselves. So there is a problem. The main problem that most of our people find there are not financial problems. The financial aspect of it is very less significant. The non financial part of that...that is saying the living conditions in those areas are really difficult. I remember one time i was paying a visit to some nurses who were working in Nyaunyau. You know Nyaunyau?	

Part 4: Mmm!

Part 2: Its far deep in the Okavango there. I drove 120 km off road to their place. When i reached there, i found them all drunk.

Int: Drunk?

Part 2: Yah! It was around...i reached the area around 5

Int; In the evening?

Part 2: It was 30 minutes after they knocked off. I found them totally drunk. They were lying on the floor in their houses. I asked them...i very interested I asked them. What has happened to you in this 30 minutes? They said Part 2 look at our area here. What can we do after 4:30? If it is not just to try and get something to get us high so that we can sleep. So it is so depressing that people can find refuge into alcohol into drugs in those areas. The other issue with that is…personally a person who finds himself in that area is even disconnected from internet, telephone there is no network. So he can’t even, say I can do online learning from here. Those things are making those areas very very difficult to work in. It’s only now that you see telephone coming to Mababe. Mababe is an area where you could find lions in the morning in the schoo…in the clinic yards. You can’t call for rescue, you just have to find a way tosurvive those lions. But now atleast there is Be mobile you can call 997 and say no am in danger here. So those are the conditions that our people are living with the other side.The other condition that most of the people especially when you go to Okavango areas, it is this crossing of rivers when you have to do your work. You have to do this outreaches then you cross rivers. More than once we found people stuck in the river. When they get out of there the tell you the only thing am thinking of is what can take me out of here. So those are the conditions that our people are living in and I think they are just very difficult and they are driving them away from our rural areas. I will rest my case there for now.`

Int: okay! Anyone else? Part 7

Part 7: Ya! I mean for me we...we also need to blame administrators and managers because sometimes when we say we set conditions for people to go to rural areas we say you will go there for two years. Yah! Then after two years someone doesn't get transferred. People don't get that as an incentive they will say i know when i go am going for ever.<br><br>Part 2: Yah<br><br>Part 7: So we should make it a point that when we say you are going for two years, by the time someone is going to...before two years he is receiving a letter that you are going to this place because people will know that within two years i will be moving but if you just let people know that there is this condition that you will be there for two years but its never applied in practical sense<br><br>Part 2: Exactly!<br><br>Int; And the other person then Part 2!<br><br>Part 5: Yes i think i wanted to say exactly that. Kana wa bona...what he said there is lack of training opportunities also. If if you are a fresh man and then all of a sudden you are thrown at Shorobe, Maun on its own it it doesn't have a lot of training opportunities. But you have to downside in to the country to be able to access this...most of the training facilities. But if you are going up to Shorobe and you are going to be stuck for...and you are a 27 year old and your your colleagues out there are maybe in Maun just for the sake of arguing we here they have the access to internet they can do online media assignments atleast. Online you know correspondent do all that you don't have that opportunity, it's like tota you are doomed. That's how it is for young people its…i know you are doomed. But this is a situation also, which is a win win. If i knew that okay, am going to that rural area just for two years. And then after two years, that is something that can you know psychologically you know tell me that okay, it's not that bad. It's only for two years, am going there and after two years am definitely out. You see? That would work, that would work	

Int: Okay! Yes Part 2 your hand was up

Part 4: Mmh! Eh...Just on top of what he has just said and alongside this posting really the...when this people comeback from such rural areas if there could be some incentives that is given to them like when you finish your two years, you come back we give to go and train at...you know that would be something to them. And also is more eh...other than the...just apart from what Part 4 said there, but i still believe that monetary...monetary part of it is very very important. This people who are in rural areas and eh...remote areas if there could be a deliberate move to say you people who are in the rural areas...remote areas you will be given...a little bit more cash despite the fact that we don't have many shops but someone will still be comforted to say i have a lot of money i can save. One time i will be able to go there, do ABCD. I think that's eh...that should also be considered.

Int; Is the cost of living higher? Are there...are there issues that may...of setting the cost of living anyway? Is that an issue?

Part4: Certainly!

Int: Yes!

Part 2: Eh...Dr F When you are in Maun, already in Maun, you pay your litre of fuel more than the one who is in Gaborone. You are aware of that don't you? Is not an incentive. When you are in Maun, for you to get to Gaborone, you spend more than the one who is staying in Palapye. So cost...living in these remote areas of the country it's by...by default expensive. Although what makes life expensive in Towns is window shopping. When you pass too much before that window you end up entering the shop and you end up spending. When you are here of course there are no windows to see then you have the impression that you are saving but you are not because the day you go to the town and see those windows after six months of not seeing them you will just you know you just eh...recuperate and spend beyond your your your budget. So I think staying in the rural areas is by default a disadvantage in terms of expenditure. Eh...If I could just answer that one. Can I just...

Int: Okay! Part 2 before we move on to the next one.<br><br>Part 4: Okay! Eh...Just to...just to argument...argument on what Part 4 has said. The Boarderative memorial hospital,There is Kgatleng DHMT vs. Ngami DHMT.<br><br>Part 7: Where i was born (jokes)<br><br>Part 4: Eh...it is terms of resources. Kgatleng DHMT if they are to refer a patient to Nyangagwe, to Marina it would be 1 hour, 2 hours go come. They will have their transport back and no cost involved for a nurse to stay there and...But us when we are referring a patient we will need a minimum of three days which means eh...we will spend more on referring one patient. And...and yet the resources that is being allocated to all this health facilities is the same. So in terms of costs actually there are a lot of differences there.<br><br>Int: Ee Part 10! am going to take you and then we move to the next one<br><br>Part 10: Although Part 4 mentioned the drinking part there are a lot of the manifestations of the hostile environment out there. People start abusing sick leaves, absenteeism because once they come to like if you are stay in Mababe you come to Maun or by luck you end up in Francistown. The tendency if you were off duties the tendency is to extend them to whatever. Until you are satisfied is what you what to do in town and if you get to Francistown you are going to get your sick leave there because there is nobody who can give you a sick leave. At the end of the off duties or leave there comes the sick leave because of the hostility of the environment where you don't talk to anybody like it has been mentioned. No electricity, no internet, no telephone nothing! You know that ounce you go there....<br><br>Part 4: (Adds) you are buried!<br><br>Part 10: Then you maximise when you go out of...<br><br>Part 2: (adds) you overdose!<br><br>Part 10: So... (Laughs)	

Part 6: You Know of recent, of recent we in different government departments have been eh... edged to implement an affirmative action policy and giving services to people who are not in the normal areas. So, unless and until you know the government makes it a policy, you know it should be on paper written in black and white and and and also for a government to propose that you know there should be a special dispensation for funding, for resourcing and for whatever which will ensure that services go to the people. You know the which Part 2 s stressing is is a very serious point and is a very serious issue because it cuts across all government departments like i said earlier that we the youth office in Maun are expected to deliver the same eh...as the office in Palapye or in Lobatse or in Mochudi. So they is no special dispensation you know looking at eh the the dynamics or the peculiarity of our area of...

Part 3: (Adds) Population!

Part 6: Which we are there is eh... our area of jurisdiction. So, and you will find that there are people, there are brains who are in that area who the government can solicit their input as to...you know how can we come up with a better model to service...You know another day i was chatting with another gentleman from another department. We were saying because we know that there are some villages which are in the delta, there are some villages which are ko bo Seronga you know all those areas which are around the Ngami area, why can’t ...let’s say the government buy a chopper or two, just a BX chopper like the pulled vehicles there, that chopper belongs to nobody. You know it just stands there. If let’s say the government department wants to the Jao flats or wherever, they just register. It’s Part 4 maybe and two other people, three other people from veterinary they just register. Eh if the register is full the chopper leaves maybe at every Friday or whatever you know so that eh...mobility and accessibility can be addressed. So the problem is that we have a problem of one size fit all eh eh eh way of doing things. So all these areas i was listening to dikgang (news) you know like di council di tsene all over he country. The chairman wa council ya Kgalagadi was saying which they cannot fill mo Kgalagadi but if you ask them why can’t you fill this

vacancies? It's the same problem tse e leng (that) gore we are we are deliberating on them here. But it's like there is no we on the part of mongwe (someone) to come up with solutions. Because i don't think this things which we are discussing here are new to somebody's ears.<br><br>Part 3: Am trying to be frank from...<br><br>Int; No Thank you very much will go on and so...having deliberated all these issues what do we think should be done for the lack of workers for primary health care in Botswana? What should be done? Ee Part 7!<br><br>Part 7: We said it eh...<br><br>Int: Ee we have already said it?<br><br>Part 7: Ee! We said it and i think all of us we have already said it. He says train for the local consumption.<br><br>Int: Okay<br><br>Part 7: Specifically for Botswana do not bring your...i don't know if i see the numbers that the write, the standards that they always write about. Don't train for that markets train for the local mid and local consumption. The other thing is, am going to just say what they have been saying already...<br><br>Int: Yes! Ee Part 7<br><br>Part 7: The incentives!<br><br>Int: Mmm!<br><br>Part 7: Eh we talked of incentives. There should be an incentive eh like eh you know incentives for this people who work in primary health care. Eh...the other thing that we talked about is eh...eh you know the support that they need that they...we need to have them supported both psychologically and the infrastructure, you know we also talked about you know mmm...recruitment and training even changing the structure of the whole system. Even moving from now the	

government as the provider because that is one thing that i am hearing from all these people who are talking, the government has turned to be the provider eh...undermining the skills of the local communities. The communities...that is why he is talking that we are looking more into the curative part of things but I think the communities if you went them with a problem and actually engage them in solving the problem not just the government skim like they say "mananeo a goromente" (government programs). Let us move from that mentality let us say communities take eh eh eh... a central role in the primary health care. Let us a nurse or whoever comes to provide that service be an an an assistant to come in to assist in the problem they are solving. I think we have talked about most of them but mmh!<br><br>Int: Ee Part 8!<br><br>Part 8: Can you remind me of the question again<br><br>Int: The question is what can be done about the lack of health care workers in primary health care in Botswana?<br><br>Part 8: Yah! I think i just want to add another point where...maybe to to consider the impact of a indigenous....indigenous knowledge of medicine because i think it has taken us through ages just to see how can eh...indigenous knowledge of medicine or primary care help in those areas.<br><br>Ink: okay! I think we will go...Part 1, Part 2, Part 4...<br><br>Part 1: I think i just want to highlight on what my brother has said here about the training. Eh...when I was trained, and i think most doctors will say that. There was very little emphasis on the community. You look at the person when he comes; look at the disease, hypertension or whatever and then pumping bullets and so on and so forth. But with an aspect of saying where is the person coming from. So i think, i think there is inadequacy of training so2 I think training should be tailored in the communities. The doctor should be exposed to the communities at an early age. I think when I was training myself there was a thing called community health	

I think just...maybe is it...a few weeks where just go to some rural area and then come back?<br><br>Part 2: (adds) for six weeks!<br><br>Part 1: Yah! For six weeks! And then come back. and even when you look at history taking, for social...social history there is just a come small line (laughs) but i think in the...the curriculum must change so that the the doctors, the nurses and the other health cadres are exposed to understand the community because there is power in the community.<br><br>Int: Thank you! Part 2<br><br>Part 4: Yah mmh! What i want to say is that first of all before we can think of what can be done what do we need to do? We need to look at...we need to carry out a need assessment and carrying out a need assessment we should be having it in our minds what do want this eh...this health structure primary health care structure to be. If we know what we...what we want it to be then we can carry out need assessment. If there is need to train a specific certain eh...cadre you know to try and give leverage in the system then we can now target you know, such area. Let's look at what we need then we can start training.<br><br>Int: Part 4! Thank you very much!<br><br>Part 2: Thanks a lot eh...eh chair for this. Eh without this diluting what have said, for me i think when you talk training. Let's look at the skill mix that we need for a health system for the...not for the global village. Let's look at which skill mix do we need. Like there was...like he just said...that need assessment i think we have already done. Botswana is a country that has enough data to know what the major problems are in the health system. And we know is maternal mortality, is infant mortality, we know is HIV, is know is Tuberculosis. We know it's becoming the metabolic and cardiovascular diseases now because we are in a transitional eh...you know...age. We are moving now from, we are having a mixture of the two. So the skill mix that we need, we need to have in mind what i said ealier, lets	

us focus on non exportable skills which will do the job for us, which we are going to incentivise in an adequate way. Such that someone even if he grows old being an auxiliary pharmacist he feels he have made his life. We are failing to have pharmacists in our clinic you know why? Because we want who deals with drugs to be that pharmacist trained in the UK model. Once he has trained that way we fail to pay him as the UK model pays their pharmacists. At the end of the day he moves to UK where we trained him so that he is paid adequately like the one. But we have an experience now; we have a guy in Tsau who is an auxiliary trained, specialised on dispensing of drugs. He is there he is really in love with his work. Let's train such things. Let's train...who have those erode nurses who are good in checking eyes, let us keep maintain them in the health system and let the eyes be treated. We have those erode nurses who know how to check a pregnant women whether she is ready to deliver or not. Let's train those cadres because we are not going to export them anywhere and they are going to be satisfied doing that job. The second thing is that let us put emphasis on the non financial incentives of our people. Let's give them good accommodation when they are in Nyaunyau. Let us give them communication, let us give them that psychological psycho-social support so that they feel at home while being there. I think when you address you know this country has been addressing the financial incentives every year until four years ago they stopped. But when they stopped four years ago, the brain drain, before they were addressing and the brain drain now, it's more less comparable. That means in other words financial incentives would not have much weight to retain somebody. If you give me a lot of money but at the end of the day you frustrate me in the way i am working i will move on. I will keep moving on. Some would even move to a lesser salary and better working conditions than bigger salaries with a lot of stress where you don't have things to use, where you are staying far...the other thing i want us to look into as policy makers is keeping husbands and wives together. In this country there is a policy i don't know whether it has been abolished, you would be with your husband or your partner where possible something like that. Now managers, they just stick on the where possible. So they can transfer a husband to Kgalagadi, wife to Chobe. Then again	

one of them will get frustrated and move on he say me i want to be with her or with him. Lets look into those little policies that are frustrating our people and clear them so that people can be satisfied where they are.<br><br>Int: Thank you! Eh... What about task shifting? Is it something we should be thinking about? It’s just a buzz way cling cling with Human resource. Task shifting is where eh eh... you look at you know we talked about..Someone talked about skill mix but we look at the job that needs to be done and we say well, this bit of the job say for instance am just giving for an example that am doing as a nurse really can be done by so and so who has had 18 months of training or six months of training just doing this thing only. And...and then this bit kana am doing right now really should be done by somebody else who has had six months of training and eh...usually is downwards is not upwards but just really what am talking about. Is it something that is worth thinking about? Ee Part 1... I know you are alluded to it…<br><br>Part 1: I think i...i think eh...task…eh…task shifting is very important because when you task shift actually you...you do involve the community at the end of the day. I think the problem we have in this country is that task shifting is being avoided because of the issue of registration. You find that people can do certain things but they say no! You are not registered with the BHPC. So you cannot do these things. So i think that is a very important thing. People can do the job, there will be supervision, i think is something we should do. I think…the problem we are having maybe in this country and next door and next door is that we want to copy so much of whats happening out there in the west where even…even to put a cadular you need to have a certificate for it. Me when I went there when I went to the UK, I was looking for a part time job. Just to take blood…says No! We must have proffesionals trained in it. So what am trying to say is that this thing of too much doing what is done in the west will not work for us we must see what can work out for us so that even the community can participate and we can do this thing so task shifting should be a good part of a community situation.<br><br>Int: Its Part 6 otherwise we move on.	

Part 6: Yes, it's just an addition to what he is saying. You know in most African countries in terms of consulting patients we have lack of assistance. This on who… who, one task that is done by these fellows who are trained just in consulting patients like you are a doctor your job is just to see patients, something that can easily be done here. I know in other countries also they are training, straight from school if you want to practice as a midwife you don't do the basic nursing, you are just trained one year six months just to do midwifery so that you are just there to do that unless what can we say we train people just for local use so that those ones cannot be registered anywhere else. You are just there to do to do this kind of job in your country.<br><br>Int: okay anybody to say something<br><br>Part 4: Task shifting is a very very critical area but if you continue to have a problem where its not clear as to who is doing what people are trained, okay I have been trained as a nurse and then you look at what else can be done by somebody else. Then I continue to be asked to do the same things and this officer. And this officer wants to progress just like the registered nurse because the question is what is that am doing which is different from what you are doing. That is where the issue of enrolled nurses come, you could distinguish between an enrolled nurse and a general nurse they were doing the same thing basically. And now if you continue to have that then its like you have not even shifted anything you have people who are there doing the same thing, increasing the work force, doing repeating what other people are doing. So until and unless again we make it very clear gore ha re shifter this task what are we shifting and this officer now will no longer be doing this will be doing what.<br><br>Int: Yah, okay thank you. Very interesting that all the discussion and we are talking about another cadre. Where South Africa is training a new cadre that will only work in South Africa. There will be…<br><br>Part 3: the way forwards, the only way forward.<br><br>Int: Okay. Now in your experience what solutions or interventions that have	

already been tried and have they worked? In addressing our issue problem, what interventions what strategies have already been tried in the country by Botswana to try and address this problem. I know Part 4 alluded to monetary incentives, what else have been tried?

Part 2: Scares skill, scare skill

Int: Scares skills, yeah I guess it is monetary.

Part 9: Go ne ga lekiwa le ka tsone di (We could try) health care auxiliary to bring them on board to try to do away with certain duties that were done by the nurses. But I don’t know what happened because that one never even took off the ground. They ended up doing even other things being clerks, jaaka gotwe the one are specialising in dispensing drugs, but that was an attempt to say lets shift certain tasks from the nurses but ha go introduciwa (if we could introduce) the axillary... ga tweng? Gatwe ba bidiwa eng?(what are they called?

Part 5: Health care axillary

Part 7: But the problem there, there was there was the there was no progression there were justtold will be, will be axillary that was the en and most of these were young people you know with ambition, they were just told. That programme should have been tailored in such a way that from here if we identify you are doing so well in the in the lab yah we can send you to school but most them there were just there like their contracts end and everyone wants to run away.

Part 4: That training was not even clear actually. What you can be trained for, because for those ones who came, the first batch. They could not even check vital signs, so we did not know what they were trained on.

Int: To health care axillaries. Yes Part 7…….

Part 6: Akere like eh... we were into eh... the the issue of what …… manpower into the.. I think at the beginning there was some process because like people knew that if you going to primary health care you are going to be housed but as

clinics grew ah, and the number of employees increased that was no longer there and now is the case that even when you go for primary health care you are not going to be housed. But the beginning, you will be housed; you will have furniture in that house. So there was no need for you to move with any furniture, it will be just your bags that you are going to be moving with. But nowadays there is no longer that housing, there is no longer that furniture because now we government policy that local, no furniturethat it should be given to locals. Then it becomes so difficult because people, when people move there, they know that there things are going to be damaged by the length of distance that they are going to travel from where they are to to other primary health care facilities

Int: Yes any other...

Part 6: Yah, let me just add other example that that of aahh, I think one intervention that was brought in was to bring in other cadres. The lay counsellors for PMTCT. (Others agree). These were brought on board and actually when the programme started I remember I was in Marina that time and those lay counsellors as we are talking now 10 years they are still on the same level.

Int: Ohh really?

Part 6: Yes, 10 years they are still on the same level even to make it worse they are the the they are I think now…I think there was still n issue of whether they should be temporary, or contract or PP. So its very discouraging so all that intervention was to bring in some… that will help and I must say that once the lay counsellor come in they picked up rate for PMTCT went up. But then this people now they are discouraged and I think they no life…

PT 4: mmm..., exactly

Int: Okay Part 4 Part 3: he touched on what I wanted to say, the lay counsellor issue its an example of the skill mix that I was talking about with a locally trained cadre that cannot be exported, I think it's a model (Murmuring)

Int: It's cold

Part 3: So I was saying that eeehhh a model that we can inspire on while we skill mix. One other thing that works is it what Part 4 refer to with the small voice it is the scare skill allowance. I remember before the scare skill allowance there was a tsunami of doctors moving on eh… it was a real big tsunami people moving on to South Africa, then came a scare skill allowance which actually almost put a s a block on that. But you can realise that its only slowed the movement, it only slowed the way, eh… the way whenever you insist on financial what you will do you will just slow the wave and once another wave comes, when inflation goes then the other wave come and sweep again. So it works but its not real the magic bullet, the magic bullet that we could embark on will be issue like just accommodation. If the government could embark on the massive campaign and say lets accommodate our people like it working in Okavango now. In Okavango the office of the president sponsored the construction of.., I think 48 houses for health care workers. Today we have a doctor in Seronga, he has been there for the past 4 years he hasn't moved, we have a doctor in Etsha 6 he has been there for the past 3 years he hasn't moved. And we have we moved from 2 doctors in Gumare to 5 now and they are there. Just by putting on decent housing, when you to houses there you can't compare them to the one am staying in in Maun. It is modern structure, when you talk those colleagues there you ask them how do you feel he says am better of here. Am paid P20 a month stay in a house like in Phakalane in Gaborone. You see those are the things that the government could embark on and make a real good difference in a short period of time.<br><br>Int: Okay. Anything else. Umm do PBRS help, akere it was eeehh, it's a recent incentive? Or was it more looking just at performance and not at<br><br>Part 4: It was supposed to be renewed<br><br>Part 1: All part of it didn't work.<br><br>Int: Ohh it didn't work.<br><br>Part 1: the other part of it (murmuring and coughing)	

Int: they… there is other recent incentive, or well in strategy of moving primary care, clinic from local government to ministry of health. Just mentioning them akere this are recent things that have happened and setting up districts health management teams although i understand its not they not really fully set yet but they are being set. Now we have a medical school and and faculty in rural areas, though this is new also so just thinking of all the things that have happened that may or could potential have an impact<br><br>Part 2: Yah I think I think like eh... I think the... the... the introduction of of medical schools in rural areas I think its an important intervention and I think it will go a long way in the sense that you know the people who are working in this places like the people who are non-academic working in this place I think I think they will feel they will feel encouraged to remain in this place. Because most of this people like in this country once you have joined work… work like neutral area you will progress academically. When you cannot go for training and so on and so fourth but I think with the medical schools coming I think it brings in an academic environment which gives some hope to the people who are working in those areas. So that from here they can even make some attachments, some networks and they can can carry on and even their… their level of performance will increase. So I think setting up of medical school in rural areas is a very important intervention. With medical schools come many infrastructures and many facilities so I think its an important intervention.<br><br>Int: Nobody is speaking to the…Yah Part 3<br><br>Part 3: eh… the… the relocation, I think the the overall objective was quite good to bring the local eh… primary health to ministry of health but now two years down the line we can see that one we have lost in term of budgetary resources. I think the under the local government clinic have their own budget which is not adding up now. In terms of transport under local government if one one programme problem of transport it it would be sources for under sections within the local government so it that one is not applicable now if you the HMD here you have one job just one there. Also ummh a lot of people have lost the they are going	

the accommodation since things have been lost out by eh… by change of ministry. In terms of the HMDs akere it's a it's a very good idea you need to district management team to be running within the districts where you are have authority to make decisions, the implementation hasn't been haven't been so good. Umm guidelines and, they have not been so they are ummh no they will tell you gore we are going to afford to do this but its taking for too long. We are we are not functioning as DHM team should function<br><br>Int: Anything else<br><br>Part 6: Ee! Nna on that one?<br><br>Int: Ee Part 6!<br><br>Part 6: I think I will share a centime because it goes back to what we have been saying. The the appreciation of local the communities in in health care provision. I think what happened is now we have just you know centralised it, make it BX and even worse because at local government atleast it belonged to the people. But and it was the responsibility of those communities of course they are any issues. But when we grew up it was the responsibility of the communities to build the houses for nurses to even provide for them and interact with them. Go ne go na le bommaboitekanelo, I don't know what they are called in English<br><br>Part 5: FWEs<br><br>Part 7: Mma…<br><br>Part 6: FWEs. They were community members, you see. Now that function ya e e thata (its difficult) to do in the central government structure. But it be it was easily fi... fitting mo (in) the local government structure<br><br>Int: Anything, anybody<br><br>Part 3: Yah maybe he has touched a little bit on the other one when he say it was a noble idea to one have authority over all matters. But like Part 6 said I think somebody just thought of the policy aspect of that but didn't think about the	

operational…the operazation of the policy and or somebody thought it was that very easy. And I think that has cost us quite a lot, all health indicators in the country are have gone down all of a sudden. The issue has been that lack of initial focus to see to analyse the rest of the decision of policy makers. Now to come back to what he said centralisation is one of the things that we are seeing now which are total against primary health care. The way we operate now in the current set up it is highly centralised. You may have as a decision maker at the district level, you may have a problem at hand and you want to address it like today but then when you start addressing it you have to communicate with an office in the ministry of health then that office tells you that your money is here but you don't have the code. It's a language that I am learning now; they will tell you the money is in your vote, the vote is with us here now we haven't given you the code. Then you tell them can I have it by four, they say no the code you need to talk to the administrator, then the administrator write to somebody then that somebody request for authority. You know that chain (red tape) just spins you on the seat and you start watch you start watching and say isn't this a problem I can address real. So I think when they were thinking of bringing the primary health care on board they didn't see that primary health care was a was a gadget a system that was in place to address problems as they were arising. Now it takes a bit longer and the community has has got the pinch of it and I was telling Part 2 here that it was going to be interesting on this panel here to have brought those representative of the community like the councillors<br><br>Int: They said they were going to come, but I guess the borne fire is…<br><br>Part 5: the borne fire is on. So those ones tell you the other view of it from the community side as here we are telling you about the code, accessibility of the funds, transport problem. Them, they will tell you the feedback of the other side. They are also the policy makers they would have told you a bit longer.<br><br>Int : Okay, thank you very much, we are almost closing now. Now I know we have already said that, but what do you think will make the biggest difference in improving primary health care? Or you think everything we have said	

Part3: What the question, what

Int: What will make the biggest in the primary health care? Part 2, Part2 then Part 8

Part 2: Oh real uuhhm all that we have deliberated on here would make a big difference if they are to if this things are to be implemented. But above all eehh we need a sound financial muscle to be able to implement all this, if we have sound financial muscle then anything that we are dreaming, you the best things that we are dreaming about then we can realise it.

Int: Ee, ee Part 8

Part 8: Just one short sentence. Political will.

Int: political will?

Part 8: Am thinking like, I think DHMTs should be given authority to manage their affairs. Like when clinical family health care was at at local government it was being dealt with at local level. (Exactly). So we need to have that authority to be able to manage our affairs (absolutely).

Int: Thank you! Now, building effective primary care team has been suggested as a potential intervention to improve primary care. Building effective primary care team has been suggested as a potential intervention to improve primary care. What is your understanding of an effective primary care team? Ee Part 2

Part 2: I think is a is a team which is which is Eh...resul... result oriented. That is when it is eh... given a mandate to serve the community with clear deliverables it will do exactly that to atleast more than 90 % level.

Int: Yes what do others say? Ee Part 5

Part 5: Even the the the the consti... the constitution of the team you know you must appreciate that when you say provision of health care just like in any setting they will need support structures and the actual technicians who would actual

carry out the service provision. I think what we have what what would happen that's why now people are crying about the DHMT. That was the the idea to have a team that can have views from eh... 360 degrees view from a on on service provision. So I think we we need to to have that in the team.<br><br>Int: Okay! Ee what do others say? Its it's eh... primary health care what will it...yes Part 7<br><br>Part 7: Eh... an effective PHC for me would be a PHC that has all the skills all the means to address the disease burden of a given setting that would be effective PHC.<br><br>Int: Okay!<br><br>Part 7: and when I talk about that am looking at it as a team that would have a vision of closing the tap rather than mopping the floor. Umm I don't if I would love to expand on that but I look at a team that has got those skills that are more into preventing the problem rather than solving the crises. Am looking at a team that is financial in charge of itself not the team that is requesting for codes today and tomorrow and am looking at a team that is fully supported by the leadership, that would make an effective primary health care team.<br><br>Int: Who should be the members of that team? Part 2 you can answer this maybe even start to say who should be in the...<br><br>Part 5: Members of the team<br><br>INT: Yes<br><br>Part 5: Mmhh! Members of the team would need a health practitioner, I mean in a…<br><br>Int: maybe we can say them out in<br><br>Part 5: Gen general practitioner, doctors<br><br>Int: Okay	

Part 5: Family.., like now we have embarked on the family medicine on the country.<br><br>Int: Okay<br><br>Part 5: Family medicine specialist would be an ideal person because he would be he would be having an integrated kind of knowledge in all eh...mmh the fields. You have eh... would be approaching a collusion or would be looking at his area in a holistic way. Would need also a a the nursing cadres, nursing cadres, a nursing cadre as who have a that a nursing knowledge that can eh...mmh... be used effectively at the level of the community and along side the other cadres. Eh..., definitely will need eh... if we can have a cadre that can be assisting the nursing cadres, nurse assistant eh... medical assistant eh... those who make the actual eh... you know technical group that would be able to deliver the service to the to the community or to the clan. And then of course will need the support staff eh... support staff in term of eh... the eh... administration, in terms of ummh from social point of view counsellors, will need the the cleaners and all those.<br><br>Int: Okay! Ee Part 8<br><br>Part 8: Nna<br><br>Int: Ee!<br><br>Part 4: Nnyaa ke ne ke ntse ke nne key taleletsa ke re bo (I just wanted to add that) lab and others, bo lab pharmacy and all support staff<br><br>Int: Maybe to extend that on that, should we have this team at each, clinic at each health post, or what what should bas where should that team function? And should there be how...Should there be somebody there who is in contact with the community? Am just throwing out ...Ee Part 9…….<br><br>Part 9 : Ke gore at first we have to have levels of care and it means that the first level it just has to be community based care. In that thing it means that, in that level it means that we have to be having people who will be educating the	

community like may be the he health education eh... cadre and then the second level now it means that it has to be primary health care. In the primary health care it means that we have to have levels like we have a clinic without, we have a health post so the the people who will be in the health post are not suppose be like eh... those who will be in the clinic or in the clinic with maternity. So we have to have all these different levels of care with eh... with staffing that is different<br><br>Int: So let's talking about health post for instance, how would you see a team in a health post?<br><br>Part 9: Mmm that<br><br>Int: ee Part 9<br><br>Part 4: Health post would of course there is going to be e nurse who is in charge of the health post, and you have the ee! mmh look here and would also have people from the community. You can have eh... representative from the community itself to in that team.<br><br>Int: what should be the skill mix….were your handup<br><br>Part 3: Am so am not sure if I will take you a little back, but I was thinking though we love a mix of these cadre in a PHC team looking at our disease burden, we need a nurse for example who. If you take a nurse for example, this person has to have a number of skills to feature effectively, to make that effective PHC team. She has to be somebody who understand HIV in our setting, she has to be somebody who understand how to deal with eehh little baby, paediatric, just to have some degree of paediatric skills and of course of general medicine as well a little bit on that. But most importantly because of our current maternal mortality, a primary health care team should have atleast a nurse who who is trained on maternal health, absolutely so that we can address the disease burden. Uumm I think we mentioned everybody except this, counsellor, we need a counsellor in that primary health care team, we need a health educator Part 5 has mentioned but also we somebody with the skills of pharmacy so that the nurse is not the one	

having to do everything. Need atleast somebody who deals with drugs, dealing drugs its not only dishing them out but its also to know how to control a stock, to prevent expiry on the shelves, so that you the drugs in and out of the place before the expiry time. So we need somebody trained on those skills<br><br>Part 6: Are you talking about the health post?<br><br>Part 3: Even at the health post level, we need also a driver. A driver is also part of that team according to me.<br><br>Int: Okay…<br><br>Part 3: And I think we don't put him there because WHO doesn't consider him as a health care worker but he is a very key person in the whole functionality of this. Now where to place them, of course they are...He said we might have a problem to place a doctor in the health post. I think we eehh already been moving to that direction of saying why don't we create some kind of clusters, a cluster that we say you are in this radius so we place a doctor here, we place a midwife and accessibility to those people will not be challenge. If we approach it that way it is achievable.<br><br>Int: Okay!<br><br>Part 3: Rather than putting a doctor in Mababe<br><br>Int: So in your opinion is it something that can be a possible intervention to explore or is it...<br><br>Part 3: Yes, very very possible<br><br>Int: Now we are going to ask the last question then we are going to end. One of the thing, I don't many of you are familiar with eh... document that is called the integrated health services management plan 2010-2020 Botswana health. Okay one of the things one of the pillars that it list there it's... its ethics. Now we are aware that umm... sometimes when when there is shortage of man you know human resources shortages and this working always in an ethical way may be	

quite a challenge. Whether its resource allocation, trust whatever. Now as a policy makers now can you tell us of any ethical of morally challenging issues that you tackle in you function as a policy maker under resource constrain that you you find, especially human resource constrain. General its some that the ministry of he.. if you read it actual talks about you know how as ethics it affects a lot of how things would be done how the resource will be allocated, how the human resource would be managed and the so does that is that a challenge for you as a policy maker because of the constraints of of that you have to work under. And if... if possible with examples that are safe.<br><br>Part 3: May I could the break the ice on that.<br><br>Int: mmhh<br><br>Part 7: yah yah! I think the lack of human resource is giving as managers' very tough time. For example, for a long time we have had in a health post one nurse, one drive, one health educational system, one cleaner, so it's a one one one team. Now we sometime get to a point where this nurse have worked for a year and needs to go on leave. So we can't refuse for him to go on leave but on the same time we don't have anybody to replace him in that place. It will be unethical for us to say okay lets leave that population to their own fate, because it happens we take this unethical decision to say okay go on leave we shall see what we do with those guys. What happen behind that nurse is eh...go for his leave will be maybe a baby who came in the nights, check this health educational assistant believing that since he has been working closer to the nurse he may be able to tell one or two things to do.And this health educational assistant also says all I can do is to call the drive so that we drive you to the nearest facility, but during that travelling the baby dies. So who kills that baby eeh its not it's not easy to say its me because am the one who allowed the nurse to go on leave. It's not easy to say it's the government because the government didn't give me enough nurses to make two, so it becomes a very confused picture. So in the face of that we as manager as policy makers we find ourselves in tight conditions. Sometimes we even ask eh... a health workers a clinic A to also cater for clinic B which at 30 km or 40 km from where they work.	

We know it puts work load pressure on this one, which is unethical in terms of the human resource management you don't put your too much stress and expect this officer to produce the quality work but we now trample on to quality to deliver quantity. So because of this constraint sometime we twist things around and it makes us feel also bad at the end of the day. I will stop there for now.<br><br>Int: Anyone else wants to say something this challenges.<br><br>Part 10: I think, I don't know maybe the other people have got more experience on this but recently we just realised that people are being asked like nurses to work after hours which is dangerous. So that people can just knock at their door go to the clinic and they can get attacked, there is no security. Like I think like I think I say I do not qualify so much on this but I think I think its its unethical to expect a nurse to provide service when they is no security.<br><br>Int: Anything else, the last one to say.<br><br>Part 5: Just like Part 6 has argued that its one of those things that are they are there like eh... like we have a gazetted villages like Somelo, Bomababe their population their people there, population is there. Then you find that their the the clinic were established there because of the population that is there in that village. Mean that they are even women there who eh... maybe expectant and can deliver but it means that we intrust people who have no midwifery qualifications to those to those communities by placing them there because tota if we had means of putting midwives will put midwives there but we don't put midwives there because we don we don we do not have enough midwives. So it means that we saying to them you can just assist with deliveries there if you can. (Very unethical). I think it's a it's a it's a very big dilemma.<br><br>Int: Okay! Is there anything else, is the bag empty? (People laugh and murmur). Okay no! Thank you very much. Now in conclusion is there anything else that you wish we have touched on that we haven't? Yah, no that's fine now we are very grateful that you came I am sorry that it has taken this long. But we we appreciate a lot for the information, and the input. We are doing it as I said in mahalapye and	

Gaborone, and in Gaborone we obviously dealing it with the new deputy PS clinical services eh... the people at the ministry and we will see how we can get local government involved and so hopefully this will come up with something that as a country we can look at and we can say now this is the evidence that we have for now, what can we do do with it? So now we are going to do another round once we have collected everything and analysed everything we will hopefully call you again towards the end of the year to say okay now this is what we gathered, this is what you were saying and what we do with it in terms of an intervention. So so thats real what I want to say, we the the supper provided, oh...im sorry it's a bit late there but please enjoy your supper and thank you for coming. Now I wonder do could do people want to pose for a picture, I just mean we got a photo a camera here so if if don't mind (murmuring and laughing).<br><br>**...The End...**	
