# Supplementary material for: Stakeholders’ Perceptions on Shortage of Healthcare Workers in Primary Healthcare in Botswana: Focus Group Discussions
Source: PLoS One. 2015 Aug 18;10(8):e0135846. doi: 10.1371/journal.pone.0135846 (PMC4540466; doi:10.1371/journal.pone.0135846)
Supplement: S6 Text — (PDF) [file pone.0135846.s006.pdf]

HURAPRIM PROJECT

Participant ID: Gaborone Policy Makers Focus Group
Date: 12th April 2012
Interviewer: Dr N
Interview Duration: 3hrs.23min.39sec
Audio File Name: Policy Makers Group

INTRODUCTION

INT: What is your understanding of Primary Healthcare?

P1: is it in terms of theALMAATA?Declaration or just primary health care?

INT: just primary health care. I mean... if for instance, because we are talking about human resources for primary health care, so in your opinion what should it be?

P1: I think primary health care, ke ray for this kind of discussion that would be the first point of contact where patients are seen at first point of contact.

INT: yah, anybody…

P2: err to me I... I... I will put it in terms of the way our health facilities are distributed like primary hospitals and clinics and err gatwe di bidiwa eng diiii...

INT: health posts...

P2: health post... hmm and outreach services like di-mobile talks.

INT: anybody wants to add anything?

P3: well, I'm not from the health sector, I would understand it would mean the basic umm… err services, actually the basic knowledge information that people have in regard with health services what are the information regarding the type illnesses that could be around and where they can access services in terms of responding to such illnesses in terms of health matters.

P1: gape ke re is it primary health care or is it primary health care services? So that we can…

INT: we… because we are talking of human resources I think we should extend it to service.
P4: ok. I think as P3 has said, primary health care or primary health care services is the health care service that we provide at a basic level, at err the periphery or where most of our people are unlike in the cities, in urban areas ko… ko… ko dikgaolong tota. That's my understanding tota.

INT: so in your opinion, that should exclude say Extension 2.

P4: not necessarily, ok ke buile ka dikgaolo, not necessarily, like I said is the basic health care services that are provided to… to… to Batswana.

INT: ok, so are we happy with that?

P4: hmm…
P1: we are happy with that.

INT: ok… now…

P1: ke gore what P4 is saying, it should be also explained, gore kana even if… I mean a city like Gaborone but then we know gore the first point of contact in Gaborone should be those health facilities tse re re… we regard as clinics, health posts, like we've health posts ko Tlokweng and Mogoditshane so those are…

P2: including home visits which I think are being neglected at the moment because of your…

INT: actually that was my next question gore primary health care only end at the clinic, how about the community?

P2: even the community.
P1: including the community.

INT: ok that's good; because we are talking about human resources I think it's important to know the parameters because then when we think about human resources we will think where… from where to what end are we thinking of so that we... Ok, now studies have showed, as I said we've just completed our bench research and we have seen that there is a shortage of healthcare workers in primary health care especially in rural areas and obviously that's what we have seen but in your opinion do think that they are enough or not enough health care workers in Botswana. At least if they are not enough why do you think this is so?

P4: err health care workers in Botswana are not enough and to me I think they are not enough because one, the training institutions for health err health programs are not able to produce the required numbers to cater for the whole country, secondly the institutions like bone bo-HIS only train up to certain level for instance diploma. They are not able to train beyond diploma. Thirdly, I think the other issue is the welfare… it's related to welfare issues gore in the periphery where I live, ke go ne ko e leng gore primary care teng tota-tota. Ga gona dilo tse di tshwanang le accommodation so people are normally not willng to go to rural areas because of this accommodation. There are no incentives that would encourage people to go and serve in the rural areas. So nna to me we have serious serious shortage. The other issues, I think is the issue ya gone fela gore the pay levels within Botswana compared to other countries like Namibia, most of our healthcare workers currently ba movela to Namibia because we are to… they have picked the pay scales…

P1: ya…

INT: anyone else?

P1: ya I think, akere the question here was do think there are enough healthcare workers and …

INT: and why…

P1: ke gore to support…

INT: yah to support…

P1: I don't think there are enough health care workers in the country even though… especially

when I look on the specialized cadres, they are not enough but looking at the output from the IHS from the basic training I think that one is such a shame. The only challenge here is that IHSs they don't give enough err slots for basic training and from my understanding it is because they don't have also enough teachers to actually train in terms of basic… post basic training. That is my first one. Secondly, the… where I think there are… the healthcare workers they are not enough is because when you look at the establishment for each facility, the establishment does not actually specify the… the specialized cadres. It just says x-number of nurses in a certain facility, so meaning that what would happen is that when you actually send people to work in these areas I'm talking in terms of nurses, they will be looking at a certain number of nurse at day-1, day-2, day-3 up to the lowest level. So… so it means that as long as the ministry feels that they have satisfied the level of establishment in that facility it is okay. But on the ground you find that this shortage is created by the number of specialized nurses who should be there because there will be so many nurses without any post basic qualifications. So from the ministry point of view, it means that the establishment is fulfilled but it is fulfilled by the wrong cadre because now these people they are not specialized. But if the establishment would actually say from the hundred nurses it means that we need this number of psychiatric nurses, this number of midwives, this number of …. Nurses. I think it would be better so that the shortage is created again by the failure of establishment to actually say specify gore this facility operating at this level should have x-number of specialized nurses in this… in this area. So the… the reason why we don't have sufficient staff especially in the rural areas I think P4 has elaborated a bit on those one, I've worked in the remote area, hospitals. So one of the… of the greatest challenge that we… we were seeing was the separate… was the separation of the spouses. If really my husband is 200… even 200km… even 100km from where I am based it is a challenge to because now I have to be visiting home ever so oftenly and he has to visiting home ever so oftenly. So… especially with the young ones, they cannot tolerate that. The older ones like myself I was there when I was in my early 40s I did not mind but the younger generation they can't cope with that kind of separation. So this is why… this is why in the remote areas we don't have sufficient number of healthcare workers; because of separation of spouses. The… the second reason why in the remote area facilities we have shortage of human resources, it's because there are no incentives. Previously, We used to have this something, rural something-something…

P2: RASA…

P1: ee, so even when people were there, but they were saying at least there is something that I'm getting. But now with the elimination of this RASA people are no longer interested in working in the rural area facilities because also the… the remotest areas, you know we were getting much more than the least remote areas but the practice now is that, because when we were enquiring go ne gotwe once we've got infrastructure like roads, go nna developments like di-shop and all those things it means that the RASA is going to be withdrawn. But looking at it, even from the previous experience when that RASA was there people were complaining but you know at the same time because they were incentified by the RASA, they were complain but also saying that at least I'm… I'm… I'm being paid for being away from my family. So I guess… this is another challenge that we are having that is really causing people to not want to work in the rural area facilities. The third reason that I had established is that, people who are placed in the rural facilities tend to be forgotten, this is what they would say; gore I have been here because when you look at the ministry of health trans… trans… gate transfer guidelines, it says an individual should serve in an areas for at least 3 years.

INT: at most or at least?

P1: at least 3 years.

INT: so it could be 3 years or more?

P1: yes. So when you reach that policy, that guideline as an individual and you are in the remote area you want this at least to be really at maximum because there are so many things that are happening out there. You are there and the family is somewhere else, so in your mind even when they complain, they would be writing that I've served for 3 the years so why you not transferring me out. So if there was enough human resourceto actually move people around like that so that when you are working the remote area facility after 3 years you are swapped with someone who worked in the urban or city or peri-urban area. So maybe it would actually work because really 3 years is just too much you are tolerating actually, for you to be working out there for 3 years it means you've really tolerated. So if you've got to work beyond 3 years it becomes the biggest challenge. So I think this… this… are some of the reasons why I said people not wanting to go and work in the remote facilities. The fourth one is lack of abilities, the time when I was in Rakops, where there are no shops; so for me just to buy just an apple I have to travel 135km to Orapa, to go just… to and buy and an apple. And you can imagine, kana go raya gore (*it means*) I'm using my car, so it means that I'm using fuel to go to Orapa and come back, just to buy an apple because some other facilities they are not there in the remote area… remote areas. So it becomes very expensive, really for people who are working out there to get just basic… basic things. So I think this is what I would say concerning the challenges.<br><br>INT: I think P3 is itching to say something.<br><br>P3: umm… ok, I haven't been in Gaborone for too long. I have been to… in places like Ngamiland and Kgalagadi. I want to belive like the rest that umm… there are shortages tsa (*of*) health workers. My… my thinking is we are not policing enough. The institutions that we have are not policing enough to support our villages. Particularly with the developments or establishments of settlements in the districts. Many dis… many villages in the remote areasthey started as cattle posts, err some small settlement and they have now developed or recognized as villages. The expectation is we probably should have institutions that will support the growth of the population or the villages in Botswana. Umm… unfortunately it doesn't seem like our policy in terms of the umm… number of people we wish to produce, the number nurses or the enrollment of nurses err doesn't seem to cope with the increase of population and increase in villages and particularly in remote areas. The other thing I had suggested to… at some of the meetings ke the provision of services in terms of what we normally call "we take service to the people", umm… the mobile health service. The manner in which they operate, it is like taking one person there at a particular date to serve that parti… err to do that service and come back. And then after 3 days or 2 weeks you know… that kind of service is not necessarily direct because you are not responding to… because illness comes at any time. Now if we had like… if we look at a place like Khawa or Ukhwi, the cost of putting services… the cost of putting facilities there would be so huge that even government cannot afford to these things in villages, but can we think of situation where health workers from say maybe Middlepitts go to these villages spent the whole day everyday moving up at there to actually attend people there. Umm probably we will have people to having access to medical health each day. If you look at farms like Banyana farms, err… the… it's in the Kgalagadi district and the nearest clinic would be like 150 km. In the other district the nearest hospital will be like or health care centre will be like 30km or 50. Now if people need services, they depend on the political representation, they say can you provide service… health service to the people in this area and the one who is in the Ngwaketsi for example, does not have much interest in the people in that area. So you realize that the responsibility of providing health services in that area, it's entirely with the… umm… with Kgalagadi which is so far away from the actual settlement. So that settlement is very far away from the services but the other thing that I think… err… creates shortage, it's… I think basically our development infrastructure di… di… they are centered in one place and people don't get used to working in places outside the center. If you look at Tsabong, Gantsi and Maun for example you still have people resisting to go to those places and yet there are social amenities, there are hospitals there are schools, banks and all infrastructure, you still have people refusing to go there. This is because usually	

when you are there, expectation is… your transfer might be in the rural areas bo-Ukhwi and so on. So I think probably within the healthcare services, I don't know what they teach but the… the ethical aspect that says healthcare comes first before your personal interest because you know… the very area that people are saying they are not… they don't want t o stay longer when they are put there it's like ba a sotlega, there are people there who need service and we cannot get those people out of the area re re it's remote. They need, we need to put that health care issues before our personal interest. That could be… I don't know what they teach but I think that could be developed there. Maybe the last thing I think… ke gore hela generally I have seen people say "no ga re ye koo" (*no, we are not going there*) ba gana go ya ko (*they refuse to go* there) and many of these people who refuse to go, don't even know the places, they have never been there. Maybe they just read stories about Tsabong… it's so far and one would not even tell you how far is Tsabong. So because re fa and we what we have here fa o tswa o re "ke ya ko Khawa" (*when you go to* Khawa)somebody would say err "ga gona (*there is* no) network ya telephone, there is no electricity", all these things. People tend to think this is not the place to go. But the last thing I think gongwe ke gore (*maybe it* is) even our health system is not so involving, I mean you need to have the communities taking the responsibility in terms of some of the basic issues like tsa bo… tsa bo… gatwe ke eng..<br><br>INT: first aid?<br><br>P3: first aid and so on. And taking the responsibility ya go itse gore (*to knowI* if somebody is not well… you know that information… you know the broadcasting… the general information about health because that's why we experience people… he has nowhere to go, there is no one at… there is no medical center, there is no nurse, the only person he is thinking of is the traditional doctor. So le bone ga ba kgone go i-detacha gone foo (*they cannot detach themselves from there*), so you… you… we also need to take these people on board le tsone (*and also*) di-traditional practices re… re… so that they support di-shortages tsa… tsa… tsa (of) health personnel.<br><br>INT: anybody about the shortages?<br><br>P1: maybe I… I… P4 had something about the working conditions I think. In rural areas these are… these are stress issues because you know in order for you to be productive, you need to… to have basic amenities like housing. So if really come from, like myself coming all the way from South Africa e be ke isiwa ko… ko… (*then I'm taken* to) like he is talking about Tsabong. Then when I get to Tsabong I find there is no accommodation, then I'm told you have told you have to share and even the way people are made to share; ke gore (*it's just that*) some things should be considered because we appreciate that there is shortage of houses but at least the way those people who are out there in the periphery should make people to share, I think they should be guided. Because you find a single guy is put in the same house with a married woman and at the same time kana a woman will always be caring the children and these houses were of… which… the officers not the senior people, you find that there are only 2 bedrooms meaning that I have to share my bedroom with my kids because my other partner is using the other bedroom. And you know there are so many conflicts that we used to receive when I was at the headquarters because people were fighting, gore now here is this one room which we are supposed to use as a dining room or as a lounge now who has to supply something like TV and the sofas and all those things. Because previously the ministry of health used to actually supply furniture for the lounge and the rooms and the like but I can't remember the year it was stopped. So if I buy you know the sofas and his visitors come and they are behaving ways that I don't feel… I'm not comfortable with their sitting on the sofas, so these are basic small… small things which bring conflict to people who are sharing… made to share accommodation. So I think this one is… it's a condition on itself. And looking again the rural facilities, they are so old and you cannot even be able to move from one area to another without having to… kana ke… ke mo gotweng re a shifta (*isn't it it's hwereby we have*	

to shift) every time we have to offer a service, we have to move in order for you to offer… because they are old and they don't have space… sufficient space to operate on and at the same time we are looking at tsone di MGD per say, gore (*that*) really if we have to archive MGDs which are all health related and we don't have the basic infrastructure how… how… how are you going to be able to succeed. Le lack of equipment especially in the remote area facilities. The equipment that you will find when you are in towns, in the peri-urbans you won't find in the remote area facilities; not that it was not there, it was there before because kana dilo tse they perish, you have to send them all the way, from Kasane you have to send to Ngangagwe gore di ye go baakanngwa (*so that they can be fixed*) and for you to get those items back it's a struggle. So if you don't have the equipment, the facility e o tshwanetseng gore o berekele mo go yone (*that you have to work from)* it's a problem. For further development, if I'm in the remote area clinic or hospital and I want to further my development like people who are around Gaborone who can say "im going to apply to UB to do my masters on these things", you can't when you are out there. So it's another challenge because people really want to be developed and if you are out there it means you are limited because you are not close to these facilities where…which offer the form of correspondence type of learning and the biggest limitation… because now you… you can't… you are not in an area that is accessible to the institutes of higher learning the other constraints now becomes the training plan, gore well maybe I want to pursue maybe masters in FNP and I'm in the training plan and I'm in the remote area facility, for as long as the slots are not sufficient and I was trained much later, maybe I was trained in 2002 and then there are… in front of us there are 10 people who were trained before me, I'm not going to be considered even though I'm in training plan because the slots are limited and I'm out there. In that way if I was close I would actually say because now the… the slots for those people who are going to be supported by government are limited and I did not manage to fit into the slots then it means that I can do whatever that I wanted to do to pursue my cause by distance learning ko UB, I can't because I'm in the remote area facility. How do I even make a request to say I want to study for masters in part… in this particular course when I'm in Kasane or in Kang or somewhere else because that that is disadvantage to people who are actually working the remote area facilities.<br><br>P3: can I… I…do we have quite a number of…. Quite a number of nurses who are idling without jobs because you know we could talk about the facilities that are… you know all these that are not available in the district and still even if we can provide those facilities we can do, the idea there with all these other things and promotions and… it's basically to attract people to work in the rural areas. My view is even if you take a nurse from Gaborone to work in ….. you are actually filling up a post, a vacant position and …. It doesn't mean that the one that is vacating in Gaborone will be replaced; you are creating yet another vacant post. I've seen clinics that are actually operated by other nurses from other clinics, you know one nurse actually running clinics in 3 villages. So nna to me it's basically shortage of the real manpower. Even if you can provide incentives and that we'll still experience the shortage. The one thing that you will normally get particularly in rural areas, very rural areas ke accommodation.You'll get every clinic will be built e na le (*with*) package ya the accommodation in terms of the establishment ya… ya.. ya that are… ya that clinic. The accommodation problems that we usually experience ke tsa bo in… in… in hospitals.<br><br>INT: oh health posts, where there are health posts.<br><br>P3: ee… but gongwe (*maybe)* the issue ya (*of*) self development ke yone (*is the one*) that matter much because one will not want to leave to go another place ko e leng gore (*whereby*) they will not be able to access facilities that would award further development but at the bottom of it to me it's just that we are not producing enough.<br><br>INT: ok, P2 wena wa reng (*what do you say*), it's your take on this.<br><br>P2: people have said a lot I don't know…	

INT: you don't have anymore?

P2: no, I can say something, umm… tota shortage of health human resource mo Botswana I think it just it stands from where we started, at independence, when we started building more of the health facilities but having shortage from output from our health institutions. Umm… it started as far as back then. So we had much renewing of health facilities but the output from our training institutions was not that much. Even if those who were being sent out to train outside the country, the output from those sources was not enough mend our facilities. so to me it's something that is still going to continue because the idea really like if you take clinics in future is to have doctors in every clinic but as of now we are not able to do that because the output of doctors from doctors from outside is not that much. UB has just started medical school, err I think maybe in future as we …. out more of doctors our own university, maybe in the long rum we'll reach ou… ou… our goals. Umm… but I think ministry, R you might correct me there, we clustered clinics so that we can put doctors there to attend to many clinics in a cluster, it's a way of doing-stop-measure at the moment but umm it's not an ideal situation but we… there is nothing we could do. The other thing as we were talking I think maybe our department of corporate services would share with you our attraction and retention strategy because it does address some of the issues we… we were talking about. Umm P3 le ene, he raise something, the very thing that is of concern to the ministry of health whereby you find that our referral system has been demarcated you find that a certain village will be serve better if it was in another district instead of the one that it is in. so we have a project ya go lebelela (*to research*), organizational and management review ya DHMT le ministry of health, so that I think will be part of the discussion so that we can solve that issue. Umm… the other thing there was a guy from BOTUSA, in think in the about 2 months or 3 months ago he made sort of a study on... resource maybe in the primary health facilities, maybe it could also help.

INT: I'd love to see that.

P2: I'll… I'll… I'll forward you the information. And also our research people, they are doing resource availability mapping in the primary health care, ba lebeletse di-clinics. So Bo R are doing that, I will find out how far they are. I think it is something that can support the study. Umm ga ke itse gore ke tlogetse eng (*I don't know what I have left*). Ya otherwise tse dingwe they've covered I will be saying the same thing.

INT: I think i… thank you very much for all the contribution. We are talking about adequate numbers trained that generally we are saying they are inadequate. P2 you said there is the output from the external or foreign institutions is inadequate. I was actually working with the ministry of health and I found from 1997-2010, one thousand, seven hundred and something, I have the exact figure if… it's on my laptop because they sent me a huge spreadsheet so I was calculating, almost one thousand, seven hundred something health professional, graduated from universities around the world and 800, almost 798 of those were doctors who graduated between 97 and 9 and 2010. So for me looking at that the output is not adequate, they are not coming back. Is it an incentive issue, is it other issues, I think that's just something I've wondered. One other thing that I've tried to do over the years that I have failed to follow up whether just providing information because if you leave this country as many of us did when we were 18, 19 and you would go and stay in Australia for 6 years and then after that you say ok I will do my internship a year or 2 years and never have any contact whatsoever with… not… ke gore not know at all what the health ser… when you leave here… you've never been sick… when I left for UK I've never been sick, I've never been to a clinic except ka mokento when I was small. Otherwise I have never been to a hospital, never been to a clinic… no clue, no idea whatsoever how the health system… so I have always wondered should we actively

actually try to find… actually try work with education… find out where these kids are and give them information from time to time; what's happening, what's the plan, what's the vision and what… because I think at some point in time I tried to do a little bit with those in Ireland and actually I think they started to come home slowly, slowly… you know every year finding out what's happening there sometimes. And also… so I… this is just something I was saying… we have a huge pull of doctors out there in the world, there are more Batswana doctors in the world than they are here in Botswana. So that… and actually I also worked out that it costs… I also calculated the costs that I got from I think 4 institutions or 5 institutions that are from ministry of education, it was almost… I have the exact figures but it was more than 140 million Pula that was paid just last year for… for medical… just medi… this… so the investment isn't small. So anyway we talked about adequate numbers and we were going to say… and you… you going to… P4? was going to say something to about incentive maybe we can continue because we… some of these will come back again. And then the other issue that I want to find out whether di-shortages especially in rural areas could it be also an issue of distribution or deployment? The reason why I say that; in the integrated health services plan ya ministry of health I think it shows there that 60, is it 60% of the doctors actually are in Francistown and Gaborone which serves 15% of Botswana population. So I was wondering could that be a contributing factor; just the way they are distributed around the country.

P1: I think that is also a contributing factor. Ke gore (*it's just that*) I don't know how best it can work gore maybe before… kana when the distribution is made ke raya fa go dirwa (*I mean when*) this establishment people are not involved, ee.

INT: who's the people?

P1: I don't know; it's the ministry of health and DPSM for that establishment. So I think the… the… the information that is needed by policy makers, DPSM as well as the ministry of health is to see the statistics in terms of the numbers of patients who are seen in each and every facility so that they adequately address the issue of human resource placement because if you are in Gaborone and you are talking giving (*cant get the name of the place*) two nurses while you don't know the work load of that facility it means that you are really doing nothing. Because you are…ke gore… it's like… these facilities are… I know when I was in the hospitals that… go ne gotwe primary hospitals should operate with 50 nurses and these other primary hospitals will operate with 20 nurses. Then you get to wonder are they looking at the annual reports in order for them to make such a decision, because also when you look to… when you look at the annual reports, the annual reports will give you something different to what the establishment is telling you. So I think really in order for the two departments to actually say this is the establishment of any particular facility they should have first consulted the records. And also looking at the records they should also look at the commonest conditions that are seen in the facility so that they will be able to say because of this we need this type of person who is trained so that they adequately address now the issue of establishment. So P3 le ene o ne a kile a botsa; don't we have enough nurses in the country. There was a time when Mma Tlou was visited by… we've some agencies P3 which actually employs nurses. So these nurses register with these agencies so that when there is a need for any facility to have more nurses than what they have like at times of the outbreaks and so forth, we can actually get the nurses from the agencies. So by the time I left and the time Mma Tlou left the ministry of health… you can imagine how long back when she had actually called these agencies because she was also looking at the shortage of nurses in the facilities, to say can we actually engage these nurses when we need them from the agencies. And the most interesting thing was that we thought that once we engage these nurses they are going to claim more money than what the ministry can pay but what they were saying was that; you pay the nursesusual… you know the salary you have always been paying them but rona as the agency we are not going to charge you as the ministry, you send the salary to us, rona for the administrative purpose we are going to draw money from the salary of these nurses. So the ministry was not going to pay anything, so fro… im saying this because I do believe that there nurses who are out there and are not

employed and this is the reason why they enroll in the agencies when we at the same time are having the shortages in the facilities. so nurses are there the only thing is I cannot quantify how many are there in the 3 agencies that we have in Gaborone but they are there because they have registered with the agencies. So the… the… the… because we wanted to know if they are there now who uses them if the ministry if not using them. So they told us about bo-Orapa, the mine hospitals, the private hospital and then there was a private hospital… another private hospital in Maun, you see. So there are some but I cannot quantify. I cannot say the ones that are out there in the agencies would be actually sufficient to cope with need that we have in our facilities. The other… I think the other shortage comes from the job descriptions that we have, looking at… kana a kere… when you look at the job description… when somebody… there is an advert in the newspapers, then they say they want to employ a driver; so this driver will be the driver, will be the messenger so it means he is doing a multiplicity of activities. So with us the shortages in health are also created by the type of job description that we have because now I'm going to employ a driver and then I want to employ a messenger. So if now this messenger is not around, this is the thing that I noted in Gaborone, when I was out there we did not have a problem. So I don't know in how many areas this is happening. So if I have a driver and this messenger is not around then you actually ask the driver to deliver something to O he says "nna kana I'm a driver" and you try to… you know… put it in his mind kana mme (*but*) there is this portion in your job description that says this. And there is one who actually won the case because we had actually forced the person to actually take the specimen from one of the clinics to extension 2. He won the case because it was not in his job description. So I think the type of job descriptions that we have also limit you know… our capabilities within the health care system because you cannot have… it's so many people… you have a driver now you have a messenger; the driver says "nna my job is to drive", and now she has to take the second person to sit next to him just to deliver a letter ko ofising ya ga molaodi (*to the office of the District Commissioner*). So this is a waste of human resource, so I think we should also look at the type of job descriptions that we have.

INT: ok, thank you very much. Anyone who want to say anything more? Umm… what about attrition, is that a problem? Are we keeping… 'cause when P3 was asking whether there are nurses out there or doctors even for that matter, I mean could they be that we are filling up a bucket that doesn't fill, when I was younger it was said that's how you decide somebody has mental illness… is when they can keep putting water in the bucket. Could that be why maybe we are in trouble?

P1&P4: yes…

P4: there is some attrition but when you compare the total establishment of the ministry it's not that much except for… from last year when we heard that some of them are going to bo bo bo-Namibia. But I also wanted to to to point out that yes I agree with her gore the current job descriptions need to be reviewed. And in fact the ministry has started to work on the new JEDs, but the other thing that is an issue regarding employment of the health care personnel is that we are also limited in terms of positions to absorb them because that is a government-wide problem; gore (*that*) it will be decide gore gompieno for the coming 2 years no more new positions whereas we still have shortages in terms of healthcare workers err across the country. So that's one of the challenges tse e leng gore di dira gore tota go nne le shortage (*that cause the shortage*).

INT: when I was in government I know there was also huge vacancy rate, has that been addressed?

P4: that… that has been addressed.

INT: ok

P1: but I think the other… especially in the remote area facilities the shortages are also increased by people who refuse to go like P3 is saying. Somebody refuses to go and work in a remote area facility. So some really actually resign because they feel they cannot go there because they are not going to cope like he is saying the person would have never been to the to… to that area. But secondly, I don't know if it's still happening now but I think it it because where I am now, we have a vacancy for somebody, a matron who will be operating at D2. So what happens in the ministry of health is that because now there is this person who had refused to go and is occupying this position, he, she occupies the position ya Gantsi a le ko (*of Gantsi whereas she is* in) Gaborone so wena when you look at your establishment you say but I have a vacancy here then you keep sending the request gore can you please fill this position, then you are told the position is filled. The day you come to the ministry of health then you request the staff from the ….. you see F, your name… appearing gotwe o ko Gantsi but wena you are not in Gantsi you are in Gaborone. So I think this is one of the things that the ministry of health should look into, gore they should not hold positions tsa facilities in some areas, ba di holda (*they hold*) ka officers who are not physically there because it creates shortages especially I the remote areas facilities. Because even here in Gaborone we have been requesting to have somebody to act for that position and people are not… none of these people have been paid because they were promoted on exactly the same day. So because of that we said ok, looking… how can we best make them to actually act in this position so the second step was to look at who was appointed first, first appointment so that we make this person to act first. So now the second… the first person will be starting to act this coming month again second round she hasn't been paid, the one who is acting now has not been paid and my… my… I… I want to believe that these people are not paid and we… there is no communication coming from the ministry of health to say these people are not paid because somebody is holding the position because looking at the establishment of Princess Marina hospital we've got… we need to have only three nurses there at D2 but there are four so I want to believe that this fourth person is holding position ya (*of*) DHMT gape ko godimo (*at the top*), but the prudent thing was for the ministry to actually inform us that the position is not vacant. It is held by somebody in this particular facility but it does not happen. So for you, you become so stupid because every time you request for somebody to act and they know that the position is not vacant. So the vacancies are created by people who are holding positions elsewhere and it is not filled where the physical person is not there.<br><br>P3: but nna (*me*)… one would look at the issue of decentralization of umm particularly with umm decision making where you actually need to have people of… err officers of high level umm great in the district such that decisions can be made there instead of you know somebody recommending a nurse for promotion a le ko Maun and the decision to promote that nurse is based mo Gaborone and you know that alone go dia (*delays*) the process but if they could umm establish umm directors at D1level in the district then they actually make decisions in terms of filling positions, in terms of you know looking at the structure sa (*for*) the health's err.. situation in the district to say look I need so many positions, so many nurses, so many doctors so that he could be able to recruit le ene (*also*) at that level. We have seen… we have experienced that with district council and the ministry took the decision to allow the district to recruit up to the level of D1, so we are able to attract people even those who are within that we are able to see the potential we can upgrade them to such levels.<br><br>INT: maybe… sorry… to understand following on that one does that mean you are able to keep them there or are they still in the pull that they can be transferred, say if they are doing so well in Ngami district they can be suddenly be transferred to Francistown for instance.<br><br>P3: yes, the powers to recruit will also give the powers to transfer, so if you can recruit a nurse or from a different district and he desires to go to Kgalagadi he is allowed he doesn't have to get the authority from the ministry to do that.<br><br>P4: I just wanted to…to… to comment with the regard to given the power to districts or	

decentralizing gore indeed like P2 has just err err pointed out gore currently there is a consultancy a leng gore it's working on restructuring DHMTs such that err at the end everything… most of the functions will be decentralized to ensure that err decisions are taken at the lower levels. The other thing ke ya.. ya (*is the one of*) course holding or people are cross; nna (*me*) when I joined the ministry ke gore I'm at head quarters but on the position e ko Maun it's just a serious… serious problem, but toward the end of last year we did what we call a head count exercise whereby people go out to the out stations to confirm bodies, the actual bodies in a given facility such that we are… they are currently still working that ensuring in the infinium people should be in positions where they are to avoid this course holding, it's… it's a serious… serious problem like you are saying you will find 5 positions tsa… I mean 5 nurses in a given facility whereas according to the infinium there are 4 positions go raya gore yo mongwe (*it means that the other one*) is cross held against post yako (*at*) Maun so that is the current situation. I also wanted to add on the issue ya (*of*) di-vacancy, gore there is also a challenge in terms of vacancy ya more especially nurses D3 and above because when they are promoted from D4 to D3 they lose… they lose…<br><br>P1: P3000.00<br><br>INT: ijoo! A month, that's bad<br><br>P4: the allowance, so you find that there are always vacancies mo D3 and above because people will decline because you lose an allowance e leng gore (*which is*) it's even more than the difference between D4 and D3. The other vacancies tse e leng gore di nna fela di le fooke tsa doctors (*that are always there are*), specialists doctors because ke (*it is an*) issue ya gore (*of*) we are not able to produce more kana ba e leng (*or the ones that are there*) gore they've specialized ke bone ba ba salang (*they are the ones remaining*) in the countries they've studied.<br><br>INT: maybe we should keep going because many of these will keep coming. Now the other thing that we want to find out is are there gaps or issues or problems related to healthcare workers for primary health or not, if there are gaps what do you think are the most important gaps and issues? We've already I know touched quite a lot of these things, we are looking at issues like whether the people are adequately trained for the job that they are expected to do, whether when they are trained do they actually get to do the jobs they've been trained for, whether there is shortages of certain cadres in particularly and whether they are supported adequately in terms of resources, in terms of coordination, in terms of management and so many of those things are they… they are issues that is guess would have an impact in human resources for primary healthcare. This was just giving ideas of things… this we looking at… but you can go as broad as… I know have touched for some of those of those issues I just thought we could maybe say a bit more.<br><br>P1: you are asking about gaps and…<br><br>INT: basically… are there any gaps or any problems related to health care workers for primary health care? So I mean P4 is talking about skills gaps but I think what we wondered is yes, are they adequately trained to work where we want them to work and even those who are trained are they actually assigned jobs that match what they have been trained for? So many others that you can think about… is it…<br><br>P2: to me… maybe I'm not a doctor or a nurse but I would say what I have been experiencing in health. I will look on issues of maternal mortality and child mortality which is a problem in Botswana. You'll find the number of obstetrician, those specialist doctors who look at err pregnant women; we don't have many of them in our health facilities, so to guide the midwife in there in the outstation, for us to reduce maternal mortality this is how we should act until the patient is referred if fa a na le (*when she/he has*) di-complication to a higher facility. So in the study, the situational analysis that the ministry did carry out we found out that women and	

children being born die because of lack of referral time to a higher level there at the clinic. We take more time to make an informed decision on behalf of the patient, so we end up losing such people. To me I think the skills gap is there umm mid-wives, I think they are not enough, I think they don't keep updating their skills. The other thing that is happening as a ministry of health, we don't have a policy for sending an expatriate for anything, for any further training. We only train Batswana, so you find that…

INT: mme external… sending them to outside the country..

P2: no le internal 2 weeks or whatever.

INT: they don't do kitso or you mean?

P2: those kitso they do but I mean training that we can identify that P2 lacks this maybe let's send him for 4 weeks in VETs or something. We don't do that. So those could be one of the challenges that causes the shortage of skills within our healthcare workers.

P1: I think the other compounding factor is placement of newly qualified… looking at the MGD files err placement of newly qualified mid-wives, when you in the remote area, you have just qualified as a midwife and you are placed somewhere out there alone, so kana in order for you to perfect your skills at least you should be working with some other colleagues so that you can see you they manage some of these conditions especially emergency conditions. So you find that newly qualified mid-wives, ke raya mo dicliniking and health posts she is placed out there alone and working at the referral distance, to the nearest referral facility it's too far. This is how we end up at times with unnecessary maternal deaths because the way the newly qualified mid-wives I don't think it makes some sense. So P2 was just talking about the skills gap in terms of the specialists like obstetricians kana gynecologists, we do appreciate that these people are limited in numbers and then you will find them akere in Princess Marina, Nyangangwe and recently Maun and you know these are referral centers which actually need to have these people and because of their shortage it means that they are not placed where they are also mostly needed. Because also I think the other thing to do will be to have… o ne o reng P2… those clusters, where they can be placed so if now you are in an area like Gantsi, looking at the vastness of Gantsi right up to bo-Tsabong bo-Hukuntsi then you don't have a single obstetrician there and kana emergency is emergency, it comes at any time so actually referring a woman e le a serious case coming all the way to Gaborone has always been a challenge because of the infrastructure and also of the airstrip in Gantsi. Ga gona di-lights, you go to Kasane ga gona di-lights, so this also compounds the factor of shortage of human… especially ya di-human resource in those areas so really their shortage really is compounding failure of the ministry of health to save lives that would otherwise have not been lost.

INT: maybe… ok

P4: just to add on what they are saying err P3 mentioned the issue regarding the distance that people travel to get assistance, o tla fitlhela e le gore (*you find that*) di-issue tsa (*about*) bo-maternal mortality that's one of the causes because a pregnant woman yo e leng gore is about to deliver and there are some complications has to travel from one area a latela sepatela se se ka mo thusang (*to go to the hospital that can help her*) very far and in the way go na le those complications. The other thing I think gongwe (*maybe*) we are not doing enough in terms of public health issues gore we are not involving the community like he is saying gone go ba ruta gore (*he is saying that they teach*) what required care they need to know such that before they get to help in the facilities this is what they should do.

INT: I think the other thing that i just want to make as a point 'cause I have been looking through the documents of the ministry of health and I really think this is an important issue about having specialists but I know that the school of medicine is introducing or training new

cadres of specialists who is called the family physician who in many countries, actually in many countries …. very strong health services like bo-Holland, bo-Australia and New Zealand and bo-UK. They are the backbone of primary healthcare in that they specialized… especially in an African setting they will attend to emergency, obstetrical case, emergency pediatrics, even emergency surgical cases'cause they are taught to do emergency surgeries… I don't know whether we we see there is a place for those people as well because the cost of having one obstetrician this is just my… and I want to just put it up, if you put one obstetrician in Gantsi it's tough to be the only obstetrician because when you need an obstetrician then you need two, then a pediatrician you probably need two, you can't just… for them to function they have to go on leave, but if you have three family Physicians they can actually do obstetrics, they can do pediatrics, they can do internal, they can do surgery, they can… one of them can even do anestisia so that you can actually have a functioning unit. But I just know that when I was looking at the documents even the ….. health packages they just don't feature really on the points that we currently have so I just thought i… because we are talking about the skills gaps.

P3: perhaps before you.. maybe... there is this issue that usually comes out in kgotla meetings, the issue of language that we place doctors who don't speak English and err

INT: Setswana

P3: Setswana and the interpreters, they usually… you have to go through 3 languages for that person… for the doctor to communicate with the patient and in the process there is distortion of information what exactly is wrong with this and that, that's another thing because it also competes with the issue of privacy.

INT: so any other issues? But when you get those people in those places do they do the jobs that they are trained for ka gore if you have an establishment re re ok now we have mid-wives, we have these… when people do get there do they actually do the jobs that…

P1: mid-wives they do but I don't know for the others gape ke tsaya gore go tswa gape (*I take it that it is to do with*) le the attitudes of the administrators because you would request and say can you give me two mid-wives. In this area I've got… giving an example of the physical thing that we've got here, we've got an oncology nurse and also we've got a theatre nurse but these people are also trained as mid-wives can we swap so that we get the mid-wives and you take these two, but I think our attitudes as administrators also is something else, so a person will say no I can't take those two because I can have a… a… a single qualified nurse and place her in the oncology, I can have a single qualified nurse and put her in theatre but I can't replace a midwife. So you can see that these people are not operating as oncologist, not operating as theatre nurses because the facilities that where they are placed those services are not there so you would rather a midwife who is going to replace her because she carries two qualifications so I think this also should…maybe as managers we should learn to appreciate. They need for us to have those people who are specialized in those areas in order for us to use them.

INT: P2..

P2: yah, umm I was going to say when we were doing 1997 human resource plan we… we… we did visit… we visited Mabutsane, so we did talk to a midwife there about issues or concerns that they do face or challenges that they do face a mid-wives in the village. She was saying you find that when you have maternal mortality…children dying during child birth, it's not like they are not doing their work. What she was saying was that you find that a woman will be attending this err go bidiwang (*what is it called*)? This err

P1: ANC..

P2: ee (*yes*) a le pregnant… ee ANC a sidilwa and they will advice if there is possibility of a

complication they normally detect that. They will advice that person to say look, as you pregnancy comes near to deliver you have to move near to Goodhope so you can be closer to where you can get assistance in case there are complications. You find that because this woman does not know anyone in Goodhope until towards the close and one day she comes as an emergency in Mabutsane so maybe then deliver a premature baby or something like that. So she was saying they don't have incubators in some of those clinics so that woman will then have to put the child on her breast only to get there they find that the child has died. So those are some of the practical things that mid-wives do face out there in the outstation.

P4: I think lastly, the other thing we keep on saying gone mme there are shortages, the… according to my own observation you'll find that there are many various healthcare professionals in the head quarters, I always wonder gore bone mme… pharmacists ba bantsintsi gone koo (*too many pharmacists there*), what are they doing as we are saying in the facilities, there are serious shortages in terms of pharmacy personnel, go na le ba (*there is*) dental, just various various types.

INT: I think this… maybe we can talk about this, I think what we can talk about… what came out from especially healthcare workers umm progression gongwe maybe it's something you can talk about, but maybe to go to the ministry maybe that's the only route.

P1: that one I think… o a bona ka (*you see in*) '08 with multi something grading, it relieved somebody I think it created a problem for others, ke gore go ile ga nna le (*there was*) this multi-grading event a kere ka 08 so what happened was that as long aa you were at C3 you are moved automatically to C2 as long as the facility has said that they are recommending you and so likewise from C2 to C1 so found that even people who have been serving for a long time but because of limited senior positions they were promoted at the same time… you can imagine you have been in the service for the past 12 years and you have not progressed because the… the… the spaces were limited and now there is this very junior who was employed 2 years ago so I'm going to be moved with this very junior officer who has been looking at me as his senior in terms of my experience, now we are moved at the same time, it was the 1st of May 2008. So all these juniors were promoted with their seniors to C2 likewise from C2 to C1and it created a lot of conflict amongst people. Now… even now since I came to the DHMT I think there it is even more pronounced because most of these people are just at C1and now you need to have somebody to supervise the others in the clinics and they are saying how can I supervise because we were promoted in '08 at the same time. Then you say you are being appointed because we look at your capability your responsibility you know you try to tell them about the way she is performing because of one… to be senior over others, she says no. we are… so you can see gore progression le yone it was good for them because at least after so many years they managed to progress but it was bad because ke gore the issue that was not look into was maybe if we say since you have been serving for the past 12 years and you are promoted with somebody who has been in the service for 2 years maybe let us put you at the different grade. They all entered at the entry grade so this is a big challenge up to now. So I think progression… the multi title grading has solved the problem of stagnation among the C3, but it has not done anything more the D-band.

INT: people at the ministry at a higher level than the people at the..cause that's what I'm trying to find out… they are all at C-level?

P4: at the higher level
P1: no… no… at the ministry, maybe she can te… talk more about it because I know gore (*that*) at the ministry the lowest ke ba (*are the ones for*) C and I know gore most of them they are in the admin more nursing, medical officers, the pharmacy and the like.

INT: is it because… what I'm trying to find out is, a kere there was always this thing ya (*of*) parallel progression that never happened.

P1: it never happened.

INT: do you think that there are… this problem as we are saying its it… could it be a contribution to the fact that people know the you know… that's a route out breaking the glass or is it just that people are made to come anyway…

P1: ya (*of*) D4?

INT: yah, to go into the ministry, why there are so many health professions in the ministry when they are not in clinics. Ee (*yes*) P3

P3: no…

P2: ee (*yes*)… I think it's only that bo- P did not come to this meeting, we… we… this one as a ministry it's up to us, I don't think it's a DPSM issue to say the health posts will be managed by somebody at D4 or whatever and then create that position. I think maybe somehow we have exerted that issue.

P1: yah but you know with the… with the man power planning for 2012/2013kana you know when you are coming from outside and then you come… because to me when I went into the DHMT I was just shocked so we did exactly that we said ok we need to create positions because we looked at the levels of clinics in Gaborone, let me say health facilities in Gaborone. Then when we prepared the manpower budget we said ok, a clinic with maternity and also a clinic with the ARV side maybe we should create a position for somebody who is at D3 then we go down like that. So ke gore… but unfortunately the issue ya madi a seyong go raya gore (*unavailable money it means that*) those positions are not going to happen mme gone what he is saying it's something that will probably work for us gore different level of facility should be managed by people at different levels but not that this is in the clinics only, because looking at the recently upgraded hospitals, you look at Maun, you look at Sekgoma, you look at Scottish Livingstone and so forth and these are newly upgraded facilities, the maximum 324 beds I think- Scottish and then you look at the primary hospital, the least bedded in primary hospital it's Letlhakane with 26 beds. Then you look at the level of the matrons at these health facilities so all the matrons in districts and primary hospitals is D2 but look at the number of beds there. Then when you look at the side of the other cadre; the companion, the medical officers, the level of the medical officer in the district facility is C2 but those ones in the primary are at D1so we are asking why can't it also happen for… you know to differentiate the matrons so that we can see this person has got more responsibility. If you are managing Scottish Livingstone it has go 324 bedsat D2 and then somebody is managing Letlhakane hospital with a maximum beds of 26, really what are we saying as the ministry of health. So I think these are things that should also be looked into gore how do we actually operate these facilities.

P4: like P2 has said it's only that bo- P have not come, I know they have been assigned to look at that, gore we should look at all our various facilities and establish gore tota fela err this type of clinic should be headed at this level such that we address these err

INT: yah no… thank you very much I think the… the other thing that came from the healthcare workers all round is that although health-posts are small and not busy some of them that are very far, they end up… you know new graduates not a midwife, new graduate sent to that health post, the only nurse with health assistant, then you find yourself delivering babies. So I don't know what… should it be… what should advise the peculiarity of the different he… because I think generality we can say health-post can have one nurse and I think in general probably it's ok but when it comes to the health-post that's a 150 km to the nearest clinic, how should that be different because that came out very strong especially in the Ngami area which seemed… they seem more acute with.

P1: I think health-posts even if they are far kana we know gore we've got issues like being off from duty e se day off e le just emergency, because somebody has passed away ko gae (*at home*) kana any type of emergency so it means I'm going to be away from work. So when you do that and there is only one nurse placed in the health post, go raya gore (*it means that*)she leaves the responsibility to the health care… gate ke eng the health assistant. So I would really like to… to… to believe that a health-post should have at least two nurses but one of them should be a midwife because midwifery is… is something… it's a post based qualification that is really needed everywhere in the country. So if gotwe the minimum… the maximum number of nurses in a health-post is two let there be a midwife gore then the general nurse… because now in the absence of the general nurse the midwife can actually undertake the GN duties as well as the midwifery duties. Mme ke tsaya gore gape (*but I take it that*) within the catchment when the other one is away they should look around for somebody to go and take that position, ke raya temporarily.

INT: yah, the other thing that came out strongly, very, very strongly especially in the Ngami, was the fact that there usually is one nurse at these… and then the nurse is with the health education assistant but there is no… there is no security, there is no gate ke eng…
P1: security
INT: so the patients come and knock on their houses at night and most f these are young women and so that actually was… came out… I mean in Mahalapye areas for some reason they said that they agreed gore no patient should come and knock at the door, at the nurse's house… I don't know, I'm not so sure what they said… I think some of those they discussed in the kgotla meetings and agreed but in Ngami it's a huge problem that they raised and so some of them… so I mean these are just some of… because you are policy makers I'm just bringing some of the things that have come out. Actually I think this one ya gore no security and the nurse is alone came out very, very strong because that the… one of the things that they are saying you know the clinic, there is no electricity in the clinic and sometimes there is not even water so some people come and knock at your door and you think, first your house has no electricity as well then you have to go to this clinic where it's dark and so they say they are never really sure of their safety. That came out really, really strong but mainly in Ngami, so just…
INT2: even the healthcare users talked about the accommodation of the healthcare workers as well I'm not sure what the policy makers…
INT: yah, these they've already…
INT2: oh sorry…
INT: so now the next thing we will t… are there any cadres that are much more short in supply than others, we've talked about specialists but because we are talking about primary healthcare are there any spec… particular cadres that will make our primary service work that we tend to struggle to get more than others.

P1: as we said a kere we said kana health we define it as the first point of contact so it should be able to serve the community… give the community the service that they need. So I think the shortage of these other cadres and also post based qualifications it's eminent throughout the country…

INT: so which other cadres?

P1: a kere (*isn't it*) we are now conversing that country-wide we are experiencing issues like alcohol abuse, suicide, this… gate… passion killings and all those things so it calls for the country to actually establish gore go nne le at least kana community ya rona says there should at least be an HIV facility to capture these people when they are still showing signs that maybe they are going to commit murder, maybe they are going to commit suicide, the child is now already an alcoholic, she is abusing alcohol, she is a drug addict; so… so these nurses can actually… if placed out there they would be adequately placed to actually address these issues

tsa suicide, and the abuse of alcohol as well as the abuse of… of… of drugs. So we don't have them, so it's either the community have nurses or maybe the counselors but we don't have them.<br><br>INT: yes…<br><br>P4: I think generally fela we don't have enough err specialist err nurses in the various specialty tsa bone (*their*).<br><br>P1: 'cause you know after this SBRANA, after it was upgraded, we've got… kana this is a specialist hospital, so because we were having general nurses who were placed there and they are still there. They were complaining that they are having a challenge in managing these patients. So we thought maybe we should try as much as possible to post most of the psychiatry mental health nurses to the facility but up to now, most of the facilities are still filled by the general nurses.<br><br>INT: but do we believe there is a role for the psychiatry trained nurses in the other…<br><br>P1: yes<br><br>INT: the other thing that came out very strongly also from the healthcare workers is that because of the shortages they find themselves doing jobs they are not trained for. For instance, in some places health education assistants do things that they really should not do only the nurses should do because they said you know they give… within a health post there is one nurse, a very sick patient comes and the nurse takes that patient to Maun using the only ambulance available to them and then another sick patient comes and they know that it's going to be a while before that nurse can come back, so they often find themselves actually stepping up and doing things that maybe they shouldn't do… they know they shouldn't do but the community doesn't know, you know they see…<br>P3: they see motho yo o apereng white(*a person wearing white*)…<br>INT: so… so… so… and then also nurses talked about you know when they are in this facility they will prescribe and give out drugs, they will… they will do… is that a problem, they will take the blood, they will take… you know they will do…<br>P4: and even collect revenue<br>INT: so is that an issue kana we are talking to policy makers is that an issue, is it something we should really address, will that improve things in terms of healthcare in our primary health facility.<br><br>P1: kana that one has been a challenge for a long time, kana we are talking about primary healthcare, so when we talk primary healthcare we are talking preventive services so to me it means that if you are going to be offering preventive services; a kere we can prevent dilo like eye conditions and the like… but now when you talk about do we really need to have these people multiplicity of jobs nurses have always done that, they have always managed to consult, to dispense, to do everything like that but now I think it came from there that, even when we do this, the ministry of health does not appreciate us, even when we do this, all these things we know gore we might be protected but we are not sure we are really protected, you know something, it belongs to a different kind of cadre and then I do it in the interim I injure the patient, but not because I wanted to cause injury to the patient, it's because I wanted to do good to the patient because of the limited number of the absence of the particular skill in the facility. Thirdly, they always quote this… this… gate ke eng… directive number 2 of 08, o kae (*where is*) P4 which said nurses should be paid for the post basic qualifications that they have so it was out there in 08 and it has never been implemented so they are actually drawing their feet back gore (*that*) why should I now do the job the pharmacist, the job of the laboratory technician and the job of a medical officer who also are paid, ke tsaya gore le ne le ntse le bona kgang ya (*I take it that you have been seeing the issue of*) scarce skill, they are paid scarce skill	

allowance, because I am doing their job bone they are scarce but nna(*me*) what I'm doing for them as a nurse ke tsaya madi (*I take blood*) because the person yo tshwanetseng go tsaya madi (*who is supposed to take blood*) is not in the facility, ke presciba(*I prescribe*) because the doctor is not in my facility and then I'm not recognized by the ministry of health, so I think this one might have come out clearly when interviewing these nurses. So… so… these are the issues tse e leng gore tota they tend to pull back our intention as primary healthcare officers to work out there, give the services that we are supposed to give as a nurse because you find yourself out there without a doctor in a health-post, without somebody from the laboratory, and somebody from pharmacy, so after consulting you might have prescribe some test, so fa ele moroto (*if it is urine*) you collect moroto(*urine*), fa ele (*if it is*) blood you collect blood, so this the area ya a different cadre, it's the area of somebody from the laboratory. So finishing now the collection of samples I move beyond because I've also prescribed some medications so the same person is going to move because she is alone to go and issue the prescriptions that she has also prescribed. Mme gape(*but then*) there are concerns that if I have prescribed something that is wrong, somebody who is in the pharmacy would say hm-hm… but from your assessment this is not the right one. But because I'm a nurse I consulted, I prescribed, I'm issuing, I'm not going to see anything wrong. So I'm the thief and I'm also the judge, so this are some of the issues that the the coming out gore now when we talk about the effectiveness of the primary healthcare are we going to be effective in such situations i don't think we are going to be effective because they are now doing thier feedbacks this this not what i'm supposesd to do and and now they are supported by the the union this unions i I don't know how to call it in pula something… Ee! so so bolau is is part of this nurses who have actually said that this are non nursing duties when i came to the DHMTi found this that nurses are are not doing one two three because bolau has actually told them not to do so.... you asking that who is your employer it is BLLAWU or it is the ministry of health because now we are in the ministry of health so they said no as long as Bolau has written to us because a we even have the letter but the bolau also wrote to say we are in cosultation with the ministry of health to look int o the issue of of non nursing duties so nad we will get back to you now we we are forcing them to work years with a hope that they will be a response from the ministry of health we are re... and to the extent that when they refuse to actually we write them letters to say we refuse to undertake this duty and it is your responsibilty as a health worker to have done one, two, three, four so..... they are saying we wil do it because now we are writing to to us but you have also to push the ministry of health to find out when are they going to respond to us. so primary healthcare fails because of small small things like that that the ministry does does not look into seriously... because if really have to offer primary health care this people are trained to do all this duties... they are trained to do all this duties and because of their training they can be effective in offering the services inth primary healthcare sector. but there are you know they they they is this barrier that the ministry of health should seriously look into gore now what are we saying ka this non nursing duties because it is simple kana rona we have always said this people were trained to undertake this duties they are in a different ministry. Why can't the ministry of health now say this is your responsibility? but now there is another statute that they hold on to which is the nursing act, it clearly says that the nurse cannot do some of this things so meaning that kana the the the the the the the nursing counselling it trys to engage the nursing council to say nursing council can web please just come up with something to say you are reviewing this thing but mean while we are still reviewing the act can we continue but the nursing council does not coming.... so now it is between the nursing council, the ministry of health have actually come up so that this people can offer primary healthcare as it as it is supposed to be they are trained in all this areas, they can consult they can actually the job des... the job	

description for for the senior registered nurse she should dispense but the the the challenge that i was talking about is when now i consult because i'm alone i prescribe and i dispense who picks up kana if there ewas somebody from pharmacy he would say emmeh! your assessment of this patient is this and your diagnosis is this but your medication the prescription is not relevant so if i do all this unknown i'm not going to pick it up

INT: OOH! Thank you very much i think maybe wee...wee... wee... will move on one of the last ones in this are are there adequately support to do the job in terms of the resources that they actually need to to do the job?

P1: in the in the permanent sector?

INT: Ee! P1

P1: No!

P3: I will i will i will say...

P1: Anything that you you are sure to find in the primary healthcare setting is the B.P machine and a thermometer and the fitters' room this three would be able to find in the primary health setting but akere they have to do more than that.

INT: mmm...

P2: But tsone this di temperature thing mmm... thermometera who who is supposed to be visiting them is it the nurse ke raa gore sometimes so of us we see this those whom re tla ba bitsang ba ba... (*we will call them…*)

P1: Health educa… kana the the

P2: No ba ba aparang (*those who wear*) purple or something

P2: Ooh! Theorderlies

P2: The ordernies yes in many clinics mo in rural dikgaolong (*rural areas*) this this are the people who will be doing that ba tsaa ditemperatura ba tsaa di di B.P and they record all this and this is basically because the nurse is not able to do all the whole process until aya go ntsha le le letsone (*he/she dispense the*) the medication so he requests assistance from this and that and that

P1: there was a directive which was actually written by the permanent secretary ene e santse ele mang (*who was it*) who said they should not do that? Ee! Instead then then another cadre was introduced ya healthcare...

P4: auxilliary

P1: Ee! There was an outcry from the country that this people do not actaully know how to check the B.P the temperatures and and all the like so there is a savingram that was written with immediate effect then the the other cadre was introduced the

healthcare auxilaries bonethey are not supposed to...

P3: but with the…

P1: But they find themselves still doing because…

AP: Yeah!

INT: yeah I think that’s what they said…. aaa.... (*coughing*) especially in Mahalapye the they was one lady who said i do it and i know i will not i I will not have anyone who supports but when the patients are there the nurses taking the patients.... anyway so maybe how about other resources you know one of the things i don't know they are people talked about was transport

P4: mmm... le mo Gaborone ga eo....one of the serious serious issues because um... most of the vehicles i don't know gore ee..... In in the eeeeeeeee..... In the past were there enough but most of the vehicles have been boarded and replacement is an issue it’s only in eee.....this year that eee....there were able to replace some of the about 90 80 there still with C.T.O that 98 is far eee... to face the challenges o transport in the country one of the most...

P3: Gape akere (*again*) mmm... the worst part of it ke gore (*is that*) during the time ga (*when*) we were moving from health sector re ya (*moving to the*) kwa council decided to take better vehicles you know this whole the whole lot ya (of...)

P4: Very true... transport is one of the challenges I understand after the takeover it was there at the hospitals (go takiwa ene ele teng ko dipateleng) but it was much better but because during the takeover ba cousele(*the councils*) decided that gore (*that*) the the the all the vehicles there were supposed to to to board and when C.T.O bought them there were all boarded so so some of the the challenges is because of this takeover which was not smooth it was not smooth at all

INT: What about this other forms of communication it might not be all round but i have heared complained all round more most especially in rural areas that (gore) they they don't have telephone in their clinics in the health post even Rojaroja le di Rojaroja even Maun use them to service their people they are part of the Rojaroja so they they have only find that they have to use their cellphones and there is no system to actually allow them to be refunded or but they they one infact one have education assistants being a story that she used her cellphone she ran out of units and she borrowed the patient's mother's cellphone to continue that was the only way they can get help

P4: Yes! That has been a challenge that’s why I kere (*say*) P should be here but eee... I I I remember the P'S made decision gore (*that*) where there are no telephone lines they should be given a eeee.... an amount of units per per month so I don't know why that is not been implemented if there are still places where people fuse their cell phone units

P1: And even in Mogoditshane (Le Mogoditshane le gone).

INT: yah even at (leko) Mogoditshane the medical officer from Mogoditshane was sitting here yesterday and i remember she was....... (Laughs)

P1: In Mogoditshane they don't have.... they they don't have....telephone so they keep on buying their own scratch cards

P2: So the phone there its for (ke ya) scratch cards?

P1: But they are not supported.... ke gore (it is) i buy my cell a scratch card at (ko) Mogoditshane

P3: Probably this is limited by maybe the (gongwe di) resources but if it was the the the the issue of (ya) finance because eee... it is the decision that the government has taken that all structures or officers who for some reason are required to communicate it to um... their respective structures and have to use their own cellphones it should be recommended that there should be given a certain amount of airtime so that they can....

P2: Communicate...

P3: Because they co-facilitated the service but you know you look at the the nurses that are in the health service and look at how much that will cost and then you don't have the budget and it becomes a problem

INT: May be we can keep going and we... P3! Ehe! Is there a problem of healthcare workers in rural areas? Is there a problem of actually keeping and attracting healthcare workers inrural areas the one that we talked about (e reneng re e buile) I think we have covered most of them. lets talk about the living conditions the remoteness I think we have already covered but but and and the one thing that came out maybe we haven't talked a lot about is the cost of living in in this places and um... we talked about opportunities for professional development the other one that came out that I just wanted to pass by our policy makers its of availability of schools for for children and jobs for for their spouses so maybe where I come from I don't feel its really very rural but the nurse there was saying you know my husband is an engineer so what job will he get in Pilikwe so you know (laughing) so um... I I I don't whether we wanted to make any comments about this because I’m and I’m glad molaodi is here because a lot of this things health is very important but i think some things need more than the ministry of health to actually address

P3: Yeah! its it’s a challenge it’s a challenge particularly the issue of transfers and you know placing somebody with the spouse and some things like that um… the issue of the (di) transfers is taking somebody from another eee… clinic to other one um… if for example you have eee…a nurse who have been eee… staying there for lets say seven years and you you realise that nurse has been probably transferred may be twice or three times and we are saying how can you transfer somebody when there is nobody there because we know once he leaves then we are going to stay for about maybe te… even a year without a nurse there so more often the the councilors once you announce such an announcement they will do what they can to block that transfer until somebody is available so that’s why people will stay much longer than even what the policy prescribes…

Int: yeah! One of the nurses they have been there for seven years she is actually right form institute of health sciences…

P4: So as much as we try to facilitate you know the comfort of the employees in the districts you know if of other ministries you know looking at their priorities still eee… becomes eee… a challenge on us
Int: how can that be addressed akere the the there are platforms because I think as much as we is good for health professionals that they should be what it is? (gate ke eng) the outreach ba and they want to do things because they have a service that is needed by the people you know at the end of the day I think its important that you know they are someone's doctor and someone's when there were growing up every parent will have dreams of their daughter progressing to be this and that and also I think they have dreams as well and so if what I'm the reasons why I'm saying its important that I think is part of our training to get that and also important that even with that you know there are some basic things that everyone dreams to attain to but if you are going to be in a place for ten years where you know that my colleague who is in Pilikwe or to a remote area there can have internet because you know the the wireless is there and the electricity and so they can maybe enroll with UNISA if somewhere else even if I beat them in class I have no that opportunity I'm there for ten years so so that's why I say its important that P3 is here because obviously this goes beyond his help. Definitely within the planning um… the program within the districts we obviously look at the issue of electricity availability of electricity but as um… for issues of providing facilities like like internet um… communication and systems telephones and so on its the ministry itself that should make sure that in a particular area there is internet there is ele… um… there is telephone conversation and all this other needs but the basic infrastructure of um… telephone eee.. electricity we we try by all means to to make sure that within our planning process they are provided we even go beyond to actually like in the districts we managed the housing um…there is specific um…professional cadres we we don't want to keep them in the the queue we don't want to have a situation where we are saying there is a doctor in in Gaborone he cannot come to Goshe he cannot come to um…Bokspits because there is no accommodation we first make sure that there is accommodationfor that we give them priority to even go to the issue of getting private houses to accommodate them
AP: I will talk about the cost of living akere we want to talk about…

Int: ee! P1

P1: I think in the from of reversal I have I have stayed for 1,2,3,4, for five years in the remote area facilities so comparatively the cost of living when I was out there in the remote area facilities was much much cheaper when when I'm in a city or in a peri-urban area because the the the rental there is was I think I was paying something like hundred and forty five pula (P145)

P2: Elea eng?

P1: Rent to to pay the house and again because you know you know don't access you know… toropo (*town*) you are just placed there you don't you don't have to spend unnecessarily so you spend you like when you go home you get some amenities and then you keep them there. So the only time you will be spending a bit more is when you are travelling now to home and then back but when when you are there really you don't spend much I used to spend three to four hundred pula per month (laughs) I have never done that since I came to to Gaborone because of you know the type of life and

its expensive…<br><br>INT: when I was in Maun even in Mahalapye I found that um… what the people said when we were with the council especially with the accomodation in council as soon as the ministry takeover they actually were told to vacate council housing there are no longer priority to now they they have to lok for houses in the private market and that actually in Maun actually were not cheap and and even those in Mahalapye were complaining I mean I was smiling because I was seeing well maybe in Gaborone is not very high for them in Mahalapye was one point eight (1800) for a house at a there were been found very high so I think the cost of living actually came out as a very strong thing for health workers in Maun and in in and around Mahalapye because those who there is no electricity they say their problem is you can't buy fresh and keep it you know so so I think there were the they actually said you know I did make a point that aah! But where do you spend money on because but they say know this is expensive we don't have a car, to rent a car to get your staff from where you are taking and then you can't so you have to do that quite a lot because you can't keep anything fresh this are areas where you don't need electricity akere?<br><br>P1: Ee, where they don't have electricity akere!<br><br>P4: Even even where the the they have electricity you know the shops like fancy shops are very far like eee…you take the nurse in Bokspits he has to travel town hundred kilometers to get supplies from Tsabong even when we used to just before bo Choppies eee… opened like last year we used to do the shopping in Kanye or either in Jwaneng or go to South Africa there is another ton that is almost the same distance its its expensive do you think you have saved if you are not like going home and so on because there is not much that you spend money on but you know when I wsa in Tsabong and I have to go home ko Chadibe and going back there I will spend going by my own vehicle I will spend I think roughly five thousnd at a time and its very very costing and it actually it takes every savings that I had but imagine if you have to go to to go home maybe twice as going for a funeral with your close relatives it is really really costing and the the absence of very good schools you know schools in in the same problems that we experience with the health sector is the same problems that policy faces we have poor um…poor schools and we don't want the children to go to that school so you rather be closer to town where you can actually take you're your children to private school so this is really really challenging.<br><br>P4: I I think the accomodation one is quite a serious one thats why we are saying a the takeover has not been smooth because indeed the the the they have been threatened gore they they should vacate all council houses and that meaning that eee…even when there were still with the council or the local authorities those houses most of those houses were bulid for the health care care workers but unfortunately there there is this problem which the PS is taking up with the local government some were threatened gore council is coming and you should all be out of this council houses …<br><br>P3: I I know that is what was happening when I was in Tsabong but the agreement because we had to advocate for the nurses the agreement that we had was infact the houses may belong to the council but as long as it is occupied by nurse it should remain so until such time that um… ministry of health has arranged proper resources to accommodate their nurses	

P4: If if the PI's the PI's local government has written to all the councils gore come this April ensure that all the healthcare workers are out of the houses if they want to remain in the council houses now they should start to pay rent at market prices so that what eee…the the councils secrectaries were implementing what they had to do….

Int: yesterday with the healthcare workers…

P1: There was even in Gaborone they are a number of houses for healthcare workers but ke tsaya gore ke yone sone se a reng the PI's is looking into because what what was happening now when we had six nurses promoted and transferred out of Gaborone so when this nurses vacated the houses kana the the the problem ke gore now the supplies officer from Princess Marina akere we are suplied by Princess Marina so the suplies officer from Princess Marina cannot go and assess the house so it is assessed by the supplies people from local government and now they have assessed it it is now remains with local government it is no longer going to be occupied available for health workers so we are experiencing the same and we we we we actually have been discussing this issue with bone ba local government that it ha sactually been said that when this houses were built when the proposla was made didn't you justify using the number of nurses, they said yes! We did so now why you reversing to say that isn't the nurse's whcich to actually help with the funds to build this houses now you are chasing them out they said no. kana kana their system is new because they had to get the loan from the bank to actually build those houses so we said ok! Why don't you now communicate with the ministry of health to actually say kana we have to built X number of houses from the funds that we have loaned from the bank so that you can say how much the ministry can actually you know forge out to actually keep keep this people because it is not only nurses we have got doctors and other cadres may be this is the one but accommodation mo matlong a council is still… because every time they say kana I don't if I will have my house tomorrow…

P3: But it's funny if…. That is the contention of local government how then can they allow the nurse to go with the vehicles and remain with the the house, yeah! Because it was the same package…

P1: Even the…
ALL: Even… Yeah! Laughing….

P3: Even if they gave you whatever at least you can argue on the basis of replacement it is easier to replace than to start from the fresh but they should have done the same with even the houses because the… it was the package ee!

P4: Ee! It is also with regard to issue ya ya support staff where where during the takeover the support staff was not not transferred from the ministry of health even to say example revenue collectors most of the clinics have revenue collectors the positions were established to mend those clinics in terms of collecting revenue but when eee… eee… the the the… during the takeover no revenue collector was transferred there were say no these revenue collectors they all fall under treasury department they were not under the health eee…

P1: Clinics!

P: Ee!

P3: Because eee… there are not only collecting for the clinics… they are not only collecting money only for the clinics there were collecting for other other source… sourceful councils, water other other and what…

P1: I think the clinics… in the clinics ee! They were… ee! In the clinics ee!

P4: But not really… clinics are not currently collecting revenues because…

P3: Because I think the council will be happy to give you that manpower because it is a burden to them what will they do with a…

P4: They have refused…

P3: With the at the first place they are no longer collecting um… revenue from water because they don't water water department gateng… water utilities has taken over what would they do with a revenue collectors

P4: ke mathata (*It's a problem*)

P1: Well we don't know

INT: Obviously you are beyond (laughs)

P2: So um… maybe before you pass I was going to say in the… policy because the ministry has realized that we cannot do health alone we need to put other stakeholders on health like ministry of Agriculture, water and so forth. So the the policy has come up with a recommendation to establish a point of eee…coordinating body called… so it will bring those stakeholders together to discuss on these issues… cut across yeah! Yeah!

INT: Ok! The next thing what do you think should be done about the lack of health workers in the prima… in primary healthcare in Botswana. What should be done, we talked about all the problems and all that what can really be done this is for us now to start coming up with strategies what can be done to improve and retain, detract and retain health workers in primary healthcare?

P1: Kana if if the the health a certain number of healthcare workers coming from those areas because kana o tlaabo a theogela ba tswa mo lapeng (*she/hewill be going to work from home*) but the experience because I was there for a long time was that nurses and other cadres who coming from those remote area facility those remote areas did not want to go work there. The only area where the majority of healthcare workers wanted to work ke (*is*) Maun hela (*just*) but you look at Tlokweng, Kasane and all other remote area facilities because you call these particular health worker to say kana go na le (*there is*) this position in this area and we are thinking of actually taking you there I don't want to go and ke bereka kwa lapeng (*work at home*) because I'm going to be pennyless because this people are going to… this one from me my relatives and everybody so so this one it cant work gore (*that*) they should maybe replace those

areas

INT: What else, yeah! That’s another idea that’s an idea so you are saying it won’t work or you are saying it can be tried.

P1: It can be tried but most of the people most of them because they are actually not many but the numbers that we have because we used to just call them for sever… small meetings to say kana this is the situation and and if people don’t want to go and work in your home village but wena jaaka ngwana yo o tswang teng (*you as a someone coming from there*) you are not interested kare (I say) only ba (those)in Maun akere le wena (*even you*) when you went to Maun you realized that quite a number of health workers there; nurses, pharmacy people they come from Maun but for other in Gweta and others they were refusing so I don’t if gore ba (*those in*) Maun ke eng bone ba rata go berekela teng (*why are they ok to work there*)

P4: Gona le difacility (*there are facilities*) ha ha ha(laughs)

INT: But actually when I was in Maun this past time there were two senior nurses who are from Maun and who want to stay in Maun and they are being transferred one is actually I think she was saying she will be transferred to Thamaga or something I don’t remember mmm… but she wanted to stay she is married in Maun but she’s is being transferred out so…

P4: mo gongwe (*the other thing*) I think ke is (the) issue of the tsone (these) strike and I have seen some nurses who were based, married ko (in) Tsabong they are being transferred to other places outside Tsabong and you know not because there is specific reasons, there are specific reasons to say go to Maun to or go to Letlhakeng to for a particular reason or because your skills are highly needed there. One would assume that is the issue of particularly those who are temporarily employed.

INT: Is it a new thing, are we saying it’s a new thing has it happened is it something really now since the strike is happening that’s why people are being transferred?

P1: It is not that it always happened… but I think what he is saying is that people who were based there whoever o ale koo he wanted to be in Tsabong eee… they are bein transferred to ko gore gagwa lebiwa gone gore this person has been in Tsabong and she didn’t mind being in Tsabong.

P4: Because in terms of policy the the transfer should be you know discussed with you if you are comfortable with certain places but even though not necessarily meaning that it would you know that you will be taken but atleast you would show your interest in going there or showing that you would not wish to go to another place

INT: So any other thing to try we could try anything and see if people who come from certain areas maybe incentified to stay in their own area or something… What else?

P4: I think the issue ya ya ya (of) increasing the output in our our um… health eee…institutions should also be looked into kana (because) currently most of our healthcare… health workers are expatriates if they decide to go we will have a serious serious challenge keya one hela ya di ya di (*the other issue is of this thing*) incentives

to work in rural areas for instance eee…bo (the) housing allowance so bo (*the*) housing subsidies bo eee… subsidies in terms of utilities giving them priority to to those in rural areas in terms of eee…causation for training I think those are some of the issues that can you know motivate people to go and work in the rural areas<br><br>P4: I think we will also need to actually make a followup or actually have a sought of an inventory of what do we have in terms of public I mean health services I mean doctors we trained and how many nurses… where are this people so that we could maybe even involve them in I mean ask them what is that you need so that we can set new inventory<br><br>INT: Yeah! I think part of this actually talking about is to try to find that and doctors working with P2 we trying to find out where this people where are and now this experts… we got a lot from he… when talking to healthcare workers what would make them to stay you know the incentives came up but incentives were not just salaries you know it was things like if you know you are told when you go and stay in Makakung you go there for three years and then you will be transferred and then you will also be prioritized as we were saying maybe also and been trained people would go to Makakung knowing this is my gate to… so you will know there will be someone in Makakung knowing that this is my gate to profession actually in Zambia I crossed the border I know I even did it for doctors we will go and live in the remotest area I think they could'nt get eee… what is it? (gate keng?) They cant get loans to buy cars you will get a loan buy car and when you finish two years you go for training so now they have doctors in rural areas in Zambia.<br><br>P1: I think another thing other strategy would be to to even create senior positions for the for those areas who went there and now you know gore kana as as a laboratory person this is where I'm just going to be but now if if senior positions are created for those areas ke tsaya gore (*I think that*) then you are promoted to third senior position in in that facility I think people will be ready to go but with also the the the maximum number of years the if like this a the grant line says the minimum o a bona gore (*you see that*) minimum is minimum but if it tells me maximum number of years it because it bind in the the people who are responsible for moving there transfer… transferring people around to be aware that this person kana has completed the maximum number of years she/he served in a particular facility and they should be moved out because kana the the the the guide that guideline I think should also not just be the guideline it should be the policy, the policy binds more than the guideline because at the end of the day if I come you are going to see mme kana(however) this is a guideline whereas if is a policy it is binding so I think if now we can have policy showing the maximum number of years one can serve in the remote area plus the incentives like P4 was saying.<br><br>INT: Ok! Now how about um… how do we get the right skill is mix in this areas what should we do I think one thing is about numbers but we were talking about maybe we are producing many general nurses and we are not producing enough midwives you know but the question of who should be really the skills that requires how do we actualy address that issue<br><br>P2: That that that (interrupted) that issue from the integrated healthservice plan is there whereby you say clinics should have this and this and this…yes	

INT: So

P4: I think the next the next thing is for us to establish gore mme currently on the ground how has we distributed eee… the posts to various eee…clinics

P1: I don't want to dispute what what is there wa ke eng? The… motivated health…… but my concern is who was involved when that document came to be because if you say a clinic should have this and this and this and this is it going to be the same country wide because if we are going to to the same criteria for for a clinic in Gaborone and then we use the same criteria for a clinic some where there in the outside of… yes, I think we are going to get… I think the starting point that would have been looking at the statistics of the commonest conditions that you find in that area maybe over five years and then you can see that the conditions the commonets conditions that are are in this particular area are one, two,three, four from that information I think this is where you can actually now say because now we have got this thing now you know the data that we have from that information and this is where you can say a cli… a clinic in this area needs to have somebody trained in this one trained in this because of the conditions that are prevalent in those areas but if we are just going to say a clinic well this people trained in this areas I think we are simply going to get it wrong because now we were we just looking at the bla… at the blankest statement that gore (*that it is*) clinic so we were not looking at the conditions that are common in those areas so I think this should this should…. From from that information I think now we will be able to find very interesting issues in human resources in this areas.

INT: P2 I think maybe you can just highlight… enlighten us about how the integrated health services plan the consultation you know what informed it I know you have read about it…

P2: There was a situation which looked at eee…problems per district what districts are facing eee… and then after that the way the way different um… reference groups which involves very clinical… I think maybe P1 might have been… and also the staffing developed ways on that yes…

INT: Yes! I guess we can also look at it that has already means for strategic planning now what about task shifting I know that is something we talked about a lot, is it something that can help us task shifting usually involves actually looking at what what are the skills or what what are the skills that are required in this place…

P2: Maybe before we pass because the issue that we are discussing are the issues that hinge on um…on the this strategy what is it called (gate se bidiwang…) Attraction and retention strategy because sometimes we can have nice ideas and plans which involves human resources but if DPSM decides to lump us with the files this and that we will never move even if we can come up with nice strategies to retain our people

P4: Even if I will sound like I want to finger point but (mme gone) like you are saying most of these issues I think the ministry of health has done a lot in terms of training and to address but… eee… kana… the overall overseer of policy maker o o wa human resources ke DPSM eee… we have come up with that draft ya attraction rentention strategy which has most of this issues that we are discussing but it is with DPSM for a

year now not even a single response to say we are figuring that so duties involving non nursing duties eee… has also been with DPSM the issue regarding eee… the nurses with several diplomas have been with DPSM and nothing is moving so we can come up with all these minds and wonderful suggestions which one have responsible at the highest level to make decisions if they don't appreciate hela like he is saying tota health should not be lump…

P3: it is a competing thing I think depending on where you are someone from a different ministry would ee! So it is government is now coming up this sought of a blanket policy one sides fits all ad it doesn't really work they are always concerned about this you may think about imagine in Tsabong they don't have fields where they can plough ploughing fields and you o ba raya ore they should take ISPAAD and then they don't benefit from ISPAAD it's a problem e e leng (which is there)

INT: Skill task shifting task shifting I was saying (kene kere) for the benefit of maybe some of you who may not be familiar with these terms when when you decide what are the tasks the skills that are required you say ok you know this task is currently been done by by a social worker maybe for instance but we think maybe we can train educational assistants well they can actually engage the community at this level we don't need social worker who has four years with a degree where you can actually have same tasks been done by this proper so is this something can be considered or you know something that can be involved in the attraction rentetion plan is this gateng ideas that could be considered or that we should consider to imprve the human resources situation

P2: um… I think this are issues that could be considered it is only that I don't at the moment they did in the integrated health system but think task shifting is one of the recommendations… recommendations there but like she was saying I think these are issues that hinge on these eee… professional councils um… we can say as a ministry to shift this to this but if they don't agree with it at the end of the it won't materialize like at the moment nurses are refusing to do laboratory and pharmacy work in some areas so yeah!

INT: So as an…

P3: I think I think maybe one should see the benefits…

INT: Ok! Maybe I can give an example in Botswana ehe! Le ne…

P3: I was just going to elaborate on…

INT: Ok! Go ahead.

P3: Giving an example of like you were saying kana the nurses are doing multiple jobs which they are trained for but the only hinge is because there are not compensated to do that they are called something else and they are expected to do this other things can they be resignated into ba fiwa (being given) something else that would encompass all other responsibilities for some people be compensated accordingly in terms of the… that's why the pro.. the problem is…

P4: I think with regard to compensating gone like P3 said at the beginning gore maybe is not the issue ya resignation or the the the nature of the position maybe is the issue regarding the the actual responsibilities that go with the resignation gore gone hela we should seriously look into the eee… job effectiveness and descriptions when we craft them such that they cooperate all this tasks.<br><br>INT2: But that could be done outside the the the legal act because there is an act that governs practice so just adding um…adding responsibilities and jobs anything goes wrong we go to Dibotelo Court and say I was to do this because they govern certain professional um… codes of conduct that are expected of each profession<br><br>P1: gape ke gore (*Again its that*) probably it it cant actually actually work to the the task shifting because this are the professionals akere because kana there are trained to to to the nurses association and the nurses the nurses association its its also governed under international council of nurses so whatever happens at the international board I mean (ke raya) at the local level like nurses association of Botswana should have been looked into whether it is within the confined to nursing itself so task shifting I think task shifting would be easier in non professionals when you look at the like we were talking about the driver and messanger when we look at the the health auxiliary this person is a health… she doesn't fall under any any professional group so maybe when we look at this ones as also the clinic kana hopitals we can look at how much we can do the ask shifting when the cadre of the health auxiliary came intrioduced there was a fight between healthcare auxiliary and the the gatwe ke eng (*what is it*) nurse order kana gatweng (*what it is*) nurse order there was a fight because essentially what the the healthcare auxiliary was trained to do and when there were introduced into the facilities they wanted the hospital or the clinic find already doing those tasks so there was a fight because now the orderlies were saying since we have come up I'm just going to sit back and look at working because gate lona this other jobs you agree so when you look at the jobe description saying orderly there are some that are overlapping with those of cleaner because in job description gana le they just do all sorts of things so this is where I'm saying for for the for the duties that are not professional I think task task shifting will be possible on on this other cadres.<br><br>INT: Ok! Maybe maybe I just just follow up on that I think task shifting even even for all all cadres I think it should have backed up with proper job descriptions because I mean that would be something that is deliberate and it will be done deliberately but I think even for professional cadres um… task shifting has been done successfully I mean for instance in in Botswana um…you know midwives has to counsel patients who uptake PMTCT but then a new cadre was introduced and then that task shifting was shifted to this cadre to be layconcillors for for PMTCT and with um… the the HIV expansion rate and care the some nurses were trained as as prescribers for ARV and they actually served a very successful they played a very good role that's why Botswana was able to reach the targets in HIV that it actually reached. In South Africa for instance some of its happenings I think its just because is not recognized its because it is not clear documented and we have nurses called family nurse practitioners what do they do differently from general nurses the same thing um…that for instance in South Africa giving an example they have trained family physicians but they have family practitioners who almost are like family physicians almost… yeah! And there are recognized separately as family well when we talk about them as family pratitioners but our when you look when you look at their training maybe not be very	

different they have things but those are their skills are recognized and so there is no issue ya gore this is a doctors job you know because all our nurses are trained for those jobs its actually recognition they are doing that and then and then they see that this is actually the task that do and it's a professional task usually maybe not the outside bound because this international I think this is happening it is still with the bounds international laws that governs it<br><br>P1: Mm… I'm not I'm not disputing that akere wa bona we are talking about now about someone who was trained to do that and the the family… family practitioner we have many of them they were trained but go to the clinics now and see they are doing it they are not because they are saying that that directive number two they were talking about that is really giving us headache gore eee… that directive of number two is saying is a nurse should be paid for the extra certificate a ministry of health has to devise a way because kana nursing you will find that she is a general nurse, midwife, FMP and obviously the the person is not using all this other other eee… certificates if she is a theatre nurse she is a midwife and whatever go raya gore (*it means that*) when she reaches the facility if there is shortage of midwives in that facility she is going to be put in the martenity and then stays in that facility meaning that she is using only this one she can be a theatre nurse another facility would use her as a better nurse so this person kana go raya gore (*this means that*) the the duty of the nurse is to found strategies on how they going to reimburse them so this ones I have got certificates so ga ga (it's) task shifting in a way but there are proud because I have seen that task that I am a family nurse pra… practitioner so task shifting yes can be considered but I I think again it is should be supported by something which is documented.Yes I think… Because without without any any form of like the the effectiveness description that she is talking about without it they are still going to receive even now what there are saying even what they are saying is that gate I should I should dispense show me the job description that I should dispense and it is only for the nurse who is asking where it is indicated that she should dispense but the C1 and the rest and even the number of C1's we have they are country wide look at the number of C1's in just in Gaborone its about 95% of them so if there are C2 nurses who are there its just less than 10 are the ones who are going to prescribe to dispense what about the 95% of the C1's?<br><br>INT: Yeah! Ee! P4 what were you saying (one oreng) I realize that we have already taken a long time and I really don't want to take any more of your time, so I really don't think clearly but can I maybe just summarise back because were saying that if it is clearly documented defined and there is an understanding of what it means because I I think even the healthcare workers complained about it that I will get gatwe ke eng (what it is called) post basic in midwifery, post basic in that and I'm not an expert in any because of when I get to this they will use me and when I get to that they will use me something like that so what they were saying was they will like to actually develop aan expertise in an area and grow and develop it and and so that anyway so what has already been tried to improve the things to improve the human resources situation?<br><br>P1: Can you come again<br><br>INT: What has already been tried, what strategies has been tried and have worked to try and improve this so that we don't repeat the same things that has already been tried and found that didn't work?	

ALL: Akere!<br><br>P3: Incentives of course go na le (there is) the issue of overtime has been implemented to motivate people to go to or to actually from all other things the issue of um… parallel progression these are the other things that um… don't necessa… some of the programmes don't necessarily force on the health sector in its interity it can't cut across like government sector as um… a response in attracting in retaining profession eee… professional in that eee… cadre gona le dilo tsabo tsa tsa um… diallowances um… but parallel progression and so on but they come a bit difficult to implement because its not very clear gore who is I think you know policy makers probably where we are because most most of them are not in that particular field they tend to look at nurses or the health professionals as you know people who are performing the same thing that's why we have like um… even in the health sector we have different signifying who qualifies for um… scare skills allowance um… you know things like that so maybe we need to categorize them in terms of specific professions so that we know where we are lacking and we know where we need to motivate and put um… motivational um… allowances or something like that because the ones that we have are just general they are not specifically focusing on that particular professional service.<br>P1: gape (*again*) the the that P4 is talking about it is going to actually address the because a kere go ne go tilwe (*is'nt it, it was said that*) they should be the one for midwife the one for general nurse the one for a different as a professional but el eke gore ele registered nurse akere jaanong ga ale professional o tlaabo ele assistant officer ee! So so midwives and all other specilities I I think when I left the ministry of Health they were actually meaning those JED's spe… JED'S specific for for the different specialities but I think also like P3 is talking about about the parallel progression kana I think implementing the parallel progression will also improve kana the the issue here is that in in a facility this why we are having this transfers and people refusing to go on transfer because in order for you to progress to the next level there is no position available in your area now you are promoted on transfer because the position is not there or you find that especially because I have is needed right there in this particular facility but because the position is not there nonetheless I'm going to be transferred out and look at the number of people who have this particular speciality I'm the only one but because the position is not there in in this facility where I'm is where my speciality is needed I'm going to be transferred out so I think parallel progression can actually address this one so that I can progress and develop my line upto a… actually ke tsaya gore (*I take it that*) ga go (*its not*) a problem when the Matron is in a higher level then I know I will progress in the same level as the Matron a le ko godimo progressa kele mo lineng mme le nna keya go goroga mo leveling ya Matron kele mo C2 ka gore nna (*as the matron is at the top, I'm going to progress to the same level when I am in C2*) I'm focusing on my line the Matron is looking at the overall administration ke tsaya gore mme even the pa.. eee… efficient implementation of the parallel progression can work the second secondly ke tsaya gore re kile ra bua ka RASA gore (I *take that we have talked about RASA)* that let let it come back let's leave this idea that go tlogelwe kgang ya gore kana jaanong gona le sekonotiri gona le banka gona le(*now there is tarred roads, there is financial banks there is*) infrastructure but the term of the area is remote because nna (I) if I'm placed in whether gona le ditsela, dishopo le dibanka (*there is roads, shops and financial banks*)to me its remote and mme gape ke tsaya gore (*but again I think*) the the other thing that can be looked in to again is of that (gape ke gone gore) to to to post people to ke raya ke raya (*I mean*)	

there is a midwife even the general law ya re (*says*) at one point at a time as a civil servant you have to go and serve at the remote areas which we all appreciate but a go lebelelwe gore ke tswa kae(*let's look at where I come from*) if nna ke nyetswe (*I'm coming I'm married in*) Mochudi and then its said (kebe gotwe) my remote area I will be transferred to rural areas that are in the you see Northeast or Northwest far there it means that yame ke isiwa ko dikgaolong tse di kwa tse di ko wa bona kana Northeast kana Northwest kwa hela go raya gore(*I'm placed in a remote area*)e e leng gore (*in which*) its far far from home where if we could look at the fact that I'm coming from Mochudi (mme ga ene ele gore gone goka lebelelwa ka gore ke tswa ha Mochudi) the remote area facilities its that they are in the side of Mabutsane (ketse di mo kaha go ya ko ee! Go ya ko Mabutsane oya kwa) then I will be ready to go but if you take me to that far (kwa hela kwa nxexe!) it becomes it becomes a challenge I feel that (ke tsaya gore) maybe maybe this has to be looked into because as a civil servant I know that even if I'm told that in some other time I will have to go to and work in a remote area akere leha ke tsena mo tirong ke boletswe gore kana mme nako nngwe o tla o tla tsamaya oya go bereka) in a remote area akere so this is preparing me at one time that I will go so I'm accepting that it's a stature that it is like that (gore gate ke statute se ntse jalo) but let consider the need that now that (gore mme hela jaanong ka gore) its your turn to go to to go and serve in the remote area where you come from and which is the remote area that because I would I would actually be ready to go if ha gotwe eya Gantsi ke tswa mo Mochudi(*they say go to Gantsi coming from Mochudi*) I will go if they say go to Mochudi, ga gotwe eya Gantsi ke tswa Mochudi I will go but ga gotwe keye (*if they say go*) beyond bo Kasane gate Nxauxau, Pandamatenga! I'm going to ask myself a lot of myself a lot of questions and this is something that would actually tie me to go because P4 ane a bua gore (*was saying that*) at the moment Namibia e butse (has *opened*) so there are taking a lot of nurses in Namibia and well ke raya gore (I mean that…)<br><br>INT: Its further than Nxauxau.<br><br>P1: Ee! (laughs) you know what they are doing because there are talking about conditions of service there ga gotwe operate when they are there they are some who have been actually coming to visit their friends and you know they are so beautiful things about Namibia and le gone gore (*even*) as as a as as an expatriate now they are also considered as expatriates and they are given something on monthly basis as an expatriate as a midwife you are given something else nurse you are given something else so the salary the basic salary is much much of what we have and then those other things that they are payed for makes them to go so very soon now because you remember kabo 1998 when we had a nurses going to to to the UK so I think they were saying things now it is going to be coming because from from from the clinics here we lost twelve already in two weeks they have all gone and kana when they come they are paying their rents they they are also preparing to go…<br><br>P3: Especially that its nearer…<br><br>P1: Especially that its nearer…<br><br>INT: Yeah! Ok, um… so hopefully hopefully the the fact that we are talking we will stamp some of that hopeful…one o reng? ,What were you saying?	

INT2: Well just two issues that I thought eee… policy makers can take on board nurses are licensed to practice they are not licensed to practice in all this um… specialities that they have they are only licensed to practice either a midwife, or a registered nurse so some of the challenges that you are raising about FMP and so forth ke gore when the when the regulatory board right now does not have post registration for FMP's for…Opthalmic nurses<br><br>INT: Opthalmic nurses…<br><br>INT2: fa ba re tota (*actually when they say*) they don't do that they are right because you cannot practice illegally so I I just wanted to shed light the training of nurses really we are not training nurses to dispense nurses are trained to you know in pharmacology, and even le bo bleeding tota (*even bleeding*) when you look at nursing curriculum that is used is not the same as the current one (e e dirisiwang ga e Tshwane leya gompieko) this are not part of the I mean if we are talking about the (ke raya gore ga go buiwa ka) non nursing duties you have to look into that because they are not there I mean that (ke raya gore) its not like when a few decades ago when nurses trained that those eee… talks can be discussion to be held with the relevant relevant bodies…<br><br>INT: What I think I don't know how because I how it is country actually did not have facilitation regulations to to facilitate our health system is that ke gore ke raya gone gore um…(*I mean that*) is it only to the nursing council to to say this is what we will allow and that's it its its not something that is as a country for instance we are training nurses they put people to sleep if I don't wake up my children so if they are not registered its its what I'm saying I'm just surprised gore the council can go the whole country at ransom but I know in many countries they will do recognize I don't know the mechanisms that they recognize them but I know that…<br><br>INT2: mme gona le (*But there is*) provision my dear e eleng gore (*that is*) like for ARV's I know on the nurses got trained for ARV's for those who are prescribing they they had to be an amendement to the act to have them covered as well because its not only about serving the person also have to to be legally recognized that you can do that I believe that jaaka kene kere(*like what I was saying*) those discussions…<br><br>INT: But I know for instance more for as long as I be an opthamologist they will try to recognize opthamology…<br><br>INT2: Actually it's being on the table right now but I was just saying shedding the light that dilo tse dingwe di ka tswa di causiwa (*some things they are caused by*) some simple things.<br><br>INT: Ok!<br>P1: And this is how actually they hold on to to the nursing council gore kana (*that*) I'm not registered to practice as as so so mme ke tsaya gore akere (*I take it that*) now they have moved to the Ministry ke tsaya gore ka (*I take it that*) they should also engage other stakeholders when they when they because I know gore they moved<br><br>P4: I think its physical movement hela.<br><br>P1: Ee! I think they should engage us people because I I at the moment akere they are	

revising the JED Act so now the DCE act can capture capture a lot of these the people are concerned about because when you look at the ke raya fela (*I mean that*) a nurse should not be registered and at the same time we received received eee… a savingram from from primary healthcare mo di-cliniking (*at the clinics*) to say is not is not its it a plan the the the… of the clinical services so that plan was now supported by the letter from bo P which says the facilities should ensure that nurses who are practicing are licensed to do so, so when, the nurses can interpret things you know are so intelligent in interpreting things because what what we we were saying is kana batho ba modimo why do we have to to write this thing like this because ophthalmic nurses are not registered ophthalmic nurses are not registered to the nursing council ke gore (*I mean that*) all all of this cadres are not registered accept the midwife and the community registered nurse this are the only two who are registered by the nursing council so this may come up and say so they say I should not practice because I'm not licensed I'm not licensed to practice as an ophthalmic nurse so what do you do?<br><br>INT2: But tota the deliberations di ale bola you know no stakeholders are involved are just for now if the kana ga gotwe go reviewa (*we say we review*) policy ya amendiwa (*is amended*) it is not an overnight thing but these things are being discussed.<br><br>INT: Ok! We are getting to the end, yeah we are getting to the to the moko (*main thing*) um… so one of the strategies that could improve primary healthcare is building effective primary healthcare teams. What is your understanding of effective primary healthcare teams I know we are really tired this could be a new concept but this is the last but one question…. In your opinion what what are effective primary healthcare teams?<br><br>P1: At facility level?<br><br>INT: Ee! P1<br><br>P1:ke tsaya gore (*I think that*) eee… the primary healthcare kana we akere you know (gore) (that) in public health this are public health oriented people so I think think the primary healthcare team should firstly have public health person…<br>INT: Public health person?<br><br>P1: Public health specialist can be from other cadres we have got the head of nursing the head pharmacy the head of laboratory etc but at the moment we are making people who who oversee performance management so we we we we a team primary healthcare team should have also have that kind of a person kana those people not necessarily that won't be able to operate alone so you need to have that kind of a person.<br><br>INT: So when you say performance you talking of what what kind of person what sort of…<br><br>P1: Performance skilled gate ba bidiwang gate gate (*what are they called*?) performance improvement I don't know whether but ke itse gore (*I know that*)<br><br>INT: They have monitored evaluation skills or what skills should they have?	

P1: We we have those ones who are still in most statistics and all this things but ba keba (*these ones are*) to look into gatweng (what is it?)<br><br>P2: gatweng (*what is it*) tsa quality (*issues of quality*…)<br><br>P1: Issues of quality… issues of quality…<br><br>P2: Le performance<br><br>P2: Mmm…<br><br>INT: And then um… what its value and what will the value of a team what don't we let a nurse do what they have been doing when and how they are doing it and everybody do what they have been doing without necessarily being a team?<br><br>P4: Kana the nna ke gore you know when the districts mo ke gore Gaborone its CBD I find it confusing it's a district within a city but if you go to the districts all efforts tsa tsa government its health education and they are coordinated by the office of the district commissioner all sectors now when this health team thing came out there was a bit of confusion how do we do the monitoring because we expect this structural reports so that we can measure performance but then there was the other thing ga ke itse gore gone go bitswa ka eng (*I don't know what it was called*)? Health committee… during the term ya council now when the health teams pu… ba go bitsang?<br><br>P1: DHMT<br><br>P4: DHMT came in it was like eee… you will have like that was the directive for instance from the ministry the permanent secretary you are going to have DH… DHMT this what you are going to do but then we were saying so what are we going what do we relate to them le bone were saying what do we relate to the district structures so gona that gap because it is also even not pronounced in the planning handbook ya planning… planning manual wa wa o o supang the performance ya distructures within the districts the services tse eleng gore um… dia dispensiwa so mm… this is I think gona le we need to find a way e e leng gore we can have the DHMT to fuse in the structures tsa districts.<br><br>INT: But I think yeah! That is important because when we were going around we found that in most of the districts we have been they did really don'y understand even the DHMT seems to be not so sure what really you know maybe that's something we can come up hope in time akere this is very new and then but the teams we were also looking at for the teams at eee… facility not just at eee… because they will be this is the administrative team at district level but actually at facility level so as a patient when I walk into a clinic and and I have a headache you know I have an expectation of the kind of people or the kind of services that I will receive and that is given in a team that is different people may help us and they might find us so I think that the concept gore also with the understanding that this people will understand they are working as a team well its something that um… what was the question? Something something that um… have been thought about so but that what I'm asking what would be its value be that team what will be its value?	

P1: Eee… I think the output kana kana if you are you are woking at as tea… really as a real team it means I know what he is expected to do and he knows what I'm expected to do so so that if isf there there is a gap between us maybe I'm not there and he si there and he knows P4 is there and he can also fit in as a team go raya gore the the gap um… that I'm leaving when I am away like when I'm in committee meeting it is not going to surface because they are people there who are aware of of the services that I offer and they know precisely what to take care during my presence so so so so so so teamwork I I think is taking responsibility when one member is not around gape re e tsaya gore I'm accounted to make sure gore this team is working as a team ke tsaya gore this what how how team team work and team spirit should be like, no gaps to be visible because one person is not around kana if if we don't have that that kind of cohesion or or that kind of teamwork when I am away they will not be aware that I am away but ke gore all the other people who are supposed to be in that team are not aware the only time that they are going to be aware when a patient maybe comes and says kana I have been waiting for this person because I know is the person who is supposed to be giving me se le se but she is not there so so it's the only time that the supposed team members are going to jump up to say go raya gore she is not there they are going to start looking around but at times how long is the queue outside waiting for my service so ke tsaya gore (*I take it that*) teamwork means really cohesion and understanding each other as folks and be able to fit in when one of the team members is not around.<br><br>INT: And who should be members of this team, members of effective primary healthcare team at facility level?<br><br>P1: At facility level aah! nna ke buile gantsi (I have talked many times ()<br><br>ALL: Laughs!!!<br><br>INT: Who should be in this team?<br><br>P2: To me I don't know if if it means a committee…<br><br>INT: Not a committee…<br><br>P2: Not a committee…<br><br>INT: No! no! a team that should provide this services, I mean go ahead sorry P2 ke seke ka go thiba molomo… go ahead I know you have a lot of information.<br><br>P2: Eeee… I think the the staff at the community level should be there um… eee… don't know specifically but in the integrated service plan we have a list of those who will be working at facility level and those who will be working at the community like social workers there there are listed there only that I can't…<br><br>INT: You mean all those people that are listed?<br><br>P2: Yeah!<br><br>INT: And how about the community should they be a representation from the list or it	

is necessary?

P3: Can I say kana rona we we are undated ka disuse tsone tse um… one would be coming from the hospital coming to Molaodi kana Molaodi I I experience this and this ko sepateleng so I'm reporting what should Molaodi do does the chief medical officer ga a mo itse(*doesn't know him*) he only knows gore I have got to the nurse and ke thusitswe jaana and I was not happy kene ke nale (*i had*) I was supposed to give birth and I lost my baby because somebody told gore dincubatara ga di bereke (*that the incubators are not working*) things like that we we receive many of this tse dingwe are because they are provided by the laws tse re nang le tsone kana the laws tsa rona tse dingwe sale di feta long time back when this facilities are not provided go santse go go gotwe ga go tshwane wa bona (*it is still said it's not bad*)so um at the district somebody is coming to me gore (*that*) kana my relative kana my brother um… ona le problem ya go mental problem o ja dithare meno I want you gore o mo ise ko (*to take him to*) mental and this is what we do ke ene the commissioner o o nang the powers tsa (*of*) detention u… u see what I mean?

INT: We see…

P3: Ee! When you get to the health team ga re itse gore the the nurse ota ko go rona are bolelela gore ministera permanent secretary has done that and that ko meetining its me who is suppose to phone the team ene eo ya…

INT: DHMT

P4: DHMT and I don't even know I don't have the terms of reference nna keya go tsaya like a committee you see how? Ga go nang le di gap teng

P1: Mme ke tsaya gore this team…

INT: Ema pele P4 a bue pele(please give P4 the floor)

P4: Kene ke re kare for the primary healthcare team to be efficient in its operations to me it should have a representative from the community like we said earlier on gore kana that representative will infact will assist us to take information to the community whatever gongwe dissue tsa tsa public health tsa bo gore gona le Malaria this is the person who is going to assist us to teach the community about whatever services we provide in the the facility so its very very critical that they should those people akere gona le hospital advisory committee…Akere eo ke committee…

INT: Yeah! That's a committee

P1: Ee! Kene ke re gape when we look at eee… issues of community akere we we have village health commitees so so this are the people you find that in most cases they are also coming in the morning to give health in the facilities so so ke tsaya gore the the village health commitees are still very important le di ward le sub ward because the village health commitees died out ga go tsena community based home care because there were incentives for community home based care so this people because they were so much conversant with health issues they moved from the village health committee which was not incentified enough and then they joined the community home based

care teams so so you can see that already already see that from the community we also need this community home based people and and from the facilities themselves we also need kana this people are volunteers even though they are incentified but who actually lives there and show them what is expected of them to do so I should believe that each facility should have maybe somebody from the community community… gatweng (*what is it called*)?<br><br>P2: Community member…<br><br>P1: Community based care ke raya (*I mean*)home based care ee! Home based care maybe they focus on local person or somebody like that, looking at the number of community home based care the people within the areas I think the nurse should be there to make sure that she covers certain areas ko go nang (*where there is*)le the community based gatweng (*what is it called*)? Community home based care people and also be supported by the structure tsone tsa home based care volunteers within her area. Ke tsaya gore (*I take it that*)the the team also at the moment we are struggling with T.B so if the team can also have kana we have the T.B coordinator for the whole district ya Gaborone but then who supports this person because you get to the facilities gaba tsaya (*when they take*) this treatment kana go raya gore the doctor go raya gore there should be somebody there to give eseng mo go tweng re santse re le busy go raya gore you will come back later because if she was not aware she will not come back later so if we have those focal persons T.B Focal persons who will be working with the T.B coordinator within the district as well as maybe the community members again will be supporting the volunteers because at the moment the structure that was is very good that was implemented now ke the CTC the community T.B care those portacabins but because now if those portacabins are there and the patients manage to get that treatment in the portacamps ga baya tirong (*when they go to work*) because the other thing that has happened ke gore there there are people who acquainted mo ditirong to give T.B treatment to the people who had T.B so once they have been counseled they will accept gore she is going to be giving me so that I don't forget and I take it and he makes sure that actual slot ke tsaya gore (*I think*) if we have T.B focal person rena le home based care focal person within the facilities because bo nurse akere (*isn't it*) we are talking about the others besides the the general nurses and the management<br><br>INT: What who should be in the team yes?<br><br>P1: Ee! Ke raya gone gore akere (*i mean that*) those those ee! In addition to those nurses who are there gongwe gonale (*maybe there is*) le midwife gongwe gona le wa matlho (*maybe there is one for eyes*) and so forth but we need these people who will be acting in the community the community volunteers, T.B volunteers the home based care volunteers ke gore (*is that*) for for all this smallernyana programs now we know this programs are going to work because they are people who are from the from the from the villages who are part and parcel of this team mm eke tsaya gape gore (*I think also*) in order for for them to work efficiently atleast if there are regular meetings or monthly or quarterly meetings so gore le bone because this are not health professionals they can also come and pot what they are saying irrespective of the fact that they will be reporting to the focal person ha ele wa T.B should be reporting to the focal person wa T.B so ke tsaya gore that kind of structure can actually make the the akere gape gona le di extension teams which have always been there so ke tsaya gore le bone as	

village extension teams and you will find that there is somebody wa health mo teng ke tsaya ke tsaya that can also work.<br><br>INT: Who should lead the team?<br><br>P1: Public health…<br><br>P3: Emang pele before anything ke botse this team are talking about teams or it's a team<br><br>INT: In each facility<br><br>P3: Or in each facility ok!<br><br>INT: Ee! Ke gore lets say a team in Bontleng clinic that providing care in Bontleng clinic.<br><br>P3: We were looking at it in…<br><br>INT: Smaller levels?<br><br>P3: No! eee… gore this is a team that will be overseeing other facilities<br><br>INT: This one working at facility level…not um…<br><br>P2: But if it is at facility level I think the nurse should be in charge…<br><br>INT: As the Matron?<br><br>P1: that's the problem!<br><br>INT: Is there a Matron for each clinic?<br><br>PT: That's the problem.<br><br>P1: Ae!<br><br>P4: No!<br><br>INT: Ok! And then also akere there smaller ones without Matrons at the moment?<br><br>P1: The majority.<br><br>INT: The majority ee! Ke raya gore structural ke raya gore maybe in each place does each place have a Matron?<br><br>P1: The establishment e teng mo kana it says kana ketla bua ka Gaborone ka gore ke mo Gaborone just says the establishment for Gaborone and it will say 200 hundred nurses so how you place this nurses but etla supa gore this one is at D2, 3 at D3, 4 at D4 so so ebe etla ere 200 at C1… Cband wa bona so obo go raya gore from the the	

Matron's office she going to gore jaanong this clinic here o ba neela bokae ha o ba neela bokae…<br><br>INT: Or! The Matron is overall<br><br>P1: The Matron is overall ale ko counseleng so aba lebelela gore maybe like now what is happeneing the the Matrons are area Matrons they are not Matrons for a facility because a certain number of of a facility so this D4 is looking at a certain number of facilities there are seven of them so D3 ba bararo ebe go raya gore the D3 scoup ke sa there are only three of them so…<br><br>INT: So how I we going to how should the quality of the team be evaluated? I know we can't have all the technical information now I mean in just general terms how are we going to make sure this team are being what what are we going to evaluate the quality of their work and the impact of their work because akere we can from team but a team may not necessarily mean…<br><br>P1: Ke tsaya gore ke tsaya gore eee (*I think*)… re re hira a team to evaluate and and where we are lacking and what is very important like like yesterday we were in Old Naledi so we have been always to Old Naledi previously ele gore rene re ile botsong jwa pitso we sold our services to them and we also have we designed um… the complaints procedure from which is placed everywhere in the clinic so akere after after that inter action theme and then we were all the staff to say this is what is expected of you, you are expected to perform like this because we are goung to come back for potsologo pitso so that the community that we are seeing are going to evaluate you so also the number of complaints that we are going to be getting from the from the residents of Old Naledi are going to tell us whether we are performing as expected or not so we went to Old Naledi to yesterday entse ele (*it has been*) potsologo pitso again but we heared one complaint ee! So this one complaint it's a complaint that we felt gore its it was it should have never been happened e e leng gore it was a complaint ya gore (*about*) bo nurse ba chaise (*the nurses to knock off*) instead of because akere that time there is a group that should be knocking of at 1430 and there is a group that should be knocking of at 1630 looking at the duties so if you need a service and then you go to the facility and then you are told that this service is not available at this time it is wrong so akere ke tsaya gore (*I take it that*) you are aware of the practice gore kana gone gotwe gore o ise ngwana mokentong (*you were supposed to take the child for injection*) is between 7:30 and 12 and we said no you offer a service until you knock off so there was that one woman who said I went and they said no the nurses who was there said no I can't there was only one complaint that we addressed also there and we said we are also coming there again so ke tsaya gore kana (*I think that*)you can'y evaluate yourselves.<br><br>INT: What compliments?<br><br>P4: Ke tsaya gore (*I think*) it is also in what he said the the responsiveness of the the team outbreaks can assist us to get the the teams effectiveness…<br><br>INT: So I think we have covered that so in your opinion how do you think of introducing this concept of primary care team as an intervention for primary healthcare in the country just actually emphasizing the concept of primary care teams? What do	

we see it as a potential um… concept where clearly defined and people understand what their roles are and what the community also knows what, what do we see that as a potential strategy to maybe even implement your strategies there?

P2: To me I I mean I I I find it as a most important eee… tool to take health to the community um… if you can look nowadays the NCD's the non- communicable diseases are on the rise not because we are um… food alone and diet and stuff like that but because people are not aware that they should be exercising doing certain things but if we have this team working closer to the community they will be effectively going to teach them about aaa…some of the primary services and how they can eee… avoid themselves eee…getting some of these diseases eee…and also if they have to enroll on time in the healthcare facilities me I find that it will help a lot.

P1: The most important cadre that I forget in the team is the health education assistants that one is crucial yeah!

P3: But I think also maybe we can also maybe if we we borrow from the um… the villages extension teams in relation to district extension teams then we will have like somebody overseeing actually getting reports or getting information from the structures at the the service areas at this institutions the clinics so that it is then reported at the district level where decisions are made

P1: I think to support what he is saying like we are recruiting DHMT rona kana we are still making some of the structures we are not looking to stop because when I went this was where I used to work in the district so I know the structures that were there and really working for us so when I came back to Ga…to to Gaborone I decided I think we should continue repoeting at the UCD but we received an early developments in the UCD because you report all the activities that you undertake within a district those that are related because rona from health ba Agriculture they are also all their sectors report at the UCD and we also ke gore we give reports on our challenges also and then you find that some people can say aa…this is a challenge but this is how we can actually resolve so you find that attending tsone those meetings tsa UCD are very important to us so we started reporting ko UCD again ga re itse gore ba Ministry of Health what are they going to say to us ya (batlo re raya bareng) (laughs) because nna (*I*) I felt gore it was very important committee when I was there at the districts because you get a lot of information there people are not health oriented akere le rona because we we are like so you just think ke health but bone they they think Agriculture and think but if you have problem this is how we can challenge it so that's that's the monitoring tool.

INT: I think I will come learn more about it because…

P1: I will I will tell you when the next meeting is…

INT: Obviously you have to be invited I don't know (laughing)

P3: the the e tsena quarterly…

INT: the last question is in the plans that the ministry of health has produced, what is called the integrated health services have got a lot of, they have a vision and a mission, but they also have values. One of the values is ethics, now because we are talking there

is a shortage in human resources, now as a policy maker sometimes you have to make difficult decisions; ethically and morally challenging because of the resources that are constraint to work with. Um so in your opinion, so basically, this is an opportunity for one to say yes I've faced morally challenging situation because of the shortage and also to say what are the real major issues that make you find, that you often deal with in terms of human resources, maybe not only human resources but in terms of resources, could it be resource allocation when you have to transfer people or any other challenge that could be a challenge.<br><br>P1: the dilemma?<br><br>INT: hmm<br><br>P2: I think as we started, rre molaodi kafa (*here*) he did talk about these ethical things, by saying sometimes in the facility a nurse because of high shortage in the facility, he has to delegate certain things that are not supposed to be delegated to those… he was talking about somebody ga ke itse gore a ke (*I don't know if*) whether those are healthcare or healthcare auxiliary I think… so those are I think some of the ethical dilemmas as health workers we do face having to assign challenging assignments to lower cadres.<br><br>INT: but what about policy level, because these are more at health… healthcare worker and they told me maybe yesterday and the other days but what about at policy level<br>INT2: which you are faced with…<br><br>P3: kana ke gone fela gore (*it's just that*) if somebody has to be in one place for many years and eventually even going to work it's a matter of routine here and there, what do I do there, somebody comes and he so used to gongwe le batho gore everybody fa a go lebelela jaana (*when he looks at you just like this*), I remember one time when I went to a clinic, my eyes were red and so on and it was a Monday, and erile a mpona fela a ba a re this one o letse ko bareng (*when he just saw me he said this one spend the night at a bar*). And I was… ke ne ke lwala tota (*I was seriously ill*) so he thought ke ne ke ile go kopa sick leave (I was gone to ask for sick leave), you see ga a go neele (*he doesn't give you*) the attention e e leng gore motho yo o lwalang (*which a person who is sick should*) should be given. And letsapa la go bereka (*the tiredness from working*), if people work for long hours jaaaka ba (*like*) fa gongwe o fitlhela e le gore nurse o 1 (sometimes you find that the nurse is alone) at night o a tsotswa(*he/she is woken up*) and for ethical reasons ga a kake a re ke chaisitse (*he/she won't say I've knocked off*) he has to attend ko… ko nnetlaneng; the following morning she is what, she is tired but she still has to start work at half-seven (*7:30 am*)… I think gongwe (*maybe*) we need to look at the hours of work for specific areas fa e e leng gore (*where*) the clinic has is only one nurse fa gongwe nurse a runa(*sometimes the nurse runs*) in between 3-4 days and define di-houra tsa tiro (*working hours*) for such areas…<br><br>P1: I think the other dilemma that we have, we are facing ke ya (*of*) receiving these posts of ……….. and so forth kana then when you assess the situation youfind that its neither no, so youfind yourself having to make that decision to say this was right or wrong and when you, the problem comes where you dig deeper you find that this person was doing this for the benefit of the patient but at the same time he finds	

himself doing something, like right noe one nurse was saying "I did it because she took her own vehicle" to transport a patient because one umbulance that was there had referred to the referral facility. So she took her own car to refer this patient o ne a bona le ene gore o (*she saw that she was critical*) critical, in doing so, she had to leave other patients, yone issue ya gore nurse a bo a theogela a le nosi (*this issue that the nurse has to work alone*). She had to leave other patients in the facility, but when she left she she felt gore (*that*) hm-hm I cannot leave the facility e butswe (*open*), I have to lock this facility and ask these patients to sit outside meanwhile I'm taking this patient, so it was an issue to these patients who sat outside, so when asked he said I did communicate with them that I have an emergency I'm taking this patient, I cant leave the clinic open because now they were saying why didn't she lock the pharmacy so she said it's not only the pharmacy that has to be secure, all the amminities within the government setting they have to be secured, so it was a big issue. So we had to engage in molaodi S, to come, to say can you come and help because he re is a situation, so ke tsaya gore because now we are also health workers the complianance did not…. They thought we were favouring this particular person but when we had other stake holders coming in and explaining… so it's a dilemma because we are dealing with situations where you don't know what people are thinking of a performance, it's a dilemma… because you do it, you are wrong, you don't do it you are wrong, because if she had left that patient to die a sa mo isa (*not taking*) then the question was going to be but what else could you do because kana mo (*in*) local government; I don't know whether the ministry of health is going to establishment the same system. In local government when I have a patient o ke batlang go mo refera (*I want to refer*) and the umbulance is not available in time and the case I'm looking at is an emergency I could go to my neighbor and say kana somebody who have just a na le koloi ke mo raya kere (*with a car I would say*) can I use your vehicle ke tsamaya le wena (*I'm going with you*) because I have an emergency case so what it was used to happen is that they were allowed in the local government I think it was working more so that with the number of vehicles that we have nowadays so once the pertson Molaodi has to say ok I will take your patient it means that I will be referring this patient to I will go with the patient and his vehicles but when I leave when I leave my facility I had to actually record re simolola ko go Zero gore ke zero I'm leaving ko Maunatlala I'm going to Palapye primary hospital when I get to Palapye primary hospital I record the millage gore ke gorogile ko Palapye millage ole ha so I'm going to be attended to ke raya molwetse is going to be attended to in Palapye if I'm going to go back with the patient I will but if she is admitted I'm going to leave the patient the same day because I should have taken even the registration number I use the same vehicle to take me back to where to my facility so you also record there so so it was working because it was it was then you get paid because I will send now the claim to the local government and it was fair because kana the local government we doing the payments right there unlike ko go rona go tla twe he! Ko Marina e tswe ko Marina eye ko Ministring etswe ko Ministring ebe eya ko revenue so it was working because a number of fatalities were were avoided by that situation eseng ke emela ambulance e ile Palapye and myself I don't have a vehicle the next vehicle ga kele mo (*when I am in*) Maunatlala yone eko (*whereas it is in*) Gootau nna ke ko (*I am in*) Goosekgweng and those areas so I think they should look in to it and see if they can implement the system ka gore gone gona le gone gore (*because sometimes*) the patient would be written gao kwala (*when you write the*) report gore this one is the patient and this is what this was the situation and this is the vehicle from Mr so and so there is the registration number and the millage	

INT: Ok! Mme we are thankful everyone else, P2… well we are grateful I hope this has taken the whole of your morning and but we hope tota we truly hope that somewhat this can help us to inform and I know the ministry has come up with many many strategies some of the strategies can be implemented using some of the ideas that that you were coming with so I'm hoping this wont be waste of your time now um… this is the end of our discussion here we are going to switch off our recorders and then the eee…..	
