# Supplementary material for: Stakeholders’ Perceptions on Shortage of Healthcare Workers in Primary Healthcare in Botswana: Focus Group Discussions
Source: PLoS One. 2015 Aug 18;10(8):e0135846. doi: 10.1371/journal.pone.0135846 (PMC4540466; doi:10.1371/journal.pone.0135846)
Supplement: S7 Text — (PDF) [file pone.0135846.s007.pdf]

HURAPRIM PROJECT

Participant ID: Focus group one(1)
Date: 28/03/12

Interviewer: Dr N

Interview Duration: 02hrs.09min.19sec

Audio File Name: Focus group Health Workers one(1)

INTRODUCTION

Intro: I'm going to ask you questions about healthcare workers in your area there are no right or wrong answer. Please answer each question in detail your truthful answer will help the ministry of health
To understand the situation of healthcare workers in the country. The first question is:-

INT: What is your understanding of Primary Healthcare? What do you understand about Primary Healthcare?

P1….Do we raise our hands or what?

INT….Yes! You can rise up your hand if you want to answer.

P1: primary healthcare eeee..; the the the main aim of of that is to provide preventive care to the community in a country, they will add more.

P2…. Yeah! I would also say to to provide preventive care to the community not necessarily moving them out from their… to to their…to for from their area so health care has to be acccessible to them the community taking it to them not necessarily them following it maybe long distances… within a radius of 50 kilometres

INT: What are others thinking?

P3: First contact of health service.

INT: Anyone with a different idea?

P2: um! I would want to add that this this was erh erh erh ee! a declaration made sometime I forgotton the year but in Almaatta, it is an Almaatta declaration

INT: Are you satisfied with what what what they they have said? Maybe you want to add something.

P3:We are

INT: so I will move to the next question; studies in Botswana have showed that there is a shortage of healthcare workers in primary care, especially in rural areas. Do you think there are enough or not enough healthcare workers in Botswana? If there are not enough why do you think it is so?

P4: (laughs)

INT:Do you think there is a shortage of healthcare in Botswana

P4: yes, there is shortage of healthcare workers like doctors, cadres like doctors, nurses, most of the the doctors that botswana send to other countries most of them they don't return back home to to come work here may be is due to the incentives in it the government of Botswana is providing to to them. like there was a time when most of ba… the nurses they migrated to European countries I really know if it was around 2000 or 1999 somewhere there they left saying that they going for greener pastures outside the country

P5: I can say there are… there is shortage of eee…health workers especially in rural areas because you find that eee…conditions working conditions are very poor and also eee… cadres like phamarcists they are not usually posted in rural areas and this thing at the end of the day the nurses end up doing the the pharmacy work, so at times when they are there they have many challenges like being there in rural areas they have to work for 24hrs if it is a clinic with maternity for example the nurse will be there working day and night after half past four that particular nurse is supposed to be on call and the following day that particular nurse is supposed to resume duty, so such things make nurses for example to be demotivated and end up leaving the job for

greener pastures or ending up working in private sectors where they are good conditions.

P6: Also. Yeah I would like to also to give you that there is shortage of healthcare workers especially in rural areas like they said but I think generally the problem that is that cause that is the general welfare of the workers like in rural areas accomodation there is no accomodation and generally hela their welfare is not taken into consideration so like we we have already said they will look for other better opportunities out there

P1: also to add on what they have said you will find that there… our institutions here for training they just take a eee... minimum number for instance if you take dental therapists you will find that they take maybe after 18 months they will take 6 trainees to go and train for that including pharmacy technician, electricain so that that one alone it will make shortage of staff because after the training you will see that those who are trained there are put in may be towels of bigger villages and then in the periphery there will be no one going there and then also the transportations in rural areas, lack of electricity eee… though now the current the government is improving on that but you will find that most of the people they don't want to go there because they will feel like they will feel that they want to to to to to to to continue with their educations after their study so you find that when there are in rural areas there will be no internet where they can do their researches so you find that most of them they will come in to towns so that they they study they study further.

Int: Alright! In other case you are saying there are not trained in adequate numbers.

P1: yes! There are taking a minimum eee… minimum number is not enough to train 6 people in in 18 months.

P2: and then this minimum number that P1 mentions it will end up now doing what P5 has said that now other cadres will have to stand in for a different cadre wa bona nurses will now stand for a Pharmacists, for a lab technician, for may be a dental therapist there you know finding a nurse doing all of this which will end up now causing demoralization on these these particular nurse

and now will go for further for for greener pastures like they have already mentioned now kana you will find that these research akere our answers will differ basing on how you have long been how long you have been in the the system, I myself has been in the system,i have been i have been in the system for i would say for quite a long time such that now I would say that our rural areas yes at the moment they are completely different from how they used to be when I was very young in the the system now I would say they are almost and were not the deep deep ones because those ones I do not know but the ones that are used to say they are rural they have so much improved that now transport is no longer an issue like coming from here to Kasane is no longer like it used to be in the past transport system has improved and the even how the the facilities are at the moment they have improved the only problem is these shortage of these other cadres that will now overstretch the nurse and the nurse ends up you know giving up and going

P3: then the other thing if you have eee… certain qualifications like degree or masters that thing… what I have observed is is not beenrecognized there are no incentives to motivatre you as an officer to say this one has a degree this one has mastere in public health you are like any other officer .Yes, they will say nursing is nursing it doesn't change yet you have eee..Qualifications tse dirileng. So this thing in which nurses or I mean nurses are not recognized when they have done certain courses it eee… demotivates us so that's why people end up leaving for greener pastures where they will be recognized.

Inter: you wanted to add something.

P4: Mmm…the other that discourage health workers not to work in rural areas is bad roads and long staying to that rural area where they are no proper schools for their kids there are no shops for you to go for shopping it means that you have to travel with the ambulance to go and do your shopping once in a month. So those things also they do discourage other health workers other health workers to go and work in those rural areas then that means that they have to everything has to be there for easy access of those health workers.

P1: Also you will find that the re…re… renumeration part of it you find that as you asre working as a midwife and a registered just a mere registered nurse

you will just get the same salary but onc…once you are in practical areas you will find that eee… you will be doing all the work in the facility consulting, dispensing, eee…bleeding everyday and the registered nurse will doing a different work from you as a Midwife you are doing but at the end of the day end month you get the same salary so that one alone the government does not recognize your post qualification so that may be will be given a better renumeration.

P2: and this eee!… this thing of us getting the same salary even if you are a midwife or what it has now even contributed further on on on this issue of shortage of midwives because now the younger nurses do not want to go for midwifery because it is not being payed for, you see so as there is this altruision while while as as midwives we live the service and go for other for greener pastures or retire now the feeling part of it is not there because we do not get as many numbers for students who are going for midwifery as in the past because they know that aah… I'm going for midwifery and from there I will not going to be you know renu… re… renumerated accordingly.

P3: again adding on what P2 have said in midwifery you find that for those who have long qualified as midwives some of them are no longer registering because they feel that it is not important to register it midwives because there are no renumerations at the end of they they will be working as registered nurses as a result of that in rural areas if one is working as a registered nurse, eee!… is not enough because it will contribute to shortage in the facility because they will be no midwife because if there is a midwife who has a midwifery and a registered nursing , then the services of the patients will be improved.

Int: what about the distributions are they distributed to certain areas equally or to different areas equally?

P3: Yes I will say they are distributed equally but the thing will be that even if they are distributed equally when they get there from there will be a particular area where where they will be now leaving the service in most numbers like those in rural areas they will leave, more of them will leave as compared to the ones in the in urban areas and like P2 has said that you are

there as a midwife you will be on call the whole night the following morning you are expected to be on duty you can be on duty 15 days continuous so this makes this ends up making you to to leave the job even if ke raya gore this have an impact on the distribution that was done fairly from the beginning.

P4: Mmm…I will tend to differ with what P3 is saying because you find that hospitals are like they are given priority over the clinics most of the nurses and doctors are posted at the hospitals and you find that the primary health is done mostly in the clinics then this other hospitals we the the clinics they do refer us to to to the hospitals so I will say that there is no the distribution is not balanced.

P1: Mmm… Again you will find that mmm… when it comes eee… promotion you find that like P3 said you will find that more nurses or more health workers working at the hospitals they are given promotion unlike in clinics and again you find again that at rural areas eee… the structure itself ee.. You will find that somebody my matron will be staying ninety kilometres away from where I'm working as eee... a …eee… eee… her surbodinate so you will find that in that case I will be doing all the administrative work eee… handling the patients this side the community like we I said we are dealing with preventive kana you have to go in to the community to give health education so that they know gore how can they handle their conditions better so you will find that you are over… eee… over eee… over… eee… loaded with all this work mean while a nurse at the hospital you will find there if she is just working in the maternity is just there in the martenity area or if its in a ward will be just confined in the ward, wena you will be running all all… in in…to the schools, eee… the community the districts everything will be doing that as eee…that one alone also causes the the nurses not to feel like working in rural areas they will rather resign to go may be come to the the hospitals or work in towns where ee… where there will be less work and the promotion at the end of the day is given to the to the hospitals and be will be just left there even there further training most of the nurses who are being further trained you will find that those ones who who have gone for their further training there are in the hospital… you will find that if if you can take the numbers at the in in clinics in rural areas only nurses only few who have done the

degree most of them are eee… on diploma level the government doesn't train nurse… nurses for local local authorities.

INT: Alright what are… are others thinking? You are saying there is nothing that they that binds them to work at the rural areas. Is that what you were saying?

P7: Yeah! Generally the incentives are not there and as for the doctors most of them like P4 was saying they don't come back when they finished training is because when we we come back for to work here in Botswana then further training is like a no no for us most of us who come back immediately up to now we have not trained further and we do'n have any post graduate that post graduate courses that we have done and I think may be is because we don't have a university here immediatelty you try and aaa… look for schools outside its very difficult to leave and go and if you are working in in rural areas like they were saying there is no communication even if when schools are out you can't see newspapers there is no internet so you just sit there and just wait and hope someone will tell you what to do next and like its already been mentioned your bosses or seniors are further away from you they don't really know if you are excelling or they don't even or I don't if they really try to recognize that you are excelling or that you are doing something that needs some incentives like promotion if your matron is in Gaborone and you are or in Molepolole and you are in Gatsalatladi for example you are sitting there working you don't know when they they they are submissins for promotion you here that ooh! so and so is promoted last month and you didn't even here about that that you could actually ask for submissions for your promotions so its generally the incentives that make people don't wanna work in rural areas even if they are posted there they will go then and within a month or so you will see that they are going away resigning, taking…or going to other countries or going to private sectors.

P1: eee… at the same time, you know the government also doesn't recognize the the social aspect of the the workers because right now may be I will be working in Nxauxau eee… whre as my husband is working in Francistown that one alone that distance when you write a letter to say I kana now I'm married I want to move closer to my husband they will tell you that you don't

have a post there you will stay there a distance of a thousand and something kilometres so at the end… so… eee… eee…you end up resigning so that you go and seek work which is closer to your husband even other health workers they feels that the schools at the rural ares there are not enough so if I'm I'm staying there with my children going to these schools where whereby these these children are always failing I will just feel that I cannot put my children in these schools that there is … there is no… mmm… there is… there is no productivity or these school these… school… the… these school… all the children are failing at the end of of the year what is the future of my my educ.. eee… education of my children so I will end up living the rural areas coming to Gaborone so that iput my my children in private schools.

P2: Mmm… you know we are working with expatriates as as we interact with them in our work area so they always tell us that in their areas the their policies that governs these the the welfare of the workers whereby they will be a policy saying this that if you are married if your husband is tranfered from from one area to the other they will…automatically they will just look at whether your husband is going if you are a nurse is there a hospital there is there a clinic there automatically you will be transferred wa bona so that helps retaining the staff but now in our in our setup where will be told that your services are needed as wife in Kasane and your husband is in goodhope you end up resigning like P1 said. So if there was a policy on that issue may be people will stay in rural areas so this one is one of the main reason why people leave where you cannot stay apart from your… lets… long distances from where you are and where your family is.

Int: Alright! Can I move to the next question?

P3: Mmm… in the past eee… they used to be something like RESA Remote Area what what allowance, so that allowance was very little so it was an incentive but the renumerations were very little so it means that with such a small amount it cannot to motivate officers to go and work in those remote areas as a result of that people they tend to be a sta… a staff turn over.

INT: Can I move to the next question alright! Do you think there are gaps, issues or problems related to healthcare workers for primary healthcare or

not? If there are gaps or problems why do you think are the most gaps, issues and problems? Are there any gaps or problems related to healthcare workers?

P1: Mmm…really there are gaps because eee… like like I I I said said as a primary healthcare provider eee… you you are charged with the responsibility of preventing their availing conditions but you will find that most of the time we are we are curing we are treating so you won't have time to go to the community to empower them with knowledge to see how they are living and to change their ways of living into a better hygiene so that the conditions the communicable the disease you you reduce the number…the number of them because wena you will be only confined in curing in the morning the the facility will be full will be consulting giving treatment. It doesn't help because you give treatment when when the roots of the problem is in in the community so curing really it doesn't help so you have to be in the community giving them health education so that they start to change their ways of life to the better of their health.

P2: Mmm… I will say this shortage of staff that you have been talking about also makes us to… it leads to improper improper use or improper utilization of staff that we have like we have the this family welfare… the hears they are now called hears we have those in the clinics by right this people should be going out in to the community to to give education that will prevent this communicable diseases. Now because of shortage of staff we sought of retain them in the clinics so that eee… they help somewhere in the clinic may be with child welfare and and the rest but we know they supposed to be going to the community to give the education but we hope it will improve because we have just had worshops and talked about it and from here we are now going to try so that they go out into into the community to do what they are supposed to do which will end up preventing all this other conditions rather than the…community coming in to the com… in to the vill… in to the clinic now being sick (pauses) so lack of stuff will lead to improper utilization of the few that we have.

INT: Anyone with a different view?

P3: Mmm… nna I will talk about the training you find that most of the nurses,

doctors are not trained on public health because as primary healthcare providers I feel that eee… for one to be effective and efficient should have either public health nursing a doctor serving public health and as a result of that who will provide primary healthcare eee… in an effective manner.

INT: Anyone who wants to add something.

INT: Alright! Are eee… healthcare workers assigned the job there are trained for?

P2: NOT necessarily as you go up the ladder you become an ad… administrator you work as a young nurse being hands on doing what you are trained for as a general nurse will be injecting, dressing doing what you are trained for as you go up now you become an administrator that you know you have to make others do the job and you find that maybe you are a midwife you had this love for midwifery and you really wanted to be in maternity but you will have to go out from the martenity ward and maybe now run the hospital. And ob… and obviously that will go with the the passion the passion for what we are doing maybe what now you going out to do you are less passionate about it compared to when you were in maternity and that we have long said at one point the government had talk had talked about parallel progression where I will just progress throughout the ladder but still be in martenity ward until I retire it was even adv… I mean the government was even advised to do that at one point we don't know where it ended but that would retain the stuff because you won't neccesarily have to be uprooted from your area of interest to somewhere else. So you will find that psychiatric eee… patient is in a general ward a midwife is in out-patient ward or or or… some… something.

P8: I have to to add on that like us lay con… consellors we firstly we were trained to do counseling on pregnant women and partners but now what it is happening we do all sought of counseling even yet we are not trained on that like rape cases like social problems if they come they are just been referred to the Caraven gore just go and see the lay councilors they will counsel you so now it's a big big problem. Because imagine someone came being raped you wound't know what you are supposed to do nna my interest will be bring your

finger so that I can bleed it and test you.

P7: Yeah! I think P8 is right even sometime they are they are requested to distribute milk to…

INT 2: Milk!!!

P7: Ee… sometime because of that shortage there is no one to give milk to PMTCT (interrupted) there is no one who gives milk to to ko PMTCT

P8: Mmm… yeah! We are the ones who are dispensing it, the formula

P7: so its there like the shortage it goes back to the shortage and like P8 was saying we don't have even in clinics we don't have psychologists so those are the people clinical psychologists maybe those are people who be assisting us most of the time but we end up even in social sometimes social workers is based in the clinics and were brought in for for HIV adherence mainly but now we have we been using them for clinical cases where we need counseling we are forced to refer them to social workers to do thst counseling and even that at the end it doesn't really complete the help you wanted to giving to the patient.

P1: Again you will find that mmm… in rural areas due like P8 said shortage of stuff kana that is the the the main issue there you will find that a registered nurse just a mere registered nurse should be put 50 kilometers where she is serving eee.. More than a thousand of people in the community she doesn't have extra qualification like midwifery so this people in that community will have to eee... wait for a midwife who is coming once a month to visit them at times those trips will will fail because these midwife she is the one that eee… my collegue here mentioned that she will be there 24/7 on call so tomorrow when she is supposed to go to Bodikwa she is tired and those poor will spend the whole day waiting there and will be told that the the nurse is not coming so that one causes the the people to to to suffer to suffer a lot because there are not receiving the the service that they are supposesd to be receiving. Ee… so even when when the government is is eee… giving out eee… the stuff they should look into the the distance of the community the community to say is is this health post need a midwife or just a eee…. Eee… Mere registerd nurse

Eee... Because at times you will find that eee… eee… also the midwife will feel that aah! To go and stay at a health post that I will only be maybe seeing 10 patients a day but distance also counts for the community also so that they receive a proper care.

INT: I here you are saying there are there is a shortage in certain cadres, which departments do you think there is more shortage?

INT2: Where is the most shortage, Who is in the least supply? (laughs)

P: Midwives, pharmacy… pharmacy technicians, labo… laboratory technicians, lab… laboratory technicians, pharmacologists, dental therapists…mmm… everybody

INT: (laughs) what what do you think could be the case? Of that shortage…

P1 & P2: I have mentioned it…Of of midwives… just mentioned those but particularly of midwives me (nna) I would say is because they are not distributed in their area you find that I I will take a hospital setting because this where have been most of the time, with the DHMT I just have been there for six months so you will find that the whole hospital almost everybody would like maybe senior stuff everyone there is is a midwife but they will not be in maternity ward you will find there maybe general wards surgical ward medical wards administration eye ward and we are we are all midwives. If we can just take round of us and take all of us to maternity then be I mean we will not be talking of of much shortage like we are. Yeah! That is why previously I have said improper utilization of of stuff.

P1: also the the renumertion because if you are eee… registered nurse or midwives you renumeration should be in line with that ee… because if you do jig and gym and at eee... at the end of the day you are been payed like gym (interrupted) I don't know if you understand

P6: the other issue of training is that mmm… how many pharmacy technicians do we have trained pharmacy technicians do we have in Botswana you see that there is less number of those so I think some of these special areas they need to eee…put more people in to training such that they can be balanced with the demand.

P7: mmm… i was gonna say talk about training as well that we have some fewer people trained in some areas that's we… its its difficult for the government to distribute them evenly and they with the may be they were talking about eee… this thing of… where we have a midwife in all the wards maybe for example in Princess Marina and then I think that something that we can contribute towards its you find tha they will be a nurse trained maybe in many different areas mmm… like we have a midwife being trained also as FMP so in the clinics is is gonna be very difficult to distri… to to allocate them properly whether you are going to allocate them as midwife or you are going to allocate them as an FMP so maybe If this can be eradicated where someone so that they can progress in one in one area like you were saying if someone goes from midwifery they can go to midwifery and progress as much as they can in the midwifery field not to be expected in some area at some point maybe after ten years as a midwife you being told go and do FMP (mmm…) when somebody else could have trained as an FMP

P2: Mmm…you can have just started with midwifery diploma go for degree; go for masters' progress in one in one line (mmm…) and like in in you were saying that lat year with doctors there were… it looks like there were many people who did masters in public health last year and most of them were taken in to the ministry when they experienced shortage of doctors there can't they give them those scales the the the… grades and whatever the the the salary scales as eee.. salary grades and have fewer working in the minis… in the ministry and have more people working in the districts becauase you can still have someone working in in these and they can still deliver they can…..

P1: also you will find that in the government spend a lot of money training stuff eee… their post basic training diploma diploma can't we maybe do one dtploma and maybe degree or masters and then you you progress than to have three diplomas like P2 said there are aaa… I think she is a registered nurse eee… a midwife eee… community health nurse FMP what what do we do with such a eee… diploma is just a waste waste of funds and again you find that bone the nursing administrators now they will be taken to to do to do the eee…the payments…

INT2: The payments of what?

P1: payments of of other workers ba dira our like my bosses they do ehh.. the payments for eee… the other workers the industrial class they will be looking at the patients

INT2: What about the administrator?

P1: ene she wants will will be wanting to find the job finished so that ene she will she she… ene she will know how many are present how many days did they work and then (interrupted: she will sign) she will just do that and give to the administrator so that administrator now makes the full payment and then you see so there are diverted from what they are been trained for ee… they will be looking for files so eee…l…… let me think

P2: we should be looking at what something is being official or is just been done out of human i I mean i… I mean the attitude maybe maybe if I like carrying files around is because is less work for me then I would opt to go for that but officially mm… people are not supposed to be doing that ad… administrators are supposed to be doing his work A-Z and the nurse should be doing her work A-Z.

INT2: So maybe I don't understand is it eee… is that part of your job description

P2: that's that's what I meant by saying it comes maybe out of human something it is not part of the the the job description

INT2: OK!

P1: what they are doing is and they are forced in doing that that is what is happening

P7: even like P8 was saying for the lay councilors um… like in our ke Nkoyaphiri in IDCC is like they they are helping to run the clinic they helping to take out the files for the patients maybe during appointments dates helping the eee… carrying those files like they they take out files from the cabinet arrange them properly take them to the doctors those those are the things that they have to do because they wouldn't be they are no people there there is only one data clerk for the whole clinic so you will find that they are

supposed to to do such things or sitting in with doctors sometimes because some doctors maybe that they need such attention at times so they are diverted from their duties that they were initially trained for because of the shortage

P1: and you know you know even this eee… are… our political arenas in the country you know they don't take eee… health workers as the people that you know they are providing healthand they should know their areas you find that this things are they are being menrtioning at the kgotlas to say if there are no drugs eee…the nurs… yes… that one we we want our patients not to be test around but if there are… if I don't have drugs in extension 15 that's not my duty to go to central medical stores but now we are forced to say if there are no drugs go to central medical stores you can't order drugs bring the drugs, I mean that one it diverts me from from my area how should I be helping patients now I have I'm now on eee… pharmacy technician site you see gone ok! We appreciate a eee… shortage of pharmacists eee… but at times we are doing something you find that they are so many pharmacy tech… technicians in hospitals but they are not deployed to the eee… to the clinics especially in the rural areas wena as a nurse in the rural areas you will be doing all the these work even the eee… the compiling of the reports you know keeping of the oh… oh… the records everything that's why it forces you to run to run away fom the rural areas and atleast to come and work in towns

P2: and to prevent this we believe that the government look into the social welfare workers eee… in the ruaral areas so as to retain them otherwise the shortage will just st… you know until you return and come back to work

INT: Alright! Is there adequate support for for your job, the resources, coordination and management? Look at the the support…

P2: what kind of support

INT: I mean are there enough resources and coordination and management?

P2: mmmh…the resources is eee a problem equipment very difficult to work the number of patients have increased drastically from how they used to be

when when we were still young nurses in the past they would not be… like when you say the ward will be full you will be 30 patients nowadays you find that female medical ward will have got something like about 70 and we are using the manual B.P machine where you are supposed to here and you know pumping like this for 70 patients at the end of the day its… you are done so lack of equipments and the other ones is very old you order it will take a long time to come you know yet you are supposesd to provide quality how? The the the the thermometres that are some are very light and fancy but the way they are done you find that probably this are meant to check four five patients per day in a private clinic you know but you have 70 patients in the morning by the end of the day they are… not working… you see so the equipment is really a problem

P3: and then the other thing shortage of transport in the rural areas you find that if there is a problem with the ambulance for example you take it to CTO there is no replacement yet you are forced to refer patients for better management so you become frustrated if you have a critical patient and who want transfer to be transferred to to a higher facility so you end up leaving the job because if you are frustrated you can work properly

P2: and the logistics surrounding this the ambulance will be there and you will be told that administratively it is not supposed to move from this speedometre is not suppose to move because if it does the CT the CTO officer if you can meet along the way he is going to take it because it will have gone over the the millage that it was not supposed to do you see this logistics and yet you have a patient here who have to take to the to a eee… to a higher facility they will be so many ambulances there parked because of those speedometer gore its not suppose to move it will be taken there there at the CTO and yet you are grounded

INT: I I don’t understand what happening does that mean that you are saying in a month you can’t do more than 100km if you reach 100 it has to come or is all or is that the speed

P2: some something like that… the administrator can…

P6: yeah! you know like they have they are saying they have their bench mark

for for bigger vehicles they have to reach between… every time they reach 20 thousand the have to go for for service and they will be there the the vehicle will be there atleast 20 thousand kilometers for us you book for service at CTO they will tell you no this vehicle like especially the Quantum ones they are still serviced by private companies they will tell you no is still owe motor center so you can't take it from motor center it can't leave motor center so if you take it there they will get yeah! So eee… just park it there until you manage to pay motor center

INT: (Laughs)

P3: when you talk of that ambulances are not given priority they are just treated as like any other eee… vehicles which goes there to CTO so such things tota eee... we are not satisfied with with the plan they have at CTO

P1: Again you will find that the ambulance that that they are providing mmm… eee… as as a nurse when you are escorting a patient eee… you have to to stay at the back with the patient than to um… monitoring you find that in a van that it it has no eee… should I say a couch or a bed for the patient and wena as a nurse you don't have a chair but you will be monitoring the patient going hundred kilometers to take the patient maybe on the way the patient deliver ou know at the back of so… I think they should provide eee… a van that is um… really user friendly ee… again I was going to say that we don't have a a support as a health workers from… from our community and eee… our politicians or our political leaders because as a as a nurse we are meant to work less like those slaves that were there in those olden olden days and that force people to move away from Africa to from other countries to Africa so because you find that right now when this people they go around addressing they don't look at the health workers side they are only looking at the community''s side because they now eee… ar are after eee… eee… at the end of the day this people are the ones who are going to give them elections to be in in that power so they will they are going to tell them that a a nurse is is meant to to work so if that nurse he doesn't work or she tell you that she can't help you go and and report her they don't come to see the cause of the problem why is the nurse maybe saying that the people they should do this or they should come at this time or or they should do what because wena you

will be working 24/7 when you saying I'm breaking for lunch the community when the parliamentary council to say she leaves us to go to her house she doesn't she doesn't eee… consult us to give us the medications whiles they don't know gore wena at work you know I just felt when when I when I was working in Etsha 6 that during Malaria era you would just feel for a nurse a midwife in that clinic working 24/7 and at night this malaria patient malaria it becomes worse at night they will come and the delivery those communities they are delivering my dear they they are bearing children so a.. a.. at night you will be working 24/7 and in the morning you will be expected to go to the the SRH program to to do the ANH seeing children and you will be tired and when you say let me just go and rest fro an hour you will just be be called at the there is a telephone call from from DHT may be the district commissioner will be phoned to say the people they are saying there are in queue but you are at your place not looking at the fact that how you are working at night and the like so our politicals are really they are only on the community side than you as a health worker .

P6: yeah! I think the other problem is eee… taking the DHMT to be under the hospitals like like now there there is a lot of channeling in taking some resources like tsone transport and may be some like protective clothing because like Gaborone DHMT they will have to order from Marina and Marina is the one who is doing the purchasing and everything so the channel between Marina and DHMT it it takes long unlike if DHMT was on its own there doing its purchasing it is responsible for its flirt and everything because I think every if it was done within Gaborone DHMT not having to go through Marina to get everything that you want so the DHMT should be on their own.

P7: even that you were saying about transport sometimes we had ambulances coming from our clinics in Mogoditshane they will maybe referring the patient and when they are coming back they will be told that this ambulance we are taking it it is going somewhere maybe is taking the patient to Nyangabwe maybe is going to South Africa and (someone coughs) so it means that unplanned you don't have transport you don't have ambulance for that trip and even minor things like basic drugs for critical patient is a problem you are told you are on call in Lesirane you are there at night

someone comes bleeding you can't even arrest the bleeding with just a needle and like a scissor is not there and you have to send the patient to Marina when they get there the medical officer in the ward in the in ENE they will scru…they will crucify you… they will tell you all soughts of things… to.. whoever is referring the patient you would be told many things even like we have maternity um… maternity services in Lesirane if you see that this patient cannot deliver here and you send them to labor ward in Marina you get it from them while you are referring that patient so its its if ve… if you are there you just get frustrated you can't help a critical patient when you try to send them to Marina to do to get that care you are crucified what can you do maybe resigning going somewhere else.

P2: but I I think he mentioned taking eee… a long from the DHMT to to to Marina to get something the red tape it's a problem such that ke gore like I said I have just joined the DHMT but what I find there now other problems are man made can I say man made or what um… but what I want to say is like DHMT hee… we are surpervising many clinics and the the communication well within the clinics may be easier with landline but sometimes you you can't get hold of the person you are you want to go through to you need a cellphone you see there is use of cellphones nowadays there has to be an arrangement to where if you can actually pick the callphone and this has to be official one and talk to… to a doctor somewhere to tell him or her about the patient you are having now you find that there is no such facility and you end up using your own cellphone which is wrong but for you to make this arrangements you now have to go through to Marina to arrange that if you had a cellphone in a certain clinic everything takes a long time and mean while how are you operating with the patient so red tape it's a problem and and really affects the operations of the clinics this what I have realized since I in Marina atleast there there is a system put I place whereby if you want the doctor in call from home you go to admin knock and use the official cellphone to to call the doctor but clinic side there is no such arrangements wa bona yet it can be difficult to reach somebody while you are calling and not knowing where the person is and you have to call using th cellphone but having to arrange that even Marina it takes a very long time to report it will take a wrong time before everything is in the ground

P3: then the other thing this system of making primary health care do be under hospital services really eee…the health workers will in primary healthcare are not satisfied with that so you find that um…the matron from the clinic I mean from the hospital say primary healthcare is given those clinics so that he/she can supervise those clinics so health workers working in in those clinics they are not satisfied at all. They say the transfer of us a clinic nurses or as clinic health care workers to the ministry, the mission was that we are going to be supervised by those eee… those officers who were who are working in hospitals so as a result of that of that some end up leaving because they feel that things arenot done correctly . Primary healthcare should stand on its own then a hospitals since they deal with a eee… curative services so they should stand on their own.

INT: what about the medical resources are they enough in hospitals I mean medicine, pills

P1: They are they are ot enough you will find that like right now oh… oh… you will be in one umbrella healthcare and the medical services you will find that even the distribution of that the… they make they make them better for the hospitals thsn the clinics so even if you go ordering those they will tell you that no this this thing is meant for for hospitals is not meant for the clinics so you will find that wena you just go back without anything you see better things in hospitals if you go to the clinics you will find that this they will be pumping the whole day without seeing seeing better results so you see its not enough so oh… we feel that this long structure will be broken up because is is really it brings a a problem like even we said before even the the promotions they are meant for the hospitals they than the clinics and so there is a a disparity…. So maybe this structure le yone they they should look on it with with a close eye because you know a vehicle eee… parking in the its not like a vehicle because that one it will be leaving from point A,B,C D and at the end of the day when it comes out of fuel they will take it to go and fuel it at Sebele go there for for three hours whiles wena at the clinic you you have patients to to to carry to to to the clinics so you will find that the take they take long time to to you don’t what is the problem but they being experienced such that the time there were using this cars they ………..

P4: um… I will say there is a shortage of drugs I don't know if the problem is the supplies and the Siemens or what but initially in while while we were still with the councils there was a vote that we used to purchase drugs from private supplies but now in the central thing it's a problem because you are staying months and months there are no drugs even there are no morality nothing but there in Siemens they will tell you that you can order there is something when you reach there there is nothing

P6: yeah! There is also issue of transport again when it comes to because at CMS there is there are specific vehicles that can transport eee… medication so and you go to Marina and ask for such vehicle with the specifications of CMS and you will take some days to take that vehicle so its another problem

INT: you wanted to add something

P8: I was going to say CMS is a problem CMS they don't tell the truth they will be saying they habe something but they don't have it in store so some of thing they have you order somethings come when they comes is not in your stock then they receive it later when you go there is not there priorities is given to bo Marina and Nyangagbwe referral hospitals and for for purchasing we had a vote in council now its at Marina they buy in Marina after they buy they just give to the wards if there is something left over then they give us or they don't give us at all so everything that we have to if we have to get drugs we have to go to Marina so it's a problem because they are looking at their…at their needs and they forget that they are others outside…

But myself I I I

P7: sometimes they put those drugs long time in in Marina when they get to the clinics sometimes there are near expiring. They was a time when when they are a lot and within a week there were expired

P1: but they will give you what is in the shelf s that is near expiring so bone they get new (interrupted)…. the doctors… (talking all of them)

P2: from the DHMT mmm… my experience with the hospitals is that even in the hospitals ward you will be ordering whatever you don't have an you get half of the product everything from pharmacy will be will out out of order and

it will stay out of order for a very long time so that in the clinics they have this feeling that at hospital there are drugs but now I have just joined the DHMT from since… my mind is still fresh from the hospital even from the hospitals there are no drugs you end up being frustrated when you order anything will not be there you will be waiting for the drug at the end it comes you see

P7: it will be better if you sometimes in Nkoyaphiri… she will go in the morning she can finish a week going to CMS everyday for example right now having you know for three months if I think we have been having drugs and right now we don't have…

P2: sometimes the logistics there in CMS they will be saying the is…. Here so but is not yet entered in to the system therefore it cannot be given to to the facilities this sometimes we here from from them.

P1: again you will find that you know this eee… supervision the structure of the DHMT the local authority it is not good like she introduced herself that she she is supervising four or five facilities wena wena just making myself aaa… ene she will be around in the clinics at my facility in Extension 15 should should be putting me to be the the the supervisors in others whiles I'm on the same level with them you see they will be saying you are the nurse in charge while I'm in the same level with with everyone because ene she will be thinking that ok! I'm just a a a covering for her whilst at Sebele while she is BH3, BH1 what what rank I will be doing her her work at the facility. So you find that the policy are just too short for the for the for for for the for the primary healthcare facilities so for the hospitals each and every unit it have been done by default following the principal registered nur… nurses so at the facilities there they are onlyprincipal registered nurses so ene she will be running around with six facilities

INT: I will move to the next question. Is there a problem of healthcare workers in rural areas if it is so gate if yes why it is so…

INT2: We are aware that you have eee… covered a lot of this already um… but it was there as a question but maybe we could look at eee… some of the probes whethe because…here we are talking about shortage in general but here it was mainly focused on the rural areas but if we feel we have covered

enough that’s fine the number of issues that we thinking about and feel we need to explore in the living conditions I think a lot of you have mentioned that you you and then the remoteness, the places themselves the cost of living um… opportunities for professional development availability of jobs schools for partners and children it was just some of the things that we I know you have covered a quite number of them somebody have something to add otheewise we will pass to the next next question

INT: What do you think should be done about the shortage of healthcare workers for primary healthcare in Botswana?

INT2: now we are looking for solutions we have now found all the problems. What could be the solutions?

P2: If if… let one work for what they have been trained for if you are a midwife then progrerss all that line midwife until she retires.

P1: renumeration let people be renumerated from where they are been trained ee…

P2: and they are given proper accommodation in rural areas even somebody going there will find everything and you arrive in the village and the house is demoralizing so accomodation is not enough.

P3: then the other thing the remote area allowance should be attractive

INT2: it is still there?

P1: YES! It is still there

P3: yes!it is still but is very little

P1: it still there but the remotest that we think it is the remotest

P3: yes! Its very little

P4: Is very little

P2: I think generally is to give the incetives to those in rural in rural areas and aaa… also to have support for the social welfare of the the health workers like I think it will be improving other things other other services like schools

ensuring that they have accomodation like she said the social welfare will include those who have partners those who are married who have spouses and they ensure that they have easy accessibility to to having a a a good family staying together you know that some sometimes is not its very difficult to have someone working in maybe maybe Ntsweletau and the husband services are only available at Tsabong maybe someone can be moved nearer to Mogoditshane so that they are making sure that people stay with their families as as they should...

P1: even flexibilty of transfers you find that you go to ruarl areas you stay there for ten years I stayed ten years in Okavango can you imagine when my family is in a Francistown that one you know is only that by then you you even if you write them telling them thaty there is no place for you to go so they should be flexible enough not to stay for a long time eee… eee… in a aaa… a in most remotest area yeah maybe you stay one year in remotest area then you are removed from a aaa… a remote area even the promotions the post post should be created such that people are being transferred right now if you go to I don't know how many lab technicians are there yo find that we are all of us we are in C1 all of us for many years staying there until you you reach the 12th notch you will be just ther so the the promotions should be there so that people get eee… attracted now if I mean in C1 if they can say P1 you being transferred to Gantsi on promotion I won't refuse because I know gore is money but if they say a aaa.. Transfer yourself to Gantsi on the same scale eeee… I will just tell that no I would rather look for a private practitioner and work there instead of going there on the same scale so so they should look on the the scales

INT2: You talked about incentives I just wondering maybe to follow it up what kind of incetives will will be meaningful and um… I mean we have talked about allowance so for many years but are ther non monetary incentiv es that maybe will make things better

P2: Mmh! Like we said accomodation, proper space for the kids as we go for transfer so that we go to those we go to those ruarl areas and find good education for our kids

P7: even when you are in rural areas I know that when when when its time to further your education you you will be because if you are there you are just there you can't get any communication you ca'nt hear about anything if people are submitted for promotions they don't know one phones you you hear that everything is done you are just there…

P1: even for further training you should be taken for further training um… instead of working for more than 20 years without going for any further training you stay redundant

INT2: The group in Maun and maybe little bit from people from the Mahalapye district because Mahalapye is not just Mahalapye and even Maun they were people from Okavango and… one of the things they have said I just want to see how they is that eee… probably it should be when you want to go to work in those remote areas you should be prioritized for training you should you go there for three years two years but after that you will be on the priority list for training so that if…

P1: yes! That would could be a eee… an incentive because people will be willing to go to remotest areas with with eee… knowledge that they will going for… further training ee… so it will keep people to work there…

INT2: And also the other thing that I think another group said I'm not sure which group it is they should a eee… an element of choice that is I I mean incentives should be there but also they should you shouldn't just wake and say tommorrow fro instance in Maun there is a quite number of people who are in Maun happy to be in Maun because that where their families are but one of them said was transferred to Thamaga or something and or some or so they they were saying they should be an element of choice and so I I just want to pass by here to see if …

All: yes!

P1: that's what I was saying about flexibility of transfer I was meaning in those areas that you choose ee… where you want but maybe if they say transfres you you wena will opt to go to Kweneng other one opt to go to Southern istead of you being just told to go there .

P2: and in the past they used to be this transparency where you just make a list ke gore like in Marina we knew that Marina stuff in Marina they transferd to Maun and whoever goes to Maun is going to stay there for two years and comes back and then the aother one goes just like that. So it was very you you would know when you are going and when you are coming back so if this system could maybe come back it would maybe it can also retain people from there like Athlone those in Athlone will be going to Gantsi so you would even know the queue where you are and that would help you to plan kana next I'm going to Gantsi I'm going to stay there for 2 years and you are coming back to work in Athlone yes, it was great that time

INT: Can I continue…

P3: then, sorry! Then the other thing if say you have transferred to a remote area then you are still negotiating at times our bosses threatens you telling you that your post will be withdrawn as a result of that you tend to terminate the the (laughs) the say you tend to say aah! If the post can be withdrawn it means that I have to look around and lok for another job.

INT: In your experience what solutions/ interventions have been already tried to solve the problem of shortage of healthcare workers

INT2: I think maybe sorry um… X just going back a little I think there were issues may we didn't really quite cover we talked about retention strategies and things and what we should cover how about getting the skill mix some of these things its about getting the right skill mix I know some of you have mentioned that the people are actually not used to what can be done to maybe to make sure that we have the right skill mix so that we don't get lay councillor to now start eee… distributing… um… mashi and also what about task task shifting this are ideas that are throwned around in the in the human resources how much would it apply to us how much can this concepts actually help us to address oue problems. Skill mix means that you know if you know that in in this clinics this are the services that we providing and therefore what skills do we have and therefore decide who should have these skills and how do we make sure that that we have followed the skills that are then required and also task shifting and I think it's a concept that in botswana it is more and

more used in HIV that we brought it to lay councillors so that the maternity the midwife no longer has to spend a lot of time counselling women about the uptake of PMTCT and we know it is also used in nurse prescribers for HIV. It is a concept that should e can be helpful and I think these are some of the things that we want to hear your opinions

All: (laughs) skill mix…

INT: And what I hear more especially from the nuirses or maybe with the lay councillors is what you say is that there insn't a eee… it is not just task shifting it is a task adding can you… I can give that a eee. But should should it si something that we should really look at as a country to say is is is it kind that we actually look at the services and that if it is something that can help us. I know for instance… I'm going to give an example or may be before I give an example

P8: I was gonna say is it can work but they have to be not be a that protecting those people who are going to be receiving additional work and also they should be properly trained of what they had to do from there.

P2: mmh.. you are trained ke gore these side you will be trained to to you know improving on the other side you now destroying whatever you have put in place , I mean, good things something that is supposed to be good now maybe somewhere doing bad things like you talk of task shifting somewhere now us we will be calling it non nursing duties and you you will be requesting for for to be renumerated on that and you will be upping gums with the government you see something with good intentions now leading to some something else so to prevent thses friction it will be best like she is saying then there should be laws governing everybody if you are going to be shifted to this task it has to be up to here and maybe talk about such things that and renumerartion…

INT2: And I think it is importanat and I think task shifting does not say there is no renumeration I think task shifting if its done properly you you will actually save and what are the tasks who is skilled to do that who can for instance the lay councillors were gone generally there were not part of our system so there were brought in because they was a new task or there was a

task that was probably be shifted because the I mean the concept is thisn person could be trained instead of a nurse a midwife who needed five years old of training you can bring someone else and train them and I don't know how long that takes and then they can still do this work well without the profession that really the concept of task shifting and so I ju.. I just want us to say what do we think about it as a concept, for instance in Maun for instance the group they don't have they don't have many nurses at all so they can't task shift the nurses so they task shift to to traditional birth attendants a lot of them they trained them you know they they spend a lot of time training tradional med… birth attendants to do a lot of what nurses will be doing for instance even traditional healers they actually spend a lot of time trainning traditonal healers for specific things so that their level of task shifting so but because servces needs to be provided and we want servicers to be provided um… every Motswana should have the right to expect those services whether they want their babies to be monitored or whether they want to deliver any born baby or whether they have Malaria somebody who who knows what they are doing but looking at what we have because money situation is different from our situation back then so that what the they are forced to do so that they provide the service but what can we do is something that we think we can think about and take it as a concept and inform policy to do something about that effect

P2: yeah! If it done well its its it can be a very good concept like at the moment we have the healthcare auxiliaries, if we look at them they are very good cadre in the system but we diidn't have them in the past they are very good if there were allowed to do what they are supposed to do because at first when they came to the system they were supposed to come bath the patients, feed the patient and turn the patient. These were the three things the healthcare auxilliaries were supposed to do when they came to our system. Now because of these shortage of staff we are using them now to do other things and this three things remain as a problem so if these task shifting was done and strictly on these three and done in a good way it is a very good concept

INT2: And and another example in South Africa I know that eee… the the family nurse practitioners they have been trained and now they have the lot of

tasks that you know it was decided that ok doctors are doing this actually the family nurse practitioners skills are such that they can do things they having been doing it so they are now treated as different cadre I mean they are still nurse practitioners but they are they are renumerated mmh… so this is task shifting and it is working and so their primary healthcare system is strong you find that these family practitioners are actually they provide a lot of the consultation and the trea… and that and the doctors also provide the service so that is really the concept I thought we could actually look at it as something viable in Botswana….most of the of the doctors that ben ts\

INT2: Alright! May we can continue…

INT: In your experience what solutions/ interventions have already been tried to solve thesse problems?

INT2: Nothing! (All laughing) no… i…

P8: They do they do get a um… healthcare workers like other countries when this people come here they are a problem to… first communication they can't provide help to the patient because of communication. How can you provide help to the patient when they are saying is that… they are trained to when you are working with that person you will be doing all the translations at the end of the of the day they get more money than you are getting so you get frustrated as an individual mmm…

P2: what what the government is trying to do it has introduced other cadres that have not been there in the system like have said the healthcare auxilliaries they have not been there, the lay councillors are there that what the government is trying to do and again…they are trying to increase the post it will mean that they are increasing the posts and hire and then altruision rates is still high.

INT2: How high is the altruision rates,im just saying, in your minds for instance if you working in a in a I know its dificult it's a difficult question but I'm just wondering becauses its not really recorded when we were doing bench research we couldn't really find find the numbers at all

P2: we can't…say its high (all laughing)

INT2: For instance in the DHT where you were supervising now for the l;ast six months how on average how many nurses would you say you have lost?

P2: No I don't know because I have just… I don't know but I have the ones that are affected is just retirement not resignation.

INT2: Retirement at what time

P2: early…

P1: people are going on early retirement because eee… they know that coming ton when you are work on on contracts e… e… it has more benefits than when you are just there you know at the service. Because right now whwn when you retire at C… C2 scale when you you are re… when you are coming to work eee… on contracts you will be given C1 notch

INT2: Why?

P1: and after every three years you will be given you will be given your…what is it called? Gratuity yes! That why people are retiring early

INT: Do you think eee…

P1: ee… continue

INT: Do you think perfomance based system could help

INT2: Has helped.

INT: Has helped

INT2: But.. (everybody talking)

P1: that one really has … it has created a lot of problems

INT2: Why?

P4: people are performing but we haven't seen anyone being rewarded for the hard work the person has put on or the initiatives that this person has brought to the society and you are doing your PDP you get ninety something percent at the end we thought this person will be promoted or will be given a something

to show that you are appreciated but nothing

P8: There was a reward from the government last…

P3: yeah! Last year…

P8: mmm… last year

P3: it was it was eee… an initiative as way of improving performance in the healthcare system so it is going to be an annual event maybe people will be motivated.

All: but really like you were saying…

P2: performance based reward system it's a good syatem if it is done rightly or done in the right way. It is only that now supervisors we are not doing what we were supposed to be doing you will know that this person is not supposed to get sixty percent because surpevision is not really… how it is supposed to be hela the system is supposed to be a good system but because of that people endup getting wrong marks and reflecting wron… wrong reflecting wrongly

P1: again how do you how… how… how do you give me marks ma… ma…when I see you once a month and you just come for fe minutes when you find me with patients you just say I came to check on you how are you, and then you go away how do you give marks it becomes biaseness ee… Supervisor Y will give me marks because she know gore atleast Surbodinate Z at her clinic when she is there I don't here eee… many queries eee… at the facility so let me give her ninety five percent while she doesn't she doesn't supervise me.

INT2: You were saying something

P7: I was saying that P1 was saying that evaluation akere we evaluate sometimes you that someone doesn't work she comes late everyday actually you are going to give them ninety percent like someone who is is doing everything that shold be done and then the the system tht she was talking about like we were saying you will be sittingb there in the remote area who is going to give who is going to forward your name to be rewarded the way are working because if some if your surpervisors are in the greater greater

Gaborone area and you are in Lentsweletau I you how are you going to be seen that you are doing something when maybe they visit you once in six months or once in three months and all they hear is when you are referring a patient to Marina someone is complaining that there is no nurse in the clinic how are you going to be in the list of people who are been rewarded I think that system it can work but its just to the one you were talikng about it just too big it it entails a lot of things I think if they can break it down just just to cate… maybe in smaller categories it could work. The PVRS I don't think its working some facilities are not doing… I was saying Nyangabwe I never did I never did PVRS I was in Marina I never did PVRS I did it when I went to the clinics so some faciklities are not doing. Even in the same facilities some some ward or some departments are not doing but still even in those areas that they are not doing them you still be taken for training and being promoted unlike someone else in their wards trying to how can I do for PVRS and yet they are not being rewarded for that so its really not working frommy my perspective.

INT: what about moving primary care from Ministry of Local Government to Ministry of Health?

P1: that's that's one was a disaster my brother (all laughing)

P2: it created a lo… a full of what to be done but staff not being increased ke gore you find that everybody wants eee… local government their files are taken to Marina and yet staff in Marina they were not increased..

P7: i even though that we had administrators in DHMT I was wondering why why were our files were taken to Marina because even if you have a problem you… its its never solved. For you to get your files you have maybe to stay in Marina and sit sit there the whole day or the whole week say that I'm not going until you find my files that's what its works nowadays so it created a lot of problems like we were saying about the if your Mmopane tra… ambulance that it has been in Mmopane for many years comes to Marina maybe to to to refer a patient sometimes they take it it happens a lot of times with Nkoyaphiri ambulance they always takes it that it is going to Jorburg and they won't tell you on time it will be going there for something and when it gets there they

say this one is remaining here and they won't give you anything and the nurse will have to find a way of going back even the driver going back to the clinic to to report that the ambulance it has been taken away

P8: Also… also it has resulted in loss of staff because like in ARV clinics they used to be social workers they were doing counselling there areb from local government, local government has taken them now there is nobody counselling the nurses have to do everything now so it's a problem

P1: including revenue collectors right now at the at the clinics we don't have revenue collectors. So so the government money is just going away because it was just neglect to get those five pulas from the from the people but it was helping maybe to purchase even the the stationery with that five pula you see but now people are not paying at the clinics. They are been only… paying in Marina this this moving eee… primary healthcare to to hospital services it has created a no no communication channel mmm… of which at the end of the day is useless and is putting eee… the nurses at the clinics as if they are not doing their work because if don't have you don't have equi… equipments, drugs and how how are you going to work at the end of the day. So people when they go to the facility they are not drugs they are not given drugs that's why you find that Ministry of Health everyday is on papers people are complaining is causing a lot of people complaining of our our poor services because you… you… to channel a thing I have to I have I start with her then go to her then go to oo… DHT and there is another one on top of the DHT and then Marina and from Marina I go to Ministry of Health the channel is just too long and again it has caused a lot of autocracy. Now we are receiving the orders we are not part and part of the people who who who who can come up with ideas and how we can improve our services is autocratic we are told you would do this or if you don't want you explain why you don't do it that is eee… autocratic order when I say why do you come up with this you tell me this is from above who you see… (everybody laughs)

P2: so this thing of of eee… merging came with with its own problems like a lot of nurses from DHMT they were accomodated by the by the council now they now that they moved to Ministry of Health the council now is saying bring back our houses so it has created a lot of problem on their

accomodations side which we have said is one of those strategies that can retain nurses now the council houses have to be returned and they are doing so even the office … mma…even the offices they want the office space so this also the other problem that has been createdf by this by by this.

P6: moreover I think this issue of greater Gaborone taking clinics which were in Kweneng area and putting them under Gaborone and eventually under Marina and in Tlokweng and those which were used to be under Ramotswa are uner Gaborone now so it means a lot of work it means a lot of work … because eee… all all this eee… officers from Kweneng east area eee… Mokolodi, Tlokweng area they bring eee… their grievances to Marina you know its a lot

P7: the services at Marina were not increased

P6: yeah!

P7: and and they are still suffering the

P6: if it was only Gaborone no this this Kweneng eee… those which eee… were under eee… Ramotswa

P7: even what she was saying making for us like making day to day schedule when maybe on a Monday you will be told no you suppose to schedule your doctors like this because when we schedule them this waty its not working you have to change the schedule if you ask the the the the Dhmt doctors you will be giving us this doctors this is from PI's and the directors so we don't have anything to say even when you know that this thing have been working for us when you were under the the the the council we try to tell them you know that one is not working we want to make everything like since January the way we have been scheduling doctors we have changed about five times if if we have a way of scheduling doctors so that we can cover for… if we cover eight where we want to cover eight it means that we don't visit other cadres and then they complain ing about other clinics the Ministry the ministry tell us we have to change the way we are scheduling doctors so its just like that we don't have any say we we don't have any say of of improving where we are working someone is sitting there visiting us not telling us how to improve it

and it never works.

P6: and and the other thing is distribution of resources like like maybe ambulances like maybe in Extension 14 they used to have an ambulance and you will be told that because Lesirane is buzier than Extension 14 the ambulance from Extension 14 taken to Lesirane now you wonder gore how because even Extension 14 they they have to refer to Marina so why is the use… because… so it also affected the issue of distributing of resources

P3: then the other thing we have a former undustrial class of workers their payments have been affected especially overtime so you will find that they go up to 2 to 3 months without getting overtime so as a result of that they are always crying demotivated and this thing at the end of the day they can affect performance.

P1: again you find that you know we I our industrial class especially cleaners they were called general assistants because they helping us to with the work, cooking, laundry.

INT2: cooking?

P1: Yes INT2 and cleaning

P4: in maternity

P1: yes! For cleaning for cooking for patients not

INT2: I find I thought there was ooh! You mean at the clinics

All: yes!

INT2: Ok!

P1: yes! Right now when this merging they are now called cleaners when they are being called cleaners now they say we are no longer laundering we are no longer cooking so you find that now they will be no one cooking at worst maternity. So the patients who will be coming will asking them to bring them foods to come and eat during during labour or after labour. so you will find that it is not a good for them to do that and you will end up putting a the patient is fainting at the night Marina kitchen is closed and they will tell you

again in Marina that you don't transport food in and out ambulance it is not hygienic so you have to find a vehicle in Marina to tale food to G-West and you will stay 2 days without food ko G-west you see its really a problem.

P8: Yes! And to talk and to add on that you know us as lay councillors we we work on contracts so what happens now is that you with the the ministry of health they take too long to pay our gratuities akere we are still waiting for gratuities from last August sometimes when it comes to month end the salary will skip and they will tell you gore just wait for the next for the next month we will pay you or take a week or two you will be payed still in the salary they will never

P7: if they skip your salary and inform them

P8: they will do the cheque eee…

P7: so that they pay you

But with Marina I know they don't care Marina even I can say Marina or who or Ministry they don't care they will just say nna mmm… we can't solve your problem just wait for next month

P7: and maybe go somewhere to the ministry in the or morning to look for your salary he is working at the clinic and it creates more shortage

INT: Can I continue or someone…mmm… what about introduction of medical school programmes and faculty in rural areas for primary healthcare will that help?

INT2: Come again.

INT: Eee… introduction of medical schools programmes and faculty in rural areas for primaru care

P4: what do you mean by programmes?

INT: we mean eee… the schools where people can be trained to be doctors or nurses or if they are … they are introduced in rural areas could that help the this problem of shortage of healthcare workers

All: the schools, or or doctors being posted to to ooo…

INT: I mean the Schools if there are introduced at the rural areas will that help with the problem of healthcare workers in the rural areas

P7: do you mean…

INT2: Alright! I think maybe to just help a little bit I think part of the school of Medicine actually this sss… sites to I don't know if you call rural sites are already are already part of the training for instance we have training sites in Mahalapye where we have already quite a lot of trainees this are post graduates doctors who are being trained speciality in in family medicine and so we have we have those in who are teachers who are doctors else providing care and more in hospitals than in clinics who have undergraduate medical students and Maun is the same so I guess is something that i… maybe for people in Mahalapye and in Maun see it because they see them and but maybe for you here you may not see it but the thing is do you think is something that might help

All: it can

P2: it can because it will reduce the number of referrals maybe to to to Marina.

P7: if if you are in those areas you want to train you know that you are going to obviously you have to come to Gaborone to to come be in medical school but if they are in the rural areas even if you want to go to medical school for example I have my kids I'm staying there they are going to schools around there sometimes it takes even from not wanting to go for example if I want to train I have to move everything so its its if I know that I will just be trained maybe in Molepolole Sccotish I will still be where I'm working like those in Scottish and …

INT2: And I think what was actually interesting was much of the people who were in the programmes now they come from around Okavango Maun and also around Mahalapye Serowe a lot of people being in this programme

P2: if we can study it and continue or leave it akere our problem if we can contiunue with it because…

INT2: Hopefully UB has already had a lot of infrastructure, internets this thing and other systems in this places

INT: what interventions will make the biggest difference in primary healthcare interventions?

INT2: This is your opinions we talked about a lot of things but what we think will make the biggest impact than if could really do this th magic bullet (laughs)

P2: improve the welfare of the workers there to keep them there so that they can stress on the preventive the permanent healthcare service p3: then the other thing primary healthcare should stand on its own not being merged in with eee… hospital.

P6: and give the maybe the the DHMT coordinator the the authority to like eee… do what he thinks its best for the DHMT should be given the autho… the management there should be given the authority

INT: Anything else

INT: Building of effective primary healthcare teams have being suggested as potential interventions to improve primary care in Botswana. What is our understanding of effective primary care team?

P2: primay primary what? Primary healthcare team

INT: Yes…

P2: I take it that it will be a team that is supervising to see that the the healthcare has been running… it runs smoothly and effectively

P7: You mean the composition?

INT2: What we understand what yeah we can talk anybody any of those even the composition can work

: I just thinking (laughs)

P7: I think… I think I would say a doctor, a nurse we need that now that we hve lay councillors and they have worked a lot we need um… not just eee… a nurse a nurse should be not just a general… registered nurse maybe those defined professionals that they have especially a midwife they will their problems with the with the pregnant women, mothers not been helped properly because the the people who were there they were not well trained we also need pharmacy technicians we need social workers gender duty assistants she she mentione earlier the the

P2: and and I will add that treasures as the community

INT2: What about?

P7: INT2

INT2: What about administrators

All: yes!!!

P2: Administrators should be in the team you can't run yourself

P4: and supplies officers

P3: then the other thing kana when youn are providing midwifery care it's a… I mean when during pregnancy we should provide primay healthcare to that woman so I feel that eee… midwifery they should be eee… a midwife who is head of the midwifery either at the district level or at sub district level so that particular officer will be responsible for a midwifery care but in our situation we don't have anything like head of midwifery but it's a… safe motherhood ee… I take it that it's a very important ee…

P1: more especially in the ministry eee…

P3: Yes!

P1: level only

INT2: Yeah! I think one of the things that we also want to explore is should we have a team that actually is a team but is a providing care not just supervisory but actuallly a team that ist well defined with defined job descriptions and defined skill mixes clearly and that the team should then provide care at at each facility that is is because I think what I hear and what I heared with all th other facilities is that the nurse is everything you know the nurses it's a the nures than the admibnistrator or maybe the midwifery the the the labotomist the the but so that is why we trying to bring the concept of a team shoul we have I know they are essential health services package actually in some way already has defined who should be there so that whats really we want to explore but if there is something you think will be emphasised gore care cannot be given by a single person doing…

All: mmm…

P2: its true

INT2: so um… so yes we have already said that so who should be the members and what should be the different roles of members

P2: the administrator should do adminstration in that team, the nurse doing nursing and been hands on

INT2: Wel well I guess one its and I feel that others are… so should the team be the same everywhere for instance um… if you are in wher is the health post closest here

All: interrupting

P2: they they should not be like this primary health care to be tailored according to where the commnunity… the local place what would work for a particular community might not

necessarily work for another community somewhere so…

INT2: who should define those teams who should decide ok, wena this places the composition should be like this but in other places this is wghat the composition should be like

P2: those whom are there just…

P1: yeah from the districts until when you reach a certain level

P2: even even at the ministry level they should work with that particular with those particular people who are in that area so that those who are in that area are not alleniated from from…

INT2: Ok! One of the things to be we you want to ok, we were talking to the community was the big emphasis that the community should be represented in this in this team.

All: yes!!!

INT2: Um… go ahead.

INT: mmm… who should lead the team at the primary the team…

P1: Anyone with the skills of leadership can lead even a community member can lead even a doctor even a nurse who has a… or they they can choose elect

INT: How should the quality of the team be evaluated and the impa… the impacts its work be evaluated

P2: you should look at their initial objectives and see whether they have achioeved them for you to say they have been doing what they said they are going to do

INT2: What about the impacts kana often we what we say we are going to do is usually processes we that you do the other but whether its… how are we going to actually ensure that its affecting the healthcare the health of the people you are serving what sought of things can help us maybe to see it?

P7: the contribution from the the the communitty they may evaluate giving us um… like they they the suggestions and the telling us whatever if they are satisfied with the services the team is providing you hear that some of them are…. I think that it can help the community as well can help…

INT2: So should only at causes I'm just thinking it just that I don't have solutions I'm just I'm trying to get ideas akere this is what the whole point of this should we only look at the processes for instance should we only look at a… um… that ok! So this month we said we are going to to to weigh this many percentages of our babies should weigh or should we

also look at um… some little bit about the outcomes um I was just thinking lastnight what kind of outcomes should we look at eventually in the long run our morternal our mortility our our are mortality rate should we even look at um… some biological markers like how our diabetics are controlled and on how our hepatitis are controlled I'm just wondering whwt wht will make sense to really say yes, this team is working becaues some researches are work you can have lots and lots of processes then get 100 percent but sometimes they how they um… they translating from process to impacts and I think that's always the difference that we are also thinking how that could be really becauses sometimes you know its given for example that we we used to laugh about that when we have UB has the PMS system but it has been suspended recently. But we find that when were struggling about recruitment and we didn't have enough people and yet there were piles and piles of people who are but now who were reporting for duty then we have our HR department getting nine percent in average each because they have been doing lots of things and things so you know we can't like we are so we are have ben thinking for this one that kind of process because at the end we still didn't have people so we don't want possibly we don't get ninty nine percent still our mothers are dying and our children are still dy.. diabetics are not controlled and hepatitis is still not so we are just really this is just at to for this group to help us think through what they think it will be really the most meaningful way that won't is too much administration also.

P1: the the community should also stop complaining

All: (laughs)

P1: they should be verbalised in to say now we are improving the service ee… even the number of our our systems maybe if we have the morta… a higher mortality we should see the eee… mortality going down ee…

INT2: Especially the team has a link with the community you know they had health education assistants sometimes they are suppoesd they supposed… to work in there in the community…ok! Thank you any other?

P2: even programmes we should see programmes working like EPI we should have a number of ,it just that we should actually see most of our children being you know immunized,you see that programme should be working well and PMTCT programme should be working well we should know that this is the programme that it is my first time to and I have to make sure she I do the work quickly so that she she is enrolled in the ARV programme as fast as possible because after all the government the district or the government will the programmes should be running.

P1: even we should…

P2: calling for our nurses and doctors

P1: even the community should be taking the programmes seeing the programmes as eee… as as eee…what can I say is they should be happy for that programme they are so many programmes that that are being written I being put for the community to utilise and they don't utilise them but when we say that I don't maybe maybe like right now we they having pregnant mothers pregnant mothers they are those who don't who doesn't come for for ANC registration you will you will you will just see them coming for deliveries but we want to see hundreds percent of mothers registering for for the ANC so now we say no this people are in a position to to to take the programme to use it they see the importance of of that programme even bone the providers they should be happy ee… they should be happy because if they are getting eee… eee… good incentives it shows that atleast there are going tob e effective

INT: i Alright I will move to next question. What is your opinion about building primary care team as one of the interventions to improve primary care in Botswana.

INT2: Sorry I just

INT: Your opinions…

INT2: Which one…

INT: this one its says (gate) what is your opinion about building primary care team as one of the interventions to improve the healthcare in Botswana.

All: noise

P2: for me I think it will be a good thing so that it drives primary healthcare so it will be best if we could have that team

INT2: Now now the next question its um… I don't know how many of you have seen this big document from called the integrated health service plan of 2010-2020 it's a document on the ministry of health but in there its actually a its kind of a strategic thing for the ministry of health that's its outlining how healthcare would be given and how it is supported it's a beautiful document it really has a lot of the things we saying here in that document and and how and so they have developed a number of the values one of the values that it is listed there is ethics so um… is just we wanted to explore this a little bit more because we know that sometimes ok sometimes people are unethical becaues they are unethical but sometimes people are unethical or find themselves facing very difficult ethical dilemmas because of the shortages or the situations that they work under so this question really it needs exploring many of you remember is a problematic situation in your work but you find particularly morally difficult and so if if there is maybe we just want people to share I mean at the end when we have shared it also bring some of the things that especially coming from the community about this issues but I'm just going to be in your

own own experience becauses it's an ethical issue its um… we know we we identify it as so that I will know gore this one is talking about so and so (laughs)

P2: you mean you mean as if you find a situation you are you are confronted by an ethical dilemma

INT2: ethical dilemma as a health professional becaues of the situations under which you went I mean we have talked about all the difficulties of of people not being adequately provided with resources ot there are not enough people and things so basiaclly its looking to have any of you then had to face situations where you really had a serious ethical dilemma but you because of the const… the constraints under which you went through

P2: I don't if this one would answer that one but we once in Nyangabwe we were in the ward there was this patient very sick we could see is passing away so we had to counsel the relatives for you know for the passing away in the files there was a different name like where the patient was admitted since we get the the relatives the the next of kin information us we wanted to counsel that one who was in the file as a relative but then somebody came to say no you shouldn't have counselled this person its me who was supposed to have being its me who was supposed to be counselled about the condition of the patient. Actually it was a wife against the girlfriend in the file we we saw that we and to counsel the wife but now is the girlfriend coming to say this is the wrong person you could have counselled me not that one so we were like we we had to be very vigilant as to who to give the death notification form after the death because we could tell eee… may be a law suiter is coming… why did you give that one the the death certificate and so it was such eee… difficult but anyway we enede up handling it because we have just said no we are going to by the file this is becaues the file is telling us if there is the law suit we will be covering ourselves by saying we gave this one because she was written as as eee… as a um…

INT2: Ee mma.

P8: Us as the lay counsellors we come up with I was met a situation where by this lady was she was not a motswana she was from Uganda she came with the husband she couldn't speak English mmm… the husband could speak English, Setswana so during our session most of the time the husband will translate to to to to the woman so when the results came out there were indeterminate the husband was positive the woman was negative so they was some problems. The man didn't didn't ask me to to interpret the results after to th lady gore kana he is positive and the lady is negative then that lady could get a little bit what I was saying. When they went back home the man said no since by the time we went to the clinic it was just too hot maybe my high blood was high that's why I tested positive so that lady stayed for two to three days after three days she came to the clinic alone so she was… losing signs gore so that I can remember gore kana she is the lady who came with the husband and they were then I picked up the story she was aah! Kana now saying something

defferent from what you were saying. So after a week the guy came in and he said no why why can't you call us kana you know we are we are from Uganda,and that's lady we are related so she is my cousin I don't want to hurt she is so frustrated so I don't know what to do because now she is seeing that maybe I'm going to die from HIV why can't we change the story gore you know is not something related to HIV so now I went to my bosses bo U stuff like I told them I have a situation like this so they have said why can't we find eee… maybe somebody who maybe is speaking French so that the lady can get the message clearly gore what we we we were saying. So we were still working on on it.

INT2: I think thank you very much for sharing and I think what we should also be given more help will be either the those that are related to the constraints that was a big constraints. They are contraints becaues we are taliking about human resources because we are we are stack out and we end up doing things that we would normally not do it or you endup facing dilemmas that

P2: There was a child delivered in Nkoyaphiri one day and the doctor had to go with the ambulance at the back of the ambulance and undulating the baby all the way to Marina

INT2: Did the baby arive alive.

P7: the baby I don't know what happened afterwards so the clinic have to be left with no doctor and we were forced to go with the doctor helping the doctor all the way to Marina

Int2: even the administrators the administrators are also faced with ethical dilemma who do we transfer and who do we leave and you know just say things that we deal with everyday because we work with lots of constraints everday because we have main constraints ok um… when we we asked ok the co… users of the health service let me th… let me say you know in the different places different things came up it sounds it sounds here that because because the situation is slightly different from in from Maun or Mahalapye I think they are really constraints because they were coming from you know bo bo Moshopha , bo Mosolotshane so because one of the people participated in the in the discussion in Maun she is in Makakung I guess you know where Makakung is there is no no means of transport she might be in her late 20's she she is pregnant she was in horse back from Makakung to Kareng so that she can get the ambulance from Kareng to Maun and so when when they talk about this and she is alone in the health post in Makakung so one of the things they talked about is tha dilemma of being alone and there is no night watchman and the patients comes and knocks at your door at night so eee… one of the ethical dilemmas was they have to choose whether they would answer or they don't answer that call when they don't answer you know they said ke kgomo ya Moshate mmm… when they don't they don't sleep because maybe the cops may opt to come to the clinic when they is there is no electricity also in the clinics even no electricity in the village and and when they do open they they open at their own risk and so so this are some of the the the re we have done that

some of those people and the question the same question that we asked the community but in the community we said the same thing as because there is shortage sometimes you work under difficult situations and have you experienced as a user of the health service or your relatives any time you gone or treated in a way that would that was difficult that was either and when we asked that question in Mahalapye it was very you know something happened I did'nt expect there was an old lady about 80 she cried she just broke down cry and walked out and and another lady cried I mean you know I thought it was a mother and a daughter so I saw them outside I realized there were two completely unrelated people with unrelated issues that happened. Yesterday also I think in here one of the ladies I think cried when we asked this question so so they cros… because the the two in Mahalapye were so upset they wouldn't talk after that but the one in Gaborone she did speak after a while she she I think here she I don't know where she came from a small village around Gaborone she was pregnant and when she got to Marina the she they looked at the cards and so this is not the first baby so you are a woman (o mosadi) and they said climb in to the bed and so she was in pain and she eventually palama bola but nobody checked her for thirty minutes by the time they came the heart sound was gone and eventually she lost the baby so she cried here and so so I mean so so this is a kind of I'm just sharing this because I think eee… that's why I think its important the community should be involved to gain the trust of of what we are doing and I think because in some places the trust is really really gone or or…..

P2: because of the attitude of healthcare workers

INT2: I think what they say more you know what the healthcare workers many of them the users they actually think there is a shortage there is a big shortage but they say it is compounded by bad attitudes

P2: attitude aggravates everything

INT2: ee…they say yeah shortage they are ware those stories of Makakung there is only one they they said there are scared of the safety of these people they they also want the night watchman to be there… so they they are aware they they said everything the healthcare workers have said literary if you listen to to the recordings almost everything the healthcare workers have said about the shortage and the living condition or the conditions of service but they say they still think that the attitude its very bad

All: ee! That one we we can't deny it

P2: we can't deny it the attitude of the healthcare worker its its needs to be dealt with again

INT2: But anyway thank you very much this is the end of our thing it actually the longest one we have so far (laughs)…. There were a lot to say but its fine

P2: but you know what I think the government must also do you will find that somebody there at (gone ko) Makakung there (ko) has been…. The salaries<br><br>Int2: that's what they said…
