# Supplementary material for: Stakeholders’ Perceptions on Shortage of Healthcare Workers in Primary Healthcare in Botswana: Focus Group Discussions
Source: PLoS One. 2015 Aug 18;10(8):e0135846. doi: 10.1371/journal.pone.0135846 (PMC4540466; doi:10.1371/journal.pone.0135846)
Supplement: S8 Text — (PDF) [file pone.0135846.s008.pdf]

HURAPRIM PROJECT

Participant ID: Focus group one(2)
Date: 11th April 2012
Interviewer: Dr N
Interview Duration: 1hr.47min.20sec
Audio File Name: HealthCare Workers Group2

INTRODUCTION

INT: What is your understanding of Primary Healthcare? What do you understand about Primary Healthcare?

P1: My understanding of primary health care is providing preventative care to the community, in other words we educate the community how to avoid attain certain illnesses or diseases. We also provide umm… what is it… we also provide sexual reproductive health.

INT: hmm…

P1: that's it.

INT: ok

P1: it's… to me basically primary health care is dealing mostly with the community, how to help them with their problems and prevent them from getting illnesses. It more the preventive side than the curative side, that's my understanding.

INT: ok. Anyone else with the… with different understanding or addition to that.

P2: the same.

INT: huh?
P2: the same.

P3: umm… essential care given to individuals, families and communities. Like he was saying they should be reached at their places and there should be health education and the community should also participate.

INT: ok

P2: maybe to add on that, the care for the (*the participants voice was very low*) …should be

available, accessible to the community.

INT2: Maybe you should place the recorder closer somewhere there!

INT: Bring that one closer. So could we please speak louder.. Studies in Botswana have showed that there is a shortage of healthcare workers in primary care, especially in rural areas. Do you think there are enough or not enough health care workers in Botswana? If there are not enough why do you think it is so?

P3: I beg your pardon.

INT: do you think there enough or not enough healthcare workers in Botswana? I mean healthcare workers… yah healthcare workers in Botswana. If they are not enough why do you think it is so?

P1: well I think that they are there, maybe not enough… enough but enough to get the job done they are there. Maybe the reason why we don't have access to them in our facilities is because of maybe the conditions of the work that could be frustrating.

INT: ok… ok

P1: ke raya gore that's… kghmmm (*clearing the throat*) that's what I think. I think they are there, I think we have enough people actually, it's either… and then another things is maybe they are not being distributed properly, looking at where we a… those people to be, in which areas and then we don't distribute them properly without think of that. Whoever does that, that's another reason that I have.

P3: we don't have enough and more should be trained.

INT: ok

P2: I don't think we have enough taking an example of cadres like doctors, pharmacy technicians because one example one would give kghmm..(*clearing the throat…*), if you go to the clinics you will find that err a nurse is charged with the responsibility maybe dispensing, taking blood because all these cadres are not in.

INT: ok… ok what about aaa… deployment or distribution of the healthcare workers. Are they distributed in adequate numbers or not?

P3: only the nurses are distributed in adequate numbers.

INT: sorry?

P3: only the nursing cadre.

INT: hmm…

P3: nurses only.

INT: ok.

P3: are distributed in large numbers; like P2 was saying there is shortage of pharmacy technicians and doctors.

INT: ok

P1: you mean deployed around the country?

INT: yes.

P1: yah. I agree or disagree, they are deployed but like I was saying sometimes the… the… there is kind of like more deployments somewhere than in other areas. Yes. Like you find that maybe certain facilities, they need to deploy more health workers and nurses there, rather than other facilitities you find that there is a lot of healthcare workers but there is really nothing for them to do there and it’s not necessary for them to be there, there is nothing for them to do there, they just need a certain amount of them to be there. So you understand what I mean?

INT: hmm. (*Agreeing…*)

P1: yah, What im trying to say is , I think that’s… that’s another key area where I see a problem, I foresee a problem… problem there ,do you get what im trying to say... The way the deployment is done, sometimes I think it’s…

INT2: I think you should forget about that recorder , X so that we can capture some of the things.

INT: ok. Umm… did you get your thoughts through?

P1: yah…(agreeing)

INT ok, so what about retention?

P1: retention?

INT: hmm… (*Agreeing…*)

P1: you mean…

INT2: retaining staff.

P1: retaining staff it's… some would say it's tricky on how do you retain people but I think staff retention kana it involves… it involves a number of issues because most of the time you find that a lot of us will be concentrating a lot on the money part of it but that's not reason… I mean that's not the reason you can get to retain people. You also have to look at working conditions, where those people are working. Is it conducive for them to be working there, for the amount of hours they are there, for the amount of time they are there because you have to consider, how many… or in a year or in a month, you check what would quantify your study or how long or how much they spend there. If you find that people spend a lot of time at work, I mean you know some people spend most of their time at work than at home. The working place has to be conducive… conducive for them to be there all the time because if it's not you wouldn't be looking forward to be there, you want to be somewhere else. So the working conditions also have to be looked at gore is it conducive for people to be there. Do we, how do we reward people…

Phone vibrates on the table…

P1: you know; how do we reward people for putting in long hours and working so hard in a place because it's not only about the money. It's also about as a supervisor how do you conduct yourself towards your employees when they've done a good job, do you congratulate them, do you always complain, do you always look at the bad things they do or do you sometimes look at the good things that they do; not just the supervisors including the institutions we work for, what do they do to recognize that people are actually working hard where they are instead of aaaalways attacking them when they do something wrong because people become disgruntle when you know you come up with good initiatives in you facilities, you do very well, you do very good thing and you are also proud of what you doing in your facility and sometimes you do need a pet on the back, well not sometimes all the times. You need somebody to be telling you, you doing a great job even if it's somebody all the way from the top, it means a lot but if you do not get that and all you get is when you make a mistake and it's like somebody is coming firing down you, it become a problem. So in order for you to retain staff you have to look at all those kghmm (*Clearing the throat*) aspects of how do we actually congratulate people, how do we actually umm… what… how can I use…

P3: motivate staff.

P1: yah how do you motivate your staff. Motivation has to be… it's very important to motivate your staff because other people do like what they do…

INT: yah

P1: regardless of how bad the conditions are but you have to motivate your staff in different ways. You have to look into that gore how can we motivate our staff because if we talk about money…

ALL: he he he (*Laughing…*)

P1: you have to live with the real world and be realistic, you have to think about other ways to retain your staff because those are some of the things that people look at gore how you are treated at work for all the hard work you out in.

INT: ok.

P3: a! You have said everything. He he he (*Laughing…*) everything.

INT: hmm…

P3: You said everything at length.

INT: ok

P2: I think in addition to what he has sad; I can add to the part ya the place of work. Well at times you go to work and when a soldier goes to… to war he has to have a gun, and at times you find that you don't have instruments or equipment to use and you, you all the time are improvising which its quite discouraging and you don't do a good job without… (*voice very low*)

INT: question number 3, Do you think there are gaps issues or problems related to health care workers for primary health or not? If there are problems what do you think are the most important gaps, issues and problems?

P3: gaps?

INT: hmm… gaps or problems.

P1: problems?

P2: this question… to me it's related to the previous one.

P1: it goes back to what P2 was saying about so much being expected of us as health workers to do yet what we need, the instruments that we need, the equipments you know, the papers or… most of that stuff you find isn't there is not easy to come by. You have to… you mind me using that language; you have to hassle…

ALL: he he he… (*Laughing…*)

P1: you really need to hassle to get things done.

INT: ok.

P1: on your own and you are not getting paid from anyone, so those are one of the biggest gaps that we have; a lack of most of these things that we need.

INT: ok, are the healthcare workers adequately trained for their job?

P2: yes and no. I have already given an example of a nurse dispensing, a nurse ….. a pharmacy technician would be better placed. So at times you do have to improvise.

INT: ok… ok.

P1: that's true. Health is something that; it's ever changing so people have… people need to be trained at all the times because things change.

INT: is there more shortage in certain cadres?

P1: yes there is.

P3: he he… (*Laughing…*)

INT: ok examples…

P1: pharmacy… pharmacy technician, there is a…

P3: technicians, doctors…

P1: there is a big shortage, huge one… doctors, there is a shortage there, and err yah

INT: he he (*Laughing…*)

P1: there is a big shortage there.
INT: ok

P1: stuff like that.

INT: ok. Is there adequate support to do the job? Umm looking at coordination and management, I know you have already spoken about resources like equipment and all that. Well what about coordination and management, is there adequate support for the healthcare workers to do their job?

P3: from the managers?

INT: err management and coordination.

P1: well from my experience, from my experience some of our managers, you know like immediate supervisors there is a lot of support from them to get the job done. I mean like the immediate supervisors or maybe let me say matron to make things easy for you, from our mangers there is support to get things done, there is a lot of support for us to get things done, you know to coordinate that things happen and to motivate where it's needed for things to be done from our managing… but I feel that maybe they themselves are not receiving enough support from maybe their managers up there in order for them to actually give us that much encouragement we need from them because I think 'cause even us we also expect a lot from them, we expect a lot from them even though we know that there is a chain of command but it is there from our managers, immediate managers from here.

INT: ok, you want to add something? (*Referring to P3…*)

P3: No..he said what I wanted to say

INT: he he he (*Laughing….*)

INT2: He said what you wanted to say.

P3: Matrons and the community, health nurses, they always support us.

INT: ok

P3: even workshops, they provide us with information and they… they do everything seriously

INT: is there… next question, that is question number 4. Is there a problem of health care workers in the rural areas? If yes why do you it is so?

P1: not sure.

P2: The thing is some of us have not worked in rural areas he he he (*Laughing…*) unless we use …

P3: there is a lot of problems there.

P1: maybe they might have problems. Their problems maybe would be being left out or not being taken too seriously because they are in a rural area, what do you need, you have this you have that, what more do you want you know, you don't see the use of you having that or needing that. That can also cause some to be frustrated because I mean,well, you are also serving people, people who also need those services from whatever you need to provide them with and maybe they might not… or maybe they can't voice out their… their problems out that well because maybe they feel maybe there won't be any point. This is just…

INT2: ok I… I… I… you know the question also looks at so you think there is a problem of shortage of heath workers in rural areas.

P1: oh.

INT2: yes…

P3: sometimes.

INT: sometimes?

P3: especially midwives.

INT: ok.

P1: yah, I think there is yah, there is a shortage.

P3: midwives and doctors, they don't have midwives and doctors

P2: umm in the rural areas you find that Nurses are everything, they are Doctors,pharmacists,and everything. And I think when it comes to workshops, they might not be able to attend because you are looking at your station and you can't leave your station alone.

INT: ok… ok err do living conditions in the rural areas contribute to this shortage of the healthcare workers?

P3: yes, because health workers or nurses they refuse to go there because, especially in rural areas like Xaxa, Oliphas Drift, they don't go there.

INT: hmm… why? Why is that so? Why do you think…
P3: it is because of the living conditions there is no electricity, he roads are bad aaa everything, the roads are bad.

INT2: he he he (*Laughing….*)
INT: ok does…

P1: people also look at comfort or comfort zone, and if you feel that you are not going to be comfortable living there especially if there is no water, there is no you know the basic stuff, water and electricity is hard to come by you wouldn't want to be there. And bad roads…

P3: bumpy roads.

P1: it becomes a problem because you know gore there is going to be… maybe if it rains heavily you will not be able to leave that place, there is no water, there is no power, nobody can

come and visit you, you can't even ask anybody to come there because the conditions are bad, you become too frustrated you don't want to stay there because of the bad conditions that you face when you are there.

INT: ok.

P1: electricity, water…

INT: what about remoteness; does it contribute to the shortage?

P1: yah!

INT: ok, can you elaborate.

P1: if it's very very remote because kana when you leave and you want to go to work somewhere, you ask yourself am I going to be able to relate with anybody who is there and then you will be told gore hey that area is so remote…

INT: he he he… (*Laughing…*)

P1: all you see is animals. Now the problem is you are too used to growing up in towns and big villages where there is always people around and stuff to do and if you go to a remote area there is nothing to do but you are too used to having something to do at least to keep you busy. But if a place is very very remote you going to become you know you going to have that environmental stress that you are there it's remote, there is nothing to do, absolutely nothing, no one to see, nothing, you are just something… it's like you have been dropped in Siberia somewhere…

INT: he he he… (*Laughing…*)

P1: you've been taken to jail.

INT: he he he… (*Laughing…*)

P1: to suffer, so remoteness does count

INT: ok

P1: does count really.

INT: what about the cost of living in rural areas, is it expensive to live there?

P1: In rural areas?

INT: hmm!

P3: he he he… (*Laughing…*)

P1: the cost of living in the rural area is not high. What causes the cost of living is rural area is what you do as an individual. For instance, if you are taken to a rural area you are not going to spend that much money doing anything but the only time that you spend a lot of money is because you want to move around a lot meaning you don't want to be there as often as you would want, you always just want to tra… just leave the place. You spend a lot of money but otherwise, coming back to your question, cost of living a rural area is not high.

INT: ok

P1: It's very low.

INT: hmm... What do the others say?

P2: I think when he says the cost of living is not very high, he is looking at err financial… looking at financial… looking at the other point of view emotional support, it can be expensive. You really need your… you really need people around. If I want to see my aunt today I can just you know board a bus and go. But if I'm… what's that… err… when I started in Maun when I was a very young girl most of the time I was crying and I would come… every… every month I would come and it's so expensive.

ALL: he he he… (*giggling…*)

P2: so emotionally it can be so expensive.

INT: ok. Now looking at jobs and schools for partners /children, I mean partners/children of the healthcare workers, umm do they have… it means the availability of jobs or schools for for bone the partners of the healthcare workers or schools for their children.

P3: there is nothing.
P1: there is nothing.

INT2: so could it be the reason why there is a shortage of health manpower in the rural areas that there are no schools or jobs for partners and children?

P1: yes, it also contributes because I mean if you know that you've got a partner and children and then you are told you need to be transferred somewhere out of town you are not going to easily accept it ,in reality you do look at those aspects that ok, where I'm going are there good schools for my children to attend, no. can my partner find a job there, probably no. even an average job that he can keep there, no. so then nobody would want to be there.

INT2: before you proceed; what about opportunities for continuing professional development like further education and stuff like that in rural areas, what about opportunities for continuing professional development for health care workers in rural areas.

P1: I think in rural areas, well my own personal… in rural areas it might be a little more difficult because communication-wise, I mean we talking about news papers and stuff, they might not as available as they are… as they would be in more urban developed areas for you to be able to see or read… you know there is these days a lot of courses being offered even in universities, you can come and do this but if you are not aware of things like that happening as like you would be in a town or developed village it tends to become a problem you cannot develop yourself further because you wouldn't know what's happening. Nothing is really available to you like it would be in a town or village, that's… to know,yes one can go to school to do this nowadays, they are offering such and such a course or tomorrow they decide to open up a new school they are offering a course to do, you can do this and that, you can go there. You in the village you wouldn't know, you probably hear somebody say it there is school now I'm doing such and such a course, a! When did you start? I've long started, a, you didn't know, they have been offering that course maybe 6 months or so ago and you didn't know. So that also contributes to the frustration.

P3: even the coverage is poor, there is no coverage for these cells, even the radio, absolutely nothing.

INT: oh the networks.

P3: yes..

INT: ok. Kghmmm… (*Clearing the throat*) do you want to say something (*Referring to P2*)

P2: no INT!

INT: ok, proceeding on t question number 5; What do you think should be done about the lack of health workers for primary healthcare in Botswana? Now that's, the solutions. What do you think should be done?

P1: are we… are we also looking at rural areas.

INT: hmm (*Agreeing…*) it said in Botswana.

P2: a while ago somebody said umm money cannot be a solution to… to the problem. But I think in this case it can be if a certain amount of money is given to these people who go to the rural areas, attractive…

INT: yah…

P2: well a number of people will go to the rural areas.

P3: it's there, it is called RASA.

P2: attractive! If it's attractive.

ALL: ha ha ha ha… (*Laughing…*)

P1: I will try to add to that too, yah, money;

INT: ok what else?

P1: I think also living conditions where you are going to be living, the place you going to be living in. they should provide proper living quarters, attractive living quarters I'm not talking about a mansion or a huge-huge house but something that is attractive to someone. Where someone can say I maybe living in a rural, I maybe working in a rural area but I'm living in a nice house, it okay, it's secure, you've got everything you need, you know it's an attractive place… well I live well, where I stay is alright, not an old, these old rusty houses that are there.

ALL: ha ha ha… (*Laughing…*)

P1: you know, it has to be something attractive too.

INT: what about working places, are they in a good state right now, I mean from what you know in the rural areas, the thing is I hear him talking about houses there.

P3: they are very small because they are health posts.

INT: hmm… ok

P1: well yah, the… the clinics or the places where you work, that one they have to look at another thing, how many people actually come there to use that particular place.

INT: ok.

P1: yah, you know you look at the demographics, where it is, would it be necessary to actually add all sorts of fancy stuff inside so to say, so to speak. It to… ok the place has to be okay, it has to be nice too you know, it has to be done nicely but they shouldn't over do it, too much to add unnecessary… that that you won't be using but they should also do something about that too because the clinics, clinics that are being used now they are old, is the… those things were built ages ago probably before I was even born, so they have to refurbish to make them a little more attractive, to be… yah I agree with what P3 is saying. I concur.

INT: now looking at the training; do you think more…more people should be trained; more

healthcare workers should be trained?

P1: yes! More health workers… they should be trained, we need them to be… and I think the training should also be improved ‘cause times are changing. So we need to make improvements here and there, not everywhere just here and there to stay with the time.

INT: yes, I would like to hear other opinions, what do you say?

P3: yes more staff should be trained.

P1: and also on the training they should also consider people’s preferences on the types of training they need, so that when you look at productivity it actually also increases if somebody is being trained on what they prefer to be trained on because they have a particular interest in that field. Productivity levels will also increase instead of just being trained because you need to be trained maybe something you don’t want to be trained in, I mean when we train, like we said more people should be trained we should also look at that aspect.

INT: ok, what about deployment/distribution? do you think… what do you think, do you think more healthcare workers should be distributed or deployed.

P1: well like I was saying, distribution and deployment you have to look at where you are taking them and the necessity of you taking a certain number of health workers and you find that some health workers actually prefer to be working in distant areas or villages or some rural areas. There are some who do. So when you do that deployment which is necessary we also consider that. Maybe what we can do is try and ask people their preferences where would they want to be deployed to besides just being, you know besides being in a town… or in a… in a town or city like this. Where would you prefer to be deployed to.

INT: ok

P1: you find that some people will say me, I prefere to work in rural areas because in rural areas I can get to do one, two, three, four, some will say I prefer to stay in urban areas, some will say a,in urban areas you use too much money here this and that and that. So I think we can also look at that aspect, it can… I think it might help with deployment of nurses oh health worker so to say to rural areas too because I mean there is a problem deploying health workers to rural areas, most of them don’t want to go there then you end up having a shortage of them being there. So maybe if we could find out what they think about it of being taken there, if they don’t mind and maybe that sort of criteria could be used. It might help.

INT: ok the other let’s talk. One person should not be contributing alone.

P3: Well I am tired of MCH at work. I spent all my time on it.

INT2: You are tired of MCH?

P3: they picked me for it at work, they just pickeed me for it without even having to knockoff.

INT2: ok, I think you can ask other questions.

INT: ok, question P6, In your experience,

INT2: I wanted to know the retention strategies would you suggest so that at least when we talk about the problems, what kind of suggestion should we have in place, rention strategies?

P2: hm-hm please be quite (*talking to P1*)
You want to talk, he will add more.

P2: Yes, it was already…well he had mentioned for example motivation program, supervisers, availability of equipment and supplies and things that are…are…and in addition to that, actually it's what he said, like accommodation, I think if we all had accommodation, we cannot be all, but a very good percentage of us having accommodation but as of now you find that maybe..maybe the 5% of health workers are the only ones who have accommodation. That's it X… ha haha (*Laughing…*)

P1: you could also… that help us do that, I think we also need to benchmark, benchmark with other institutions gore how do you actually help to retain your staff where you are. And then it also help to built on the strategies that we might have in you know in place right now, thinking of doing. It can help us maybe look at it in a different angle maybe we could try this or try that because like I said, retention of staff involves a number of things. So if we could benchmark and then add on the strategies that P2 was talking about, a lot of motivation. The other motivation that we have to look at how are you going to motivate your staff, what am I looking at, what do I want to do, do I just want to call them in the room and in the report and then congratulate them by mouth, word of mouth or is there something we want to put in place, maybe we could start employee of the month, we put a picture of somebody there- employee of the month, he gets a certificate for that month, you have been working so hard this month, we've noticed that including your colleagues. Then you give them a certificate that's been signed and maybe something… a little trophy… something to cherish him.

INT: ok.

P1: as an example. We should look at things like that.

INT2: so…

P2: in addition to that…

INT2: ee P2!

P2: well we need to motivate the people, we should have an idea of what it is that motivates the people. Then we can send people around in order to hear from them (*voice very low*)

INT: ok
INT2: that is true. Aa… what should be done to get the right skill mix?
P1: the right?

INT2: skill mix. Well you now facilities work…. There are different groups of people, so what can be done to get the right skill mix if we are to alleviate the shortage.

P2: Well at times you… you will not have enough people in the facility because you are thinking of vacancies, yes.. maybe if vacancies are created le gale you would be told that there is only one vacancy found.

P1: and also maybe to add on to what P2 is saying. I think you have to look at the facility and the type of… they type of conditions you may encounter and then maybe that will also help, I mean kana we talk about the right skill mix, you can't just put everyone there… somebody who in fact… somebody necessarily isn't supposed to be there because they will be sitting there and doing nothing they won't be using their skill to its outmost best. I think you can look at what type of conditions you come across weekly or monthly basis and then you look in to that see gore okay we seem to be having more of this than that so maybe because we have a lot more of this we might need a lot more people skilled in this, problem 2 that we don't get that that much then other areas you look at that too.

INT: ok.

INT2: so what do you think about task shifting? Is it another way of dealing with shortage or; what are your thoughts?

P2: what was the question again?

INT2: I said what about task shifting; you know could it be another strategy to resolve shortage whereby for example the work that usually… the work that is usually done or carried out by registered nurses to another group or by doctors shifted to different cadres.

P2: it's not a solution; it's a problem.

INT2: hmm… how is it a problem?

P2: it's a problem because you wouldn't get the quality job, I mean if the job was to be done by a PR and you give it to a SRN ,its just a problem.

INT2: he he he! (*Laughing…*)

P2: so I think it's not a solution, it's a problem on its own.

INT2: ok. You think…

P2: but in the absence of any other solution…

P1: because I think you looking at that, you also looking at stream lining of duties, talk about task shifting, that can I move… it's a bit of a problem.

INT2: it's a problem.
INT: ok. In your experience; what solutions/interventions have already been tried to resolve these problems? The problems that you have mentioned already.

P3: we have been allocating nurses to do doctors' work and we don't… I don't think we produce quality, I mean! I mean just in case.

INTS: he he he (*giggling*…), ok.

P1: another thing that we have done, I think… I think to try and solve a problem we have to… to try and solve a problem, one thing we've tried to do at the facility, we… for us to solve a problem we have to accept that it's there as a facility, it shouldn't be an individual's problem, we solve… we accept we… we… have a problem here. Then we have to agree that this is a problem and we sit around and then you ask yourself ok what is it we want to achieve as a facility not just as an individual but as a facility because we've got what we all want to achieve as individuals but as a facility what do we want to achieve with our facility as a whole. We sit down we want or our facility to be recognizable, to be providing quality care and all the like, you know we come up with all these nice things. Right, if that's what… how are going to do… how are going to do it, that's when we start coming up with ideas of how we can solve our problems with what we have how are we going to do this. We can you know form groups in our facility gore this particular group takes care of this, this particular group takes care of that you know, you get what I mean. And then from there on we also come up with our own individual objectives as those little groups gore as a group since maybe we are dealing with… let's say management of kids' illnesses, this is what we want to achieve. Looking at what we get every day you know. Those who are dealing with maternal child; this is what we want to achieve. And then at the end of the day we absorb… those problems become like they are obstacles, things that we face every day, and then we see how we can get through to these obstacles as a facility. And then at the end of the day all the solutions we come up with you manage to solve your problems. There will be issues here and there along the way but it's how you actually want to deal with them as a facility at first.

INT: ok has Performance based reward system worked?

P2: I wanted to add on…

INT: oh ok…

P2: he's talked of planning and implementing; and I think it would be wise to …… to inform those committees to carry out certain activities then regularly you sit and evaluate if you have achieved and if you haven't see what it is that failed you and then re-plan.

INT: yes I was saying, performance based reward system, umm has it worked? Well the question was in your own experience what solutions have already been tried to solve the problems.

P1: it's tricky, BPR is… it's… in a health facility…I think it's… it's a bit difficult, especially in out-patient department, it's… it's a bit difficult to… to measure sometimes certain performances because out-patient you deal with people today, same day they are gone. Yah but I think what you can measure is what you implement in your facility because I mean because some of our managers they encourage us to come up with individual implementations; as an individual you have to come up with an implementation and then you implement what you have to do and then you have to come with evidence to quantify what you have been doing. When you do your performance base… you know you implement something, you come up with that… your own objective and then from then on you do it, you actually… after doing you have to show the evidence, this is what I have been doing but I managed to go this far pertaining to your objectives and milestones… this is what I've been doing, this is what I have managed to do. I think PBR is just to get high marks which I don't would help to get the quality… would it help for you to get the quality care that needs to be given, 'cause PBR to is 50/50, I don't really believe so much in it in a health facility.

INT: Moving primary care from Ministry of Local government to Ministry of Health, as it worked in your own experience.

P2: problem…

P3: such as what P2 ha ha ha (*Laughing…*)

P2: maintenance of the equipment or the one regarding buildings, if you call people from the council they will tell you to go to ministry of health, ministry of health will tell you "No, we have not been handed over". You end up running around; at time a door is broken then the carpenter will tell you "ok I will come and assess it", and then he says "go and buy the lock", and you are like "I'm a nurse" then he says "it's up to you, you will see how you go about it". So when you go to supplies at central government, so it's really causing a lot of problems. I had worked before for central government, I've just moved to the primary health, so they are telling me, we have shortage of transport and they would say you know before this merging we never had any problem, like you see people have not come for the meeting because of transport. They'd tell you if we were still with the council we wouldn't have a problem. So it really has its own goods… good and bad.

INT: ok what do the others say?

P2: and besides… even management-wise it's like two different err management… types of management are merging because you hear them saying that "a! this is how we have not being doing", so it has its own problems.

INT: yes, others; I would like to hear your comments on that.

P1: we'll be fired (*whispering…*)

INT: he he he (*Laughing…*) you have to fight?

P1: no I think… I'm going to agree with what P2 is saying, right now I think it's in a state of confusion, a serious state of confusion because the… the… on one hand, we used to have things functioning in a certain way, so now like P2 is saying, when something wrong… when something wrong happens, you know we used to go, we were using office to report such and such a problem has happened; "no, go and talk to the guys at the council", you go to the guys at the council that you went to report we are having a problem with… maybe something broken or this and that; they will tell you a, the report hasn't been handed over to them, they can't touch anything there then you have to go back to here and there… it's… it's a state of confusion.

INT: ok

P1: esp… you know… people… everyone is telling their own story, so nothing has actually being done, even when it comes to the equipment being provided and stuff; ok other basic stuff they do provide but when you talk about other major things like transport and electrical problems and this and that it's a problem. So you don't know exactly where to go to get anything resolved because you don't know who is going to tell you where to go or who will help.

INT: ok.

P1: so it becomes a problem for us to… wanting to deal with problems that are there because you'd say "what's the point", you are going to be chased around the whole building; go see semangmang, go see semangmang. So I think it's in a state of con… it's not working.

INT: ok, and then the setting up….

P3: even the supervision. For us to supervise the industrial class a ijaija… they will tell you that they are now cleaners they are no… they are not general duty assistants. There is no job description now.

INT2: so what has caused this difference, I mean if they are not general duty assistants, if they

are cleaners don't they still fall under that.

P3: ae! Nowadays you can't send then, even to collect specimens or what, to… to transport them to the l….. But we used to use them to transport specimens. I am stummering.

ALL: he he he (*Giggling…*)

P1: coordination and understanding of duties actually… I don't know… explaining that to people it's a problem, some say hey this is what you are supposed this is what you are supposed to be doing whereas before then they were told this is how you are supposed to be doing it, maybe the explanation… the explaining part of it is the problem, you know I don't think…what I am saying is that even when you hand things over you tell people that ok, fine this is what has been going on,you see! If then people are saying no,but it was not suppose to be like that,it was suppose to be like,but it was suppose to be like this,that there has to be a way… you tell people about change, you don't tell people that.. yah, stop doing that now. This is what… you not supposed to… maybe some words when you tell someone that you are no longer called something.something, you are called this you are this,they then take it in the literal sense, maybe not meaning it in that way you see.., because I do not see how there duties can change just drastically like that but … but what P3 is saying is true, because they refuse to work…

P3: they are just cleaners.

P1: yes they refuse… if you tell them that… if you want them to do something you really need to ask them, and you know ask them in a kind of begging way.

P3: even for cooking for patients in maternity they do not in fact,who cooks for them by the way?

INT: do you want to say something (*asking the other interviwer*) ok, aa… looking at setting up of district management teams, has it helped? Setting up of district management teams… has it…

P1: DHMTs?

INT2: yah, the DHMTs, has that helped in addressing the shortage problem.

P2: this would want somebody who has been there… (*voice very low*)

INT: how long?

P2: 6 months.

INT: 6 months.

P1: I think with DHMT… DHMT will know gore who is responsible for what where because I don't know… I dot fully quite understand the DHMT in the setup right now gore who is where, who is responsible for this and that and how… how… how does it match together. It's… it's a bit confusing.

INT2: is it a new thing; the DHMT?

P1: it seems like it's new, because it …

P3: Is it not the same DHT that was there?

INT2: District Health Teams…

P3:Yes, it is the one.

P1: Wait , DHT was just a council alone, the hospital had its own,now they are an entity.

INT2: ehee…

P1: You see, it's from the hospital side and the council side, a kere now they are all under one ministry, so the council guys cannot be DHT of council alone. Now they are merging with the hospital and then from there that's where we don't quite understand who is who, where, how it functions properly, for us to know that ok,when we look at the DHMT, is the hospital person confining themselves to the hospital only you know or does it include the hospitals and the clinics or have they just phoned the DHMT and said ok you guy… umm you guys there your team there that's been dealing with clinics carry on with dealing with the clinics, we will carry on dealing with the hospitals because sometimes what would happen in the clinics you find that a directive or a letter would have come from the manager from the hospital side to the clinic side and they don't possibly like agree with it but that's what just happened. Then we ask but what about semangmang, you know yo re itseng gore le ene ke motho yo o ko DHMT from the clinical side, how do you… how did you… what's going on, you know people will be like nna we are just told what to do and the you are like that's what we don't quite understand gore how do they work together, who is who, because I think if they… I don't know… if they look seriously into it, it will work, if they converge together and make improvements.

INT2: so do you think…

P2: yes INT2 I was just saying.. I've just remembered that…that to me there is that lot of confusion or maybe some gaps in the management arrangement because the DHMT relies on Princess Marina, the Gaborone, depends on the Princess Marina. There are no corporate ……… (*voice very low*) when you have a messenger, there is nothing like ………(*voice very low*) we rely on Marina, everything we rely on Marina. So I think there is… there is confusion.

INT: ok. Now umm introduction of Medical school programs and faculty in rural areas and

primary care, umm has that worked?

P2: introduction of?

INT: Introduction of Medical school programs and faculty in rural areas and primary care?
INT2: I don't know if you are aware, do you think the introduction of medical school programs and faculty in rural areas and you know and primary care, if it's… it would you know help with the shortage right now in Maun and Mahalapye. They are trying to get some faculty ba school of medicine, training some students there, medical doctors there, do you think now that we have a medical school and also that we have those satellites tsa medical school offering those programs, do you sss… think it will help cut the shortage?

P2: I would think so because from experience, where there is a school next to the hospital, when the students are in their practical, it somehow solves the problem.

INT: and others; what do you think?

P1: yah… I think it… it… it would help yah; it also gives people exposure to what's… you know exposure to different things, I mean it would help a lot, it's a good initiative because I mean something has to start from somewhere,

P3: definitely

P1: yah… and to me it sounds like a very good initiative. It would help because that would mean we would have student doctors or… who would be helping around the villages the same way it usually happens when you training in medicine, you go out into those villages and you continue your training. It would help…

INT: ok.

P1: …especially that it's here.

INT: now looking at the interventions/solutions that the government has tried… has implemented to try and help with the problems that existe… or still existing. Umm do you think those solutions worked.

INT2: maybe just to help just you out; we were talking about… we talked about the… the merging of the local government and ministry, I mean it's one of the things that the government… I don't know if that was the reason but they merged, and they introduced performance based systems so looking at some of those things do you think you know… or the task shifting that they introduced err you know, do you think those things have worked and if they haven't… if they worked why do you think they worked, if they haven't worked why do you think they haven't worked? Like we said there is right or wrong answer.

ALL: ha ha ha (*Laughing…*)

INT2: so you don't worry… I'm not sure, maybe this is wrong, just say what is in your… your… your… your opinion or what you have observed.

P2: the problem with that system, kana when it came, a kere there was registry like 1, 2, 3, but from experience I've heard people complaining that kana this is the reward system was to offer people but never once have we… have we had of anyone who had lunch with the president like it was one of them.

INT2: oh?
ALL: ha ha ha (*Laughing…*)

P2: so… that was one of them, ha ha ha (*Laughing…*). So I really don't see any changes it has brought about.

INT: ok.

P1: yah.

INT2: but what do you think really prevented that from happening? Just to use that example.

P2: I don't know because people have performed, I mean the… the… there are people who got their 90% and it didn't…

INT2: that didn't happen?

P2: hmm… (*Agreeing…*)

P1: no, you know what happened, you know like yah… like I was saying this PBRS, I don't believe in it really.

ALL: ha ha ha (*Laughing…*)

P1: It doesn't… nothing has happened what they said it would because when they are talking about performance based reward, even in the work place, you know promotions, stuff like that you know small little things and nothing… nothing… absolutely nothing has happened and it's becoming too monotonous. PBRS comes, next quarter PBRS comes again you see… it's so monotonous that it… it has no importance

INT: hi hi hi (*Giggling…*)

P1: it's… the only time really, to be… the only time I ever so it challenging it was when I was in a different facility with a certain manager of mine who will actually made you… you know

you didn't just, in the olden days what you just have to do is you just bring it and then they write whatever and you are there ,then someone records, you know coming up with something out of your head; ok .i know this one they are like this you know. And then you will also ask yourself if,,. but then what's the point of doing this, while it has been ordered to be done, I say there is no point you know, you can't call me and have me sit here you don't even… nothing! You are supposed to mark me down in my performance from what I do day and day out when I come in. you are not supposed to be biased you are supposed to be you know… and that also helps you to improve as an individual that! I need to pull my socks up, I need to do something. It's only the current manager that I was working with she made you do it she just gave you the form as it was a bo are, "go and come up with objectives. Tell me what do you want to do, what…", you know you go and do it, you sweat, at first yah you would be angry like exactly what is hapenning why are they making me do this, yah..someone want me to do for them PDP and stuff like that but it might seem like that but at the end of the day when you actually do it, you can see how this will benefit you in the long run, I mean even if you go and work in a different facility believe you me, you can see how it will benefit you because it helps you use your mind. It helps you initiate things, it helps you think, it helps you use yourself to the potential you didn't think you have,you see, but the way it's going now, I mean that a manage would make you do it that way, but when you perceive it ahead that for the government it's not working the working the way they thought it work, the way they wanted it to happen; it's not, it's not happening. Not at all. It's just a paper that's there that you were forced to have signed. But if it was done the way… you know we looked at it in that perspective that shows us… because no one is being rewarded.

INT2: so nobody is being rewarded for those 90's?

P1: no one. Nobody.

INT2: ok

P1: it a PBRS, you don't secretly reward people if you do that then what's the point,you see. People have know that you see; if you work hard you achieve your goals, this is where… this is where… this is where you'll end up and it will motivate you.

INT2: ok.

INT: ok number 8, What interventions would make the biggest difference to improve primary healthcare?

P1: big?

INT: hmm…

INT2: yah something, you've… you were helping us understand that PBRS has really not worked the way it was according to the stipulations. It was supposed to… you know… it was a good tool but in terms of implementation, you haven't seen that. So what interventions would

the biggest different to improve primary health care because these ones you are saying they really haven't been successful. So maybe we need new interventions.

P2: I think re-visiting the same PBRS and implementing that which was not implemented, like the same reward they were saying put in place given to the right people who deserve…

INT: ok, what else?

P1: I think also according to what P2 is saying, maybe certain, certain, certain changes can be made to the way I… the PBRS form is done. I mean especially the health facilities or that's a… it's, it's, it's a just that like a clinic or a hospital, I don't think you can measure what happens there the same way, each and every facility has a way things are done. I think what they need to is also change that format that is used to accommodate what we are doing there. So you can properly measure how people are doing their jobs and people should be rewarded it should be done in such a way that it's recognizable to everybody else gore this person have achieved 1, 2, 3 for looking at the way they bring performing because obviously there are people out there we know or we can see gore this person is really performing, they deserve to be rewarded in a certain way, you get me, and it should show. So if they can make changes to the way it's done it will help.

INT: ok.

P2: in addition to that, if this were to be implemented and maybe we have a.. whether a monthly or quarterly, some application sort of to see for this… maybe for the past so many years these are the people who have performed and this is what they have received so that it becomes public and I see my name… you see my name there.

INT: ha ha ha (*Laughing…*)
INT2: recognition!!!

P1: yah, some form of recognition.

INT: ok,

P1: and also being given like scholarships, something that could work,not everybody can be promoted, but if you are given rewards in such a way like scholarships, like "oh yah, I get a scholarship", you know you work hard you are given a scholarship to further yourself. It could also work to help solve the problem.

INT: ok.

P2: then again… because it's not everyone who can get a scholarship, maybe an outing to places like Kasane.

INT: ok, continuing on to the next question. Building of effective primary care team has been suggested as a potential intervention to improve primary care in Botswana. What is your understanding of effective primary care team?

P1: who it should include?

INT: hmm?
P1: like whom it should include.

INT: no when you a primary care team is effective what do you mean by that, what… or should I say characteristics… for you to be able to say this is an effective team.

P2: I think the team should have all the qualified stuff necessary to run a… what… a district and all the necessary resources; human, material and finance.

INT: ok. And err… you want to say something?

P1: yah they should also have their own mandate on how they do things, the shouldn't be in too much control of them, telling them what do, they should have their own separate mandate of how… how to run… how… how… that team actually runs itself, how it works, you know its expectations, objects and it shouldn't be something that be like, it should under a certain… ok it could be under an institution but they should be given the freedom to be able to run themselves. And they should also have a umm… how can I put it… they shouldn't be too aloof from those who are working on the ground, they should be hand in hand, communication or partnership between them and everybody down on the ground; they should concentrate so that we know what going on, they should have those characteristics. Because at times we hear that there is aa… health team or a team that's doing something and nobody actually knows who they are, what going on, what are they doing and you were never told anything. So I think there should also be that joined custody between them, the necessary people who they going to be dealing with.

INT: who do you think should make up this team?

P3: different health professionals.

INT: for example…

P3: nurses, doctors, pharmacy technicians, lab technicians, FWEs, and social workers.

INT: ok

P3: and there should be a coordinator, and feedbacks should be given.

INT: ok looking at the examples of the people that you have given, what about if one the

members of the community are amongst those who make the team? What do you think about that?

P3: it's okay because there would be community participation.

P2: what was the question again?

INT: the question was umm…well P3 mentioned examples of people who make up the team, so I was saying… because P3 said it should be healthcare workers, so I was saying what about if somebody from the community is also a member of that team?

P1: yah, it's good, they are one of the major stakeholders because we are dealing with them. Like I was saying that, we need that… that…. Because we need constant communication with them and also to the community so they know what's going on, since we deal with them mostly,again.. . It's no longer about us it concerns them in a way. So if we keep them out of it, it will make life difficult for us.

P2: I have a different view, I don't think it would be, when.. when we discuss we should all be at the same level and I don't what a member community you are thinking of. I would rather… we have an advisory committee, that would bring in the community with err people with different qualifications.

INT: ok, what should the roles of the different members be?

INT2: yah what should the roles of those people that you've mentioned be? You know like social worker, doctors and nurses; in the team, remember we are talking about a team

P1: well in that team, we are thinking of various issues and various cadres. So I think their roles should be in line with the cadres that they are dealing with; if it's nurses they should be dealing with what nurses are dealing with, if it's doctors what doctors are dealing with and so forth. And then from there when they come together,since each of us… each of them will be dealing with… there is a nurse, there is a doctor, a social worker, they dealing with those issues that they face then each of them should be… in fact, rightfully they should be doing that, nurses should be dealing with nurses' issues, doctors with doctors' issues and so forth. But when they come together I think they should find a way in which they can put those issues to an understanding for both of them to work together, but just in perspective they should each deal with issues from their different cadres because a kere you will know gore as a doctor this is what you deal with, as a nurse you can't say no, but I know it wouldn't work that way.

INT: and who should lead the team?

P1: Who?
INT: hmm… amongst all these people with the different cadres, who should lead the team?

P1: I think with whom should lead the team; would have be someone they should err… somebody they should choose among themselves, who has those strong characteristics of being a leader who can objectively deal with issues. Looking at the fact that there are different cadres there… so if you are too individualistic you won't want to deal with other people's issues, you want to say your issues are much be… are much bigger or more of a problem than everybody else's. So I think the best would be for them to choose someone within that group who has those strong characteristics to be a leader. You know the characteristic of a leader, you look at all sorts of types issues to be dealing with because I mean if… if… a kere we'd be trying… in that case we'd be avoiding creating another problem on top of another one, we are human beings, we behave differently, we think differently, if we say, let a doctor be a leader… why?... why?... why should a doctor be a leader? Every time there is talk that Doctors this,Doctors that, you see what I am saying!You knpw people will start bringing up these unnecessary issues tse e leng gore di teng. So the best way to it is to choose somebody at that point that group could see has those good characteristics to lead them.

INT: ok. Do you also agree or you have a different opinion?

P2: kana ke gore the tendency is we… we've been socialized to believe that a doctor can do everything and I think it's time we move from there. If maybe a nurse or social worker with some background of management, the person can actually lead the team.

INT: ok. And how should the quality of the team work be evaluated and the impact its work evaluated?

P1: since umm… that team like I said they will have come up with objectives and those objective they have to have you know milestones and then a time-frame. And then I think if they use that type of criteria, they can be able to have an evaluation at the end,to say okay this was the objective, that mandate I was talking about, they gave to us; they have to provide it gore this is what we want to, this is what we want to work to, this is what we want to achieve. Then we leave everything… then they do whatever they do, and at end there has to be an evaluation, this evaluation will include them at first meeting, then from there they have to meet the very stakeholders who are also involved; sit down with them and say, right in the beginning this what we wanted to do, this is what we've been doing but of all this is what we have managed to do and this is what we haven't managed to do. But according to our evaluation as a group as when we sat down we saw that we have managed to accomplish 1, 2, 3. They give themselves praises here and then maybe say we've done badly here then they give the floor to the various stakeholders for them to also be, not to say judgmental but also for them to have an input in the evaluation to say ok ever since we've been with you as a group when you've been there, we've managed to have such and such achieved or you have managed to do a good job here and here, then maybe you haven't managed to make a good job there, you might need to improve here and there. And have that sort of juncture or meeting with them for that evaluation because obviously they have to evaluate themselves first as a group then after that then they involve the stake holders. I think that's how they should do it.

INT: ok. Another opinion?

P2: I think in addition to that;you there are -job descriptions, I think they can also be of help to see if people are performing.

INT: ok. Alright. So what is your own opinion about building primary care team as one of the interventions to improve primary care in Botswana? Your own opinion.

P1: for my own opinion; for once I want to see something that's being done and it works.
Phone vibrates on the table…
P1: not something that's being done and then it dies or something… it disappears, you don't know what's happening, because in most cases you have all these great ideas and mind you they are very great and then to get them off the ground, I don't what barrier lies between getting them of them off the ground and getting that team started… I don't know what happens in between there. Because we have people who are capable, who are actually very capable of doing this because it's… it's… some people might think this is a waste of time teams are always formed but at the end of the day when come to look at it, you can't just come up with ideas and say something and then expect them to take off from the ground and make themselves happen. Something has to make that happen, like that team you are talking about; it's a very good idea to me. My own personal idea, meaning that I would actually love to see that happen.

INTs: ha ha ha (*Laughing…*)

P1: it's good! Because you always have something to fall back on and say, at least we tried, we did do this, we did that and if at all we failed which is not always the case,actually people want results because they want something interesting to happen there and then. But there are those little objectives that would have been achieved, which is something. Well to me, I would like to see it happen. I would, for it remain and happen the way like… I would like to see it, you can have a dream about something… a vision… this is my vision; I would like to see this happening like this and like that because a kere it doesn't just happen. You know these things don't just happen like a week or a month or a year, it takes time, not too much time. It does take time, but if something of that nature is there at least you can see something is happening, so it would be nice to have something like that; my own personal opinion. I would love to see it.

INT: umm question number 11; do you remember any problematic situation in your work, you found particularly morally difficult? If yes, can you tell us about this situation, about what happened? Just your own experience.

INT2: yah we want to know if you remember problematic situation you found yourself confronted with err… you know that particularly morally difficult to deal with.

P1: concerning patients?

INT2: yah in the workplace, it could be patients, it could be other colleagues… you know the

job.

ALL: ha ha ha… (*Laughing…*)

INT2: moral… you know… we know that you could err… we know the codes of ethics, you know turning patients away without treatment…

INT: and making patients wait for too long.

INT: too long… things like that. So you might in you r practice come across such situations that were maybe difficult for you to deal with that you had to do something.

P2: we do have patients waiting for treatment for too long, at times it something you can do nothing about because at times even if somebody is number one in the cure then there comes the accidents, the casualties….. (*voice very low*) and at times this is very pathetic because this somebody who waited… think of block 9, Julia Molefe clinic, somebody has been there at 9 moving and moving and when he is about to enter here is the accident and at times it's not even easy to explain because even if they see with their own eyes and you try to explain to the people that here is the situation… and you a really forced to understand their situation while you on the other side you are torn apart.

INT: but what I wished for was if you could give us examples that you as an individual actually were confronted with situation you found yourself … this moral dilemma.

P3: there are many…

P1: they are just too many.

INT: just tell us one.

P1: yah, well…

INT: you don't have to use names or places…

Silence…
INT2: you are stuck?

INT: they do not want to talk..

INT2: ok, just give us one and it might help others think about…

P2: what would be your example?

P1: Well I have a problem, I have had a problem with a colleague…

INT2: I wouldn't say because I want to get views from you.

P2: a clue…

INT2: let him talk then I can give you an example.

P1: ha ha ha (*Laughing…*) I have had a problem once with a colleague or one of the problems that I've had, I think it was with a colleague umm… I we just changed, people swapped, new people at the work place there and then that person finds that maybe we do things a little bit differently than what he thought or where he had come from. You find some people… I think he was used to la affair then… where I work I don't allow lays affair at all, because I said to him you can't just sit here and do nothing, when it's so busy like this… you are supposed to at least… I mean right now you got nothing to do, why don't you go help somebody else… there people who need help somewhere else, go and help them, and see how you can help them rather than doing nothing when you there is something you can actually do. Then someone says to you, 'if you want to work,work! Someone will do nothing so I lost my temper like man, if you do not want to work and be defensive and he should just go home, because I don't see you getting up in the morning coming to work to just sit here the whole day and do nothing and then tomorrow when people complain that's nothing…you do not work, he would be the first one to jump and say what do they mean yet you are sitting here and doing nothing. He said "he… he…", I said "just go back home, just go home and waste my time or waste our time, we have seen with our own eyes you doing nothing … go home".

INT2:you know moral issues are not necessarily legal issues you could for instance, there was a time in the health care where you would not give family planning devices to young people without their parents; and you might find yourself you know the child is sexually active and you know if you don't give them something they are either going to fall pregnant or they are going to make somebody pregnant and you found yourself faced with a situation where you might give them soothing that you know is not allowed and morally it seems wrong for you as an adult to give a child a… or somebody considered young contraceptives because it's like you are encouraging them to go have sex but as health care provider you are aware that this person is sexually active and they are not going to stop whether you give them today or not they are going to do it. So as a provider you do that or you might have to turn them away that you know go back, knowing full well that turning this person away means more problems for this person than good. So you find yourself facing moral dilemma you know. So these are difficult situations that first you are concerned, if I give them and their parents find out and maybe your supervisor finds out; why did you do it, but if you turn them away and follow the procedures you have your conscience knowing every day, why didn't you something.

P2: I can't think of any but I can remember something that happened to somebody else , I was in a ward with her …… so I called here what is your problem? She narrated the story… I admitted this HIV positive and I don't know if you are aware that people don't like number puncture, so the reading were saying no number puncture and that was the… very much

indicated for… to diagnose a patient and somehow she managed to talk to the mother because it was a group of victims. So she managed to talk to the mother and says… she didn’t even convince… it was the mother who says… she didn’t… and the following day that patient died… and she was in tears, you know I don’t know why I did it maybe she wouldn’t have died, I said are you sure, looking at the condition of the patient so I can see she was also in a dilemma.

INT: in conclusion is there anything else we haven’t discussed that you wish we had?

P1: just about covered everything.

INT: ok, ok we thank you very much for you time and your participation.
