# Supplementary material for: Stakeholders’ Perceptions on Shortage of Healthcare Workers in Primary Healthcare in Botswana: Focus Group Discussions
Source: PLoS One. 2015 Aug 18;10(8):e0135846. doi: 10.1371/journal.pone.0135846 (PMC4540466; doi:10.1371/journal.pone.0135846)
Supplement: S9 Text — (PDF) [file pone.0135846.s009.pdf]

HURAPRIM PROJECT

Type of Participant: HealthCare Users Focus Group2
Date: 10th April 2012
Interviewer: Dr P

Interview Duration:01 hrs.37min.39sec
Audio File Name: Healthcare Users Group2

INTRODUCTION

INT: According to you, are the health workers in Botswana enough? If there is a shortage what could be the cause? A….yes…

P1: they are not enough.

INT: They are enough?

P1: yes they are not enough.

INT: according to you, how do you …..

P1: according to me, I suspect there are certain jobs that they are not contempt with in Botswana, so they end up leaving to look for greener pastures in other countries.

INT: in other countries?

P1: hmm...

INT: ummm... may you please speak louder when you speak so that we can hear each other. What do others say? If you see there is shortage of health workers in Botswana ….anybody to comment.

INT2: wow! But you are quite, you never get sickHehehe... (*laughing*)

ALL: hehehe... (*laughing*)

INT: but really, is there a shortage?

P2: yes...

INT: is there a shortage?

PART(*all*): yes…they are not enough.

INT: what could be the problem…I mean based on your observations…what causes it? What causes it?
P2: yes…I think that the shortage especially at far away places, deep in the rural areas,

at times some people do not want to work there cause there is a shortage…what do you call it…shortages like maybe at Maweneno where there are no televisions…. They wont be…there wont be cellphone reception. I am saying it could be that a lot of them do not want to work there because it is far, there is shortage of services at such places.

INT: hmm... ok what does someone else say?

P 2: yes..i can say…actually it is its not that e can say they are not enough. It is just that the way it is to find services in places that are so remote as they have been mentioning, and at times its because of their issue now is payments, they are the ones that make it as if they are not there, actually its not that there is a shortage. I believe we have many institutions that produce nurses, I mean every year they are being produced, its not like there is a shortage. Lets just ay they are not happy because of ….payments and work conditions, you see…its like there is a shortage.

INT2: when they…when they…when they. If they are not happy what are they expected to do? They do not work they stay at their homes or they work and not do good work? I mean …if they are there, they are enough…

P2: Yes….

INT2: but the pay is not making them happy, so now what is missing?

P2: but if it is like that, if in fact… if it is the case….for example if one of them I will put it that way and I will see that actually in… in the Ministry of Health salaries are not satisfactory, there is a possibility…I will take a different decision, saying "isn it not…it means there is nothing, let me go in to something different or go in to business, you see. Yes…not actually that they are not there. They are producing and some are in Universities they have not graduated but they are there, but they are not happy about payments. Yes….

INT: ok. Is there anybody who wants to add on what P2 was saying?

P3: yes.. personally I believe that there is a shortage because in some rural areas there is only one Family welfare educator, when a nurse come to vaccinate children she is taken far away and she comes once in a month, you then wonder if there is only one family welfare educator and there is no vehicle, you wonder what will happen if a child suddenly become sick when you are that far you wonder what you will do.
P2: I will differ. I think I should rephrase my statement; there is no shortage because…its that we do not…lets avail our resources that..at this far rural areas, then you see that for real there are nurses. If we focus onone district it seems as if there is a shortage in Botswana while they are actually there,actually if we could avail resources at the rural areas, that are..that are…things that are basic that are needed by…. By everybody in life that can lead to them staying there. Its not like there are not enough its because of shortage of resources, there are there! Its just that… its just that…its just that they are not there in many rural areas. For example, I once saw on tv saying the minister.. minister of ehh..of trade and industry went to a rural area saying that Botswana… they tell those at Gaborone that we are still in Botswana you see how it is.. because the resources that are in Gaborone are not available in other areas. Now they are saying Gaborone is Botswana because everything is here. If we cn take these things to them we will also see them working hard. Not to be told that there is no vehicle, which means we wait for a vehicle from Gaborone to collect patients to Gaborone for assistance. Its just that there is a shortage of resources and nurses.

INT: ok. Did..did you want to say anything?

P4: yes… for me I wil say there is a shortage, because our village is closer to…I mean its not that far moreso that you find a Doctor and oe nurse and when it comes time for deliveries of babies the poor person is all alone. Which means she goes with.. with…with the pregnant woman to.. where deliveries are made at.. at Ramotswa, you see. There would come be a patient with athsma in the village..There is no.. There is no Doctor, no vehicle, I mean theses are things are inhumane but in our village we have everything. There is nothing we are short off. Yes...

INT: ok. A.. do you think health workers are posted equally to different villages?

P3: they are not distributed equally like I am there is this area, called Dipotsana. There is a Family Welfare Educator, nurses stay at Digawaneng.

INT: hmm...

P3: she is just 1 (*lifts up the index finger*), she does not have a vehicle, she just stay there alone, but this Family Welfare Educator…..family welfare educator do not consult, she just weigh children and issue children's food.

INT2: is it Family Welfare Educator or Social Worker?

P3: those that wear blue uniform.

INT2: they are family welfare educators.

ALL: hehehe... (*laughing...*)

P3: she does not consult, now if its like that I am wondering if….actually when you come with a patient to her, she then strt calling a vehicle, if the vehicle is not from Digawaneng,it comes from Pelotshetlhe, then you ask yourself that he is not consulting its not in….g…g…he is not a Doctor, if this person can..call a vehicle, because there is a possibility that the person can die here. Because it would not be intentional there will be nothing you can do. Are…If the vehicle stayed with him he could also drive there quickly. Actually….there is a shortage they are transported equally, even these resources of vehicles they are not….maaybe they are not there. How it is going to be I do not know each and every clinic must have a vehicle, a Nurse and Family welfare Educator.

INT: is there anybody who want to add on what he is saying or do you have different perspective. You are queit!!!

ALL: he he he... (*laughing*)

INT: I do not want only one person to answer, I want us to all speak. We said there is no right or wrong answer. Now please I implore to freely participate. Yes… you wanted to say something? (*pointing at participant-2*).

P2: yes..its not that or I will be criticing her, its just that I wanted to comment based on what she said. She says she is not sure whether in her village there is a shortage of vehicles or her. You see how it is, only one vehicle, do you get what I am saying, so this definitely shows that the services and ammunities that can appeal to the nurses are not there. Yes…I, now if the Doctor is taken there there is no vehicle, at times you find

that even the resources in the hospital are lacking. Now the ones that he is supposed to use to help patients are not there…no medcation, absolutely nothing there, what is his work going to be there? I mean people cannot come to hospital and just be looked at …its not …. He should have things that he can assist with, if they are not there I do not see why he should be there, like she was saying there is no vehicle, she doesn't know there are no services.

INT: ok, I will go to the second question. Do you things there are gaps, shortage or problems that related to health care workers in hospitals?

Silenceeeee.....

INT: should I read it again?

ALL: yes...

INT: Do you think there are gaps, shortages or problem or problems that are related to health care workers in clinics? Well we are saying are there any problems that are related to health care workers in clinics…clinics.

P5: I think they have problems because you find that they do not have resources which they can use , even they can prescribe you medication that is not there..

INT: ok. What does somebody say?

P1: well ido not understand by what it means when you when you say there are problems.

INT: umm... let me..i..i..i…I am asking ….im asking …. Umm have they have been trained sufficiently? The other point that you can consider is that have they been trained sufficiently, based on your views, according to you have they been trained sufficiently.

P6: according to me I think they should be trained. They have a lot of education. Yes.. they do manage the job the problem is that at times, at times its like… like I am placing myself in this situation since I stay in the village of Old Naledi, but at times when they are to be placed at Old Naledi they will consider that "there at the Old Naledi, they will not want to go to such a place resulting in a shortage of health workers, saying " we do not want to go there it is difficult, the people there are difficult, we do not want to go there". But they do have the education, they have been well trained.

INT: What do the others say?

P4: yes.. truly they have been trained well, the only problem is manners that are…. They are not patient you see. Because one time I went to Marina to give birth,you see.. and I was having difficulties, when I got there, they asked me how many children have I given birth to? I told them! "o! there is the bed, because I am a woman, you are a woman" you see how it is. Yes… I stayed there for 30 minutes while there was nobody to check on me until my child passed away. When they came to check on me they discovered that her heart had stopped.

P6: I was adding on to the one she said …that our health workers , at times I think its laziness, at times they are they become lazy, but educational wise they are alright. They have some laziness.

INT: ok... are there cadres that lack education compared to others?

P3: cadres for nurses?

INT: yes.. its just that nurses, Doctors, and the others….

INT2: the type that when you went to the hospital you will want them to…. That on that day you are sick and you wish for that person to attend to you.

P3: no Doctors are not enough, moreso that you can go to the clinic at village, I will refer to it because we use it often. When you get there they will tell you to go to Extension 2, they tell you he will come on Friday while you are sick on that day he is not there. Which means you go to Extension 2. When you find it there , he attends to you but the problem is that there will be no medication, you are told to get them elsewhere.

INT: hmmm...

P7: no I believe that even if a nurse knows their job, while she is suppose to attend to a patient knowing the Doctors job…. Its because…its because she will probably say its not her job but the Doctors job.

INT:hmm... ok

P2: yes.. still on this issue of Doctors, really what I have realised is that Doctors are mostly foreigners, you see the ones that come from other countries.

INT: they?

P2: foreigners…yes…those that come from other countries. Now I am wondering if in Botswana is it not possible not to…what do they call it….to remove those that are similar to them that come from outside, foreigners. I was saying are they not around because often when you hear that a Doctor does not have all the credentials normally they will be reffering to a foreigner. Batswana are never talked of, its always a foreigner. And I ask myself what... I mean the institutions that we have in Botswana are failing to produce the same number as compared to the number of people that come from outside. I mean I do not understand. Most of the time its usually foreigners, Doctors are always foreigners.

INT: ok. Someone else?

P8: let me add. Last year.. and if you really want to see the shortage of Doctors, it should be around Christmas time. You will go to Block 8, they will tell you to go to Marina, when go get to Marina they will tell you to go to Extension , when you get to Extension 2 they will refer you to another clinic. When you are about to go in, you will find two Doctors, at times one. When you want to ask, they will say "a..they are not around, they have gone home" you see. It mainly because they are foreigners like my sister has said, that why its like there is a shortage of Doctors.

INT: hmm... ok, is there any….i..

P5: yes, I am adding on to what they have said, them being foreigners again… its their work times they adhere to, a clinic would be opened at 7:30, he will come around… around 8, he will come at 8, at 11:4he is gone he is going to come back at 11:45 having

left patients, then he will come back at 2:30, maybe knockoff at around 3. It now means there are loopholes resulting in patients not being attended to.

INT: ok.

P2: yes..i anted to ask that since it is evident that foreign Doctors are… if its like that what do we do? We say Nurses or Doctors from there do not have the skills like the Doctors from around. I want to know if we are still saying that, something has to be done so that thery are comoarable to those from outside, so that we do not wait for someone who is a foreigner, he wil doo his job at his own time as he wishes, you see. I want to know now when we say Doctors are forigners that we have what are we saying they have done, they have an education similar to people from outside or what are we saying?

INT: no. actually the question was that…is there a certain cadres of health workers that you realise… you realised in Botswana now but now you are saying its Doctors but you realised that many Doctors are foreigners, you see? Now..

P2 : yes … I heard,but someone else says they are there and they know and can do the job. That what I am asking is that since we are saying they can do the job, is it necessary for us to wait for someone from outside being the one who comes and assist on something that could be done by by Batswana, it then shows that the Batswana Doctors somehow have the same credintials similar to those that come from outside, from the way it seems. If they had the credintials we could not be suffering like we are now. We forgot that every year they are graduating, where are they going maybe they went to plough the fields, I wil just say….

INT: now what you are saying is that…your feelings are such that….

P2: what can be done so that the ones that we have here, so that they are in clinics, so that i..it would mean you can move from Extension 2 to Broadhurst, Broadhurst Block 8, Block 8, Block 8 going to village again wanting to see the Doctor until it gets dark still waiting to see the Doctor. We want to to …our own Doctors in Botswana, you see how it is. Its something that is not good.

INT2: but do we really have enough of Batswana Doctors, I am referring to Doctors not Nurses. Remember the forst questionwas asking that do we really have them? Do we have enough Batswana Doctors.

P2: that one I have not done research regarding that one.

P3: if they are there, they are not many. Most of the time we meet foreigners only. When he consult with me what can I say because I do not know English and if there is no nurse you are in trouble. He has to go find her first to come and translate. There are no Batswan. Its here and there that you can meet a Motswana at some times.

P1: Batswana Doctors are not enough.

P4: hmm Doctors are not enough, the other day I went ti the clinic to see a Doctor, they wrote for me the 5th, they told me that when it’s a holiday and we can come next week.

INT: ok... we will go to the third question. Is there a problem of shortage of health workers in rural areas? If its like that, what is the cause?

P3: P2 is not yet done, you want to continue after he concludes.

INT: he hehe... (*laughing*)... I did not see him.

Comment [k1]: ???

P2: now I am not sure because the I do not know if you will be able to answer the next question that I am going to ask. Then I ask….but that INT2 is asking whether we have Batswana Doctors. Now I am wondering if really does it mean that botswaa does not have students studying medicine considering the situation.

INT: INT 2 please answer the question.

INT2: we did not have a medical school, they have been trained in other countries. So we have just started but it has not started to graduate them yet. The first batch to graduate, will graduate in 2014.

P2: so all along we did not have those studying medicine?

INT2: hm-hm... they were going outside the country.

P2: now after they finish in other countries what then happens?

INT2: this is why we are investigating if they are there , do you think they are there since government have been sending them every year.

P2: yes.. but when government sends ….

ALL: (*everyone talking*)

P2: ...it then means something is not done but they are there….they are out there in other countries… yes ..now…anyway.

P3: its okay just drive your point home best way you can, because we are assisting each other.
P2: I mean …i..are you saying every year the government train. If the government is training students, what could be contributing to them refusing to return could be because of the salaries just thinking. Actually we can do comparisons while in the workplace maybe..if could be better if its like this. Yes.. maybe it's the reason why they are not returning.

INT: ok.. umm I will continue with the third question. Is there a problem of shortage of health workers in rural areas? If ots like that, what could be the cause? We are looking at similar points, is the level of services at these places….could they have contribute to the shortage of health care workers?

P9 : it has an influence.

INT2:but the question is that is it… it is just that we were talking about the issue of shortage of health workers in general, now we are asking about rural areas, that do they actually, is it true they have a greater shortage? If that is the case ,what could be the influencing factors? It then means you should answer all these questions.

P9: he he he.. (*laughing...*) yes.. it is always…services also has a influence at the rural areas because you will find a building like this one built, but they wont be where you can consult with the Doctor but there will have a spare bed or two, since when you

leave the Doctor should….from the nurse to go to the Doctor you find that there is where the nurse is located without you being able to consult with the Doctor, moreso that when you are expecting and you are close to delivery you will find that there is no room where they can admit you. And assist you.

INT: ok. What about the others?

P1: hmm..even services at the remoteness rural areas….nurses at the the rural areas always are having it hard. I can give an example of where you are living in Maweneno. The nurse there do not have a house she is rent…she is renting, she rented elsewhere. Also the very same house not having electricity, without anything you get the idea. That is what is meant when it is said that there is a shortage of resources, so that is why it is almost like there is a shortage of nurses in those areas. Someone will want to go to an area where there is water, but now she gets water from a stand pipe.

INT: hmm... someone else…I want all of us to participate, only one person should not participate. I think all of us are not from Gaborone, we are all from outside Gaborone.. Okay.

P2: yes… like she has already said, again..anyway..i once heard an issue being addressed in the news, you see surgical gloves, the gloves they use some time you find that the gloves they use are in shortage in some areas for maybe about a monthonly, when someone thinks of being posted…. Someone will wonder what is going to be done since there are no surgical gloves that can happen to not be there....... at times it is the equipment that is missing for instance when someone want to take an x-ray, if they are..remote villages like… at Mmashoro, they do not have equipment that takes x-ray, which means you come here, you come to get x-ray in Gaborone. Its just afew who will manage, not all of us can manage to get in to a bus and come here…..to come for an x-ray. Even the transport money does cost us.

INT: ok. Now if we look at the distance issue, is the far distances have an influence?

P3: far distance does not have an influence, the problem is the services and ammunities.

INT: ok

P3: like they have been saying, it can happen that when you are such a nurse who…. You can be renting a distance from that school(*referring to Tshiamo Primary school, pointing from UB*) that clinic that is here, you rented elsewhere, you are not in the clinic, but when you are wanted you are wanted in the clinic. It is there, there is no water, there is absolutely nothing. The water is being taken there with a council vehicle like they always say in the radio. The day the water does not come what are you going to do, so you see that its better you request for transfer and move out of this place. Like there is no water in Monwane, it is transported by vehicles. Now a nurse can not stay in such a place. Even the people are just staying there because its there home, there is nothing they can do. There is a shortage of services. In that regard, it means when it late afternoon it means…at…4:30 pm you knock off you got to where you are renting to elluminate your house. Even if there is a problem while she is there, even if she comes running to the clinic to assist what is she going to say to the patient since there is no electricity. There is a shortage of services.

INT: what do the othets say? When you look at the issue of services….i ,ean when you are considering the issue of remoteness? According to you when you look at

remoteness, does it have an influence, I am referring to those that have not contributed yet…

Silenceeee......
INT: ok, nowis the high cost of living in the rural areas…. Let me read it again is it expensive?...is it expensive/ does the highcost of services in the rural areas have an inpact? Does the high cost of living have an influence?

P2: what does it have influence on? Tha the nurses are ….

INT: yes there is a shortage...

P3: you mean high cost of services?

INT2: no, not based of services, by services I actually mean …of high cost of living…when I am being transferred to work in Maweneno, i.. i...the question is saying that…the actual question is that is there a greater shortage of health workers in rural areas compared to urban areas, now we want to see what could be contributing to it? Now we will consider things that..factors that could contribute, but now we are asking if the high cost..i mean basics like sugar, I mean does it have an influence on whether staying there is more expensive than here, could it have an influence on the shortage of workers, actually that I focus.

P3: it has a greater influence because, you are there and you do not own a car like me but it will happen that you will buy food in Jwaneng, maybe you are from Sekhutshaneng you are going to buy food in Jwaneng, you use transport money. When you get there you buy this and that, then you are compelled to hire a vehicle to take you back. Now it has an influence because you can not go to buy without needing transport.

INT: ok, did you conclude?

P3: yes...

INT: I heard you agreeing, may you please give us a reason. Add on to what she said or give another reason.

P10: the other point that I wanted to raise is that when you are doing groceries you have to hire a vehicle.

INT: ok... hmm (*pointing at participant 2*)

P2: yes! This issue is like this, if..when we talk about developed shopping malls here in Gaborone, the one at the less developed villages will have to come from Bokaa to here to shop, because every thing is here. Now this is going to force the nurse or doctor to leave that place and come and work here. If these things were in rural areas I do not see if there was going to be a problem. What is done in cities should also be equally be done rural areas……so that they do not…the persons wellbeing is the one that we give first priority, at times we would say because Gaborone is a capital city we will give it first priority, we avail everything on to it, the others we will see later. Doing that in a way put peoples lives at risk. I mean we will consider that in Gaborone we are doing everything,while the villages remain behind, even the Ministry of Health is there, the other ones health we will see later lets attend to those in Gaborone first. If we considerd that….we stay..we should consider that what we are doing in health at

Gaborone we should also do at the rural areas simultaneously and equally since the persons health comes first. If we say we will bring developments to the Gaborone because it is a capital city that's…at the end we forget the other rural areas that are there…they are there they need to be accorde health services like it's the case in Gaborone. If we share these services equally I do not see a problem.

INT: hm...ok. is there anybody else who want to add ?
Silenceeeeee...

INT: is the absence of opportunities for further education have an influence ? still on the issue of a shortage of health workers.
Silenceeeeeeeee.......

INT: we mean when health workers further develop their academic qualifications, does it have an impact on shortage of health workers?

INT2: it just that this question considers that when you attend school you end…you will often think that you want to develop academically as time goes on. Now what we want to … since all the factors tat can arise can cause a shortage of health care workerske, now they are…they are things that we think that there absence even if I can go to Mamuno where I ca n be able to develop academically so that I progress, is staying there or a shortage of such opportunities could be having an influence on whether people agree or when the Mamuno ticket comes out then you say I am going to work in a rural area. The question is asking just that, if it has an influence or not at all? Its just a question!

P3: it has an impact, when you are placed there at Mamuno, there is no tertiary school, there is no university there, there is no Limkonkwin, there is absolutely nothing there. Its just a primary and junior school, there is no senior school at all. Ther is no where you can academically develop yourself, for you to develop yourself you should take transfer to be near large academic institutions.

INT: ok. P2 go ahead.

P2: yes, I wont differ with anyone, I wil say what I am thinking.

INT: no… mm.. we did say there is no right or wrong answer. Now everyone has something to contribute, allcomments are appreciated.

P2: it is the way I see it…I can just say the shortage of health workers in remote rural areas, its just that I do not know the names I could be saying them. Err..there shortage could be influenced by the fact that they want to advance academically, "lets be closer to the town so that we develop academically". The thing is we should give them….we should build Block 7 like in Gaborone, we should give them electricity and everything. We should avail those that they need. Eee..there should be infrastructure which is for..is ideal for market places, it should show life and development, you see right. You see if its like that I even if I have stayed 10 years in London, even if you tell me that you are posting me to Mmamuno , I will not hesitate because I will be considering the standard of living. Now if I look closely and I am going to stay in thatched house, I also ignore and come and stay in Gaborone, but if the places they stay in, I mean clean environment,one that is beautiful allows them to…it makes them happy, they wont be a shortage rather they will opt to go there. We are trying to improve the situation, just a place to stay! Then lets have tarred roads, we can use a gravel road.

INT: what do the others say?

P8: ee... I will add on to the words she just said that they will consider a place to stay, a person will consider their status of being educated, educated as they are as they are seen living in a place that is not well conducive. This can also lead to people refusing to go to the place, they will say, " what will people say if they see an educated person like me waking from a shubby house without electricity, my laptop will always be off with no television, not having access to the news, you only get access to a daily news after a period of a month, not having access to current affairs.

INT: ok, alright the…the…fourth question. What do you think can be done to improve the shortage of health workers in clinics?

P8: personally I think if they can be given the resources that are conjucive we will never have this problem of….shortage of health care workers. Given services irrespective of the place. Every placed should be ensured that they receive all the services they need, electricity, water and everything, medication in clinics because a person…. Or a nurse or a Doctor will be demoralised, " I always prescribe "I always prescribe medication fro patients but they always transfer to get them elsewhere", it end uo demoralising them, if all the services and ammunities could be improved, they can then have interest to live in remite of rural area.

INT: ok. What does someone else say? How can it be improved?

P10. Hei…at times when you consider where a nurse is coming from, at times when they started working they started in Gaborone, later maybe after two years she is to go there, at the remotest of areas. When she looks at the place, maybe at Ghanzi, she considers that the area that she is being transferred to In Ghanziis…the house! She start by saying " I am from Gaborone , where it is like this, now she must go to the other place…..they just have to develop the roads, be developed…at least a tarred road at least so that it looks better, you see? The house should be fixed, cause maybe ut can be a breeding to go to rural areas, they are just considering the situation, its just that….and you also see that where your child is placed is just to endanger themselves from wild animals and thieves.

INT: ok, what do the others say?

P2: err anyway…I am going to say something again, maybe I will remember something, since we are going to talk about shortage of nurses in Botswana .

INT2: all health workers not only Nurses.

P2: but when I am listening its like we are considering places that are remote, we are talking much about Botswana generally. Because Gaborone is also in Botswana, even if we can say those in Gaborone live well, at the rural areas… the fact of the matter is that health workers in Botswana. At times I just see that…even noe I am talking about salaries, its just that people should be paid because they also have been working and making comparisons with those that work in other countries, they then consider that they have the same qualification as someone in UK, they are paid this much and I am paid this much, that in its self makes them not to have…it does not encourage them to do their job efficiently. At times they even think that, " let me put this nursing aside and look elsewhere or I can go to the Uk". A oerson in the Uk is paid three times my salary, so I am looking at these issues, or Ub by ten times since they get paid even with same qualifications. Now if they take to UK i will just leave them and not come back.

Then Botswana will experience this problem of shortage of health workers, without forgetting remote areas, we are looking at Botswana in general.

INT2: ee... the first question confined its self to Botswana in general, that why I was explaining that research shows that there is a shortage in general, but there is a huge shortage….especially in the remote areas, so actually we are talking about all health workers. But now we….now the issue is what can be done at rural areas since it is evident that in rural areas the shortage is greater than the other areas. But your point make sense since we believe the shortage is everywhere since people are taking about village, the clinic in Village here, but when you go there the shortage is also greater.

INT: ok umm... Training in large numbers and with educational quality to improve on the shortage of health workers? Them being trained in large numbers and provided with a quality education to improve this situation of a shortage of health workers

P4: it can only be better if only the services are going mainly to small villages. It is the only way it can be better, when we train in larger numbers they would leave us here and look for jobs in private companies, you see.

INT: ok

P4: if the services can go to villages then it can be better, electricity, water, these are the most important thing in our lives.

INT: hmm… i want all of us to speak, only P2 does all the talking...

P3: Really!!!

INT: he hehe (laughing...) only P2, these two ladies, and the lady at the corner. May we all speak please, we all know clinics, we have been there, we have stayed at the vill... at different places. Now i want all of us to try to...to remember, we all have to say something, are we together? Yes ..P2 you may speak.

P2: ee.... but as they are saying, training them in large numbers and with good grades can improve the situation but without forgetting to pay them, what i am saying is that they should be well paid, its only way it can be okay. Because a lot of people are well educated moreso that i do not know....its suprising, but for a person to think to go and work, but having spent 10 years in London yet you will be paid P3000.00, it cannot be. The only way is for them to be paid.

ALL: he he he… (*Laughing...*)

P2: I mean it has always been about issue of money, its not like they do not want to help us, actually talk about the issue of ... it is said there is go- slow in the clinics. I do not know how they will help you with their speed cause they still want to be better paid, the real issue is that they will still pursuing it,it is still in there. However they do actually help, but we we... to put pressure on top so that we assist each other. But they should be paid for the situation to be better.

INT: ok. Are there duties that are done by health care workers that can be done by people with lower academic credentials than them? Should i read it again?

ALL: hmm! (*Agreeing...*)

INT: Are there certain duties that health care workers do that can be done by other people with lower qualifications than them? Do you understand?

P11: yes..i think they are there, like to dress wounds, i have an education...i did form 3, i can dress wounds.

INT: ok...

P8: even duties such as weighing children, i think its a job that can be done by someone who did standard 7.

INT: hmm... ok. Umm... anyone else?

P6: i was just adding on to what they said that even checking temperature can be done by anybody.

INT: ok... yes, its just that what i want to...is that if they can bring people who will do all these duties who are not health workers, can this shortage of..workers be made better. If a nurse is there treating people in the clinic, there should be people there who are having a lower qualification than theirs, one who dress wounds or weighing children as you said. Can doing that help the shortage of health care workers?

ALL: Yes INT, it can be better.

INT: ok.

P4: it can be better if you went to hospital to be taken temperature, and BP on the other hand. Someone who is from seeing a nurse supposed to go get medication, will then wait for someone who takes temperature, same person who is going to issue pills.

INT: ok

P4: yes..because when you done…when you are done with nurse, you go get pills and leave. While the other person will be dressing wounds, taking temperature, doing this and that.

INT: the fifth question: according to you, what strategies have been tried in Botswana to solve this problem?

P3: I do not understand(*Whispering....)..*

INT: huh? Should I read again? According to you, what strategies have been tried in Botswana to solve this problem? It means..what are things that Botswana tried to solve the problem we have mentioned. Things that realised government did to try solve the problem we have been talking about.

P9: similar to commities ?

INT: hmm like comitees.

P8: personally I think they have tried I saw from IHS students. Yes, it was a great effort since the students were being taught. This is the first effort.

INT: yes, someone elsse?

P9: even this school of….what do you call it? Boitekanelo. They do take temperature.

INT: ok.

P11: even the home-based care committee, tey helped a lot because they could visit patients at their homes. If it is a patient who is terminally ill, they could bath them and do there washing.

P3: the problem will then be money as the P11 has already said. They have offered their services and they are being renumerated I do not dispute that. They…every morning they attend to patients, they bath them, feed them, maybe…maybe the patient living with children, you know people can die. I can die leaving my old mother with children. As young as they are the home-based care people take care of her; bathing her, feeding her, washing clothes. Then they will apparently be thanked with P40.00. later they give up saying, 'i am just helping there is no importance", they will then go do there own thing growing vegetables and selling them in front of Supa Save.

P2: yes actually KFC is P26.00, when you give him P40.00 to buy…it also means they are having good food again…is given some more money so that they are able to buy toiletries and…..

P4: even to take care of the family, you see.

P3: even to take care of the family, the problem is he will be taking care of me, when he…while his job has stopped while he could be doing it for himself, he has taken care of me even if he is not given anything.

P4: with all his heart.

P3: with all his heart.

P2: ...'cause when he finish working he has to reward himself here and there, the money is now over, you see.

P4: even them at home , its not like as they have availed their services its not like they are…having a caring heart because they are parents. Children are waiting for her at home for her to provide. Even P40.00 does not buy 12.5 of maize meal, you see how it is.

P3: even the detergent they are buying for the patients they are using there own money. They contribute money for this patient. You can never recover the money; there is no where you are going to get the money. The only way is to try to live with the little that you have .

INT: is the establishment of a school of medicine, the one that we are in right now, can it...is it a one of the ways the country is trying to resolve this problem. I mean baes on your views, that the school have been completed for training Doctors, is it a way of improving the health situation.

P3: it could improve the situation, the problem is the salaries. They will be tsught and not be paid enough. If they are not well paid here, theirs peers will be attending schools in other countries, now when they completed school they will tell their counterparts that they worked aftercompleting in those countries and now they are getting something. The one trained in Botswana will remain in Botswana and not be paid equitably. They will have problems moreso that they also end up going thre too.

P1: actually it could be much better, I mea this school. If they did not hear from their peers for them to migrate to such countries, like when they complete and graduate in 2014, and they..government recognising them as nurses, our Doctors, so that they do not follow their peers they should be equitably paid also. They should not leave us, they have been trained here, they used our resources in…. here in the University, since when they complete they go to other countries, so when they graduate they should also be well paid so that they do not go anywhere.

INT: ok. Hmm... what does someone else say?

P6: I was saying they would be given this better education , when they complete they will be taken to these places where there are no developments yet they are educatedthe very same person end up leaving the very same place because of a lack of developments and better services, there is no electricity. It will be the same as never having gone to school..

INT:ok.

P2: ee…I can say the completion of this school has an influence. Yes,they are doing something, it has an influence to improve the situation. Now, I am wondering after they complete here and graduate , where can they get jobs? Because the what I suspect is that they will either go to GPH or Bokamoso, ad that’s one place I can never go to, because they are going to say I should pay P5000.00, because if i am going to ask for minor help I should go to Marina and …you se. now I am wondering when they finish graduating them and take them to Marina and the smaller clinics, and give them something better, “they will want a salary so that they have better payment, ‘because at GPH they will maybe I don’t know around P15000.00 now they will bwe paid p3000. they can not go anywhere with that amount.

INT: ok. And then INT2 this point.

INT2: which one?

INT: B, the one that clinics used to underrrr…

INT2:is that so, another person….we want to know programs, or or pillars, or what are they called the ones they have tried, that transferring clinics from the Ministry of Local Government to Ministry of Health has it done any difference. Because these are the things that are done to improve or have you noticed any difference.

P1: there is no difference and actually its actually getting worse. There was a shortage of medication which, there is no difference, the way I see it there is none.

P4: and it was the first time we saw a strike involving both the nurses and the Doctors you know, ever since long time back, they say in all distrits and small areas, it was the first time we saw a nurse not caring about a patients.

ALL: he he he (*Laughing...*)

INT: he he he(*Laughing...*) ok. Can I move on? Is there anybody who want to add? Ok. Did the plans work according to you? Why are you saying that? We mean the initiatives that we just mentioned, did they work?

P3: initiatives for what? For merging different departments or what?

INT: all that you have mentioned. Does the students of Boiteko…

INT2: Boitekanelo.

INT: Boitekanelo, what is the other one? Initiatives such as home-based care, did these programs based on you?

P3: they do work, very much. They are hard workers those people. The problem is not being renumerated accordingly. They have worked because now there is no patient who is kept indoors, they would rather volunteer and hire a vehicle for her becoming her parents and taking her to hospital when they see that she is critical. The issue is that we would be volunteering our time, we contribute as we are contributing around P1.00 so that we hire a car for her, after this we don't benefit anything yet we are …. We are supposed to be reimbursed here in home-based care or the VDC. I will be given very small amount of money that …the money that I used to assit the patient you will never get back, now because its love god would have volunteered to take care of that patient. They are very patient. I wll be given small amount of money more so that… the money that I volunteered to assist a patient I will never get back, but it's the love of god and has chosen to assist such a patient. They are very patient but if they can be given a raise instead of being given P40.00, they can… because they have volunteered with all there caring and love. Now when they are suppose to be renumerated, they are not given anything, they are given the hungry lion money that my brother was talking about.

ALL: he he he (*Laughing...*)

P3: my child at home is not eating anything because I spent the whole day caring for ap patient of..she is ours because she is from Botswana, I have volunteered for her and spent the whole day there and I do not know if my children have eaten or are hungry. When I come back there is nothing I can thank them with, "my children I am from Patient P and I found her not feeling well so I spent some time there, take and buy some mealie meal so that you can cook.

INT: what does someone else say, when you looking at government initiatives that he tried to use and that actually worked, the ones that we just mentioned, looking at home-based care, HIS, Boitekanelo training institute.

P8: personally I think they did some progress, all that have been willing. They did something big because, because their lives have been preserved by home-based care people since they could not take them selves to hospital, they took them to hospital. They have done their part.

P2: hmm if it were not because of them they could have died in their homes.

INT: ok

P3: they do work hard, if only they were well thanked, they could grow more.

INT: ok. The seventh question. Based on you, what can make a big difference to improve the health workers situation in clinics?

P8: personaaly I think, we are moving backwards, if they cpuld be given more resources In clinics, their health care workers will..they would be motivated, they will then help with all their hearts until patients get their medications, if one is for x-ray, they take their x-ray, everything, knowing well there health status. Given more resources and money.

INT: what does someone else say?

P3: resources and services should be increased. Even if they can be hired they become many when there are no resources, there is nothing they can do because what they can help with. Now when they, their services should be increased where they work so that they are contempt without not attending to patients. When they are not contempt they will leave I assure you. Even the Doctors that we are educating, if they do not get satisfied they are going to leave us here and we will go through the same ordeal of trying to improve.

P2:mmm….it is like that, you said a good thing P3. Now the thing is we are going round in circles, we are hearing each other here, the main issue is, leys raise there salaries and ensure that the remote areas have all the resources they need, but everthing starts at the Ministry of health, if they do not make sure there are resources, otherwise there is no way the nurses will be able to assist. They should make sure that there is medication, in each and every hospital. Doctors should be in every hospital. If it is like that, there is no problem, the thing is we do not know how they see it at the top , they should make sure there is medication, all the resources are in all the hospitals. If they do not re-consider this issue, there is no way we can help. Now we are going to tell you initiatives that we know may work. We wish they could work.

INT: ok. The eighth question. To form Primary health teams for health workers it is considered as one of the ways of improving health services in Botswana. What do you understand about Primary health teams for health workers? They are the commitees, right?

INT2: no they are not commitees, rather Primary Care teams.

INT: could you please be more specific?

INT2: should I clarify it? Since we have been talking about improving human resources, we have talked about some such as better salaries and provision of resources , and others. Even if those are there, they are a lot of things that needs to be done to better the situation. Now something that was once thought to better the situation of human resources is to come up with Health teams, moreso that in every clinic you will find such teams, maybe the team will have….Just giving an example, maybe it will be a Dentist, Paedietrician and others. So I am giving an example that way out there so what do you understand about.. Actually the first question was asking what you understand about these teams? It then means I have answered it because it means these teams should have….. so that you know for this clinic I will find I have a team of health workers, I will be helped by the team members and it will consist of what kind of people. Consider a football team, you know that in football, I

know that when a team came I know it means the goal keeper has his role. Even there, these workers can be made in to teams can it help improve the human resource aspect of health.

P3: it could help because the nurses are short tempered. Some are so shrt tempered, but maybe when they are groups they can remind each other that no way to be treating patient, this is how you are suppose to do it. Not when we get here in the morning we start working, I am going for temperature check there, someone goes there and others there. They are very short tempered more so that at times when you ask where you can get particular assistance they will tell you to go that way.O a tsamaya ga o itse gore o yak o go mang, o botsa yo mongwe fale e tla re a go tlhomogela pelo a bo a go raya are “nnyaa mme go tsenwa fale”. Jaanong gongwe fa ba le ditlhopha e kare ba ka ne... e kare fa yo mongwe a go felela pelo yo mongwe a go tlhomgela pelo a go thusa. Bone ba fela pelo thata, thata le gone.

INT: ok.

P8: I will also add on to the one that she just commented on regarding the short temperedness of the nurses. I think when they health teams, its because I remember at Old Naledi clinic there was aa men who wanted, they had long stayed at the dispensary counter waiting for pharmacist who nobody knew where she was. He then called a male nurse who was giving injections, he then asked……we have long stayed here maybe you should help us since we long waited here, iyoo! So that it be like…he then he only learned to inject patients only so wait there, she has learnt to give medication.. Now I think if the team could be something efficient. But when they are individuals, one will do whatever they are doing, everyone will come with their own mentality. If they are together they will advice each other. One will say this is how we treat patients, and all will be good.

INT: ehee...

P8: yes…I thought somewhere it could work like this, I do not if the whole day there would be someone who will check , or people instead of an individual, they are human so they can not manage alone, who checks if there is a Doctor, is there one who inject, there is a pharmacist. So if these were the things they chck everyday to avoid me going to Block 8 and being told that the Doctor is at Extension 2, when I get to Extension 2 they will say he has gone to Broadhurst.I mean they can make sure that everyday everyone is in there position of specialisation. Yes, for instance the Doctor, pharmacist, one who weighs children, its something they check so that we have…when we get here In the morning we be told….if the Doctor….so that you are told in time that there is no Doctor so that you do not stay and later be told to go to Block 8. Its things that we should make sure that those people are available , because we also incur costs, going around until 5 looking for a Doctor who is not there.

INT:who shou;d be in these teams? Who should constitute a team? Whether it should a team of nurses only mixed with Doctors and others that…..what do they call them? Family welfare educators who are in shortage.

INT2: the ones they want, so that the team have specific people in them.

INT: having a defender and all that.

P10: I think if the team have nurses, doctors and people from the village, they should be included so that they can watch over nurses to see if they treat patients well.

INT: yes..you wanted to say something?

P1: yes, the very same team should have Doctors and the Nurses, there are also dentist, optometrists. I am saying if we are going to say it is a team it should consist of, in that team .If we are going to say it's a team it should have, actually in that team every one who is going to part take, like an optometrist, and other speciallizations, one is a Dentist and so on, then they will all be in this team., so that they can not say when a patient has tooth problem therefore go elsewhere to get you tooth removed,you see. That how the team will work, all those that are there in that team.

INT: now who can lead such a team? From those that…that you think … who can constitute this team? I mean looking at Doctors, Nurses, different people and even the community.

P3: a! they can be led by Doctors since I will be part of the community…I volunteered. There will be times when I will not manage to come, the Doctor works everyday if all goes well considering that they should be there as a provision.

The Doctor will lead this checking whether they are all there, do we end up with a certain number. From these nurses do we have one who takes temperature and one giving medication, and the other weighing children, whether everything is there. While I come from the village, yes the village people may not be there, but they can not be there on a daily basis. At times in the morning at home while still preparing porridge for the children, you end up cleaning so that when you there the children can have something to eat.

P2: yes, you are talking some sense P3, actually it should be headed by a Doctor, moreso that we should make sure that, to ensure he is always there because he is the most needed person in the hospital, maybe if we give the responsibility it will ensure he is always available, so that he does not have….have…err we gave him that responsibility we wont have difficulties since the Doctor will be there, it okay that way.

INT:hmm...ok what does someone else say?

Silenceeeeeeee...

INT: ok. The ninth question. What are your impressions about the establishment of Primary health teams as a way of improving services in clinics?

INT2: but maybe when we go a bit back, we are talking about small teams, if it was soccer you wil know the team is working after you have won the league, now how are we going to ensure that the services they provide is the one that we need? Do you what I am trying ti say, I mean we can form a team and…so how can we ensure that actually the services are actually working.

P3: we can only understand if people are placed in to that group, When I go to a clininc at Tsholofelo, I will not get the assistance that I can get from the Village clinic, one day when I am walking and someone asks me I will then say but when you go to the clinic at Village you will get assistance fast you do not spend the rest of the day there.Now when you there you will spend the whole day there more so thea you end up polishing and shining seata all day. This is how we can establish who are more productive. Then the Ministry of health will then know that a particular clinic is efficient in service

delivery. Then the Ministry will then know that there is a clinic there that is better where they can get some productive ideas, then they can get appropriate actions against the other groups to say why are they perfoming better and you are not.

INT: hmm…

P2: mmm….you are telling the truth. The way it is maybe after a certain period of time, you go in to a certain village, you go to people, you then ask the residence impressions regarding the clinic.So that they can tell, "they are being helped or not", the other thing will be to determine if the group formed there is perfoming, even when you go to Extension 16 or Old Naledi asking residents of Old Naledi's thought about their clinic, maybe that "yes indeed we are getting help or we are not getting any". This can determine if the group works and works for the good of the community.

INT: I will continue with the ninth question. What are your thoughts and feelings regarding the health teams as a way of improving services in the clinics?

INT-2: basically is it something that we should be encouraging the ministry of health to come up with these teams of health workers, is it something that you think should be encouraged?

ALL: yes...

P3: very much…

INT: one of the pillars of the Health service act of 2010-2020 is to adhere to provisions that guide or that control the health services accordingly. Shortage of health workers can hinder the implementation of such provisions. Umm… question 10, since they have answered the other one?

INT2: yes that one they have answered it, the one that you were reading concides with question 10.

INT: ok. Question 10, do you remember an incident that happened to you as a patient, someone that you saw, or a patient that you took to a Doctor, where by a health worker treated you in a degrading way, one that is discouraging,one that discriminates, or one that be-littles? Well remembering an event that you were not treated well at the hospital or clinics, even if it was not you but someone that you know.

INT2: its just that the mandate of the Ministry of health is that people shpuld be treated in a way that is respectful to the person's wellbeing and integrity, now we will like to find out if these mandate is being adhered to.

ALL: she is not around…hm-hm…she is not around INT 2, not around.

P3: the lady I work with once came crying intensively when she was from the extension 2 clinic.

P4: I had influenza that was like TB, then i went to the clinic at extension 2 for consultation with the Doctor. When I was consulting with the Doctor he asked if I had

tested myself for HIV, I told him, he threw me two tubes, “go and check yourself, go spit in the bottle do what and what” more so that he did not give them to me, giving them to me to ask what to do with them, you see, to ask from the woman that took my temperature.

Hey! She then ran to the door and opened it, then she put the musk on. Heee! Now what is happening because I have TB that is very rear, then I went this side….she said to me she is new in Extension 2 and does not know what the processes there are.

Then I went to the lady that is taking patient temperature and told her that the Doctor requested that he shows me how the tubes are used, they said I should go have an x-ray, but I do not know the x-ray screen, I did not what I was suppose to do with those tubes, then she said “ijoo! Madam I am also knocking off, go and ask someone else”A! I went to ask the security lady, then she said “why are they treating you this way, cause the other day they helped well when I went through the same process. Go and ask”. One lady was wearing purple uniform, then I went to ask her, then she said “madam this Doctor just arrived from overseas”… he was tall and dark, I thought he was not a Motswana. The Doctor was a Motswana. Then the lady helped me then I went to Marina and x-ray was done and I handed over the tubes. Then I went to the person that collected the tubes, then I told the person, who then said I should have told the Matron. Then I asked how will I know a Matron that I have never seen, there are no manners in our Doctors. I felt disheartened.

P3: they lack manners, she came crying but when they assist you they will push tubes on to you, take take and stay away, they take your card and never even open it, when you are sick, while you are sick more than anybody it will be as if it is the first time for you to go in to the clinic. .then she will leave you there, you will remain there, whom do you ask? You say you are going to this person, when they knock off my time is also late, you see, what is that? Its disrespect to patients and to themselves too.

P8: ee…I also want to add on to that one of health, our health workers, at times its at…. It can happen a lot at martenity. At times when a woman is in labour pains being untreated, they will be looking at your card, looking at your age and looking at whether you have children or if have not had a child.If they realise you have a child , they are going to tell you things that you will feel that if God willing you be free if only I can see myself free from this lifelong and, feeling that you are not welcomed at all. The pain you are in, they think you are playing.
Someone will tell you to be serious, you better move there if you are here to play. Telling you that while you are in labour pains to be serious, otherwise get off the bed I am helping a lot of people this side.At times you will think that assisting in giving life on to this world should be something that makes one happy, she has assisted you and it would be like …. Its like some are feeling sad as if you done wrong by someone in life..

P3: but as a job that he has agreed that I will help these people. But they should be helping people with care.

Comment [k2]: DELETE??

Comment [k3]: DELETE??

P1: No health workers, they lack manners. Last year December I had problems with my eyes as they were hurting, then I went to Block 8; the lady then…she is not a Motswana, she is a foreigner from Ghana. So it ..it was…it was on a Friday. I spent the whole day waiting for the Doctor…the was long line.So it means I was attended to around past 12 to lunch. I then went in to consult with the Doctor, and she said I should go to the clinic, then I said the distance to Marina, I will find it closed as it closes at 4:30, I will find the hospital closed, then she asked me what she is suppose to do, then I said,she…I do not know what to do, I am suppose to be at work , at least write a letter for me that I came to the eye clinic on Monday or clinics does not open on weekends.Then the Doctor took my card and wrote sick leave. Then I told her that this card I am going to show to the my boses at work you can not write sick leave for me on a medical card I should show my boses at work.The Doctor then took my card and threw it away; I then went outside calling…. I asked the person who was taking temperature, madam me and the Doctor are not treating each other well, I was asking her to write for me sick leave as I wanted to see the Doctor, I will go to the eye clinic to Marina on Monday.The nurse went with me going that side and she said she can not locate the sick leave papers which means that Doctor, when she pulled the drawer she was next to, when she pulled the drawer she found the sick leave papers which means that Doctor….she asked her to fill them for me and she refused , that nurse….she was apparently the supritendent or what do they call them? She is the one that filled them for me and stamped it.Then I told them I am not leaving until they tell me why the lady was behaving that way, then she said “we will talk to her later”. This year again when I went to Block 8 I found her again, I had a splitting headache moreso that I felt as if I was going to die. She then did not prescribe pills for me saying I should go to Marina. I told her that I am not leaving until she gives me at least one pill that I can take. The Doctor at Block 8 does not have manners, even now she is still there..

P3: there are no people…

P1: totally they do not have manners. People could be many, some were police, the other person came from somewhere angry and wanted to beat her up the Doct…it is a woman…not a man. They totally lack manners.

INT: ok.

P3: at times I wonder when someone is a Doctor and knows very well that he is suppose to attend to patients, and when his duty time is done he will just leave patient saying he is leaving while there is nobody there. Based on Botswana laws as a Doctor, ae! Since illness does not have aholiday , it will always be there everyday, now you will find a Doctor not attending to you saying its time up he is going, its time up!

P4: for me people were in front of me, easedropping, “yes, here he is” twisting this way and that way, since I did not know them I decided to enter that side.

P3: but you had caused problems for yourself.

P2: I agree with them, I have once an incident happen in extension 16 you see, it was a

Comment [k4]: ??

queue, it was around to one, that Doctor just passed us, maybe I do not know maybe he is the same Doctor that the lady here is talking about, he is a giant he….

P4: the giant tall one, he is dark in complexion.

P2: he just left, holding a cup, he went outside the clinic, if it were not for the cleaning ladies, we would have never known that he had left. They told me that the man was going, if it were not for them, we would still be there. I mean this does nit sure respect. Its like we were stupid, he does not…at least when he would have said to us that he is leaving we would have…then we would have left.

INT: ok, another person?

P4: Again you can sit here waiting for the Doctor, people were go in, when someone else was suppose to go in she was told to wait, he will leave with the back door and go home. You will remain behind waiting not knowing that he has gone, at Extension 2 here..

P3: This clinic, but they say it's a big clininc. No but its like that, I once I took my husband to the clinic when he was sick he ended up passing away, sick, he left us here leaving with that door, when the nurse tried to check him she found nobody, she is the one who organised the vehicle so that she takes us to Marina since it means the person has gone. When ..personally I deem it not necessarily for patients to be left in a queue. When it comes to the back door, the dorr should be right here, he should leave here. At least when he has left, you will give up, now if they left with the back door you willnot know whether the person is there or not.

INT: ok, is there someone who still wishes to add something? Ok, if there is nobody, we have come to the end of our qustions, umm …if there is anybody who wishes to ask a question feel free to ask?

P1: I want to know about this school of medicine after it graduates students, Batswana Doctors are not the same as foreign Doctors, they are well mannered more than…I have never seen a Motswananbut he does that in another fellow Motswana, they should be looked at so that people get to see them, they should not go anywhere, they should remain in this country its theirs, they should come and assist us like they have been doing.

P11: yes, I mean for example, if you could go to the clinics, its in block 6 right, there is a Motswana, that man who wears glasses, they are well mannered you see. They are Batswana man, they are Batswana Doctors.

INT: Ok we have come to the end of our discussions.

THE END!
