# Supplementary material for: Stakeholders’ Perceptions on Shortage of Healthcare Workers in Primary Healthcare in Botswana: Focus Group Discussions
Source: PLoS One. 2015 Aug 18;10(8):e0135846. doi: 10.1371/journal.pone.0135846 (PMC4540466; doi:10.1371/journal.pone.0135846)
Supplement: S10 Text — (PDF) [file pone.0135846.s010.pdf]

HURAPRIM PROJECT

Participant ID: Gaborone Focus group one(1)
Date: 05/04/2012

Interviewer: Dr N

Interview Duration:

Audio File Name: Focus group Users of Health services (1)

INTRODUCTION

Int: I am going to ask you questions about health workers in your district. Please answer all questions in detail. Your truthful answers will assist the Ministry of Health to understand the position of Health care workers in our country. Research in Botswana has shown that there is a shortage of primary health care workers (like at the clinics), especially in the rural areas. The first question reads: according to you, are health care workers in Botswana enough? If they are not enough what could be the cause?

P1: they are not enough, it's because of low level of education, and we have only one institution that train health workers. And it also train workers in less numbers, it trains workers in large numbers.

P2: the other problem is that after health workers are trained they end up going to work in other countries because of the remuneration they are given in those countries.

Int: Is there anybody who wants to add something? Or you are agreeing with them?

ALL: Yes! We agree with them.

Int: Are they really trained in sufficient numbers?

P3: the numbers are there, the problem is the remotest of areas, there is no…there is a shortage in the numbers of health care workers in villages.

P1: the numbers are low, health is similar to education, and institutions for health should be increased so that we have sufficient numbers.

Int: is there anybody who wants to add on what he has said?

ALL: we do agree.

Int: Are they equitably posted to all the places?

P1: No..! there are villages that you find having Nurses and Doctors, while others do not have Nurses and Doctors, while others do not have Nurses in some places like at the rural areas, they are not distributed equitably.

Int: What do you think about worker's long stay in their jobs?

P3: Eee… if you stay somewhere, you get used to the place and gradually become lazy in your job.

Int: What do the others think?

ALL: We are agreeing.

Int: I will go to the second question; do you think there are gaps, shortage or problems that are concerned with health workers in clinics? Are there shortages of health workers in clinics?

P1: Yes! There is a shortage; there are places that have a clinic with only one nurse. Nurses only do the job they are trained for only in the training institutions. There are different cadres of nurses, they are not enough, doctors and nurses are not enough they are found far away.

Int: Aa! The health workers are doing the jobs they were trained for?

P1: Yes! You will hear a nurse saying they have trained in general nursing, they will consider this. They will not consider that if a person comes with a condition that requires a midwife, I may help, but the problem is they become focused only on what they have learnt at school.

Int: In other words, they are doing the job they were trained for!

P1: Yes! They cannot expand their. They cannot at least.. (laughs)

Int: are there certain cadres of workers that are in shortage more than the others? Are there certain cadres that lack more than the others? I heard that there are midwifes there is…

All: Yes very much, very much

P1: there is a greater shortage of nurses. Dentist are nonexistent, you find that you long suffered from toothache you will stay at home waiting for the nurse to come from Molepolole…

AP: So that she leaves the urban area to a rural area.

P1: maybe she came from rural area..

Int: aa…let say someone speak

P1: Now if you are suffering from that toothache now you know, now you know why there is a shortage, they are not enough.

P2: yes! Even Doctors you find that they are not there in hospitals, I do not where they were specialized you will find that they are not there….

All: especially in hospitals..

P3: even Doctors in the rural areas, so that if he comes from the urban areas going to rural areas, maybe coming on Wednesday, now if the Doctor would stay there at the village would be much better.

AP: it could be better

Int: Yes… aaa… does the workers get enough assistance, resources, health user's leadership, health workers do they get the necessary assistance?

All: mmmh…

P2: At times when you there you will be told that there is no medication, you will have to look for them at the Chemists.

All: mmmh…

P3: at times when we speak of resources, you will be told that. Especially as a woman who uses family planning, you will hear that they only have three instruments they are going to be sterilized in the clinic, at times you are given expired medication then you ask yourself..! Shouldn't this person have realized that this while the medication was still in the shelves, you will only realize at home.

Int: Ok..! is there anybody who wants to add on to what have been said? Alright ! Do the work conditions and remuneration enough, I mean work conditions and remuneration…payments.

All: you mean for the workers… (laughing)

Int: health workers!

All: maybe they are discouraged, maybe they are discouraged by….because of

payments.

P1: mmm…

Int: ee! I will..

P2: we hear that the payments are low but we do not know how much they get paid!

Another p: that is the thing.

P4: we do not how much they get paid!

Int: I will go to the third question which says; is there a problem of shortage of health workers in rural areas?

All: very much!

P2: Aaa!!! Yes! They are there because..

Int: ee… what..what could be the cause?

P1: people love the cities.

P4: people prefer the city.

P1: people don't, they do not want to work at the villages…

P4: when you tell them to go to Artesia they refuse.

AP; When you are to take them there they refuse, I want to go to Gaborone and Lobatse.

P1: that's why you see them leaving this side and leaving for private companies mmm…because they want cities, you cannot be simply be taken to a place like Ghantsi so far away. Maybe you are person who comes from Bokalaka, now you will see that ehe.. you what I will quit and go to private companies.

All: mmmh… or maybe go overseas mm..

Int: Does the level of services in these villages have an influence on the shortage of health workers?

All: that! Very much!

P2: at times you are taken to rural areas where there is absolutely nothing, there is

no water, there is no electricity. That why people refuse to stay in the rural areas…

P4: there are no roads..

All: there is nothing…maybe at the villages…a place to live mmm…

Int2: you were about to say something.

Ap: yes! I was saying also a place to stay

Int2: Ehe! A place to stay

Int: Aa! Does the distance of villages have an influence on health workers ending up staying in areas closer to to urban areas as compared to staying in the rural areas?

All: very much!!! Very much!!!! (laughing)

P5: personally I don't think the far distance, there are roads, there is a problem because of developments that are there….

P2: actually the farness…actually farness…

P1: very far away places….

P5: it can be considered…

P1: it will be better provided there are basic needs. If there are at least it is a bit better provided there are basics needs you see. When there are roads, electricity then you will now know they won't mind the problem might be if it is so far! Without anything. Where they call remote rural areas! Some distance they do not mind

P5: mmm… there is no electricity, there are no roads…

Int: Could it be expensive or because of high prices of goods and services in rural areas?

P2: no, even if it's not only prices, at times you will find there is no transport you see, you have to hike in the road and thing s like that are not nice.

P6: Yes! At our village at Mookane tarred road is used by nurses to come and go, come and go because they are the ones who have their own vehicles, others vehicles are destroyed by the gravel…

Int: Alright! Aa… is the opportunity for academic development or its absence

could It have influence?

Int: the availability of the opportunity or its absence for continued educational development because at the rural areas there are no schools.

All: Yes! It’s true…

P1: I don’t think so because with technology you can study online, so I don’t think it applies you can always read online, you will ask for a day off, so that you go write the examination , I don’t there is a problem…

P2: stil still even if technology my dear, some areas do not even have a computer, there is no how you can setup internet, you see how it is.

P6: Mmm… there is no electricity

Int: mmm… I will go to the fourth question; aa! What do you think can be done to improve the shortage of health workers in clinics..

Int: wait first! I was saying…it’s like we have skipped something, we want to know if….to live in the rural areas…..are there opportunities for jobs for those accompanying the health worker, and those for the children like schools, does it have an influence? Schools for the children yes they have an influence on whether someone can stay there….at the rural area or what?

P2: mmm… if I understand well yes! I think everyone wants their children to live well! You see. If you then take your child and go live in the rural area , you have never went to see where she will be going to school, you won’t notice any difference in the child, it’s not nice you want to see them excel at that age, and nobody want to take their child while they are living better to….

Int3: actually it makes their job easier, let only one person talk so that they are able to write well, and you should also raise your hands so only one person talk.

P3: point taken

Int2: ee…its okay it is acceptable

Int: yes I will I will….

Int2: yes! You can continue..

Int: I will go on to the fourth. I had already said it, but then we went back a bit. Aa…what do you think can be done to improve the shortage of health workers in clinics?

P1: Aa! Aa medical school should be opened, you will see at least there would two institutions that train health workers, it will mean when others go to HIS while others will go to UB, obviously Ub will get a considerable number of students so it means the people….the more there is more education for nursing, the more our standard will match for the other countries.

P2: ok! You will also find that people who are studying medicine, the doctors they don't come back home those who are studying abroad, I know a few of them, maybe they could be forced maybe it will be much better.

P3: I will will add on to….actually there were some students who have assisting nurses with their duties. They took temperature, and then the nurse will be injecting patients. There are no longer there who did not go to HIS, by reducing pressure I mean they should be returned as they reduced congestion in clinics.

Int: Aa… can training in large numbers improve the situation?

P1: Ee… like she saying I think ee…but it means let's not only teach people who are going to be doctors and nurses, like she was saying lets take other people maybe those that finished form 5 and did not….they will be just taught so that they are the ones that will weigh children…so that the nurse does not do everything, you see. I think the more people are educated the more we will have more people in the clinics.

Int: ee… is there anybody who wants to add on to what they were saying?

P3: even the campaign that we do for us that weigh children after six months, children get that.. Since the nurse will go to weigh…. (laughs)

Int: Aa…can deploying workers to all rural areas help the situation? To deploy workers to the rural areas?

P2: Ae… it can help! Maybe I don't know you will find that in in in other countries because let say I am from from the kweneng district you will be posted in to the same district that you come from unlike maybe when you are from far away like Bokalaka, then you are posted beyond Ghanzi…If when you are given a post you are posted to the district that you come from, that what I'm trying to say.

Int: Eee… what can be done? Or you wanted to add!

Int2: Aa…do you agree that we should give these people a chance?

You mean people are given the chance of choosing to…

All: Ee… (laughing)

Int: mmm… what can be done so that workers in these rural areas work there for a long time after they are not posted there?

P3: maybe maybe if you could develop them, maybe build for them, I am from far away nurses stay In a room That is one, at least as government do not build roads, you should improve their houses, so that you use solar power system, give them certain things so that they also get things that are available in urban areas Like generators, and other things even though they cannot get access to crèches and roads. Where they are posted there should be better houses at least or clinic. At least the clinic in our village even a nurse will not want to stay there.

Int: what do others think? Or do you agree with her on what she said? Ee… aaa… aaa… there are certain duties that are done by health workers that can be done by other people people with a lower education.

P1: question again…

Int: aa…are there duties done by health workers that can be done by people with qualifications lower than theirs?

P1: Yes! At times there are there.

Int2: like what?

Int: like which ones?

P1: taking temperature…

Int2: ok P1..

P1: taking temperature, taking temperature and to issue supplementary foods.

P3: at the hospitals like bathing patients, so that we relieve the nurses as they are being interchanged…

Int: is there anything that you can add on?

Int2: they are very useful answers, you can continue Mogomotsi.

Int: eee… I will go to the fifth question. What initiatives have been tried in Botswana to resolve this problem?

P3: I do not know, we saw that they had tired recently with graduates that we have been talking about today, but they are not there…

Int: which…which children?

P3: its just a problem, they say the ones that were wearing blue uniforms…they were a real effort, I think even those that were taking temperature, not a nurse going back inside then to consult for illneses what is happening today.

Int: eee… Can remuneration for duties performed be a likely solution to this problem?

P2: Yes! Money is a …… (Laughing) if the money is there, you have to do your best, and if there is no money you will drag your feet….

P3: that why they end up going

Int: eee… eee… transferring clinics from the Ministry of Local Government to the Ministry of Education… Could it not improve the conditions of health care workers?

Int2: Do you understand this question? The question is saying transferring clinics from the Ministry of Local Government to Ministry of health

Int: Ministry of health

Int2: transferring it from the ministry of local government to the ministry of health…the question we wants to know if the transfer of clinics from the ministry of local government

To the Ministry of health…the question wants to know if the transfer of clinics from the Ministry of local government now they have been transferred to the Ministry of health aa. Where do you think this will take us?

Int: it can solve!

Int2: to the Ministry of health as something that can solve the problem of the shortage.

Int: of the problem of shortage of workers.

Int2: of workers!

P1: I think it could be helpful because um…because the Ministry of health is the one which now remain in health issues and what. What...so obviously they will now know that here and there we have a shortage unlike when it is at another different Ministry

The Ministry of health will assume that this is the situation but now since they will be part of them, they will know that the situation is like this and this. So that they

do not have to assume but knowing that this is where we stand, this is what we need, hence it can improve in a way.

P3: some of us did not know that it was at the Ministry of local government (laughing)

Int2: Really! You did not know….

Int: eee… to have in place Health leadership in rural areas, to establish Health leadership in rural areas.

Int2: the question is actually saying… the establishment of leadership for health in rural areas can it… can it help to solve this problem, since the problem is shortage, now we will like to know if…if the establishment of health leadership in rural areas can help?

P3: mmm…yes!

Int2: in what way can it help?

P3: hee! It will mean that the long distance that could be travelled to rural areas from Gaborone will be reduced since there would be roads. I do not know if I answered right?

Int2: No, you answered, others will add

Int: they will add!

P1: I think it is going to help very much because that's why you see In a school there are a school and a deputy school head, there are HOD'S. so in a way if a child have a problem it means it will go step by step.so I think even the Ministry if that person is one that person knows that they will keep an eye on that clinic, but she goes to say we have a shortage of this and that since they are the service providers…for the clinics and and all that so I think it can help

Int: is there anybody who wants to add? Eee.. To start the school for training Doctors, can it solve the problem of a shortage of health care workers?

Ap: mmm…

Int2: I like it when you speak like this…that. One ..eee..the establishment of a medical school can assist solve the problem in what way ee…

P1: it will greatly help eee..because we do not have Doctors in our country, people have gone to places like Ireland for training now they have gone forever to work there, so I think that why you see, so now it means that they will be trained here so

there is going anywhere when they complete, we will start having Doctors.

Int: is..is…is there anybody who dispute or agree with what he was saying?

All: yes! She spoke the truth

Int: ee! Is there anybody who wants to add? Mmm…I will go to the sixth question. Did the initiatives that we have been discussing work?

P1: so it means that this this this one of medical school is still in progress so… it is open but we have not yet had its Doctors, so I mean that we have not graduated them…they haven't graduated so actually I think it's going to work, so personally on my own I think it is going to work.

Int: do you want to add?

P7: I want to add, she said the truth

Int: ee! I will go to the seventh question. According to you what can make a huge difference in improving health workers situation in clinics? What can make a huge difference in improving health workers situation?

P3: eee…I do not know if I understand, the. The…are the nurses taught discipline really you know at times You can just leave home to the clinic if you are not really sick and knowing that the nurses will give you hustles. Even if there is only one nurse, you just sit there because she is alone. You wonder if they have had counseling for their job. Or where they are they are just taught nursing only, and then are posted so how patients are assisted...

Int: What do the others say?

Int2: What do the others say? You are saying you know what can make a big difference to improve health care workers in clinics. Based on you what can improve this?

P7: to be treated well.

Int2: to be treated well by whom?

Int: to be treated well on what basis?

P7: for us…..

P7: maybe at times you will find that..eee you see. Some of them you will take as young as you are. Maybe I would have left with..

That child will not say she is my parent let me help her because she is not well, maybe they start telling me how dirty I am, like my child has already said that we do not know if they would have been counseled for the job. Or they are just taken and be told to go work…they are not caring for us!

Int2: really!

P5: they do not speak to us with respect we as their parents, they should say madam may you come this more so that, maybe I will say it as it is, but she is trying this and that since I can tell that now clothes are. Start to be uncomfortable that..

Int: ee! I have heard about being mistreated, actually I wanted to know that according to you what can make a big difference to improve the health workers situation in clinics? Since we have been saying that there is a shortage.

All: Yes INT!

Int2: now what can be done to reduce this challenge? I mean these ones of having disrespect….

P4: they will have to be increased then! Be increased.

P3: Again they should try pay for overtime since in clinics they knock off at 16:30, the moment it is the will tell you what other clinics are still open and you wonder what that all about!

Int: yeah! It's just to increase, since..

All: ee!
P6 The first thing…our children should be increased. Their salaries should be increased really. It's like laziness is caused by the money issue. These children really have a problem because they are not paid well, the reason it seems like they are lazy it's because they did not tell you how they are struggling, they have nothing, I have also seen it for myself.

P4:I am happy that they have been trained for their job; maybe i just worked because I was not taught how to clean, and I learned it here. Now the one who is from school is paid money that is not satisfactory to them, the reason why there is a lot of laziness is because of money.

Int: aa! Can I move on? The establishment of health workers teams is seen as a way of improving the workers situation in clinics in Botswana. What do you understand by health workers teams?

P2: groups as…

Int: by groups I mean as sought of a team.

P4: team…teams…

Int2: it's just that building of health workers teams is considered as one of ways of improving health workers situation in clinics in Botswana eee…The Ministry thinks that if we think like you have been saying, there is a Nurse, there is a Doctor, there is s family welfare educator, and there are those children that you were speaking of that were being trained that you do not see. As a team like that government thinks that if maybe we build these teams of health workers. Actually they form health teams, maybe it would be better. Eee…the health workers situation in clinics, now the question is that aa…. How do you understand? What do you understand about the health teams that if it were to be established aa! You do not understand anything about them or do you know something about them?
Even as I am showing that there is no nurse, but we have a doctor at a clinic that is in a rural area, then we will have family welfare educator….some amongst you think that it will be better if we have health workers teams aa! Can they have some importance?

All: mmm… they can have some importance

Int2: if there is some importance explain it in detail?

P2: it will have its relevance because there would be a doctor a nurse… not where at the clinic you will then be told that there is no doctor you will come tomorrow, so we expect that people will be there full time.

P1: I think it is going to be …because.. team work team work it's always um… the best way to go because …I mean because even in a football match when you come with 9 men, you cannot get the goals. So as a team you should always be a team…all in one sprit, your thoughts together more so that you can help which means when you are in a team, when one get discouraged some you encourage them that that's not how you do things, so as a team you can do it.

Int: mm… do you agree with them

All: yes!

Int: is there anything that you want to add? Who should be in these teams…the one for health workers?

P2: Doctor, nurse, mm… what else? Social worker, yeah! I mean socila workers…

P1: and even others who are on their way that know the feeling of how you being

treated at the clinics and that all that I think when they are given advice. That people are always saying now the time is up I have to go home, or its Saturday the dispensary is not opened! I think if there is someone who knows that people often say this, I think they can involve even the people from the community the end users, people who are stake holders, in this case, they are helped I this case I think they should also be involved

Int: eee…

Int3: what do the others say, it's like only one person is speaking? What do the others say?

Ap: we agree with her!

Int2: don't be afraid to speak…

Int: just speak openly!

Int2: ee! Move on Dr N

Int: ee! What are the different participants in the teams supposed to be doing? What do the participants in these different teams supposed to do?

P3: I think they should have sprit of working together…

Int3: at times we can consider that she was saying eee….there should be a doctor, the doctor….

Int: according to him what are they supposed to do?

Int3: it's because it is one of them in the team…If some are doctors what will they be doing in the team what will the doctor be doing in such a team? If it is a nurse what will their job be? focusing on what?

P1: I think everyone should look at their job that she is doing so that they reach a certain goal. You will know that your goal is to at the end of the day they will have helped patients. Patients so when a nurse is there she should do their job perfectly, the doctor also doing there's, so everyone to theirs, working as a team, knowing that at the end of the day the goal that you have collectively set you achieve

Int: do you want to say something? Do you agree with P1 or did you want to add!

Int3: who are the others that are supposed to be in these teams? Who are the others? Are they the only ones you can think of?

P2: there are there…these ones what are they called….the ones that bathe patients. The ones that bathe patients and ….

P5: even in clinics there are people who volunteered to give Tb patients medication, I think as they are there they are also helping..

Int2: volunteers

Int: team. Who should lead the team?

P1: I think the team itself will know that no..But this person like earlier on a question was asked here in the clinic. I think amongst themselves they should try to see that really if a doctor is the one who will be leading, or someone else. I think one should depend on them.

P3: even the leadership that was already in place, including Matrons, even in our hospitals you will find that each one has a Matron. It helps mores o that you as a patients you feel free to tell them about what you are not happy about.

Int: aa… is there anybody who wants to add something…

Int2: what can the others add? Without considering what you are doing? Ee…. Because they can say according to them who should not lead the team, considering the job they do, that a doctor…someone can lead us for this reason and this reason.

P7: Doctor!

Int: why?

P7: because he is the one who knows the job well

Int2: what do the others say? Because we all have our own opinions….

P6: I also think the Matron is also alright.

Int: you mean I can n move on?

All: mmm….

P3: go on!

Int: how can health workers teams and its relevance in improving the health situation? How can that be achieved?

P5: what?

Int: how can health workers teams improve the health situation through the reaching their mandates?

P5: ehe!

Int: it means..

P2: maybe if there can be eee..What do they call it? Some questionnaires so that when they finish attending you, you fill it and leave it there..

All: sugge… sugge… ee suggestion box ee! Suggestion box

P4: there are there, even these days, I do not know if they stopped.

Int: is it the only one, or are there other means of trying to improve?

P1: according to me, if there is is a complaint you should go there straight knowing that you will find someone whom you can tell about your grievances, because we do not know if the suggestions that we write every day in the suggestion boxes…But if you complete two weeks without going to the clinic, when you come back you will find the very same situation, we do not know if they take those papers and throw them in to the dustbin, or if there is someone who attends to them? But in a case where you find there is placement you will know that for sure I have spoken to someone or you will just be speaking to someone.

Int: the way it is ..the way it is ..There should be someone that ….so that when you have complaints they are the person to talk to.

P1: the one who will immediately go to that team and inform them them that this and that complaint have been forwarded to them.

Int: aa! Do you agree with P1….yes! I will go to the ninth question. What are your views about building health workers teams as a way of improving human resource in clinics?

P6: any views?

Int: what are your views about building health teams as a way of improving human resource in clinics? As we speak of such teams.

Int2: we have..We have been talking about the teams, now we wish to know how you feel about building these small teams of health workers as a way of improving human resource in our clinics.

P2: umm… I think it is something that is great because. How can I put it, when they are all there even if there is congestion you know you at least you will be

attended to unlike you find especially in rural areas, you find a nurse running up and down at least if they are all there ooh! When I need assistance from a Doctor I will get it, when I need it from a social worker I will get it there and there.

P1: I think its I think go it's a good thing because just maybe we will go to a clinic and return without stress. You go there as a patient only to get stress now our BP's will go up, we won't be afraid to go to a clinic, we will go there freely knowing well that we will get the help.

All: laughing

Int: we did not hear so you will need to speak up a bit!

P6: I was saying when you disseminate your programs with the districts; they only concentrate on urban areas where the doctors were already located.

Int2: you mean we should disseminate them?

P6: I was saying you should return them!

Int2: Return them?

P6: yes! You should take them to rural areas yes! You post them at the rural areas.

Int2: o… ok! I thought that maybe they….. (laughing)

P6: no not returning them home, they mostly prefer urban areas

Int: they should be sent to the rural areas, they should not be concentrated only here.

P6: yes!

Int: I will continue…

P1: are you not going to finish INT2?

Int2: no! we are about to finish

Int: we are going towards the end!

Int2: you know what! You are giving us useful suggestions because….your suggestions and answers will help us to think more than we have been thinking, as you see us asking questions and probing we want to understand since you heard Setswana can use a single word can mean tow things, you can say let go back to the clinics or they should go back from their countries. Now we are about to finish

so please let’s be patient with us….ee! go on X.

Int: one of the pillars for the health act of 2010-2020, its provisions that are in line with appropriate health practices. Shortage of workers can fail such intentions and provisions.Yes! I will go on to the tenth question. Do you remember an incident that happened to you as a patient or a patient that you took to the Doctor and a health care worker treated you disgracefully, hurtful, discriminative or that belittled? That one I think that…

P1: that incident I never forget about it it was back in the years when I was doing form 3 in 2003, when I went to the clinic that nurse told us that we school going students how we like to come to the clinic to come and play there, you can imagine. You have taken permission at school saying you are going to the clinic, a student ended up dying there while some of us we returned to come with our parents from home. For him he could not back so he ended up dying at the clinic in front of a nurse, you can imagine while a nurse is there, that was very painful, I will never forget that incident, distracted by telling us that we would come to seat in their chairs we should go back to our homes o go get our parents, can you imagine back then maybe we did not have parents there as they were staying in the rural areas, but you know what? Nurse’s mmmmh… it was very painful and they still do that even today. They should be taught that when they go toa clinic they are going to attend to patients , and they also going to meet up with adults, they should be taught as such.

Int: what have happened to the others or someone that you were with more so that something ended up happening to them that you did not like?

Int2: they had raised their hands!

P7: ee….! For me in Lobatse while I was on the line very early in the morning coming from Digawana, but that lady I do not know if she knew the ladies that were in front of me or what! She was assisting people who were behind me over and over again more so that I ended up hitting her with my hospital cards that. That I had taken..i had an accident..

Now I had a copy so that I can give it to her, but now she was skipping me so I ended up hitting her with the papers, and she called the man at the gate to give me my copy, so I said to the man out of heart ache that if he want to die, not that I would have killed him, so I ended up coming to Gaborone with my copy.

I mean you will never know why you can never be treated well, especially when you are sick and they can see how unwell you are, like someone skipping you over and over again like this child….i wonder if she could have died there, I mean we are not treated well. We end up beating them up(laughs)

Int2: you end up taking the law in to your own hands?

P7: yes! We take the law in to our hands as you will have lost patience, she assist this one and that one while you had come earlier than them.

P1: there is no..there is no discipline…

All: at all!

P1: you find the elderly and us having waited in line then you hear someone saying they are going for a tea break…

P7: yes!

P1: you will remain there and you can imagine you are a patient and you are sick…

P7: that what I was saying earlier on, that the team will help so that that person….some lady could not be assisted to give birth as they transported her at the back of the vehicle saying they do not assist In giving birth, you could see that the person was going to die due to not being assisted along the way, even if she does not assist in delivery she could have been with her to assist.

Int: others!

Int2: others! I do not think they are the only ones that went to the hospital?

Int: we do not mean only something that happened to you even someone that you know or do not know…

Int2: or while you were at the hospital just watching….have you concluded?

P7: should I continue?

Int2: yes! P7.

All: laughing!!!

Int: yes! If there are….

P7: again……. Some other time I went to Marina, there was a lady there whom I did not what had happened to her, soldiers came and were all attended to while this lady was there very sick, so I am a short tempered person, I cried I did not even manage to wait for the Doctor and I could see the lady saying nye! Nye! Nye! Now in life I do not want to see a person so ill, I am short tempered so I ended up telling the person who took me to the hospital that I am better now…

Now she was scaring me with nye! Nye! Nye! While the soldiers were given first priority one after the other…but as were, seeing all these things I did not know where to lodge a complaint that someone is elderly and is our parent, now she was worse than me. This one is sick and the soldiers were given priority, I ended up going and not knowing where I could complain on behalf of the unfortunate patient, I did not like it at all, I ended up going to the private Doctor and spending more money there.

P1: I think they usually treat people because they are..like she is saying mostly in clinics if you there putting on a police uniform or for whatever they will say this person is in a hurry to go to their duties hence they are attended to first. What if this person just brought a mere headache while the other person actually brought a very serious illness? Actually this man..actually the very same people like to come late in the morning around 10, finding you having being in line earlier around 6 at the clinic, they get there and say this policeman is passing through to go to work hewe! Hewe! He will then be attended to…

P5: but that one they have written it…

Int2: they have written it?

P5: in there notice boards that public servants, policeman and soldiers do not have to be in line….

P6: mm… we often hear that they are given about 5…counting us 1,2,3,4,5, and it would be the only way they can get in that way.

P6: but when it comes to the elderly person I saw! She was an elderly person!

All: but the elderly do not wait in line!

P6: they do wait in line that why I end up going, asking myself that this elderly lady is sick but…..

P2: it then means the nurse who was on duty did not have manners…

Ap: Yes!

P2: did not have the emotion to see that…

P1: even if its children

P6: I wonder If that lady survived?

P1: you can take a newly born baby to the clinic but I tell you they will never…..even if its evident that the child cannot breath easily, they are going to

leave in the line, but when a person come wearing a uniform they will attend to them immediately while you have been in line since morning, it has happened to me many many times.

P7: again they we are always shouted at in hospitals more so that when I get to a hospital and say my womb hurt iyoo!!! You are lying….(laughs), do you know a womb where have you seen it (laughs) I mean we have a problem, the sun will set if I were to say all the many things that I have to say

Int2: really! AP aa….

Int: yes! Should I continue or is there anybody who wants to talk about an event that has happened to them?

P1: yes! Go on…

Int: eee…we have come to the end of our discussion…

The End!
