# Supplementary material for: Stakeholders’ Perceptions on Shortage of Healthcare Workers in Primary Healthcare in Botswana: Focus Group Discussions
Source: PLoS One. 2015 Aug 18;10(8):e0135846. doi: 10.1371/journal.pone.0135846 (PMC4540466; doi:10.1371/journal.pone.0135846)
Supplement: S11 Text — (PDF) [file pone.0135846.s011.pdf]

HURAPRIM PROJECT

Participant ID:

Date: 02/04/2012

Interviewer: Dr N

Interview Duration: 1:49:08

Audio File Name: Focus group Health Workers one(1)

INT:We have agreed that we would keep our phones in silent mode so that they do not distract us. There is no right or wrong answer, its qualitative research so you say your thoughts and feelings regarding topics we will discuss. So what is your understanding of primary health care? We said our study is about shortage of human resources for primary health care so I think it is important that we understand health primary care so that we addressing the things we think we are addressing.so anybody can…anybody..(background noise)

P1: yaa...i can answer that

INT:yes

P1: primary is a place where you can provide first care of a patient, if you have a patient with this complication the patient can be attended the first and to be provided the first care for the patient.

INT:mmhh

P1: actually it's where we are receiving people in the rural areas

INT: in the?

P1:rural arears

INT:mmhh

P1: they are coming with all the problems but when you receive them you know how to attend them and to give them the first care and you are going to identify if the problem can be solved in the primary health care or you can even refer the patient to higher facility but it's the first place you can provide first care for the patient.

INT:ok.thank you.ee

P2: to add on what he has just said, the main focus of the primary health care is to host the preventive part of disease.

Int: mmhh…does anybody want to add something?ok lets move on.studies in Botswana have shown that there is a shortage of health care workers in primary health care..kgm.. (clears throat) morespecial in rural arrears..kgm..kgm.. (clears throat) do you think there is enough or kgm..(clears throat) not enough health care workers.I mean according to you.so fi you prefer to speak in Setswana although we prefer to be in English,if you feel you will talk better in Setswana that is also welcome. i think its very important for everybody to participate. Just based on your experiences since you work in primary health care and all that.

P3:They are the primary health care

Int: Do you think there is a shortage of healthcare workers,or are they enough or how is it ?...yes

P4: definitely there is a shortage of health care workers, beginning with the same one who guards the building, cleaners ,even health care workers, all in all they are not enough.

Int:yes P4.so P4 believes that they are not enough.

P4:yes INT!

Inter: of all level of workers

P3:Yes INT…!

Int: from security to the cleaners to……..what do others say?

P1: Yes,INT it is not enough, like P4 was saying it start from the bottom to the top, which means if you find patients take long time waiting for one person who helps because of the shortage of workers.

Int: ok… long ques P1 says reflect the shortage.

P2:there is a shortage because it is not allowed to….its not like when people are there,its not like they get all the services they need,some only get their services once a month, some once a week, which really shows there is a shortage.

Int:ok.. another person….yes P3!

P3: at times is when the workers have gone to their offices, you will see there is shortage since the health users are not getting the services they seek since they are told that a person who is responsible for something is not there.

Int:so ok all of you show that,,I mean your answers show that there is a problem. Now I was asking if there are articulars numbers trade? Like you had already explained, basically we are asking why?why is there a big shortage?is it because we are not training enough?is it because…what are the reasons?.......yes P4!

P4: you are not training enough,because you find that skill skill like midwives and you find that there are a lot of health care facilities which realy need midwifes but you will find a non midwife there the reason being there are shortage of midwives.and for other services like phycatrist like althalmic those people visit private once a month and they will visit health facilities once a week like health posts

Int: ok…..kgm..(clears throat)

P4: Even if there is a shortage for training, they end up going for greener pastures

Int:ok so there is an issue of retension as well

P4:mmm

Int: yaa..yes you can say it and then..

P1: to add to that question,the Ministry they are lying on the old organtry doctors for example they train before the beginning of the primary hospital started

Int: mm

P1: they will just say we need 3 doctors since they believed but there are a lot of program that came,…..clinic, circumsition but even now the Ministry is lying on the orgumtry doctors

Int: mmm

P1:and they still left to increase number of workers because because other program came they still using the old workers.

P2:yes I was people are not employed, yet a lot of people have retired and they have left vacant posts that are not being filled, which contribute to the shortage in that hospital or in that district. You find that there is only one midwife,like P4 had said, a midwife who works day and bight since she is the only one hence not having time to rest. You find that an FW is one,and when she has to go for leave, who will assist you, that the shortage of health care workers.

INT:yes P1…you raised your hand?

P1:yes INT, actually when it comes to health workers, there are certain cadres that are absolutely hit by shortage, which result in certain workers being made to do jobs that are not their,really in health workers there is a shortage that…

INTmhh:

P1: so it would mean that those that are ear marked fall in every category, they are the ones that government is eager to train. Now if I consider the nursing situation you will realize that government is eager to train nurses, yet they do not train more Doctors. They train in few numbers , pharmacist are trained in fewer numbers, in ahurry to train nurses because when they are trained they will do all the duties more so that they will wonder what they really are? And those that were smaller in number will end up conforming to certain culture. So all these careers should be considered and see if all is going accordingly, nurses should do their jobs, they should do their jobs and stop doing other people's jobs.

Int: mmhh..so quite a number of them has come out so keep on summarizing so that everybody will understand because we are speaking two languages,basically we said,,the one we agreed on is shortage,two we think its because inadequate numbers are trained but also not just inadequate numbers but also inadequate..especially certain cardas are not enough trained overall everybody is short but there are those who are shorter than others,and then also we have increased services but do not increase workers and there is poor retension I understand also,so I think what they are trying to capture.is there anything else?

P3:mm like P1 have just said , after nurses are trained,you find that its like government has deserted us matrons, let put it straight like that. When we go in to the health post issue, a village like Dikoma,you find that you are the matron as well as a nurse,and there will be one GDA. The thing is when the nurse is not around you will have too come up with a plan of what to do, but when you get there you find that it is not your job,but if it is not your job there usually end in conflict.

Int:mm

P3: when you get to work, your expectation is that when the nurse is not there when a patient arrives, maybe when a child has high fever,you as a matron you then take a decision ot assist. Then thhe government will say you were not suppose to do that and you end up not knowing what to do.

Int:mm

P3:I have a sick person in front of me, I have an idea of what to do when a child has a fever, since you give the child paracetamol, and I often wonder what will happen when things do not go accordingly you wonder what you will do.

Int:mmm

P3: this really disadvantage us,why don't they all knock off.

Int: mm

P3: Yes INT

Int:so that is another point that an adequate number have difficult working conditions because if the nurse is away and you are a health assistant you sometimes don't know how far you can go on your are on your own because you can put yourself unto a lot of the risk so that's…ok.anything else ?

P1: I was checking to see whether you had noted P3's point that there are replacement made.

Int: yes replacemrnt and also poor recruitment

P1:mm

INT:it will also mean we are not recruiting too.what about deployment or distinguishing are they realy distributed in these primary hospitals?primary care institutions are we.. we are talking abpout shortage..is it right one P2? The one concerning shortage. The thing is that we are not enough because we are not enough or maybe we are enough but only distributed to certain arears,does distribution play a role in shortages.

P2: that one is true because and I can give an example Sefare is a primary hospital and Masunga is a primary hospital but so far we are 3 doctors Masunga there are 6.what makes the difference of them to have 6 doctors and us have 3 it means the distribution was not done well and we don't know what criteria they are using because all they are primary care

Int: ok so anybody else to comment on distribution? Or you are not sure?Pilikwe is not better than Pala Road you are just the same?haha..(laughing)

P3: the problem is we are distributing with what is not there.

INT:mmhh

P3: you also have been mentioning that there is no stuff, again we do not have an idea of how many nurses a clinic with a martenity ward should have, maybe based on ratio like the P2 said that wrong factors are taken in to consideration, for example some of the needs were based on when the hospital was opening long time back and we stick to it.

Int:ok

P3:even this distribution of theirs means that now we have been thinking that it is based on the level of need, for example, we have a village called Pilikwe, we also have a village called Maape, which is run by midwifes from Pilikwe. You find that the midwifes will attend to 16 pregnant women in Maape in a day, which means Maape is visited regularly hence it should have its own midwife based on a need to visit there frequently and large number of pregnant women..

INT:Ok

P2:yes…to add on to that one, really we do not know how it is done based on what, cause right now if you were to go to..post,there is only one person who issue ARV's, so if she is not there then there is an evident problem. I have included places like Kudumatse, during some days when you come they have taken leave because they have to take it meaning then you stay without being attended to. kgm..(clears throat)

P4:mm..well if you take the district that you live in and then compare it with other districts. You will find that there are differences, its like we are rulled by differents governments.

INT:mm…(listening)

P4:like if you consider north east, Masunga I think is in the north east

INT:mm…(listening)

P4:you will find that in all the clinics there are midwifes, that's what they tell us, then take aplace like Mahalapye,you will find that there is a shortage of midwives, then you wonder how others manage and others fail, which only mean there is unfairness somewhere.

INT:mm.ok

P1:actually we hope that when we are merged,moving from local government to central government we thought the distribution will be better.

INT:mhh

P1:but I do not know if it moved from worst to worst ,I do not know how to put it…haha(all laughing) because its something else because if you take the busy facilities or clinics, you find there is a shortage of stuff, but when we talk of a pharmacy technitian, I mean they could try improve the services offered in the clinic because the situation is still the same.

INT:ok…yes P3!

P3:I also wonder about the distribution because when I got to Machaneng, there were 6 nurses,meaning 5 then remain. If the others take transfer they do not replace them and that's lead to the shortage of staff.

INT:ok..so those who leave are not replaced?

P3:yes INT.

INT:ok lets move on.do you think there are gaps of problems or issues regarding health care workers for primary health or not?if there are gaps or problem,what do you think are more important gaps, issues and problems?

P1: gatweng?

INT: are they adequately trained for the job?i think we have covered a lot of that one like what actually P3 as saying in the first part we just want to focus a little bit gore o bua ka di gaps.what are the important gaps,some of the things tou have mentioned but do you think people are adequately trained for jobs that they do?and what were the other things?basically we are exploring the different gaps,I think we have covered some of them but we just think we would ask if people are adequately trained for their jobs are they assigned the jobs that they are adequately assigned for and is there a shortage of caders,I know you have mentioned some already and what is the other thing?is there adequate support in terms to do the job,in terms of resources,in terms of organization,or coordination, management or is there..how about condition of service?so these are some of the examples we putting forward but we want you to explore broadly the issues that could be contributing to the shortage.

P1:yes INT, when we talk of having trained you e=will end up doing your job it's a wrong perception, because we are people and have other nurses who trained as psych. Yes they can do some things in their facilities, and its not adequate that they should be at such places because it means then they

do not get to practice because the conditions the conditions that they can treat are mostly in psychiatric clinics. You end up not having such cases, because you mostly work with cases that are cool that have already being attended in hospital.

P2: eh..support.. when you talk about resources,there is none, cause now we are talking about transport,because there are no cars at all, it's a big cause for concern,when you get to a clinic you will find that there are no resources, equipments we do not hav,but maybe two clinics ,maybe I can say Mahalapye sub district they may survive we talk of processor in those clinics but when you get to other clinics tere is absolutely nothing…(pause) which is a big problem.

INT: when you say there is nothing what sort of things are we talking about cars or drugs or what source of things

P2: let me talk about transport, we don't know what to do, even though when we came from local government there was also a lack of transport, here the situation is much worse, you will notice that more clinics in Mahalapye do not have vehicles, which results in patients suffering, not knowing what to do when there is no medication, medication that we get from the hospital.yes INT.

INT:ok.

P3: the one that you said whether people did the right duties, no, but you can find matron or a cleaner end up being forced by circumstances to issue medication, even though they have never read or learned about pills.

INT:mm

P3:so circumstances end up issuing pills because the nurse is not there and people have been waiting, on the other hand you are consulting, you dress wounds since there is a shortage, actually there is no one who is doing a job that they have gone to school for.

INT:ok… is there anybody who want to add something? Ok…so basically we are saying there are lots of gaps?we are doing things that we are not adequately trained for even when we are properly trained we do things that we shouldn't be assigned to.i think maybe we can..Unless there is somebody to add something.

P1:can I say something on gaps?

INT:mm

P1:the other thing is the management or the head of the …to address and to know where to address the problem because you can have the shortage even if you can identify there is shortage here you do not have power its your management to address the problem to whom it may concern the problem will remain like for example we have been having 6 pharmacist 3 left and now we have only 3 left

INT:mm

P1:and they close, we are not dispersing drugs during the week

end because they are shortage and this is the problem, management need to solve the problem not to close the service but if they are not addressing the problem when the patient comes during the week and they are on call and they are not dispersing there are 2 choice you have to let the patient go without drugs or to admit the patient because generally we can provide but its difficult the management that is suppose to..the head or dsmt, chief medical, the clinic they have to address the problem as soon as possible.

INT:ok

P1: again you find that nurses a lot of the times they are trained on a lot of things yet they have their scope of work, its just that a lot of the times a lot of things are spread like the Mahalapye east area, their clinics are 12, the pharmacy technician is 1, even if that is the case, nurses are then trained to be part of pharmacy technicians, they will be trained even though they have their own job description. Then they will say a Doctor who attends to ARV patients…..the Nurse will then be trained for prescribing ARV's, the thing a lot of responsibility is given to one person , then they will not be able to provide the quality but to stick to quantity instead, that do not also work wel for us.

INT:ok..so lets move on to the next one.Is the a problem of health care workers in rural arears?if yes why do you think it is so?do living conditions in rural arears contribute?akere we are now talking about primary care which is practiced everywhere?

P1:mm

INT: even in door steps of princes Marina or extension 2 clinic that is primary care provided but now we are talking about rural areas gore is that a problem in rural areas and why in rural areas particularly?

P1: the problem with what?

INT: we are talking about human resources, what he is trying to say is, is the problem worse in rural areas of human resource and why is it like that in rural areas?

P3:yes INT..their is a lot of shortage. O think the problem might be that when you are at Gaborone and you are requested to go to rural area you may end up giving lesser effort because the transfer is not easy on you to be taken from a remote area to Gaborone, you see how it is?

INT:what do you mean?

P3:I am saying that because transfers are not easy from remote areas to urban areas.

INT:mmhh…(listening)

P4: and they are still not easy for the one in urban area to a remote area they refuse whereas, while maybe I will be in a rural area and I want to go to an urban area, again you find that people who refuse to come to a rural area are doing justice to themselves because we are lost and hopeless.take Mahalapye as it is, workers are wondering about without accommodation at all. You find one government employee paying P300 accomodation, while I pay P1800 at a village,you see how painful it is. So if you are in Gaborone maybe you will run this and that and maybe end up with you own house. Live in Gaborone is a bit easier and better. Now if you are from Tshabong and look for a

house in Mahalapye, while will you want to buy it because in Gaborone its where there is a better life.

INT:mhh. so opportunities are better in town?

P4: in town the living is better than in remote areas.

P1: ya the living condition can also contribute to that shortage in rural area because. City people from Gaborone don't like to come to rural areas. I remember like Moa…they don't have electricity and they wanted to transfer a nurse there all the nurses refused but when this thing of dismiss came they used a nurse from there and didn't warn her and she went there you see that is the living condition.

INT:and the problems in the rural arears

P3:Again you find that in rural areas you feel rejected, even when developments come in the village , you are going to sweat so that the clinic has electricity,but while there is electricity in the village. Its just that we are not given priority that here is a clinic that must have electricity, you will keep on lighting your torches until.

INT: what about the cost of living in rural areas?

P3:like you have heard us saying it is expensive because our children are paying rent of P1800. It is so hurtful that if I am offered transfer I will refuse instead opt to resign, because I can not go to a village and pay P1800. I would rather prefer that …we are feeling for those paying P1800, at least if you were in rural areas you be given some incentives such as houses, I mean you staying in your house with electricity . you children given an education of good standards. Still if you are still in the rural area you share a house otherwise you go and rent a more expensive house.

P2:Really the rural area are not alike. A village north of Shoshong is not a bog village but if Matrons would consider the situation they would not place youth who are still young because there are not much challenges there so instead people like Nurses, meaning the elderly ones. I mean since a grasuate can be from the 1st to the 30th of a month without meeting challenging situations. And you wonder what if someone like this is to be placed there for 5 or 8 years, is ther going to be any productivity, why not an elder person there?

INT:Challenge?it means they are no challenges they are disliking.yes P4..

P4: again if you are in a rural area you are placed there for a long time , even if there is someone willing to swop citing that there is no money for transfer. They do not consider your living condition. At least they should rotate us every three months in clinics , considering that nothing is spent in a transfer so that others can gain exposure . when you are there you will be there for 7 years until you ccamp at the matron's office asking what the development in your request. Its like you are ideal for the place while those in the cities are left there, staying in the community for a long time is not good.

INT:ok. Is there anybody who wants to add? as we are working in rural areas is there an opportunity for you to further your studies?to further your education when you are in rural areas is there any opportunity? For you to go for further studies does government take for further studies?

P1: I think its depends on where you are,maybe you are where there is no electricity, there is nothing there ,you can not do anything, but if you are at a rural area where there is electricity and better roads its much better, like Pilikwe. There is noexcuse of not being able to further your studies,since you are closer to Palapye. I can access things there. But if you are at Kodibeleng where there is nothing, you cannot do anything do much,but the work load is normally easy.

P2:To add on, I want to differ with her since some places are far, when we look at Bocodol and you wish to correspond,yet you are staying in far away places with no services will make it more difficult to. You end up wishing but failing to.

INT: how about education opportunities for your children, do you think? we are trying to extend our thinking, what are the issues that people don't want to come..i think we should establish gore people don't want to come but maybe opportunities can differ or maybe they can be available in some places but not others. is that a problem, do you think people don't come because their children will get…

P2: yes, some people are not coming because of the school for the kids because someone here transferred to Francistown because they wanted their kids to study at English school and the other doctor left because he had an opportunity to study at ub and he said he cant stay this far and he left the post is still vacant until today so its not easy and people prefer to leave to where they can have opportunity to study.

INT: Is there anybody who want to comment?what about availability of jobs?for you partners ,really this is about exploring what are the other issues because we just want to see if they would be a problem that you people feel my partner, my husband, my wife cannot find a job here so I wont come or something else. possible contributing factors.

P1: At the rural areas you will only have a man who is a teacher. Hahah..(laughing) because only nurses are the only people at the rural areas, actually people can request for transfer because she is married to an engineer in Gaborone, imagine you living there you will suffer because of separation from your spouse which is not nice.

INT:what do you think should be done about lack of health care workers in Botswana? Training, should we train more, quantity or quality?

P4:when we do training you are mostly looking at quality, like P1 said there are so many blames with regards to nurses, when I went to the clinic, they did not take care of me because I spent the whole day there, there was only one nurse who prescribes,who dispensate, so all these. Only one nurse? There has to be quality!

INT: so how would that training come?

P3:train more,pharmacy technitian,even Doctors, train matrons. They should train them.if they don't retain the Matronsbo they should train them.i think the percentage we are having regarding Matrons is less than half the percentage when you talk Mahapye by its self and it's a issue of the nurse and a nurse is not expected to complain. So lets do quality not quantity.

INT: mmhh..training others?ok

P1:i.. I would say the vacant posts should be filled. On certain dates there are certain things that you find that have been given regarding needs, I think it appears when you have submitted that here we do not have a night watch man or there is no GDA, furthermore when someone retires the government knows that, its now vacant when they written you about prioritization, yet every person will suffer like if we take exampleof Tumasera/Seleka clinic there is only one GDA and imagine there is a structure for ARV's there, there is a clinic there that she is expected to clean. How do you expect someone to be productive in that state ? if only a person was replaced when they retire it would be better.

INT: what else can be done ?deployment or distribution.what should be done to distribute or how should they be distributed?we talked about all these issues like distribution is not equitable so basically what should be done about it?how do we know how many we should send and to where?

P1: I think statistics for the facility should be looked at, maybe like when overseeir fro the martenity ward maybe like at Shoshong, can submit that in a month we get this many we attend to this many people, and we will need what or ARV, so maybe like Shoshong has more than 900 pills things like this are being considered.

INT:ok so distribution should be based on evidence? Anyone to add?so what about retension strategies?remember we said some people leave to early so how can we try and retain them?

P2:I think we should consider living conditions like provide electricity because in Pilikwe, machaneng we don't have shops, atms all these people have to travel 90km after money to look for money and food so if they can improve the living condition there it can be better because some which are from Gaborone find it hard to adapt and they ask for transfer to go back.

INT: so basically they need improvement in living conditions anything else that maybe the Ministry of health could do?

P2:the other thing is there are no houses and people go because of that.

INT: any other strategies?

P3:even the one of staying for a long time in a rural area should be considered, at if you have stayed long in a rural area, at least 3 years, as a standard not only for Mahalapye ,it should be for the Ministry so that a person can ask why they are still there for that period it should be standard so that the other person also hears what you heard.

P1: the ministry needs to make a policy that binds health workers so that those who are in the city also feel what we are feeling in rural areas. that will make people to commit to what they are doing.

INT:ok

P1:I think people who live in the cities have a lot of things to do, so people in the rural areas should also be considered. There are incentives they can be given even if you have stayed there for 3 years, so that you feel confident like having a child going to an English medium, they are not just there they get something.

INT:mm

P3: you see places that hosted the desert runs, when you were in such areas you managed to get something moreso that you are also benefiting.

INT: mm..(listening)

P3:Even if one has found a found a BHC house, for you to be able to say at least let me me fuel you car. Its something because you will do the job well because there is something that you are getting and on top of that I will be here for 3 years or such a time.

INT:ok so intensives

P1:mm.again you should have preference to say to say I want to go there,even if it is in the rural area, not to be just thrown there resulting in people swopping wasting money. But if only you were placed where you initially wanted like Palapye, it means I can work at Makoro if I am required to go to a rural area.

INT:ok.i think they should be given preferences.what about being trained for different skills?

P3: different skills in terms of?

INT:ehe..skill mix, we were talking about addressing shortage of human resources like what can be done to retain people in case of skill mix. We were saying ke nurse, next time you prescribe ARV,and then we want them to test eyes so how do we get the right skill mixes that's what we are basically looking at.maybe them all together or take them as task shift. should we be relooking at the skill mixes we have and decide whether the job has to be done by so and so or canit be done by someone else and it become legal for them to do it so that they cant be afraid to do it because of what may happen.do you understand what am saying.we could look at what everybody is doing like if the job is done by a Nurse and they are properly trained and supervised for it they can do it or maybe get someone from the community and train them for 3 months would that help to reduce the problem of shortage of health care workers not only have more people but have quality service.

P3: There would be a lot of difference if everybody was to be told you are placed here, like I gave an example that at times when you are a Matron you end up giving pills, like we have said theses rural areas differ. Some have 3 nurses, others like at Ikongwe you have 1. Now if she is not there you find that at times you use your phone to call other clinics, problem you find that they also do not have a vehicle. They will then instruct you on what to do until assistance comes. If Matron's job was to weigh children it would be much better, it would avoid certain issues since everyone will be doing their job.

P1:nn that one INT I am failing to answer it because as wea re working we do not have a job description, but maybe I am hoping this will lead to us having them, they can guide us on how we work. I don't know, but I think if they are there it is the main guide.

INT:I think description will describe what you should do and they will allocate you according to the skills you have.i assumed people had job descriptions.they are very generic,are there no job descriptions?

P1: they are not there. They are still being developing them, the gist of the matter is that you will find that it becomes anyother, so whats the use of having any other because it will get me in trouble tomorrow. If its okay I get commended for it and vice versa.

INT:mm

P4: I think if It were to be made legal so that it does not become about the nurse only being asked why she had to got to the dispensary? So we have to know that our role as nurses so that other work assigned should be paid for. So that there is no saying you agreed to ARV prescription, but now that you have commited yourself moreso that when the Doctor is there I will just sit and say the doctor is paid for this.that is exactly what is happening because if the doctor is there I go back to my job as a nurse but if we were paid it could be ok.

INT:so basically each extra skill should have an insentive .

P2:I want to ask if really we were to get paid for it and be trained, is there going to be any productivity or value of what was done?

P3:is it not the same if you prescribe or dispensing without getting paid for it. The thing is we are doing it for quality not quantity, so all these people to be trained while the Doctors are there, same as Pharmacy Technicians, whivh means we nurses we will be less on those services because relavant people will be trained based on statistics . because when you consider Sefhare three Doctors are not enough.

INT:ok,so in your experience what are your visions about intervensions that have already being tried to resolve these problems?perfomances in remote systems?its just an example so basically you just have to come up with your own.What has Botswana tried to resolve this?

P3: I don't believe there is anything that has been done because this things happen right before our eyes, like our was talking about FEW to dress wounds and to dispensate because they have not tried to fill the gap in Pharmacy. So like I said its better than nothing in the health post, so even here in Mahalapye if you check the possibility that you find that the disoensary GDA is very high, but not because nurses are not working, but which end up others volunteering their services.

INT:ok,so what else have been tried? Yes P2, did you raise your hand?

P2:extra jobs are alwalys put in for Nurses, so I think they are trying to fill in the gap, the ones that are there, which I don't think its good for Nurses.

INT:ok,so nurses have been trained and given more and more things?

P2:mm

INT:so what else have been tried?How about pms pbrs?was it attempt to increase or an attempt for performance?

P1:pbrs?

INT:yes

P1: for me it brought pressure.

INT:really!

P1:Even if I see that I shouldn't be doing this because its there in app of Mahalapye and I will do it, even at a clinic irrespective. When we speak of drug management, I was taken as a nurse to a workshop on drug management while the pharmacy technician is there, I am doing stock taking because the objective says I should drive which brings more pressure on my side.

INT:ok, no other strategies that are positive and they worked?or they is no other strategy tried except for more…..

P2: This one of nurses being given more work load works well, people do not sleep, but this one of PBRS I see it as waisting stationery. Haha… (laughing), it really consume time meant for patients, at least if you were requested to implement only, instead of sitting down saying I did this and that it could be better. Then the reports pile up after a year, then you feel pity for yourself that with so much work who is going to take the reward, having limited time, since you are also working since you have pledged your commitment..

P3:then again the bprs is not well planned, is well structured, for example, for example in Pilikwe clinic im there with a Nurse both of us are on C1, because the other worked first I was asked to asses heer ,does it make sense , even when the information comes I don't take it seriously since I was assessed by a person like me. It is not well thought in terms of its structure, for instance its not put in to practicesince a register is supposed to be done by chief registered nurse, at times she wont be there!

INT:you ladies wanted to say something?

P4:for me personally I don't see its relevance,its just a waste of time, stationery with money that could have been used to increase people's salaries since there is no relevance, and its outcomes never materalise. There are no increases, there is nothing, so I actually wonder where we are going. Its just a waste of time.

INT:so that strategy does not work?so what about moving primary health care from local gorvrnment to ministry of health did the strategy work?has it helped as a strategy to improve things for primary health care?

P1: maybe it helped maybe it did not, for me I will say on issues of drugs its much better, you ca go freely to Sefhare to ask for medication. But before it was not easy because we were told we are of the Ministry of Health you job is to counsel, but with transport they did because I don't know if they have CTO's standards that are high, because we do not have a vehicle, but they were there at the council. They also take along time when they went to be serviced they are not given priority because they are ambulances. It is treated like every other vehicle. When you were at the council you will call and a replacement will be given as a replacement so that you continue with your duties. If you want to reach areas you cannot.

INT:ok

P3:for us Matrons we should have been left at Local Government because they do not where they can fit us. Even our uniform is an old one, and we enquired from the matron about it and she said they are still considering where they can place us. At times when you are at the rural areas where there is no nurse you find that you are compensated for her work. Right now we are unsure of where we are since they are not sure where to place us. So at local government there was no problem so they could have left us there.

INT:so basically yo are saying may have full support or maybe have not in harmful or other way.

P3:I would think the government should have considered us by districts, we evaluate the district to see how it is because when you get to places like Mahalapye…we see that we are not yet there. We are unsure where to find accommodation because clinics workers are still on the line to be placed which shows we are not yet welcomed, so if we were given a choice I wouldn't be going there. To sum up, there are lots of drugs, like I am saying now there are no cars because they broke down, can you imagine cause we are talking about Mahalapye hospital, we should be saying the hospital vehicle has broke down not clinics!

INT:ok

P4:us cleaners when you try to ask for cleaning equipment at the hospital, they will say they do not use manual but electric equipments, but when we request for them thery are not there, unlike when we were under Council being issued.

INT:ok

P4:everything that we just said, if things have changed then they have changed from bad to worse because us who work at the clinics have gotten nothing, when you request for this they say its not there . like the lady have just said, they will say there is no soap, patients give birth and you have to clean the sheets, now water is being used only because of the absence of theses. But all these were not there at the council, when we wanted toiletries from Pilikwe, they tell us we use machines so we don't need soap. When it comes to cars there are patients who require to be referred while there is no vehicle, but when you call where you ccan find help they decline. We did not know these things at the council..

INT:ok ..talk so that we move on!

P2:this one regarding probation annual report, evaluation is done every week and you find that testing has gone down because people who counsel work with contracts that are often not renewed,like as we speak right some are not at work,they is nobody to test people. It has created problems and I wonder what the Ministry is saying in terms of skils…

INT:what about having district management team?district management team is also setting up a team that manages the whole district in your opinion has that helped?

P2: dhmt?

INT: yes P2!

P2:DHMT is really creating trouble for us, for instance if the supretendent will advise to take you queries to DTNT coordinator,but when you do that you are wrong again, but I don't know if its only the case of Mahalapye only,where DTNT have not consulted with us regarding how we will be working. You end up with hospital manager because we do not what to do.

INT:so its not very clear how it works?

P2:yaa its not clear because they need to fix the problem before they involve others.What is its purpose if they cant solve the problem?

INT:what about introduction of medical school programs?in rural areas and primary health care?

P2:I did not hear the question.

INT: we are talking about intervensions

P2:mm…(listening)

INT:so they introduced medical school programs in rural areas and primary health care sso does it work?

P1: thank you INT, personally I think there is some relevance to it, you find that Doctors are trained and they end up in hospitals and their office luxuries, but when they get to outreach they end up having problems, but for now since we are in clinics with them they at least have an idea of what to expect.

INT: ok,anybody to add? To the ones we have been talking about. What intervension will make the difference to primary health care?if you had ability to make interventions like if they do this human resource will improve.

P3:improvement can be there if Primary care can be returned to the council…haha..(all laughing) issues of stuff were done up to…how do you sing for transfer, how do you make small monies as this days it takes time. If they can return us to Council, if only Botswana health care system had taken number 1 otherwise it would maintain that, but I don't sewe how it can pass anything.

INT:so you think the only thing is to reverse back to the coucil?

P3:yes, alternatively CSS would have to be reconsidered?

INT:what is css?

P3:council secretary. Its high time this position is reconsidered in to Primary health care so that we do not dwell much on hospitals because it does not offend us, not keeping on making reference to clinics as it discriminatory.

Int:ok so you want a structure that prioritises primary health care?

P1:and the dhmt should rectify all the problems because they always call us nothing here in Mahalapye they have to go to the clinics identify the problems and solve them.

P1: again during orientation when we relocated they told us that these posts are temporary its not that they has to be dhmt.so I think now we should be having dhmt that is functioning well.they might be because they know that its temporary and there is no communication between them because they can tell you different stories.

P2:I think if there would be someone who confines themselves to clinics because nurses are hard hit by the transport situation. When they are to transport a patient like us in Machaneng, we request for transport from Sefhare, but it should not leave without their permission, and at times they delay hence when you get to hospital the patients returns without consultation. While at the council nothing is happening.

P1:even at the Minstry of Finance there are people employed to pay health workers in clinics, and then there are those that pay the hospital workers, if they could this with the others it would be better.

INT:there should be some focus in to primary health ?

P1:yes INT.

INT:ok.building of effective primary care health team has been suggested as the potential intervension to promote primary health care in Botswana what is you understanding of effective care taker.akere we are talking about strategies so the other thing that has been proposed is that primary health care teams,care that is given in teams usually it can be one way of making sure that proper care is given so primary care is one of the concept so we want to know your understand about primary health care teams?

P2:I don't understand the team from where,hospital or…?

INT:team from primary health care ,basically what we want is to say if we are told to build effective primary health care teams and those teams can be at primary health care,health post or clinic or hospital but they are for primary health care and provide care as a team.i don't know if you still don't understand.

P2:what was the question saying?

INT:what is your understanding about about primary health care teams?

P2: to my understanding it should be a team consisting of all those different skills according to the needs of a particular setup.

Int:so it should have all the skill mixes that are going to provide care.ok.what do others say?so should they be the same in each primary setting or should the teams be different depending on where they are?

P2:its still fine.we are working as a team and without a team you cannot do it.

INT:but do you think there is a concept in a team ant everybody in a team knows that am realy part of this team akere in a football team the goal keeper knows that others will tackle the ball before it reaches him so who should be part of the team.

P2:who should be in the team?

INT:who should be in the team when we talk of skill mixes who has the skills to be part of the team?is it difficult?

P1:nurses, doctors, ocxillary, pharmacist,lab technitian,cleaners,everybody.because if you are in the team you are going to provide good service.

INT:so if we are going a bit further should this team be the same sa other primary health care?like the team in Pilikwe should it be the same sa the team in astreep clinic?

P1:that will depend on the stuff situation ,but the thing is ,whoever is there is in a team because its all about giving care to the clients.

INT:I still want us to explore it a bit because earlier you said you don't know where you boundaries are and who is playing which part of the team.

P1: yes INT, I was making reference to those at the top. The thing is we do not understand DHMT. BUT then you get into teams at the facilities everybody is participating.

INT:the operation here is effective because what we have said so far is we are not a very effective team by making the goal keeper making the goal scoring. what will make primary health care an effective team?

P2:it has to be organized

INT: who should lead it?

P1: I think we can start at the top, like I said earlier on that the structure should be well balanced starting fromm the top.

INT:am still going to ask again about effective,the team is complete right?so what is going to make your team complete and effective?what am driving at is it cost effective?

P3:t will depend on the level of the facility since health post is not the same size as a clinic.

INT: but everybody should know their job ?

P3:yes INT, we are talking about quality not quantity.

INT:so anybody should be trained for their job and supervised.

P3:yes and supervised because in some clinics if you go to maternity there is a team from doctor to cleaner so all the health post should have that.

INT:ok,how should the quality of the job for the team be evaluated and the impact of its role?this is important because we have to know how the team is doing?

P1:but now we are taking it back to the one that we said we do not want ya PBRM, because the team has to set objectives and they should be evaluated how can we do that if not through pbrs?

INT:does it always have to be pbrs?

P1: it goes right back to pbrs.

INT:so we still going to have that.

P1:or also have a comette or standard operation that evaluate the whole thing and how we can improve

INT:ok.i think this one everybody can answer it,what is your opinion about building primary health team as one of the intervension to improve primary health care?your opinion,can it work?should it be encouraged actively?

P2:I think as the P1 said it can work but things must be defined.

INT: the last question do you remember any problematic situation at your work that you find immorally difficult? this is about ethics.

P1: sometimes you can have a patient that is suppose to operate using scissor but the person who is suppose to do it is on leave to transfer them from sefhare the patient can loose it so you can call another doctor and try to help the patient and everything goes right but what if something went wrong.we have difficult situations but we normally just risk.

INT:any other?

P3:I was hardly 3 years in service and a non mid wife so we were only 3 in the facility all non midwifes.a 35 year old lady came and I took her to Tutume, on the way I was force to assist her give birth, then when we got there the midwife then said what was I doing! I had no choice,but then if that baby would have died I would have been in serious problems, but god helped me by reminding me what I was taught, but it was in a big clinic that should have a midwife.

INT:any other?

P2:A lady with a two year old child while there was no nurse and nothing at all. The child's temperature was so high thst I could not see where the reading was on the thermometer, and I didn't have airtime to call Shoshong clinic, I mean there was nothing I can do, it was around past 4, the child 's mother started crying and my mind got confused. We went to the bus stop and spent a hour there but at Ikongwe only the shop lady has a car in the village. After an hour some gentleman then I had to beg him to take them to hospital where they were attended to and released

INT: We are done. thank you very much ladies and gentlemen.we thought we will be here from 12 to 2 but now am even afraid to look at the time.

THE END!
