# Supplementary material for: Stakeholders’ Perceptions on Shortage of Healthcare Workers in Primary Healthcare in Botswana: Focus Group Discussions
Source: PLoS One. 2015 Aug 18;10(8):e0135846. doi: 10.1371/journal.pone.0135846 (PMC4540466; doi:10.1371/journal.pone.0135846)
Supplement: S12 Text — (PDF) [file pone.0135846.s012.pdf]

Participant ID:USERS OF HEALTH CARE GROUP 1

Date: 03/04/2012

Interviewer Name: Dr N

Interview Duration: 2.03.37 min

Audio File Name: Mahalapye

INT: I am going to ask you questions regarding health workers in your area. There is no right or wrong answer. So please answer the questions in full. Your truthful responses will assist the Ministry of health to understand the situation of health workers in our country.

Research in Botswana shows that there is a shortage of Primary health care (or from the clinics), especially from the rural areas.

According to you, are there enough health workers in Botswana? If they are not enough what could be the cause?

P 01: There is a shortage of health workers because of shortage of money, there is no money that is why there is a shortage because you can take the whole day at the hospital without any help, at times you will be assisted at around 4pm or 3 pm

P2 that is the consensus, there is a shortage of health workers in our country, when they complete school they go to work in other countries.

P3 for me I am saying there is a shortage of health workers, since we are referring to the clinics.

INT: yes P4…

P 04: most of the time I see them by-passing each other as if they are not doing anything, but for us at the clinic we are just sitting here we nobody to assist us, but if you could go to the hospital now there is no way you will walk outside this room without meeting at least 4 nurses.

INT: the nurses?

P 04: yes INT!

P 05: the second thing that causes answers like this it's the reason that have been said that in clinics they start consulting from Monday to Friday, but on Saturday on the weekend there is nobody to assist and be attended, and that it could be one of the reasons why the health situation is not getting better.

P05: I was saying there was a shortage of health workers; the problem is that Doctors cannot visit rural areas once or twice in a week.

INT: so is there anybody who wants to add something? So according to you, are they trained in enough numbers? Are they graduating in enough numbers when they graduate?

P 01: no INT….it's because we are hearing from the Doctors themselves that they are not enough, now we think we train in lesser numbers, if that the case obviously it cannot cover the whole nation.

P 02 it is so low, even the Nurses and Doctors after completing their studies in Botswana they go for greener pastures saying the money is not enough.

INT: is there anybody who wants to add something?

P 01: yes! It is actually true, we have been listening but we do not want to say things without thinking. The truth is that there is a shortage of nurses especially in the rural areas, more so that when you have gone for check-up you will be told they are not available they have been deployed elsewhere, with only one person remaining. It means then they are trained in small numbers that is not enough. Actually there is a shortage of health workers in clinics more so that someone can die there waiting for the nurse who is not there.

INT: you can speak your mind!

P 01: there is a shortage of nurses in the hospitals, you can find that people(patients) will be many outside while there is only one nurse, while the line is long, there are a lot of people there how is she going to manage? At what time? While someone else will end up dying there whilst in the queue

INT: at times do you think they are posted equitably in the rural areas. Is Palla road given the same number of nurses as Machaneng or is there a difference?

P 01: it looks like there is a difference, because when you ask you will hear that Pallaroad is a health post, that one is a clinic, which will mean you cannot have many nurses while you can see there is a shortage. Considering that these days there are many diseases, the same diseases that they say a nurse should go and treat that person. While we are left to wonder what time will she come back, perhaps she had gone to the cattle post.

P 02 when it is said there is a shortage of nurses at times it is caused by them because as they work from 7am, they will get in their rooms without telling you are in a meeting or we are doing something. You will stay there until later in the day and then you will start being agitated, then people will say they are not working, while you do not know what they were not doing. After that people will become many while there are not there.

INT: I was asking if they …. (Baby crying) they are sent equitably at the rural areas.

P 01: I thought they will answer it, they are not sent equitably because you will find that there are people assisting with maternity, like for us we do not have midwives but only nurses, I do not think they are equitably deployed, if they were deployed equitably deployed it would places like Machaneng and Mookane, there are those that assist at Palla road where they are mmm…it's only the nurse.

INT: is there anybody who wants to add something on that? If they are deployed equitably?

P 01: I do not agree because they do not send them equitably because at Machaneng they can have a shortage when someone gives birth, then the nurses will go there to assist, while people will wait in vain there in large numbers, while someone is still giving birth which means that really there is a shortage, so I do not believe they are deployed equitably. Because at Machaneng there is a huge shortage.

INT: yes let's move on! But as they are sent to the rural areas, can they take a long time stationed there? Can they take a long time working at the rural areas?

P 01: yes. Some of them when they are sent to the rural areas they can take a long time there because at times you can find someone has been there for 2 years, maybe the 3rd year, then they can go elsewhere.

INT: the third person?

P 01: the other one can stay there for a year, then on the 2nd year they will leave, unless it is not the ones that are going to school they will take around 6 months then leave.

INT: another person…yes P2!

P 02: me! (laughing) I even forgot what the question was saying!

INT: I was saying as they are sent to the rural areas, can they take a long time working there?

P 02: yes INT…some years back they could take a long time compared to today, they use to work for a long time, and some for a long long time, it's just depend on where the person was because some can take a long time while some took less, it's not like all of them take a long time.

INT: Ok, let's continue…

P 03: yes INT! I have raised my hand!

INT: (Ehee).

P 03: yes they do take a long time, but not that long. The thing is sometime you will hear they are going for a course, and then we will hear they are taking transfer. At times we think it's because of their education, or maybe we are just guessing because we do not know if it is actually happening that they are taking a long time or not because it is something that is still confusing us.

INT: ok P1! Let's move on. Do you think there are gaps, shortages or problems that are concerned with health workers in clinics that they have been adequately being trained? Are they training enough for their job? According to you, do you think there are efficient in the job they are doing?

P 01: I suspect that they do not know it because they would have been taken to school knowing the problems…

INT: I mean do you think they know it? We do not dispute the fact that they are from school. Do you think they know what they are doing as they assist you at the clinics?

P 02: they do know the job, their problem is laziness because at times the nurse who helps can assist you more so that you are satisfied when they want, but sometimes when you come back now the nurse have changed it's like it's a different person, they do not help satisfactorily.

INT: do you think they are doing the job that they have been trained for like those at the clinics; are they doing the job that they have been trained for?

P 01: they are causing problems! Its laziness, laziness in our children, mmmh…the thing is our children when you get in to a clinic…I do not dispute that you should converse with someone, but they will converse with you more so that later they will start writing and writing then tell you to go get medication. How you came about he will never ask you? Not even to ask for details in to your discomfort. They are forever in to their phones; maybe it would be the Doctors enquiring about the day's proceedings.

P 02 it's like we will say the same thing, nurses do know the jobs they have been trained for, when someone wants a job they want it with all their hearts, when they get hired they work with all their hearts. But the moment they see that they are in to the system, even when you come to them under great discomfort, if a phone rings they cannot even say may you excuse them,

They will then talk to the phone while you are there uncomfortable, the nurses are very lazy, they are lazy they do not perform well in their jobs even when you are under discomfort, or maybe when you are experiencing asthmatic episodes when you are waiting outside, they will pass now and then as if you are invincible, they are very lazy. They know the job that they have been

hired for, the problem is that they do not know how to treat people well, the very same people they are serving, that pays their salaries, they do not treat them well and they do not have time for them.

P 03: yes the nurses are very lazy it's true, especially when it comes to the elderly, when they just take a look at you; I tell you they will not have time for you.

P 04: the nurses know there job like it has already been said, the problem is that it's like we are going to say the same thing. The problem with them is laziness, these phones! When you get in there they would just look at you and pick up the phone, they are going to focus on that until they are contempt, then after they finish you will see them writing then you wonder when you consulted with him.

INT: I was asking if they were doing the jobs they were trained for? Like, I mean like a midwife at the clinic if she is only focusing on the job they have been trained for? The one who issues pills will focus mainly on issuing pills only. Are they confined only to what they have been trained on?

P 01: yes, they are doing them because when a woman experiencing labor pains comes they should go to the maternity ward to massage the pregnant woman, at times when they are not busy they can go and assist patients elsewhere if there is nobody issuing pills.

INT: in other words you are saying they are not doing what they were trained for; rather they are doing all the jobs?

P 01: yes INT they are doing all the jobs.

INT: yes! Anybody to add?

P 01: I wanted to say…it was said by that P2 that they do not do their work well, later you see them there you will never understand where they actually are.

P 02: yes it will be as if we are saying the same thing, because in a hospital like this one you will find that there is an eye Doctor, there will be one for injections, one for dressing wounds. But the nurses at the rural areas are doing all these jobs. They will consult with you then go the other side,

it’s the same person who is going to issue medication, same to dress your wounds, same person who collect blood specimens. It now means they are not doing the jobs they were trained for if the midwife will not confine themselves to assisting in delivering babies.

INT: based on you…you wanted to add?

P 01: yes, we do not see it well, you can say if they realize that they are having difficulties, I work with them most of the time because I was assisting with deliveries in the clinic and even in the village. Now the poor people are in trouble because there is no midwife, but when you are there they will have to help you as a woman from the village, because now she is not doing her job if there is nobody who weighs. You will see them shifting there. So we should think of them as volunteers or what is it? But when I am looking at that one, I think we should have not considered clinics because they are not highly valued, All the clinics should be taken seriously and equally, it should be said that this is a clinic and they should hire stuff like a Doctor, there should be midwife for that purpose only. They are always saying there is a shortage. But if there a could be a house for the nurse and the doctor so that they help people, I think maybe the health situation will improve. The nurse is really in a tight corner, because as one take leave, one is left alone. Considering that she will be weighing children, and to dress wounds, and issuing medication. She will then consult and then issue medication by the window. So I think this one can be solved those in management. I think the shortage can be reduced, even though we are always been told that there is a shortage. Like now our views are being taken, so I hope they will find a way to increase the number of doctors and nurses even though the government has a limited budget.

INT: is there anybody who wants to add something? Which department do you think have a greater shortage than the others? Having a greater shortage of health workers more than the other places! So according to you which department seems to have a greater shortage of health workers more than others? The ones that you have been saying like issuing pills, especially the midwifes, it’s like they are the ones with a greater shortage. Which ones can you say are having a greater shortage compared to others?

P 01: for me I think the doctors are the ones with a greater shortage, and then followed by midwifes, because right now at the rural areas can give birth in ambulances after being transported, and there was nobody to attend to them. This is a shortage! But those going to get medication are very rare that they can be without anybody’s assistance. I think there is no problem there. The problem in the rural areas is the Doctors, because as we are working with Ipelegeng they require you to have sick leave in cases where you are sick, so if there is no Doctor to do that it is a problem. Hence it shows that there is a greater shortage, but of course there is also a shortage of nurses. But the midwifes and the Doctors that have been trained for the job, since giving birth is the most important ahead of everything, even if someone is sick; they may die, but they will be in need of that urgent help.

INT: yes P2!

P 02: P1 has said what I was going to say!

INT: you can just repeat there is no problem, according to you where do you think there is a great shortage? Which one can you say it is mostly influenced by the shortage of workers?

P 01: there is a shortage at the dressing room, at the dispensary. You can sit for a long time waiting for the nurse to give you pills.

P 02: let me say it, the thing we are answering considering the area we are coming from.

INT: that one you should answer it considering your area.

P 02: it's the one that I am most concerned with, because we are mostly concerned about midwifes and Doctors, we do not have a midwife, and we do not have a Doctor for consultation. We only have a nurse so we never,

For us to be able to see a Doctor we have to come to Mahalapye, and at times we take a longer time here and it would be mostly painful. At times when you are trying to go back to the nurse, because of these many duties that they have, maybe when you are very sick, and sometimes you will find that even the vehicle is not there and then they will require finding alternative transport for yourself to go to Mahalapye. But then what if you did not manage, but then the Doctor is not there more so that people can end up dying.

P 03: even outside of Machaneng. Even here in Mahalapye you can come to the hospital to see the Doctor for the whole day, and later they will tell you to go somewhere to see a Doctor. But if you do not have the money to go there, and maybe if you are so sick! Really there is a shortage of Doctors.

INT: Do the health workers have access to resources that can make their job more effective? Even the right equipment to do the job?

P 01; I think they do get enough resources because at times when you go to the clinics you will feel pity for them because when you are to get medication they will tell you the ordered medication has not yet arrived.

P 02: I also think think there is a shortage because at times in clinics when you want to go for blood tests they can even not have a tube that they can use for the procedure.

P 03: (laughing) there is a shortage of resources; from the beddings to the medication.

INT: Do you think they get enough support and encouragement from those in management positions to be able to effectively do their job well? Do you think they have enough resources?

P 09: they are not there.

INT: There is no what?

P 01: just sometime ago I had taken my child to the hospital after she was stung by a spider, and then they said there is no medication for that. And it was early in the morning for that matter, mm...i did not have money to go to Mahalapye, and I did not know the after effects of the spider! This shows that there is a shortage of resources; medication sometimes is in a shortage.

P 02 resources at the rural areas are in such a shortage that at times when you go to the clinic you can find that there is no medication. They will then tell you to go Sefare. But now when you get to Sefare you find that there are many people awaiting consultation. But then most of them will be from the neighboring villages, meaning you will spend the rest of the day there without any chance of seeing the Doctor. Hence this shows that there is a shortage of resources in the clinics.

P 03 I personally think that there are enough resources in the clinics. The only issue is the clinic management who are supposed to ensure that the necessary resources are available. I think they are the ones that should make sure that everything is ordered in time.

INT: are you referring to the leadership?

P 01: Yes!

INT: are the work conditions for health workers where they work and remuneration they receive enough?

P 01: Remuneration?

INT: yes, also the work conditions, the conditions they are working on and remuneration.

P 01: with regards to remuneration we cannot say much about it because we do not know how much they get paid. We might end up saying the amount is okay, while it is not enough on them. I think where they work is alright.

INT: Yes P2!

P 02: we do not know how much they are paid, but if we hear their grievances regarding their working over the weekend especially when we are requiring their service. Since they were saying they are not paid overtime for the weekend. Because I had mentioned the issue of weekends earlier on. How much they get paid we do not know, hence we don't know if they are satisfied or they are not satisfied with their salary. Hence if they could be paid for weekend overtime then we will be able to be assisted. If only the weekends will be compulsory in all the clinics.

P 03: we do not know how much they earn, but if we look closely to their work load, it is not equivalent to their payments. Because now most of the Doctors are leaving for greener pastures.

INT: outside of Botswana!

P 01: yes, they are saying in Botswana the money is just too little.

INT: is there anybody who wants to say something? Nobody has not commented on the working conditions? How are they? The very things that determine how you will feel as a worker. It could be a promotion! It could be the way you are supervised! It could be about you getting leave over the holidays, but everything that concerns that concerns health workers.

P 01: it affects the nurses.

INT: all of the health workers, it could be nurses, Doctors, those that dress wounds, pharmacists, all of the health workers.

P 01: we did not hear you well. Could you repeat the question? We do not understand it!

INT: we are talking about health workers, now we want to establish things that contribute to the shortage of health workers? Since we have already said it could that they were not trained in sufficient numbers. then we moved on to say they are not even having sufficient access to resources they need, more so that when a patient come you know that you will give them this medication. Knowing well that certain conditions need immediate attention otherwise the patients can have complications. This could be one of the reasons some people might not want to become doctors mostly due to the shortage of resources, and shortage in health workers.

P 01: well it is getting difficult for us because we had explained that we think people are not trained in adequate numbers to training institutions. But now as the INT is speaking about health workers it's becoming difficult because we know that we have a nurse, but as we have a nurse I do not know who is superior to her? All we know is that the nurse there would be the main person in that clinic that is why we are confused.

INT:Didyouwant to sayanything?

P 02: yes! Do You know whatisconfusing us? I want to saysomething i am not sure if i wouldbetaking us back! I want to talk about Doctors but i dont know if wewillbetakingastepbackwards?

INT: it's okay you can say something!

P 02: the doctors in the hospitals whenever you come with a patient who is uncomfortable they will say they are going to look for the doctor, you will wait there with the patient until later when the nurse tells you the doctor will be coming. But then it will be late. The situation is very disappointing because doctors in our hospitals are not staying in there consultation rooms so that when patients come they can be attended to, but now they have to be called from wherever. More so that by the time the doctor come maybe god would have bestowed his will to take the person's life. Now it is if the doctors are not doing their jobs accordingly. Really doctors are not doing their jobs.

P 03 at times we hear the health workers saying they are expected to do more than their job description yet at the end of the day only the foreigners are paid a lot of money. There was a time when the nurses refused to dispense medication citing that it is not their job rather the doctor should come and do it.

INT: is there a problem of a shortage of health workers in the rural areas? If that's the caser what could be the contributing factors? Does the availability of resources have an impact? Do the living conditions in the rural areas have an impact on the health resources especially in the rural areas?

P 01: Repeat it again we did not hear the question?

INT: I was asking if there is a shortage of health workers in the rural areas. If it's like that what could be the cause? Does the level of resources have an influence? By the level of resources I mean in terms of the standard of living whether it has an influence? Like the unavailability of electricity, tarred roads. Do they have an impact on health workers' shortages in such places?

P 01: there is a shortage of health workers, because there is a shortage of vehicles, even if they can be informed about a patient somewhere far away, there is a shortage of vehicles. Even when a patient is so sick that she has to be brought here.

INT: our main issue is the health workers, now it is evident that there are a lot of health workers in villages near Gaborone and Francistown. The question then say why are there many of health workers in villages near such areas compared to the villages that are far from these places? Why don't they go to Shoshong or Moshopha, I want to be able to establish the factors that will influence them not to want to live in rural areas, it might be due to conditions they are not satisfied

with. Like shortage of electricity and vehicles. For them to come to a meeting like this one they will have to ride a horse for 100 km to also go to a clinic. So could it be one of the reasons health workers refuse to come to rural areas

P 01: yes it could be having an influence on health workers deciding to come and work in the rural areas. At times it’s because of the housing situation, some might be coming from houses that had everything in them, but when they are asked to go to Pallaroad to see how things are, they will then refuse.

INT: yes!

P 02: when we speak of workers we are talking about those in management positions, they are the ones that can ignore going to rural areas they will be eyeing places like Mahalapye, Francistown and Gaborone like the lady have said. In the rural areas at times you can see that the nurses are having it hard. After they complete training they are sent to rural areas where there is not even a house at the clinic for the health worker, more so that if the VDC does see to it that the person gets accommodation, it will be up to that person to make their own arrangements. So now we do not understand what the workers will do, since firstly the availability of clinics is established, then even accommodation for health workers must be confirmed. That is why health workers seem to be abandoning the rural areas; there are problems after the other. We are losing our children because of the shortage of health workers in rural areas, for instance a child can have an infection, but since the vehicle could be doing other duties. Now you will have to hike or hope that the ambulance catches you on the way. These things really do affect us. At least if there was a doctor! At least the doctor will know what to do to preserve the child’s life since he is the most senior person. Of course these things are not only happening in the rural areas only, we sometimes do not say certain things, some people get worse right here in the clinics, because they will say the nurse is not maybe they will be there around 11 am or at 12 pm. Hence many people will return without having a consultation. This is because we are talking about those in management positions that do not care much for rural areas, even though they have been deployed to attend to such areas. So we do not know who is responsible, but we have problems after the other, it’s a difficult situation.

INT: Does the far away distance have an influence on how far the village is from the urban areas?

P 01: yes the distance has an impact, regardless whether it’s a big or a small closer rural area. If it does not have health workers it is the same as the one that is so far. When we speak of Palaroad we are talking close to 35 km, it’s not that far, but a person can die in the ambulance maybe because

it has gone elsewhere or having to collect a patient from the cattle post or something. There are those that cannot manage to come for consultation because they are in poor health, maybe at the cattle post. So this also disadvantage those remaining behind as the nurse have gone to attend at the cattle post under disadvantage as there are those that are also in a critical condition remaining behind. That is why we are saying the distance really does not matter.

INT: far way distance in the sense that health workers might refuse to go work there citing the far distance.

P 01: yes INT. The first as our children come to work in the rural areas they want to work where there are resources such as electricity and good houses. But now the far away distance has an influence because now they will think they have been abandoned and not treated like other nurses. They do not consider that they have been sent there to attend to people, but they think they have been abandoned. For instance those posted to areas in the south region they are always crying foul that they are having it difficult. So the far distances have an influence!

INT: is there anybody who wants to add something?

P 02: Mmmh…. In the rural areas there are few good accommodations, so those that are heading some of these clinics are very lazy because they are not making an effort to build good houses for health workers. At least if they build two houses, then later another two. We could end up having 5 houses that can accommodate health workers. So this thing must be looked at carefully, since the workers like to do their work, and also consider why we have such a shortage in the rural areas. If the workers could work in the rural areas knowing that they are going to what type of area, perhaps the same that they came from. They will not refuse it, now the leadership is the one that is lazy. Now there is electricity in the rural areas but they are not building houses for the workers. I think that way the job will be done smoothly with everyone doing what they have been trained to do. For instance like pharmacist will do only what they have been trained for. While people have since came in the morning and the afternoon will pass by they are still at the hospital in a very long queue. There is a shortage of housing even though there is electricity. So I thinkthey should protest. The management that is leading these workers is so lazy.

INT: yes P2. Anybody who wants to contribute? Does the expensiveness of resources in the rural areas have an influence on whether health workers come to work in the rural areas? Expensiveness of houses and high prices of fuel, and even high prices in shops. Could they have an influence?

P 01: in the rural areas it's not often that you find expensive houses, since you cannot rent a house as if you are in an urban area. Houses are cheap, but expensive at the shops, and also it's very rare to have filling stations in the rural areas. But some are there but I do not think the pricesare…

INT: if a nurse is placed here, you will then decide to increase the rent money.

P 01: yes! Because someone else would consider that the nurse has a lot of money. Yes…

INT: does it have an influence.

P 01: yes it can have an influence!

P 02: at times they rent houses without a toilet and a bathroom. They so not like to go outside at night since people are killed.

INT: yes

P 01: the other issue is food. Sometimes they rent houses without electricity, more so that if they want to buy more food so that they will go shopping every time, the food ends up being bad. They do not want these things.

INT: is there anybody who wants to add something?

P 02: it has an even greater influence, because as the lady was reminding us about the shortage of places such as butcheries. They buy meat products so far, but now tell me how those who are just beginning to work without vehicles! What are they going to do? At times you will rarely come across a house that has no electricity; hence if they decide to buy more meat products there would not be anywhere to store such products. Some will feel that they are totally lost.

P 03: I was saying accommodation also has an impact. For us that live in the rural areas it's sometimes rear to find a house for a nurse that is closer to the clinic where they are working. So

when you have a patient they refuse to go to the clinic because it is far and they could be victims of violence, even the night watchman cannot go that far!

INT: yes…even the watchman cannot go fetch them!

P 01: even men are scared.

P 01: they have also being stopped from doing that.

INT: what about them?

P 01: they have stopped so that they do not leave the place unguarded.

P 02.The only place that is there, so they do not want it to be broken in to.

P 03: it's an issue that is with us also because also there are two houses that we have built, one of the houses we built the other day we were told that we won't be getting the second nurse, we built two houses one for a nurse the other for the driver. They had already told us, so they are saying we can't leave houses unoccupied. The other day the second nurse came but we had already given the house to the teacher, now she is staying in the VDC house, in our VDC there used to be houses closer to the clinics that we used to rent them for the nurses, now the nurse that we are talking about now she is staying there. Now we have been instructed never to wake her up at night because it could be dangerous for her if she goes to the clinic. If she is the only one in that post! At times there is no one who can assistance especially at night. At first we use to wake them up to go the nurse or the driver because the driver is that side. The problem is that you can find yourself sick or someone, you will find yourself running in the village wondering when it would be morning, wondering if they will make it. These are some of the things that have an impact on the workers and also on us the villagers more so that a t times we wonder if the government is really thinking about us.

INT: ehh…is there anybody who wants to add something?

P 01; yes INT, I was saying to work in the rural areas by our children without having a vehicle…but when you want to come and buy groceries in Mahalapye you will wake up early to go to the bus stop for you to be able to buy what you needed, you will get transport at around 2, then you will return back later.

INT: as they are deployed to the rural areas do they have a chance to academically develop themselves?

P01; I do not think they could have it because places for academic development are so far away and most of the time they will be in cities, they knock off late so they cannot say they are from Mahalapye I will also make the return journey.

INT: is the lack of opportunity to further their studies having an influence in their refusal to come and work in the rural areas? For a person to stay here for a week and call it quit and find another job so that they can have the opportunity to further their studies.

P 01: yes because now I will be in Palaroad while other are developing themselves at Mahalapye, some come to learn how to drive, but the daily transport money to come here when I knock off its half four they have knocked off In Mahalapye so I can't go to a driving school, you cannot got to school because it can affect even those who are closer, because if one is off the other will knock off at one because of their shift, and can be able to go to driving school or do other tasks, it's like the one in the rural area.

INT: is there anybody else? Yes P2!

P2; it affect them because at times you will find that nurses want to go for further studies but there is no where they can do that, you have to apply to Gaborone, then there would be a shortage in the rural area.

INT: eehhat times it is the lack of job opportunities for spouses or partners, when you are a nurse and be posted to the rural areas do you want other job opportunities for your spouse can it also have an influence?

P 01: yes. At times if one leaves Mahalapye to Palaroad, when I get there there is nowhere my child can go to school like Crèche or even an English medium.

INT: is there anybody who wants to add something? Most of you agree with it. I mean when you are going to work in a rural area and your husband is an engineer, can it influence your decision to refuse to go there because there is no job opportunity for your husband?

P 01: it can influence! Again it also contributes to the breakdown of homes, because at times you will go work there and they would be a problem. But when you work well but your heart remained behind maybe as you keep phoning and he is not there or somewhere there is no network.

INT: what do you think can be done to improve the shortage of health workers in clinics? What can be done for them to be trained in large numbers to improve the situation?

P 01: if government can train the youth who have academic qualifications in large numbers then deploy them to the rural areas can improve the situation, not only focusing in urban areas, they are only focusing in the urban areas it's like they have forgotten about us. They just give us a small number, after that they just disappear without checking on how the individual is doing.

INT: So they should be trained in large numbers. Yes P1!

P 01: I was saying if we have many of our children in that situation we can call those from this area and bring them here and do the same for other areas so that everyone who wants can work in the area of origin.

INT: it can only be better if the institutions such as HIS could graduate them in greater numbers.

P 01: yes

INT: it can….

P 01: it can become better

INT: then they would refuse to go to the rural areas!

P 01: and to satisfy their wishes

P 02: even rural areas these days are developed they have electricity.

INT: so all the health workers should be deployed from all the areas, so each area should be given a Doctor. Nurse...

P 01: Yes

INT: may you please answer in detail!

P 01: in the rural areas the hospital administration should deploy doctors and nurses in rural areas so that there is no shortage of health workers, so that they are always there at all times.

INT: you wanted to say something.

P 01: the way I see it I think what can improve…we can say them all, but I think what can improve the situation it's for every clinic should be treated equally because for us we are disadvantaged by the fact that they consider the number if people instead of people's health. They think you can only have access to health care only if you are more than a thousand. But if they could refrain from this way of doing things, not to say it is a health post so that everything becomes a clinic.

Let ensure that everywhere there is a hospital everywhere, if at other places there is a clinic; it must be in good condition. Because at places like Mookane we used to transport patients there and you will find that there is no tarred road more so that the vehicle will be travelling slowly on the gravel. This will happen with an expecting mother being transported, but it is deemed relevant that we bring them to Mahalapye, that is why we were saying if only clinics could be

improved and be stuffed with health workers like now as clinics have workers. I think health can improve very much.

INT: in other words you are saying the workers should be placed at the rural areas! So that referrals are not made in some of the rural areas.

P 01: even if they say that or it is like that because there are Doctors who are specialists on certain diseases, rather than having Doctors that are not knowledgeable in certain conditions. I think it can improve!

INT: is there anybody who wants to add? The other issue is what can be done so that they stay a long time in the rural areas? Like the others have said that they leave such areas because they will be searching for greener pastures. What can be done so that they can stay in the rural areas?

P 01: they should consider there remuneration since they are not satisfied with it, it is the one that can be improved. If there remuneration can be improved maybe they can stay there for a longer time or even years.

P 02: even the issue of accommodation isaffecting the nurses and even all of the workers, youcanfindthat a teacherwho has good accommodation herstudentswillperformwellbecausetheywillbeteachingwithaclearmind. And i alsothinkalsothat if a nurse is not satisfiedwhenshecomes to work in the morningshemight not have the right state of mind more sothatshecan end up givingyou the wrongpills, not likesheisdoingit on purposebecauseherheartwillbeelsewhere! I mean accommodation also affects the workers, reallythey are suffering and alsothatthey are not wellpaid and itreally affects them! Theseworkers in the rural areas are reallysuffering more sothateven us as the elderswerealiseitlikeweearliertoldyouthattheycan attend to you, then dispense medication, and afterthatshewill go help elsewhere. Do you know that there salaries are based on there qualification and the job they have been trained for? Maybetheywould have gotten to the point wherethey are volunteering! These are some of the thingsthat affects them, eventhoughthey do not consultwith us regarding the issue. But they do affect thembecause as youhadheardearlier on that one of themwassaid to have gone for leave of absence thatwillbeso long thatwestarted to complainthatwe do not have a nurse. Or aftertheycomplete the leavetheywillratheropt to get a job elsewhere. These are some of the thingsthat are verypainful.

INT: mmm…is there anybody who wants to add something? So you have already said that for them to spend a longtime in the rural areas they should get a salary increase. If they could also be given better houses it can also improve the situation. Is there anybody who wants to add some more points?

P 01: but what is more important is to have love and admiration for the work one was hired for and to care for the people whom you serve. But if you are just working without care it then means someone will just be working to get paid. You will find that it has a huge influence if someone is only considering to be paid only!

INT: we work with that lady, its okay you can continue talking. We are talking about improving the health workers! Do you think there are certain jobs that can be done by other people with lesser education compared to that of health workers?

Comment [k1]:

P 01: I mean there is no one who had even done form 5 who is not trained in the health area can ever go fill some of the gaps! I don't know. But this can be explained by the educated ones, because I think health workers would have been trained to preserve people's lives. But you cannot just come with education and say you will manage that why they are taken for further studies after they have completed form 5, so that they learn how to preserve human life. So I do not know how the others look at it.

INT: yes P1 what did you want to say?

P 01: there are other things like giving food supplements, we never see them!

INT: like we said when we talked of health workers we meant all of them! But does a Doctor have to do everything?

P01: No!

INT: ok! Is there people who are less educated who can help the nurses in the clinics.

P 01: oh! But that can happen because we sometimes see with those that repair houses especially when the family welfare educator is not there, because I think its depend on the level of their education since it will determine if they read what has been prescribed. It can happen; it's just that you were leading to the doctors even though I said the doctors have been trained. It can happen more so that she cannot read the nurse's writing such that she will ask the nurse what she has written.

INT: yes P1!

P 01: yes INT! There are roles that can be done by people that are not so educated, like taking temperature and to also check high-blood pressure, and to dress wounds.

INT: at the clinics are nurses the only ones who supply supplementary feeds? Are they ….

P 01: (group answers; no)

INT: it's not them?

P 01: yes INT! (group answer)

INT: yes we can continue. (Since I am just arriving I would say it is important that all of us should answer so that we get your views). What do you think are some of the initiatives that have already been used in Botswana?

P 01: What initiatives?

INT: initiatives to try and solve the problem of shortage of health workers, remuneration that goes hand in hand with improving work condition, to improve this situation!

P01: with regard to remuneration we cannot be happy!

INT: Another person!

INT: to make the question easier, What have you seen being done to improve this situation? Like moving clinics from the councils, do you think it has improved the situation or nothing has changed?

P 01: We hear them saying it is hard we do not know what the situation is.

INT: according to you do you think there is a change or nothing has changed?

P 01: yes!

INT: so has the health administration tried? Like the establishment of school of medicine! Students have been going abroad to study there, however now they instead attend school locally. So did it improve anything?

P 01: yes it is like that…let just hope it will get better because it is still a new endeavor, since there have not been any graduates.

P 02: no...The problem is that we Batswana we are always complaining, we never except anything even though it is something even though no graduates have completed yet. Even these students are better when they are placed in hospitals because they work more than some of the nurses, you will see then running up and down with patients. In addition they are the same ones that meet you at the entrance to guide you of where to go, and even asking you how you are? While the nurses can never do this, now it’s like like the nurses and the Doctors.

INT: yes they do improve the situation.

P 01: very much (more than three people answered)

Yes they do improve the situation, but the issue we were talking about that they are allowed to knock off at 1630 pm, if I consider the situation at our village, we had a new nurse who just completed whom we could see was good at her job, but like we have been talking about issues such

as faraway places, she left on that basis, even though it was not that far! She complained that she worked till late even though she improved the situation.

INT: is there anybody who wants to add anything? Do the initiatives that I have been talking about of transferring clinics to the central government, and formation of DHMT, did they work?

P 01: the initiatives have worked. It's because I see them working well in terms of helping people. They have really worked.

INT: which initiative did work?

P 01: we are referring to the nurses, those still in school!

INT: so are you saying what made a huge difference is the placement of students in the clinics?

P 01: yes INT. They do work these people they are very energetic, and when a patient comes they are very fast to attend to them. I think even at school they were learning hard.

INT: Anybody to comment on the formation of work related teams for health care workers? As they are seen as a way of improving health care workers situation in Botswana. So what do you understand by the health care workers teams?

P 01: we do not know the ones you are referring to?

INT: in a situation where you find workers in a hospital working as a team; where there is a Doctor, nurse, them working as a team. Please explain it there? (Working as a team) working as a single entity. Do you understand anything about this?

P 01: we still do not understand it!

P 02: it means when there is a Doctor and a nurse who issue medication, since it was said we do not understand the question. Like the gentleman was saying tem working as a team, now we do not understand (INT: working as a single entity) it would be difficult for us because we do not know why they will be forming a team. Of course we can give them a chance to explain. So that we know what the relevance of them forming a team, since we will also enlighten each other. I do here the lady! Whether we think its okay or not to form such teams, right now I will move away from P1’s point of view, because we just know that they work together.

P 03: yes P2…actually the question mmm…they are health workers.

P 04: yes P2! I think they are nurses and they work together! We do not know if they work as a team….

INT: ee.. The question was saying the establishment of health workers teams is seen as a way of improving health care services in clinics in Botswana! We want your impressions on the formation of health care teams (Group: yes INT. If they could be formed) yes, we wanted to know what you understand about workers working in teams?

P 01: personally I think if the workers work as a team you become one entity and do the job diligently to do what you have to do. I think if they can work as a team, they can produce better results in their work and even patients will be happy about it.

INT: yes, anybody to add on to what she was saying? It’s just that she answered it and gave the importance of such teams, like she said it could bring better results. Yes P1! The importance!

P 01: it can do away with jealousy that the other knows the job and others do not know it, which can lead to jealousy, but now if they are working together it is a good thing since they will remind each other, so I think it is okay.

INT: so its importance is that it will reduce misunderstandings amongst them. Yes P1! You will remember someone wanted to say something.

P 01: working as a team will cover me who is slow when doing the job; those who are fast can do the job in a short period of time more so that they can then advise me on best to undertake my job well.

INT: it's one way of encouraging those that are a bit lazy. It then continues to say, who do you think they should be? Who should be in these groups?

P 01: it should be the health workers, nurses and doctors.

INT: you are saying nurses and doctors. Who do you think the others should be?

P 01: I think they work guided by job standards, so I think the Doctors, Nurses, and other health workers should form independent teams. So that they are always guiding each other, so that those that are lazy can be guided out of such a mentality in to a more productive one. I do not know if am right to say they should have independent teams?

INT: yes P1... because we wished that they could be a team consisting of different cadres. So I want us to complete the question on who should be in these teams, like the lady has already said that it should consist of nurses and Doctors. Yes P1!

P 01: family welfare educators! Yes those are the ones.

INT: Do you not wish for other people who come from outside the health system to going these teams of health workers in hospitals?

P 01: all the civil workers like teachers, there are parents, and PTA.

INT: yes. Let me now ask who can be in the team from outside?

P 01: Chiefs, councilors and VDC.

INT: Even the home-based care team. What are some of the roles of those that you want to take part in these teams? Those that will be in those teams; the nurses, Doctors, Chiefs and the others.

P 01: the role they can play is to ensure that there is a difference in output from when the teams were not formed to when they were formed. Their usefulness should reflect on the service to the nation, they should not just form these teams.

INT: anybody to add on what they can do?

P 01: as theyformed these teams like the home-based care people canmeetwiththese teams to discusswhowillbevisitedwhen? By whom? Afterthatthey return to the clinic to give a report. There will go withonly one nurse.

INT: as these teams are formed, whom do you wish to lead such teams? The one that would have been formed by home-based care, councilors, nurses and so on.

P 01: I think it can be led by the Doctors because they have more experience. Let me just say they have a lot of experience without saying anything much.

INT: anybody to add on whom you think should lead these teams if they were there who could lead them?

P 01: they can be led by Doctors because they have more experience.

INT: yes! Do you also agree with the lady there?

P 01: they are also expects, hence they should be the ones leading the team.

INT: mmm... don’t you think they can be led by the chief?

P 01: no! (group answers)

INT: because the Doctor will continue to go to the workshops.

P 01: cant there be number one, number two, and number three, such that it is a Doctor assisted by other people so that when she is not there, the others can then lead the team.

INT: yes. P1 feels that the Doctor should lead the team, and there should also be an assistant, so that when he is not around he knows that the chief will take over. Yes P1...

P 01: well it's like a I am going to differ with the others (INT: there is no problem) as we are talking here that the doctors are lazy, and someone happens to die while still waiting for the doctor, How can a doctor lead the team? We should think in detail about these things so that the very same people we are complaining about are the ones that we would want to lead the teams. I do not know if! (We hear you! another participant) we want these people to work as a team? So this way even the doctor will not object to advices from fellow team members on how to best attend to people? Because we will be working as a team since nobody will want to be left behind. If one was left behind they will want to push themselves since we are working towards improving the health care situation.

INT: like the P1 was saying those that are below you will also have an opportunity to advice management how best they should approach situations, since they will be working as a team. Is it clear P1?

P 01: it is clear! But I want you to listen to me attentively. Since the chief is the head of the village and the doctor will be working in my village. If my chief and doctor are both lazy, it's just that I want to say in such away that it becomes clear. We should look at it that there are lazy chiefs and doctors, why don't we have someone from the ministry of health to come and supervise so that we do not avoid each other, for instance that I as a chief people will see that I am not working well. Hence both parties will have difficult time explaining why the team is not working.

P 02: we were just giving an example we did not mean the chief will come last. Even the person you were talking about the chief can come behind them. It was just an example of how we can structure people, that for instance if you are from homed based care you can be the assistant chairperson, so that the work get done.

P 03: it's not like the chairperson will be a doctor because they elect!

INT: the other thing that I wanted you to understand is that this is a team not a committee. They are not a committee such that they will be so many meetings, but they will every now then have discussions regarding whether people are effectively undertaking their roles, such that the health of the sub district is improving. Dependent on people that are willing to work.(GROUP: with their hands!)

P 01: the group laughs!!!!!!!!!!!!!!

INT: so?

P 01: hold it right there INT! We do understand it, but I am not sure where it confuses us, but as we speak about doctors we know that they will be doing health related work. Like the chief will be representing the village by virtue of being in that team.The same people that are supposed to be receiving health services. Hence that is why there would inclusion of family welfare educators and home-based care as a responsibility they will be sharing. Actually I do not think there is anybody who would be lazy from a group of selected people. Let me give an example, there are public workers teams some of which are headed by the Chief, and vice chairperson will be the head master, and the members will consist of public workers. The chief will know more about the village situations while the headmaster also has a thorough understanding of the school. While on the other hand public workers understand their departmental working situation. So all these things will be done by these teams, so I do not know what the difference will be.

INT: the team should be the same everywhere; it should be the same at the health post in Mahalapye, Pililwe and Shoshong.

P 01: mhh ...nngh no INT I do not have an answer because I do not understand.

INT: I mean that they should be similar teams since we have already said who should be in them (yes P1 they have already agreed) now the people who should be in these teams are Doctors,

Nurses, and family welfare educators, and other that we have mentioned, hence in every clinic you go to should have such teams.

P 01: we do not know how people in other areas will be thinking about it. Maybe for us at Machaneng we would think about including Chiefs and councilors. So we do not how Mokwate would think about it!

INT: I mean that it will be the same for all the public workers in all the clinics.

P 01: we do not know if it would be the same!

INT: we wish it would be like that since we are considering a possibility for the future.

P 01: yes... (group answers)

P 02: it's just that everyone would be doing it how they want it to be!

INT: that is why we are asking you how you think it should be? But if you could be given an example of who these people should be! We are now asking if they should be the same?

P 01: it should be the same (group answers)

P 02: it should be uniform!

INT: you mean if every clinic is going to have all these health workers; nurses, doctors, and midwifes, you mean even a health post should have the same health workers team

P 01: yes. It should be like that even though I did not raise my hand!

P 02: yes…I do not know if it would be possible?

P 03: it is going to be possible

P 04: (let P2? talk) how will it be possible? The reason why I am contributing is because I did not that by the formation of teams was meant to be done at Shoshong or Kalamare, I thought maybe they were going to form these groups at a sub-district level. I guess I did not understand it there.

INT: no it's because we thought that especially in the rural areas there are situations where only one nurse would perform all those tasks we have talked about attending to patients. The reason why we are asking this question is because there are situations where there is only one nurse ,now we were saying if they are a team it would consist of different people with different functions, rather than one person doing all the work. Hence they will have a schedule of doing tasks. We have said that in all the clinics there would be such teams. Like football has a goal keeper who knows he stand there and there would be another person who keep their position. Now we were saying what if we borrow the team mentality from the football teams and instill it in to our health system. More so that you know that you know for instance if you are in Pilikwe, when you have a patient at home, whom from the team will attend to the person.

P 01: Yes! If it's like that then I think there would be a difference. Now since the leader of the group will be the Doctor, it then means there would be a shortage of Doctors. Hence it goes back to

what the lady was saying even though it’s like we are differing with her, I do not know if I also understand the question well, because what we are saying is that there are more clinics out there more than the Doctors. There is a shortage of Doctors even though I do not know there actual number. I know this because there is always a shortage of doctors in the clinics and hospitals.

INT: that is why we are asking if these teams should be the same? If not! How are they supposed to be formed?

P 01: there will never the same, there should be a difference when it comes to management. That is why I am saying if we are going to make Doctors the leaders, then we will have a shortage of doctors in clinics. So it will be up to people to decide whom to bring to this team. Whether it be led by the village chief or by the village councilor? These are people who carry people‘s hopes and aspirations. It’s not like I am politicizing, I was giving an example. If it is this way, now I am beginning to understand it that there can be a difference in Mahalapye. Hence if we are going to have doctors lead these teams it should be in hospitals since if we are going to have them lead at the rural areas we should have one or two doctors visiting numerous villages around, hence they will have to establish who will lead the teams.

INT: so, you have understood it?

P 01: yes.. (group answers)

INT: so what are your thoughts regarding the formation of these health teams? Do you think it can improve the health services in the clinics? So what are your thoughts?

P 01: well I think it will be better if we knew the channels of complaints in the event that you were not treated well by one of the health workers. Because right now when you go to a hospital you will start by asking where the matron is even when she is around. So that when there are errors in judgment they are solved quickly. That all I have to say.

INT: Yes, it can improve.

P 01: yes it can only improve if maybe when you are untreated unfairly by maybe a nurse they will tell her that she is not acting accordingly, otherwise if it is laziness because at times we will be delayed by our phones. They will say do this so that as you answer your phone, your work will not suffer.

P 01: yes it can improve because they had brought a suggestion and grievances box that when I am improperly treated so that the comment can be then be thrown in to the box (Laughing from the group) maybe that can also improve the situation.

P 03: maybe if someone untreated you well they should just apologize because at times people make mistakes, maybe it can be better (someone laughing from the group).

INT: Yes! What do you think about formation of health teams?

P 04: yes they are okay, they can improve the situation, especially encouraging some people who are taking expensive medication and throwing it the drainage systems. While some are being consumed together with alcohol. Perhaps this can encourage them to use medication as it is supposed to be used. The reason why combating diseases is so difficult is because people do not follow instructions accordingly, now such a team will…

INT: you think it can improve?

P 04: well there is not much I can say, except to say there are situations where nurses will come and later disappear while there are still patients, but then if the councilor passes by I can inform him about the nurse more so that he can inform the nurse that it is not fair on patients. Can that improve the situation?

INT: let move on she is saying something. How do you think it can improve ?

P 04: ohhh!! ish (laughing from the group)

P 06: but it is coming to an end!

INT: ok! This is the last one! One of the pillars for policy for health workers for 2010-2022, is to follow guidelines that guide health workers that are accordingly! But a shortage in health workers can hinder such initiatives. Do you remember an instance where you or someone you were taking to hospital and you were mistreated in an unrespectable and inhumane way?

P 04: I do not want it at all! (P4 was crying intensively because she was affected by the question).

INT: you do not want this question? If it remind you of unpleasant thing s do not answer it. If it is doing this to P4 then let's leave P4 and maybe someone else can answer it. Why was it difficult?

P 01: mhh….ladies and gentleman, this issue of having been untreated well by the nurses is very disheartening. If you had ever experienced this inhumane treatment it is because of the total disregard from the nurse, more so that at times you wish you were rather at home at least because the nurses are very heartless. And when you went at night it's a total disaster! It has happened to me.

P 02: they have also treated me badly.

INT: those of you who can manage to talk can do that

P 01: I was once admitted in the hospital while it was still on the other side, the patients there could not even help themselves. They were there just in the bed helpless, even when one called for help! (INT: without no help?) one even fell from her bed she tried to ask for help, but hopelessly, she couldn't even pull her up on to the bed. She ended being there until the morning. Even when a patient wanted water, a very painful situation.

P 02: nurses and times are very heartless because a while back i was feeling very dizzy, and the nurse was about to knock off, so I had decided to wait for her outside her house. When she got there she found me throwing up .she then took me to Marina Hospital. I was then admitted there, after that she decided to return home, but I had an stomach ache, so asked them to help me. But they just looked at me! I then dragged myself moving on all fours. I had to ask the cleaner for directions to the toilet, and when I got back I rested on the bed. Then one of them came asking me who had brought me? And I told her The nurse, and then the nurse said to others if they wanted to lose their jobs because I was brought by the Matron.

INT: Is there anybody to add on to what she was saying?

P 01: they do that so that if you had a child, even if she is not a nurse, but if they have status they will treat you like they treat the nurses.

P 01: I was also admitted in Mahalapye the other day. During the night an old woman who was in a worse state than me was calling for the nurses so that they can take her to the toilet, but they were not there, so since I was feeling much better I decided to take her there hoping the nurses would return her. But they did not come so I had to go back and collect her, but when the nurse came she said who do I think I am because I might get in trouble for my efforts if anything were to happen to the elderly woman. The old woman was very hurt by the nurse's remarks, more so that the following day when her son arrived they told him, but I was afraid to tell him. But I was discharged the following day. But the son was threatening to sue the hospital, so I do not know if he proceeded with it or not. They can only help you if they know someone related to you who is well known.

INT: mhhhit then means we have to the end of our discussion. So I am grateful for your participation, the recordings we made will be kept safely.

T
