# Supplementary material for: Stakeholders’ Perceptions on Shortage of Healthcare Workers in Primary Healthcare in Botswana: Focus Group Discussions
Source: PLoS One. 2015 Aug 18;10(8):e0135846. doi: 10.1371/journal.pone.0135846 (PMC4540466; doi:10.1371/journal.pone.0135846)
Supplement: S13 Text — (PDF) [file pone.0135846.s013.pdf]

A qualitative study of Human Resources for Primary Healthcare (HURAPRIM) in Botswana (Focus Group Discussions)

Date: 2012

Interviewer: Dr S

Interview Duration: 02.16.58

Audio File Name: workers of Health Care 2(Mahalapye)

INT: …eeh good day ladies and gentleman! We will start with our first..uh.ah… our first question…what is your understanding of primary health care?..(repeats the question)…(I forgot, tell them I am coming, the reason why I am also writing ladies and gentleman is because from past experience you will think this thing is working until to try to listen later. It will mean it did not record hence we would have lost everything that was said people. Now as backup I will write (P1) the first question is what do you understand about primary health care in clinics? That is our first question.)

P1: Are you saying hospitals?

INT: Yes P1…yes P1...may you..yes..P2!

P2: ... personally I think primary health is, is a part of the Ministry of health which focuses on teaching people about health, to teach them about ways they can use to stop the transmission of many diseases which are managable, and also to…maybe to treat some of the illnesses. The other thing is to prevent the symptoms of illnesses people already have.

INT:Thank you P2, anybody who wants to add? Yes P1…

P1 : I can say is where health begin, patients are first seen there if there is a problem and refered to the relevant unit.

INT;Thank you P1. Is there anybody who would want to add something, to say something on top of what has already been said?

P3;Yes..i will just say its about preventing and controling of infections of the users at a community based level...that is taking those services to where people are.

INT:.Ok..thank you very much..is there anybody who wants to add anything? Ok thank you. Our second question. Do you think there are enough or not enough health care wokers in Botswana,if there are not enough(clears throat)oh sorry.umh..studies in Botswana has showed that there is a shortage of health care workers in primary care especially in rural areas?so the second question is asking that do you think there are enough or not enough health care workers in Botswana,if there are not enough why do you think so?

P1: Please go over it…

INT: my question goes, according to you are there enough health workers in Botswana, if they are not enough,what could be the contributing factors? It depends on how you answered the are they enough question, if they are not enough what could be contributing to it?

P4: They are not enough..

INT: Yes P4 go ahead..

P3 : I think that, from my own observation, I feel that our government when it comes to health really they have no clue, it is not given first priority, and tat its you see. Like when you compare with other countries , these are the things that result in some slowness, strikes and other things…because you find that a health worker is not entitled to benefits that a health worker can be given, so I think our government does not give us priority, it does not see our value as health care workers.

INT: Thank you,yes P4 there…

P4: they are not enough, now the problem became…what is it called…this relocation of ours, we are still under Council. Maybe stuffing at the hospital;s is better, its like we don't know if its only Mahalapye, districts we don't….is just that a Nurse at the hospital can never be taken to a clinic. Those at the hospitals are many, but it is not enough.

INT:All right,thank you…based on you …training, what can you say about training, are they trained well? You can comment…its just that … are they adequately trained? They are trained on….do they graduate in large numbers

P1: well I can say it is not enough because the resources that government bring so that health workers to..to give to people . the people are not trained . new things keep coming up all the time and they are pilled up on to the same people.and the fail to give them to people accordingly. I mean if you are a nurse you bleed, you do everything, you consult,on the other hand you councel. You test, all of these things are dumped on to you. Again expected to attend to children who….supply them with this and that. You supply supplements alone,it becomes too many even what you were not taught .

INT: Yes P5..

P5:I also agree with the ones that say em…there is a shortage of health workers. And I think they are not enough because we graduate em…we do have enough training institutions. Even those who do graduate from these institutions its tough to retrain them because truly the package from government is not at all attractive. You find that someone stays in government for a certain number of years and gain experience feeling that his service is worthy, they can go to private companies or abroad or elsewhere, so its part of the reason they are not enough.

INT:Ok..eh..have you been looking at the numbers have been trained, and retention like somebody has covered it, doe anybody want to add onshe just said including numbers and retention..yes P2…

P2 :um..they..they are..not enough and the other thing that complicate that aspect its like there are expected to do everything, there are not enough semi skilled personnel who can relief them on some of the things,I mean if you consider in.. in a…in a hospital uses..?as an example, for a nurse to just sit behind her computer for retrieving results, that's a waste of time,there are many other people who can..how could be doing that and retrieving this files and putting them into the patient's folders.so I actually feel there is no structure, it does not exist, its just to say go and work.

INT: Yes..thank you….the P6 that side!

P6 :ok, what I can say is that you find that, like in rural areas im a not sure if statistics are ever considered when posting people to different areas looking at how many are needed there. You will find that maybe at IHS a 100 nurses are trained, maybe when there is a greater need in Mahalapye

sub, you find that only two nurses are posted. They are absorbed in to Mahalapye while rural areas are suffering. And you find that thre is only one nurse in a clinic, in the morning she is the one who consult, and everything, so I think that where the problem comes from,statistics.

INT:(clears throat)anything to say on retention on top of what she said?ok… is there anybody who want to add? Ok..Yes P7..

P7:um..i think they are not enough because…I think like P2 said about….statistics, I don't know if people want to stay in Mahalapye in large numbers, are people afraid of going to small rural areas? Because I realized the other day when people were suppose to be transferred,people rejected those posts, that why you heard the office wasthreatening. The other thing like the Doctor said it's a waste…of manpower. If you are anurse and you will be required to go do Data job! I just heard some guy who is a pharmacist who is going overseas because he is placed in hospital just to issue pills, that do not make him happy,it is not his job. So the peole are not being replaced.

INT:Ok..anybody want to add?ok..let see.. …what can you say about deployment/distribution of health workers, what can you say about it? Considering whether they are being deployed…what do you call it? To be posted, now are they being posted in enough numbers? And are the distributed equally considering the hosp…clin..other clinics? That how the question is actually is and he had commented on it so I wanted us to add on what he said (P1) to add more....(whether they are equally distributed in different clinics (P5) yes P1…(me?) no..P5 raised her hand let P5 speak….

P5: I think they are not enough mainly because, its just that , I think I worked in all deliveries of health care in Botswana and tertiary and district level.The one that I did not have direct contact with it is Primary care, I mean from going there visiting clinics, you will find that…that at all levels there is no stuff so…actually the fact that they are not there everywhere, I mean when you look at it from the perspective of being in a rural area and there is only you, you might think there are many people in mahalapye, but at the end of the day when you return to Mahalapye hospital, you will find that still there is a shortage of health workers, So based on me it is just an issue of there is actually a shortage at large across the board, whether Primary or otherwise, so I think when deploying statistics should be considered. Part of it I do not know if we have the statistics, like the lady alluded, I think….i do not know if they have them….the correct ones, the ones that are enough more so that we can say at this rural area we are 20 000, therefore the personnel that is enough maybe 5 nurses or something,I don't know if there are such kind of statistics maybe that what make seem like the proportions are not proportional.

INT: so based on your experience, when you are going to different clinics you find that its like certain clinics have more workers than other clinics.

P5: Um.. honestly I wont be telling the truth if I said there is a health institution that I have attended in Botswana and concede that they are enough. I have never been ina situation where I have had not to be queue of one to two hours ,unless if it is where I know someone and maybe ask for a paracetemol. But everywhere where I have gone to either asa patient or stuff its always overwhelming…this is too much.

INT:.Ok…yes..let me give this P4 a chance to talk she has long wanted to say something I will get back to you.yes..

P4 : but there is..there is I mean there is what is called establishment register..they say the establishment register shows the number of staff a clinic with a maternity should have. But here in mahalapye we're..we're..things are not done in the same way.staffing is not the same.that one is just a management issue; it is up to them to see how they can handle it. But facilities like currently in mookane, the clinic that has a maternity ward has had for so many years only two midwives..i mean it does not..even just management should have ..a few that is there..they should try to distribute them well.

INT: May I please follow up your comment.there is what is called an establishment register which is used when there is need for more staff?? So..what do you think of that, those government establishment registers, those for..for health workers?(P1)

P4: We don't know them we are never told of them, you will only know that a health post is supposed to have one nurse, even if you ask about how many staff members should a clinic with a maternity ward have you will never be told.

INT: Ok..yes P2..

P2: I think these establishment registers are irrelevant in the sense that they look at the infrastructure aspect of the facility you see..its just that when a primary hospital is built its just a primary hospital, if it's a health post it's a health post, but they disregard the services and products that are being provided by that particular facility, so like today the understanding is that there are hardly anybody who have proves but everybody who wants to..who..who is..who doesn't want..ah..those that want to be realistic can appreciate that there is shortage through out the system you see. But these things..these establishment registers.. are no longer relevant to the..to the situation on the ground.

INT: Ok, thank you P2..yes P1..

P1:..But then again I think it's the issue of, I don't know..i just feel we have a tendency of leaving things to be too outdated in that you will find that when you research where these things come from, you will find that these have been established in..i don't know 66 or whenever, and these don't match with the current population, its just that there is ..if we say if onlythey could be done, lets say maybe on a 2 year basis there nwould be continuos review every now and then, because the other day I was hearing someone….i don't know if its true or not, but alluding to the fact that there were saying,there are like they have reach the maximum limits for doctor posts mo Botswana but we all know that…come on..realy..that…(laughs)that apparently there are.. we just have too many doctors that they can't even hire,there are not..according to the government thing.. e there…they have reach their limit they can't hire anymore.(they have got extra doctors)they have got extra 40 doctors,they…they have reach the and they have gone above the limit ka 40 but practically.I mean we all know that that is not true so I feel gore such kind of think should be reviewed every now and then.

INT: Mmm…yes…

P1 : Yes let me add on what you've said, the issue of continuous review you've mentioned is similar to that one of having one same staff member in a health post for a long time, and on top of that that particular nurse will be given the responsibility of a place like mosolotshane which has a huge population. And he/she will be entrusted with running everything in the health post and even forced to work under extreme and unconduicive conditions and insuitable structures. And considerations are never made as to when these structures were built; whether in 1960 or 60 something. And today they want to upgrade to have a particular number of nurses and there is no…like Yako had said that that thing…this thing is irrelevant because they always take things there, its not working because the only consideration is population growth.but there are other considerations like how the structure needs to be improved, how many people need to be deployed, and who. This clinic does not have a maternity ward but I used to help people in that regard…I work at Shoshong in a maternity ward and we help a lot of people from Mosolotshane because that clinic does not have a maternity ward.

INT: Thank you ladies and gentlemen..lets move on to our next question… do you think there are gaps issues or prolems related to health care workers?sorry…do you think there are gaps issues or problems related to health care workers for primary health or not,if there are problems what do you thing are the most important problems?

P4 : Please explain..

INT: Sorry..

P4:Say it in Setswana….

INT: in setswana? We are saying, is there a problem of shortage of health workers in the rural areas? If it is like that what could be the reasons? Like considering if they are adequately trained for the job? Considering what could be contributing to such a shortage?

P1: Excuse me?

INT :Oh..the way she is saying it in Setswana.(clears throat) it is saying…but it is similar to the one we just asked, so it is saying..do you think there are gaps or issue of..or problems related to health care workers for primary health or not?if there are gaps or problems why do you think are the most important gaps? What has the greatest shortage? Sothe other thing is that the nurses at the health posts. You call them health posts?

P3:Mmmhh…

INT : are they doing the work that they have been trained for? The one that they went to school for?

P1: do you want to know if they focus specifically in their duties?

INT: yes that…,that they are doing exactly what they are trained for?

P1: Its just that I did not get you well! i just want to know if you mean the job they have been trained for as a nurse?

INT: that's what I want..i want to know exactly that, that if you are a pharma..what do you call it? If you are a pharmacist do you do exactly that? Just knowing that ok fine, if I don't have any clients then my work is done. Yes P6..

P6:.they do not do their work that they went to school for, they do any other work. They will consult a patient and then move on to issue that very same patient medication. Yes..they take blood samples but that's among doctors' job description, all the work that a doctor should do, nurses do that at that particular clinic.

INT: Ok..somebody to add on? Yes P2..

P2 :. I think,on the aspect of a gaps that are there,I will say its still shortage of manpower because really its unrealistic to expect a critical service like health and you..to be particular?? It will be provided by only 1 individual you see, and then the expectation will be for you to be available to help around the clock.i personally think that you will..you..you..you wont be facing the truth. its..its not possible.

INT: Are you done?yes P4

P4: Yes its tough..its tough because as employees in these health posts,even if you are not a midwife you end up helping as such, because when a person is already here you ought to help them.you know its tough that you end up doing almost everything. Even when you make a mistake they don't consider the condition you were in, its really tough. But then again the pharmacy law does not allow that a person prescribes and issue medication at the same time.but you see we end up doing that, even when I have done a mistake it goes unnoticed but I think that policy was meant to cover up such loop holes. But to be honest we do everything.

INT: Mmh..ok..yes P5

P5: I think this P4 has touched on the issue I wanted to talk about, I personally feel that primary health care is not practiced in Botswana,do you remember that we started this conversation we defined what primary health care is? I don't think it's practiced because when I go to the clinic or take somebody there all they ask is what is wrong, do you have a headache? They will give you medication and will never sit down with you and explain what could have caused your aliment. All they do is consult because they do not have time to do that preventive primary health care that they are required to do,the kind of health care that will help reduce the kind of risk and complications..there is no education offered at all.the pressure that you have because you are alone hinder us sometimes.even us patients do not help the situation because we keep on complaining. Pregnant women have this tendency of just coming..i mean they are..they need education but I

don't think enough is done, mainly because like this woman has been saying nurses do a lot of things, they are a jack of all trades,but the education that is more seesential is not done in primary health care but the biggest challenge is lack of manpower.

INT: are you done? Yes thank you P5..yes..is there anybody who wants to add something? P4..ok. so is there more shortage in certain cadres?

P4: Do you mean within health?

INT: yes P4..(yes within health)

P1:.Ahh..I personally see a shortage everywhere.

INT: You can..you can talk..

P1 : I see a shortage in pharmacy, doctors and nurses alike.

INT : Which one is mostly affected(P5)?

P1 :.its the nursing department, there is a great shortage and doctors take second position.even though I speak of such great shortage in the nursing department, when you take shoshong and its catchment areas there is only one doctor but you know a catchment area has lots of areas like otse, korwe, mosolotshane, moralane and there is only one doctor.

INT: ok..yes P7..

P7 : i.. I think if we don't focus on the cadres available, I think when time to introduce many cadres in the lower thing those that will serve as a relieve to some duties like dispensery for instance because I think we cant have a nurse prescribe and dispense medication while we could have a cadre who can dispense medication and many other things. But if we are going to go with what we call professional

cadres though we are faced with this shortage I really don't see us going anywhere. I think we need to beef up up coming cadres who can do these non technical challenging duties and have professional cadres who will focus on those aspects that really call for their attention.

INT: ok thank you…anyone who wants to add?

P8: i say there is a shortage in all departments,because even us child welfare officers we face challenges.lets say if maybe you are only one at shoshong and there are …there are 8 nurses.this means that they all depend on you,if one wants help they come to you,if they want a follow up to be done they come to you but you also have things you need to take care of. I feel that we all have this challenge of shortage. People retire and they are never replaced by deployed other new ones.

INT: what do you mean, do you mean that when a person has retired the post should be left vacant? Ok P6..yes P6..

P6: yes there is a shortage everywhere, maybe that's why you find that nurses are burdedned by doing all these other duties like taking blood samples and helping but if duties could be specific to certain people it could be much easier. We all know that there are people who are being trained, they are called petrons. Only if these people were hired straight from school, a lot of them are roaming the street but we could do with their help, rather than sit here and wait for professionals with diplomas and what what..

INT: ok thank you..is there anyone who wants to say something?

P3: Yaa..but we have said it, I think it will be just repeatition to say its not only nurses who face a challeng of shortage of staff, we mention them because they are overwhelmed by all since they are also the primary health care nurse, so we will feel that whatever! When you look in to other cadres, even the cleaners. More so that you don't need people of a considerable skill, hence they should have those cleaners, imagine working in a clinic! …that is so dirty, and calls for you as a nurse of family welfare educator to begin your day cleaning the clinic. So you find that there is a shortage everywhere, more so that we are getting worried since there are ARV's that are supposed to be monitored, but at the end of the day there is no night watchman in the facility. You sleep wondering if your home and the clinic will be broke in to. So you find that there is a shortage of primary health care virtually across.

INT: ok, lets continue;. Is there adequate support to do the job? I mean in terms of resources, management and coordination. Are you supported with all the resourcdes you need, and is management giving you support are you as staff getting resources?

P4 : resources are not there.as we speak it is tough to be a government employee, there are no resources at all. All they do is wait for incidents that are in most cases caused by lack of resources and then threaten that they will write warning letters,there is no support at all, all they do is threaten us.

INT: aren't you going to say something about management?

P4 :..INT….

INT: Management ..

P4 :.i am saying management is not supportive, all they do is threaten us,they only step in when something that could have been avoided by them through their support has happened. Of course all these things do come back to you as an employees when they have happened.

INT: ok..someone to add on..yes P6..

P6 : yes the issue of resources ia s pressing issue, for instance the setting that we work within you find that we spend almost 10months working in an area without a vehicle, but where I stay the clinic is situated along a gravel road and the nearest clinic is about 10 km.imagine when a person needs referral what do I use? When you call management will tell you there is no car and this means there is nohow I can help the client.and patients never understand, you see there is really no support, not even to say we will try source something out.

INT Ok..yes P7..

P7 :.Ahh..personally where I work there are no resources, and there is no help that management can give because when you look at it nw, in a catchment there are three clinics, 3 facilities and only 1 car. Other cars are at CTO and some have over a year and even more there but when you ask

management about it they will tell you there is no money and yet they did not do anything at all. And some of these faults are just so small.There is this issue of there is no money these days we don't know where its coming from, there is no support at all.

INT: Mmh..yes P2!..

P2: Yes (clears throat)I personally believe that..i don't know but sometimes management compromises; its like they are more focused on protecting their own jobs rather than doing whats good for this country,so you will find that sometimes,particularly these people called expatriates..you ask yourself aah..is this thing good..the feel good impression they give to the ministry or whoever is their boss, is it going to help the health care of this country in the long run? There is nothing these people are doing but when we say we don't do 1,2,3..i personally think they don't have interest he is more concerned about doing a good name for themselves at their superior rather than create a situation where clients will benefit from their good service, for example if you take Mahalapye, if you take orthopedic patient from Nyangagwe to Mahalapye and in the process the only things that you bring from Nyangagwe are the instruments you leave the complimentary staff but surgeons can not work on their own. Or maybe I diagonize because we are talking about primary health care but I think some situations are similar..some people give attention to protecting their own jobs   than really looking at what needs to be done for the improvement of the health system.

INT: yes P1

P1: like she is saying it is clear there is no staff, we have astayed in rural areas and we help peole who are coming as far as CKGR and a person will come and do what they need to do and interview us but when we call and ask for a vehicle to help clients we don't get that assistance we need.you are never really given a proper answer when it comes to situations like that..there is no support at all and it seems as if we are bothereing them…

INT: are you done? Yes P4..

P4: even the issue of relocation. We did not..we were never orientaited, there is a way central government operates. Like transport, they used to carry us anywhere and anytime, but now at CTO they were saying they are below standard so they had to be removed. Even a person in Mahalapye hospital does not know what an emergency is. So thast means even when it comes to relocation they were not told anything that…that at the council the situationis like this. Even the attitudes that the council people are difficult to deal with is still there. They used to say vehicles are not refueled,

even though we knewe that an ambulance covers 5 villages that are so distant, then you are told ours does not do that. It is never refueled twice…a government vehicleis never fuelled twice in a day. These are some of the challenges that we have encountered while we were still with the council, even now with the hospital…again even our management did not..they were fighting for positions..so they did not work well together, so we expected our management to say in clnics this is how…this how things are done. In Mookane we had a gun shot accident, the nurse was forced by the situation to use her own vehicle. She was told that there was no vehicle yet the patient was there bleeding.

INT:.. thank you;;yes P5..il get back to you..

P5 : I feel there is shortage of resources across board, from human resource to the actual things we use in their setup.we recently had a conversantly at??there are procedures that sometimes a doctor has gone to shoshong clinic, there is no point to carry the patient, taking the whole patient to where there are things you can do right there but you will find that those equipments that can do things like that,another step that I wanted to touch on is that I feel health workers are among the people whose health are being neglected in a way(clears throat)it is neglected in way that is not understandable, in that you have placed only one person, so its basically like expecting even health workers to transport patients in their own cars. I don't think there is anybody really who ever actually think about how this thing affect the nurse psychologically. I don't think anybody, whether management or the government itself, I do not think they look at it that way this person that I expect to work 24 hours how is this affecting their health, so I think its one of those things we should try and look at , the very same workers that we over use more so that at the end we end up …. there is this common thing that nurses are rude, nurse are this and that but realy when a person is tired is tired and quite frankly speaking there is no person who can be friendly I feel its one of things that step from always lacking, frustration of not having the equipments.i could help this person this way but the things that you can help with are not available plus you are just tired you you know?

INT: yes thank you P5..yes P3..

P3: ehe eh…I want to add on the issue of resources and not staffing, resources are scarce and it's a big issue because even the medication we prescribe to relieve pains you can never have it though we order, at CMS the order takes about 9 months and then you ask yourself what do you do all these 9months,how do you manage to help people. These things end up causing things like stress like he said and also as an officer at the rural arears there is nowhere you can get help for your own welfare because you are in an area where it's a clinic and a kgotla full stop. But in the end when you go out to seek help you are needed to help in your area to cover your station,you see? These are just things that we face..

INT: thank you..yes P2.

P2: yes i (clearsthroat) on the aspect of management I feel that sometimes there is just too much pressure that is brought them on the operatives, its just that people who are on the ground more especially if you can look at the issue of lack of medication you see.imagine telling people that the medication they need is not there, imagine what happens to the patients in the process. A person with both hypertension and diabetis for instance who you have put in the proper treatment taking into consideration their illness. If you are going to be helping that particular person you end up substituting the drug that's not available with something else and we end up messing up in the end.and the first rule is that we shouldn't do any harm to this person you see but the pressure is..i think if we can talk to the minister because it has to be a top down approach you see..i feel that that it is very wrong to put pressure on someone who doesn't have the control of shortage to this particular drug because a person who prescribes isn't a pharmacist, when they do drug management, he only prescribes as per the best practice looking at patints' condition and the..a..a..what the patients really needs.

INT: ok..yes P5..

P5: I think the other thing that worsen the issue of resources is that power is too centralized in that, its like they just want..it comes from the top..just getting something small that I fill that ..lets say in an instant where a vehicle is malfunctioning. If only the management from that place would request another vehicle or call the closer clinic that we need a vehicle here. If it were that easy, it could help the situation, but the amount of beaurocracy, a lot of paper work more so that even when you are trying to acquire resources needed by the patients that ahh….you end up feeling lazy to ah…actually the amount of trouble you go to forgetting something very small. If we could try to bring down certain decision so that they can be made at…at primary care level. If I did not know well, the highest ranking person is the nurse. If we could only increase their influence, that theycan make crucial decisions..it can help in that way.

INT: ok..mh(clears throat) so you are saying a person can just say there is no car because they do not want to go through all the process?

P5: I sometimes think that this kind of issue ah...you know when i just think that we dont have a vehicle. A person will say a vehicle is not there,though its true there is no car but there is someway this issue can be resolved. There is..but when you think of the initi..the amount of paper work, the amount of bureacracy you will encounter in trying to get the car ah...you might as well say you know what the car is not there you will come tomorrow or next week.

INT: mmh

P2:.and eh..to just chip in,you can see even its been a while now a hospital like mahalapye operating without an ambulance, the only ambulance which they have is reportedly reached the mileage service and they took it to CTO, and then you ask yourself ah..cant we just write and explain that this is the only vehicle we have so we need to keep it until we get a replacement?

INT:.ok..but when you look at conditions of service and renumerations are they ok?

P-all:(laughs)mmhh

INT: lets raise our hands to air our views ladies and gentlemen(where you work)lets raise our hands incentives,salaries..P7..yes P7..

P7 : we just work so we put food on top of our tables but it becomes tough sometimes. I mean at the moment we work 24hours but il be on a 730hrs shift knocking off at 1630hrs, from 1630hrs to 0030hrs and il be paid standby, what standby when its clearly written 4.30 to 7.30 and I am only paid half the amount, and they say the other half is voluntary and I am expectated to be there when caleed for duty and there are no befits and the salary as well eish…(laughs)

INT: yes someone to add on what P7 said..yes P8..

P8: To be honest our salaries are low, and now we are forced to dwell much on debts because there is nothing we can do. We live on cash loans because they assist us when we are in need. Government salaries are low and don’t even compare to the work we do. When it comes to us family welfare officers you will go on a trip with a nurse, though you all are given money when the nurse claims extra money for overtime you will be requested to take off days. They say…they say you count those hours and you claim them as off days, I wonder why we are never given money like drivers. Its very painfull because we not interested in those day offs but money because you want to feed your children.

INT: Yes let's not talk about money only, how are the conditions of service, is whare you work conduicive for what you do? And what incentives are there?

P5 : yes I mean Botswana salaries are too low. Work and pay is...is not just..i mean first of all like he said our basic salary is low, and then when you look at allowances so many things are just not so pleasant about those allowances. And then again there are no incentives for working in the health care in Botswana in that we don't even benefit first of all and that even when I am in Mahalapye and not feeling well you can not be given any priority..even when the our children are sick we are forced to be in long lines for a very long time though we have the public to serve….you see in the end there are no incentives, there is no money so we are forced to just work because we have to..this is all we work for.

INT: are you done?

P5 :..yes INT.

INT: thank you..yes P3..

P 3: yes those conditions we work in are not conduicive, even our own safety because there are no night watchmen.patients will knock at your door at night because you are on call and you will have to wake up and help them so we never really know if these people are patients or people with alterior motives so you see there is no safety at all. And the other thing our health is also at risk,it is at a very high risk but there are no compensations if something happens to us even if you can get MDR for example you get affected and then you are forced to stay home and the government stipulates that if you have missed six months of work then it means you are unfit for work and therefore they cut you out. These are undesirable things that do happen, you can imagine…we are just here by the grace of God (laughs)

INT: yes P2..il give you a chance..

P2: the other issue is that of working hours! Because you will find that the working hours of health workers because of the shortage that is there, its not ideal as you can find a doctor working more than 36 hours continuously. Then you wonder if the person will be able to deliver effectively since they will get in at 7:30 am and knock off late. when they come in the morning you give them the report but then since there is nobody who can do your work you have to continue again after 4.30. I mean it is something that happens on the ground. But there is no rear estimations that allows it, it

happens, so at the end nobody is doing anything about it, people are just happy that they are volunteering. Particularly those ones that think they are Batswana! You see how it is? I think its something that is going to be similar to the nurses situation. For example where fatique is going to chase people away and cause a vicious cycle.

INT: Mmhh

P5: Again things like accommodation, I think I have worked for government for the past 4 years or so, but for the four years I have been staying in the government house. But the rest of the 3 years even now! Even now I am still paying from my own pocket because I am a Motswana . even when I am on call I transport myself, they are some of the things that I am not compensated for anyway. In addition this things where by you will not be sleeping for 36 hours they think its ok. They say you should have a good heart, but in the end when you counteract! Talking about the issue of safety, the other I heard from one of my friends who works at the Letlhakeng, apparently the other day when he was walking home after work going home, when he got there someone hiding poured acid on him! Just imagine the safety…because you are in good health you end up thinking its okay if you go elsewhere cheaper where you will compromise on your safety.

INT:.. ok..you done? Thank you..

P7: it's a lot of problems, especially when you look at us drivers when we transport …maybe you will be carrying specimens driving vehicles that are covered with mattress, driving in unworthy bumpy roads. The specimens wil spill ..when you get there (laughs) why? Also there is no orientation because I would have done my job of driving the ambulance. The other issue is the issue she was talking about….lets say the clinic is here and I stay at the Zero stop, these vehicles that are not road worthy place us in danger since the nurse will…most of the times they are fortunate, I am not saying they are alright but at times they will be staying within the clinic where there is a night watchman. When a patient comes ten the night watchman will wake the nurse and they will go straight to the clinic. They are also not safe since they are not covered by anything in case they are also attacked by patients. Tomorrow they will be blaming him saying why didn't he stick to his job of ensuring that the buildings were guarded. So they come the nurse will call me even when I am at the zero stop, of which I m ust come irrespective of whether its raining or not, whether there is a vehicle or not! It means I should got to work since there is nobody who cares about how I get there. Even pon my return it would mean I will have to pack the vehicle at the clinic and return home walking. Sometimes they can even call me back. So it is not okay.

INT: Ok..so there is no one who wants to add?

P9: we understand it well, I work in a clinic and I have a proble! I was with another guard, but he transferred and he was never replaced, this forced me to end up working 24 hours. Even the money incentive always comes late and you will always be told to wait. This becomes hard for us. Even as we assist the nurses, its difficult for us because we do not even get tourches, we walk in the dark, risking being bite by snakes and not being able to see through the dark.

INT : ok..is there anyone who wants to say something?

P8: no,our hands are not up

INT: Is there a problem of rural workers in rural areas?(P5)(problems such as, well we have already talked about living conditions (P1) , so we are asking if the living conditions do contribute to the shortage of health workers?

P5: very much..yes..

INT: How?

P5: before I leave, let me say something , personally I prefer if my child goes to English medium, even for me there are things that I wish for that are not available at the rural areas. So it contributes to that! So I will know that if I was given some form of allowance or be given something better, maybe a better package I would not mind to go work in the rural area moreso that as I forbid this at least I am getting that. But now its double tragedy if you go work in the rural areas and miss out on all of these for life. There are no opportunities to work part time, there is no opportunities of doing anything like part schooling, or in terms of doing online courses. I don't know its just that I am frustrated so I …I think that's how those people look at it.

INT: another person! Your thoughts and views? (P5)

P1: yes INT, like she was saying that we do not grow, for instance, you will find that people in Mokgenene there is absolutely nothing that they have access to, even news on BTV since there is no electricity. Even the tuckshops in Mokgenene do not sell newspapers. This comes from personal experience as I had been there a couple of days back. They are out of touch with the world. All they

know is that there is no transport to go where ever one wants to go, unless there are BX vehicles passing by to get a lift, you see how it is. You will not be happy to stay there while there is no electricity, when you can be withput water because they are saying the engine broke down. Water is collected at the well, so nobody will like that. But maybe like the lady was saying that maybe if they were given something better to attract them since they will be working in a rural area. Again someone should not be placed in a rural area for a long time. Lets say maybe a person is there for only one year and then the other person also spend the year there. You will hear that a person have six years staying there. Maybe when a person has a spouse whom they will not easily see because there is no transport..some of these things can be frustrating.

P4: then what do you become? Basically for everyone who comes there…

INT:(clears throat)

P6: Again this issue of the rural areas , you find that there are others who are not going, even if they get transferred they refuse the transfer. I remember the time we were transferred from Mahalapye, around 2007 transfering our small group that recently graduated who would not refuse . ever since then we have been placed at the rural area. When you speak of transfers, we have like 5 or 6 years at the rural areas. When you look at transfer to cities, only elder people who can speak for themselves. For those of us who do not have….we have been placed there,when we will be transferred…. At the moment they are saying there is no money. Only for your age mates to progress more than you.

INT: yes. Is there anything else that you want to add? Regarding barriers and challenges like costs and prices at the rural areas, there is need for schools with value for the children, or wishing as a woman to stay with your mate working there.

P6 :Yes it is similar to it, its just that at our age we have had our children who are around 6 years old, we wish for them to attend crèche. At Maape there is no where you can find a crèche, which means you failed in raising the child. The child will stay with their grandparents and you only get to see her only at month end for 2 days only,you see.

INT: Eemhee..

P3 : the other thing is the nature of the road that we are using ah… when you think of getting in to that gravel, when you also think of buying a vehicle! You become demoralized to even buy a vehicle .

why? Because there is no worthy road that one cannot drive on with your car because then you will only be working for car maintance. Its one of the things that make us wonder! Again when we are there the other thing that makes us seem like we do not want to , anyway we end up going. When you are in the rural area they totally forget that you are there, starting with management downwards. The most painful thing is that even when you want assistance its better you come to the urban area because if you will call they will not help you. You will not receive help because you are in the remotest of areas. These are some of the things that affects us, but at the end of the day we end up going there to assist our fellow men. But if there were choices I do not think there would be a person to go there, otherwise our government has to put more effort to develop industries. I only wish they could give health first priority, since I feel it is not given priority at all. If only our government could recognize that it is a basic need in the lives of the people maybe it could be better. Even the vehicles are forever not in a road worthy condition as they are forever serviced at the CTO. They are forever breaking down, like ours the other time had the same problem. So we ended up borrowing the one from the neigbouring village that was not even in our catchment area. It broke down along the way as they sought to assist us. Then we called another one that broke down along the way as they were from getting the specimens that were supposed to be brought to the city. So at the end I mean the working conditions are very strainuos, they are not conducive.

INT: yes P6…do you want to add anything?

P6: yes INT let me add, the things she is saying, when a patient is here and then you tell them the car has a problem and the next time again you tell them the same thing they lose hope and think you are not being honest at all you see, they will never have trust in us especially if you can spend a longer perion in their area, when you tell them about the car they brush you off immediately.

INT: yes P7!

P7 : I think some of these things are caused by our government. Because I think if someone were to come from outside the village, for those of you who normally watch television , there is an impression that this is done this way, like about three years to four years back, I think it was said…I once saw one of the senior people saying that the health posts will be stuffed with two nurses. I donot know which one since 2007 that have been give an extra nurse at least. But everytrime there are reports in television or newspapers, there are always nice and there are no problems regarding those working in the rural areas, but for those of us who work in the rural areas we are often left wondering…and you wonder why they are misleading Batswana since this is not what is happening on the ground. These things end up frustrating further because we end up thinking it is what is happening in the urban areas, when you come you will tell people not to come there because it’s not like in television.

INT:OK. Thank you very much! (So what do you think of the cost of living? (P5))

P1:Is too high, since there are no shops but tuckshops only. So if they buy Mayonise for P12 and transport it in these roads, they will resell at P24.

INT:mh..anyone to add on that?(those who rent? P5!)

P1: there are no houses for rent! Maybe...there are no houses for rent. Even if you are taken there, there are no houses for rent you will only find VDC houses that have been previously been occupied by teachers, at least they do have accomodation. There will be leakages in the house, more so thst when it is raining you are going to do some drying, but you will be paying the house P200.00. maybe it would be just a room and maybe there would a small kitchen in there or what is it called?

INT:Ok thank you..Yes P7..

P7 : if you could look closely in to the cost of living you will think it is okay. Maybe you will take a decision to go to such a village. You will be staying in a two bedroomed house , while your child goes to junior school, where you have requested accomodation for the child elsewhere, since you will be also staying in a village with no junior school. You request accomodation for them maybe while the others are staying with there parents in the urban areas. So what soes this mean? You are going to travel during the weekend to feed that child and you should also priovide for your mother. You should provide for your family in the urban area and also provide for your self. Having thought you were save. I can even request for meat from this side. It is expensive in the rural areas. You eve have to buy airtimre in order to communicate with them on a regular basis. The other issue i forgot to mention is about incentives, we use so much airtime at work that even at home you dont use that amount of airtime.

P4: are you not employed? Facilities have been given...at least these days they have been given something because we were using our personal phones every month, its like a clinic is given P200.

INT:Ok .anyone to add? cost of living?

P4 :Ah… it is actually expensive! Also it epended on how you look at it, because there is no truth to it, moreso that when you are a person of …ehh..you can end up realizing that ahh..if you want to save you can do that. But if you will also be miserable and frustrated., you end up not even being able to think clearly, because living in the rural area does not necessarily mean you can not buy house in Gaborone. It also does not mean you cannot buy shares. And at times we end up being frustrated and not looking at things from other perspective. Actually the savings are not that much, but if you have intentions to stay in Gaborone would be a problem. Because I started my job at Okavango, but when I compared my things with those that stay in Gaborone I realized I had achieved more things than most of them, yet I stayed in Okavango and visited home every three months. I bought goods in Maun even though it was far. It depends where you categorise it. Even the RASA incentive is about P322, its not much,but it is something.

INT: anybody to contribute?

INT: um..what is this P322 incentive for? (P1

P4 : its RASA, its Rural Area… because there is a list of remote and remotest, but I am not sure because I think remote is around P322, I do not how much it is for remotest.

P7: when it comes to the issue of RASA, this issue of RASA…we once received the list, I realized we were on the list, when you try to claim for it they tell you that the list is outdated the new one will be created, even now there is nothing. They have swept it under the carpet, you will never know if we are delaying or not.

P3:mm… poor management.

INT:Eee.. availability of jos or schools for your partners? Is there anybody who want to talk about it? Whether if your partner or spouse have a different profession from yours, whether it also contribute?

P4 : no INT… I do not agree with that one.

INT: Like being an engineer or something…

P4: that one is disadvantaging us, because for us that live in the rural areas you end up not being with someone that you deserve. But now if the spouses for soldiers will be placed where there are camps. If now the spouses of the soldires will be placed where there are camps, those of us who are not with the soldiers we are going to end up…posted where there are no BDF camps, I do not agree with that one. We should all have families, at times we do not have the partners due to staying in the rural areas. That one will not balance because as we speak now those with the soldiers will go to Tonota, Francistown, and Gaborone. This means some of us will be disadvantaged by the rural areas. Since all the camps are in better developed villages. This means we are going to end up living in the rural areas because we do not have…

INT: are you saying that because you have a partner who is a soldier? (P5)

P4 : no, I meant..no I do not have one. I was just giving an example, that for us who do not have anybody, am I going to be disadvantage by noty having a partner who is an engineer who is hould be working alongside in Gaborone. Don't I have an opportunity, a right to work in Gaborone? Mainly because I do not have someone who is working in Gaborone, do you understand what I mean?

INT: do you think it contribute? That others are…(P5)

P4: it does contribute. But I disagree with you because it would mean that for us who do not have such people we end up staying in the rural areas.

INT: what she is saying in short is how she hears it, it can be both an advantage and disadvantage.

P4 :Ehee..

INT: yes..thank you P4. Yes P1 you had raised your hand…

P1 : yes I wanted to add, at times a person will be married and since her husband works at Phikwe, she cannot leave Phikwe because of her job. And because you as Molosi you are not married you are requested to go to Mokgenene. Hence it makes people's choices to make us suffer since they chose marriage. Then you will suffer because of someone's choice.

INT:Ok..

P1: if it is …mhh..if all of us…hence we should all suffer the same way, we should all be placed there and be given a time line. If we were given this time…there should be a stipulated time of perhaps a year, then someone else go there after that period. It would not have that much of an impact, but I will know that after 12 months I would have suffered I can then go back to my husband or an urban area. Even asking to be transfed to the urban area so that you could also enjoy its goodness. More so that your children can enjoy it by going to better schools that you want them to attend.

INT :Ok.. we can move on to the next one, if there is nothing you want to add? Our next question say what do you think can be done to improve the health workers shortages in clinics? Your thoughts, maybe considering training, being trained at a certain standards as a way of trying to improve! Yes P7.

P7: Umm..i think it can be improved only by increasing health workers where there are shortages first of all. The second thing is to increase renumerations.

INT:what?

P7 : Renumeration, I mean just like that lady was saying that if someone at the urban area has an advantage that they have acquired certain money that they can…after that I will them with the money at the mall. If my children didn't manage to go English medium scholls at least I should be compensated with some money.

INT: Ok..you want to add something P6?..

P6: Yes, no I feel what can be done is to increase manpower where we are trained because you will find that maybe a total of about 500 nurses are required, and the government can only train 200 a year still aware of the shortage.atleast if he can increase trainers I think it could be the solution.they should look at each cadre that does have a shortage and deal with that. Because you will find that those cadres that don't have a shortage the government is busy training more people and they end up in the streets not working…

INT: what do you have to say about the quality of the education received from training? Is it of highest quality or…?

P1:Training on its own?

INT: Yes P1..the training you get there what can you say about it?

P1 : ah I feel its of high quality

INT:Its of high ?quality?

P1 ;Aahh I feel the training in botswana is good because we are better that even people who trained overseas. Even if you can compare a nurse who just completed with one that has been trained outside. They know so much, even the practicals.

INT:.. you can also give examples if you have them.

P1: yes, like when the nurse is fresh from school and he/she is given a health post when they are pretty new,and they are given the Mahalapye area alone..will they ever manage?

INT: do they do the job?

P1: yes they do, they find family welfare officers and they really help..though its tough they end up getting there.

INT:OK..

P1: I wanted to add on the issue of…what is the other thing? You also talked about stuffing, there should actually be stuff, where everyone will be able to do only there job, I am telling it will be better. Right now only one nurse will be required to do every thing, and this not good for them. There should be people who are doing only their jobs. Even the buildings should be well arranged,

more so that you will know that the pharmacy is there, know where the consultation room is! Unlike before when you came and this and that. So that everybody will be able to do their jobs.

INT:thank you P1..did you want to add?

P3:Ehee,I was saying, you were asking what can be done to improve the situation?

INT: yes P3..

P3: I was saying…I have a point of even distribution of cadres, or different people of different skills. The one she was talking about earlier on! The other point that have not be said is the one of extra qualifications. Wherever a person is posted their skill should be considered first, because you cannot really take an RNO to a rural area where you know there is no how they are going to be there without cases of midwifery. The one for martenity, what do you want her to do? If people…if only people were trained in large numbers so that when they come back there is renumeration of some sought, it could be financial, material possesions, or even property it could be better. The other thing…I also wanted to say, they should hire people who will not work for a long time, I do not which word to use.

INT:Part time..ma temporary..

P3: yes temporary stuff like health care assistants, flomotomist. Or the ones that are not highly skilled. There should be such posts, the job would be much better. It is painful is the job is hard more so that when you wake up in the morning would have body pain, also wondering how you are going to manage to run the clinic. It is one of the things that could improve the health care system of Botswana.

INT: what are the other things?.emm..what can be done so that workers work in these areas for a long time? What can be improved? Like when you are deployed to Maapye, more so that you can forget about transferring elsewhere. Yes P3…

P3 :Ah..its hard, it is acatually hard, we have different kinds of needs and wants.

INT:Ok

P3: someone might feel that if the salary could be increased it will do, but it will never. But for me it wont affect me in anyway.The only thing that will come into my mind can be my family, my children, I will wish to stay with children while they are going to a prestigious school. Since I am building there future. I would stay in a place where there is a church, even though we are in health department,they cannot build a church for us. There are a lot of things that we would want from the government, but one of my main ones would be the improvement of things like roads. Other things like…the other thing is health! I am really worried about our health. If there were phychologists or councilors that would cousel us every now and then, there should be also where we exercise every now and then. It could be better, those things could motivate us, they will…it would be like a siurce of entertainment, knowing that from here I am gouing to exercise.

INT: What do you mean by exercising, going to the gym?(P5)

P3:mm..yes, even aerobics.

INT:yes P4..did you raise your hand…

P4: I think people can go to the rural areas of their origin, for instance as I am from the Kgatleng and placed in Leshibitse, even though it is a remote area, while I am there at least I will be close, then maybe I can decide to buy a ranch or something, its better than being so far away. So I think if you are in a remote area within your district, hence it would be that bad for the worker, because really some villages do not develop; even if we could speak of roads, same roads irrespective of how we raise our concerns. But if I am in Oodi that is closer to my village, even if its also tough at least its closer to home than far away.

INT:Mmm.. anbody to add? Ok…are there duties done by health workers that can be done by less qualified people? Like you are nurses are there roles that can be done by less qualified people that you work with like the cleaners. Do you things there are things they can do?

P3: they can be there but with caution, because when we speak of health we are talking about people's lives. I think with training I think those people can do things like dressing wounds, but I think this one need a nurse to asses the wound, but if you have trained a family welfare educator,

they can dress the wound. Things like taking metal sign can reduce the burden on the nurse. But others I do not know but…like taking blood samples , I think the flotomist they are below the urses. They are people who can do that, rather tyhan the nurse leaving their other duties to take blood samples.

INT: anybody to add on to what she was saying?

P4: we can do it with caution because if it is metal sign, maybe it's a baby, then it would be said its 39 implication of there temperature? Its better if not me, but they can help us if there could be done with caution, because right now those from pharmacy have been asked to be assisted by auxiliary, but there are still challenges.

INT: is there anybody who want to add on to that one? P1 ok we will move on to the next question. According to you what initiatives have been tried to solve the problem of primary health care? Like for example, ypu know primary health care is moving from the Ministry of local government to the ministry of health. How has this plan worked?

P-all : you are saying those are initiatives? Initiatives for what? What about the initiatives?

INT: Eee…lets raise our hands so that we can say our thoughts well (laughs) yes P7!

P7: these are not effective initiatives, I actually think they are degrading primary health care. The first thing they are going to post pharmacisits to rural areas, we have a pharmacist at..pharmaciy technician at Seleka, the person does not do their actual job, they are there only for ARV's. they have tried to bring Doctors there, but they are not there to assist other patients but to assist with the ARV program because the pharmacy technician on Monday she is at CVhadibe, on Tuesday she is at..at Selepa, on Wednesday at Machaneng, on Thursday at Pilikwe, and Friday at Chadibe. After that it means it is weekend in and out. They do not get to do their management work. Even Docotrs do not part ways with pharmancy technicians, now they have taken us to the Ministry of health so that we work together as a team. That messed up everything now. I do not know if its because in government there are so many channels, or we are taking shorts cuts! But everything they are trying to do is killing us those at the clinics.

INT:Ok..anybody to add on to that one? Yes P6…

P6: this issue of being taken to the ministry of health hascomplicated things for us, considering the way they nicely reported about it in television. In the radios the management will give patients the impression that they are going to receive better services like pills. So that patients should not get them for themselves anymore, but now they have complicated things for us. They told us that we will have transport, the nurse would take a vehicle from the hospital to the clinic, but that has never happened. Now the vehicles they promised they have taken it and placed them at CTO. Yet the patients are expecting the services they were promised at the clinics as was said on television, there is nothing thatnis alright. I do not know what to mention and what to leave out, things are already messed up. Starting with people's salaries, like at the facilities for instance. When we started we were working for nurses…that meant they exchanged with those that went to school, that menat primary heal;th care remained at the council. That means they work alone for 24 hours every seven days, mainly because they do not have a chance to knock off, you do not know who can assist you. When you try to ask from that person they refuse, yet the patients will demand a nurse on duty, and you are also expected to give feedback. I mean they are situations that you do not know where to report them. At times when you get to the matron and say here is the issue she should…just give it a try at the ministry of health, when you get there she does not even know where your file is! You wonder who can assist you.

INT: Someone to add..about moving..

P1: like P6 was saying that this have degraded the clinics, because when they merged, they did not consider the way we worked. They just looked at it as if we are working only in Mahalapye hospital only. Because in the rural areas we cover mobile clinics and go in to the villages to check on patients. A person can come walking to report that a particular patient is not feeling well, then an ambulance will go get the patient, that how we worked. Now at night, even as we are in Shoshong, places like Morolwane and Ikongwe, amd Otse, they will call for a vehicle that is not there. So even when achief calls to say they have a patient who is about to give birth, we need your help, so this merger I think they only considered only hospitals and then said we should join them. But now what I think happened is that right now as you request for a vehicle…Shoshong is a clinic with a martenity ward, but it has been a while without it having its own vehicle. But now if you have a patient you request for transport that will come, but when you say return us they …we do not transport the nurses.

INT:Umm

P1 ; yes and you have to sit on abench and they will tell you that they transport patients when they have come,no…

INT: what about times when it has happened at night? Like midnight? .

P1: I mean at night, I do not mean during the day, then you will say in the morning you will hike, just spend the night there. While you are still there, the Shoshong clinic will call again because they are always busy, then you will ask for a lift and go back. But then when you get there you return again. They have not done ant arrangements that were good for us especially those of us who work in the mobile clinics. For mobiles, there are patients whom we are supposed to attend, but there are times when we would not have a vehicle for about six months that we can use for consultation. I mean when someone call from far away where there are ranches, where they use two way communication systems. There boses are in Gaborone hence they have put in place two way communication systems for them. They will communicate with there boses in Gaborone, who wil in turn call you from there telling you to…that there is a patient at Lephate who just gave birth but the placenta is not properly removed, this will mean you have to go to her. You will try to organize a vehicle in the morning as you begin work at 7.30 am, even this time you will not have any vehicle. Maybe because there is no vehicle, even when you try to tell the that its for the patient…they will say that when they say there is no vehicle, there is no vehicle, they wil say there is no where they can get the vehicle from. At the council when you called saying there is a patient at a particular place, they will tell you there is no vehicle, but they will pull in. you will be told that there is a vehicle coming, whether you are going to S&CD or elsewhere, but there would be a vehicle there, and you will go get the patient. But now what the government has put in place is a trap, because a patient will die on you and you will told its because you never requested for any transport. Now everybody will not want to take responsibility. I remember I onced called the matron telling her that I have long requested for transport and the time is three in the afternoon, but still there is no vehicle, and I am about to knock off, so I do not know what to do! Then she said if that's the situation I will speak to the boss, that one did not even work as she spoke to the boss. The police ended coming there, but they were tracking thiefs. Then they gave us a lift. So INT the I think this has complicated things further. I think they could have treaded carefully before they initiated anything. They could have considered district by district, firstly considering people… they should have firstly learnt how we work. They should even consider these mobiles that we cover, not to sit only in there offices, they should go and see what it really means to be doing work in these mobiles, how far it is? If there is a patient somewhere…so that when there is a call regarding a patient somewhere the Matron or…what are they called? Supritendants! They should come with us to see the situation for themselves. So they have not taken us seriously.

INT: in other words there is no difference? Its matters worse! Ka mantswe a mangwe ga gona pharologanyo,e bile ke matters worse?

P3: it has destroyed..the difference made is bad…

INT:..yes the difference made is bad?ok..

P1: bad.yes..

INT: yes P3..your hand was up

P3 :.yes I was saying..you were saying what has been done to improve right? that was the question.

INT:Ee..mmm

P3: I feel that the other thing though I do not know I just found things like that, I think deploying doctors to other clinics is an improvement that the government has done, even though those doctors were reportedly for ARV but I think somewhere it has helped but I don't want to say it's a disadvantage but it was the government's effort to take doctors to villages.

INT: mm..but when we look at they say Performance Based Reward System(PBRS)

P4:PBRS?

INT: yes, what can you say about it,where has it improved?

P3: its there..

INT: you are laughing P3..

P3:? Its there(laughs)people may laugh.do you know why we are laughing?

INT: yes P3..

P3 :That's a reform that the government chose to assess our performance, but because we came from the council council we have never been trained about those things like PBRS, we do not understand them. Even though we do not know them, they have transferred us to the Ministry of health. They expect us to submit the reports or is it to submit? These assessment are very stressful, but if there could be someone who can come and explain to us how these things work so that we have a better understanding. Maybe its something that can help primary health care at the end, but now because that we are in the dark, there is nothing that we understand. But mnow this thing is worrying us as primary health care workers, we are having fear that we might not get an increment towards the beginning of april. You get scared that you might never get that promotion, even worse loose your job. The president has come up with the D's, but we have added another one for Dismissal. Its just that people are being dismissed , when we speak of PBRS we are always uncomfortable.

INT: yes anybody to add?

P6 : actually regarding this PBRS, if you could look closely at those at the ministry of health that came from local government, when we speak of where we came from, they start wondering why we are not enjoying ourselves.

INT: them?

P6 : when you speak of PDP's , when there time comes you never know what to do because you will be looking for perfect things that you were supposed to have done. So I do not understand why they never took the effort to atleast train us regarding the work environment we were moving to,but the issue…

INT: someone to add ? ok considering the newly established school of medicine, where do you think it has improved the situation? (it is the one that they have been talking about of placing Doctors at the clinics(P5). I mean since now they are training at home, they can then be able to train more Doctors at the school of medicine, of which they will be able to place Doctors in clinics since there is a shortage. How can thaty improve the situation? Yes P4…

P4: we have not yet seen its results, because they have not yet graduated. But we suspect it is cheaper to train at home than abroad.

INT: mmhh

P4:yes..

INT: because maybe in your clinics they have not yet completed because in Mahalapye they are there, because they mostly learn as they are doing, and they are mostly in the field rather in a classroom. It could probably mean you have never met them.

P3; I have met them when I was working in Gaborone, I have met them they are there. But generally the precence of the studfents is almost the same as for nursing students. When they are there you have the hope that you wont be running around with curnular, at least you know that when there are you do not have to run up and down, you know that at least there is someone to assist the Doctor. But a student is a student at the end of the day they can never perform. If they are going to do anything, they should have a qualified employee by there side. But if they are medical students they should have a Doctor by there side, but the presence of a student has an advantage. And we also have hope that the work that we have been doingt will get better, even though we hear that Doctors in Botswana are over 40 years, though we do not know if our young siblings are not completing their training, so that they are hired. But we have hope that as time goes on, things will be better.

INT: Ok..

P1 : and we hope that since we will be having the Botswana Doctors, communication will flow better, since an old woman can consult with a Doctor alone, since when there are3 three people it means you are crowded in the room and people wont be comfortable.

INT: anybody to add on that? Yes P6…

P6: even the patient’s help, I think if they can graduate in larger numbers it will be better because mostly our Doctors are foreigners. They are from other countries, but now as they are assisting a Motswana its not the same as giving assistance to their fellow country men. They will not assist them efficiently, only so that they can go. So I think that those that are Batswana can do something better.

INT: OK(how have the District Health Management Teams helped?)

P3:(laughs)aaah..that one…that one as it has been said of merging, moving from the council to the central government has destroyed everything, I feel that the DMHT people , even it is a combination of different people, but still the people from central government are dominating, they are only considering their side only, they do not consider the expectations of those from the clinics. I mean even if you had to take a trip, the claim is always difficult, but those are the things that could be motivating us, because you know that if you slept outside your station you will be given at least P129. But for you to fill the claim like my colleague..it took her 6 months, because she felt that it is stressful to claim the money and receive it. I mean DHMT's are there but ahh…they have tried to show that we are together, but there is still that difference.

INT: mmhh, anybody to add? Yes P7!

P7: the DHMT's I think the problem with this merging is that we did not meet collectively, some joined others. Its just that the hospital people think they have us on board. Like right now I think I have the privilege to talk to some people, because when you consider the way people are resigning in large numbers…they are the ones that came from the council, those that are in hospitals since they are having it comfortable they are not resigning. If you could count the number of Matrons in Mahalapye who have resigned, and then look at those in hospital, there is nobody who resigned. PHS of Mahalapye I think she also retired. I understand they are frustrated by the way they are treated by those people, imagine if these guys are frustrated by there own colleagues imagine how we feel those that are there below them in the hierarchy. We are..we have given up because we want to feed our children. But this thing of merging with DMHT is frustrating, but I managed to speak to the head of the DMHT only to realize that they were informed regarding certain things, but luckily he managed to go around the rural areas to see how things are working. He said he found a nurse with 60 patients in a health post. He was to take a tour of the area, but because he is a Doctor he stopped there to try and help the nurse. He said within two weeks he had brought another nurse. But other DMHT's like the one in Mahalapye aee..that one is just sitting there! They do not know that we know that there is Doctor somebody. But whether they can talk to us and visit our area, no that can never happen. They are DMHT's but we do not know there job.

INT:Ok. We are looking at initiatives that we have been talking about, like moving clinics from local government to ministry of health. Even the PBRS. Do you think it has worked somehow? Yes P6..

P6: it did not work!

INT: Yes P6 is there any thing you want to add…

P6:No INT…

INT:Ok, anybody to add something?

P3 : based on my observations rather than feelings.. this issue of PBRS can work, but it would be tricky because we already have a shortage of resources. That one is going to disadvantage us because PBRS is about improving your work. But if it could be introduced to us so that we understand it, it will be able to work. And I will also go for that reform.

INT: Yes P7…

P7: personally I will say it did not work because it let us down, because…is it because in government there are so many channels, because I think at the council..like P3 was talking about the issue of claims, whenever you wanted to claim you knew that you filled it at the clinic and will be signed by whom! Then it goes to account, then pass by SCS who will sign it, then you will be able to go get your cheque. But there! I do not know what the problem is? Tomorrow the money is not there, so its..reallly let us down.

INT:Alright, now I wasn't to know your feelings and thoughts about what can make a difference to the situation as you see it? Yes P6…

P6 : for me in the situation that I am in, that could be done if it were possible; they would return us to the councils and those that were in the ministry of health remain there. Because the management knew us s we were not many, we managed better but now we are just conjested in one place, because now the hospital manmagement do not know who went to school who did not. Since at the council the matron knew her stuff and knew what she needed to do with them. If only they could return us to where we were working before, I think that's what will make the situation better.

INT: to go back to the councils?

P6: to go back to the councils so that the councilors can be able to help us.

INT: Yes..anybody to add regarding what can improve the situation?

P4: for me I would say if we were oriented, like be told that the Ministry of health mandate is done at Gaborone, ours were in Mahalapye, the serious ones were handled at headquarters in Serowe. But we also moved with our attitudes…if all of us would leave our attitudes behind; if only we could leave them behind and know that at the end of the day we are all public workers and our mandate is to attend to patients. The main issueis to orientate. Even though we are about to complete a year, but I think a lot of the work…even to write a letter we do not know whom the letter is addressed to, but in local government we knew whom it was addressed to. Only the supplies department took the initiative to communicate through writing that this is how we order things at local government, if only we could…and be informed of our feelings, maybe then since it is a final decision taken maybe then we can end up excepting the situation and knowing that as you work you are expecting 1,2,3.

INT: someone else to add on so we can move to the next question?

P7: we now have been at the Ministry of health for almosty two years, but the situation is getting worse, actually we think that…we thought they would be patient with us. But its like they do not want to accept us, you can actually meet someone saying you people from the council do not know what vehicles are…yet the same vehicles that we came with form the council. So the only thing that could rectify this situation is for us to go back where we were so that we can improve where we lacked…I do not have proof but I think you knew primary health care was number one, it was better than secondary care.

INT: yes P1..

P1: I also think we could go to the council. While we were at the council we used to get loans from standard chatterd at lower interest. These were advocated by the councils,but now when you there asking for a loan they refuse citing that the government does not allow people's salaries to be deducted from there salaries unless they were using standard chattered bank. Of which they will also charge us the same interest as everybody. Its some of the things that want us to be returned to where we were.

INT: yes P8..do you want to say something?

P8 : yes INT, I was saying we are really working but there are no outcomes. Ever since we have gone to the ministry of health there is nothing that we have access to, for instance I as a cleaner I will have

laundry to do, but if I go to Sefare to ask for detergent since our clinic also have a maternity ward. More so that even when you go to ask for floor polish they will say its not there. But at the council when there was no floor polish, it will be available on Friday; we used not to have shortages in resources like it is happening at the moment. The situation is much worse that we no longer enjoy our jobs more swo that if we had achieved much in our careers we could be retiring.

INT:ok. Thank you very much ladies and gentleman! The formation of health workers teams is considered as one of the initiatives of improving the workers situation in clinics in Botswana; that if there would be a formation of teams, would it improve the health workers situation in Botswana? So I want to know what you understand about it?

P7: what kind?

INT: Like those from…maybe from the clinic in…or maybe from the Mahalapye hospital forming a team in the clinic or at the hospital.

P3: please say it in English, its like Setswana is proving difficult.

INT: you do not understand it?

P3: or maybe just explain…

INT:Ok ..building effective primary health care teams have been suggested as a,..since that was a suggestion , they have not been formed yet, its just a suggestion, so we wanted to hear from you regarding how you think they could work for you. The question is building of effective primary health care teams has been suggested as a potential intervention to improve primary health care? so what is your understanding about those primary health care team?

P4: they can work because the fact that they are a team, since in a team you work together as an entity, rather than working as individuals. But they can actually imnprove the situation since we will not be intimidating each other, since when you are a team there is no team leader, but when you are team you become a single entity to improve the services offered by the clinic. There services can improve because everything happened in the clinic. So that ony one person cannot be blamed, they will know that it was the team as an entity. I think it can work.

INT: yes, I want someone to add on to the importance of such teams? What relevance do they have?

P3:Mm, I think the distribution of this teams will be representative to..like if we are in a team like for instance Mahalapye south, they can be a representative from Shoshong catchement, Mookane, Sefare, and places like Machaneng. I think this thing also will help us for in times of needs like she was saying that we could help each other with anything, I think if we are team we can get somewhere because we will have one goal.

INT:Ok, to add on to what she was saying? Anybody with a different point of view that if there was a precence of such teams, whom do you wish could be in this teams?

P8 : I was saying if these teams can be formed they can do something better. Because it will consist of differen cadres rather management only who might only make sure they are benefiting, it is therefore better that way. They will give us a hard time. Like its happening now with the DMHT'teams, when you go to the matron having written you a letter telling you to go to a particular DMHT. she will say she has written to the dmht but when you requst about the letter there is no answer. They will say there is need to merge the different carrers to form such a team.

INT: yes P8…anybody to add?

INT:Ok, if the team was already established whom do you expect to head it?

P4: I do not know if its protocol or what? The most senior person will head the team.

INT:Ok.. 9. What is your opinion on establishing primary health care teams to address the shortage of primary health care workers? Can these teams work after their establishment.

INT:Ok.. anybody regarding tese teams? Anybody to add? We are about to completer. We are going to the last question and you should ask it in English. Ok.. one of the an important aim of the national health strategy 2010-2020 is to follow clearly established operational plans for health care workers. The shortage of health care worker can render this aim unachievable..Do you remember any

problematic situation in your work you find morally difficult,if yes can you tell us about this situation,about what happened?(P5)

P3:Morally?

INT: mm..like ethically difficult. (P1)

P3 :mm , I will share with you,like the incident… the one she talked about. We had this client,this patient was reported and came with the people she was with. This guy was shot by a gun with the people he was with. So they came to the hospital, luckily he was attended to by another nurse who called me. I went there to assist her,but there was not even a single vehicle . we tried to phone the hospital and the police, but there was no vehicle. We ended up in a situation where my colleague decided that ican use my own car! I told her that I was not sure about that one because I called and was told that there were no vehicles. Imagine a person shot by a gun in the chest, after around 30 minutes I received a call from the police saying they are on their way and the vehicle they were using did not have light and it was not in a good condition, they just wanted to take a step of ation. But the situation of the situation changed , he was a patient who breathed spontanueosly, but his breath were alright, but all of a sudden she had respiratory distress…now that was a problem! My colleague and I got a van, but what can we do? I mean if its like this we look at the situation that what if the patient dies? Firstly if a patient dies in your car, whoever is going to come will never ask you…they wont look at it that you took a step of action to take the patient to the hospital. They will ask you why you had to transport a patient in your car. Even when you made such a decision considering the patient's situation and the urgent need for them to be at the hospital. Then there was the issue of what could have happened if my colleague's vehicle could have broken down or had an accident at night! It was at night early in the morning. What if we had an accident? You see how it is? You start asking yourself if you can do it, even the person who brought the patient will beg you to use your own vehicles, you see! That significant other was putting pressure on us. We ended up taking a decision that was good for the patient at the end, but it was not also good for the patient, it ws good for us. But not disadvantaging us especially that we were dealing with a human like at rural areas where there is no management to listen to our grievances. That why I said at the beginning that I felt that government is not taking health seriously…they do not prioritise it. I still stand by those words because the way wea re treated, like now if you go to the department like BURS, when you there you will never a vehicle whatever that you do. They are never in short of vehicles, but when it comes to health… look at Mahalapye sub district, the size. The shortage is greater more so that the hospital can lack a vehicle, so that it collect a patient 80 km from the hospital. That why I feel that this kinds of situation are not alright. For me that is what I went through.

INT:It was a difficult situation..but did you manage to assist the person?

P3;At the end we end up using our own car, luckily we came across a police vehicle, we took one and half hours to meet us where we were and we then we transferred the patient at the police vehicle because we felt that we were with other workers its better because they also assist with transportation here and there, and then we ended up coming to the hospital so that the person get assisted. Haa… you will never know.

INT:Anybody else who have encountered this problem. Yes P1…

P1: For us the problem that we encounter is CHPC patients, the Doctor recommend them for come for review at such a date… they never get to see the Doctor at all, which will mean they will fail to honor the appointment, then they re-book at the end they end up passing away.

INT: Someone to add on to that so that we conclude.

P1 : yes… I was not there on the particular day, but I was given a report that a pregnant woman was admitted. She camre complaining about high blood pressure and dizziness, so they ended up calling Mahalapye to say there is a pregnant woman here in this condition so that they can send a vehicle. You will call all these places like the library, schools. The schools were saying they only have a combi. But as the nurse was calling, the patients began to have fits due to the high blood pressure. At the time the nurse was alone since there was no… she ended up transporting her with her own car to a clinic in Shoshong with a martenity ward. So when they got there it meant Mahalapye now did not have transport. They ended up requesting for a van from some government department that were on their way passing through, and they agreed since they were willing to transport the patient since she was having fits episodes. The van had no canopy, so the nurse had to ride with the patient at the back transporting her to Mahalapye. And you know how twin cabs are! But she ended up taking her, but it is difficult like you ended up using your own car even though it was not necessarily good for you and those that helped you use your car remain as if…but if you were going to have an accident nobody will give you money to…then you will also be the one driving and at the same time attending to the patient, and also writing at the same time writing. Then again you cannot just write, because you will also be driving at the sametime, since she is also having fits episodes, more so that you can end up causing an accident that might result in the patients dying. That would mean one's journey with the government will be over. And everybody will isolate themselves away from you, leaving you to answer.

INT:Yes..anyone wish to add? They are difficult conditions…

P7: I did not hear your question, were you saying as a patient or worker?

I

INT: Whether you are worker or you are not a worker, whether it happened to you or if you saw it happening to someone.

P7:Mmh…I have a problem regarding…I will give an example of the area we work in. there are patients whom the nurses feel they have the right to be transported, yet there are other patients who I feel are in the same condition yet they are not transported. The other thing is that apparently there are standards, so I do not know if these standards stipulates who is suppose to be transported or not, so this always give us problems moreso tha we end up arguing that this patient deserve to be transported. The other one I can debate regarding the case that I have, I have a problem in my cheek, so I see a governemt specialist in Gaborone. I have had this situation for the past 7 years, I have had 6 operations at the same place. So I ask that I be moved forward quickly, but I am told that it would involve writing of many letters. So it's a long procedure, so I am still waiting with the condition that does not show any progress. That is very heartning.

INT:Ok.. have you said everything you wanted to say? Thank you, we are very greatful to you. Once again we thank you very much, so we want to re-assure you gore whatever was said in this room will stay in this room.(P1)

THE END!

Q
