# Supplementary material for: Stakeholders’ Perceptions on Shortage of Healthcare Workers in Primary Healthcare in Botswana: Focus Group Discussions
Source: PLoS One. 2015 Aug 18;10(8):e0135846. doi: 10.1371/journal.pone.0135846 (PMC4540466; doi:10.1371/journal.pone.0135846)
Supplement: S14 Text — (PDF) [file pone.0135846.s014.pdf]

HURAPRIM PROJECT

Participant ID:Focus group

Date: 28/03/12

Interviewer: Dr N

Interview Duration: 01.50.33

Audio File Name: Focus group Health Workers one(1)

INTRODUCTION

INT….Your understanding ofeeh...Primary health care?Because studies have shown that there is a shortage of eh... health care workers in the primary care system, especially in rural areas, so what is your understanding of primary health care? Yes P1!

P1; according to me primary health care is giving health services to...to the community eh …in the rural areas.

Int: At the rural areas?

P1:Mmm!

Int:yes P1! Thank you. That is her opinion, what do the others say?

P2:I think it's it's a system that provides the first line...eh to care to patients, that's where patients are first consulted before they can go elsewhere.

Int: mmhh...Thank you P2!

P3: ee I was going to go along the same line, it's primarily the first contact of the client of the health care system and is basically aah… focused on preventative measures, rather than dealing with chronic and complications of disease processes the first contact, so all prevention all health education should start there.

Int: mmh thanks P3, eehwhat do the others say?

P4: Is to motivate people or community about health education during home visits and other outreach.

Int: ok during home based care and other outreach!Thank you P4, any other idea or opinion? Ok... So do you think there are enough or not enough health care workers in Botswana? If there are not enough why do you think it is so? Do you think there are enough or not enough health care workers in Botswana if there are not enough why do you think it is so? P5!

P5: I think there are not enough, because the institutions especially that train nurses here are few, and they train less number in a year, so you find that less than five hundred Doctors and nurses are graduating.

Int: mmh…yeah maybe I should even give you the points to look at, you can look at whether the numbers trained are adequate, and also take about deployment and distribution you can also talk about retention while trying to doctors this eh...This question is they enough or not enough health workers in Botswana? P2!

P2: eh...i don't think there is enoughaah... and mostly is to do with the distribution of facilities around the country because eeh..We turn to focus mostly on developed areas.

Int: developed areas?

P2:Yeah like the cities and turn and forget mostly thethe rural areas so in rural areas, because of lack of facilities we turn not to find the government supplying more health workers because of lack of clinics,so if there was more clinics maybe the supply will increase.

Int: so when you say you think that problem is distribution you think that but if there were to be equitably distributed there will be enough there is enough pull but there are not…

P2: still there will not be enough because i think when we go with the amount of health workers per population per certain number of population; there is still a long way because we are not training enough to go about.

Int: mmh alright! What do the others say? maybe we can talk about this one of training before we can move on to the next one are there adequate numbers trained according to you the number of people that are trained…..yes P1!

P1: to add on that, again there is this issue of greener pastures .people turn to go out even if there are there they try to go… to go to work in a better area where they are paid better than here.In our country like now in the past years a lot of nurses were moving from here to the places like the UK, and even the Doctors!When you are looking on the side of Doctors who are trained outside they sometimes prefer to work on those eeh areas... countries where they are trained sometimes there are a few whowill be coming back to Botswana to work in Botswana.

Inter: thank you P6! What do the others say? P3?

P3: ee... no um…I still believe and agree with the previous speakers that we do have acute shortage

of health care providers in this country, and if I remember quite well one of our former health educationattaché was telling us about a research that was done by UN one of the German embassies about our training especially in Botswana. Doctor's ee… they revealed some shocking results that at the rate at which Botswana was training Doctors.Every time it will take atleast 150 years to have adequate number of Doctors covering the whole country and he said the prognosis was even poorer when looking at tertiary level health care in terms of training post graduatepeople. So much of our problem wascoming I think to the fact that we did not have medical school and therefore it was quite expensive to send people abroad to study. because some will not even come back like P6 alluded to greener pastures conflict and the fact that you have better packages better living conditions ,better many incentives that really will keep you there rather than coming back here. And our problems now in rural areas are we send people there but we are not retaining them in rural areas why because there are no incentives for people who work in rural areas.People prefer to stay in townsstay in cities given the opportunities; to further their studies, given the opportunities of electricity clean running water, and the basic amenities that should be there for a person to live a decent life….yah!it's a reality that there are not there in our rural areas and therefore unless we address and give people incentives to continue to stay in places that are notreally the best in terms…nobody will move from their rural areas it'stheir home they know this all that they know.But thehealth care services must come to them, how do we retain people who go there? we must go beyond the ordinary that we give to somebody who stays in Gaborone to somebody who stays in makakung there should be a difference and the person should feel that although I am not getting the benefits that I will get in Gaborone but still I have an added something that will make me stay in makakung.. Something. Some resign you tell them we are sending you to kareng automatically... they resignation letter they send in a resignation letter.So how do we address these issues?I think eeh… I have spoken!

Inter: ok! P3 has talked more about retention.Can we just finishup this issue of whether the numbers…the numbers of people that are trained that are trained are enough before us…?

Int2: I thinkto make things easier, if you are comfortable, because we really want people's opinion. it's a small number, so if you are more comfortable speaking Setswana I think most of us can understand Setswana and if need be we can try and speak back in English to see whether we have captured what we are saying.

Int: solet's talk some more about the number of people that are trained, whether thereare enough? Let's hear others opinion! That was his opinion and he…yes P6...

P6: OK! I will try and speak in Setswana. Training is not enough, looking at the health services we are providing in different cadres especially… even in general nursing you even trying to be a general nurse, but when you get there at the service you find that all those other people's jobs are piled onto one person who is a nurse. There having the training the way you are being put there let's say let me give exampleatmakakung.She is the only nurse there at makakung so she is supposed to provide all health services there, she is a nurse, pharmacist, and she is a midwife.All those things there are put on her and she is alone when she is there she is on call from the 1st till the 31st, if she refersthere is no body there! I think the training is so low, I think at least if they say they have trained enough they should be able to occupy all those areas, so that there is at least two in the health facilities such as health posts that are said to be far. And while you are still there alone they will require you to go to

the mobile stops! While you are the only nurse there! Therefore training at the institutions is so low.

Int1: ok! Thank you P1, P6 any other opinion? Anybody to conclude on the question? Yes P7!

P7: ee…I will speak in Setswana.

Int2: P7

Inter: P7….

P7: I also believe that the training is so low.

Intr2: Raise up your voice!

Inter1: let's raise our voices.

P7: I believe they are trained in fewer numbers because there is a shortage in clinics and hospitals, you will find that people had long queued, the nurse having a difficult time dealing with the patients. At times when the nurse has taken leave the clinic will be closed. Meaning people will go to neighboring villages.

Int: thank you P7…P7. Let's talk about deployment and distribution is the health workers evenly distributed across all parts of our country. Someone had mentioned it briefly, let's try talk some more on the issue!

P2: to go back on that one two minutes I think we shouldn't only focus on training becauseI think the main thing is recruitment! what is lacking is recruitment because training is another way to fill the void, but if there are other ways that the government can do like we know that sometimes we recruit from places like Cuba, I think even in terms of nursing we can still recruit from other countries to fill the defect of…..

Int: thank you P2, let's talk about deployment as I ve already said let's talk about deployment, when health workers are they evenly distributed? Are they equitably sent to the rural areas? Yes P5!

P5: I think they are not distributed equitably considering district wise, you will find that there is low number of health workers in the north west district. If they are asked to go to the north west they resign. For example when we were placed from school and were told that we would placed there, if we were 20, 50%(half) will resign!

Inter: why do they resign?

P5: I do not know if because there are no developments thats why they resign. For example, in clinics in Maun you will find there are 5 nurses, while in Lobatse they would be more than 10, and ...

Inter: so what you are saying it's the people that choose to resign?

P5: yes, it's because they would not be satisfied with the working conditions there for them to go there.

Inter2: do you think the numbers in clinics a subject to is it because eeh people don't want to go there or do you think that maybe they could also be considering the population size being served?

Justjust wondering!

P5: I do not believe it. I believe people do not want to go there. For example if 15 nurses are needed only 10 will be available. While the remaining five will go work in Lobatse, while they were also given nurses, meaning they will increase. It's a real example that if some refuse to go somewhere they will be hired elsewhere, yet they are still part of Botswana government.

Inter: ok...Thank you P5.eh P2!

P2: eeh i think P5 is right when it comes to distribution there is a bit of politics, for when distributing people especially in our country aah… depending on who you are!Whereyou are from, your first appointment.Most of the time is based on that if it so happens that maybe you are from Maun and there is somebody from Gaborone and you are both nurses the possibility of youwho is from Maun to be distributed to maun is very high and if that person fromGaborone is distributed to Gaborone the Gaborone person distributed to Gaborone the likely hood of her resigning is very high.

INT2: To Maun?

P2:To Maun yeah, and then you look at situations, sometimes I think that issues of population is being looked into because if take for instance our unit in Gaborone, the dental unit in Gaborone its full of doctors I think there is probably around 20 doctors in there, and then the population because of their infrastructure.Their infrastructure doesn't allow them to see a lot of patients, but because it Gaborone they are all there and then you have people at Sefare seeing the same kind of population that is the same as Gaborone, so the reason why it's like that is totally because of politics that people know so and so. And they will be stuck in Gaborone, so that's why sometime distribution is not fair.

Int: thanks! Doctor politics involved. What do the others say?

P1: I also believe that favoritism also contributes.

Int: favoritism?

P1: yes. If you say here, if I am to be posted to Makakung and I refuse to go, the next person also next time will also refuse resulting in a chain, nobody will like to go there. They will always say why that one?

Inter: ok thank you P1! Aaah what do the others say? P6!

P6: again on this…this issue of the distribution of health care workers to other areas, when time comes to transfer people they refuse!

Int: transfers?

P6: yes, maybe people are transferred from places like Gaborone when they have stayed there for more than 10 to 20 years. But when you tell her to come to areas like Seronga and Gumare that side, they will refuse. They will go to the Doctors and they will come with a lot of medical conditions what...What...but basically when you look at the issues you will really there is nothing on theconditions that someone hide behind them, others will tell you they have children and they are going to English medium schools. But when you go to places like Seronga and Nxauxau you can't find

these things. They are the incentives issues, and you really feel bad about being transferred there, they will brag about us having gone to school and being in the same class with them, and that we will stay in north until since they are not moving anyway.You end up seeing that really people are refusing to work in areas this side like Dikwa and Betsha that are far! They are faraway places with nothing; no transport, no communication. It's a very irritating situationto people, even when you are around P5's age. I really feel sorry for them because the last time we were in Okavango, we talked about that issue that at least those mmh...Clinics or the health posts should be run by two people; at least there should be a midwife in that area. Those that re in C1.Since when you are a midwife you are everything, you can can cover all areas. Since when you are a midwife you can cover all areas.

Inter: eeh P6, thank you! You are talking about solutions that we are about to talk about. Let talk about retention. What your take on retention? Are people that….

Inter2: yeah because what we are trying to find out is the problem that there is shortage is aproblem of inadequate production or recruitment as you said or misdistribution or is it a problem of poor retention that the people live the system so it it a multiplicity of all this, that's that's we want to get your opinion on. I see the P6 has been working for 30 years in the same place, but maybe P6…. (Participants laughing).

P2: i think it's a concoction of all those factors, firstly ehhh people are…there isn't enough people trained there isn't enough recruitment and even the distribution is not fair, and then for those who have managed to go to the rural areas, retention is very poor.Because people do not like it there so there is nothing that keeps them there until we talk about incentive. Other retention is very poor.

Int: thanks very much I think P2 has summarized it unless there is anyone who wants to say something else before we move on to the next question? Ok… Do you think there are gaps/issues or problem related to health workers for primary health care or not, if there are gaps or problems what do you think are most important gaps or issues or problem? We can look at whether there are adequately trained for the job that they do...ehhh are health workers assigned jobs they are adequately trained for? Is there more shortage in certain areas than others? Ahh is there adequate support to do the job in terms of resources coordination and management ehh and the conditions of service? Some gap issues, are there any gap issues, looking at whether people are trained for the job that they do? Or whether they are assigned the jobs they were trained for? Maybe we can start there… ee P4?

P4: I will speak in Setswana, I will say it is not right, you will find yourself youwill be transferred for a job that you have been trained for, but since there is a shortage you are a jack of all traits. It's a double edge knife, since if you do not do the job you are wrong, since personally you will be looking at saving the patient's life that would be there. It then means I should do someone's job, it's only here and there that I would be doing the job I have been trained for. Most of the time I will be doing other people's jobs, I have to do the job because there is nobody to do the job. I will give an example of where I am based; we issue medication since there is nobody to do that. The nurse consults, after that we dispense medication! There is nobody to dispense medication. Also there is the issue of rationing supplementary foods for children, the nurse do the checkups and those from child welfare will then go in to the store room and ration the food. This means you are doing the job that is not in

your job description. That is why I am using the hook that we are jack of all traits, any job that is there you do it. But now you will be forced by the situation there to save someone's life.

Int2: maybe…listening to what you were saying, I will also speak in Setswana so that we understand. So when you are doing the job, will you be feeling that you can do the job that you will not be causing problems since that is also part of what we want to find out?

P4: yes at times you will have the ability, it's just that you will find that the in service training….in service training is there to show you how you can do things, even though you are not allowed since you will not be trained on such a job, but the situation there forcing you to do it. In the in service training I…I have started 30 years ago in a health post, I was even dispensing, consulting, dressing wounds, doing child welfare, I was basically a jack of all trades. Ever since I started after being trained for my job, I was shown how to do certain things in certain situations that force me to act.

Int: jack of all traits, what do the others say? Master of all problems! What do the others say?

P2: I think the main thing with our government is exploitation, since government is is a nonprofit making organization, we turn to be more of a charity organization.So people who work for charity in turn are very loving. So wherever we are, we help people anyhow we can, whether you know the job or have been trained, somebody comes in and you look and them and say I can't do this I have to help this person, so you try.So a lot of people are not necessarily trained for what they do because of lack of man power, if you are a doctor and you are some where there is lack of specialist, I mean before you can even think of referring.Most of us will attempt to do what's there because of lack of training, and staff like that. and when it comes to other issues there is lack of management ,people are not trained in management, so they just role play and try to improvise and that's the most problematic issue lack of trained management people are into management because of being a member of the service. You go by the ranks until you are a manager, yet you are not trained so that creates a problem a huge problem.

Int: thank you P2…what do the others say regarding issue of people doing jobs they are trained for?

Int2:although we have listed a few things about regarding the issue of gaps and problems, you are not limited to just what we have mentioned.

Int1: P1

P1:I will speak in Setswana, In government, especially in botswana, if you are there at the rural areas you are doing work that you're not supposed to do. Like someone just said you are jack of all trades, when you are being trained there you are being trained as a nurse under the nursing service. But then when you get to the field, its everything, even jobs for other people; you consult, you dispense since there is no pharmacy technician, you do child welfare. when the health education officer is not there you are a counselor, we do not have a counselorthere for pmtct in kareng, all those things there are on that officer, by that time you run all the rooms around … as I was saying this girl is non-midwife and she is being posted in makakung, there are lot of deliveries there in makakung and kareng and there is no midwife in kareng, when the situation come the woman is in labor and she don't know how to deliverthe woman.Thesituation forces her to do that work, really in Botswana we are doing everything, there is no manpower who are trained for that service.

Int: ok …what do the others say? Maybe to summarize Ok, so let's talk about the support, is there adequate support to do the job In terms of resources and management and coordination?Are they receiving enough support or are you receiving enough support or not?

P3: Support and resources to do the work? I think the main challenge is that there is a lot of centralization, take for example equipment that we need to provide basic services in clinics… in hospitals. Even mops just for cleaning everything comes from CMS.There is no specific budget that you can say this is forletsholathebe Hospital, Letsholathebe will say how to distribute the money according to their own needs they will give their own quotation, that they will need so many …. Tubes from their statistics looking at the number of years they have been dealing with these things. Voice...ok!And there for much of the time we are lacking behind in providing service because many of the things you need to do the job are not there, we will be waiting for two months just to get a singledrug we will be waiting for two monthswithout even basic normal celli.

INT: mmmhhh...

P3: because it is centralized we are not independently. We are getting resources to provide resources in the nicely timely fashion that you would rather do. So centralization is our key weakness.

INT: mmhh... so he has talked about resources,let's talk some more about resources and maybe give examples. Ehh...if we can can...regarding resources. P6

P6: eish...really we have alot of problems when it comes to resources. Our resources are not adequate. For me looking at the situation nowadays, since this migration of clinics to ministry of health, we have problems. There is a problem of resources pertaining to...one transport. You will find that maybe there in the facilities there is no transport. There is no ambulance at all. There are no even resources, there are no Doctors. There is nothing!

INT: mmhhh....

P6: and in other facilities you will end up even lighting with candles, just imagine! There is no electricity in the facilities. Andit's ...hey...so iritating. To be there in a facility with patients waiting for your assistance. And for you to reach a point b…there is no how you can reach that point. Our vehicles are gone. Our vehicles are at CTO, others have gone to the hospital... and it's a very big problem when it comes to transport in our side, especially for those of us at the far end in the healthposts in the rural areas there. It's really painful!

INT: you said the vehicles are taken where?

P6: the vehicles are in the hospital!

P2: they are centralized (interrupting)

P1: they are centralized now.

INT: ok...

P6: now they are in the hospital and...And you are there at the local. ..Facility when there is no

transport and the vehicles are not there, maybe they are having gone for service. Maybe there is something happening. You ask for transport at the hospital, they will end up maybe waiting for some time. Maybe for the whole day. While here in front of you, you could have saved lives.

INT 2: maybe so that i understand, how is it...how was different then and now? The situation you are describing!

P6: mmhhh...actually for us when we were in the local...ministry of local governement, there was shortage of transport yes... it was there, but not like nowdays. When you compare it with nowdays, thesedays it's bad. Extremely bad.

INT: mmm...P10 do you want to add something? Ok…I will come back to you.

P8: yes INT…I want to add on what P6 was saying that there is a greater difference. Ever since we moved from local government to the ministry. Like for example, we were volunteers that helped patients at home, home based care…

INT: we will talk about the movement from local government later.

P8: ok...

INT: we will talk about it as we move on, just hold on it! Yes P8…

P1: I would say the resources are not enough in the health facilities. Let me maybe touch the transport issue…you will see each…it's just that the in facility they are…they are being given those vehicles, maybe let's say…in such a way that the transport is one. And that transport they use it to transport the specimen, they use it to transport patients. The very same vehicle is used to do outreach programs for mobiles. The service associated with the transport is the only Driver allocated to that clinic. The Driver is also having difficult times, they are even working during the weekends, and they never have off-days. Even though they work together with the Driver fromMakakung that we use sometimes, it's really hurtful. When he knocks off, other people at the other facilities will use the same vehicle. They transport machines, specimens, for official trips, it's not enough in the area that I am in at Kareng. I have a clinic there it's a new structure, but its ehh…a building that really could…ahhh (laughing). That building was built with a maternity wing, but it's just OP. is has no electricity. We have an instrument there…even the bed side lamps they are just toys there. Theydon't have the electricity in the health facility. and when the patient comes at night you are expected to go attend that patient in that big building using… my this cellphone for me to enter that building so that i can move where there is a ehh.. Cardec lamp… just imagine if you are having an emergency, you are being called at your house to attend to that patient and it's an emergency you don't have electricity. You run around to look for a light.

INT: some with a torch!

P1: let's say maybe a patient is having difficulty breathing, there is no…I have to take that patient to sit down, i don't know if that patient will reach a seat or what. Whereasthere is equipment there that is just toys! Mm…

INT: ok...thank you P1. Before we move on...P5!

P5: I know that the resources from the remotest…since I come from very remote. There have been there since the opening of the clinic! Since 1978, and they are still using old equipment. The infrusture is outdated; the seats are so worn out. There is nothing that is…windows are not closing. I feel that people are not supporting us. They never come to see where they can renovate. You will find that maybe they were using two way communication system at the rural area. There are no longer working. There is no communication. I mean if I did not buy airtime a patient will die in front of me because I did not use my salary to buy a cellphone…what is it? To buy airtime to call. It's really painful to see someone suffer while you do not have airtime. Then it seems like you do not want to help the person. And it's painful to use your airtime for work related purposes. I mean there is no communication.

INT: ok...thank you P5...last words! We should talk about coordination, management! Ehh...the conditions of service! Theconditions of service, and management, I mean how are management? In the primary health care system. How is management?

P2: management...in...I can...in general is very poor. Because if you were to take example of the movement from local government, at local government i think they were better managed. The...the...even though there is shortage and what not. Ehh... the shortage wasnot bad and it was understandable and they could cope, but now they gone to central government, in central government the management is harp hazard,ehh.. Starting with budgeting for instance, there is poor budgeting and most of them. Most of the time when they budget, they look at the overall ministry. And some of these small facilities are looked down upon. So they will just support ok, like letsholathebe maybe they will be supported. But it might end up here and not go straight in t the rural areas because of poor budgeting. Andthe. The way they the government does ehh the procurement it's very poor, the way they procure materials, equipment and what not. It's very poor because they they also look at big facilities as priorities. Like for now marina as a referral hospital it will get priority before anything else. Yes they all serve Batswana. So managers really at the top there, they cannot discriminate between facilities, looking down on rural health services as if they are not providing enough good services to Batswana. And the other thing as well is accountability. Most of the managers are not accountable because they don't go in to these rural areas to see exactly what is going on. Ifif you were to ask him right now what is going on in tsau, most of them wouldn't have a clue what is going on.so they are not accountable.

INT: thank you P2! What do the others say? It's like the lady is not clear in what P5 is saying, we do not hear what P5 is saying!

P9: mmhh...you will understand!

INT: ok...what do the others say?

P9: yes, also me...

INT: management...

P10: regarding…especially the issue of management, I think our management is not alright, like here in Ngami…

INT: ok...your name? Name...

P10: Marietta

INT: ok...

P10: I think our management is not managing, I mean it is failing. Like I will give an example that I work as a counselor. It's like certain things are only in certain districts. I mean like when they bring something or a new initiative, when something is supposed to be done…but they are going to be pilled on the table. But when you go in person to go check you will find that those things have never been touched. Like I can give an example that we were hired for 3months on a temporary basis. But at the other districts people have been hired on a permanent basis, but ours are just there! Unless we go there every day to check. Or maybe ensuring the ones at the ministry to call here. Maybe that's when something better can happen. Like right now our contracts have long expired, we have not been paid. But it is said a person should at least be. They say 14; I am not sure how many! I am not sure if its 14 days or how many days for a person to have already been paid. But ever since the contracts expired we have never been paid. Then you wonder how someone will survive.

INT: ok...thank you P10...aahh...ok P1...

P1: management. Maybe you are going to blame this people, this management here in Ngami. Maybe because they are not trained for that, maybe they are managers because they are there because they have been working for...on that service. There is there is...let me give you an example like Okareng, okareng is a big clinic with a big population from also the smaller villages, but that clinic is still underPRS,more so that she has never being training for anything related to that management. Then expects to manage a clinic like she has been trained for that. Maybe this even the ones in …the ones also in the hospital are just being blamed while it's actually the government's problem.

INT: mmhh.thank you P1. Anybody to summarize it before we move on? Who else can summarize it for us? Before we move on to the next question.

P3: i think they have touched on it well. I think the big problem is…training people especially for management. It's because we assume that as long as you are a doctor, you are a nurse you are being exposed to running your daily duties in the hospital and therefore you have managerial skills. People in other hospitals there are people who are hospital managers. They are people who are trained specifically to run the welfare of the hospital, people they are trained in public relations, they are trained in management of different areas, ehh...for coordination of the hospital and the district. But here and elsewhere around the country they trend is to assume that as long you have the qualifications you have your MBBS, you have your RN certificate that should qualify you to be in a managerial position. There has to be a need for us to go beyond the traditional method of doing things. We have to train people in to managerial positions, send them to courses, send them to degrees, specifically for management purposes so that they have adequate distribution of resources and ehh...coordination of event in the district, and hence improve overall improvement of the health care system of Botswana.

INT: thank you Doctor! I think he has talked about it…ahhh…I think we can precede... is there a problem of adequate health care workers in the rural areas? If yes, why do you think it is so? Maybe we changed a lot. Things like that. But i want us to look at the living conditions in the rural areas and

whether they contribute. The remoteness of the areas or some parts ehh... the cost of living in rural areas ehh... the opportunities for continued or continuing professional development or further education, the availability of jobs, schools for partners or their children. The living conditions in the rural areas do that ehh do you think it contributes? Living conditions! P5.

P5: eee...the living conditions at the rural are not ideal! You will find that there is only a primary school in Makakung. More so that when you have a spouse and children, you will have to make a plan so that they are able to attend at least junior school elsewhere, which is something not nice! Even us we do not have transport, there is no bus to Makakung. For us to travel is through the use of horses…if not a horse then a donkey (participants laughing). There is no mode of transport, absolutely! If I had come to Maun and remained behind, I will have to see how I return since there is no transport.

INT: thank you P5, what do the others say? P1!

P1: to work in the remotest areas is really the most…

INT: cost of living...we are talking about cost of living.

P1: ohh...we are having a difficult time here in the rural areas! Actually as I am, with children you expect your child to be like…the one whose parent work in Maun. You want them to attend pre-school, you want them to attend junior school, and there are no private schools. There is not even a tuck-shop that you buy from. All those things count, even there is no electricity, there is nothing, there is no life there, we just work there because we do not have options.

INT: mmhhh.... aa...what do the others say? Yes P11…what is your name?

P11: Mmm...P11

INT: mm...P11.

P11: eework conditions...at the rural areas are not conducive, like raising children, basically everything is not ideal. Even as an elder you will not manage to attend your church because it's a rural and it's prone to traditional beer. At the end of the year you will end up drinking traditional beer too, joining the groups to drink alongside them! The conditions in the rural areas are really depressing, they are not very encouraging in anything, and instead they discourage you. Even when a 5 year old get there they would be my size (participants laughing). We will be age mates!

INT: thank you P11…what do the others say? Living conditions! Ahh... the opportunities for further develpment or further education? In the rural areas!

P5: may I ask a question? I think when you are at a remote…at the rural…people tend to forget us. Later you become a deadwood! There are instances…if there are things that people are being trained on, you will about them from people working in Maun. Those workshops…because they feel I do not need those workshops, it is only needed by someone in Letsholathebe only. That means as I am in the rural area I will tend to forget certain things more so that I become a deadwood, when you ask me how certain things I will have forgotten. I will be like P11 was saying that you end up Drinking traditional beer, you will close the clinic at 12 noon, and you will open it tomorrow just to go drink traditional beer. It's not at all nice. There are no shops, the cost of living is expensive, to buy food

here and take to Makakukung is not easy. There are no shops, no bars; there is no where we can meet at least to… (Laughing) this side is worse. I know the other rural areas, but the ones this side of Maun! There is no electricity.

INT: P5 has talked about the opportunities...for continued professional development and also the living conditions. Yes. What do the others say? P10!

P10: even when you are there and you want to develop yourself academically you will there…you will be old not having managed to do that, without any options to develop further. You will be just buying vehicles, but there won't be anywhere you can get your license, but when you try to get it here you will fail to do so.

INT: thank you P10! P6 do you want to add something?

P6: I wanted to talk about this one…issue of electricity. If there was electricity in the rural areas at least, we could buy ourselves laptops, even when there is network internet we could enroll with UNISA. But it disadvantages us when you are in a rural area. I mean there is nothing that you can do to upgrade while you are there.

INT: mmhhh...what about the availability of jobs and schools for partners? (Participants laughing)...

P3: i think it its self-explanatory; health care conditions are so green for you. How much more about your partner and your kids? While you are working they are sacrificing yourself, your kids, your partners, and the general welfare for further development as a person and as a professional in your field. You are so much disadvantaged, given the same qualifications, given the same basic training you are expected to compete with someone at Gaborone, atFrancistown, while you will be inseronga. For job opportunities, for scholarships. Somebody will get to know about it, newspapers reach there after 3 months. Somebody sees it there and there they apply and they have a competitive advantage over you. And therefore when you are in rural areas, i always advocate for incentives that will keep people there. Because you are disadvantaged in almost all areas of your life.

INT: thanks P3! P9 do you want to add anything so that we do not exclude you?

P9: I wanted to add on to the one we just discussed…

INT: aaahh...go ahead

P9: on that one of rural areas. The one about how problematic rural areas are. Last year there was one of my colleagues, I do not know if it's okay to go ahead and say it? We were…

INT: no names...as long as you don'tdivulgewho it is so that we cannot identify the person.

P9: ok…we were in a workshop here at Maun Lodge, we came for training...(interrupted)

INT: raise up your voice!

P9: ok! We were in a workshop here in Maun Lodge for RHT training…. (interrupted)

INT: voice! Raise up your voice!

P9: we were in a workshop here in Maun lodge for RHT, when we were doing the introductions she then immediately requested to be transferred away from Bodibeng, at least to be transferred elsewhere. She was being terrorized by...i do not know if it was only due to witchcraft. She has a year trying to ask assistance from the churches, like ZCC. When she realized…she is not even going to work, you could tell it was not nice the way things were going. Even a gathering had been called at the main Kgotla the issue being discussed with the chief. Ahh…she ended up…she ended up deciding to leave anyway from the rural area. She opted to rather lose the job. I mean something else can be torture, especially some of these supernatural things, or what do you call it? The ones that are happening in the rural areas.

INT: ok...thank you P9! What is your name?

P9: P9.

INT: P9?

P9: yes...

INT: thank you P9. I think we can proceed, we are chasing time. What do you think should be done about the lack of health care workers for primary health care in Botswana? Ahh… you should look at the issues of training, deployment, distribution and retention strategies. What iniatives can be used?What should be done? In terms of training, what do you think should be done?

INT 2: since...ehh...this is important because i think one of you said, that the people who are there are there, you know, i know somebody once said maybe a little irresponsibly by saying people should be moved closer. But i mean how do you move people closer? When you live in Makakung, if you originate at Makaukung i don't think anybody wants to move from the grave sides of one of their ancestors. Soobviously there need to be provided with services, so what can be done to make sure that ehh... there still should be services provided for the adequate number of people. What can be done? What can Botswana do? What should Botswana do?

P3: eehh.i think one of the basic areas to start is when you get people there, don't forget them. In other words there are people who live in one area for 5, 10, 20 years. There should be a program where other people are given the chance to experience and transfer to better facilities. So that you don't keep someone indefinitely in one area that disadvantage. That disadvantages them for long. And just improve the basic working conditions. Let your clinic just have your basic electricity. The facilities that you have ehhh...athsmatic patients comes in there is enablizer there, but you can't get anything. It's becoming a white elephant, at least make the basic things work for the person who is there, so that the work optimally with the little they have. Yes... that what i think the basics will start from.

INT: thanks P3!

P2: ahh... the main issue is when it comes to training people, is to train people according to the needs of our country. Ahh...rural areas like she was saying ehhh to take somebody like a general nurse to a rural area knowing that she is going to do everything is not the right thing to do! So if the government can target specifically looking at the needs, for instance sending ehh midwives for instance, to rural areas because those those do a better job than just a general nurse. So is to train

people appropriately looking at the needs. Ya and, to train more, to train more people.

INT: thank you P2! What do the others say?Training! Deployment and distribution...

INT 2: or any other that is not listed! That you think should be done.

P3: incentives…incentives, let there be a difference between someone who works in Maun and a person working at Makakung...(interrupted)

INT 2: what sought?

P3: what sought! Financial. Financial and ehh encouragement with job opportunities. I mean further education opportunities that you stay in a place for three months, i mean three years, and you are automatically entitled to go for further studies. Compared to somebody who stays here for 5 to 10 years.They are here they can do an extra course, they can do ehh yah an extra degree via a university, interne t access is easy, they are accessible in Maun. They have easy access to other job opportunities they can find greener pastures while they are here. While you are lost in the rural area that is it for you, so there should be something that encourages you to stay, knowing that you are looking forward to something that would reward you.

INT: mmhh.other strategies eh…P5

P5: looking at training, I think there are other employment opportunities right here in Maun, it's just that Maun is developed, but they are not there in the rural areas. Like there are people who assist in the hospital, those who collect blood samples, those who….work in the hospital. I think they can be trained in areas such as Tsau, Kareng,andMakakung. If the people who are assisting could be trained. It's really not nice to work in a rural area while you are alone. Places like Kareng you when you work as a midwife; you attend to pregnant women, attend to patients, you are forever in a hurry. While you are at training still…when they train in auxiliary you can ask questions about the vital signs. Someone can be trained so that they are able to assist in any way they can like at Letsholathebe having assistants. Instead of you doing everything, after that you travel 174 km to come to Maun. You refer and leave ,then come back later, you get tired

INT: mmhh...ok thank P5, P1!

P1: eish! I am saying government should train the different cadres that are based in health facilities. But in the training you will be trained as a midwife, and you are doing all other services that are supposed to be done by other cadres. And our work according to our training as a midwife I will be just like P5, I will have a special course to be a midwife, but there is no incentive that I am getting! I am like…. we are being promoted at the same level, yet I have the skills similar to her's. Yet she can end up being my senior even though we are in the same service, because government incentives. All the cadres like pharmacy technicians should be trained if there is a greater shortage for pharmacy technicians that can help. At the end of the month you are supposed to…and those people at the ministry have given you a deadline. They will want their reports. While all the duties are piled on you as a health worker in a rural area. Like P5 had already said, at times if the nurse is not there, and she is the only health education officer there, the patient comes and there is no stuff, I have made a referral, when a patient comes, when the community comes they expect you to assist them, whether

you are trained or not. Because you are a worker within that village.

INT: mmhh.thank you P1. Maybe we have been talking about that. Ehh what about task shifting. Ahh..Maybe for example, are there any duties that you feel. Duties that you do that you feel can be done by somebody else. Perhaps somebody of lower qualifications? The duties that you do.

P2: i think that one P1 touched on it! And actually the support stuff, i mean if. If she was to improve on her work she needs assistance. Some of the things she is not meant to be doing, for her to carry out her job properly. So i think support stuff, the issue of support stuff is the main one in terms of stuff in the clinic.

INT: mmmhh...lets talk some more about it...ehh maybe even give examples! Task shifting, duties that you do that can be done by someone else. Or even people with lower qualification...P5!

P5: I think people can be trained on how to counsel when it comes to issues of counseling. Because I think in the rural areas if you had done the course on HIV testing, RHT, you are forced to conduct testing. But I feel if people who have done these things could be trained, it could reduce the work load, and even stress associated with work. Because working alone is not nice…while you are standing, when you seat down someone wants your help in the other room. It is…I feel it affect our health. If you are at a rural area. Let me not say when you are at PHC, when you are at primary health care because I worked in primary health care in Maun. You will find that it's similar like when you are at a rural area, because every job at the clinic you have to do it, whether you are in Maun or not. In Maun you find that you do a lot of those duties. Like P1 was saying that they did not…counselors were not employed. It then means the nurses covered their jobs, But in Maun they are greater in numbers. If there could be people who are hired who it would be known that they are there, not like they are just there. If there contract expires it should be renewed. They should know they are needed time after time. If the contract have come to an end, it should be renewed in the same way a Doctor's may be renewed or a nurse's being renewed. It should not be 3 months without them because it then means the work load is greatly increasing.

INT: mmmhhh.thank you P5...aaa...anybody to summarize it for us? Before we move forward!

P10: to add on to what P5 said, I was saying they should…there should be home based volunteers. I was saying they should also be trained for that job! Its risks so that they are able to assist the nurses. For when a nurse is alone, or while it's the two of them. Like at Maun clinic, so that they attempt to fix things there. So that they know how to care for patients at home since they would have knowledge. So that we do not have a situation where they won't be able to assist the patient on the basis that the nurse has gone to attend prenatal care.

INT: thank you P10…what are the others?Who should be trained to do what? Examples...P4 before I move on!

P4: ee...the other thing is taking blood samples!

INT: taking blood samples?

P4: yes people should be trained, there should be people who take blood samples since every

Tuesday and Thursday at the rural areas blood samples are collected. Because people mostly want to be done by 11am. But if they get here late there are normally vocal about its late coming.it would be better.

INT: ehe.P10did you want to add anything? Before we proceed.

P7: I think if you are a health worker at the clinics, that is why I am saying maybe I work in health education, at least if I could be taken for 2 months to be trained, so that P10 is not around I can be able to supply milk forchildren, so that at least there is someone to issue milk.. Maybe when there was a single nurse like I was saying, not being able to assist those children. I think if you are trained you can be able to help the other person even if they are not around, so that they can do other duties

INT: thank you! Ahh… in your experience that is the next question. What solutions or interventions have already been tried to try and resolve solve these problems? Maybe we can talk about ehh...the issue that was raised by P10 earlier on....moving primary care from ministry of local government to the ministry of health?

INT 2: that one there is nobody who saw anything good about it, i have heard everybody say it was bad! Is it really bad?

INT: P10 you wanted to talk about it! I think she is the one who…

P10: ee...i was saying ever sine we moved there, there is nothing that is alright compared to where we were coming from. Iwant to give an example that right now we have patient of...we had home based care volunteers. Ever since we moved from to ministry of health they have not beenremunerated…they are only given transport allowance to assist the patients in their homes. When you inquire there things are all mixed up, they would say people should submit their id's so that they get paid. Nothing is like where we came from; they have not been paid until now. There is so much difference compared to where we came from

INT: another person. Thank you P10, P5!

P5: I think our moving from the local government is a burden on the ministry of health. I feel that Primary health care is so large more so that it needed to remain where it was, having its own management. You will remember some people were retrenched because it was said they were the council's plans. I think council. What do you call it? Local government its main aim is to made resources and services accessible even to the remotest of areas like Makakung. You will find that there are other jobs like security, they are not secure? There are no security guards at the rural areas. Even I am not safe. I know that I will knock off safe if there is a security. Even if a patient comes, security can know it's a patient, but if he is not there I will be in danger, maybe it's someone who is intending to kill me. Since we moved to the ministry of health…we do not have...the security guards at all. But when you go to a hospital there is someone who is monitoring everything that goes in to the hospital, because it is not safe.

INT: thank you P5…is you done? You have said everything, thank you P5! Aa..Another person! What else?

INT 2: what is government trying to do? We are still talking about...

P4: yes. I will talk about the transport issue. Transport it's a problem, you may find that specimens have been there for a while! While they were supposed to be taken to hospital, since the there was no transport.

INT: have they done anything to add to the issue of transport? Because we are talkingabout strategies that have already been tried by the government!

P4: ee, at times there is no vehicle, asking where the vehicle is!

INT: mmhh...any other?

P2: the other is the issue of recruitment, i think what the government has tried to do is to explicitly recruit ehh...the likes of the cubandoctors,ahh foreign nursesto try to fill the void. Like i don't know now, but i know there used to be a lot of Zambiannurses, Zimbabwean nurse and the like. That was just to try to fill in the gap of shortage of manpower. And i think they are still doing it in some specialties as well.

INT: thanks P2. Yes, did the P11 want to add something?

P11: eee…I was pointingout that while we were still in the council, we had our own supplies and pharmacies, and hence it went well. We used to run short of Doctors, but not that much, but now when you request you are sent next door. You are referred there and there. The Doctors has really gone down, we need Doctors most of the time.Even the resources, for us we had a person who was focused, knowing the number of clinics with maternity! The others I have this many. But now even if you go to the supplies people they do not even know what you need.Where do you get those things, you have to go yourself to find a quotation for yourself.

INT: mmhh...thank you P11! Eehhh... strategies, we can talk about..ehh maybe the setting up of the district management teams and maybe the introduction of the medical school. also maybe to evaluate them because that what will do on the next question, at the same time cause we try to evaluate the strategies and see if they are....

INT 2: no no...I was saying we are not limiting that, we just wanted to just. These were just prompt to help you think broadly. What are strategies that have been and they have worked?

INT: initiatives that were tried!

P2: in terms of management, i think like you were saying that that government has tried to come up ehh management health teams, the DHMT to try to centralize, to centralize the management of the district to one. But it is still a new thing so we don't if it's going to work or not, because here for instance here in Ngami it'sstill a challenge. It's not properly setup, even if when you look at it the the head of the DHMT is the superintendent, the hospital superintendent. His priority is mainly punitive, so he is just going to try to address issues of the hospital more than anything else. you come from the clinic from the rural areas, he might not listen because he is looking at his patients, so whether it will work i don't know, it's still too early to know until its properly implemented then we will know exactly where it is headed. Butit'sa its good initiative cause it will probably solve some of the issues

like the ones that came from the councils and stuff. Because at least things will be together.

INT: mmhh...thanks P2! District Management Teams and also the medical school what do we say about it? managementschool. I mean ehh...medical school...ehh particularly in the rural areas. Is it working?

P3: ehh... it think its a positive development from the government and the university to start the medical school, ehh trends show that where there are medical school ehh services get delivered better because people are trained, researches are done and therefore how resources are distributed, they are supported by that sorry the presence of the school. In its self it's inherent that the researches have to be done, and for the research to be done, some of them will be done in these rural areas. Here people get there recommendations, serves the needs of people first hand. And therefore it will have impact in the policy making and decision making, on how resources are distributed to reach people. And to know exactly which resources suit them the most according to their needs, based on the research that would have been done. And therefore in the long term it's a positive development that would have more manpower, more medical doctors would be trained and eventually more specialties. Specialists would come from it. Andeventually helping the remotest of the remote. At least in a clinic you will find a doctor or two. in the long run if we continue to train people locally, because it won't be that expensive, maybe where you trained one doctor you might train 10 from the equivalence of the amount now yah.

INT: he is talking about the possible interventions which are actually the next question. What interventions will be the biggest difference? Interventions that can make the biggest difference in improving the situation? Let's probably look at what we talked about in the previous questions. Interventions! P1. Or anyone!

P1: because i don't understand the...i was saying maybe i am quite because i do not understand the question!

INT 2: well it's probably more repetition of the previous question, but i think it's just to say do you think there is one thing that if we can do this, this will make the biggest difference to supply of health care workers especially in rural areas? P5...

P2: for me the most important thing is incentives. If we can bring proper compensations to people working in the rural areas, a lot of people would volunteer to work there, yah than just being ordinary like any other person who are working in the urban areas.

INT: thanks!

P3: and is not just out of the blue we have neighbors in South Africa for example somebody who works in Johannesburg and somebody who works in Mpumalanga the remotest of the parts. the further you are from a major center, the more incentives you have and some people are posted in Johannesburg but they rather opt to go to Mpumalanga for the reason that they have incentives that they can benefit from, they might go there three four years and come back to the city they know that they have scarified but at the same time they have benefited from the system so we can bench mark at list in our neighboring countries if a need is there.

Intr: mmh I think he gave a very good example, does the P11 want to say something?

P11: I was saying also we should stick to the agreement, if you are going to Kareng for 2 years, it should be strictly 2 years not 7 years, because I think that way it will make people know that it is 2 years then I will be out. If they forget you that'swhen problems begin. The other one I was asked what could be done? Years back midwifery used to be compulsory becauseI think now midwifery Is less than general nurses, that's why there is a shortage and you find a midwife being overworked she is alone. What if it can be made compulsory, so that every nurse is trained to be a midwife? So that if a nurse is taken to Kareng she can be able to do everything rather than them hiring one, it should be compulsory.

Intr: ok thanks I think P11 is throwing the question to everybody! We can have a look at it.

P1: I do not think it should be compulsory becausereally there are no incentivesin mid-wifery. It only brings frustration on us, that's why maybe these kids do not have interest in it. Maybe if there could some form of incentive, maybe they will want to go train because today if I am like me them there is no relevance for me to do midwifery, but if it will be compulsory we would be put under pressure.

P4: but in 10 years to come there won't be any midwifes.

P1; it's up to the government.

Inter2; so you think we should get carrots not just sticks (participants laughing!)

Inter: I think we should move on to the next question,becausetime is not on our side, building an effective primary care team has been suggested as a potential intervention to improve primary care in Botswana what is your understanding of an effective primary care team ? An effective primary care team? P5?

P5: I think an effective primary health care team is a team that will bring all resources that are needed by people especially in the rural areas. I think that would be an effective team.

Int: aa what do others think? An effective primary health care team, maybe we can talk about also the value of it, an effective primary health care team? P6?

P6:I think an effective primary health care team it must be a team containing of all the cadres in the health care system, so that it can be able to meet the demands of all the cadres of those people in the health care system. and also it be there to make sure that it distributes the resources equally or if by need arise looking at the at the at our area that if your area and the population, how best can we divide our resources in the team. I think it could work if it's like this.

Int: who should be members of this team who should be in this team? Somespecify rules what are they supposed to be doing?

P6: mainlyi think there should be a doctor, there should be a nurse mid wife in the team,and there should be somebody from the health education division. all those departments that are in the health care, there should be a member for each responsible for her departments that they should come together and share ideas from each side regarding their needs and how we going to share the

resources and plan ahead when it comes to issues of health.

inter: maybe what I wanted to say is that what you are suggesting then that each clinic should have a full team with a doctor a nurse and a health education assistant is that what you are suggesting?

P6: basically nowadays I think each clinic should be having a doctor, there should be a Doctor pharmacy technician there should be everybody responsible for her part in the running of primary health care

intr: the reason why I'm asking is that I recently had a hat with one of the young Doctors that I met at a meeting he said he has been posted to Boteti and said there is nothing to do you knows it's the first time there's been a doctor there by 11. I have seen everything I want to see and they won't even allow to go and make a call at Maun I am just saying looking at what you are suggesting is.

P6: what I am suggesting we will be looking at the clinics, since there are clinics we are having clinics the bigger clinics ee... like clinics with martinity those ones there can be a doctor in those clinics.And there are clinics maybe or health post where there can be maybe a nurse midwife. maybe two or three nurse midwife in the clinics, then in health posts like Kareng

Inter: ah…P5?

P5: I also feel that we do not have Doctors especially in the rural areas, they are mostly in Maun. I do not believe there is a doctor who can leave her fromMaun because it is a great distance. What is it called? I think in school they called it accessibility or what? It is so far to come from MAKAKUNG to come and see a doctor here; when you get here you will find a huge queue. If there was at least a Doctor in Tsau or Kareng, that will be better. Again so that we reduce the greater distance for travelling in the gravel. Especially when a doctor is not needed at Makakung, I mean in reality they are not necessary there because they have fewer patients. At least if he is at Sehitlhwa or Karengsince this side we do not have any doctors. You will find that the doctor starts with Maun clinics in Maun primary health care. It's the one that have doctors outside of Maun. There are no doctors more so that even patients are not receiving resources well even the ARV site. It's similar to kareng, it gives ARV''s, there is no doctor there; it's a doctor from Maun. And times they do not come at all, because like today we left Kareng around 10 am but the doctor was not there. I believe he has not even began his journey there because we did not by pass him, so I believe we do not give patients our clients a good service, while they have come there at 6am in the morning, at least by 9am he should be finishing more so that after that he go assist in the cattle posts. I mean P1 can even comment on that given that we do not have doctors especially in Sehithwa.

Inter: ok ok thank youP5!P5 has given her opinion on who the members should be on the team. What do others say? Who should be the members of this team?Who should be on this team and the roles what are they supposed to be doing? The members in this team, the health management team, the primary health care team rather, who should be the other members in the team? P5 has talked about Doctors, Midwives. Who can the others be in the team? Who should be the leader of this team?

P10: (whispering)Doctor

Inter: P4 says Doctor!

Inter2: but she is not very confident saying that.

Inter: why o re Doctor? why should it be a Doctor

P10: I would say a doctor since a doctor is the head of the department.

Inter: what do the others say? Is it a general consensus? P7?Who should be the other members and who should lead this proposed team?

P7: I think the members can be all these service providers; the doctors, nurses, health care assistants, counselors, basically all the service providers. They should all be in the team.

Inter: ok...is there anybody who wants to add anything before we move on? How should the quality of the teamwork be evaluated and impact its work? The impact of this team how should it be evaluated to see if its working or is not working

P2: I think already in govern we have a tool that we use to assess the efficiency of how people work the PBRS, I think it's a good tool to assess people but the problem is people are not using it effectively because they do not get benefits out of it but it's a tool of assessing anyway so it's one of the tools we can use to check if the team is working or not

Int: how does it work?

P2: aah INT I am not explaining PBRS, (laughing)

Inter2: maybe doesn't it er" that one looks at what I do as an individual, but when three or four individuals are put together to say now you are a team and you are providing primary care to this population here how are we going to be sure that that team is effective.

P2: it starts at an individual level because as a team to work a team even though it's a team it's made out of different people, right? So every, a nurse would have his own objectives of what he wants to achieve in a year, a Doctor would have his own, a health promotion person would have, so even though it's a team its working in the same community but with different objectives so collectively I don't know how that can be assessed but it starts at individual level.

Intr2: I am just going to be the devil's advocate again and push that a little bit further, when I started work at the university were recruiting for the school of medicine we really struggled to recruit and there was a joke that we had that the HR team the average was 98% but we still didn't have people so see what I am saying maybe saying it's not being used effectively but the individual I think well they and also in a hospital you find…(pause) I think we are going to be chased out if we do not have lunch, in a hospital you'll find maybe the superintendent gets 80% ,everybody else get 90%or 80 and yet the complaining rate, the rate at which patients, the services that you are providing the rating isn't going up ,so that although I think that works maybe that works for an individual I don't know whether that always translate

P2: it doesn't translate because it's not used properly cause it's a very subjective tool that's used by people the subjectively of it is thing that spoils everything if it's used properly if that 80% could

reflect exactly what goes on I mean we get 90% but while the job is not 90% so that's the problem its very subjective it doesn't show properly.

Intr: mmh P3?

P3: I think the bigger picture I think would be the clients that you are serving would be the true reflection of how the primary purpose of primary health care is prevention, prevention, prevention, how many of your diabetic are well controlled that they do not go into complications, how many of your hypertensive ,how many of your …….. patients come to primary health care , how many kids under one year who die before they reach one, so those are parameters that we can objectively measure to see how well your team is doing I think if we were to do statistical analysis that every team documents and gives reports of their mortality rate of their how well, because in other countries actually people are paid based on how they performance ok you are given a clinic you ran a clinic how many people in your area their diabetic is controlled their hypertension is controlled, and they do not go into complications you are paid according to that ,if our health care system primary health care in particular because if we manage them so well not so many of them should reach tertiary hospital they reach their because they failed in the primary setting and therefore our measures at the primary site should be tight , that the team should be holistic if there is no body who is going to do health education if there is nobody there to deliver services at home if there is no body to go and make sure that a patient is getting their TB treatment infect ant diseases can go rampant in the village and therefore all these measures the people that are needed to make this work as a team should be there and they should be doing the work and how do we know? By the number of the decrease in infactantdisease the number of chronic diseases that are going down well controlled so I think that's an objective measure to……….

Inter: last words in regards to…

Inter 2: you had raised your hand!

P6: they have already covered it.

Inter: last words in regards to primary health care team before we can move on? Ok aah the last question, do you remember any problematic situation in your work one that you found particularly morally difficult, if yes can you tell us about this situation about what happened for example maybe you had to turn patients away for a particular reason or make them wait too long, basically what we are looking for personal experiences.

Inter2: I think this question isreally looking at ethics because eeh ,I don't know whether any of you like some of you said have seen a document produced by the ministry of health called integrated health services management of 2010/2020 in one of the pillars that said there is ethics, that you know patients will be treated in an ethical manner, but we are aware that you know shortages and other reasons mean is not always only shortages may make it difficult for somebody to actually always make one act in a way that is un ethical so basically what this is asking is that have you found yourself in sometime in situations where you ve acted in ways that are rather contrary to your choices or in ways that you rather not work but because of the way you findingyourself and situations that you find yourself working that way, these are just examples but you can give others whether sometimes you have made people wait too long or you send them away because you

couldn't serve them for whatever reason in terms of human resource and why was that difficult these are just personal experience.

Inter: P2?

P2: I will start with the one of DHMT, here in our department oral health we have an issue of segregation of DHMT, we have the Okavango DHMT we don't have the dental facility there , and here there are two separate DHMT but the thing is we don't have access to patients in Okavango because the DHMTS are fighting over budget issue, this one does not want to transport us to Okavango to treat the Okavango patients they do not want to pay for subsistence allowance to go there so we find ourselves lingering there not able to helping the Okavango patients, then what happens is those people will have to travel all the way do not know what the distance is or 600km to come here and sometimes when they come here somebody will have to come in at around 4 o clock because they been looking for transport all day and what not , and then at 4 our instruments are in a sterilizer or something like that, you find yourself in a situation that you cannot help that patient because you are sterilizing instruments at that time and you have to tell the patient I cannot help you after somebody has travel for 600km it's a very painful situation and some of the issue you find is that we can spend 2 months without local anesthetic and we are turning patients away saying there is no local and yet at central medical stores sometimes you find out that local anesthetic is there, but nobody because of priority issues nobody is willing to take the care from here and send it to Gaborone to get that ,because some of these things they do not find them as priority is not like somebody who is in labor they will just say ahh dental, you see for us in our department is very painful we find ourselves in such dilemmas and is not right.

Inter: thanks P2, more stories? P1

P1: hey… really we are working under difficult situations every day every day and night, nowadays there in rural areas we do not have the security we are being called by patients. They go to our houses to knock saying we should open for them, and we will never know if they are patients or just other people…whatever we are at risk in the rural areas. This is very painful on us, and you work in a situation where you do not have electricity. You go to the clinic you assist the patient while using a cardec lamp, which also places your life at risk .at the same time we don't have the communication, this communication …but while there are networks that are already installed like mascom and bemobile. We are trying by all means for them to provide us with communication so that I can be able to communicate with makakung or Sehitlhwa. I use my phone and my airtime while I travel during referrals at night. Butit's the same thing even during the day. It's useless because when I get to the hospital I will still be queuing while I am all the way from Kareng with an emergency. There is no Doctor in SEHITLHWA, you can imagine the distance!

Inter: so what experience do you remember with regret because of all those circumstances?

P6: hey...because god would be with me, I have had asituation of that magnitude. We are working under tremendous pressure due to a place that is not conducive. The experience I had one day I had this patient who was burnt at the mobiles, I think she was 100 years or something! She was being referred to Kareng with whole body burns I had to take that patient I didn't have time. I was lucky that time there was a doctor on duty at kareng. But if she was from makakung I would have had to call for transport at Makakung so that the patient is transported. I put her in an ambulance and she

was pink all over her body. I referred her to Maun, more so that I was praying that she does not die along the way. At least if we could reach Letsholathebe with that patient. I was happy that that day the doctor was on duty, more so that when he heard the ambulance siren, he was the only one who was prepared. But our colleagues were just relaxed, unless the person was IDC; he is the one who came to help us with the emergency…

Intr: (interrupting)mmh ok can perhaps eh perhaps the…

P6; and its painful that there is no Doctor at Kareng, and that one situation where there was nothing I could do for the patient, hence the doctor understood there was nothing I could have done...

INT: Ok thank, thankyou P1, the last person?

P5: for me the problem I had happened two weeks back, when I was about to leave the clinic, then came three little girls, whom were probably under 5 years; it seemed as if they ate something poisonous. They were puking so much that i did not have any cannula. I wanted to do a concoction but there was no water! So I had to improvise by going to my place to fetch some water there. All the vehicles had come to Maun, which meant I had to call for transport. I nearly called all the vehicles within our area for transport, since almost all of them could not make it. So I had to call senior nurse s that I had a problem. I had only P50 of airtime, which was done in no time. I had to ask patients for theirs. They came around 4pm, but they were helped around 9pm at night, that's when they were taken to Letsholathebe. They were so restless, that when I realized that we do not experience nice things. But perhaps if there were resources, maybe they could have been assisted at Makakung instead of travelling, they could have benassisted at Kareng. They were admitted for almost a week. I was so scared for their life.

Inter: thank you P5, I think we have come to the end of this discussion but is there anything else that you fell we did not discuss any issue that, ok P6…

P6: I wish to add something; the other issues really are out of our control. Like the issue of resources. Maybe in the past, how many months has it been? We were turning patients away who came for CD4 because our machine was not working. Even when you at the patient you could see. But because due to things beyond our control, it end up endangering the patients.

Inter: thank you P6! Is there anything else that we have not discussed that you wish we had?We have come to the end of our discussion, is there anything that we forgot that you think we should talked about?

P2; I was saying if the government could come up with the policy of human resource particularly for stuff in the rural areas. That would help as a guide line so that things are not haphazard. I think it's the one that could differentiate between someone who works in the city and somebody who works in the rural area. Otherwise we are just under one general umbrella and if they say go to kareng, you aregone! And there is nothing that could help you, so if there could be a policy that will help.

Inter: thanks P2 is there anything else?

inter: well I guess in ending we want to say thank you for participating now as I explained at the begin this is what'sgoing to happen so we have met yesterday we met the users of health services

interestingly quite a lot of the issues you were raising they raised which is really I think is interesting and then tomorrow we are meeting your policy makers here and then we are meeting the last group on Friday now what we are going to try and do is actually to try and once we have done the three sites we have done and finished we are going to look at the results and then we are going to try find a way of actually bring together the people who participated well those who are interested in actually hearing what the final result is of all that has come out of these in the three sites and then we are going to then start and intervention the EU has given us little bit of money that we can try oneintervention during this time so really that's going to be the process you might here from us again like but as i said that will be those who are still interested so that's why we have taken your contacts but that will probably be towards the end of the year but otherwise thank you very much.
