# Supplementary material for: Stakeholders’ Perceptions on Shortage of Healthcare Workers in Primary Healthcare in Botswana: Focus Group Discussions
Source: PLoS One. 2015 Aug 18;10(8):e0135846. doi: 10.1371/journal.pone.0135846 (PMC4540466; doi:10.1371/journal.pone.0135846)
Supplement: S15 Text — (PDF) [file pone.0135846.s015.pdf]

A PARTICIPATORY QUALITATIVE STUDY OF HUMAN RESOURCES FOR PRIMARY CARE IN BOTSWANA

INTERVIEW GUIDE: Questions policy makers and healthcare professionals with prompts

Introduction:
I am going to ask you questions about the health care workers in your area. There is no right or wrong answer. Please answer each question in detail. Your truthful answer will help the ministry of health to understand the health care workers situation in the country

1. What is your understanding of Primary Healthcare?

Studies in Botswana have showed that there is a shortage of healthcare workers in primary care, especially in rural areas.

2. Do you think there are enough or not enough health care workers in Botswana? If there are not enough why do you think it is so (**probes)**

   a. Are there adequate numbers trained?
   b. What about deployment/distribution?
   c. What about retention?

3. Do you think there are gaps, issues or problems related to health care workers for primary health or not? If there are gaps/problems what do you think are the most important gaps, issues and problems? (**Probes)**

   d. Are they adequately trained for the job?
   e. Are health workers assigned jobs they adequately are trained for?
   f. Is there more shortage in certain cadres?
   g. Is there adequate support to do the job (resources, coordination and management)?
   h. What about conditions of service?

4. Is there a problem of health care workers in the rural areas? If yes why do you it is so? **(Probes)**
   a. Do living conditions in the rural areas contribute?
   b. Does remoteness contribute?
   c. What about the cost of living in rural areas?
   d. What about opportunities for continuing professional development/ further education?
   e. What about availability of jobs/schools for partners /children?

5. What do you think should be done about the lack of health workers for primary healthcare in Botswana? (**Probes)**
   a. Training (quantity and quality)
   b. Deployment/ distribution?
   c. Retention strategies?
   d. To get the right skill mix?
   e. What about task shifting?

6. In your experience, what solutions /interventions have already been tried to resolve these problems? **(Probes)**

a. Performance based reward system?
b. Moving primary care from Ministry of Local government to Ministry of Health?
c. Setting up of district management teams?
d. Introduction of Medical school programmes and faculty in rural areas and primary care?

7. Did the solutions work? Why or why not?

8. What interventions would make the biggest difference to improve primary healthcare?

Building of effective primary care team has been suggested as a potential intervention to improve primary care in Botswana.

9. What is your understanding of effective primary care team? (**Probes**)
    a. What is the value of primary care team?
    b. Who should be the members of an effective primary care team?
    c. What should the roles of the different members be?
    d. Who should lead the team?
    e. How should the quality of the team work be evaluated and the impact its work evaluated?

10. What is your opinion about building primary care team as one of the interventions to improve primary care in Botswana?

Healthcare workers ONLY:

11. Do you remember any problematic situation in your work, you found particularly morally difficult? If yes.
    (a) Can you tell us about this situation, about what happened? **(probes)**
        i. Turning patients away without treatment?
        ii. Making patients wait too long?
    b. Why was it difficult?

Policy makers only:

12. Can you tell me what kind of ethically or morally challenging issues you had to tackle in your function? **(probes)**
    a. In your opinion, what are the major issues?
        i. Allocation of resources?
        ii. Transfer of health workers?
    b. Why is it difficult?

13. In conclusion, is there anything else we haven't discussed that you wish we had?
